# Regiodivergent Deuteration of Pyridine-Based Heterocycles

**DOI:** 10.1021/acs.orglett.5c02961

**Published:** 2025-10-06

**Authors:** Wei Du, Santosh C. Gadekar, Álvaro Velasco-Rubio, Jesus Rodrigalvarez, Ruben Martin

**Affiliations:** † 202569 Institute of Chemical Research of Catalonia (ICIQ), The Barcelona Institute of Science and Technology, Av. Països Catalans 16, 43007 Tarragona, Spain; ‡ Universitat Rovira i Virgili, Departament de Química Orgànica, 43007 Tarragona, Spain; § ICREA, Passeig Lluís Companys, 23, 08010, Barcelona, Spain

## Abstract

Herein, we describe a regiodivergent deuteration of pyridotriazoles under mild conditions. Site-selective deuteration can be tuned, controlled, and switched by a subtle interplay of the base and solvent utilized. Given the ease at which pyridotriazoles can be converted into a variety of pyridine congeners, this protocol might offer a new gateway to access a variety of deuterium-labeled pyridine-containing heterocycles in a site-selective, yet predictable, manner.

## Graphical abstract

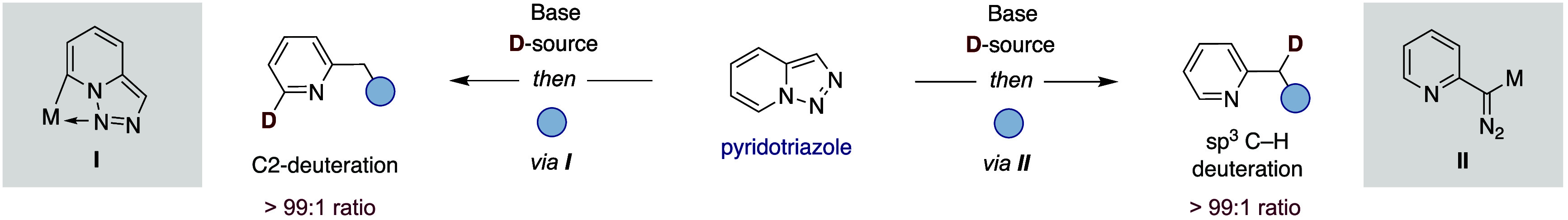

Deuterium labeling is becoming increasingly important in drug discovery.^1^ Indeed, the incorporation of deuterium atoms alters the physical and chemical properties of the parent compound, increasing selectivity, metabolic stability and potency of drug candidates.^2^ Driven by the prevalence of azines in marketed drugs (Scheme 1, *top*),^3^ chemists have been challenged to introduce deuterium atoms at pyridine backbones with improved modularity and site-selectivity.^4^ Despite the advances realized with transition metal catalysts,^5–11^ supercritical fluids (SCF),^12^ electrochemical settings^13^ or by leveraging the potential of phosphonium salts for C4-deuteration,^14^ a site-selective C2-deuteration of pyridines still remains a particularly unexplored endeavor (*bottom right*).^15^

**Scheme 1:**
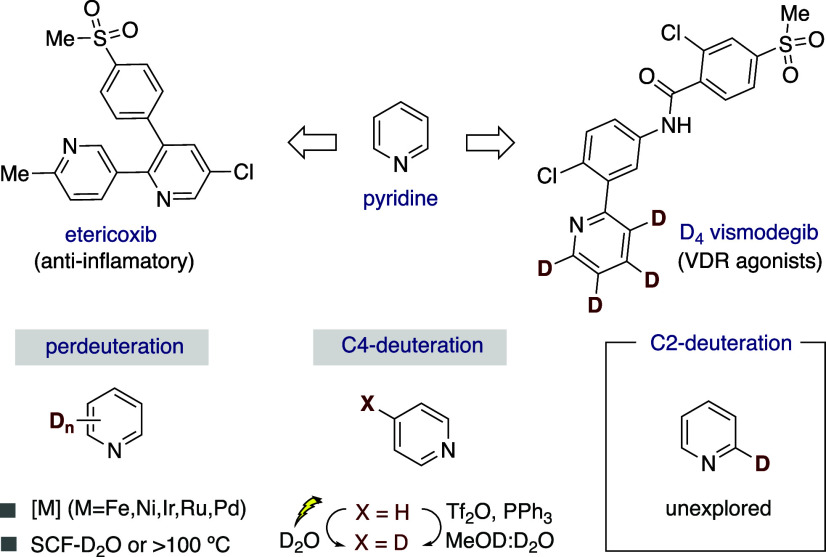
Deuterium Labelling and Pyridine Architectures

A close inspection into the literature data reveals that pyridotriazoles **1** can serve as “*masked*” pyridines due to their ability to undergo ring–chain isomerization via diazo intermediates and subsequent denitrogenative events (Scheme 2).^16^ Given that pyridotriazoles **1** are readily available either through reaction of pyridine-2-yl acetates with benzenesulfonyl azides or oxidative *N–N* coupling of hydrazones from ketones,^17^ we envisioned that a site-selective deuteration of these heterocycles might provide a new entry point to access isotopically labeled pyridines at later stages. We anticipated that regiodivergent deuteration could be dictated by a subtle interplay of electronic effects and site-selective deprotonation at the pyridotriazole core, either favoring metalation at C7 via **I** (Scheme 2, *left*) or leveraging a ring–chain isomerism en route to **II** (Scheme 2, *right*). If successful, such a regiodivergent scenario might not only offer new opportunities in isotope labeling but also overcome existing limitations when selectively incorporating deuterium atoms in pyridine-based heterocycles. As part of our interest in site-selective functionalization of heterocycles,^18^ we report the successful realization of this goal. The protocol is distinguished by its broad applicability and an exquisite, yet predictable, site-selectivity pattern, thus setting the scene for postmodifications that enable access to a variety of differently substituted deuterated pyridine-type heterocycles.

**Scheme 2:**
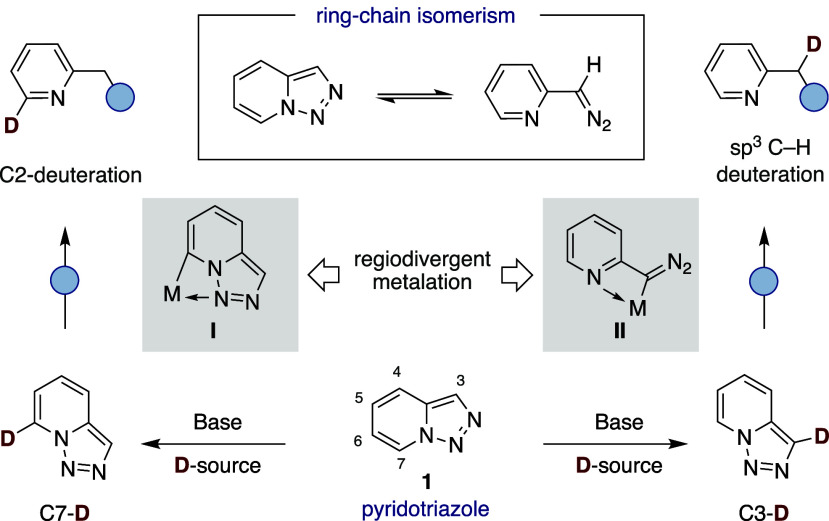
Regiodivergent Deuteration of Pyridotriazoles

We began our work by evaluating the deuteration of **1a** (Table 1), easily accessed in three steps from pyridine on a large scale.^17c^ After some experimentation,^19^ we found that the utilization of LiO*t*Bu (0.40 equiv) and CD_3_CN (12 equiv) in 1,4-dioxane (0.20 M) at rt resulted in **2a** with 95% deuterium content and excellent site-selectivity (**2a:3a** = 95:5). Interestingly, we noticed a strong solvent effect on selectivity; while nonethereal solvents provided traces, if any, of **2a** (entries 6 and 7), an erosion in site-selectivity was observed with THF, DME or *i*Pr_2_O (entries 7–9). In addition, lower selectivities were found with DMSO-*d*_6_ as the deuterium source (entry 11). Equally striking was the influence of the base and the escorting counterion. Specifically, statistical mixtures of **2a** and **3a** were found when employing NaO*t*Bu or KO*t*Bu (entries 2 and 3) whereas a 2.5:1 ratio of **2a:3a** was observed for LiOMe (entry 4), revealing a noninnocent behavior exerted by both countercation and counterion in both reactivity and site-selectivity. In addition, weaker bases such as Li_2_CO_3_ or Li_3_PO_4_ resulted in no conversion of **1a** to either **2a** or **3a** (entry 5). It is worth noting that *n*BuLi in THF at −78 °C followed by D_2_O quench resulted in exclusive C7-deuteration, thus showing the subtleties of controlling deuteration at C3 (**2a**) or C7 (**3a**).^20^

**Table 1:**
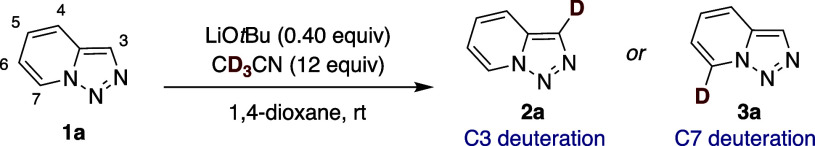

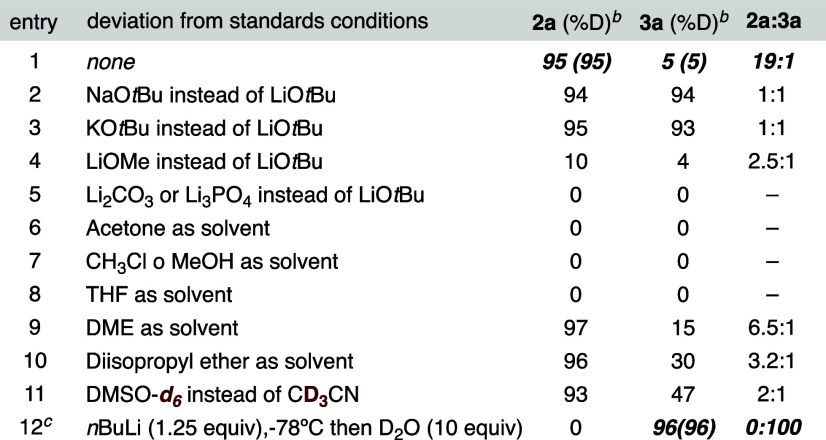
Optimization of the Reaction Conditions^a^

a **1a** (0.20 mmol), LiO*t*Bu (0.08 mmol), CD_3_CN (4 mmol) in 1,4 dioxane (0.20 M) at rt for 17 h.

b D-incorporation calculated by ^1^H NMR of the crude mixture. % D-content in the isolated product shown in parentheses.

c *n*BuLi (0.25 mmol) in THF (0.4 M) for 20 min (−78 °C), then D_2_O (2 mmol) at rt, 1h.

Intrigued by the results compiled in Table 1, we wondered whether aggregation of the metal cation in different ethereal solvents might be responsible for the observed site-selectivity.^21^ To this end, we turned our attention to study the diffusion coefficients of the intermediate species in the reaction of **1a** and MO*t*Bu (M = Li, K) in THF-*d*_8_ or 1,4-dioxane-*d*_8_ by Diffusion-Ordered NMR Spectroscopy (DOSY).^22^ Intriguingly, a different scenario was observed depending on the nature of the base utilized. Specifically, a high diffusion coefficient (MW_det_ = 114 g·mol^–1^; *D* = 17.31·10^–10^ m^2^·s^–1^) was observed with LiO*t*Bu (Scheme 2, *top left*). In sharp contrast, the lower diffusion coefficient found with KO*t*Bu (MW_det_ = 1980 g·mol^–1^; *D* = 5 × 10^–10^ m^2^·s^–1^) suggests the intervention of aggregated species (*top right*) due to the larger size of the ionic radius of K^+^ (1.38 Å) when compared to its Li analogue (0.76 Å).^23^ Putting these results into perspective, we concluded that aggregation has a negative impact on selectivity and that solvated ion pairs might be responsible for C3-selectivity en route to **2a**.^19,21^

Aiming at shedding light on the origin of site-selectivity, we wondered whether a subtle interplay between the p*K*_a_ values at C3 and C7 in the pyridazole core and its proclivity for dynamic ring–chain isomerism en route to 2-diazomethylpyridines might come into play (Scheme 3, *bottom*). To this end, we turned our attention to DFT calculations. Although the calculated p*K*_a_ values at the **1a** core argued against a preferential metalation at C3 (p*K*_a_ = 34.9) vs C7 (p*K*_a_ = 28.3), the higher acidity of H_c_ in **4a** (p*K*_a_ = 17.4) suggested that C3-selectivity might be attributed to the ring–chain isomerism of the pyridazole core. Interestingly, DFT calculations revealed that the equilibrium of **1** and **4** was heavily influenced by the nature of the C5 substituent at the pyridotriazole core.^19^ Indeed, **1a** was favored over **4a** (population ratio of **1a:4a** = 1784:1) whereas the inclusion of electron-donating groups at C5 resulted in a markedly lower **1:4** ratio. In light of these results, we tentatively propose a canonical Curtin–Hammett scenario,^24^ with product formation not arising from the equilibrium distribution of **1** and **4**, but rather by a preferential deprotonation at H_c_ with weaker bases such as LiO*t*Bu, thus leading to C3-selectivity. In contrast, the utilization of *n*BuLi (p*K*_a_ = 50) might result in a deprotonation at the most acidic H_a_ prior to ring–chain isomerism, thus ultimately leading to **I** (Scheme 2) where the lithium cation is stabilized by the lone pair of the adjacent nitrogen atom at the pyridazole core.^25,26^

**Scheme 3:**
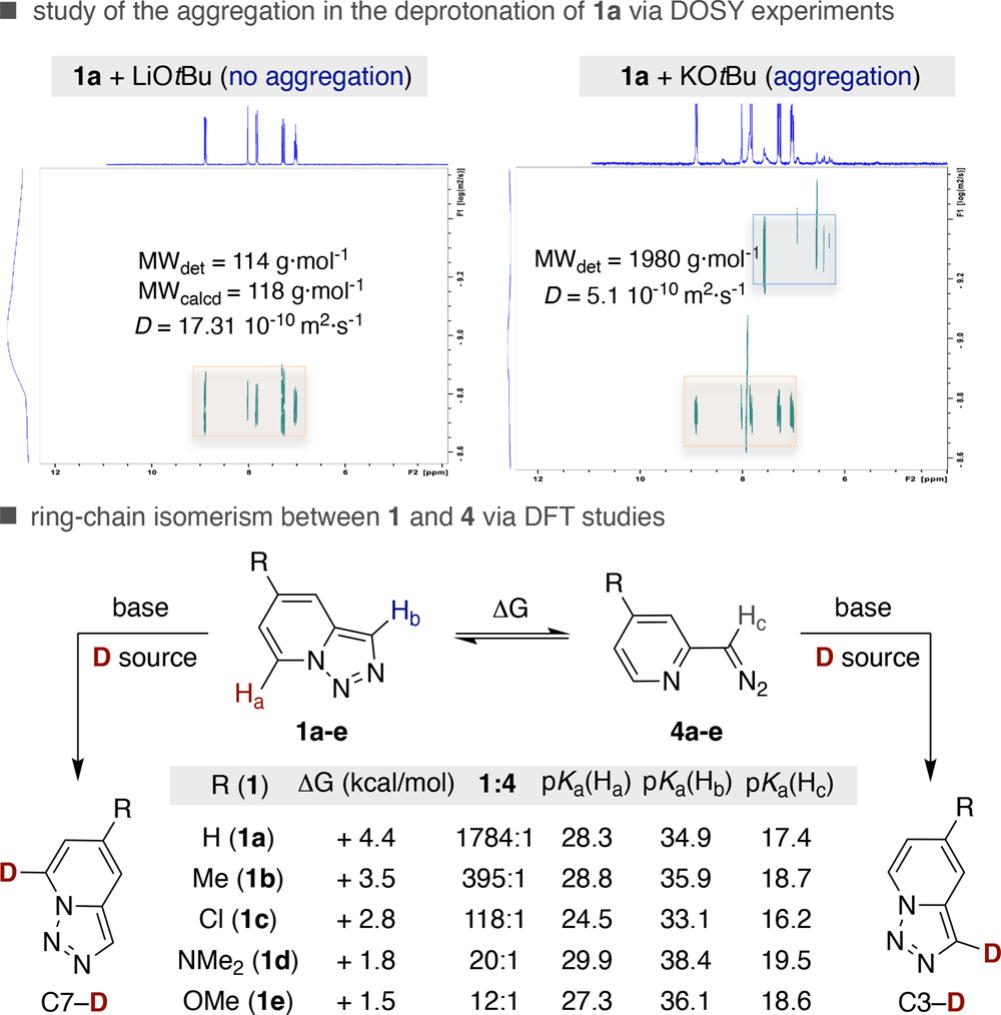
DFT Studies and Aggregation Scenarios

With the optimized conditions in hand, we turned our attention to exploring the generality of our site-selective deuteration in a variety of substituted pyridotriazole motifs (Table 2). As shown, site-selective C3-deuteration could be applied across a wide number of substituents at the pyridotriazole core, including alkyl groups (**2b**, **2c**), electron-donating motifs (**2d**, **2e**) or even in the presence of acyclic or cyclic amines (**2f**, **2g**, **2h**). Likewise, the inclusion of arenes at the pyridotriazole core did not interfere with the site-selective incorporation of deuterium at C3 (**2i**–**2l**). Even heterocyclic rings such as pyridine (**2m**), indole (**2n**) or dibenzothiophene (**2o**) could be employed with similar ease, resulting in high selectivities for C3-deuteration. Note, however, that pyridotriazoles bearing electron-withdrawing groups such as chlorine (**2s**) or amides (**2t**) resulted in a non-negligible erosion in site-selectivity. This result can tentatively be ascribed to the lower acidity at C7 when the pyridotriazole is decorated with electron-withdrawing groups (Scheme 3, **1c**; p*K*_a_ H_a_ = 24.5).^19^ As for site-selective C7-deuteration with an *n*BuLi regime, the reaction turned out to be widely applicable to a variety of substrates bearing either alkyl or arene substituents at the pyridotriazole core. Exclusive C7-selectivity was observed in most cases under the standard conditions regardless of the nature of the substituents at the pyridotriazole core. Unlike the slight erosion in C3-selectivity found for a protocol based on LiO*t*Bu, nitrogen-containing heterocycles such as indole (**3n**), pyridine (**3m**) or dibenzothiophene (**3o**) resulted in an exquisite C7-deuteration under an *n*BuLi regime. Given that triazolopyridines serve as an entry point to access a variety of pyridyl-containing heterocycles via ring–chain isomerism,^16^ we focused our attention on the versatility of **2a** and **3a** as vehicles for downstream applications (Scheme 4). As shown, pyridoquinolinones were easily within reach by reaction of **2a** or **3a** with 2-formylphenyl trifluoromethanesulfonate (**5**) under a protocol based on Pd/DPEPhos,^27^ giving access to the targeted compounds in high yields and with high deuterium content at C11 (**6**) or C4 (**7**). Likewise, simple exposure of **2a** or **3a** to AcOH at 100 °C resulted in **8** or **9** in excellent yields.^28^ In addition, reaction of **2a** or **3a** with phenyl boronic acid in 1,4-dioxane resulted in **10** and **11**, thus allowing an effective incorporation of an aromatic ring at the benzylic sp^3^ C–H site.^29,30^

**Table 2:**
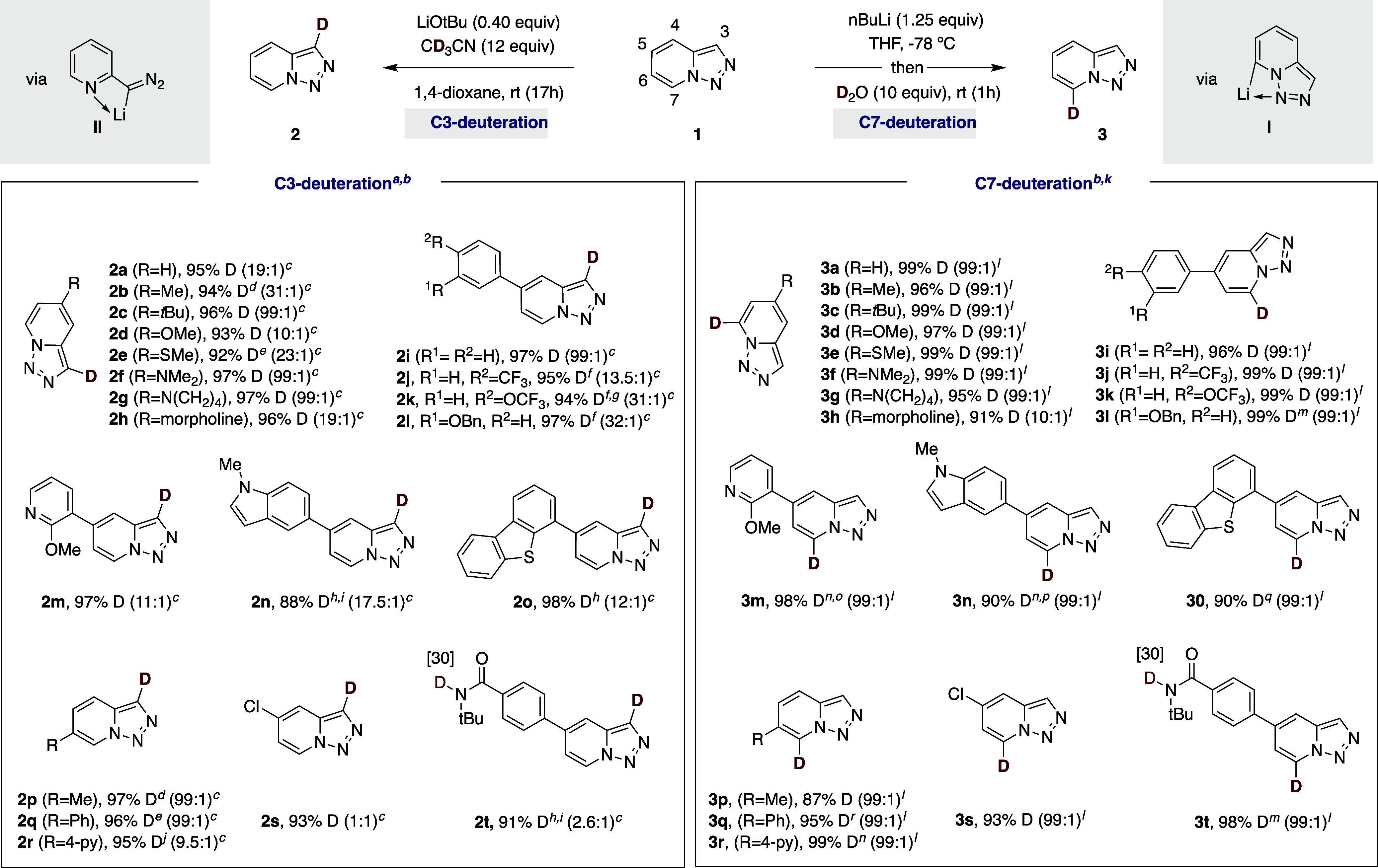
Site-Selective C3 and C7-Deuteration of Pyridotriazoles

a Conditions: **1** (0.20 mmol), LiO*t*Bu (0.4 equiv), CD_3_CN (12 equiv), 1,4-Dioxane (0.2 M), rt, 17 h.

b The majority of products were obtained in >95% yield, see Supporting Information for details.

c C3:C7 ratio.

d LiO*t*Bu (1.0 equiv).

e 8 h reaction time.

f LiO*t*Bu (0.6 equiv).

g 4 h reaction time.

h LiO*t*Bu (0.8 equiv).

i 40 °C.

j 1,4-Dioxane (0.05 M) as solvent.

k *n*BuLi (1.25 equiv), THF (0.4 M), −78 °C, 20 min; then D_2_O (10 equiv), −78 °C, 10 min; then 1h, rt.

l C7:C3 ratio.

m *n*BuLi (2.0 equiv).

n **1** (0.10 mmol).

o THF (0.03 M).

p THF (0.025 M).

q *n*BuLi (1.7 equiv).

r THF (0.08 M).

**Scheme 4:**
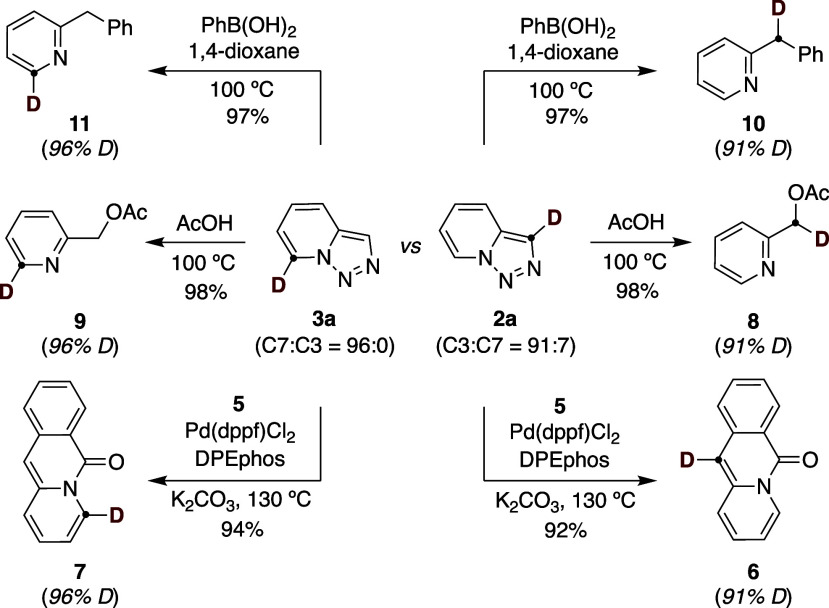
Applicability of D-Labelled Pyridotriazoles

Encouraged by the results shown in Table 2, we wondered whether we could apply an otherwise similar site-selective deuterated scenario to nitrogen-containing heterocycles other than triazolopyridines. As shown in Scheme 5, this turned out to be the case and a site-selective deuteration could also be within reach with pyrazolopyridines with similar ease.^31^ Specifically, C3-deuteration of **12** could be accomplished with catalytic amounts of trifluoroacetic acid (TFA) and D_2_O in MeCN en route to **13** (>99:1)^32^ whereas a site-selectivity switch was found by exposure to KO*t*Bu (50 mol %) and CD_3_CN in 1,4-dioxane, leading to **14** (up to 99:1) with high deuterium content. These conditions were found to be widely applicable across a number of aryl-substituted pyrazolopyridines, resulting in either **13a**–**e** or **14a**–**e** with an exquisite site-selectivity pattern (>99:1) and deuterium content (>92%). Site-selectivity in the former can be interpreted on the basis of an initial protonolysis of the pyrazolopyridine ring (**III**) prior to site-selective reaction with D_2_O at C3,^32^ whereas regioselective metalation at the heterocyclic ring preferentially occurs at the most acidic C7–H bond via **IV**.^33^

**Scheme 5:**
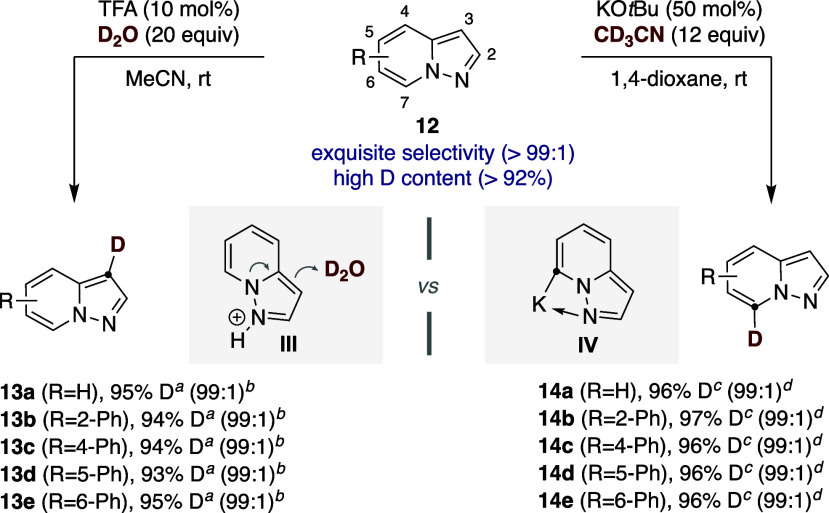
C3 and C7-Deuteration of Pyrazolopyridines

a Conditions: **12** (0.2 mmol), TFA (0.1 equiv), D_2_O (20 equiv), MeCN (0.2 M), rt, 17 h.
b C3:C7 ratio.
c Conditions: **12** (0.2 mmol), KO*t*Bu (0.5 equiv), CD_3_CN (12 equiv), 1,4-Dioxane (0.2 M), rt, 17 h
d C7:C3 ratio. The majority of products were obtained in >95% yield, see Supporting Information for details.

In summary, we have developed a regiodivergent deuteration of pyridotriazoles under mild conditions. Site-selectivity can be dictated by a subtle modulation of the ring–chain isomerism, where the nature of the base and the solvent utilized has a profound impact on the reaction outcome. Given the versatility of pyridotriazoles as linchpins for further functionalization and the extension to pyrazolopyridines, we believe the method might hold promise to offer a new gateway to access a variety of deuterium-labeled pyridine-containing heterocycles in a site-selective, yet predictable, manner.

## Supplementary Material



## Data Availability

The data underlying this study are available in the published article and its Supporting Information.

## Notes

Detailed experimental procedures, characterization data, DFT Calculations and NMR spectra for all new compounds (PDF)
