# Supplementary material for: Regiodivergent Deuteration of Pyridine-Based Heterocycles
Source: Org Lett. 2025 Oct 6;27(41):11428–33. doi: 10.1021/acs.orglett.5c02961 (PMC12538594; doi:10.1021/acs.orglett.5c02961)
Supplement: Supplementary file 1 [file ol5c02961_si_001.pdf]

Supporting Information

Regiodivergent Deuteration of Pyridine-Based Heterocycles

Wei Du,[#§‡] Santosh C. Gadekar,[#‡] Álvaro Velasco-Rubio,[#] Jesus Rodrigalvarez[#] and Ruben Martin*[#†]

rmartinromo@iciq.es

[#] *Institute of Chemical Research of Catalonia (ICIQ), The Barcelona Institute of Science and Technology, Av. Països Catalans 16, 43007 Tarragona, Spain*

[§] *Universitat Rovira i Virgili, Departament de Química Orgànica, 43007 Tarragona, Spain.*

[†] *ICREA, Passeig Lluís Companys, 23, 08010, Barcelona, Spain*

General information **3**

List of starting materials **5**

Synthesis of the starting materials **6**

Synthesis of [1,2,3]triazolo[1,5-a]pyridines **10**

Optimization of the C3-Deuteration of [1,2,3]triazolo[1,5-a]pyridine 1a **18**

Optimization of the C3-Deuteration of pyrazolo[1,5-a]pyridine 12a **20**

Optimization of the C7-Deuteration of pyrazolo[1,5-a]pyridine 12a **21**

Selective C3-Deuteration of [1,2,3]triazolo[1,5-a]pyridine **22**

Selective C7-Deuteration of [1,2,3]triazolo[1,5-a]pyridine **33**

Unsuccessful substrates **44**

Scale-up and Synthetic applications **44**

Selective C3-Deuteration of pyrazolo[1,5-a]pyridine **53**

Selective C7-Deuteration of pyrazolo[1,5-a]pyridine **55**

Mechanistic Investigations 58

Kinetic Isotope Effect (KIE) 58

Hammett-Plot experiments 61

Diffusion-Ordered NMR Spectroscopy (DOSY) Experiments 62

DFT Calculations 67

References 78

NMR Spectra for Known Compounds 80

NMR Spectra for Unknown Compounds 85

General information

Analytical methods: ^{1}H and ^{13}C NMR spectra were recorded on Bruker 400 MHz and Bruker 500 MHz at 20 °C. All ^{1}H and ^{13}C NMR spectra are reported in parts per million (ppm) downfield of TMS and were calibrated using the corresponding residual solvent peak. Coupling constants, *J*, are reported in Hertz. Melting points were measured using open glass capillaries in a Büchi B540 apparatus, with samples unless otherwise stated recrystallized by slow evaporation of a solution of DCM. Infrared spectra (FT-IR) measurements were carried out on a Bruker Optics FT-IR Alpha spectrometer equipped with a DTGS detector, KBr beamsplitter at 4 cm^{-1} resolution using a one bounce ATR accessory with diamond windows. Mass spectra were recorded on a Waters LCT Premier spectrometer or in a Micro TOF Focus, Bruker Daltonics spectrometer. Flash chromatography was performed with Sigma-Aldrich silica gel, pore size 60 Å (230-400 mesh). TLC silica gel F254 aluminum sheets from Sigma-Aldrich were used with visualization through UV irradiation. The isolated yields reported represent an average of two independent runs.

Reagents: Unless otherwise noted, all materials were used from commercial sources without further purification. LiO*t*-Bu was purchased from Aldrich and stored in a nitrogen-filled Glovebox. KO*t*-Bu was purchased from Thermo Scientific and stored in a nitrogen-filled Glovebox. *n*-BuLi (2.5 M in hexanes) was purchased from Sigma-Aldrich and stored in fridge. Trifluoroacetic acid (TFA) was purchased from Sigma-Aldrich. CD_3CN (≥ 99.8 atom % D) was purchased from Sigma Aldrich, degassed and stored in Schlenk tube over molecular sieves. D_2O (99 atom % D) was purchased from Aldrich, degassed and stored in Schlenk tube. 1,4-dioxane (anhydrous, 99.5%) was purchased from Thermo Scientific over molecular sieves. THF (anhydrous, 99.5%) was purchased from Thermo Scientific, degassed and stored in Schlenk tube over molecular sieves.

Structural assignments were made with additional information from DOSY experiments.

Calculation of deuterium incorporation: Unless otherwise noted, %Deuterium incorporations are shown. Deuterium incorporation was determined by ^{1}H NMR, based on comparison with non-deuterated C4–H sites by applying the following equation: $\%D\ incorporation\ = \left(1 - \frac{IntegralC3}{IntegralC4}\right)\ x100$ (see an example below).

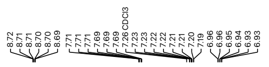

$$\%D = \left(1 - \frac{0.05}{1}\right) \mathrm{x}\ 100 = 95\%\ \text{D incorporation}$$

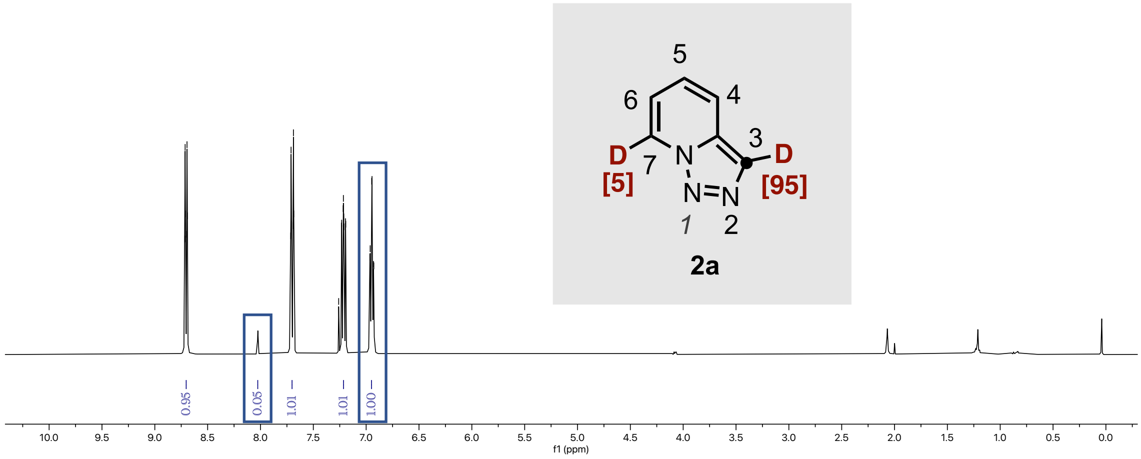

List of starting materials

Commercially available compound **12a** was purchased from Apollo Scientific and used as received without further purification. Compounds **1a**,[13] **1b**,[14] **1d**,[15] **1h**,[15] **1p**,[15] **1s**,[14] **12b**,[16] **12d**,[17] and **12e**[18] were prepared according to literature procedures. All the starting materials are listed below:

[1,2,3]triazolo[1,5-a]pyridine

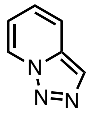

1a

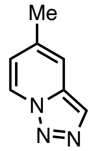

1b

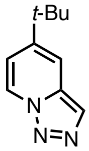

1c

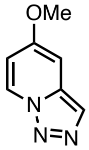

1d

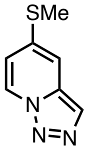

1e

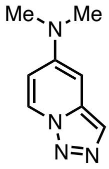

1f

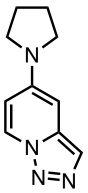

1g

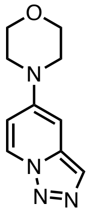

1h

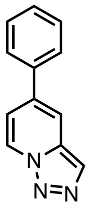

1i

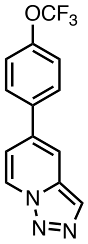

1j

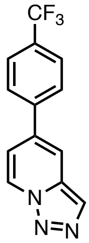

1k

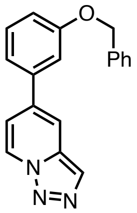

1l

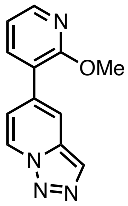

1m

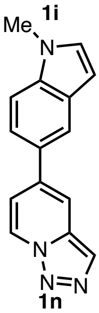

1n

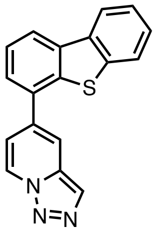

1o

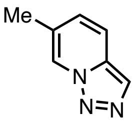

1p

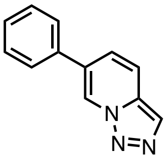

1q

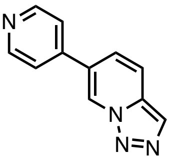

1r

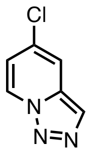

1s

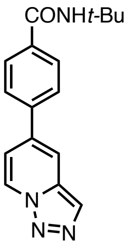

1t

pyrazolo[1,5-a]pyridine

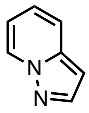

12a

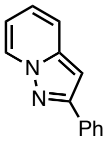

12b

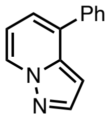

12c

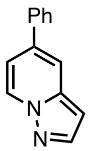

12d

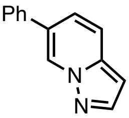

12e

Synthesis of the starting materials

Commercially available compounds (**S1**, **S2**, **S3**, **S17**) were used as received without further purification. Compounds **S4**,[1] **S5–S8**,[2] **S9**,[3] **S10**,[4] **S11**,[5] **S18**[6] and **S19**[7] were prepared according to literature procedures.

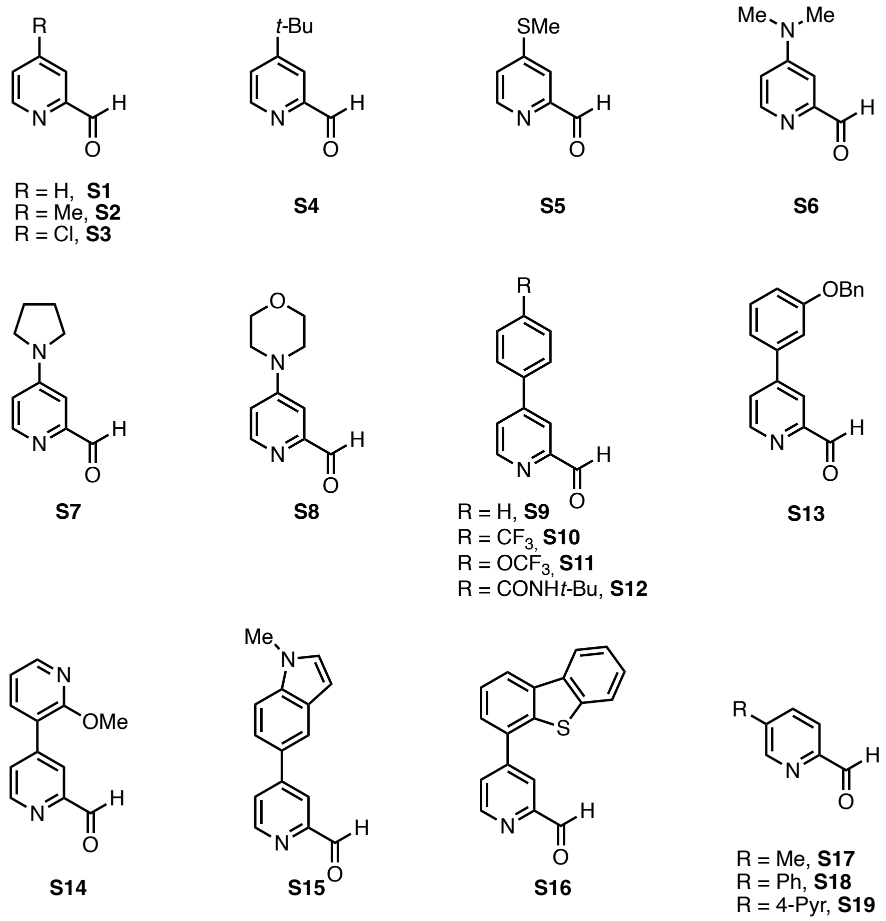

Figure S1. List of picolinaldehydes

Synthesis of picolinaldehydes S12-S16

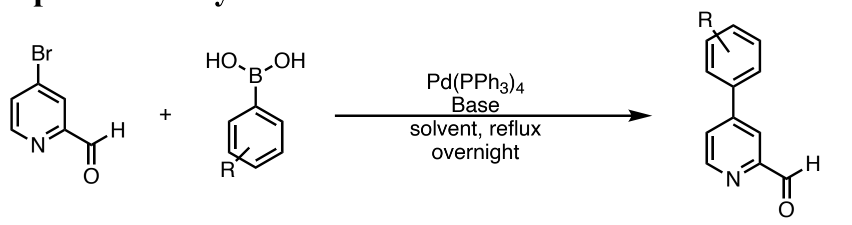

S12–S16

General Procedure A: An oven dried three-neck flask equipped with a stirring magnetic bar was charged with 4-bromopicolinaldehyde, phenyl boronic acid, $Pd(PPh_3)_4$ and solvent. Afterwards, base (or aqueous solution of the base) was added, and the reaction mixture was degassed by bubbling argon for 30 min. Then, the reaction was stirred at reflux in an oil bath overnight. Afterwards, the reaction was cooled down to rt, quenched

with water and extracted with EtOAc. The solvent removed under vacuum and the crude was purified by flash column chromatography to give the desired of picolinaldehydes.

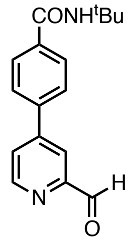

***N*-(*tert*-butyl)-4-(2-formylpyridin-4-yl)benzamide** (**S12**).[8] Following general procedure A, utilizing 4-bromopicolinaldehyde (1.86 g, 10.0 mmol), (4-(*tert*-butylcarbamoyl)phenyl)boronic acid (2.2 g, 10.0 mmol, 1.0 equiv), $Pd(PPh_3)_4$ (580.0 mg, 0.5 mmol, 0.05 equiv), 1,4-dioxane (40.0 mL) and a solution of Cs_2CO_3 (8.15 g, 25.0 mmol, 2.5 equiv) in H_2O (4.0 mL). The reaction was purified by flash column chromatography on silica gel (20% EtOAc in hexanes to 50% EtOAc in hexanes) to provide **S12** as white solid (2.2g, 0.78 mmol, 78% yield). M.P.: 133°C– 137°C. ^{1}H NMR (500 MHz, $CDCl_3$) δ 10.14 (s, 1H), 8.85 (dd, *J* = 5.1, 0.8 Hz, 1H), 8.18 (dd, *J* = 1.9, 0.8 Hz, 1H), 7.89 – 7.82 (m, 2H), 7.77 – 7.69 (m, 3H), 6.02 (s, 1H), 1.50 (s, 9H). ^{13}C NMR (126 MHz, $CDCl_3$) δ 193.5, 166.1, 153.6, 151.0, 148.7, 139.6, 137.1, 127.9(2C), 127.3(2C), 125.7, 119.6, 52.0, 29.0(3C). IR (neat): 3306, 3062, 1701, 1635, 1541, 1314, 1220, 994, 768, 738, 645 cm^{-1}. HRMS (ESI) m/z: $[M+H]^+$ Calcd for $C_{17}H_{19}N_2O_2$ 283.1447; Found 283.1437.

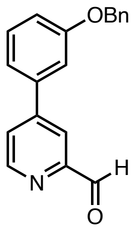

4-(3-(benzyloxy)phenyl)picolinaldehyde (S13).[9] Following general procedure A, utilizing 4-bromopicolinaldehyde (1.86 g, 10.0 mmol, 1.0 equiv), (3-(benzyloxy)phenyl)boronic acid (2.74 g, 12.0 mmol, 1.2 equiv), $Pd(PPh_3)_4$ (0.57 g, 0.5 mmol, 0.05 equiv), toluene (19 mL) and a solution of Na_2CO_3 (0.32 g, 3.0 mmol, 0.3 equiv) in water (0.2 mL). The reaction was purified by flash column chromatography on silica gel (20% EtOAc in hexanes to 50% EtOAc in hexanes) to provide **S13** as white solid (0.67g, 0.23 mmol, 23% yield). M.P.: 83°C– 85°C. ^{1}H NMR (500 MHz, $CDCl_3$) δ 10.16 (s, 1H), 8.82 (d, *J* = 5.2 Hz, 1H), 8.19 (dd, *J* = 2.0, 0.8 Hz, 1H), 7.73 (dd, *J* = 5.1,

1.9 Hz, 1H), 7.49 – 7.37 (m, 5H), 7.38 – 7.32 (m, 1H), 7.31 – 7.27 (m, 2H), 7.11 – 7.08 (m, 1H), 5.15 (s, 2H). ^{13}C NMR (126 MHz, $CDCl_3$) δ 193.4, 159.5, 153.2, 150.5, 149.7, 138.4, 136.6, 130.5, 128.7(2C), 128.2, 127.5(2C), 125.6, 119.7, 119.6, 116.0, 113.9, 70.3. IR (neat): 3089, 2922, 2831, 1708, 1596, 1453, 1290, 1175, 1024, 854, 789, 739 cm^{-1}. HRMS (ESI) m/z: $[M+H]^+$ Calcd for $C_{19}H_{16}NO_2$ 290.1181; Found 290.1187.

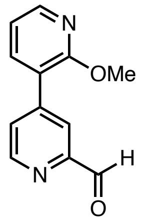

2-methoxy-[3,4'-bipyridine]-2'-carbaldehyde (S14).[10] Following general procedure A, utilizing 4-bromopicolinaldehyde (1.86 g, 10.0 mmol, 1.0 equiv), (2-methoxypyridin-3-yl)boronic acid (1.84 g, 12.0 mmol, 1.2 equiv), $Pd(PPh_3)_4$ (1.20 g, 1.00 mmol, 0.1 equiv), 1,4-dioxane (100 mL) and a solution of K_2CO_3 (4.0 g, 30.0 mmol, 3.0 equiv) in water (25 mL). The reaction was purified by flash column chromatography on silica gel (20% EtOAc in hexanes to 50% EtOAc in hexanes) to provide **S14** as white solid (1.1 g, 5.0 mmol, 50% yield). M.P.: 133°C– 139°C. ^{1}H NMR (500 MHz, $CDCl_3$) δ 10.11 (s, 1H), 8.80 (dd, J = 5.1, 0.8 Hz, 1H), 8.24 (dd, J = 5.0, 1.9 Hz, 1H), 8.15 (dd, J = 1.9, 0.8 Hz, 1H), 7.76 (dd, J = 5.1, 1.8 Hz, 1H), 7.70 (dd, J = 7.4, 1.9 Hz, 1H), 7.02 (dd, J = 7.4, 5.0 Hz, 1H), 3.98 (s, 3H). ^{13}C NMR (126 MHz, $CDCl_3$) δ 193.6, 160.8, 153.1, 150.2, 148.1, 146.0, 138.7, 127.9, 121.8, 120.7, 117.5, 53.9. IR (neat): 3018, 2961, 2809, 2714, 1714, 1579, 1465, 1401, 1263, 1183, 1007, 868, 825, 798, 771 cm^{-1}. HRMS (ESI) m/z: $[M+H]^+$ Calcd for $C_{12}H_{11}N_2O_2$ 215.0821; Found 215.0821.

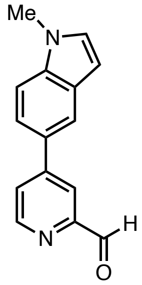

4-(1-methyl-1*H*-indol-5-yl)picolinaldehyde (S15).[11] Following general procedure A, utilizing 4-bromopicolinaldehyde (3.80 g, 20.0 mmol, 1.0 equiv), (1-methyl-1*H*-indol-5-yl)boronic acid (5.2 g, 30.0 mmol, 1.5 equiv), $Pd(PPh_3)_4$ (1.2 g, 1.0 mmol, 0.05 equiv), 1,4-dioxane (50 mL) and a solution of K_2CO_3 (5.6 g, 40.0 mmol, 2.0 equiv) in water (10.0 mL). The reaction was purified by flash column chromatography on silica gel (20%

EtOAc in hexanes to 50% EtOAc in hexanes) to provide **S15** as yellow solid (4.27 g, 18.0 mmol, 90% yield). M.P.: 74°C– 78°C. ^{1}H NMR (500 MHz, $CDCl_3$) δ 10.15 (s, 1H), 8.77 (dd, J = 5.1, 0.8 Hz, 1H), 8.25 (dd, J = 2.0, 0.8 Hz, 1H), 7.98 (dd, J = 1.8, 0.7 Hz, 1H), 7.78 (dd, J = 5.1, 2.0 Hz, 1H), 7.55 (dd, J = 8.5, 1.8 Hz, 1H), 7.42 (dt, J = 8.6, 0.8 Hz, 1H), 7.12 (d, J = 3.1 Hz, 1H), 6.57 (dd, J = 3.1, 0.9 Hz, 1H), 3.83 (s, 3H). ^{13}C NMR (126 MHz, $CDCl_3$) δ 193.9, 153.3, 151.1, 150.6, 137.5, 130.4, 129.2, 128.2, 125.5, 120.7, 120.0, 119.6, 110.2, 102.0, 33.1(3C). IR (neat): 3053, 2916, 2808, 2710, 1702, 1592, 1470, 1338, 1199, 844, 805, 725, 658 cm^{-1}. HRMS (ESI) m/z: [M+H]$^+$ Calcd for $C_{15}H_{13}N_2O$ 237.1028; Found 237.1032.

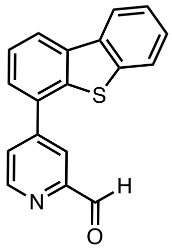

4-(dibenzo[*b*,*d*]thiophen-4-yl)picolinaldehyde (S16).[12] Following general procedure A, utilizing 4-bromopicolinaldehyde (3.29 g, 17.7 mmol, 1.0 equiv), dibenzo[*b*,*d*]thiophen-4-ylboronic acid (4.84 g, 21.2 mmol, 1.2 equiv), CsF (8.07 g, 53.1 mmol, 3.0 equiv), $Pd(PPh_3)_4$ (0.82 g, 1.0 mmol, 0.04 equiv) and 1,2-dimethoxyethane (90 mL). The reaction was purified by flash column chromatography on silica gel (20% Et_2O in DCM to 50% Et_2O in DCM) to provide **S16** as yellow solid (3.0 g, 9.9 mmol, 56% yield). M.P.: 154°C–156°C. ^{1}H NMR (500 MHz, $CDCl_3$) δ 10.20 (s, 1H), 8.93 (dd, J = 5.0, 0.8 Hz, 1H), 8.34 (dd, J = 1.9, 0.8 Hz, 1H), 8.24 (dd, J = 7.8, 1.2 Hz, 1H), 8.22 – 8.18 (m, 1H), 7.94 (dd, J = 5.0, 1.9 Hz, 1H), 7.87 – 7.83 (m, 1H), 7.61 (t, J = 7.6 Hz, 1H), 7.55 (dd, J = 7.4, 1.2 Hz, 1H), 7.53 – 7.47 (m, 2H). ^{13}C NMR (126 MHz, $CDCl_3$) δ 193.4, 153.6, 150.9, 149.6, 139.1, 138.2, 137.0, 135.5, 133.0, 127.5, 127.1, 126.9, 125.5, 125.0, 122.8, 122.5, 122.0, 121.1. IR (neat): 3056, 2809, 2704, 1710, 1579, 1470, 1382, 1200, 991, 864, 744, 614 cm^{-1}. HRMS (ESI) m/z: [M+H]$^+$ Calcd for $C_{18}H_{12}NOS$ 290.0640; Found 290.0627.

Synthesis of [1,2,3]triazolo[1,5-*a*]pyridines

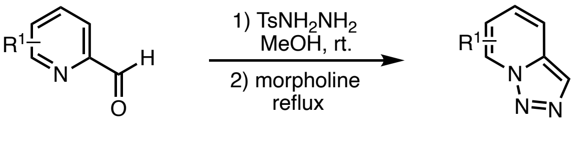

General Procedure B: To a mixture of *p*-toluenesulfonylhydrazide (1.1 equiv) in MeOH was added the substituted picolinaldehyde (1.0 equiv). The reaction was stirred for 1h at rt, and all volatiles were removed under vacuum. The tosylhydrazone obtained was dissolved in morpholine and the mixture was stirred at 100 °C in an oil bath overnight. Afterwards, the volatiles were removed in vacuo, and diethyl ether was added to the crude of the reaction. The resulting mixture was filtered, and the solvent removed under vacuum. The crude was purified by flash column chromatography to give the desired triazolopyridines **1**.

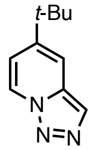

5-(tert-butyl)-[1,2,3]triazolo[1,5-a]pyridine (1c). Following general procedure B, utilizing 4-(tert-Butyl)picolinaldehyde **S4** (1.50 g, 9.2 mmol, 1.0 equiv), *p*-toluenesulfonylhydrazide (1.86 g, 10.1 mmol, 1.1 equiv), MeOH (2.0 mL) and morpholine (12 mL). The crude residue was purified by flash column chromatography on silica gel (20% EtOAc in hexanes to 50% EtOAc in hexanes) to provide **1c** as white solid (595.8 mg, 3.4 mmol, 37% yield). M.P.: 67°C– 73°C. ^{1}H NMR (400 MHz, $CDCl_3$) δ 8.65 (dt, J = 7.5, 1.0 Hz, 1H), 7.97 (d, J = 1.0 Hz, 1H), 7.58 (dd, J = 2.0, 1.0 Hz, 1H), 7.04 (dd, J = 7.4, 2.0 Hz, 1H), 1.37 (s, 9H). ^{13}C NMR (101 MHz, $CDCl_3$) δ 148.9, 133.6, 124.8, 124.1, 114.6, 111.6, 34.6, 30.0(3C). IR (neat): 2953, 2834, 1568, 1409, 1385, 1240, 1110, 943, 857, 729, 633, 529 cm^{-1}. HRMS (ESI) m/z: $[M+H]^+$ Calcd for $C_{10}H_{14}N_3$ 176.1188; Found 176.1190.

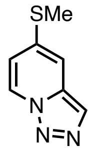

5-(methylthio)-[1,2,3]triazolo[1,5-a]pyridine (1e). Following general procedure B, utilizing 4-(methylthio)picolinaldehyde **S5** (1.70 g, 11.1 mmol, 1.0 equiv), *p*-toluenesulfonylhydrazide (2.25 g, 12.1 mmol, 1.1 equiv), MeOH (4.0 mL) and

morpholine (20 mL). The crude residue was purified by flash column chromatography on silica gel (20% EtOAc in hexanes to 100% EtOAc) to provide **1e** as brownish-yellow solid (306.0 mg, 1.85 mmol, 17% yield). M.P.: 72°C– 75°C. ^{1}H NMR (400 MHz, $CDCl_3$) δ 8.55 (dt, J = 7.4, 1.0 Hz, 1H), 7.88 (d, J = 1.0 Hz, 1H), 7.29 (dd, J = 2.0, 0.9 Hz, 1H), 6.79 (dd, J = 7.4, 1.9 Hz, 1H), 2.54 (s, 3H). ^{13}C NMR (101 MHz, $CDCl_3$) δ 138.6, 134.2, 124.4, 124.1, 115.3, 110.1, 77.8 – 76.8 (m), 15.0. IR (neat): 2953, 2834, 1568, 1409, 1385, 1240, 1110, 943, 857, 729, 633, 529 cm^{-1}. HRMS (ESI) m/z: $[M+H]^+$ Calcd for $C_7H_8N_3S$ 166.0439; Found 166.0437.

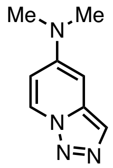

***N,N*-dimethyl-[1,2,3]triazolo[1,5-*a*]pyridin-5-amine (1f)**. Following general procedure B, utilizing 4-(dimethylamino)picolinaldehyde **S6** (500.0 mg, 3.30 mmol, 1.0 equiv), *p*-toluenesulfonylhydrazide (700.0 mg, 3.63 mmol, 1.1 equiv), MeOH (1.0 mL) and morpholine (4.0 mL).The crude residue was purified by flash column chromatography on silica gel (20% EtOAc in hexanes to 100% EtOAc) to provide **1f** as brownish-yellow solid (149.0 mg, 0.92 mmol, 28% yield). M.P.: 113°C– 117°C. ^{1}H NMR (400 MHz, $CDCl_3$) δ 8.51 (dt, J = 7.7, 0.8 Hz, 1H), 7.68 (d, J = 1.0 Hz, 1H), 6.66 (dd, J = 7.8, 2.6 Hz, 1H), 6.49 (d, J = 2.6 Hz, 1H), 3.05 (s, 6H). ^{13}C NMR (101 MHz, $CDCl_3$) δ 147.5, 135.8, 125.0, 122.1, 106.8, 91.5, 40.3(2C). IR (neat): 2953, 2834, 1568, 1409, 1385, 1240, 1110, 943, 857, 729, 633, 529 cm^{-1}. HRMS (ESI) m/z: $[M+H]^+$ Calcd for $C_8H_{11}N_4$ 163.0984; Found 163.0981.

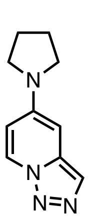

5-(pyrrolidin-1-yl)-[1,2,3]triazolo[1,5-*a*]pyridine (1g). Following general procedure B, utilizing 4-(pyrrolidin-1-yl)picolinaldehyde **S7** (352.0 mg, 2.0 mmol, 1.0 equiv), *p*-toluenesulfonylhydrazide (409.0 mg, 2.2 mmol, 1.1 equiv), MeOH (1.0 mL) and morpholine (4.0 mL). The crude residue was purified by flash column chromatography on silica gel (20% EtOAc in hexanes to 100% EtOAc) to provide **1g** brownish-yellow solid (106.0 mg, 0.56 mmol, 28% yield). M.P.: 128°C– 132°C. ^{1}H NMR (400 MHz,

$CDCl_3$) δ 8.46 (d, *J* = 7.7 Hz, 1H), 7.61 (s, 1H), 6.49 (dd, *J* = 7.7, 2.4 Hz, 1H), 6.30 (s, 1H), 3.51 – 3.00 (m, 4H), 2.57 – 1.93 (m, 4H). ^{13}C NMR (126 MHz, $CDCl_3$) δ 144.9, 136.1, 125.2, 121.7, 107.0, 90.2, 45.0(2C), 25.6(2C). IR (neat): 2953, 2834, 1568, 1409, 1385, 1240, 1110, 943, 857, 729, 633, 529 cm^{-1}. HRMS (ESI) m/z: $[M+H]^+$ Calcd for $C_{10}H_{13}N_4$ 189.1140; Found 189.1138.

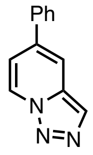

5-phenyl-[1,2,3]triazolo[1,5-*a*]pyridine (1i). Following general procedure B, utilizing 4-phenylpicolinaldehyde **S9** (350.0 mg, 1.9 mmol, 1.0 equiv), *p*-toluenesulfonylhydrazide (390.0 mg, 2.1 mmol, 1.1 equiv), MeOH (1.0 mL) and morpholine (4.0 mL). The crude residue was purified by flash column chromatography on silica gel (10% EtOAc in hexanes to 50% EtOAc in hexanes) to provide **1i** as white solid (220.0 mg, 1.13 mmol, 59% yield). M.P.: 111°C– 114°C. ^1H NMR (500 MHz, CD_3OD) δ 8.98 – 8.89 (m, 1H), 8.17 (d, *J* = 1.0 Hz, 1H), 8.13 (dd, *J* = 1.9, 1.0 Hz, 1H), 7.80 – 7.74 (m, 2H), 7.55 – 7.48 (m, 3H), 7.47 – 7.41 (m, 1H). ^{13}C NMR (126 MHz, CD_3OD) δ 140.3, 138.9, 135.9, 130.3, 130.0(2C), 128.0(2C), 126.9, 126.4, 117.3, 115.5. IR (neat): 2953, 2834, 1568, 1409, 1385, 1240, 1110, 943, 857, 729, 633, 529 cm^{-1}. HRMS (ESI) m/z: $[M+H]^+$ Calcd for $C_{12}H_{10}N_3$196.0875; Found 196.0874.

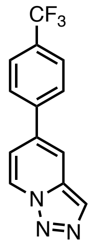

5-(4-(trifluoromethyl)phenyl)-[1,2,3]triazolo[1,5-*a*]pyridine (1j). Following general procedure B, utilizing 4-(4-(trifluoromethyl)phenyl)picolinaldehyde **S11** (1.50 g, 6.0 mmol, 1.0 equiv), *p*-toluenesulfonylhydrazide (1.20 g, 6.60 mmol, 1.10 equiv), MeOH (2.0 mL) and morpholine (10.0 mL). The crude residue was purified by flash column chromatography on silica gel (10% EtOAc in hexanes to 25% EtOAc in hexanes) to provide **1j** as white solid (1.24 g, 4.8 mmol, 80% yield). M.P.: 133°C– 136°C. ^1H NMR (500 MHz, $CDCl_3$) δ 8.83 (dt, *J* = 7.3, 1.0 Hz, 1H), 8.15 (d, *J* = 1.0 Hz, 1H), 7.93 (dd, *J* = 1.9, 1.0 Hz, 1H), 7.79 – 7.74 (m, 4H), 7.25 (dd, *J* = 7.3, 1.9 Hz, 1H). ^{13}C NMR (126 MHz, $CDCl_3$) δ 141.4, 137.2, 134.0, 131.0 (q, *J* = 32.8 Hz), 127.5, 126.6, 126.4 (q, *J* =

3.8 Hz), 125.7, 124.05 (q, J = 272.2 Hz), 115.5, 115.2. ^{19}F NMR (376 MHz, $CDCl_3$) δ -62.78. IR (neat): 2953, 2834, 1568, 1409, 1385, 1240, 1110, 943, 857, 729, 633, 529 cm^{-1}. HRMS (ESI) m/z: $[M+H]^+$ Calcd for $C_{13}H_9F_3N_3$ 264.0749; Found 264.0754.

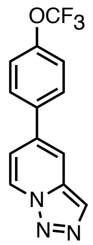

5-(4-(trifluoromethoxy)phenyl)-[1,2,3]triazolo[1,5-*a*]pyridine (1k). Following general procedure B, utilizing 4-(4- (trifluoromethoxy)phenyl)picolinaldehyde **S10** (1.0 g, 4.00 mmol, 1.0 equiv), *p*-toluenesulfonylhydrazide (800.0 mg, 4.40 mmol, 1.1 equiv), MeOH (2.0 mL) and morpholine (8.0 mL). The crude residue was purified by flash column chromatography on silica gel (10% EtOAc in hexanes to 25% EtOAc in hexanes) to provide **1k** as white solid (1.09 g, 3.92 mmol, 98% yield). M.P.: 100°C– 104°C. ^{1}H NMR (500 MHz, $CDCl_3$) δ 8.81 (d, J = 7.3 Hz, 1H), 8.12 (d, J = 1.0 Hz, 1H), 7.87 (dd, J = 1.9, 1.1 Hz, 1H), 7.69 – 7.65 (m, 2H), 7.36 (dt, J = 7.8, 1.0 Hz, 2H), 7.21 (dd, J = 7.3, 1.9 Hz, 1H). ^{13}C NMR (126 MHz, $CDCl_3$) δ 149.8, 137.2, 136.6, 134.1, 128.6, 126.3, 125.5, 121.7, 120.5 (q, J = 257.9 Hz), 115.2, 114.8. ^{19}F NMR (376 MHz, $CDCl_3$) δ -57.89. IR (neat): 2953, 2834, 1568, 1409, 1385, 1240, 1110, 943, 857, 729, 633, 529 cm^{-1}. HRMS (ESI) m/z: $[M+H]^+$ Calcd for $C_{13}H_9F_3N_3O$ 280.0689; Found 280.0701.

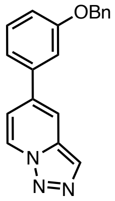

5-(3-(benzyloxy)phenyl)-[1,2,3]triazolo[1,5-*a*]pyridine (1l). Following general procedure B, utilizing 4-(3-(benzyloxy)phenyl)picolinaldehyde **S13** (665.6 mg, 2.3 mmol, 1.0 equiv), *p*-toluenesulfonylhydrazide (465.0 mg, 2.5 mmol, 1.1 equiv), MeOH (0.5 mL) and morpholine (3.0 mL). The crude residue was purified by flash column chromatography on silica gel (17% EtOAc in hexanes to 30% EtOAc in hexanes) to provide **1l** as white solid (392.0 mg, 1.3 mmol, 57% yield). M.P.: 83°C– 86°C. ^{1}H NMR (500 MHz, CD_3OD) δ 8.91 (dt, J = 7.3, 1.0 Hz, 1H), 8.15 (d, J = 1.0 Hz, 1H), 8.10 (dd, J = 1.9, 1.0 Hz, 1H), 7.49 – 7.43 (m, 3H), 7.43 – 7.36 (m, 4H), 7.35 – 7.29 (m, 2H), 7.08 (ddd, J = 8.2, 2.5, 0.9 Hz, 1H), 5.17 (s, 2H). ^{13}C NMR (126 MHz, CD_3OD) δ 160.9, 140.3,

140.1, 138.6, 135.8, 131.4, 129.6(2C), 129.0, 128.7(2C), 127.0, 126.3, 120.6, 117.3, 116.4, 115.7, 114.7, 71.2. IR (neat): 2953, 2834, 1568, 1409, 1385, 1240, 1110, 943, 857, 729, 633, 529 cm^{-1}. HRMS (ESI) m/z: $[M+H]^+$ Calcd for $C_{19}H_{16}N_3O$ 302.1293; Found 302.1298.

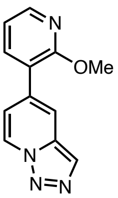

5-(2-methoxypyridin-3-yl)-[1,2,3]triazolo[1,5-*a*]pyridine (1m). Following general procedure B, utilizing 2-methoxy-[3,4'-bipyridine]-2'-carbaldehyde **S14** (1.10 g, 5.00 mmol, 1.0 equiv), *p*-toluenesulfonylhydrazide (1.02 g, 5.50 mmol, 1.1 equiv), MeOH (2.0 mL) and morpholine (10 mL). The crude residue was purified by flash column chromatography on silica gel (10% EtOAc in hexanes to 50% EtOAc in hexanes) to provide **1m** as white solid (565.0 mg, 2.5 mmol, 50% yield). M.P.: 138°C– 142°C. ^{1}H NMR (500 MHz, $CDCl_3$) δ 8.75 (dt, *J* = 7.3, 1.0 Hz, 1H), 8.25 (dd, *J* = 4.9, 1.9 Hz, 1H), 8.10 (d, *J* = 1.0 Hz, 1H), 7.90 (dd, *J* = 1.8, 1.0 Hz, 1H), 7.70 (dd, *J* = 7.4, 1.9 Hz, 1H), 7.25 (dd, *J* = 7.3, 1.8 Hz, 1H), 7.04 (dd, *J* = 7.3, 5.0 Hz, 1H), 4.02 (s, 3H). ^{13}C NMR (126 MHz, $CDCl_3$) δ 160.9, 147.5, 138.5, 134.6, 134.0, 126.2, 124.5, 121.5, 117.5, 117.3, 117.1, 53.9. IR (neat): 2953, 2834, 1568, 1409, 1385, 1240, 1110, 943, 857, 729, 633, 529 cm^{-1}. HRMS (ESI) m/z: $[M+H]^+$ Calcd for $C_{12}H_{11}N_4O$ 227.0933; Found 227.0936.

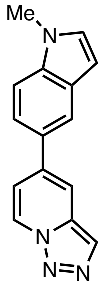

5-(1-methyl-1*H*-indol-5-yl)-[1,2,3]triazolo[1,5-*a*]pyridine (1n). Following general procedure B, utilizing 4-(1-methyl-1*H*-indol-5-yl)picolinaldehyde **S15** (4.27 g, 18.0 mmol, 1.0 equiv), *p*-toluenesulfonylhydrazide (3.72 g, 19.8 mmol, 1.1 equiv), MeOH (10.0 mL) and morpholine (25.0 mL). The crude residue was purified by flash column chromatography on silica gel (10% EtOAc in hexanes to 50% EtOAc in hexanes) to provide **1n** as yellow solid (1.49 g, 6.0 mmol, 33% yield). M.P.: 183°C– 188°C. ^{1}H NMR (400 MHz, $CDCl_3$) δ 8.77 (dt, *J* = 7.3, 1.0 Hz, 1H), 8.06 (d, *J* = 1.0 Hz, 1H), 7.92– 7.88(m,

2H), 7.52 (dd, J = 8.6, 1.8 Hz, 1H), 7.44 (d, J = 8.6 Hz, 1H), 7.35 (dd, J = 7.3, 1.8 Hz, 1H), 7.14 (d, J = 3.1 Hz, 1H), 6.58 (dd, J = 3.1, 0.9 Hz, 1H), 3.86 (s, 3H). ^{13}C NMR (101 MHz, $CDCl_3$) δ 140.2, 137.1, 134.6, 130.4, 129.4, 129.2, 125.7, 125.0, 120.8, 119.7, 116.2, 113.7, 110.1, 101.8, 33.2. IR (neat): 2953, 2834, 1568, 1409, 1385, 1240, 1110, 943, 857, 729, 633, 529 cm^{-1}. HRMS (ESI) m/z: [M+H]$^+$ Calcd for $C_{15}H_{13}N_4$ 249.1140; Found 249.1139.

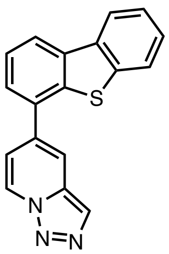

5-(dibenzo[*b*,*d*]thiophen-4-yl)-[1,2,3]triazolo[1,5-*a*]pyridine (1o). Following general procedure B, utilizing (dibenzo[b,d]thiophen-4-yl)picolinaldehyde **S16** (3.00 g, 10.0 mmol, 1.0 equiv), *p*-toluenesulfonylhydrazide (2.05 g, 11.0 mmol, 1.1 equiv), MeOH (5.0 mL) and morpholine (25.0 mL). The crude residue was purified by flash column chromatography on silica gel (10% EtOAc in hexanes to 50% EtOAc in hexanes) to provide **1o** as yellow solid (512.3 mg, 1.7 mmol, 17% yield). M.P.: 173°C– 175°C. ^{1}H NMR (400 MHz, $CDCl_3$) δ 8.89 (dt, J = 7.2, 1.0 Hz, 1H), 8.29 – 8.23 (m, 2H), 8.19 (d, J = 1.0 Hz, 1H), 8.14 (dd, J = 1.8, 1.1 Hz, 1H), 7.95 – 7.83 (m, 1H), 7.64 (t, J = 7.6 Hz, 1H), 7.59 (d, J = 1.2 Hz, 1H), 7.57 – 7.52 (m, 2H), 7.41 (dd, J = 7.2, 1.8 Hz, 1H).^{13}C NMR (101 MHz, $CDCl_3$) δ 139.2, 138.4, 138.1, 137.0, 135.6, 134.0, 133.8, 127.5, 126.9, 126.4, 125.5, 125.5, 125.0, 122.9, 122.1, 122.0, 116.6, 116.4. IR (neat): 2953, 2834, 1568, 1409, 1385, 1240, 1110, 943, 857, 729, 633, 529 cm^{-1}. HRMS (ESI) m/z: [M+H]$^+$ Calcd for $C_{18}H_{12}N_3S$ 302.0752; Found 302.0748.

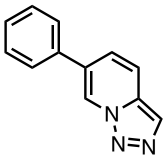

6-phenyl-[1,2,3]triazolo[1,5-*a*]pyridine (**1q**). Following general procedure B, utilizing 5-phenylpicolinaldehyde **S18** (380.0 mg, 2.07 mmol, 1.0 equiv), *p*-toluenesulfonylhydrazide (428.0 mg, 2.30 mmol, 1.1 equiv), MeOH (1.0 mL) and morpholine (4.0 mL). The crude residue was purified by flash column chromatography on silica gel (10% EtOAc in hexanes to 30% EtOAc in hexanes) to provide **1q** as white solid (290.0 mg, 1.49 mmol, 72% yield). M.P.: 97°C– 100°C. ^{1}H NMR (500 MHz, $CDCl_3$)

δ 8.93 (d, J = 1.2 Hz, 1H), 8.09 (d, J = 1.0 Hz, 1H), 7.80 (dd, J = 9.1, 1.1 Hz, 1H), 7.65 – 7.60 (m, 2H), 7.57 – 7.50 (m, 3H), 7.49 – 7.44 (m, 1H). ^{13}C NMR (75 MHz, $CDCl_3$) δ 136.2, 132.9, 129.9, 129.5(2C), 128.9, 127.3(2C), 126.3, 125.6, 122.3, 117.8. IR (neat): 3118, 3101, 3064, 3037, 1637, 1579, 1518, 1468, 1453, 1335, 1200, 1100, 966, 883, 818, 755, 688 cm^{-1}. HRMS (ESI) m/z: $[M+H]^+$ Calcd for $C_{12}H_{10}N_3$ 196.0875; Found 196.0878.

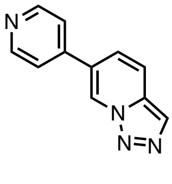

6-(pyridin-4-yl)-[1,2,3]triazolo[1,5-*a*]pyridine (1r). Following general procedure B, utilizing [3,4'-bipyridine]-6-carbaldehyde **S19** (500.0 mg, 2.71 mmol, 1.0 equiv), *p*-toluenesulfonylhydrazide (558.4 mg, 3.00 mmol, 1.1 equiv), MeOH (2.0 mL) and morpholine (6 mL). The crude residue was purified by flash column chromatography on silica gel (30% EtOAc in hexanes to 100% EtOAc) to provide **1r** as white solid (310.0 mg, 1.58 mmol, 58% yield). M.P.: 183°C– 188°C. ^{1}H NMR (500 MHz, $CDCl_3$) δ 9.03 (d, J = 1.5 Hz, 1H), 8.85 – 8.69 (m, 2H), 8.13 (d, J = 1.0 Hz, 1H), 7.87 (dd, J = 9.2, 1.1 Hz, 1H), 7.57 – 7.52 (m, 3H).^{13}C NMR (101 MHz, $CDCl_3$) δ 151.0(2C), 143.6, 133.3, 127.0, 125.9, 125.0, 123.2, 121.5 (2C), 118.6. IR (neat): 3118, 3063, 3032, 1625, 1579, 1480, 1445, 1227, 977, 839, 819,795, 741, 686 cm^{-1}. HRMS (ESI) m/z: $[M+H]^+$ Calcd for $C_{11}H_9N_4$ 197.0827; Found 197.0821.

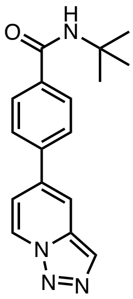

4-([1,2,3]triazolo[1,5-*a*]pyridin-5-yl)-*N*-(*tert*-butyl)benzamide (1t). Following general procedure B, utilizing *N*-(*tert*-butyl)-3'-formyl-[1,1'-biphenyl]-4-carboxamide **S12** (2.20 g, 7.80 mmol, 1.0 equiv), *p*-toluenesulfonylhydrazide (1.60 g, 8.58 mmol, 1.1 equiv), MeOH (5.0 ml) and morpholine (10 ml). The crude residue was purified by flash column chromatography on silica gel (10% EtOAc in hexanes to 50% EtOAc in hexanes) and further purified by another column chromatography on silica gel (10% EtOAc in hexanes to 50% EtOAc in hexanes) to provide **1t** as white solid (300.0 mg, 1.02 mmol, 13% yield).

M.P.: 208°C– 210°C. ^{1}H NMR (500 MHz, CD_3OD) δ 8.98 (dd, J = 7.2, 1.4 Hz, 1H), 8.29 – 8.17 (m, 2H), 7.93 – 7.82 (m, 4H), 7.54 (dd, J = 7.4, 2.1 Hz, 1H), 1.49 (s, 7H). ^{13}C NMR (101 MHz, $CDCl_3$) δ 166.2, 140.5, 137.6, 136.3, 134.1, 127.9 (2C), 127.2 (2C), 126.5, 125.5, 115.3, 115.0, 52.0, 29.0. IR (neat): 2953, 2834, 1568, 1409, 1385, 1240, 1110, 943, 857, 729, 633, 529 cm^{-1}. HRMS (ESI) m/z: $[M+H]^+$ Calcd for $C_{17}H_{18}N_4NaO$ 317.1378; Found 317.1372.

Synthesis of 4-phenylpyrazolo[1,5-*a*]pyridine 12c

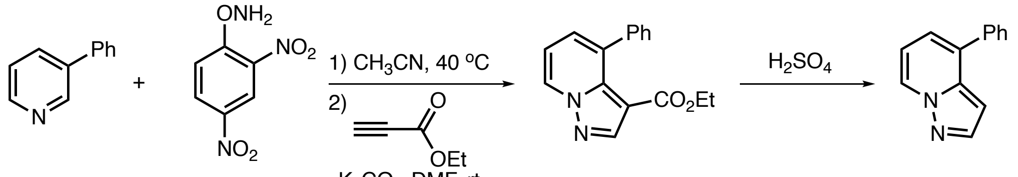

S20 **S21** **S22** **12c**

4-phenylpyrazolo[1,5-*a*]pyridine (12c).[19] An oven-dried Schlenk tube equipped with a stirring magnetic bar was charged 3-phenylpyridine **S20** (1.94 g, 12.5 mmol, 1.0 equiv), *o*-(2,4-dinitrophenyl)hydroxylamine **S21**(2.69 g, 13.5 mmol, 1.0 equiv) and CH_3CN (20.0 mL). The reaction mixture was stirred at 40 °C in an oil bath for 20 h. Afterwards, the mixture was concentrated in vacuo, and K_2CO_3 (2.42g, 17.5 mmol, 1.4 equiv), ethyl propiolate (1.80 mL, 17.5 mmol, 1.4 equiv) and DMF (25 mL) were added and stirred at rt for 20 h. The reaction was quenched with water (250 mL) and extracted with EtOAc (3x80 mL). The combination of organic layers was washed with brine (2x80 mL), dried over anhydrous Na_2SO_4 and concentrated in vacuo. The resulting residue was purified by flash column chromatography on silica gel (15% EtOAc in hexanes) to provide **S22**[20] (630 mg, 19% yield). An oven-dried Schlenk tube equipped with an stirring magnetic bar was charged ethyl 4-phenylpyrazolo[1,5-*a*]pyridine-3-carboxylate **S22** (630 mg, 2.5 mmol) and H_2SO_4 (2 mL, 50% v/v). The reaction mixture was stirred at 110 °C in an oil bath for 2h. The mixture was quenched by addition of water (10 mL) followed by extraction with EtOAc (3x10 mL). The combined organic layers were washed with brine (2x10 mL) and a saturated aqueous solution of Na_2CO_3 (2x 10 mL), dried over anhydrous Na_2SO_4, and the solvent was removed under reduced pressure. The resulting residue was purified by flash column chromatography on silica gel (15% EtOAc in hexanes) to provide **12c** as a white solid (370.0 mg, 76% yield). M.P.: 79°C– 82°C. ^{1}H NMR (300 MHz, CDC13) δ 8.48 (dt, J = 7.0, 1.0 Hz, 1H), 7.98 (d, J = 2.4 Hz, 1H), 7.74 – 7.62 (m, 2H), 7.55 – 7.37 (m, 3H), 7.13 (dd, J = 7.0, 1.0 Hz, 1H), 6.84 (t, J = 7.0 Hz, 1H), 6.67 (dd, J = 2.4, 1.0 Hz, 1H). ^{13}C NMR (75 MHz, $CDCl_3$) δ 142.1, 139.9, 138.2, 132.5, 128.9

(2C), 128.5, 128.1 (2C), 127.7, 122.3, 112.0, 97.2. IR (neat): 3020, 1622, 1412, 1170, 775, 756, 699 cm^{-1}. HRMS (ESI) m/z: $[M+H]^+$ Calcd for $C_{13}H_{11}N_2$ 195,0922; Found 195.0914.

Optimization of the C3-Deuteration of [1,2,3]triazolo[1,5-*a*]pyridine 1a

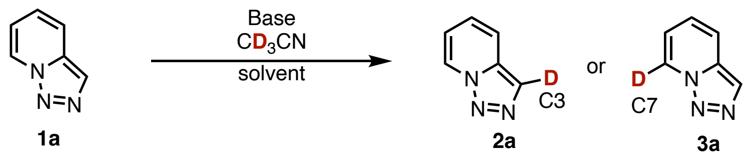

General Procedure C: An oven-dried 8 mL vial equipped with a stirring magnetic bar was charged with **1a** (0.2 mmol, 1.0 equiv) and put into a nitrogen-filled glovebox. The corresponding base was added and the vial was taken-out from the glovebox. Then, the corresponding solvent (1.0 mL) was added, followed by CD_3CN and the reaction was stirred for 17 hours at room temperature. Afterwards, the reaction mixture was diluted with EtOAc, filtered through silica plug and concentrated in vacuo.

Table S1. Effect of the solvent

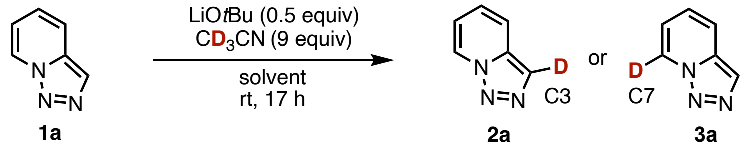

Entry	Base	**C3-D** (%D)[a]	**C7-D** (%D)[a]
1	1,4-Dioxane	95	16
2	THF	93	17
3	DME	94	29
4	Et_2O	94	45
5	MeOH	4	2
6	$CHCl_3$	3	5
7	DCE	9	3
8	Acetone	3	2

[a] Deuterium incorporation at C3 & C7 is determined using ^{1}H-NMR, based on comparison with non-deuterated C4–H of **2a** and **3a** position.

Table S2. Effect of the Base

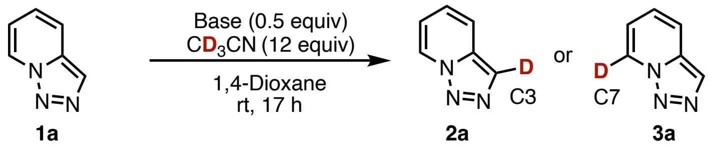

Entry	Base	**C3-D** (%D)[a]	**C7-D** (%D)[a]
1	LiO*t*-Bu	95	16
**2**[b]	**LiO*t*-Bu**	**95**	**5**
3	NaO*t*-Bu	93	93
4	KO*t*-Bu	92	93
5	LiHMDS	0	0
6	NaHMDS	41	52
7	KHMDS	93	93
8	2,6-Lutidine	0	0
9	DMAP	0	0
10	DABCO	0	0
11	LiOMe	12	5
12	LiOAc	0	0
13	Li_2CO_3	0	0
14	Li_3PO_4	0	0
15	$LiClO_4$	0	0
16	$LiNH_2$	0	0

[a] Deuterium incorporation at C3 & C7 is determined using ^{1}H-NMR, based on comparison with non-deuterated C4–H of **2a** and **3a** position.

Table S3. Effect of the Deuterium Source

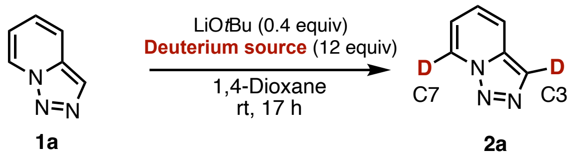

Entry	Deuterated reagent	***d*-C3** (%D)[a]	***d*-C7** (%D)[b]
1	CD_3CN	95	5

2	DMSO-*d6*	93	43
3	Acetone-*d6*	0	0
4	D_2O	0	0

[a] Deuterium incorporation at C3 & C7 is determined using ^{1}H-NMR, based on comparison with non-deuterated C4–H of **2a** and **3a** position.

Optimization of the C3-Deuteration of pyrazolo[1,5-a]pyridine 12a

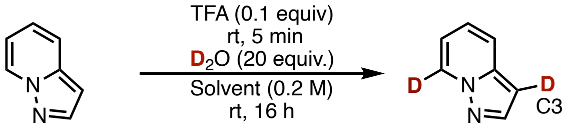

12a **13a**

General Procedure D: An oven-dried 8 mL vial equipped with a stirring magnetic bar was charged with pyrazolo[1,5-*a*]pyridine **12a** (0.2 mmol, 1.0 equiv). The vial was evacuated and backfilled with argon for at least three times and solvent (1.0 mL, 0.2 M) was added. TFA (1.5 µL, 0.02 mmol, 0.1 equiv) and D_2O (72 µL, 4.0 mmol, 20 equiv) were added at room temperature and the reaction was stirred for 16 hours at rt. The reaction mixture was quenched by Et_3N (10.0 µL), diluted with EtOAc, filtered through a silica plug and the concentrated in vacuo. The reaction was examined by ^{1}H-NMR.

Table S4. Effect of the solvent

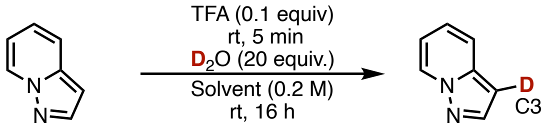

12a **13a**

Entry	Solvent	**C3-D** (%D)[a]	**C7-D (%D)**[a]
1	$CHCl_3$	88	0
2	DCM	90	0
3	Toluene	93	0
4	MeOH	25	0
5	THF	77	0
6	Et_2O	94	0
7	DCE	92	0
**8**	**CH_3CN**	**96**	**0**

[a] Deuterium incorporation at C3 & C7 is determined using ^{1}H-NMR, based on comparison with non-deuterated C4–H of **2a** position.

Optimization of the C7-Deuteration of pyrazolo[1,5-a]pyridine 12a

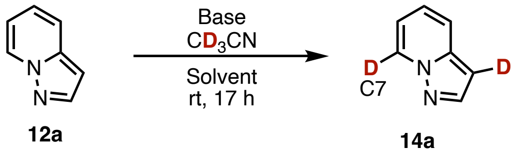

General Procedure E: An oven-dried 8 mL vial equipped with a stirring magnetic bar was charged with pyrazolo[1,5-a]pyridine **12a** (1.0 equiv). The vial was put into a nitrogen filled glovebox and base (0.1 mmol, 0.5 equiv) was added. The vial was taken out from the glovebox and, 1,4-dioxane (1 mL, 0.2 M) followed by CD_3CN (125 µL, 2.4 mmol, 12 equiv) were added at room temperature and the reaction was stirred for 17 hours. The reaction mixture was diluted with EtOAc, filtered through a silica plug and concentrated in vacuo. The crude was purified by flash column chromatography on silica gel to provide the desired product.

Table S5. Effect of the base

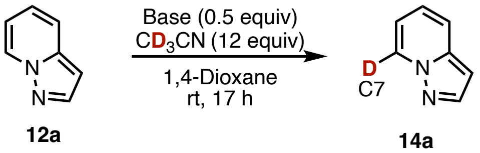

Entry	Base	**C7-D** (%D)[a]	**C3-D** (%D)[a]
1	LiO*t*-Bu	0	0
2	NaO*t*-Bu	68	0
**3**	**KO*t*-Bu**	**96**	**0**
4	LiHMDS	11	0
5	NaHMDS	82	0
6	KHMDS	80	0
7	NaOAc	0	0
8	KOAc	0	0
9	K_2CO_3	0	0
10	KOMe	31	0
11	NaOMe	0	0
12	K_3PO_4	0	0
13	NEt_3	0	0

14	2,6-Lutidine	7	0

[a] Deuterium incorporation at C3 & C7 is determined using ^{1}H-NMR, based on comparison with non-deuterated C4–H of **2a** position.

Table S6. Effect of the solvent

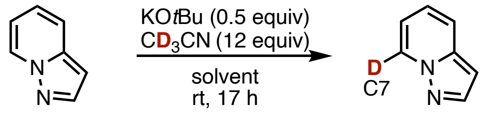

12a **14a**

Entry	Solvent	**C7-D** (%D)[a]	**C3-D** (%D)[a]
1	THF	93	0
**2**	**Dioxane**	**96**	**0**
3	DME	90	0
4	Et_2O	84	0
5	$CHCl_3$	2	0
6	DCM	11	0
7	DCE	0	0
8	Acetone	4	0
9	MeOH	0	0
10	Toluene	85	0

[a] Deuterium incorporation at C3 & C7 is determined using ^{1}H-NMR, based on comparison with non-deuterated C4–H of **2a** position.

Selective C3-Deuteration of [1,2,3]triazolo[1,5-a]pyridine

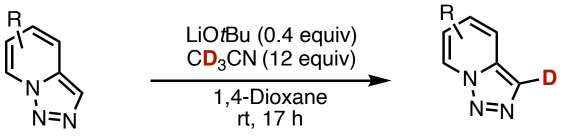

1 **2**

General procedure 1 (GP1). An oven-dried 8 mL vial equipped with a stirring magnetic bar was charged with **1** (0.2 mmol, 1.0 equiv) and put into a nitrogen-filled glovebox. LiO*t*-Bu (6.4 mg, 0.08 mmol, 0.4 equiv) was added and the vial was taken-out from the glovebox. 1,4-dioxane (1.0 mL) was added, followed by CD_3CN (125 µL, 2.40 mmol, 12 equiv) and the reaction was stirred for 17 hours at room temperature. Afterwards, the reaction mixture was diluted with EtOAc, filtered through silica plug and concentrated in

vacuo. The residue was purified by flash column chromatography on silica gel to provide the desired product.

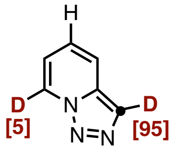

[1,2,3]triazolo[1,5-a]pyridine-3,7-*d*$_2$(2a). Following **GP1,** utilizing [1,2,3]triazolo[1,5-*a*]pyridine **1a** (23.8 mg, 0.2 mmol, 1.0 equiv), LiO*t*-Bu (6.4 mg, 0.08 mmol, 0.4 equiv), CD_3CN (125 µL, 2.40 mmol, 12 equiv) and 1,4-dioxane (1.0 mL). Flash column chromatography on silica gel (50% EtOAc in hexanes) afforded **2a** as amorphous white solid (23.5 mg, 0.196 mmol, 98% yield, 95% D, C3:C7 = 19:1). In an independent experiment, 23.6 mg (98% yield, 95% D, C3:C7 = 19:1) were obtained, giving an average yield of 98%. Deuterium incorporation was determined by ^{1}H NMR, based on comparison with non-deuterated C4–H position. ^{1}H NMR (500 MHz, $CDCl_3$) δ 8.53 (q, *J* = 1.2 Hz, 0.95H), 8.00 (d, *J* = 1.0 Hz, 0.05H), 7.62 (dd, *J* = 9.0, 1.0 Hz, 1H), 7.10 (dd, *J* = 9.0, 1.4 Hz, 1H), 2.40 (d, *J* = 1.2 Hz, 3H).^{13}C NMR (126 MHz, $CDCl_3$) δ 133.6, 125.6, 125.2, 117.9, 115.2, 115.1. IR (neat): 3072, 2919, 2848, 1476, 1241, 1158, 1028, 637 cm^{-1}. HRMS (ESI) m/z: $[M+H]^+$ Calcd for $C_6H_5DN_3$ 121.0624; Found 121.0617.

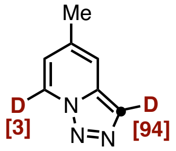

5-methyl-[1,2,3]triazolo[1,5-*a*]pyridine-3,7-*d*$_2$ (2b). Following **GP1**, utilizing 5-methyl-[1,2,3]triazolo[1,5-*a*]pyridine **1b** (26.6 mg, 0.2 mmol, 1.0 equiv) and LiO*t*-Bu (16.0 mg, 0.2 mmol, 1.0 equiv), CD_3CN (125 µL, 2.40 mmol, 12 equiv) and 1,4-dioxane (1.0 mL). Flash column chromatography on silica gel (50% EtOAc in hexanes) afforded the **2b** as white solid (26.3 mg, 0.196 mmol, 98% yield, 94% D, C3:C7 = 31:1). In an independent experiment, 26.3 mg (98% yield 94% D, C3:C7 = 31:1) were obtained, giving an average yield of 98%. Deuterium incorporation was determined by ^{1}H NMR, based on comparison with non-deuterated C4–H position. M.P.: 63°C– 65°C. ^{1}H NMR (500 MHz, $CDCl_3$) δ 8.59 (dd, *J* = 7.2, 1.0 Hz, 1H), 7.89 (d, *J* = 1.0 Hz, 0H), 7.44 –7.41 (m, 1H), 6.77 (dd, *J* = 7.1, 1.8 Hz, 1H), 2.41 (d, *J* = 1.2 Hz, 3H). ^{13}C NMR (126 MHz, $CDCl_3$) δ 136.3, 134.0, 124.5, 124.3(t, *J* = 30.1 Hz), 118.0, 115.9, 21.3. IR (neat): 3118, 3064, 2917, 2855, 1641, 1462, 1362, 1198, 801, 618 cm^{-1}. HRMS (ESI) m/z: $[M+H]^+$ Calcd for $C_7H_7DN_3$ 135.0781; Found 135.0780.

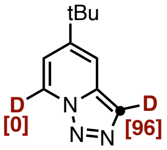

5-(*tert*-butyl)-[1,2,3]triazolo[1,5-*a*]pyridine-3-*d* (2c). Following **GP1**, utilizing 5-(*tert*-butyl)-[1,2,3]triazolo[1,5-*a*]pyridine **1c** (35.0 mg, 0.2 mmol, 1.0 equiv), LiO*t*-Bu (6.4 mg, 0.08 mmol, 0.4 equiv), CD_3CN (125 µL, 2.40 mmol, 12 equiv) and 1,4-dioxane (1.0 mL).. Flash column chromatography on silica gel (10% EtOAc in hexanes to 30% EtOAc in hexanes) afforded the **2c** as white solid (34.5 mg, 0.196 mmol, 98% yield, 96% D, C3:C7 = 99:1). In an independent experiment, 34.2 mg (98% yield, 96% D, C3:C7 = 99:1) were obtained, giving an average yield of 98%. Deuterium incorporation was determined by ^{1}H NMR, based on comparison with non-deuterated C4–H position. M.P.: 56 °C– 63 °C. ^{1}H NMR (500 MHz, $CDCl_3$) δ 8.64 (dd, J = 7.4, 1.0 Hz, 1.0H), 7.95 (d, J = 1.0 Hz, 0.04H), 7.56 (dd, J = 2.0, 1.0 Hz, 1H), 7.02 (dd, J = 7.4, 2.0 Hz, 1H), 1.35 (s, 9H). ^{13}C NMR (126 MHz, $CDCl_3$) δ 149.3, 133.9, 125.0 (t, J = 30 Hz), 124.6, 115.0, 112.0, 35.1, 30.4 (9C). IR (neat): 3120, 2961, 1638, 1524, 1474, 1367, 1163, 887, 798, 657 cm^{-1}. HRMS (ESI) m/z: $[M+H]^+$ Calcd for $C_{10}H_{13}DN_3$ 177.1250; Found 177.1252.

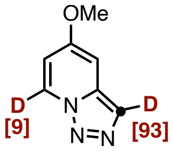

5-methoxy-[1,2,3]triazolo[1,5-a]pyridine-3,7-d2 (2d). Following GP1, utilizing 5-methoxy-[1,2,3]triazolo[1,5-a]pyridine **1d** (29.8 mg, 0.20 mmol, 1.0 equiv), LiO*t*-Bu (6.4 mg, 0.08 mmol, 0.4 equiv), CD_3CN (125 µL, 2.40 mmol, 12 equiv) and 1,4-dioxane (1.0 mL).. Flash column chromatography on silica gel (50% EtOAc in hexanes) afforded 2d as white solid (29.4 mg, 0.196 mmol, 98% yield, 93% D, C3:C7 = 10:1). In an independent experiment, 29.3 mg (98% yield, 93% D, C3:C7 = 10:1) were obtained, giving an average yield of 98%. Deuterium incorporation was determined by ^{1}H NMR, based on comparison with non-deuterated C4–H position. M.P.: 96°C– 100°C. ^{1}H NMR (500 MHz, $CDCl_3$) δ 8.54 (dd, J = 7.6, 0.8 Hz, 0.91 H), 7.84 (d, J = 0.9 Hz, 0.07H), 6.85 (d, J = 2.5 Hz, 1H), 6.65 (dd, J = 7.5, 2.5 Hz, 1H), 3.87 (s, 3H). 13C NMR (126 MHz, $CDCl_3$) δ 157.6, 135.0, 126.0, 124.1(t, J = 28.5 Hz), 110.8, 93.6, 55.9. IR (neat): 3117, 3079, 2921, 2308, 1633, 1542, 1459, 1308, 1202, 1153, 969, 811, 734 cm^{-1}. HRMS (ESI) m/z: $[M+H]^+$ Calcd for $C_7H_7DN_3O$ 151.0730; Found 151.0731.

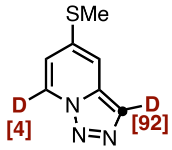

5-(methylthio)-[1,2,3]triazolo[1,5-*a*]pyridine-3,7-*d*$_2$ (2e). Following **GP1**, utilizing 5-(methylthio)-[1,2,3]triazolo[1,5-*a*]pyridine **1e** (33.0 mg, 0.20 mmol, 1.0 equiv), LiO*t*-Bu (6.4 mg, 0.08 mmol, 0.4 equiv), CD_3CN (125 µL, 2.40 mmol, 12 equiv) and 1,4-dioxane (1.0 mL) for 8 h. Flash column chromatography on silica gel (50% EtOAc in hexanes to 100% EtOAc) afforded the title compound **2e** as white solid. (32.9 mg, 0.198 mmol, 99% yield, 92% D, C3:C7 = 23:1). In an independent experiment, 32.5 mg (99% yield, 92% D, C3:C7 = 23:1) were obtained, giving an average yield of 99%. Deuterium incorporation was determined by ^{1}H NMR, based on comparison with non-deuterated C4–H position. M.P.: 74 °C– 78 °C. ^{1}H NMR (500 MHz, $CDCl_3$) δ 8.56 (dd, J = 7.4, 0.9 Hz, 0.96 H), 7.89 (d, J = 0.9 Hz, 0.08H), 7.29 (s, 1H), 6.79 (dd, J = 7.4, 1.9 Hz, 1H), 2.55 (s, 3H). ^{13}C NMR (126 MHz, $CDCl_3$) δ 138.6, 134.1, 124.4, 123.9 (t, J = 28.9), 115.3, 110.1, 15.0 (3C). IR (neat): 3064, 2917, 2323, 2163, 1624, 1520, 1429, 1165, 1062, 931, 792 cm^{-1}. HRMS (ESI) m/z: $[M+H]^+$ Calcd for $C_7H_7DN_3S$ 167.0502; Found 167.0498.

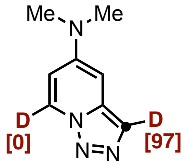

***N,N*-dimethyl-[1,2,3]triazolo[1,5-*a*]pyridin-5-amine-3-*d* (2f)**. Following **GP1**, utilizing *N,N*-dimethyl-[1,2,3]triazolo[1,5-*a*]pyridin-5-amine **1f** (32.4 mg, 0.20 mmol, 1.0 equiv), LiO*t*-Bu (6.4 mg, 0.08 mmol, 0.4 equiv), CD_3CN (125 µL, 2.40 mmol, 12 equiv) and 1,4-dioxane (1.0 mL). Flash column chromatography on silica gel (50% EtOAc in hexanes 50% EtOAc in hexanes to 100% EtOAc) afforded the title compound **2f** as green solid (32.0 mg, 0.196 mmol, 98% yield, 97% D, C3:C7 = 99:1). In an independent experiment, 32.0 mg (98% yield, 97% D, C3:C7 = 99:1) were obtained, giving an average yield of 98%. Deuterium incorporation was determined by ^{1}H NMR, based on comparison with non-deuterated C4–H position. M.P.: 120°C– 122°C. ^{1}H NMR (500 MHz, $CDCl_3$) δ 8.48 (dd, J = 7.8, 0.8 Hz, 1H), 7.66 (d, J = 0.9 Hz, 0.03H), 6.63 (dd, J = 7.8, 2.6 Hz, 1H), 6.46 (d, J = 2.6 Hz, 1H), 3.03 (s, 6H). ^{13}C NMR (126 MHz, $CDCl_3$) δ 147.6, 135.8, 125.1, 122.0 (t, J = 28.9 Hz), 106.9, 91.7, 40.4 (2C). IR (neat): 3055, 2923, 2807, 1645, 1522, 1487, 1437, 1183, 786 cm^{-1}. HRMS (ESI) m/: $[M+H]^+$ Calcd for $C_8H_{10}DN_4$ 164.1046; Found 164.1047.

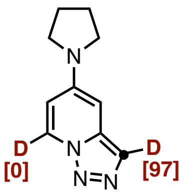

5-(pyrrolidin-1-yl)-[1,2,3]triazolo[1,5-*a*]pyridine-3-*d* (2g). Following **GP1**, utilizing 5-(pyrrolidin-1-yl)-[1,2,3]triazolo[1,5-*a*]pyridine **1g** (37.5 mg, 0.20 mmol, 1.0 equiv), LiO*t*-Bu (6.4 mg, 0.08 mmol, 0.4 equiv), CD_3CN (125 µL, 2.40 mmol, 12 equiv) and 1,4-dioxane (1.0 mL). Flash column chromatography on silica gel (50% EtOAc in hexanes to 100% EtOAc) afforded **2g** as green solid (37.5 mg, 0.198 mmol, 99% yield, 97% D, C3:C7 = 99:1). In an independent experiment, 37.6 mg (99% yield, 97% D, C3:C7 = 99:1) were obtained, giving an average yield of 99%. Deuterium incorporation was determined by ^{1}H NMR, based on comparison with non-deuterated C4–H position. M.P.: 137°C–144°C. ^{1}H NMR (500 MHz, $CDCl_3$) δ 8.45 (dd, *J* = 7.6, 0.8 Hz, 1H), 7.61 (d, *J* = 0.9 Hz, 0.03H), 6.49 (dd, *J* = 7.7, 2.5 Hz, 1H), 6.29 (d, *J* = 2.4 Hz, 1H), 3.37 – 3.29 (m, 4H), 2.57 – 1.19 (m, 4H). ^{13}C NMR (126 MHz, $CDCl_3$) δ 144.9, 136.0, 125.2, 121.5(t, *J* = 29.2 Hz), 107.0, 90.2, 47.9(2C), 25.5(2C). IR (neat): 3059, 2965, 2859, 1643, 1517, 1485, 1378, 1158, 793 cm^{-1}. HRMS (ESI) m/z: $[M+H]^+$ Calcd for $C_{10}H_{12}DN_4$ 190.1203; Found 190.1203.

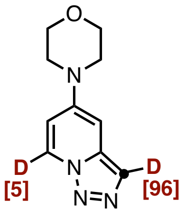

4-([1,2,3]triazolo[1,5-*a*]pyridin-5-yl-3,7-*d*$_2$)morpholine (2h). Following **GP1**, utilizing 4-([1,2,3]triazolo[1,5-*a*]pyridin-5-yl)morpholine **1h** (40.8 mg, 0.20 mmol, 1.0 equiv), LiO*t*-Bu (6.4 mg, 0.08 mmol, 0.4 equiv), CD_3CN (125 µL, 2.40 mmol, 12 equiv) and 1,4-dioxane (1.0 mL). Flash column chromatography on silica gel (30% EtOAc in hexanes to 80% EtOAc in hexanes) afforded the title compound **2h** as yellow solid (40.2 mg, 0.196 mmol, 98% yield, 96% D, C3:C7 = 19:1). In an independent experiment, 40.2 mg (98% yield, 96% D, C3:C7 = 19:1) were obtained, giving an average yield of 98%. Deuterium incorporation was determined by ^{1}H NMR, based on comparison with non-deuterated C4–H position. M.P.: 135 °C– 140 °C. ^{1}H NMR (500 MHz, $CDCl_3$) δ 8.54 – 8.50 (m, 0.95H), 7.75 (d, *J* = 0.9 Hz, 0.04H), 6.73 (m, 2H), 3.88 – 3.84 (m, 4H), 3.22 – 3.20 (m, 4H). ^{13}C NMR (126 MHz, $CDCl_3$) δ 148.5, 135.1, 125.4, 123.4 (t, *J* = 28.7 Hz), 109.0, 96.2, 66.5(2C), 48.6(2C). IR (neat): 3064, 2953, 2850, 1639, 1526, 1446, 1264,

1118, 1045, 970, 901, 826, 796 cm^{-1}. HRMS (ESI) m/z: $[M+H]^+$ Calcd for $C_{10}H_{12}DN_4O$ 206.1152; Found 206.1151.

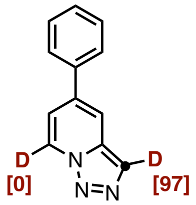

5-phenyl-[1,2,3]triazolo[1,5-*a*]pyridine-3-*d* (2i). Following **GP1**, utilizing 5-phenyl-[1,2,3]triazolo[1,5-*a*]pyridine **1i** (39.0 mg, 0.20 mmol, 1.0 equiv), LiO*t*-Bu (6.4 mg, 0.08 mmol, 0.4 equiv), CD_3CN (125 µL, 2.40 mmol, 12 equiv) and 1,4-dioxane (1.0 mL). Flash column chromatography on silica gel (10% EtOAc in hexanes to 30% EtOAc in hexanes) afforded the title compound **2i** as white solid (38.0 mg, 0.194 mmol, 97% yield, 97% D, C3:C7 = 99:1). In an independent experiment, 38.2 mg (97% yield, 97% D, C3:C7 = 99:1) were obtained, giving an average yield of 97%. Deuterium incorporation was determined by ^{1}H NMR, based on comparison with non-deuterated C4–H position. M.P.: 110 °C– 114 °C. ^{1}H NMR (500 MHz, $CDCl_3$) δ 8.77 (dd, J = 7.3, 1.0 Hz, 1.0H), 8.09 (d, J = 1.0 Hz, 0.03H), 7.87 (dd, J = 1.9, 1.0 Hz, 1H), 7.65 – 7.62 (m, 2H), 7.52 – 7.48 (m, 2H), 7.46 – 7.42 (m, 1H), 7.24 (dd, J = 7.3, 1.8 Hz, 1H). ^{13}C NMR (126 MHz, $CDCl_3$) δ 138.7, 137.9, 134.1, 129.4(2C), 129.0, 127.0(2C), 125.9(t, J = 28.9 Hz), 125.2, 115.5, 114.5. IR (neat): 3052, 2956, 2924, 2349, 1634, 1525, 1458, 1169, 1076, 761, 686 cm^{-1}. HRMS (ESI) m/z: $[M+H]^+$ Calcd for $C_{12}H_9DN_3$ 197.0937; Found 197.0936.

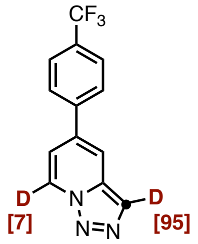

5-(4-(trifluoromethyl)phenyl)-[1,2,3]triazolo[1,5-*a*]pyridine-3,7-*d*$_2$ (2j). Following **GP1**, utilizing 5-(4-(trifluoromethyl)phenyl)-[1,2,3]triazolo[1,5-*a*]pyridine **1j** (52.6 mg, 0.20 mmol, 1.0 equiv), LiO*t*-Bu (9.60 mg, 0.12 mmol, 0.6 equiv) CD_3CN (125 µL, 2.40 mmol, 12 equiv) and 1,4-dioxane (1.0 mL). Flash column chromatography on silica gel (30% EtOAc in hexanes) afforded **2j** as white solid (52.8 mg, 0.198 mmol, 99% yield, 95% D, C3:C7 = 13.5:1). In an independent experiment, 52.0 mg (99% yield, 95% D, C3:C7 = 13.5:1) were obtained, giving an average yield of 99%. Deuterium incorporation was determined by ^{1}H NMR, based on comparison with non-deuterated

C4–H position. M.P.: 133°C– 137°C. ^{1}H NMR (500 MHz, $CDCl_3$) δ 8.81 (dd, J = 7.3, 1.0 Hz, 0.93H), 8.14 (d, J = 0.9 Hz, 0.05H), 7.96 – 7.88 (m, 1H), 7.76 (s, 4H), 7.24 (dd, J = 7.3, 1.9 Hz, 1H); ^{13}C NMR (126 MHz, $CDCl_3$) δ 141.4, 137.1, 134.0, 131.0 (q, J = 32.8 Hz), 127.4 (2C), 126.6, 126.3 (q, J = 3.7 Hz), 125.6, 125.1, 123.0, 115.4, 115.1. ^{19}F NMR (471 MHz, $CDCl_3$) δ -62.7. IR (neat): 3116, 3052, 2924, 2853, 1615, 1521, 1320, 1111, 1069, 838, 798, 731 cm^{-1}. HRMS (ESI) m/z: [M+H]$^+$ Calcd for $C_{13}H_8DF_3N_3$ 265.0811; Found 265.0812.

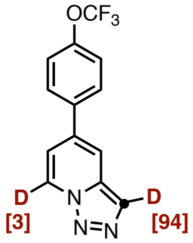

5-(4-(trifluoromethoxy)phenyl)-[1,2,3]triazolo[1,5-a]pyridine-3,7-*d*$_2$ (2k). Following **GP1**, utilizing 5-(4-(trifluoromethoxy)phenyl)-[1,2,3]triazolo[1,5-*a*]pyridine **1k** (55.8 mg, 0.2 mmol, 1.0 equiv), LiO*t*-Bu (9.6 mg, 0.12 mmol, 0.6 equiv), CD_3CN (125 μL, 2.40 mmol, 12 equiv) and 1,4-dioxane (1.0 mL). for 4 h. Flash column chromatography on silica gel (30% EtOAc in hexanes) afforded **2k** as white solid (54.3 mg, 0.194 mmol, 97% yield, 94% D, C3:C7 = 31:1). In an independent experiment, 54.4 mg (97% yield, 94% D, C3:C7 = 31:1) were obtained, giving an average yield of 97%. Deuterium incorporation was determined by ^{1}H NMR, based on comparison with non-deuterated C4–H position. M.P.:97 °C– 101 °C. ^{1}H NMR (400 MHz, $CDCl_3$) δ 8.78 (dd, J = 7.3, 1.0 Hz, 0.97H), 8.10 (d, J = 1.0 Hz, 0.06H), 7.85 (dd, J = 1.9, 1.0 Hz, 1H), 7.70 – 7.63 (m, 2H), 7.37 – 7.31(m, 2H), 7.20 (dd, J = 7.3, 1.9 Hz, 1H). ^{13}C NMR (126 MHz, $CDCl_3$) δ 149.8, 137.2, 136.5, 134.0, 128.5 (2C), 126.1(t, J = 29.0 Hz), 125.4, 121.7(2C), 120.5 (q, J = 257.8 Hz), 115.2, 114.8. ^{19}F NMR (471 MHz, $CDCl_3$) δ -57.8. IR (neat): 3072, 2922, 1642, 1516, 1459, 1251, 1198, 1157, 855, 801 cm^{-1}. HRMS (ESI) m/z: [M+H]$^+$ Calcd for $C_{13}H_8DF_3N_3O$ 281.0760; Found 281.0763.

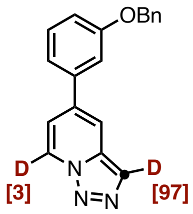

5-(3-(benzyloxy)phenyl)-[1,2,3]triazolo[1,5-*a*]pyridine-3,7-*d*$_2$ (2l). Following **GP1**, utilizing -(3-(benzyloxy)phenyl)-[1,2,3]triazolo[1,5-*a*]pyridine **1l** (60.3 mg, 0.20 mmol, 1.0 equiv), LiO*t*-Bu (9.60 mg, 0.12 mmol, 0.6 equiv), CD_3CN (125 μL, 2.40 mmol, 12

equiv) and 1,4-dioxane (1.0 mL). Flash column chromatography on silica gel (10% EtOAc in hexanes to 30% EtOAc in hexanes) afforded **2l** as white solid (59.7 mg, 0.198 mmol, 99% yield, 97% D, C3:C7 = 32:1). In an independent experiment, 59.7 mg (99% yield, 97% D, C3:C7 = 32:1) were obtained, giving an average yield of 99%. Deuterium incorporation was determined by ^{1}H NMR, based on comparison with non-deuterated C4–H position. M.P.:82 °C– 86 °C. ^{1}H NMR (500 MHz, $CDCl_3$) δ 8.77 (dd, *J* = 7.3, 1.0 Hz, 0.97H), 8.10 (d, *J* = 1.0 Hz, 0.03H), 7.86 (dd, *J* = 1.9, 1.0 Hz, 1H), 7.47 (ddt, *J* = 7.5, 1.4, 0.7 Hz, 2H), 7.44 – 7.40 (m, 3H), 7.38 – 7.33 (m, 1H), 7.26 – 7.21 (m, 3H), 7.06 (ddd, *J* = 8.2, 2.4, 1.0 Hz, 1H), 5.15 (s, 2H). ^{13}C NMR (126 MHz, $CDCl_3$) δ 159.6, 139.4, 138.5, 136.7, 134.1, 130.5, 128.8 (2C), 128.3, 127.6 (2C), 126.1 (t, *J* = 29.4 Hz), 125.3, 119.7, 115.6, 115.1, 114.6, 114.1, 70.4. IR (neat): 3034, 2925, 2872, 1637, 1583, 1525, 1454, 1295, 1192, 1014, 871, 845, 753 cm^{-1}. HRMS (ESI) m/z: $[M+H]^+$ Calcd for $C_{19}H_{15}DN_3O$ 303.1356; Found 303.1374.

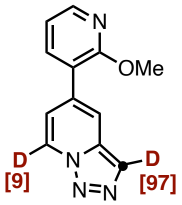

5-(2-methoxypyridin-3-yl)-[1,2,3]triazolo[1,5-*a*]pyridine-3,7-*d*$_2$ (2m). Following **GP1**, utilizing 5-(naphthalen-1-yl)-[1,2,3]triazolo[1,5-*a*]pyridine **1m** (45.2 mg, 0.20 mmol, 1.0 equiv), LiO*t*-Bu (6.4 mg, 0.08 mmol, 0.4 equiv), CD_3CN (125 μL, 2.40 mmol, 12 equiv) and 1,4-dioxane (1.0 mL).. Flash column chromatography on silica gel (30% EtOAc in hexanes) afforded **2m** as white solid (42.7 mg, 0.188 mmol, 94% yield, 97% D, C3:C7 = 11:1). In an independent experiment, 42.5 mg (94% yield, 97% D, C3:C7 = 11:1) were obtained, giving an average yield of 94%. Deuterium incorporation was determined by ^{1}H NMR, based on comparison with non-deuterated C4–H position. M.P.:137°C– 144°C. ^{1}H NMR (500 MHz, $CDCl_3$) δ 8.72 (dd, *J* = 7.3, 1.0 Hz, 0.91H), 8.22 (dd, *J* = 5.0, 1.9 Hz, 1.0H), 8.07 (d, *J* = 0.9 Hz, 0.03H), 7.88 (dd, *J* = 1.8, 1.0 Hz, 1H), 7.69 (dd, *J* = 7.3, 1.9 Hz, 1H), 7.22 (d, *J* = 1.8 Hz, 1H), 7.02 (dd, *J* = 7.3, 5.0 Hz, 1H), 4.00 (s, 3H).^{13}C NMR (126 MHz, $CDCl_3$) δ 160.8, 147.4, 138.4, 134.5, 133.8, 126.0(t, *J* = 29.4 Hz), 124.4, 121.4, 117.4, 117.2, 117.1, 53.8. IR (neat): 3090, 2962, 2922, 1638, 1579, 1459, 1401, 1286, 1209, 1008, 786 cm^{-1}. HRMS (ESI) m/z: $[M+H]^+$ Calcd for $C_{12}H_{10}DN_4O$ 228.0996; Found 228.1001.

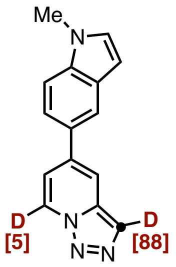

5-(1-methyl-1*H*-indol-7-yl)-[1,2,3]triazolo[1,5-*a*]pyridine-3,7-d_2 (2n). Following **GP1**, utilizing 5-(1-methyl-1*H*-indol-5-yl)-[1,2,3]triazolo[1,5-*a*]pyridine **1n** (49.6 mg, 0.20 mmol, 1.0 equiv), LiO*t*-Bu (12.8 mg, 0.16 mmol, 0.8 equiv), CD_3CN (125 μL, 2.40 mmol, 12 equiv) and 1,4-dioxane (1.0 mL) stirred at 40 ºC in an oil bath. Flash column chromatography (silica gel: 50% EtOAc in hexanes) afforded **2n** as yellow solid. (47.5 mg, 0.19 mmol, 95% yield, 88% D, C3:C7 = 17.5:1). In an independent experiment, 47.5 mg (95% yield, 88% D, C3:C7 = 17.5:1) were obtained, giving an average yield of 95%. Deuterium incorporation was determined by ^{1}H NMR, based on comparison with non-deuterated C4–H position. M.P.:206 °C– 209 °C. ^{1}H NMR (400 MHz, $CDCl_3$) δ 8.76 (dd, *J* = 7.3, 1.0 Hz, 0.95H), 8.06 (d, *J* = 1.0 Hz, 0.12H), 7.92– 7.86(m, 2H), 7.51 (dd, *J* = 8.5, 1.8 Hz, 1H), 7.45 – 7.42 (m, 1H), 7.34 (dd, *J* = 7.3, 1.9 Hz, 1H), 7.14 (d, *J* = 3.1 Hz, 1H), 6.57 (dd, *J* = 3.1, 0.9 Hz, 1H), 3.85 (s, 3H). ^{13}C NMR (126 MHz, $CDCl_3$) δ 140.2, 137.0, 134.5, 130.3, 129.3, 129.2, 125.4(t, *J* = 27.7Hz), 125.0, 120.8, 119.7, 116.2, 113.6, 110.1, 101.8, 33.2. IR (neat): 3079, 2921, 1727, 1609, 1507, 1459, 1338, 1248, 1157, 1080, 852, 791, 738 cm^{-1}. HRMS (ESI) m/z: [M+H]$^+$ Calcd for $C_{15}H_{12}DN_4$ 250.1203; Found 250.1209.

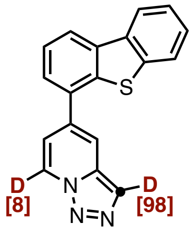

5-(dibenzo[b,d]thiophen-4-yl)-[1,2,3]triazolo[1,5-a]pyridine-3,7-d_2 (2o). Following **GP1**, utilizing 5-(dibenzo[b,d]thiophen-4-yl)-[1,2,3]triazolo[1,5-a]pyridine **1o** (60.2 mg, 0.2 mmol, 1.0 equiv), LiO*t*-Bu (12.8 mg, 0.16 mmol, 0.8 equiv), CD_3CN (125 μL, 2.4 mmol, 12 equiv) and 1,4-dioxane (1.0 mL). Flash column chromatography on silica gel (50% EtOAc in hexanes) afforded 2o as yellow solid (59.8 mg, 0.198 mmol, 99% yield, 98% D, C3:C7 = 12:1). In an independent experiment, 59.8 mg (99% yield, 98% D, C3:C7 = 12:1) were obtained, giving an average yield of 99%. Deuterium incorporation was determined by ^{1}H NMR, based on comparison with non-deuterated C4–H position· M.P.:183 °C– 187 °C. ^{1}H NMR (500 MHz, $CDCl_3$) δ 8.86 (dd, *J* = 7.2, 1.1 Hz, 0.92H),

8.27 – 8.19 (m, 2H), 8.16 (d, $J = 0.9$ Hz, 0.02H), 8.10 (dd, $J = 1.8$, 1.0 Hz, 1H), 7.91 – 7.83 (m, 1H), 7.61 (t, J = 7.6 Hz, 1H), 7.57 – 7.48 (m, 3H), 7.37 (dd, $J = 7.2$, 1.8 Hz, 1H). ^{13}C NMR (126 MHz, $CDCl_3$) δ 139.2, 138.3, 138.1, 136.9, 135.6, 133.9, 133.7, 127.5, 126.9, 126.2 (t, $J = 29.3$ Hz), 125.5, 125.5, 125.0, 122.9, 122.1, 122.0, 116.5, 116.4. IR (neat): 3054, 2922, 2853, 1721, 1637, 1438, 1387, 1278, 1160, 788, 754 cm^{-1}. HRMS (ESI) m/z: $[M+H]^+$ Calcd for $C_{18}H_{11}DN_3S$ 303.0815; Found 303,0813.

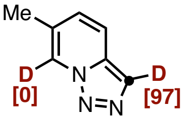

6-methyl-[1,2,3]triazolo[1,5-a]pyridine-3-*d* (2p). Following **GP1**, utilizing 6-methyl-[1,2,3]triazolo[1,5-a]pyridine **1p** (26.6 mg, 0.2 mmol, 1.0 equiv), LiO*t*-Bu (16.0 mg, 0.2 mmol, 1.0 equiv), CD_3CN (125 µL, 2.4 mmol, 12 equiv) and 1,4-dioxane (1.0 mL).. Flash column chromatography on silica gel (50% EtOAc in hexanes) afforded **2p** as white solid (26.3 mg, 0.196 mmol, 98% yield, 97% D, C3:C7 = 99:1). In an independent experiment, 26.3 mg (98% yield, 97% D, C3:C7 = 99:1) were obtained, giving an average yield of 98%. Deuterium incorporation was determined by ^{1}H NMR, based on comparison with non-deuterated C4–H position. M.P.:93 °C – 99 °C. ^{1}H NMR (500 MHz, $CDCl_3$) δ 8.50 (q, $J = 1.2$ Hz, 1H), 7.97 (d, $J = 1.0$ Hz, 0.03H), 7.60 (dd, $J = 9.0$, 1.0 Hz, 1H), 7.07 (dd, $J = 9.0$, 1.4 Hz, 1H), 2.38 (d, $J = 1.2$ Hz, 3H). ^{13}C NMR (126 MHz, $CDCl_3$) δ 132.4, 128.5, 125.6, 125.2 (t, $J = 30.2$ Hz), 122.9, 117.1, 18.3. IR (neat): 3072, 3038, 2923, 2852, 2335, 1932, 1641, 1516, 1176, 995, 800 cm^{-1}. HRMS (ESI) m/z: $[M+H]^+$ Calcd for $C_7H_7DN_3$ 135.0781; Found 135.0779.

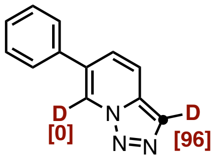

6-phenyl-[1,2,3]triazolo[1,5-a]pyridine-3-*d* (2q). Following **GP1**, utilizing 6-phenyl-[1,2,3]triazolo[1,5-a]pyridine **1q** (39.0 mg, 0.2 mmol, 1.0 equiv), LiO*t*-Bu (6.4 mg, 0.08 mmol, 0.4 equiv) CD_3CN (125 µL, 2.4 mmol, 12 equiv) and 1,4-dioxane (1.0 mL) 8 h. Flash column chromatography on silica gel (50% EtOAc in hexanes) afforded **2q** as white solid (38.5 mg, 0.196 mmol, 98% yield, 96% D, C3:C7 = 99:1). In an independent experiment, 38.5 mg (98% yield, 96% D, C3:C7 = 99:1) were obtained, giving an average yield of 98%. Deuterium incorporation was determined by 1H NMR, based on comparison with non-deuterated C4–H position. M.P.:98 °C– 100 °C. ^{1}H NMR (400 MHz, $CDCl_3$) δ 8.91 (t, $J = 1.3$ Hz, 1H), 8.08 (s, 0.04H), 7.78 (dd, $J = 9.2$, 1.0 Hz, 1H), 7.64 – 7.57 (m, 2H), 7.55 – 7.48 (m, 3H), 7.48 – 7.41 (m, 1H). ^{13}C NMR (75 MHz, CDC13) δ

136.2, 132.9, 129.9, 129.5(2C), 128.9, 127.3(2C), 126.3, 125.6, 122.3, 117.8. IR (neat): 3063, 2922, 2333, 1485, 1452, 1330, 1196, 1122, 752, 748, 622 cm^{-1}. HRMS (ESI) m/z: $[M+H]^+$ Calcd for $C_{12}H_9DN_3$ 197.0937; Found 197.0931.

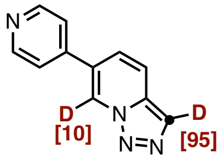

6-(pyridin-4-yl)-[1,2,3]triazolo[1,5-a]pyridine-3,7-*d*$_2$ (2r). Following **GP1**, utilizing 6-(pyridin-4-yl)-[1,2,3]triazolo[1,5-a]pyridine **1r** (39.2 mg, 0.2 mmol, 1.0 equiv), LiO*t*-Bu (6.4 mg, 0.08 mmol, 0.4 equiv), CD_3CN (125 μL, 2.4 mmol, 12 equiv) and 1.4-dioxane (4.0 mL) . Flash column chromatography on silica gel (50% EtOAc in hexanes) afforded **2r** as white solid (38.7 mg, 0.196 mmol, 98% yield, 95% D, C3:C7 = 9.5:1). In an independent experiment, 38.5 mg (98% yield, 95% D, C3:C7 = 9.5:1) were obtained, giving an average yield of 98% Deuterium incorporation was determined by ^1H NMR, based on comparison with non-deuterated C4–H position. M.P.:180 °C– 183 °C. ^1H NMR (300 MHz, $CDCl_3$) δ 9.06 – 9.00 (m, 0.88 H), 8.77 (d, J = 5.8 Hz, 2H), 8.13 (s, 0.05H), 7.87 (dd, J = 9.2, 1.0 Hz, 1H), 7.59 – 7.50 (m, 3H). ^{13}C NMR (101 MHz, $CDCl_3$) δ 151.0(2C), 143., 133.2, 127.0, 125.6 (t, J = 30.2 Hz), 125.0, 123.1, 121.5 (2C), 118.6. IR (neat): 3074, 3031, 2921, 2333, 2289, 1600, 1552, 801 cm^{-1}. HRMS (ESI) m/z: $[M+H]^+$ Calcd for $C_{11}H_8DN_4$ 198.0890; Found 198.0884.

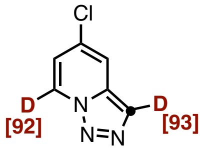

5-chloro-[1,2,3]triazolo[1,5-a]pyridine-3,7-*d*$_2$ (2s). Following **GP1**, utilizing 5-chloro-[1,2,3]triazolo[1,5-a]pyridine **1s** (30.7 mg, 0.2 mmol, 1.0 equiv), LiO*t*-Bu (6.4 mg, 0.08 mmol, 0.4 equiv), CD_3CN (125 μL, 2.4 mmol, 12 equiv) and 1.4-dioxane (1.0 mL). Flash column chromatography on silica gel (30% EtOAc in hexanes) afforded **2s** as white solid (30.0 mg, 0.194 mmol, 97% yield, 93% D, C3:C7 = 1:1). In an independent experiment, 30.0 mg (97% yield, 93% D, C3:C7 = 1:1) were obtained, giving an average yield of 97%. Deuterium incorporation was determined by ^1H NMR, based on comparison with non-deuterated C4–H position. M.P.:100 °C – 102 °C. ^1H NMR (400 MHz, $CDCl_3$) δ 8.67 (dd, J = 7.4, 0.9 Hz, 0.08H), 8.01 (s, 0.07H), 7.73 (d, J = 2.1 Hz, 1H), 6.96 – 6.92(m, 1H). ^{13}C NMR (101 MHz, $CDCl_3$) δ 133.9, 131.8, 126.1 – 124.8 (m)(2C), 116.7, 116.6. IR (neat):

3061, 2325, 1615, 1519, 1481, 1333, 1260, 949, 855, 700 cm^{-1}. HRMS (ESI) m/z: $[M+H]^+$ Calcd for $C_6H_3ClD_2N_3$ 156.0298; Found 156.0293.

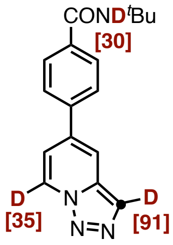

4-([1,2,3]triazolo[1,5-*a*]pyridin-5-yl-3,7-*d*2)-*N*-(*tert*-butyl)benzamide (2t). Following **GP1**, utilizing 4-([1,2,3]triazolo[1,5-*a*]pyridin-5-yl)-*N*-(*tert*-butyl)benzamide **1t** (58.8 mg, 0.2 mmol, 1.0 equiv), LiO*t*-Bu (12.8 mg, 0.2 mmol, 0.8 equiv), CD_3CN (125 µL, 2.4 mmol, 12 equiv) and 1.4-dioxane (1.0 mL) stirred at 40 ºC in an oil bath. Flash column chromatography on silica gel (30% EtOAc in hexanes) afforded **2t** as the white solid (58.4 mg, 0.198 mmol, 99% yield, 91% D, C3:C7 = 2.6:1). In an independent experiment, 58.5 mg (99% yield, 91% D, C3:C7 = 2.6:1) were obtained, giving an average yield of 99%. Deuterium incorporation was determined by ^{1}H NMR, based on comparison with non-deuterated C4–H position. M.P.:204 °C – 208 °C. ^{1}H NMR (500 MHz, $CDCl_3$) δ 8.80 (dd, J = 7.3, 1.0 Hz, 0.65 H), 8.12 (d, J = 0.8 Hz, 0.09 H), 7.93 – 7.91 (m, 1H), 7.87 – 7.80 (m, 2H), 7.69 (d, J = 8.5 Hz, 2H), 7.27 – 7.23 (m, 1H), 6.00 (s, 0.70 H), 1.50 (s, 9H). ^{13}C NMR (126 MHz, $CDCl_3$) δ 166.2, 140.4, 137.6, 136.3, 134.0, 127.8, 127.1, 126.5, 125.5, 115.3, 115.1, 115.0, 115.0, 52.0, 29.0. IR (neat): 3372, 2965, 2924, 1644, 1530 1452, 1362, 1308, 851, 796, 769 cm^{-1}. HRMS (ESI) m/z: $[M+H]^+$ Calcd for $C_{17}H_{16}D_3N_4O$ 298.1747; Found 298.1742.

Selective C7-Deuteration of [1,2,3]triazolo[1,5-a]pyridine

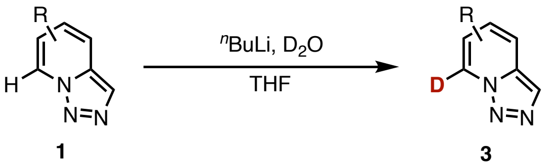

General procedure 2 (**GP2**): An oven-dried 8 mL vial equipped with a stirring magnetic bar was charged with the [1,2,3]triazolo[1,5-*a*]pyridine **1** (0.2 mmol, 1.0 equiv). The vial was evacuated and backfilled with argon for at least three times and THF (0.5 mL, 0.4 M) was added. The solution was cool down to -78 ºC and ^{n}BuLi (100 µL, 0.25 mmol, 1.25 equiv) was added dropwise to the mixture and stirred for 20 min. Afterwards, D_2O (36 µL, 2.4 mmol, 10 equiv) was added at -78 ºC and stirred for further 10 min. The reaction was warmed up to rt and stirred for 1 h. The reaction mixture was diluted with EtOAc,

filtered through a silica plug and concentrated in vacuo. The residue was purified by flash column chromatography on silica gel.

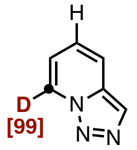

[1,2,3]triazolo[1,5-*a*]pyridine-7-*d* (3a). Following **GP2**, utilizing [1,2,3]triazolo[1,5-*a*]pyridine **1a** (23.8 mg, 0.2 mmol, 1.0 equiv), ^{n}BuLi (100 μL, 0.25 mmol, 1.25 equiv), D_2O (36 μL, 2.4 mmol, 10 equiv) and THF (0.5 mL). Flash column chromatography on silica gel (50% EtOAc in hexanes) afforded **3a** as amorphous white solid (23.0 mg, 0.19 mmol, 95% yield, 99% D, C7:C3 = 99:1). In an independent experiment, 23.0 mg (95% yield, 99% D, C7:C3 = 99:1) were obtained, giving an average yield of 95%. Deuterium incorporation was determined by ^{1}H NMR, based on comparison with non-deuterated C4–H position. ^{1}H NMR (300 MHz, $CDCl_3$) δ 8.78 (dd, J = 7.1, 1.1 Hz, 0.01H), 8.10 (s, 1H), 7.77 (dd, J = 8.9, 1.2 Hz, 1H), 7.33 – 7.27 (m, 1H), 7.01 (dq, J = 6.6, 1.1 Hz, 1H). ^{13}C NMR (75 MHz, $CDCl_3$) δ 133.7, 125.6, 125.2, 124.7 (t, J = 28.7 Hz), 117.9, 115.1. IR (neat): 3130, 3086, 2314, 1624, 1495, 1324, 1103, 975, 810, 736 cm^{-1}. HRMS (ESI) m/z: $[M+H]^+$ Calcd for $C_6H_5DN_3$ 121.0624; Found 121.0619.

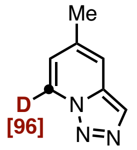

5-methyl-[1,2,3]triazolo[1,5-*a*]pyridine-7-*d* (3b). Following **GP2**, utilizing using 6-methyl-[1,2,3]triazolo[1,5-*a*]pyridine **1b** (26.6 mg, 0.2 mmol, 1.0 equiv), ^{n}BuLi (100 μL, 0.25 mmol, 1.25 equiv), D_2O (36 μL, 2.4 mmol, 10 equiv) and THF (0.5 mL). Flash column chromatography on silica gel (50% EtOAc in hexanes) afforded **3b** as yellow solid (25.0 mg, 0.196 mmol, 95% yield, 96% D, C7:C3 = 99:1). In an independent experiment, 25.0 mg (95% yield, 96% D, C7:C3 = 99:1) were obtained, giving an average yield of 95%. Deuterium incorporation was determined by ^{1}H NMR, based on comparison with non-deuterated C4–H position. M.P.:63 ºC – 65 ºC. ^{1}H NMR (500 MHz, $CDCl_3$) δ 8.37 (d, J = 5.1 Hz, 0.04H), 7.89 (s, 1H), 7.45 – 7.42 (m, 1H), 6.77 (s, 1H), 2.41 (d, J = 1.2 Hz, 1H). ^{13}C NMR (126 MHz, $CDCl_3$) δ 136.3, 134.1, 124.5, 124.2, 117.9, 115.9, 21.3. IR (neat): 3061, 2173, 1631, 1388, 1193, 890, 796, 754 cm^{-1}. HRMS (ESI) m/z: $[M+H]^+$ Calcd for $C_7H_7DN_3$ 135.0781; Found 135.0781.

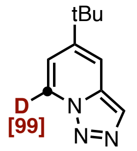

5-(*tert*-butyl)-[1,2,3]triazolo[1,5-*a*]pyridine-7-*d* (3c). Following **GP2**, utilizing 5-(tert-butyl)-[1,2,3]triazolo[1,5-a]pyridine **1c** (35.0 mg, 0.2 mmol, 1.0 equiv), ^{n}BuLi (100 μL, 0.25 mmol, 1.25 equiv), D_2O (36 μL, 2.4 mmol, 10 equiv) and THF (0.5 mL). Flash column chromatography on silica gel (10% EtOAc in hexanes to 30% EtOAc in hexanes) afforded **3c** as white solid (33.8 mg, 0.192 mmol, 96% yield, 99% D, C7:C3 = 99:1). In an independent experiment, 33.8 mg (96% yield, 99% D, C7:C3 = 99:1) were obtained, giving an average yield of 96%. Deuterium incorporation was determined by ^{1}H NMR, based on comparison with non-deuterated C4–H position. M.P.:67 °C– 71 °C. ^{1}H NMR (500 MHz, CDCl3) δ 8.65 (d, J = 7.4 Hz, 0.01H), 7.97 (s, 1H), 7.58 (d, J = 1.9 Hz, 1H), 7.05 – 7.01 (m, 1H), 1.36 (s, 9H). ^{13}C NMR (126 MHz, $CDCl_3$) δ 149.3, 134.0, 125.3, 124.4(t, J = 29.1 Hz), 114.8, 112.0, 35.1, 30.5. IR (neat): 3079, 2963, 2869, 2279, 1634, 1474, 1367, 1240, 966, 880, 745 cm^{-1}. HRMS (ESI) m/z: $[M+H]^+$ Calcd for $C_{10}H_{13}DN_3$ 177.1250; Found 177.1249.

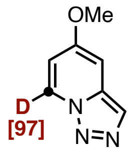

5-methoxy-[1,2,3]triazolo[1,5-*a*]pyridine-7-*d* (3d). Following **GP2**, utilizing 6-methyl-[1,2,3]triazolo[1,5-a]pyridine **1d** (29.8mg, 0.20 mmol, 1.0 equiv), ^{n}BuLi (100 μL, 0.25 mmol, 1.25 equiv), D_2O (36 μL, 2.4 mmol, 10 equiv) and THF (0.5 mL). Flash column chromatography on silica gel (50% EtOAc in hexanes) afforded **3d** as white solid (22.8 mg, 0.152 mmol, 76% yield, 97% D, C7:C3 = 99:1). In an independent experiment, 23.0 mg (76% yield, 97% D, C7:C3 = 99:1) were obtained, giving an average yield of 76%. Deuterium incorporation was determined by ^{1}H NMR, based on comparison with non-deuterated C4–H position. M.P.:99 °C– 101°C. ^{1}H NMR (500 MHz, $CDCl_3$) δ 8.55 (d, J = 7.5 Hz, 0.03 H), 7.84 (s, 1H), 6.85 (d, J = 2.5 Hz, 1H), 6.74 – 6.64 (m, 1H), 3.88 (s, 3H). ^{13}C NMR (126 MHz, $CDCl_3$) δ 157.6, 135.1, 125.8 (t, J = 29.0 Hz), 124.3, 110.6, 93.6, 55.9. IR (neat): 3118, 3080, 2915, 2829, 2309, 1636, 1543, 1459, 1309, 1202, 1154, 969, 831, 734 cm^{-1}. HRMS (ESI) m/z: $[M+H]^+$ Calcd for $C_7H_7DN_3O$ 151.0730; Found 151.0729.

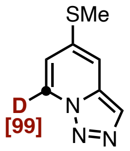

5-(methylthio)-[1,2,3]triazolo[1,5-*a*]pyridine-7-*d* (3e). Following **GP2**, utilizing 5-(pyrrolidin-1-yl)-[1,2,3]triazolo[1,5-*a*]pyridine **1e** (33.0 mg, 0.20 mmol, 1.0 equiv), ^{n}BuLi (100 μL, 0.25 mmol, 1.25 equiv), D_2O (36 μL, 2.4 mmol, 10 equiv) and THF (0.5 mL). Flash column chromatography on silica gel (50% EtOAc in hexanes to 100% EtOAc) afforded **3e** as yellow solid (32.9 mg, 0.198 mmol, 99% yield, 99% D, C7:C3 = 99:1). In an independent experiment, 33.0 mg (99% yield, 99% D, C7:C3 = 99:1) were obtained, giving an average yield of 99%. Deuterium incorporation was determined by ^{1}H NMR, based on comparison with non-deuterated C4–H position. M.P.:77 °C– 80 °C. ^{1}H NMR (500 MHz, $CDCl_3$) δ 8.54 (d, J = 7.3 Hz, 0.01H), 7.87 (s, 1H), 7.28 (d, J = 2.0 Hz, 1H), 6.86 – 6.70 (m, 1H), 2.53 (s, 3H); ^{13}C NMR (126 MHz, $CDCl_3$) δ 138.7, 134.3, 124.2 (t, J = 29.0), 124.1, 115.1, 110.1, 15.1 (3C). IR (neat): 3124, 3063, 2918, 2853, 2319, 1614, 1511, 1317, 1068, 953, 833, 800, 706 cm^{-1}. HRMS (ESI) m/z: $[M+H]^+$ Calcd for $C_7H_7DN_3S$ 167.0502; Found 167.0502.

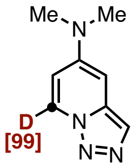

***N*,*N*-dimethyl-[1,2,3]triazolo[1,5-*a*]pyridin-5-amine-7-*d* (3f).** Following **GP2**, utilizing 5-(pyrrolidin-1-yl)-[1,2,3]triazolo[1,5-*a*]pyridine **1f** (32.4 mg, 0.2 mmol, 1.0 equiv), ^{n}BuLi (100 μL, 0.25 mmol, 1.25 equiv), D_2O (36 μL, 2.4 mmol, 10 equiv) and THF (0.5 mL). Flash column chromatography on silica gel (50% EtOAc in hexanes to 100% EtOAc) afforded **3f** as green solid (27.7 mg, 0.17 mmol, 85% yield, 99% D, C7:C3 = 99:1). In an independent experiment, 28.0 mg (85% yield, 99% D, C7:C3 = 99:1) were obtained, giving an average yield of 85%. Deuterium incorporation was determined by ^{1}H NMR, based on comparison with non-deuterated C4–H position. M.P.:122 °C–124 °C. ^{1}H NMR (500 MHz, $CDCl_3$) δ 8.48 (d, J = 7.8 Hz, 0.01H), 7.66 (s, 1H), 6.63 (d, J = 2.6 Hz, 1H), 6.46 (d, J = 2.6 Hz, 1H), 3.03 (s, 6H). ^{13}C NMR (126 MHz, $CDCl_3$) δ 147.6, 136.0, 124.9(t, J = 28.6 Hz), 122.3, 106.7, 91.7, 40.4 (6C). IR (neat): 3112, 2921, 2808, 2281, 1634, 1529, 1433, 1181, 969, 918, 806, 730 cm^{-1}. HRMS (ESI) m/z: $[M+H]^+$ Calcd for $C_8H_{10}DN_4$ 164.1046; Found 164.1046.

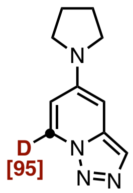

5-(pyrrolidin-1-yl)-[1,2,3]triazolo[1,5-*a*]pyridine-7-*d* (3g). Following **GP2**, utilizing 5-(pyrrolidin-1-yl)-[1,2,3]triazolo[1,5-*a*]pyridine **1g** (37.6 mg, 0.20 mmol, 1.0 equiv), ^{n}BuLi (100 µL, 0.25 mmol, 1.25 equiv), D_2O (36 µL, 2.4 mmol, 10 equiv) and THF (0.5 mL). Flash column chromatography on silica gel (50% EtOAc in hexanes to 100% EtOAc) afforded **3g** as white solid (37.5 mg, 0.198 mmol, 99% yield, 95% D, C7:C3 = 99:1). In an independent experiment, 37.0 mg (99% yield, 95% D, C7:C3 = 99:1) were obtained, giving an average yield of 99%. Deuterium incorporation was determined by ^{1}H NMR, based on comparison with non-deuterated C4–H position. M.P.:138 °C– 145 °C. ^{1}H NMR (500 MHz, $CDCl_3$) δ 8.45 (dt, *J* = 7.7, 0.9 Hz, 0.05H), 7.61 (s, 1H), 6.48 (d, *J* = 2.4 Hz, 1H), 6.29 (d, *J* = 2.4 Hz, 1H), 3.37 – 3.30 (m, 4H), 2.06 – 2.01 (m, 4H). ^{13}C NMR (126 MHz, $CDCl_3$) δ 144.9, 136.1, 124.9 (t, *J* = 29.8 Hz), 121.7, 106.9, 90.2, 47.9 (2C), 25.5 (2C). IR (neat): 2966, 2860, 2284, 1633, 1459, 1377, 1160, 972, 800, 729 cm^{-1}. HRMS (ESI) m/z: $[M+H]^+$ Calcd for $C_{10}H_{12}DN_4$ 190.1203; Found 190.1206.

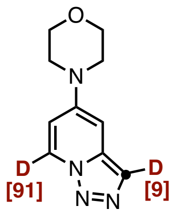

4-([1,2,3]triazolo[1,5-*a*]pyridin-5-yl-3,7-*d*2)morpholine (3h). Following **GP2**, utilizing 4-([1,2,3]triazolo[1,5-a]pyridin-5-yl)morpholine **1h** (40.8 mg, 0.2 mmol, 1.0 equiv), ^{n}BuLi (100 µL, 0.25 mmol, 1.25 equiv), D_2O (36 µL, 2.4 mmol, 10 equiv) and THF (0.5 mL). Flash column chromatography on silica gel (50% EtOAc in hexanes to 100% EtOAc) afforded **3h** as white solid (35.0 mg, 0.17 mmol, 85% yield, 91% D, C7:C3 = 10:1). In an independent experiment, 35.0 mg (85% yield, 91% D, C7:C3 = 10:1) were obtained, giving an average yield of 85%. Deuterium incorporation was determined by ^{1}H NMR, based on comparison with non-deuterated C4–H position. M.P.:136°C– 142 °C. ^{1}H NMR (500 MHz, $CDCl_3$) δ 8.52 (dd, *J* = 8.6, 1.0 Hz, 0.09H), 7.75 (s, 0.91H), 6.73 (s, 2H), 3.98 – 3.74 (m, 4H), 3.35 – 3.07 (m, 4H). ^{13}C NMR (126 MHz, $CDCl_3$) δ 148.5, 135.2, 125.2 (t, *J* = 29.8 Hz), 123.6, 108.8, 96.1, 66.5 (2C), 48.5 (2C). IR (neat): 3102, 2850, 2290, 1632, 1521, 1444, 1349, 1198, 1119, 973, 903, 826 cm^{-1}. HRMS (ESI) m/z: $[M+H]^+$ Calcd for $C_{10}H_{12}DN_4O$ 206.1152; Found 206.1144.

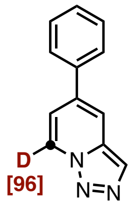

5-phenyl-[1,2,3]triazolo[1,5-*a*]pyridine-7-*d* (3i). Following **GP2**, utilizing 5-phenyl-[1,2,3]triazolo[1,5-*a*]pyridine **1i** (39.0 mg, 0.20 mmol, 1.0 equiv), ^{n}BuLi (100 μL, 0.25 mmol, 1.25 equiv), D_2O (36 μL, 2.4 mmol, 10 equiv) and THF (0.5 mL). Flash column chromatography on silica gel (10% EtOAc in hexanes to 30% EtOAc in hexanes) afforded **3i** as white solid (38.0 mg, 0.194 mmol, 97% yield, 96% D, C7:C3 = 99:1). In an independent experiment, 38.0 mg (97% yield, 96% D, C7:C3 = 99:1) were obtained, giving an average yield of 97%. Deuterium incorporation was determined by ^{1}H NMR, based on comparison with non-deuterated C4–H position. M.P.:115 °C–118 °C. ^{1}H NMR (500 MHz, $CDCl_3$) δ 8.77 (dt, *J* = 7.3, 1.0 Hz, 0.04H), 8.09 (s, 1H), 7.86 (d, *J* = 1.9 Hz, 1H), 7.67 – 7.60 (m, 2H), 7.53 – 7.47 (m, 2H), 7.46 – 7.41 (m, 1H), 7.24 (d, *J* = 1.8 Hz, 1H). ^{13}C NMR (126 MHz, $CDCl_3$) δ 138.7, 137.8, 134.2, 129.3 (2C), 129.0, 127.0 (2C), 126.1, 125.0(t, *J* = 28.9) 115.4, 114.4. IR (neat): 3075, 3039, 2923, 2853, 1631, 1504, 1327, 1200, 1109, 979, 886, 758, 688, 669 cm^{-1}. HRMS (ESI) m/z: $[M+H]^+$ Calcd for $C_{12}H_9DN_3$ 197.0937; Found 197.0935.

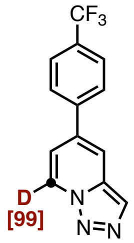

5-(4-(trifluoromethyl)phenyl)-[1,2,3]triazolo[1,5-*a*]pyridine-7-*d* (3j). Following **GP2**, utilizing 5-(4-(trifluoromethyl)phenyl)-[1,2,3]triazolo[1,5-*a*]pyridine **1j** (52.6 mg, 0.20 mmol, 1.0 equiv), ^{n}BuLi (100 μL, 0.25 mmol, 1.25 equiv), D_2O (36 μL, 2.4 mmol, 10 equiv) and THF (0.5 mL). Flash column chromatography on silica gel (10% EtOAc in hexanes to 30% EtOAc in hexanes) afforded **3j** as white solid (51.3 mg, 0.194 mmol, 97% yield, 99% D, C7:C3 = 99:1). In an independent experiment, 51.0 mg (97% yield, 99% D, C7:C3 = 99:1) were obtained, giving an average yield of 97%. Deuterium incorporation was determined by ^{1}H NMR, based on comparison with non-deuterated C4–H position. M.P.:135 °C– 137 °C. ^{1}H NMR (500 MHz, $CDCl_3$) δ 8.81 (d, *J* = 7.2 Hz, 0.01H), 8.14 (s, 1.0H), 7.92 (d, *J* = 1.9 Hz, 1.0H), 7.76 (s, 4.0H), 7.25 – 7.22 (m, 1.0H).^{13}C NMR (126 MHz, $CDCl_3$) δ 141.4, 137.1, 134.0, 131.0 (q, *J* = 32.7 Hz), 127.4 (2C), 126.6, 126.3 (q, *J* = 3.8 Hz), 125.4 (t, *J* = 29 Hz), 125.1, 123.0, 115.4, 115.0. ^{19}F NMR (471

MHz, $CDCl_3$) δ –62.7. IR (neat) 3116, 3052, 2924, 1615, 1521, 1320, 1156, 1111, 1069, 838 ,798, 731 cm^{-1}. HRMS (ESI) m/z: $[M+H]^+$ Calcd for $C_{13}H_8DF_3N_3$ 265.0811; Found 265.0812.

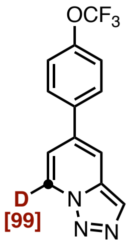

5-(4-(trifluoromethoxy)phenyl)-[1,2,3]triazolo[1,5-*a*]pyridine-7-*d* (3k). Following **GP2**, utilizing 5-(4-(trifluoromethyl)phenyl)-[1,2,3]triazolo[1,5-*a*]pyridine **1k** (55.8 mg, 0.20 mmol, 1.0 equiv), ^{n}BuLi (100 μL, 0.25 mmol, 1.25 equiv), D_2O (36 μL, 2.4 mmol, 10 equiv) and THF (0.5 mL). Flash column chromatography on silica gel (10% EtOAc in hexanes to 30% EtOAc in hexanes) afforded the **3k** as white solid (55.5 mg, 0.198 mmol, 99% yield, 99% D, C7:C3 = 99:1). In an independent experiment, 55.0 mg (98% yield, 99% D, C7:C3 = 99:1) were obtained, giving an average yield of 99%. Deuterium incorporation was determined by ^{1}H NMR, based on comparison with non-deuterated C4–H position. M.P.:100 °C– 102 °C. ^{1}H NMR (500 MHz, $CDCl_3$) δ 8.80 – 8.77 (m, 0.01H), 8.10 (s, 1H), 7.86 (d, J = 1.9 Hz, 1H), 7.68 – 7.65 (m, 2H), 7.36 – 7.33 (m, 2H), 7.20 (d, J = 1.9 Hz, 1H).^{19}F NMR (471 MHz, CDCl3) δ -57.8. ^{13}C NMR (126 MHz, $CDCl_3$) δ 149.8, 137.3, 136.6, 134.1, 128.6, 126.3, 125.2 (t, J = 29.0 Hz), 121.7, 115.1, 114.8. IR (neat): 3076, 2922, 1642, 1516, 1251, 1198, 1157, 855, 801 cm^{-1}. HRMS (ESI) m/z: $[M+H]^+$ Calcd for $C_{13}H_8DF_3N_3O$ 281.0760; Found 281.0766.

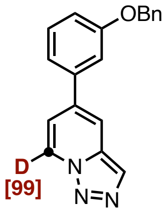

5-(3-(benzyloxy)phenyl)-[1,2,3]triazolo[1,5-*a*]pyridine-7-*d* (3l). Following **GP2**, utilizing (benzyloxy)phenyl)-[1,2,3]triazolo[1,5-*a*]pyridine **1l** (60.3 mg, 0.20 mmol, 1.0 equiv), *n*-BuLi (160.0 μL, 0.4 mmol, 2.0 equiv), D_2O (36 μL, 2.4 mmol, 10 equiv) and THF (0.5 mL).. Flash column chromatography on silica gel (10% EtOAc in hexanes to 30% EtOAc in hexanes) afforded **3l** as white solid (27.2 mg, 0.09 mmol, 45% yield, 99% D, C7:C3 = 99:1). In an independent experiment, 30.0 mg (50% yield, 99% D, C7:C3 = 99:1) was obtained, giving an average yield of 48%. Deuterium incorporation was determined by ^{1}H NMR, based on comparison with non-deuterated C4–H position. M.P.:86 °C– 89 °C. ^{1}H NMR (500 MHz, $CDCl_3$) δ 8.77 (d, J = 7.2 Hz, 0.01H), 8.10 (s,

1H), 7.86 (d, $J = 1.9$ Hz, 1H), 7.48 – 7.45 (m, 2H), 7.45 – 7.39 (m, 3H), 7.38 – 7.33 (m, 1H), 7.26 – 7.22 (m, 3H), 7.06 (ddd, $J = 8.3, 2.4, 1.0$ Hz, 1H), 5.15 (s, 2H). ^{13}C NMR (126 MHz, $CDCl_3$) δ 159.6, 139.4, 138.5, 136.7, 134.2, 130.5, 128.8(2C), 128.3, 127.6(2C), 126.2, 125.0, 119.7, 115.4, 115.1, 114.6, 114.1, 70.4. IR (neat): 3034, 2961, 2924, 1584, 1451, 1258, 1016, 870, 792, 693 cm^{-1}. HRMS (ESI) m/z: $[M+H]^+$ Calcd for $C_{19}H_{15}DN_3O$) 303.1356; Found 303.1350.

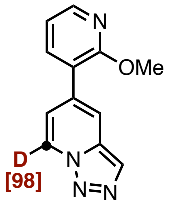

5-(2-methoxypyridin-3-yl)-[1,2,3]triazolo[1,5-*a*]pyridine-7-*d*₂ (3m). Following **GP2**, utilizing 5-(naphthalen-1-yl)-[1,2,3]triazolo[1,5-*a*]pyridine **1m** (22.6 mg, 0.10 mmol, 1.0 equiv), ^{n}BuLi (100 μL, 0.25 mmol, 1.25 equiv), D_2O (36 μL, 2.4 mmol, 10 equiv) and THF (3.0 mL). Flash column chromatography on silica gel (30% EtOAc in hexanes) afforded **3m** as white solid (22.2 mg, 0.098 mmol, 98% yield, 98% D, C7:C3 = 99:1). In an independent experiment, 22.5 mg (99% yield, 98% D, C7:C3 = 99:1) were obtained, giving an average yield of 99%. Deuterium incorporation was determined by ^{1}H NMR, based on comparison with non-deuterated C4–H position. M.P.:133 °C– 140 °C. ^{1}H NMR (400 MHz, $CDCl_3$) δ 8.73 (d, $J = 7.3$ Hz, 0.02H), 8.23 (dd, $J = 5.0, 1.9$ Hz, 1H), 8.08 (s, 1H), 7.89 (d, $J = 1.8$ Hz, 1H), 7.69 (dd, $J = 7.4, 1.9$ Hz, 1H), 7.23 (d, $J = 1.8$ Hz, 1H), 7.02 (dd, $J = 7.4, 5.0$ Hz, 1H), 4.01 (s, 3H). ^{13}C NMR (126 MHz, $CDCl_3$) δ 160.9, 147.4, 138.5, 134.6, 133.9, 126.2, 124.2 (t, $J = 29.0$ Hz), 121.5, 117.5, 117.2, 116.9, 53.9. IR (neat): 3122, 2922, 1629, 1579, 1457, 1401, 1286, 1208, 1008, 803, 778 cm^{-1}. HRMS (ESI) m/z: $[M+H]^+$ Calcd for $C_{12}H_{10}DN_4O$ 228.0996; Found 228.0982.

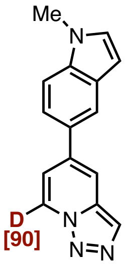

5-(1-methyl-1*H*-indol-5-yl)-[1,2,3]triazolo[1,5-*a*]pyridine-3,7-*d*₂ (3n). Following **GP2**, utilizing 5-(1-methyl-1*H*-indol-5-yl)-[1,2,3]triazolo[1,5-*a*]pyridine **1n** (24.8 mg, 0.10 mmol, 1.0 equiv), ^{n}BuLi (100 μL, 0.25 mmol, 1.25 equiv), D_2O (36 μL, 2.4 mmol, 10 equiv) and THF (5.0 mL). Flash column chromatography on silica gel (30% EtOAc in

hexanes) afforded **3n** as yellow solid (24.2 mg, 0.097 mmol, 97% yield, 90% D, C7:C3 = 99:1). In an independent experiment, 24.2 mg (97% yield, 90% D, C7:C3 = 99:1) were obtained, giving an average yield of 97%. Deuterium incorporation was determined by ^{1}H NMR, based on comparison with non-deuterated C4–H position. M.P.:201 °C– 206 °C. ^{1}H NMR (300 MHz, $CDCl_3$) δ 8.74 (dt, *J* = 7.4, 1.0 Hz, 0.10H), 8.05 (s, 1H), 7.92 – 7.85 (m, 2H), 7.54 – 7.39 (m, 2H), 7.34 – 7.29 (m, 1H), 7.13 (d, *J* = 3.1 Hz, 1H), 6.57 (dd, *J* = 3.1, 0.8 Hz, 1H), 3.84 (s, 3H).^{13}C NMR (126 MHz, $CDCl_3$) δ 140.2, 137.0, 134.5, 130.3, 129.3, 129.2, 125.6, 124.7 (t, *J* = 30.4 Hz), 120.8, 119.7, 116.0, 113.6, 110.1, 101.8, 33.2. IR (neat): 3102, 2922, 2852, 1610, 1508, 1340, 1199, 1080, 851, 741, 681 cm^{-1}. HRMS (ESI) m/z: $[M+H]^+$ Calcd for $C_{15}H_{12}DN_4$ 250.1203; Found 250.1201.

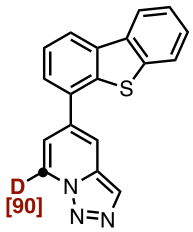

5-(dibenzo[*b*,*d*]thiophen-4-yl)-[1,2,3]triazolo[1,5-*a*]pyridine-7-*d* (3o). Following **GP2**, utilizing 5-(dibenzo[*b*,*d*]thiophen-4-yl)-[1,2,3]triazolo[1,5-*a*]pyridine **1o** (29.8 mg, 0.20 mmol, 1.0 equiv), *n*-BuLi (140.0 µL, 0.34 mmol, 1.7 equiv), D_2O (36 µL, 2.4 mmol, 10 equiv) and THF (0.5 mL). Flash column chromatography on silica gel (30% EtOAc in hexanes) afforded **3o** as yellow solid (36.2 mg, 0.12 mmol, 60% yield, 90% D, C7:C3 = 99:1). In an independent experiment, 36.0 mg (60% yield, 90% D, C7:C3 = 99:1) were obtained, giving an average yield of 60%. Deuterium incorporation was determined by ^{1}H NMR, based on comparison with non-deuterated C4–H position. ^{1}H-NMR. M.P.:189 °C– 195 °C. ^{1}H NMR (500 MHz, $CDCl_3$) δ 8.87 (dd, *J* = 7.2, 1.1 Hz, 0.10H), 8.27 – 8.19 (m, 2H), 8.16 (d, *J* = 0.9 Hz, 1H), 8.10 (dd, *J* = 1.8, 1.0 Hz, 1H), 7.91 – 7.83 (m, 1H), 7.61 (t, *J* = 7.6 Hz, 1H), 7.57 – 7.48 (m, 2H), 7.37 (dd, *J* = 7.2, 1.8 Hz, 1H). ^{13}C NMR (126 MHz, $CDCl_3$) δ 139.2, 138.3, 138.1, 136.9, 135.6, 134.0, 133.7, 127.5, 126.9, 126.4, 125.5, 125.0, 122.9, 122.1, 122.0, 116.6, 116.2. IR (neat): 3057, 2922, 2853, 1630, 1439, 1387, 1253, 1193, 1050, 890, 754 cm^{-1}. HRMS (ESI) m/z: $[M+H]^+$ Calcd for $C_{18}H_{11}DN_3S$ 303.0815; Found 303.0806.

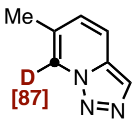

6-methyl-[1,2,3]triazolo[1,5-*a*]pyridine-7-*d* (3p). Following **GP2**, utilizing 6-methyl-[1,2,3]triazolo[1,5-*a*]pyridine **1p** (26.6 mg, 0.20 mmol, 1.0 equiv), ^{n}BuLi (100 µL, 0.25

mmol, 1.25 equiv), D_2O (36 µL, 2.4 mmol, 10 equiv) and THF (0.5 mL). Flash column chromatography on silica gel (50% EtOAc in hexanes) afforded **3p** as white solid (26.3 mg, 0.196 mmol, 98% yield, 87% D, C7:C3 = 99:1). In an independent experiment, 26.4 mg (98% yield, 87% D, C7:C3 = 99:1) were obtained, giving an average yield of 98%. Deuterium incorporation was determined by ^{1}H NMR, based on comparison with non-deuterated C4–H position. M.P.:90 °C– 96 °C. ^{1}H NMR (500 MHz, $CDCl_3$) δ 8.53 – 8.48 (m, 0.13H), 7.98 (s, 1H), 7.61 (d, J = 9.0 Hz, 1H), 7.08 (d, J = 9.0 Hz, 1H), 2.39 (s, 3H). ^{13}C NMR (126 MHz, CDCl3) δ 132.6, 128.8, 128.6, 125.5, 125.4, 117.1, 18.3. IR (neat): 3120, 3070, 2920, 2851, 2306, 1632, 1505, 1222, 1102, 848 cm^{-1}. HRMS (ESI) m/z: $[M+H]^+$ Calcd for $C_7H_7DN_3$ 135.0781; Found 135.0782.

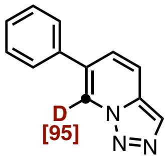

6-phenyl-[1,2,3]triazolo[1,5-*a*]pyridine-7-*d* (3q). Following **GP2**, utilizing 6-phenyl-[1,2,3]triazolo[1,5-*a*]pyridine **1q** (39.0 mg, 0.20 mmol, 1.0 equiv), ^{n}BuLi (100 µL, 0.25 mmol, 1.25 equiv), D_2O (36 µL, 2.4 mmol, 10 equiv) and THF (2.5 mL). Flash column chromatography on silica gel (30% EtOAc in hexanes) afforded **3q** as white solid (39.0 mg, 0.196 mmol, 99% yield, 95% D, C7:C3 = 99:1). In an independent experiment, 39.0 mg (99% yield, 95% D, C7:C3 = 99:1) were obtained, giving an average yield of 99%. Deuterium incorporation was determined by ^{1}H NMR, based on comparison with non-deuterated C4–H position. M.P.:99 °C– 102 °C. ^{1}H NMR (300 MHz, $CDCl_3$) δ 8.93 (s, 0.05H), 8.09 (s, 1H), 7.80 (d, J = 9.2 Hz, 1H), 7.69 – 7.59 (m, 2H), 7.56 – 7.41 (m, 4H). ^{13}C NMR (101 MHz, $CDCl_3$) δ 136.0, 132.8, 129.6, 129.4(2C), 128.8, 127.2(2C), 126.2, 125.5, 121.9 (t, J = 28.8 Hz), 117.7. IR (neat): 3118, 3063, 2921, 2282, 1625, 1480, 1445, 1227, 977, 741, 686 cm^{-1}. HRMS (ESI) m/z: $[M+H]^+$ Calcd for $C_{12}H_9DN_3$ 197,0937; Found 197.0935.

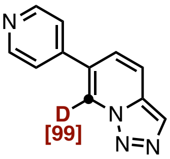

6-(pyridin-4-yl)-[1,2,3]triazolo[1,5-*a*]pyridine-7-*d* (3r). Following **GP2**, utilizing 6-(pyridin-4-yl)-[1,2,3]triazolo[1,5-*a*]pyridine **1r** (19.6 mg, 0.10 mmol, 1.0 equiv), *n*-BuLi (50 µL, 0.125 mmol, 1.25 equiv), D_2O (18 µL, 1.0 mmol, 10 equiv) and THF (4.0 mL). Flash column chromatography on silica gel (30% EtOAc in hexanes) afforded **3r** as white

solid (39.0 mg, 0.196 mmol, 99% yield, 99% D, C7:C3 = 99:1). In an independent experiment, 39.0 mg (99% yield, 99% D, C7:C3 = 99:1) were obtained, giving an average yield of 99%. Deuterium incorporation was determined by ^{1}H NMR, based on comparison with non-deuterated C4–H position. M.P.:142 °C– 143 °C. ^{1}H NMR (400 MHz, $CDCl_3$) δ 9.03 (s, 0.01H), 8.77 (d, J = 5.5 Hz, 2H), 8.13 (s, 1H), 7.87 (d, J = 9.2 Hz, 1H), 7.57 – 7.54 (m, 3H). ^{13}C NMR (126 MHz, $CDCl_3$) δ 150.9, 143.6, 133.3, 126.9, 125.9, 125.0, 122.9, 121.5, 118.6. IR (neat): 3119, 3035, 2922, 2290, 1622, 1600, 1481, 1402, 1287, 977, 827, 800, 764 cm^{-1}. HRMS (ESI) m/z: $[M+H]^+$ Calcd for $C_{11}H_8DN_4$ 198.0890; Found 198.0885.

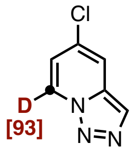

5-chloro-[1,2,3]triazolo[1,5-*a*]pyridine-7-*d* (3s). Following **GP2**, utilizing 5-chloro-[1,2,3]triazolo[1,5-*a*]pyridine **1s** (30.7 mg, 0.20 mmol, 1.0 equiv), ^{n}BuLi (100 μL, 0.25 mmol, 1.25 equiv), D_2O (36 μL, 2.4 mmol, 10 equiv) and THF (0.5 mL).. Flash column chromatography on silica gel (30% EtOAc in hexanes) afforded **3s** as white solid (25.0 mg, 0.16 mmol, 80% yield, 93% D, C7:C3 = 99:1). In an independent experiment, 26.0 mg (85% yield, 93% D, C7:C3 = 99:1) were obtained, giving an average yield of 83%. Deuterium incorporation was determined by ^{1}H NMR, based on comparison with non-deuterated C4–H position. M.P.: 98 °C–100 °C. ^{1}H NMR (400 MHz, $CDCl_3$) δ 8.67 (d, J = 7.4 Hz, 0.07H), 8.01 (s, 1H), 7.73 (d, J = 2.1 Hz, 1H), 6.94 (s, 1H). ^{13}C NMR (126 MHz, $CDCl_3$) δ 134.0, 131.9, 125.9, 125.4, 116.8, 116.7. IR (neat): 3119, 3062, 2318, 1616, 1285, 1198, 948, 857, 708 cm^{-1}. HRMS (ESI) m/z: $[M+H]^+$ Calcd for $C_6H_4DClN_3$ 155.0235; Found 155.0233.

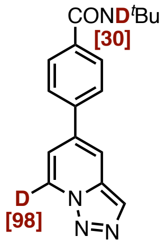

4-([1,2,3]triazolo[1,5-*a*]pyridin-5-yl-7-*d*)-*N*-(*tert*-butyl)benzamide (3t). Following **GP2**, utilizing 4-([1,2,3]triazolo[1,5-*a*]pyridin-5-yl)-*N*-(*tert*-butyl)benzamide **1t** (58.8 mg, 0.2 mmol, 1.0 equiv), *n*-BuLi (160.0 μL, 0.25 mmol, 2.0 equivD$_2$O (36 μL, 2.4 mmol, 10 equiv) and THF (1.0 mL). Flash column chromatography on silica gel (50% EtOAc in hexanes) afforded **3t** as white solid (50.2 mg, 0.17 mmol, 85% yield, 98% D, C7:C3 =

99:1). In an independent experiment, 50.2 mg (85% yield, 98% D, C7:C3 = 99:1) were obtained, giving an average yield of 85%. Deuterium incorporation was determined by ^{1}H NMR, based on comparison with non-deuterated C4–H position. M.P.: 205 °C– 208 °C. ^{1}H NMR (500 MHz, $CDCl_3$) δ 8.74 (d, *J* = 7.3 Hz, 0.02H), 8.07 (s, 1H), 7.87 (d, *J* = 1.9 Hz, 1H), 7.82 (d, *J* = 8.3 Hz, 2H), 7.66 – 7.60 (m, 2H), 7.21 (d, *J* = 1.9 Hz, 1H), 6.12 (s, 0.70H), 1.47 (s, 9H). ^{13}C NMR (126 MHz, $CDCl_3$) δ 166.2, 166.1, 140.2, 137.5, 136.2, 136.2, 134.0, 127.8, 127.0, 126.4, 125.1(t, *J* = 29.9 Hz)., 115.0, 114.9, 51.9, 29.0 (d, *J* = 2.8 Hz). IR (neat): 3370, 2967, 2499, 1643, 1532, 1308, 1205, 799, 773, 738, 670 cm^{-1}. HRMS (ESI) m/z: $[M+H]^+$ Calcd for $C_{17}H_{17}D_2N_4O$ 297.1684; Found 297.1671.

Unsuccessful substrates

In addition to the pyrazolotriazoles included into the manuscript, the deuteration was attempted with other substrates bearing different acidic functional groups such as OH- or NH_2. Unfortunately, a loss in site-selectivity was observed in the former (C3:C7 ratio of 1:1 regardless the equivalents of base utilized) whereas the no deuterium incorporation was observed in the latter.

Scheme S1. Unsuccessful substrates for the C3-deuteration

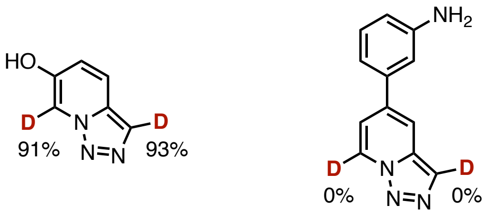

Scale-up and Synthetic applications

Gram scale synthesis of 2a

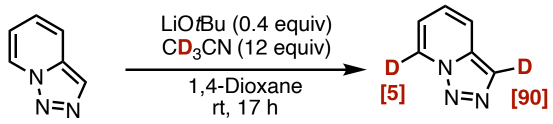

1a, 1.4 mmol

2a

An oven-dried 8 mL vial equipped with a stirring magnetic bar was charged with [1,2,3]triazolo[1,5-*a*]pyridine **1a** (167 mg,1.4 mmol, 1.0 equiv) and put into a nitrogen-filled glovebox. LiO*t*-Bu (44.8 mg, 0.568 mmol, 0.4 equiv) was added and the vial was taken-out from the glovebox. 1,4-dioxane (7.0 mL) was added, followed by CD_3CN (875 µL, 16.80 mmol, 12 equiv) and the reaction was stirred for 17 hours at room temperature. Afterwards, the reaction mixture was diluted with EtOAc, filtered through silica plug and

concentrated in vacuo. The residue was purified by flash column chromatography on silica gel to provide **2a** as amorphous white solid (164.5 mg, 1.372 mmol, 98% yield, 90% D, C3:C7 = 18:1). Deuterium incorporation was determined by ^{1}H NMR, based on comparison with non-deuterated C4–H position.

Synthetic applications

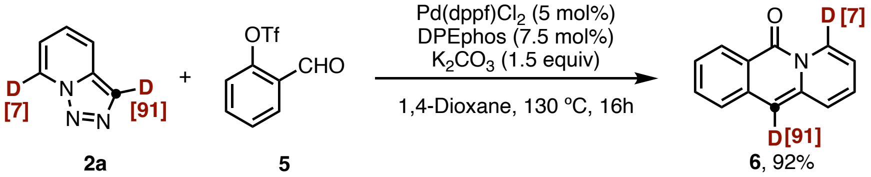

6*H*-pyrido[1,2-*b*]isoquinolin-6-one-4,11-*d*₂ (6).[14] An oven-dried Schlenk tube equipped with a stirring bar was charged with Pd(dppf)Cl_2 (7.3 mg, 0.01 mmol, 0.05 equiv), DPEphos (8.10 mg, 0.015 mmol, 0.075 equiv), K_2CO_3 (41.5 mg, 0.3 mmol, 1.5 equiv), [1,2,3]triazolo[1,5-*a*]pyridine-3,7-d_2 **2a** (24.0 mg, 0.2mmol, 1.0 equiv) and 2-formylphenyl trifluoromethanesulfonate **5** (76 mg, 0.75 mmol, 1.5 equiv), anhydrous 1,4-dioxane (3.0 mL) and the mixture was stirred at 130 ºC in an oil bath for 16 h. Afterwards, the reaction was cool down to room temperature, and the volatiles were removed in vacuo. The crude was purified by flash column chromatography on silica gel (5% EtOAc in hexanes with 5% Et_3N) to afford **6** as pale-yellow solid (35.3 mg, 0.182 mmol, 91% yield). In an independent experiment, 35.5 mg (92% yield) were obtained, giving an average yield of 92%. Deuterium incorporation was determined by ^{1}H NMR, based on comparison with non-deuterated C1–H position. M.P.:153 °C– 157 °C. ^{1}H NMR (400 MHz, $CDCl_3$) δ 8.82 (dt, J = 7.7, 1.1 Hz, 1H), 8.63 – 8.58 (m, 1H), 7.76 – 7.62 (m, 2H), 7.52 – 7.46 (m, 1H), 7.28 – 7.26 (m, 1H), 6.96 (ddd, J = 9.2, 6.2, 1.2 Hz, 1H), 6.84 (s, 0.09H), 6.64 – 6.59 (m, 1H). ^{13}C NMR (126 MHz, $CDCl_3$) δ 159.4, 137.2, 136.4, 132.4, 128.3, 126.2, 126.0, 125.6 (d, J = 1.9 Hz), 125.1, 120.2, 111.9, 101.1, 100.8. IR (neat): 3050, 1657, 1525, 1467, 1137, 761, 682 cm^{-1}. HRMS (ESI) m/z: $[M+H]^+$ Calcd for $C_{13}H_9DNO$ 197.0825; Found 197.0822.

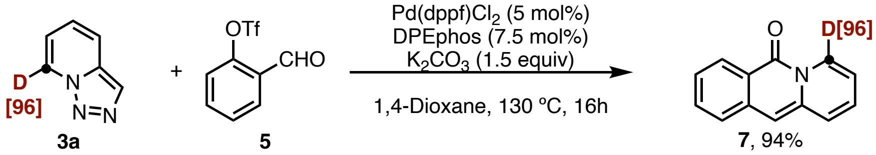

6*H*-pyrido[1,2-*b*]isoquinolin-6-one-4-*d* (7).[14] An oven-dried Schlenk tube equipped with a stirring magnetic bar was charged with Pd(dppf)Cl_2 (7.3 mg, 0.01 mmol, 0.05 equiv), DPEphos (8.10 mg, 0.015 mmol, 0.075 equiv), K_2CO_3 (41.5 mg, 0.3 mmol, 1.5

equiv), [1,2,3]triazolo[1,5-*a*]pyridine-7-*d* **3a** (24.0 mg, 0.2 mmol, 1.0 equiv) and 2-formylphenyl trifluoromethanesulfonate **5** (76.0 mg, 0.75 mmol, 1.5 equiv) and 1,4-dioxane (3.0 mL). The mixture was stirred at 130 ºC in an oil bath for 16 h. Afterwards, the reaction was cool down to room temperature, and the volatiles were removed in vacuo. The crude was purified by flash column chromatography on silica gel (5% EtOAc in hexanes with 5% Et_3N) to afford **7** as pale-yellow solid (37.0 mg, 0.188 mmol, 94% yield). In an independent experiment, 37.0 mg (94% yield) were obtained, giving an average yield of 94%. Deuterium incorporation was determined by ^{1}H NMR, based on comparison with non-deuterated C1–H position. M.P.:154 °C– 157 °C. ^{1}H NMR (400 MHz, $CDCl_3$) δ 8.82 (d, *J* = 8.0 Hz, 0.04H), 8.61 (ddt, *J* = 8.3, 1.4, 0.7 Hz, 1H), 7.76 – 7.62 (m, 2H), 7.49 (ddd, *J* = 8.2, 6.6, 1.5 Hz, 1H), 7.28 – 7.26 (m, 1H), 6.96 (ddd, *J* = 9.2, 6.2, 0.6 Hz, 1H), 6.84(s, 1H), 6.61 (dd, *J* = 6.3, 1.3 Hz, 1H).^{13}C NMR (126 MHz, $CDCl_3$) δ 159.4, 137.3, 136.5, 132.4, 128.3, 126.2, 126.0, 125.7, 125.1, 120.2, 111.9, 111.8, 101.1. IR (neat): 3050, 1656, 1612, 1570, 1155, 810, 667 cm^{-1}. HRMS (ESI) m/z: [M+H]$^+$ Calcd for $C_{13}H_9DNO$ 197.0825; Found 197.0822.

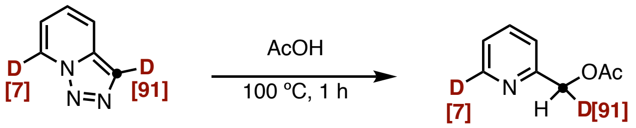

2a

8, 98%

(pyridin-2-yl-6-*d*)methyl acetate (8).[15] An 8 mL vial equipped with a stirring magnetic bar was charged with [1,2,3]triazolo[1,5-*a*]pyridine-7-*d* **2a** (24 mg, 0.2 mmol) and AcOH (1.0 mL). The resulting mixture was heated to 100 ºC in an oil bath for 1h. Afterwards, the reaction mixture was cooled down to room temperature, and a saturated aqueous solution of $NaHCO_3$ was added until reaching pH = 8. The resulting mixture was extracted with DCM (3 x 10 mL). The combination of organic layers was washed with brine, dried over Na_2SO_4, filtered, and concentrated in vacuo. The residue was purified by column chromatography on silica gel (30% EtOAc in hexanes) to afford **8** as yellow oil (30.0 mg, 0.197 mmol, 98% yield). In an independent experiment, 30.0 mg (98% yield) were obtained, giving an average yield of 98%. Deuterium incorporation was determined by ^{1}H NMR, based on comparison with non-deuterated C3–H position. ^{1}H NMR (400 MHz, $CDCl_3$) δ 8.64 – 8.58(m, 1H), 7.70 (td, *J* = 7.7, 1.8 Hz, 1H), 7.35 (d, *J* = 7.8 Hz, 1H), 7.27 – 7.21 (m, 1H), 5.24 – 5.18 (m, 1.09H), 2.16 (s, 3H). ^{13}C NMR (126 MHz, $CDCl_3$) δ 170.8, 155.8, 149.5, 136.9, 123.0, 122.0, 67.2– 66.2 (m), 21.0. IR (neat): 1736, 1594, 1372, 1229, 1043, 755 cm^{-1}. HRMS (ESI) m/z: [M+H]$^+$ Calcd for $C_8H_9DNO_2$ 153.0774; Found 153.0776.

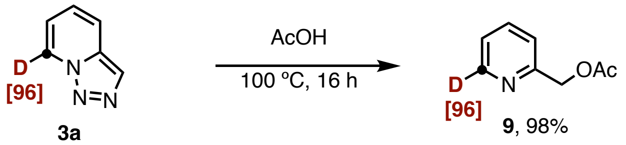

(pyridin-2-yl-6-*d*)methyl acetate (9).[15] An 8 mL vial equipped with a stirring magnetic bar was charged with [1,2,3]triazolo[1,5-a]pyridine-7-*d* **3a** (24 mg, 0.2 mmol) and AcOH (1.0 mL). The solution was heated to 100 ºC in an oil bath for 16 h. Afterwards, the reaction mixture was cooled down to room temperature, and a saturated aqueous solution of $NaHCO_3$ was added until reaching pH = 8. The resulting mixture was extracted with DCM (3 x 10 mL). The combination of organic layers was washed with brine, dried over Na_2SO_4, filtered, and concentrated in vacuo. The residue was purified by column chromatography on silica gel (30% EtOAc in hexanes with 5% Et_3N) to provide **9** as yellow oil (30.0 mg, 0.197 mmol, 98% yield). In an independent experiment, 30.0 mg (98% yield) were obtained, giving an average yield of 98%. Deuterium incorporation was determined by ^{1}H NMR, based on comparison with non-deuterated C3–H position. ^{1}H NMR (500 MHz, $CDCl_3$) δ 8.60 (d, J = 4.8 Hz, 0.04H), 7.70 (t, J = 7.7 Hz, 1H), 7.35 (d, J = 7.8 Hz, 1H), 7.23 (d, J = 7.6 Hz, 1H), 5.22 (s, 2H), 2.16 (s, 3H). ^{13}C NMR (126 MHz, $CDCl_3$) δ 170.8, 155.9, 149.4(t, J = 27.4 Hz), 136.9, 122.9, 122.0, 67.0, 21.1. IR (neat): 1742, 1589, 1447, 1374, 1219, 1042 cm^{-1}. HRMS (ESI) m/z: $[M+H]^+$ Calcd for $C_8H_9DNO_2$ 153.0774; Found 153.0763.

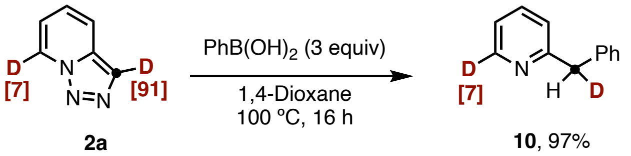

2-(phenylmethyl-*d*)pyridine-6-*d* (10).[21] An oven-dried Schlenk tube (10 mL) was charged with [1,2,3]triazolo[1,5-a]pyridine-7-*d* **2a** (24 mg, 0.2 mmol, 1.0 equiv), phenylboronic acid (73.2 mg, 0.6 mmol, 3.0 equiv) and 1,4-dioxane (1.0 mL). The mixture was stirred at 100 ºC in an oil bath for 16 h. Afterwards, the reaction was cooled down to room temperature and volatiles were removed in vacuo. The crude was purified by flash column chromatography on silica gel (5% EtOAc in hexanes with 5% Et_3N) to afford **10** as colorless oil (33.0 mg, 0.19 mmol, 97% yield). In an independent experiment, 33.0 mg (98% yield) were obtained, giving an average yield of 98%. Deuterium incorporation was determined by ^{1}H NMR, based on comparison with non-deuterated C3–H position. ^{1}H NMR (500 MHz, $CDCl_3$) δ 8.58 (dd, J = 5.3, 2.0 Hz, 0.93H), 7.59 (td, J = 7.7, 1.9 Hz, 1H), 7.35 – 7.28 (m, 4H), 7.27 – 7.22 (m, 1H), 7.16 – 7.11 (m, 2H), 4.20 – 4.16 (m, 1.01H). ^{13}C NMR (101 MHz, $CDCl_3$) δ 161.1, 149.5, 139.6, 136.6, 129.2,

128.7, 126.5, 123.2, 121.3, 45.0 – 43.9 (m). IR (neat): 3026, 1589, 1473, 1432, 738, 697, 607 cm^{-1}. HRMS (ESI) m/z: $[M+H]^+$ Calcd for $C_{12}H_{11}DN$ 171.1033; Found 171.1030.

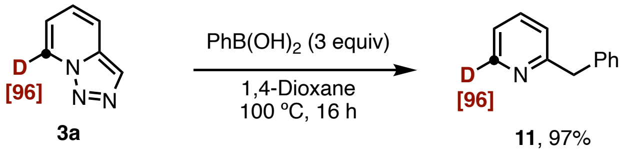

2-benzylpyridine-6-*d* (11).[21] An oven-dried Schlenk tube (10 mL) was charged with [1,2,3]triazolo[1,5-a]pyridine-7-*d* **3a** (24 mg, 0.2 mmol, 1.0), phenylboronic acid (73.2 mg, 0.6 mmol, 3.0 equiv) and 1,4-dioxane (1.0 mL). The mixture was stirred at 100 ºC in an oil bath for 16 h. Afterwards, the reaction was cooled down to room temperature and volatiles were removed in vacuo. The crude was purified by flash column chromatography on silica gel (5% EtOAc in hexanes with 5% Et_3N) to afford **11** as colorless oil (33.0 mg, 0.17 mmol, 97% yield). In an independent experiment, 33.0 mg (97% yield) were obtained, giving an average yield of 97%. Deuterium incorporation was determined by ^1H NMR, based on comparison with non-deuterated C3–H of pyridine position. ^1H NMR (500 MHz, $CDCl_3$) δ 8.62 – 8.53 (m, 1H), 7.60 (t, J = 7.7 Hz, 1H), 7.36 – 7.28 (m, 4H), 7.27 – 7.22 (m, 1H), 7.13 (d, J = 7.7 Hz, 2H), 4.19 (s, 2H). ^{13}C NMR (101 MHz, $CDCl_3$) ^{13}C NMR (101 MHz, $CDCl_3$) δ 161.1, 149.1, 139.6, 136.6, 129.2(2C), 128.7(2C), 126.5, 123.2, 121.2, 44.8. IR (neat): 3027, 1582, 1440, 740, 695, 598 cm^{-1}. HRMS (ESI) m/z: $[M+H]^+$ Calcd for $C_{12}H_{11}DN$ 171.1033; Found 171.1030.

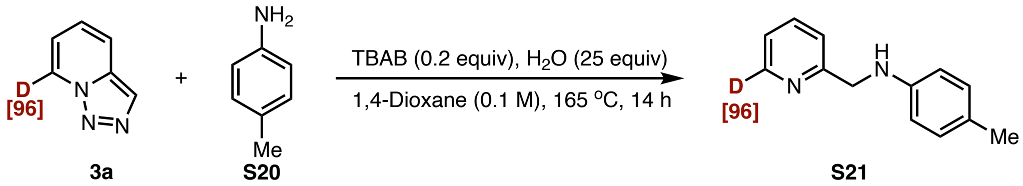

4-methyl-*N*-((pyridin-2-yl-6-*d*)methyl)aniline (S21).[23] An oven-dried Schlenk tube equipped with a stirring magnetic bar was charged with [1,2,3]triazolo[1,5-a]pyridine-7-*d* **3a** (24 mg, 0.2 mmol, 1.0 equiv), *p*-toluidine **S20** (49.3 mg, 0.4 mmol, 2.0 equiv), Tetrabutylammonium bromide (TBAB) (12.9 mg, 0.04 mmol, 0.2 equiv), H_2O (90.0 µL, 5.0 mmol, 25.0 equiv) and 1,4-Dioxane (2.0 mL). The mixture was stirred at 165 ºC in an oil bath for 14 h. Afterwards, the reaction was cooled down to rt and the volatiles were removed under reduced pressure. The residue was purified by flash column chromatography on silica gel (20% EtOAc in hexanes with 5% Et_3N) to afford **S21** as brown oil (44.0 mg, 0.18 mmol, 90% yield). In an independent experiment, 44.0 mg (90% yield) were obtained, giving an average yield of 90%. Deuterium incorporation was

determined by ^{1}H NMR, based on comparison with non-deuterated C3–H position. ^{1}H NMR (400 MHz, $CDCl_3$) δ 8.58 (d, J = 5.0 Hz, 0.04H), 7.63 (t, J = 7.7 Hz, 1H), 7.36 – 7.32 (m, J = 7.8, 1.2, 0.6 Hz, 1H), 7.17 (d, J = 7.5 Hz, 1H), 7.05 – 6.94 (m, 2H), 6.62 – 6.57 (m, 2H), 4.63 (s, 1H), 4.45 (s, 2H), 2.24 (s, 3H). ^{13}C NMR (126 MHz, $CDCl_3$) δ 158.9, 149.7 – 148.5 (m), 145.8, 136.7, 129.9, 126.9(2C), 122.0, 121.7, 113.3(2C), 49.8, 20.5. IR (neat): 3385, 3017, 2918, 1616, 1585, 1519, 1442, 1315, 1266, 806 cm^{-1}. HRMS (ESI) m/z: $[M+H]^+$ Calcd for $C_{13}H_{14}DN_2$ 200,1298; Found 200.1287.

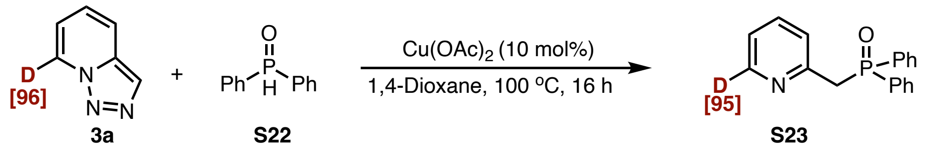

Diphenyl((pyridin-2-yl-6-*d*)methyl)phosphine oxide (S23).[24] An oven-dried Schlenk tube equipped with a stirring magnetic bar was charged with $Cu(OAc)_2$ (3.6 mg, 0.02 mmol,), [1,2,3]triazolo[1,5-a]pyridine-7-*d* **3a** (24 mg, 0.2 mmol, 1.0 equiv), diphenylphosphine oxide **S22** (60.6 mg, 0.3 mmol, 1.5 equiv) and 1,4-dioxane (1.0 mL) under argon atmosphere. The mixture was stirred at 100 ºC in an oil bath for 6 h. Afterwards, it was cooled to room temperature and diluted with saturated EDTA-4Na solution in water (5 mL). The mixture was extracted with EtOAc (10 mL×3), and the combination of organic layers was dried with $MgSO_4$, filtrated and concentrated in vacuo. The residue was purified by column chromatography on silica gel (80% EtOAc in hexanes with 5% Et_3N) to afford **S23** as white solid (48.0 mg, 0.19 mmol, 95% yield). In an independent experiment, 48.2 mg (95% yield) were obtained, giving an average yield of 95%. Deuterium incorporation was determined by ^{1}H NMR, based on comparison with non-deuterated C3–H position. M.P.:128 ℃– 132℃. ^{1}H NMR (500 MHz, $CDCl_3$) δ 8.40 – 8.37 (m, 0.05H), 7.79 – 7.71 (m, 4.0H), 7.56 (t, J = 7.6 Hz, 1.0H), 7.52 – 7.46 (m, 3.0H), 7.46 – 7.41 (m, 4.0H), 7.08 (d, J = 7.5 Hz, 1.0H), 3.93 (d, J = 14.2 Hz, 2.0H). ^{13}C NMR (126 MHz, $CDCl_3$) δ 152.6 (d, J = 7.0 Hz), 149.6 – 148.5 (m), 136.5 (d, J = 2.2 Hz), 132.9, 132.1, 131.9 (d, J = 2.8 Hz)(2C), 131.2 (d, J = 9.5 Hz)(4C), 128.6 (d, J = 11.9 Hz)(4C), 125.1 (d, J = 3.6 Hz), 121.8 (d, J = 2.4 Hz), 41.0 (d, J = 64.5 Hz). ^{31}P NMR (202 MHz, $CDCl_3$) δ 32.84 – 32.46 (m). IR (neat): 3054, 2948, 2904, 1578, 1437, 1403, 1183, 1118, 833, 737, 718, 693 cm^{-1}. HRMS (ESI) m/z: $[M+H]^+$ Calcd for $C_{18}H_{16}DNOP$ 295,1111; Found 295,1105.

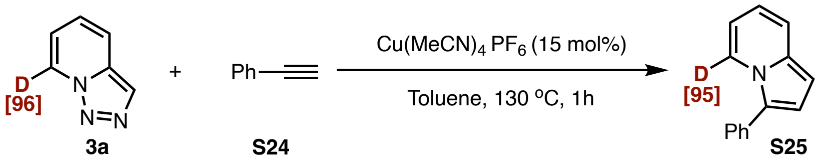

3-Phenylindolizine-5-*d* (S25). [25] An oven dried Schlenk tube equipped with a stirring magnetic bar was charged with [1,2,3]triazolo[1,5-*a*]pyridine-7-*d* **3a** (24 mg, 0.2 mmol, 1.0 equiv), ethynylbenzene **S24** (26.3 µL, 0.24 mmol, 1.2 equiv), $Cu(MeCN)_4PF_6$ (11.2 mg, 0.03 mmol, 15 mol %) and toluene (0.5 mL). The reaction mixture was stirred at 130 ºC in an oil bath for 12 h. Afterwards, the reaction was cooled down to rt and the solvent was removed under reduced pressure. The residue was purified by flash column chromatography on silica gel (20% EtOAc in hexanes with 5% Et_3N) to afford the **S25** as black solid (20.0 mg, 0.18 mmol, 52% yield). In an independent experiment, 20.0 mg (52% yield) were obtained, giving an average yield of 52%. Deuterium incorporation was determined by ^{1}H NMR, based on comparison with non-deuterated C8–H position. ^{1}H NMR (500 MHz, $CDCl_3$) δ 8.30 – 8.27 (m, 0.05H), 7.60 – 7.56 (m, 2H), 7.51 – 7.46 (m, 2H), 7.42 (dd, *J* = 9.0, 1.3 Hz, 1H), 7.38 – 7.33 (m, 1H), 6.87 (d, *J* = 3.9 Hz, 1H), 6.68 (dd, *J* = 9.0, 6.4 Hz, 1H), 6.54 (d, *J* = 4.0 Hz, 1H), 6.48 (dd, *J* = 6.4, 1.4 Hz, 1H). ^{13}C NMR (126 MHz, $CDCl_3$) δ 134.0, 132.8, 129.1(2C), 128.2(2C), 127.2, 125.5, 122.2, 119.8, 117.0, 114.2, 110.6, 99.9. IR (neat): 3256, 3061, 3026, 1599, 1506, 1473, 1352, 1295, 744, 696 cm^{-1}. HRMS (ESI) m/z: $[M+H]^+$ Calcd for $C_{14}H_{11}DN$ 195,1033; Found 195,1018.

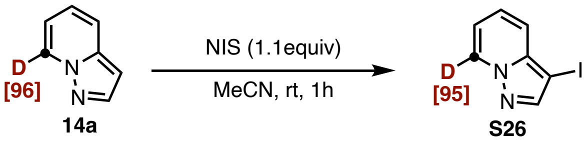

3-iodopyrazolo[1,5-*a*]pyridine-7-*d* (S26). [26] An oven-dried 8 mL vial equipped with a stirring magnetic bar was charged with pyrazolo[1,5-*a*]pyridine-7-*d* **14a** (24 mg, 0.2 mmol, 1.0 equiv.), N-Iodosuccinimide (NIS) (49.0 mg, 0.22 mmol, 1.1 equiv) and MeCN (0.4 mL). The mixture was stirred at room temperature for 1h. The reaction was quenched with H_2O, and the mixture was extracted with EtOAc (2x 10 mL). The combination of the organic layers was washed with brine, dried over Na_2SO_4, filtered and concentrated in vacuo. The residue was purified by flash column chromatography on silica gel (30% EtOAc in hexanes) to afford **S26** as red solid (44.0 mg, 0.18 mmol, 90% yield). In an independent experiment, 44.0 mg (90% yield) were obtained, giving an average yield of 90%. Deuterium incorporation was determined by ^{1}H NMR, based on comparison with non-deuterated C4–H position. M.P.: 62 °C – 64 °C. ^{1}H NMR (400 MHz, $CDCl_3$) δ 8.45 (d, *J* = 7.1 Hz, 0.04H), 7.96 (s, 1H), 7.50 – 7.45 (m, 1H), 7.24 – 7.16 (m, 1H), 6.80 (d, *J*

= 6.7 Hz, 1H). ^{13}C NMR (126 MHz, $CDCl_3$) δ 146.4, 141.1, 129.1, 124.8, 118.0, 112.7, 112.5. IR (neat): 3098, 1620, 1500, 1442, 1308, 988, 879, 785 cm^{-1}. HRMS (ESI) m/z: [M+H]$^+$ Calcd for $C_7H_5DIN_2$ 245.9638; Found 245.9633.

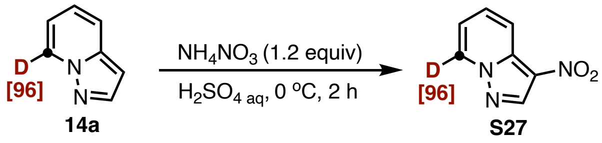

3-nitropyrazolo[1,5-*a*]pyridine-7-*d* (S27).[27] An oven-dried 10 mL round bottom flask equipped with a stirring magnetic bar was charged with pyrazolo[1,5-*a*]pyridine-7-*d* **14a** (24 mg, 0.2 mmol, 1.0 equiv) in concentrated sulfuric acid (0.2 mL) and NH_4NO_3 (19.2 mg, 0.24 mmol, 1.2 equiv) in concentrated sulfuric acid (0.3 mL) was added dropwise at -5 ºC. The mixture was stirred at 0 ºC for 2 h and added dropwise to a solution of 4 N sodium hydroxide (2.0 mL) at 0 ºC, followed by the addition of sodium bicarbonate (0.6 g). The resulting mixture was extracted with EtOAc (3 x 10 mL). The combination of the organic layers was washed with brine, dried over Na_2SO_4, filtered and concentrated in vacuo. The residue was purified by flash column chromatography on silica gel (30% EtOAc in hexanes) to afford **S27** as green solid (25.9 mg, 0.158 mmol, 79% yield). In an independent experiment, 26.0 mg (79% yield) were obtained, giving an average yield of 79%. Deuterium incorporation was determined by ^{1}H NMR, based on comparison with non-deuterated C4–H position. M.P.:80 °C– 83 °C. ^{1}H NMR (500 MHz, $CDCl_3$) δ 8.62 (s, 1H), 8.58 (dt, J = 6.9, 1.1 Hz, 0.04 H), 8.36 (dd, J = 8.9, 1.4 Hz, 1H), 7.68 (dd, J = 8.9, 7.0 Hz, 1H), 7.16 (dd, J = 7.0, 1.3 Hz, 1H). ^{13}C NMR (126 MHz, CDC13) δ 140.9, 136.5, 131.3, 130.6 – 129.9 (m), 118.9, 116.1, 115.9. IR (neat): 3123, 1627, 1500, 1457, 1400, 1281, 1175, 1134, 796, 740 cm^{-1}. HRMS (ESI) m/z: [M+H]$^+$ Calcd for $C_7H_5DN_3O_2$ 165.0523; Found 165.0520.

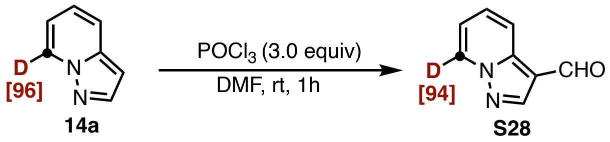

pyrazolo[1,5-*a*]pyridine-7-*d*-3-carbaldehyde S28). [28] An oven-dried round bottom flask was charged with pyrazolo[1,5-*a*]pyridine-7-*d* **14a** (24 mg, 0.2 mmol, 1.0 equiv.), $POCl_3$ (56.0 µL, 0.6 mmol, 3.0 equiv.) and DMF (0.24 mL). The mixture was stirred at room temperature for 1h. The resulting mixture was extracted with EtOAc and washed with 2N aqueous solution of sodium hydroxide. The organic extracts were combined,

washed with brine, dried over Na_2SO_4, filtered and concentrated. The residue was purified by column chromatography on silica gel (30% EtOAc in hexanes) to afford **15** as green solid (44.0 mg, 0.18 mmol, 75% yield). In an independent experiment, 44.0 mg (75% yield) were obtained, giving an average yield of 75%. Deuterium incorporation was determined by ^{1}H NMR, based on comparison with non-deuterated C4–H position. M.P.:84 °C– 86 °C. ^{1}H NMR (400 MHz, $CDCl_3$) δ 10.05 (s, 1H), 8.59 (d, *J* = 7.0 Hz, 0.06H), 8.39 (s, 1H), 8.30 (dd, *J* = 8.8, 1.4 Hz, 1H), 7.54 (dd, *J* = 8.8, 6.9 Hz, 1H), 7.08 (d, *J* = 6.9 Hz, 1H). ^{13}C NMR (126 MHz, $CDCl_3$) δ 183.5, 146.7, 139.9, 129.3(2C), 119.5, 115.2, 113.9. IR (neat): 3093, 2842, 1659, 1625, 1512, 1370, 1167, 857, 787, 681 cm^{-1}. HRMS (ESI) m/z: $[M+H]^+$ Calcd for $C_8H_6DN_2O$ 148.0621; Found 148.0622.

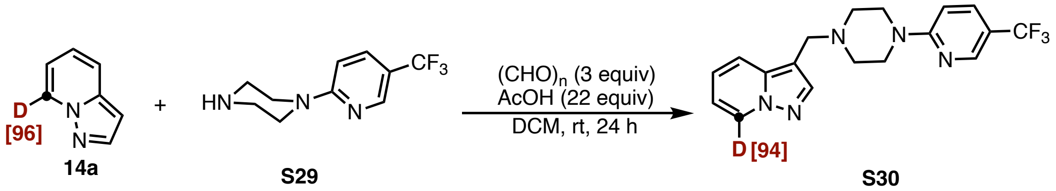

3-((4-(5-(trifluoromethyl)pyridin-2-yl)piperazin-1-yl)methyl)pyrazolo[1,5 *a*]pyridine-7-*d* (S30).[29] An oven-dried round bottom flask equipped with a stirring magnetic bar was charged with pyrazolo[1,5-*a*]pyridine-7-*d* **6a** (24 mg, 0.2 mmol, 1.0 equiv), 1-[5-(trifluoromethyl)pyridin-2-yl]piperazine **S29** (46.2 mg, 0.2 mmol, 1.0 equiv), $(CHO)_n$ (37% wt in H_2O) (50.0 μL, 0.6 mmol, 3 equiv), AcOH (0.26 ml, 4.4 mmol, 22 equiv) and DCM (0.04 M). The mixture was stirred at room temperature overnight. The resulting mixture was extracted with EtOAc (3 x 20 mL) and the combination of the organic layers was washed with brine, dried over Na_2SO_4, filtered and concentrated in vacuo. The residue was purified by flash column chromatography on silica gel (50% EtOAc in hexanes with 1% Et_3N) to afford **S30** as white solid (50.7 mg, 0.14 mmol, 70% yield). In an independent experiment, 50.6 mg (70% yield) were obtained, giving an average yield of 70%. Deuterium incorporation was determined by ^{1}H NMR, based on comparison with non-deuterated C4–H position. M.P.:103 °C– 106 °C. ^{1}H NMR (500 MHz, $CDCl_3$) δ 8.44 (d, *J* = 7.0 Hz, 0.05H), 8.37 (dt, *J* = 2.7, 0.9 Hz, 1H), 7.90 (s, 1H), 7.64 –7.57 (m, 2H), 7.10 (dd, *J* = 8.9, 6.7 Hz, 1H), 6.74 (dd, *J* = 6.6, 1.4 Hz, 1H), 6.59 (d, *J* = 9.0 Hz, 1H), 3.74 (s, 2H), 3.69 – 3.59 (m, 4H), 2.62 – 2.41 (m, 4H). ^{13}C NMR (126 MHz, $CDCl_3$) δ 160.5, 145.8 (m), 142.5, 139.3, 135.2 – 133.8 (m), 129.0 – 128.2 (m), 124.7 (q, *J* = 270.2 Hz), 123.1, 117.3, 115.2 (q, *J* = 33.0 Hz), 111.7, 106.6, 105.6, 52.6(2C), 52.2, 44.8(2C). IR (neat): 2925, 2783, 1607, 1505, 1314, 1253, 1077, 997, 813,

786 cm^{-1}. HRMS (ESI) m/z: $[M+Na]^+$ Calcd for $C_{18}H_{17}DF_3N_5Na$ 385.1475; Found 385.1484.

Selective C3-Deuteration of pyrazolo[1,5-*a*]pyridine

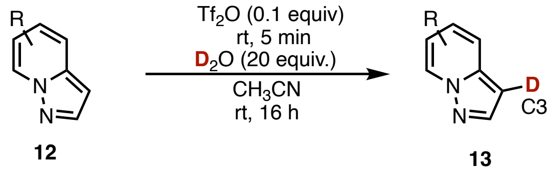

General procedure 3 (**GP3**). An oven-dried 8 mL vial equipped with a stirring magnetic bar was charged with pyrazolo[1,5-*a*]pyridine **12** (0.2 mmol, 1.0 equiv). The vial was evacuated and backfilled with argon for at least three times and CH_3CN (1 mL, 0.2 M) were added. TFA (1.5 µL, 0.02 mmol, 0.1 equiv) and D_2O (72 µL, 4.0 mmol, 20 equiv) were added at room temperature and the reaction was stirred for 16 hours. The reaction mixture was quenched by Et_3N (10 µL), diluted with EtOAc, filtered through a silica plug and the concentrated in vacuo. The residue was purified by flash column chromatography on silica gel to provide the desired product.

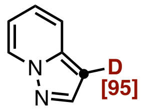

pyrazolo[1,5-a]pyridine-3-*d* (13a). Following **GP3**, utilizing pyrazolo[1,5-*a*]pyridine **12a** (24.0 µL, 0.20 mmol, 1.0 equiv), TFA (1.5 µL, 0.02 mmol, 0.1 equiv), D_2O (72 µL, 4.0 mmol, 20 equiv) and CH_3CN (1 mL). Flash column chromatography on silica gel (50% EtOAc in hexanes with 1% Et_3N) afforded **13a** as black oil (26.3 mg, 0.196 mmol, 96% yield, 95% D, C3:C7 = 99:1). In an independent experiment, 26.2 mg (96% yield, 95% D, C3:C7 = 99:1) were obtained, giving an average yield of 96%. Deuterium incorporation was determined by ^1H NMR, based on comparison with non-deuterated C4–H position. ^1H NMR (300 MHz, $CDCl_3$) δ 8.48 (dt, *J* = 7.0, 1.1 Hz, 1H), 7.95 (s, 1H), 7.54 (dt, *J* = 8.9, 1.2 Hz, 1H), 7.09 (ddd, *J* = 8.9, 6.7, 1.1 Hz, 1H), 6.74 (td, *J* = 6.9, 1.4 Hz, 1H), 6.51 (d, *J* = 1.9 Hz, 0.05H).^{13}C NMR (126 MHz, $CDCl_3$) δ 141.81, 140.16, 128.74, 123.23, 118.21, 111.69, 96.63(t, *J* = 27.2 Hz). IR (neat): 2925, 1632, 1510, 1335, 1240, 746, 732cm^{-1}. HRMS (ESI) m/z: $[M+H]^+$ Calcd for $C_7H_6DN_2$ 120.0672; Found 120.0672.

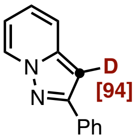

2-phenylpyrazolo[1,5-*a*]pyridine-3-*d* (13b). Following **GP3**, utilizing 2-phenylpyrazolo[1,5-a]pyridine **12b** (38.8 mg, 0.20 mmol, 1.0 equiv), D_2O (72 µL, 4.0 mmol, 20 equiv) and CH_3CN (1 mL).. Flash column chromatography on silica gel (50% EtOAc in hexanes with 1% Et_3N) afforded **13b** as white solid (37.0 mg, 0.19 mmol, 95% yield, 94% D, C3:C7 = 99:1). In an independent experiment, 37.0 mg (95% yield, 94% D, C3:C7 = 99:1) were obtained, giving an average yield of 95%. Deuterium incorporation was determined by ^{1}H NMR, based on comparison with non-deuterated C4–H position. M.P.:104 °C– 107 °C. ^{1}H NMR (300 MHz, $CDCl_3$) δ 8.46 (s, 1H), 7.98 (s, 2H), 7.61 – 7.30 (m, 3H), 7.09 (s, 1H), 6.80 (s, 0.04H), 6.74 (s, 1H). ^{13}C NMR (75 MHz, $CDCl_3$) δ 153.61, 141.7, 133.4, 128.9(2C), 128.6, 128.5, 126.6(2C), 123.5, 118.0, 111.8, 93.8. IR (neat): 3033, 1629, 1455, 1329, 747,732, 685cm^{-1}. HRMS (ESI) m/z: $[M+H]^+$ Calcd for $C_{13}H_{10}DN_2$ 196.0985; Found 196.0981.

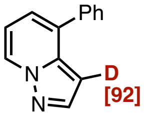

4-phenylpyrazolo[1,5-*a*]pyridine-3-*d* (13c). Following **GP3**, utilizing 2-phenylpyrazolo[1,5-a]pyridine **12c** (38.8 mg, 0.20 mmol, 1.0 equiv), D_2O (72 µL, 4.0 mmol, 20 equiv) and CH_3CN (1 mL).. Flash column chromatography on silica gel (50% EtOAc in hexanes with 1% Et_3N) afforded **13c** as white solid (37.0 mg, 0.19 mmol, 95% yield, 92% D, C3:C7 = 99:1). In an independent experiment, 37.0 mg (95% yield, 92% D, C3:C7 = 99:1) were obtained, giving an average yield of 95%. Deuterium incorporation was determined by ^{1}H NMR, based on comparison with non-deuterated C5–H position. M.P.:82 °C– 84 °C. ^{1}H NMR (300 MHz, $CDCl_3$) δ 8.48 (dd, J = 7.0, 1.0 Hz, 1H), 7.99 (s, 1H), 7.71 – 7.64 (m, 2H), 7.57 – 7.39 (m, 3H), 7.14 (dd, J = 7.0, 1.0 Hz, 1H), 6.85 (t, J = 7.0 Hz, 1H), 6.69 – 6.65 (m, 0.08H).^{13}C NMR (75 MHz, $CDCl_3$) δ 142.0, 139.8, 138.2, 132.4, 128.9 (2C), 128.4, 128.1(2C), 127.7, 122.2, 112.0, 97.2. IR (neat): 3085, 3054, 1620, 1412, 1355, 797, 761, 702 cm^{-1}. HRMS (ESI) m/z: $[M+H]^+$ Calcd for $C_{13}H_{10}DN_2$ 196.0985; Found 196.0977.

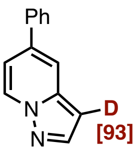

5-phenylpyrazolo[1,5-*a*]pyridine-3-*d* (13d). Following **GP3**, utilizing 5-phenylpyrazolo[1,5-a]pyridine **12d** (38.8 mg, 0.20 mmol, 1.0 equiv), D_2O (72 µL, 4.0 mmol, 20 equiv) and CH_3CN (1 mL).. Flash column chromatography on silica gel (50% EtOAc in hexanes with 1% Et_3N) afforded **13d** as white solid (37.9 mg, 0.194 mmol, 97% yield, 93% D, C3:C7 = 99:1). In an independent experiment, 38.0 mg (97% yield, 93% D, C3:C7 = 99:1) were obtained, giving an average yield of 97%. Deuterium incorporation was determined by ^{1}H NMR, based on comparison with non-deuterated C4–H position. M.P.: 98 °C– 100 °C. ^{1}H NMR (300 MHz, $CDCl_3$) δ 8.52 (dd, *J* = 7.3, 0.9 Hz, 1H), 7.97 (s, 1H), 7.73 (dd, *J* = 2.0, 1.0 Hz, 1H), 7.69 – 7.60 (m, 2H), 7.53 – 7.35 (m, 3H), 7.07 – 6.99 (m, 1H), 6.57 – 6.54 (m, 0.07H). ^{13}C NMR (75 MHz, $CDCl_3$) δ 142.5, 140.3, 138.9, 136.4, 129.1 (2C), 128.6, 128.2, 126.9(2C), 115.1, 111.7, 97.4 (t, *J* = 13.8 Hz). IR (neat): 3078, 2346, 1632, 1522, 1427, 1212, 871, 759, 687 cm^{-1}. HRMS (ESI) m/z: $[M+H]^+$ Calcd for $C_{13}H_{10}DN_2$ 196.0985; Found 196.0988.

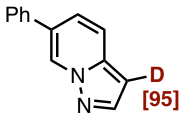

6-phenylpyrazolo[1,5-*a*]pyridine-3-*d* (13e). Following **GP3**, utilizing 6-phenylpyrazolo[1,5-a]pyridine **13e** (38.8 mg, 0.20 mmol, 1.0 equiv), D_2O (72 µL, 4.0 mmol, 20 equiv) and CH_3CN (1 mL). Flash column chromatography (silica gel: 50% EtOAc in hexanes with 1% Et_3N) afforded **13e** as white solid (35.9 mg, 0.184 mmol, 92% yield, 95% D, C3:C7 = 99:1). In an independent experiment, 35.9 mg (92% yield, 95% D, C3:C7 = 99:1) were obtained, giving an average yield of 92%. Deuterium incorporation was determined by ^{1}H NMR, based on comparison with non-deuterated C4–H position. M.P.:57 °C– 59 °C. ^{1}H NMR (300 MHz, $CDCl_3$) δ 8.70 (dd, *J* = 1.6, 1.0 Hz, 1H), 7.98 (s, 1H), 7.66 – 7.58 (m, 3H), 7.54 – 7.45 (m, 2H), 7.43 – 7.36 (m, 2H), 6.54 (d, *J* = 2.2 Hz, 0.07H). ^{13}C NMR (75 MHz, $CDCl_3$) δ 142.3, 139.2, 137.4, 129.2 (2C), 127.9, 126.9(2C), 126.2, 126.1, 123.9, 118.0, 96.8 (t, *J* = 14.0 Hz). IR (neat): 3032, 1636, 1488, 1432, 1417, 1322, 755cm^{-1}. HRMS (ESI) m/z: $[M+H]^+$ Calcd for $C_{13}H_{10}DN_2$ 196.0985; Found 196.0984.

Selective C7-Deuteration of pyrazolo[1,5-a]pyridine

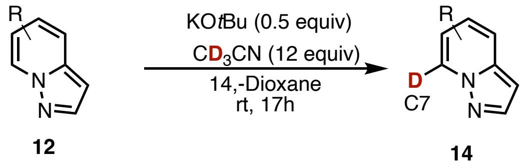

General Procedure 4 (**GP4**): An oven-dried 8 mL vial equipped with a stirring magnetic bar was charged with pyrazolo[1,5-a]pyridine **12a** (1.0 equiv). The vial was put into a nitrogen filled glovebox and KO*t*-Bu (11.2 mg, 0.1 mmol, 0.5 equiv) was added. The vial was taken out from the glovebox and, 1,4-dioxane (1 mL, 0.2 M) followed by CD_3CN (125 µL, 2.4 mmol, 12 equiv) were added at room temperature and the reaction was stirred for 17 hours. The reaction mixture was diluted with EtOAc, filtered through a silica plug and concentrated in vacuo. The crude was purified by flash column chromatography on silica gel to provide the desired product.

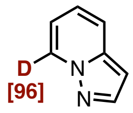

pyrazolo[1,5-*a*]pyridine-7-*d* (14a). Following **GP4**, utilizing 2-phenylpyrazolo[1,5-*a*]pyridine **12a** (24.0 µL, 0.20 mmol, 1.0 equiv), KO*t*-Bu (11.2 mg, 0.1 mmol, 0.5 equiv), CD_3CN (125 µL, 2.4 mmol, 12 equiv) and 1,4-dioxane (1 mL). Flash column chromatography on silica gel (5% EtOAc in hexanes with 1% Et_3N) afforded **14a** as black oil (26.3 mg, 0.196 mmol, 96% yield, 96% D, C7:C3 = 99:1). In an independent experiment, 26.2 mg (96% yield, 96% D, C7:C3 = 99:1) were obtained, giving an average yield of 96%. Deuterium incorporation was determined by ^{1}H NMR, based on comparison with non-deuterated C4–H position. ^{1}H NMR (500 MHz, $CDCl_3$) δ 8.48 (d, J = 7.0 Hz, 0.04 H), 7.95 (d, J = 2.3 Hz, 1H), 7.54 (dd, J = 8.9, 1.4 Hz, 1H), 7.10 (dd, J = 8.9, 6.7 Hz, 1H), 6.74 (d, J = 6.7 Hz, 1H), 6.51 (d, J = 2.3 Hz, 1H). ^{13}C NMR (75 MHz, $CDCl_3$) δ 141.8, 140.2, 128.4, 123.2, 118.2, 111.5, 96.8. IR (neat): 3032, 1636, 1488, 1432, 1417, 1322, 755cm^{-1}. HRMS (ESI) m/z: $[M+H]^+$ Calcd for $C_7H_6DN_2$ 120.0672; Found 120.0667.

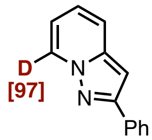

2-phenylpyrazolo[1,5-*a*]pyridine-7-*d* (14b). Following **GP4**, utilizing 2-phenylpyrazolo[1,5-*a*]pyridine **12b** (38.8 mg, 0.20 mmol, 1.0 equiv), KO*t*-Bu (11.2 mg, 0.1 mmol, 0.5 equiv), CD_3CN (125 µL, 2.4 mmol, 12 equiv) and 1,4-dioxane (1 mL). Flash column chromatography on silica gel (5% EtOAc in hexanes with 1% Et_3N)

afforded **14b** as white solid (37.9 mg, 0.194 mmol, 97% yield, 97% D, C7:C3 = 99:1). In an independent experiment, 37.9 mg (97% yield, 97% D, C7:C3 = 99:1) were obtained, giving an average yield of 97%. Deuterium incorporation was determined by ^{1}H NMR, based on comparison with non-deuterated C4–H position. M.P.:103 °C– 105 °C. ^{1}H NMR (500 MHz, $CDCl_3$) δ 8.50 – 8.45 (m, 0.03H), 7.99 – 7.93 (m, 2H), 7.52 (dd, *J* = 8.9, 1.4 Hz, 1H), 7.48 – 7.43 (m, 2H), 7.41 – 7.34 (m, 1H), 7.09 (dd, *J* = 8.9, 6.7 Hz, 1H), 6.80 (s, 1H), 6.73 (dd, *J* = 6.7, 1.3 Hz, 1H); ^{13}C NMR (75 MHz, $CDCl_3$) δ 153.7, 141.8, 133.4, 128.8 (2C), 128.7, 128.5, 126.6 (2C), 123.5, 118.0, 111.6, 93.8. IR (neat): 3123, 2267, 1623, 1500, 1463, 1307, 797, 725, 683 cm^{-1}. HRMS (ESI) m/z: $[M+H]^+$ Calcd for $C_{13}H_{10}DN_2$ 196.0985; Found 196.0987.

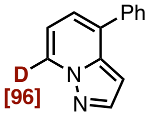

4-phenylpyrazolo[1,5-*a*]pyridine-7-*d* (14c). Following **GP4**, utilizing 4-phenylpyrazolo[1,5-a]pyridine **12c** (38.8 mg, 0.20 mmol, 1.0 equiv), KO*t*-Bu (11.2 mg, 0.1 mmol, 0.5 equiv), CD_3CN (125 μL, 2.4 mmol, 12 equiv) and 1,4-dioxane (1 mL). Flash column chromatography on silica gel (5% EtOAc in hexanes with 1% Et_3N) afforded **14c** as white solid (37.0 mg, 0.19 mmol, 95% yield, 96% D, C7:C3 = 99:1). In an independent experiment, 37.0 mg (95% yield, 96% D, C7:C3 = 99:1) were obtained, giving an average yield of 95%. Deuterium incorporation was determined by ^{1}H NMR, based on comparison with non-deuterated C5–H position. M.P.:57 °C– 59 °C. ^{1}H NMR (500 MHz, $CDCl_3$) δ 8.48 (d, *J* = 7.0 Hz, 0.04H), 7.99 (d, *J* = 2.3 Hz, 1H), 7.69 – 7.64 (m, 2H), 7.54 – 7.47 (m, 2H), 7.46 – 7.39 (m, 1H), 7.14 (d, *J* = 6.9 Hz, 1H), 6.85 (d, *J* = 7.0 Hz, 1H), 6.67 (d, *J* = 2.3 Hz, 1H). ^{13}C NMR (75 MHz, CDC13) δ 142.1, 139.8, 138.2, 132.4, 128.9 (2C), 128.4, 128.1(2C), 127.4, 122.3, 111.8, 97.1. IR (neat): 3030, 2299, 1607, 1449, 1178, 774, 735, 701 cm^{-1}. HRMS (ESI) m/z: $[M+H]^+$ Calcd for $C_{13}H_{10}DN_2$ 196.0985; Found 196.0984.

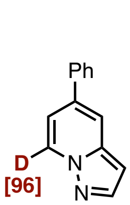

5-phenylpyrazolo[1,5-a]pyridine-7-*d* (14d). Following **GP4**, utilizing 5-phenylpyrazolo[1,5-a]pyridine **12d** (38.8 mg, 0.20 mmol, 1.0 equiv), KO*t*-Bu (11.2 mg, 0.1 mmol, 0.5 equiv), CD_3CN (125 μL, 2.4 mmol, 12 equiv) and 1,4-dioxane (1 mL).

Flash column chromatography on silica gel (5% EtOAc in hexanes with 1% Et_3N) afforded **14d** as white solid (37.9 mg, 0.194 mmol, 97% yield, 96% D, C7:C3 = 99:1). In an independent experiment, 38.0 mg (97% yield, 96% D, C7:C3 = 99:1) were obtained, giving an average yield of 97%. Deuterium incorporation was determined by ^1H NMR, based on comparison with non-deuterated C4–H position. M.P.: 100 °C– 102 °C. ^1H NMR (300 MHz, $CDCl_3$) δ 8.55 – 8.48 (m, 0.04H), 7.97 (d, J = 2.3 Hz, 1H), 7.73 (d, J = 2.0 Hz, 1H), 7.69 – 7.61 (m, 2H), 7.54 – 7.44 (m, 2H), 7.44 – 7.37 (m, 1H), 7.03 (d, J = 2.0 Hz, 1H), 6.56 (d, J = 2.3 Hz, 1H). ^{13}C NMR (75 MHz, $CDCl_3$) δ 142.6, 140.4, 138.9, 136.4, 129.1 (2C), 128.4, 128.3, 126.9(2C), 115.1, 111.5, 97.3. IR (neat): 3132, 2299, 1626, 1512, 1423, 862, 774, 682 cm^{-1}. HRMS (ESI) m/z: $[M+H]^+$ Calcd for $C_{13}H_{10}DN_2$ 196.0985; Found 196.0980.

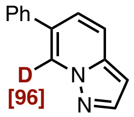

6-phenylpyrazolo[1,5-*a*]pyridine-7-*d* (14e). Following **GP4**, utilizing 6-phenylpyrazolo[1,5-a]pyridine **12e** (38.8 mg, 0.20 mmol, 1.0 equiv), KO*t*-Bu (11.2 mg, 0.1 mmol, 0.5 equiv), CD_3CN (125 μL, 2.4 mmol, 12 equiv) and 1,4-dioxane (1 mL). Flash column chromatography on silica gel (5% EtOAc in hexanes with 1% Et_3N) afforded **14e** as white solid (37.5 mg, 0.192 mmol, 96% yield, 96% D, C7:C3 = 99:1). In an independent experiment, 38.0 mg (96% yield, 96% D, C7:C3 = 99:1) were obtained, giving an average yield of 96%. Deuterium incorporation was determined by ^1H NMR, based on comparison with non-deuterated C4–H position. M.P.:57 °C– 59 °C. ^1H NMR (500 MHz, $CDCl_3$) δ 8.70 (d, J = 1.6 Hz, 0.04H), 7.98 (d, J = 2.3 Hz, 1H), 7.62 – 7.56 (m, 3H), 7.48 (t, J = 7.7 Hz, 2H), 7.41 – 7.37 (m, 2H), 6.54 (d, J = 2.3 Hz, 1H). ^{13}C NMR (75 MHz, $CDCl_3$) δ 142.4, 139.2, 137.3, 129.2 (2C), 127.9, 126.9(2C), 126.1, 125.8, 123.9, 118.0, 96.8. IR (neat): 3134, 3027, 1626, 1485, 1430, 813, 737, 696 cm^{-1}. HRMS (ESI) m/z: $[M+H]^+$ Calcd for $C_{13}H_{10}DN_2$ 196.0985; Found 196.0980.

Mechanistic Investigations

Kinetic Isotope Effect (KIE)

Kinetic profile for deuteration of 1a at 25 °C

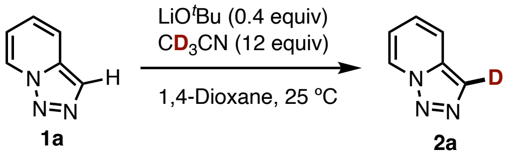

An oven-dried 8 mL vial equipped with a stirring magnetic bar was charged with **1a** (47.6 mg, 0.4 mmol, 1.0 equiv) and brought it into a nitrogen-filled glovebox. LiO*t*-Bu (12.8 mg, 0.16 mmol, 0.4 equiv) was added and the vial was taken-out from the glovebox. 1,4-dioxane (2 mL) was added, followed by CD_3CN (250 µL, 4.80 mmol, 12 equiv). Aliquots were taken every 5 minutes, taking as t = 0 once CD_3CN was added. Each aliquot was filtered through a silica plug, washed with EtOAc, and concentrated in vacuo. Deuterium incorporation was determined by ^{1}H-NMR, based on comparison with non-deuterated C4–H site.

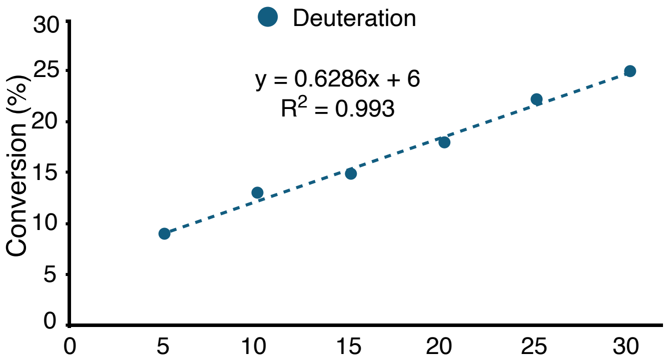

Figure S1. Kinetic profile for deuteration of **1a** at 25 °C

Kinetic profile for deuteration of 1a at 4 °C

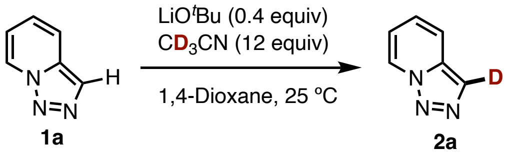

An oven-dried 8 mL vial equipped with a stirring magnetic bar was charged with **1a** (47.6 mg, 0.4 mmol, 1.0 equiv) and brought it into a nitrogen-filled glovebox. LiO*t*-Bu (12.8 mg, 0.16 mmol, 0.4 equiv) was added and the vial was taken-out from the glovebox. 1,4-dioxane (2 mL) was added, and the vial was cooled down using an ice-water bath and stirred for 5 min. Afterwards, CD_3CN (250 µL, 4.80 mmol, 12 equiv) was added. Aliquots were taken every 5 minutes, taking as t = 0 once CD_3CN was added. Each aliquot was filtered through a silica plug, washed with EtOAc, and concentrated in vacuo. Deuterium incorporation was determined by ^{1}H-NMR, based on comparison with non-deuterated C4–H site.

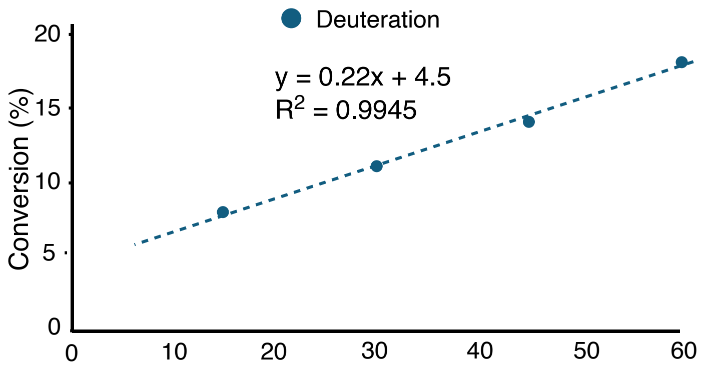

Figure S2. Kinetic profile for deuteration of **1a** at 4 ºC

Kinetic profile for protonation of 2a at 25 ºC

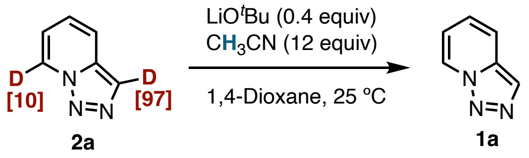

An oven-dried 8 mL vial equipped with a stirring magnetic bar was charged with **2a** (47.6 mg, 0.4 mmol, 1.0 equiv, 96% D content) and brought it into a nitrogen-filled glovebox. LiO*t*-Bu (12.8 mg, 0.16 mmol, 0.4 equiv) was added and the vial was taken-out from the glovebox. 1,4-dioxane (2 mL) was added, followed by CH_3CN (250 µL, 4.80 mmol, 12 equiv). Aliquots were taken every 5 minutes, taking as t = 0 once CH_3CN was added. Each aliquot was filtered through a silica plug, washed with EtOAc, and concentrated in vacuo. Deuterium incorporation was determined by ^{1}H-NMR, based on comparison with non-deuterated C4–H site.

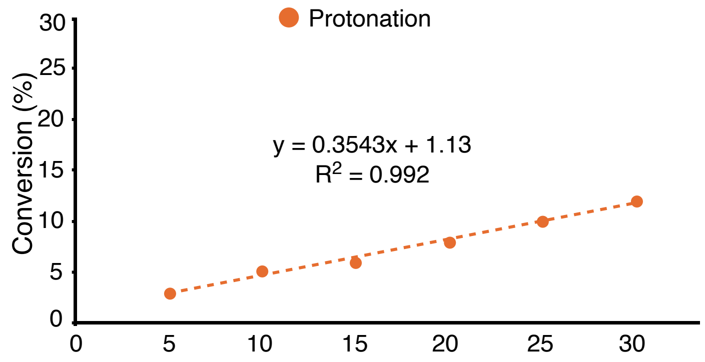

Figure S3. Kinetic profile for protonation of **2a** at 25 ºC

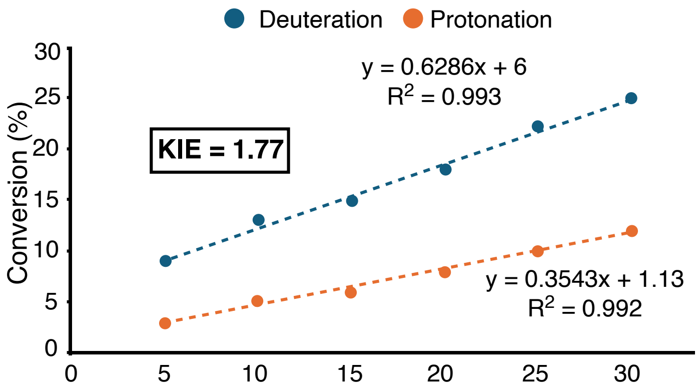

Figure S4. KIE at 25 ºC

Note: Kinetic isotope studies reveal that C3–H bond cleavage is 1.77 faster than C3–D.

Hammett-Plot experiments

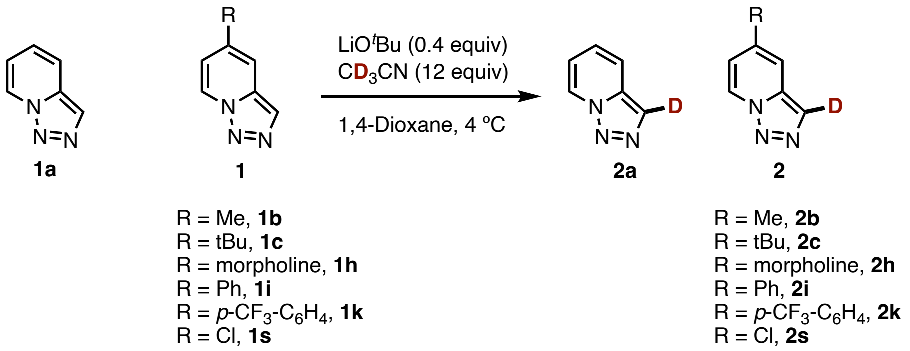

An oven-dried 8 mL vial equipped with a stirring magnetic bar was charged with **1a** (23.8 mg, 0.2 mmol, 1.0 equiv) and **1** (0.2 mmol, 1.0 equiv), and brought it into a nitrogen-filled glovebox. LiO*t*-Bu (6.4 mg, 0.0.8 mmol, 0.4 equiv) was added and the vial was taken-out from the glovebox. 1,4-dioxane (2 mL) was added, followed by CD_3CN (125 μL, 2.40 mmol, 12 equiv). Aliquots were taken every 5 minutes, taking as t = 0 once CD_3CN was added. Each aliquot was filtered through a silica plug, washed with EtOAc, and concentrated in vacuo. Deuterium incorporation was determined by ^{1}H-NMR, based on comparison with non-deuterated C4–H site.

Table S7. Hammett data

Substituent	K_X/K_H	Log K_X/K_H	σp	σm
H	1	0	0	0

Ph	0,559	-0,253	-0,01	0,06
Morpholine	4,167	0,619	-0,83	-0,16
tBu	0,943	-0,025	-0,2	-0,1
p-CF_3-C_6H_4	2,273	0,357	0,06	0,12
Me	1,695	0,229	-0,17	-0,07
Cl	2,780	0,444	0,23	0,37

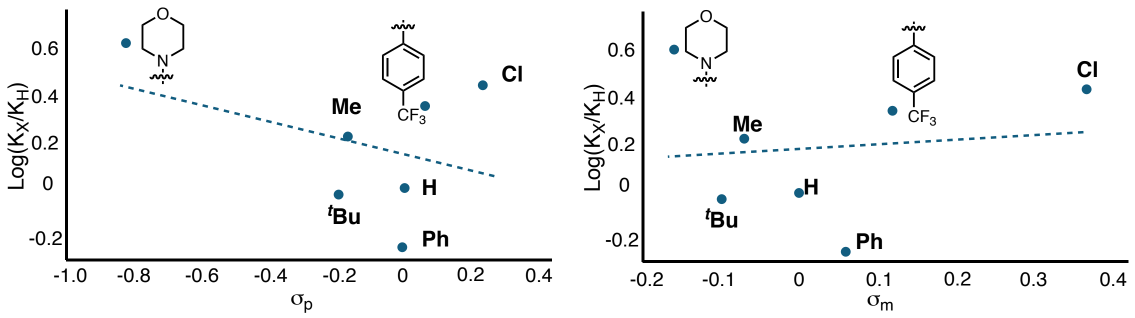

Figure S5. Hammet-studies for C5-substituted triazolopyridines

Note: The data for the chlorinated compound was recorded at 4 ºC given that our optimization studies revealed a 50% D-content with a 10:1 selectivity, and the K_X/K_H value was calculated according to Arrhenius equation. Given that σ-values for C5-substituted triazolopyridines are not tabulated, we represented the kinetic profiles vs σ_p and σ_m values. While we observed that electronics play crucial role in the deprotonation step, the inclusion of alkyl or aryl substituents did not have a significant impact in the reaction.

Diffusion-Ordered NMR Spectroscopy (DOSY) Experiments

Diffusion-Ordered NMR Spectroscopy were conducted by NMR using External Calibration Curve (ECC) method at 15 mM in 1,4-Dioxane-d_8 or THF-d_8.[30] Data was accumulated by linearly varying the diffusion encoding gradients over a range of 2% to 95% for 32 gradient increments. The signal decay dimension in the pseudo-2D dataset was obtained by Fourier transformation of the time-domain data. The diffusion profile and coefficients were determined by use of the DOSY processing tool in TopSpin 4.2.0 software.

DOSY Experiments Using LiO*t*Bu

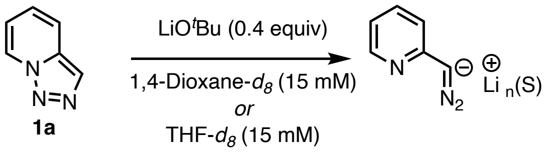

In an oven-dried 4 mL vial equipped with a stirring magnetic bar was charged with **1a** (3 mg, 25 µmol, 1.0 equiv) and the vial was brought into a nitrogen-filled glovebox. LiOtBu (0.80 mg, 10 µmol, 0.4 equiv) was added to the vial followed by 1,4-Dioxane-d_8 or THF-d_8(1.7 mL). The mixture was stirred for 5 min. The solution was then filtered through a PFTE-HPLC filter and transferred to a Young-NMR tube.

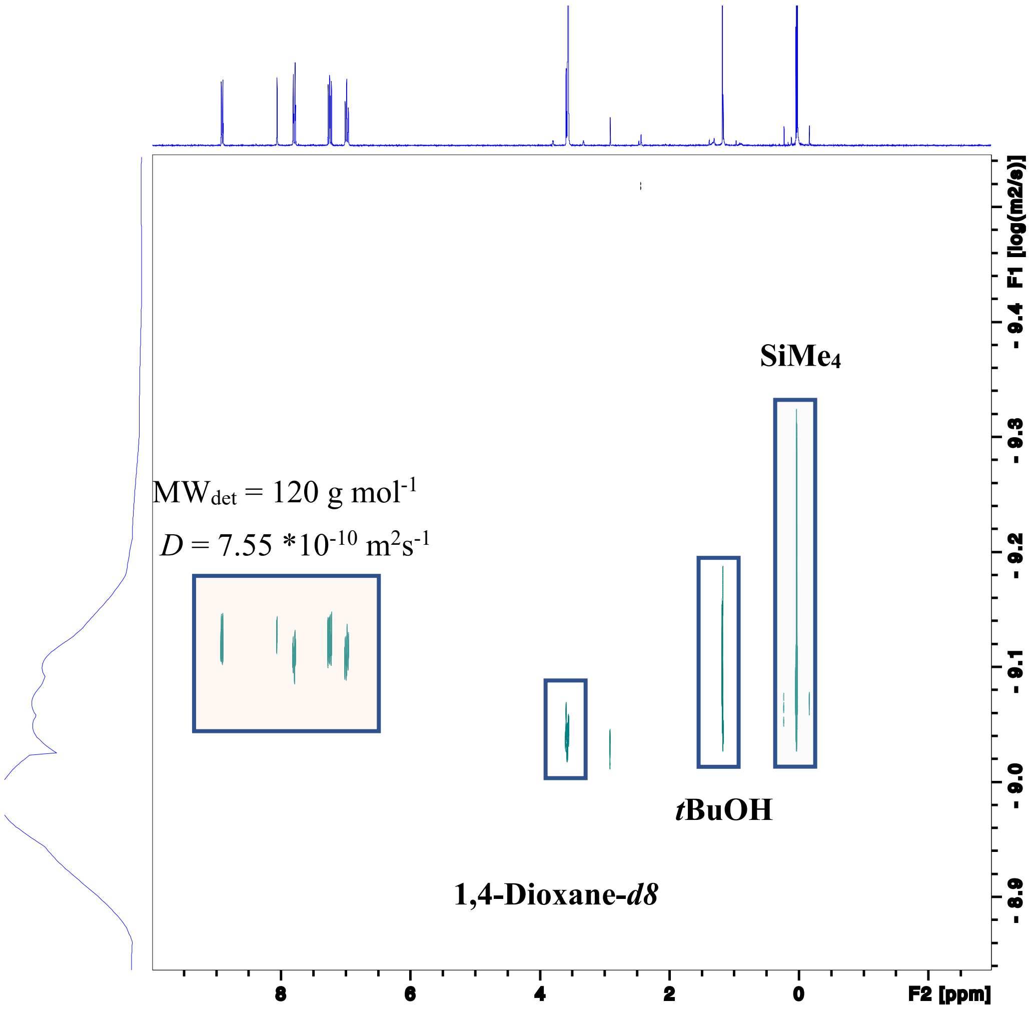

Figure S6. DOSY experiment with LiO*t*Bu in 1,4-dioxane-d_8

Note: DOSY experiments using LiOtBu as base in 1,4-dioxane-d_8 resulted in new species with MWdet = 120 g·mol^{-1}. These species are tentatively ascribed to the anionic form of pyridotriazole **1a** (MWcalcd = 118 g·mol^{-1}), thus leaving a reasonable doubt that aggregation comes into play.

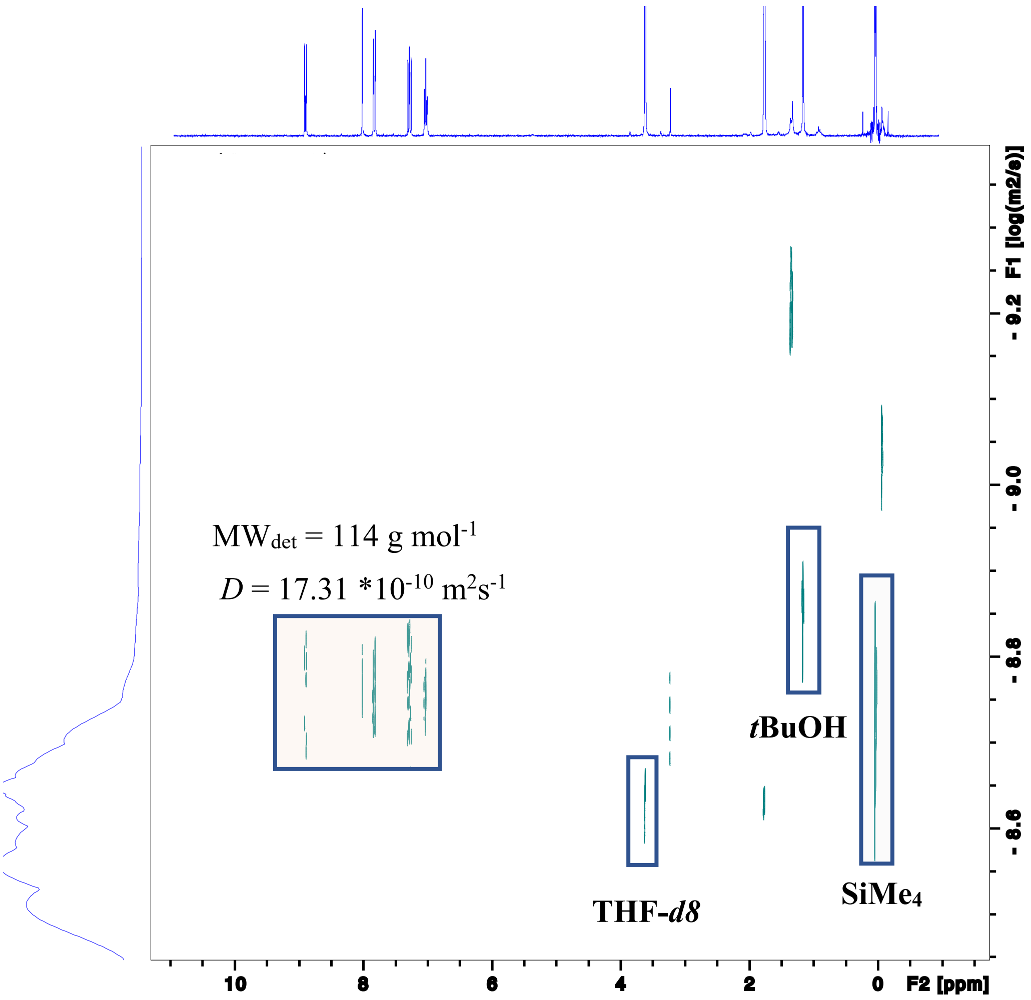

Figure S7. DOSY experiment with LiO*t*Bu in THF-d_8

Note: DOSY experiments using LiO*t*Bu as base in THF-d_8 resulted in new species with MWdet = 114 g·mol^{-1}. These species can be tentatively ascribed to the anionic form of the pyridotriazole **1a** (MWcalcd = 118 g·mol^{-1}), thus leaving a reasonable doubt that aggregation comes into play.

DOSY Experiments Using KO*t*Bu

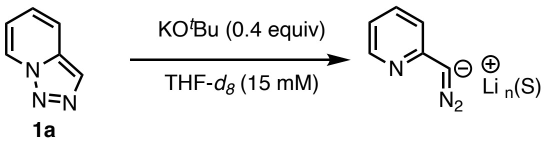

In an oven-dried 4 mL vial equipped with a stirring magnetic bar was charged with **1a** (3 mg, 25 µmol, 1.0 equiv) and the vial was brought into a nitrogen-filled glovebox. KO*t*Bu (1.10 mg, 10 µmol, 0.4 equiv) was added to the vial followed by THF-d_8(1.7 mL). The

mixture was stirred for 5 min. The solution was then filtered through a PFTE-HPLC filter and transferred to a Young-NMR tube.

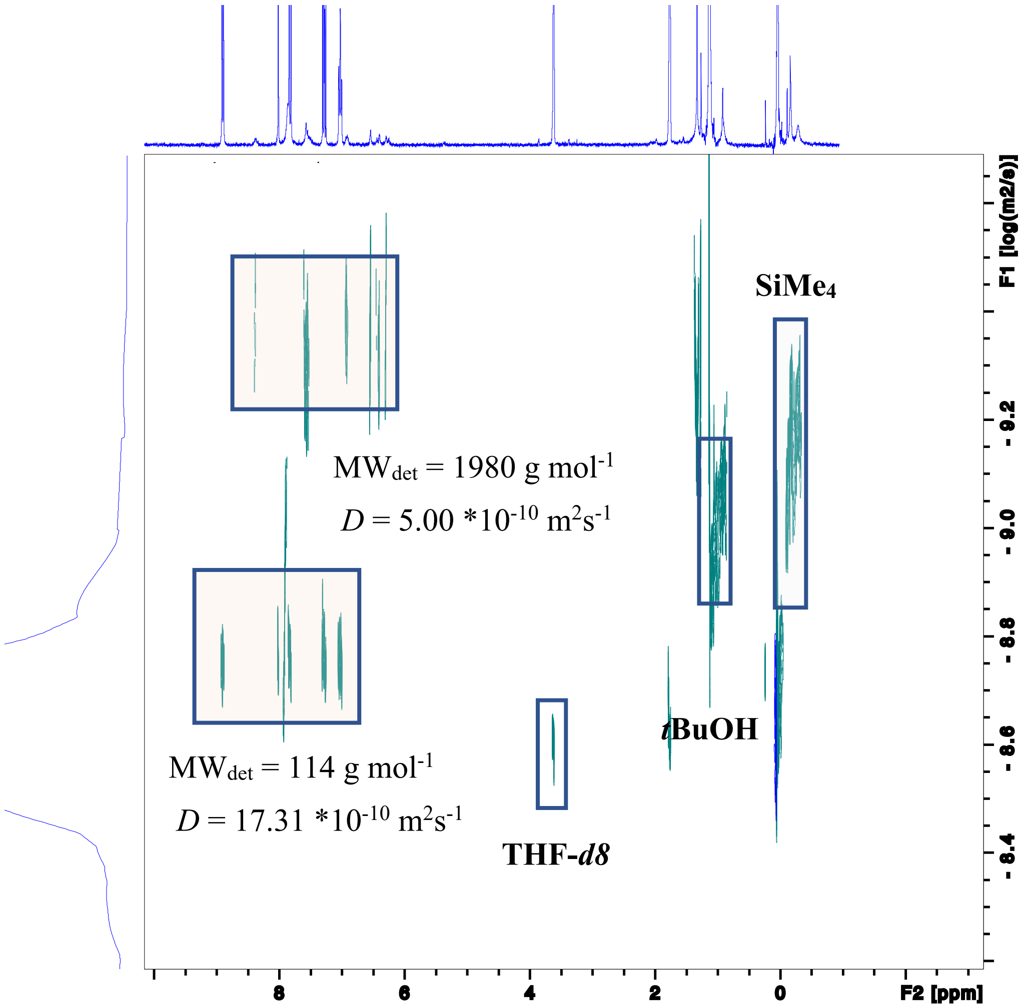

Figure S8. DOSY experiment with KO*t*Bu in THF-d_8

Note: DOSY experiments using KO*t*Bu as the base resulted in the formation of new species with MWdet = 114g·mol^{-1} that can be tentatively ascribed to the anionic form of the pyridotriazole **1a** (MWcalcd = 118 g·mol^{-1}). In addition, a new set of signals with a significantly lower diffusion coefficient was observed (D = 5.0·10^{-10}m^{2}s^{-1}), corresponding to a MWdet = 1980 g·mol^{-1}. The formation of such high-molecular-weight aggregates when using KO*t*Bu, may potentially explain the loss of site-selectivity when employing KO*t*Bu.

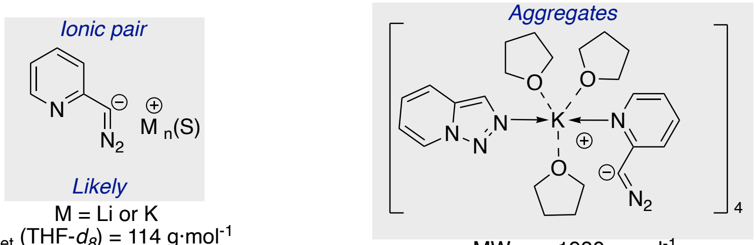

M = Li or K
MW_{det} (THF-d_8) = 114 g·mol^{-1}
MW_{det} (1,4-dioxane-d_8) = 120 g·mol^{-1}
MW_{calcd} = 118 g·mol^{-1}

MW_{det} = 1980 g·mol^{-1}
MW_{calcd}= 1915 g·mol^{-1}

Figure S9. Plausible structures

DFT Calculations

All calculations were performed using the Gaussian 09 program. All geometries were optimized at the B3LYP/6-31+G(d,p) level of theory, accounting for solvation effects when applicable using the self-consistent reaction field polarizable continuum model (IEF-PCM) in tetrahydrofuran, T = 298.15K. *pKa* values were calculated following prior literature procedures and using deprotonation at the C2 position of furan (*pKa* = 35.0) for direct comparison.[31]

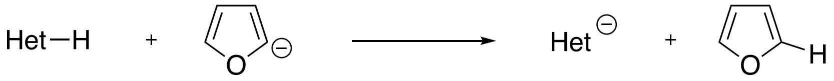

Furan

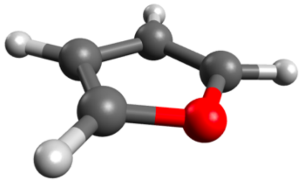

Energy (gas phase) = -229.994600
Energy (solvated) = -229.997445
0 imaginary frequencies

C	1.09745	-0.34826	-0.00013	H	2.05300	-0.84870	-0.00020
C	0.71833	0.96069	0.00013	H	1.37481	1.81906	0.00023
C	-0.71842	0.96062	-0.00004	H	-1.37499	1.81893	-0.00008
C	-1.09742	-0.34837	-0.00011	H	-2.05291	-0.84889	-0.00019
O	0.00006	-1.16106	0.00014				

Furan – C2 deprotonation

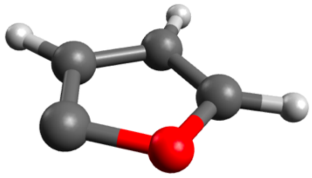

Energy (gas phase) = -229.375119
Energy (solvated) = -229.459157
0 imaginary frequencies

C	0.98477	0.53871	-0.00006	O	0.84004	-0.82254	0.00002
C	-0.24474	1.13603	0.00013	H	1.99519	0.93103	-0.00010
C	-1.19572	0.05395	-0.00002	H	-0.43145	2.20607	0.00023
C	-0.54562	-1.18240	-0.00010	H	-2.27616	0.16544	-0.00003

1a (R = H)

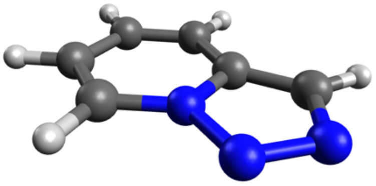

Energy (gas phase) = -395.802617
Energy (solvated) = -395.811768
0 imaginary frequencie

C	2.17245	-0.20222	0.00000	N	-2.10776	1.08137	0.00000
C	1.35685	0.89404	0.00000	N	-0.93873	1.68084	0.00000
C	1.61526	-1.51853	0.00000	H	3.24692	-0.06022	0.00000
C	0.25337	-1.69926	0.00000	H	1.68243	1.92634	0.00000
C	-0.59156	-0.56020	0.00000	H	2.27906	-2.37668	0.00000
N	0.00000	0.69621	0.00000	H	-0.18974	-2.68905	0.00000
C	-1.95500	-0.26043	0.00000	H	-2.80156	-0.92992	0.00000

1a – C3 deprotonation

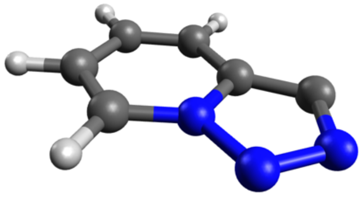

Energy (gas phase) = -395.189058
Energy (solvated) = -395.273630
0 imaginary frequencies

C	1.36485	0.83018	0.00000	N	-2.10843	1.12660	0.00000
C	2.12977	-0.30750	0.00000	N	-0.89395	1.71035	0.00000
C	1.50773	-1.59796	0.00000	H	2.12504	-2.49251	0.00000
C	0.12963	-1.68943	0.00000	H	-0.37542	-2.65187	0.00000
C	-0.68072	-0.53139	0.00000	H	1.75794	1.84114	0.00000
N	0.00000	0.71006	0.00000	H	3.21206	-0.21344	0.00000
C	-2.06842	-0.25597	0.00000				

1a – C7 deprotonation

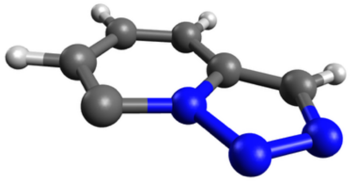

Energy (gas phase) = -395.211206
Energy (solvated) = -395.288069
0 imaginary frequencies

C	2.17670	0.00467	0.00000	N	-2.16020	-0.94137	0.00000
C	1.33886	-1.11785	0.00000	N	-1.03956	-1.62579	0.00000
C	1.73674	1.35842	0.00000	H	3.25258	-0.17294	0.00000
C	0.39028	1.66158	0.00000	H	2.46877	2.16756	0.00000
C	-0.51672	0.57557	0.00000	H	0.01768	2.68217	0.00000
N	0.00000	-0.72283	0.00000	H	-2.69389	1.12470	0.00000
C	-1.90034	0.38901	0.00000				

4a (R = H)

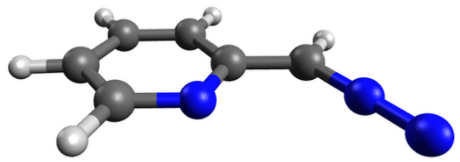

Energy (gas phase) = -395.794466
Energy (solvated) = -395.799771
0 imaginary frequencies

C	1.33375	-1.40546	0.00004	H	0.80367	2.37206	0.00003
C	2.46866	-0.59181	0.00003	H	3.46135	-1.02933	0.00002
C	2.27873	0.79382	0.00003	H	1.43314	-2.48909	0.00005
C	0.98502	1.30149	0.00003	H	3.12942	1.46942	0.00001
C	-0.09696	0.39645	0.00006	N	-2.47257	0.07600	0.00004
N	0.08034	-0.93992	0.00006	N	-3.34657	-0.65731	-0.00035
C	-1.46057	0.90076	0.00008	H	-1.70771	1.95404	0.00001

4a – C3 deprotonation

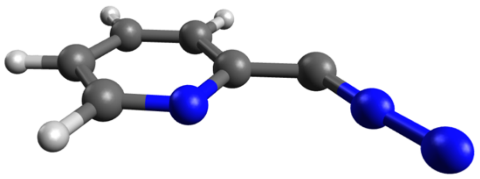

Energy (gas phase) = -395.226795
Energy (solvated) = -395.299790
0 imaginary frequencies

C	1.34288	-1.40572	0.00004	H	0.79157	2.37016	0.00003
C	2.47235	-0.58557	0.00003	H	3.46688	-1.01837	0.00002
C	2.27558	0.79980	0.00003	H	1.45060	-2.48830	0.00005
C	0.97906	1.30112	0.00003	H	3.12257	1.47951	0.00001
C	-0.09890	0.39165	0.00006	N	-2.47418	0.07674	0.00003
N	0.08438	-0.94580	0.00006	N	-3.34906	-0.65812	-0.00034
C	-1.46302	0.89790	0.00008	H	-1.70737	1.95224	0.00001

1b (R = Me)

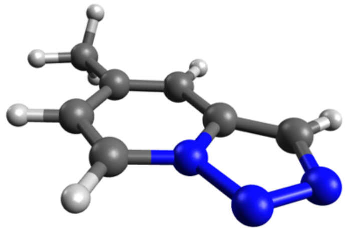

Energy (gas phase) = -435.097734
Energy (solvated) = -435.107395
0 imaginary frequencies

C	-0.09110	1.60722	0.00000	H	-1.02661	-2.17175	0.00000
C	-1.40340	1.22581	0.00000	H	0.26825	2.62836	0.00000
C	-1.78158	-0.15774	0.00000	H	-2.16863	1.99431	0.00000
C	-0.78689	-1.11281	0.00000	H	2.10376	-2.36033	0.00000
C	0.57436	-0.72138	0.00000	C	-3.24326	-0.54449	0.00000
N	0.87063	0.63374	0.00000	H	-3.88912	0.33743	-0.00000
C	1.83945	-1.31393	0.00000	H	-3.49528	-1.14236	0.88288
N	2.77760	-0.34201	0.00000	H	-3.49528	-1.14236	-0.88288
N	2.21712	0.84549	0.00000				

1b – C3 deprotonation

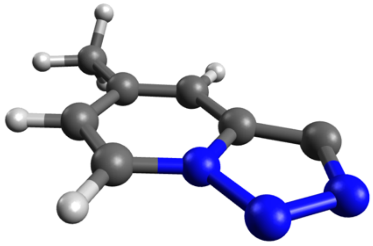

Energy (gas phase) = -435.481657
Energy (solvated) = -434.567117
0 imaginary frequencies

C	-0.03849	1.59331	0.00000	N	2.25012	0.78558	0.00000
C	-1.36106	1.22885	0.00000	H	-0.99647	-2.17051	0.00000
C	-1.75295	-0.15167	0.00000	H	-2.11700	2.01018	0.00000
C	-0.75466	-1.10935	0.00000	C	-3.22044	-0.52781	0.00000
C	0.61602	-0.76589	0.00000	H	-3.85771	0.36417	-0.00000
N	0.91759	0.61581	0.00000	H	-3.49273	-1.12235	0.88260
C	1.84398	-1.46967	0.00000	H	-3.49273	-1.12235	-0.88260
N	2.78149	-0.45331	0.00000	H	0.31779	2.61765	0.00000

1b – C7 deprotonation

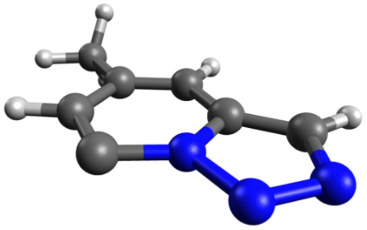

Energy (gas phase) = -434.505636
Energy (solvated) = -434.582568
0 imaginary frequencies

C	-0.06681	1.73601	0.00000	N	2.21108	0.87618	0.00000
C	-1.37763	1.24525	0.00000	H	-1.01578	-2.16462	0.00000
C	-1.76296	-0.12958	0.00000	H	-2.18425	1.98070	0.00000
C	-0.77968	-1.10250	0.00000	H	2.09729	-2.33174	0.00000
C	0.57059	-0.68462	0.00000	C	-3.22894	-0.51513	0.00000
N	0.84558	0.68400	0.00000	H	-3.86720	0.37516	-0.00000
C	1.83271	-1.28252	0.00000	H	-3.49167	-1.11297	0.88332
N	2.77613	-0.30804	0.00000	H	-3.49167	-1.11297	-0.88332

4b (R = Me)

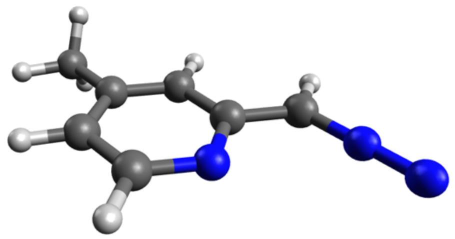

Energy (gas phase) = -435.092089
Energy (solvated) = -435.097984
0 imaginary frequencies

C	-0.60944	1.75676	-0.00002	N	3.85919	0.17594	0.00030
C	-1.86979	1.15624	0.00004	H	-0.76248	-2.04562	-0.00005
C	-1.95442	-0.24343	0.00003	H	-2.76613	1.76901	0.00009
C	-0.75530	-0.95838	-0.00004	H	-0.52060	2.84148	-0.00002
C	0.47012	-0.26598	-0.00010	C	-3.28420	-0.95758	0.00011
N	0.54278	1.08107	-0.00008	H	-4.11697	-0.24931	-0.00002
C	1.71962	-1.01080	-0.00018	H	-3.38591	-1.59937	0.88255
N	2.86465	-0.38360	-0.00012	H	-3.38587	-1.59965	-0.88215
H	1.77194	-2.09130	-0.00011				

4b – C3 deprotonation

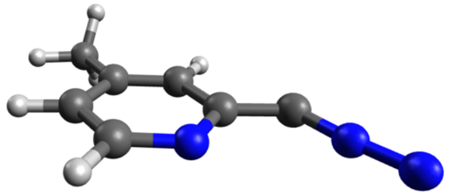

Energy (gas phase) = -434.521680
Energy (solvated) = -434.595171
0 imaginary frequencies

C	-0.65369	1.75431	0.00004	N	3.98251	-0.01468	-0.00006
C	-1.90415	1.12554	0.00006	H	-0.67118	-2.03943	-0.00005
C	-1.92548	-0.28554	0.00003	H	-2.82168	1.70926	0.00010
C	-0.71141	-0.95266	-0.00004	H	-0.60820	2.84694	0.00007
C	0.53422	-0.24602	-0.00012	C	-3.23572	-1.04493	0.00009
N	0.52382	1.13377	-0.00003	H	-4.09200	-0.36119	-0.00009
C	1.75011	-0.98597	-0.00005	H	-3.32348	-1.69133	0.88241
N	2.88177	-0.39924	0.00003	H	-3.32336	-1.69166	-0.88200

1c (R = Cl)

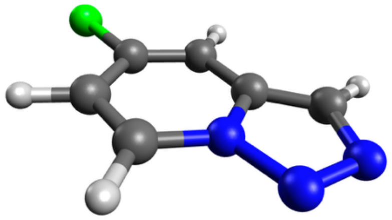

Energy (gas phase) = -855.406052
Energy (solvated) = -855.414792
0 imaginary frequencies

C	0.36843	-1.64973	0.00000	N	-3.01660	-0.89951	0.00000
C	1.45217	-0.81995	0.00000	N	-2.05638	-1.79204	0.00000
C	1.25570	0.59611	0.00000	H	-0.15062	2.22073	0.00000
C	0.00000	1.14798	0.00000	H	0.41002	-2.73135	0.00000
C	-1.11547	0.27233	0.00000	H	2.45273	-1.23331	0.00000
N	-0.88417	-1.09623	0.00000	H	-3.14271	1.22607	0.00000
C	-2.50808	0.35335	0.00000	Cl	2.67142	1.62539	0.00000

1c – C3 deprotonation

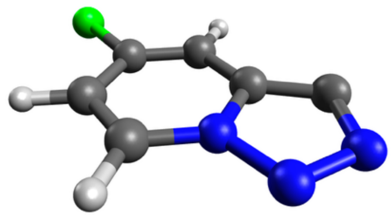

Energy (gas phase) = -854.801260
Energy (solvated) = -854.880549
0 imaginary frequencies

C	-0.25622	-1.66712	0.00000	N	3.08884	-0.70413	0.00000
C	-1.38129	-0.88529	0.00000	N	2.16790	-1.68162	0.00000
C	-1.23532	0.53604	0.00000	H	0.11517	2.21427	0.00000
C	0.00000	1.13562	0.00000	H	-2.36383	-1.34229	0.00000
C	1.16766	0.33565	0.00000	Cl	-2.71838	1.52142	0.00000
N	0.97268	-1.06804	0.00000	H	-0.26751	-2.75139	0.00000
C	2.55895	0.57706	0.00000				

1c – C7 deprotonation

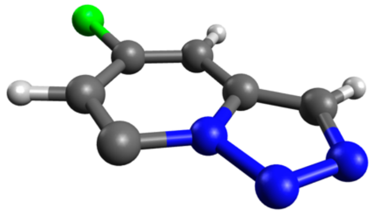

Energy (gas phase) = -854.828493
Energy (solvated) = -854.899379
0 imaginary frequencies

C	-0.37515	-1.78476	0.00000	N	3.01402	-0.91760	0.00000
C	-1.43209	-0.86766	0.00000	N	2.05491	-1.81085	0.00000
C	-1.24088	0.53578	0.00000	H	0.14158	2.19991	0.00000
C	0.00000	1.12585	0.00000	H	-2.45201	-1.24414	0.00000
C	1.10446	0.24045	0.00000	H	3.12628	1.21489	0.00000
N	0.85621	-1.13287	0.00000	Cl	-2.68250	1.60835	0.00000
C	2.49544	0.33645	0.00000				

4c (R = Cl)

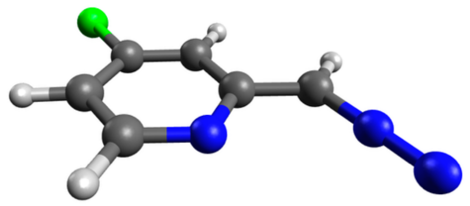

Energy (gas phase) = -855.401551
Energy (solvated) = -855.406906
0 imaginary frequencies

C	0.09934	1.88008	-0.00005	N	-3.19491	-0.52311	-0.00005
C	1.40793	1.39283	0.00002	H	-1.97556	-2.14532	-0.00006
C	1.56409	0.00533	0.00014	N	-4.22727	-0.04014	-0.00023
C	0.45502	-0.82798	0.00016	H	0.56572	-1.90641	0.00003
C	-0.82071	-0.22512	0.00026	H	2.26034	2.06107	-0.00018
N	-0.99336	1.11188	0.00012	H	-0.07582	2.95375	-0.00010
C	-2.00513	-1.06417	0.00007	Cl	3.17240	-0.69232	-0.00012

4c – C3 deprotonation

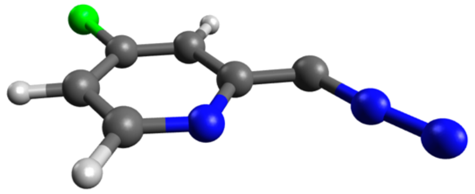

Energy (gas phase) = -854.842095
Energy (solvated) = -854.909546
0 imaginary frequencies

C	0.12848	1.89475	-0.00009	N	-3.21057	-0.54866	0.00005
C	1.43430	1.38654	-0.00005	N	-4.33765	-0.25897	0.00032
C	1.52831	-0.01363	-0.00001	H	0.47925	-1.89397	-0.00001
C	0.41125	-0.81285	-0.00006	H	2.30563	2.02995	-0.00002
C	-0.89064	-0.20092	-0.00023	H	-0.00918	2.97866	-0.00010
N	-0.98995	1.17508	-0.00014	Cl	3.14811	-0.76278	0.00009
C	-2.03277	-1.04049	-0.00005				

1d (R = NMe2)

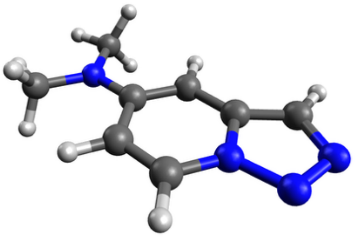

Energy (gas phase) = -529.713699
Energy (solvated) = -529.725734
0 imaginary frequencies

C	-0.75941	1.67303	-0.00058	N	2.43521	-0.18188	-0.00048
C	0.57817	1.40975	-0.00063	C	3.38614	0.92035	0.00146
C	1.07559	0.05136	-0.00028	H	3.28040	1.55418	0.89221
C	0.14196	-0.98555	0.00011	H	3.28343	1.55485	-0.88919
C	-1.24074	-0.68838	0.00012	H	4.39773	0.51374	0.00307
N	-1.64457	0.63496	-0.00018	C	2.91910	-1.55235	-0.00079
C	-2.45654	-1.37953	0.00046	H	2.57975	-2.10087	0.88908
N	-3.47100	-0.48298	0.00049	H	4.00919	-1.55087	-0.00239
N	-3.01432	0.74280	-0.00006	H	2.57729	-2.10098	-0.88960
H	0.44011	-2.02479	0.00051	H	-1.19126	2.66591	-0.00088
H	1.25869	2.24968	-0.00111	H	-2.63822	-2.44325	0.00077

1d – C3 deprotonation

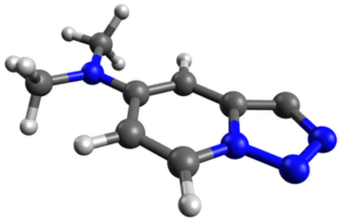

Energy (gas phase) = -529.097610
Energy (solvated) = -529.180077
0 imaginary frequencies

C	-0.79161	1.66687	-0.00771	N	2.38696	-0.18156	-0.02249
C	0.55427	1.41698	-0.01153	C	3.28479	0.93216	0.02680
C	1.01976	0.03700	-0.00974	H	3.15702	1.55981	0.93065
C	0.11319	-1.00474	-0.00347	H	3.18067	1.61133	-0.83876
C	-1.28381	-0.79608	-0.00084	H	4.31596	0.58322	0.02975
N	-1.64847	0.61397	-0.00267	C	2.85801	-1.55346	-0.00180
C	-2.48611	-1.47263	-0.00022	H	2.51505	-2.10122	0.89036
N	-3.44415	-0.57245	0.02090	H	3.94406	-1.56101	-0.01431
N	-2.96468	0.68918	-0.00135	H	2.50090	-2.11349	-0.87541
H	0.44903	-2.04045	-0.00295	H	-1.25420	2.63643	-0.00931
H	1.27290	2.22484	-0.01970				

1d – C7 deprotonation

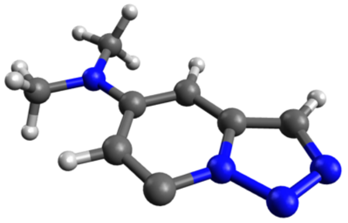

Energy (gas phase) = -529.117522
Energy (solvated) = -529.198470
0 imaginary frequencies

C	0.79886	-1.80453	-0.00080	N	3.01269	-0.77625	0.00006
C	-0.54445	-1.42754	-0.00094	H	-0.41752	2.01547	0.00041
C	-1.04352	-0.07762	-0.00057	H	-1.26932	-2.23663	-0.00158
C	-0.12632	0.97231	-0.00005	N	-2.42195	0.17337	-0.00088
C	1.25012	0.64746	0.00010	C	-3.37201	-0.91950	0.00196
N	1.63248	-0.68902	-0.00021	H	-3.26464	-1.56251	0.88840
C	2.46029	1.34495	0.00060	H	-3.26868	-1.56383	-0.88403
N	3.48034	0.44651	0.00064	H	-4.38619	-0.51069	0.00396

C	-2.89900	1.53646	-0.00078	H	-2.55473	2.09483	-0.88690
H	-2.55714	2.09439	0.88664	H	2.64147	2.41172	0.00099
H	-3.99204	1.54294	-0.00234				

4d (R = NMe2)

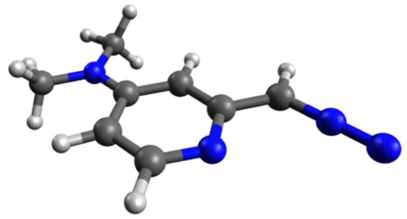

Energy (gas phase) = -529.710837
Energy (solvated) = -529.717837
0 imaginary frequencies

C	-0.32571	1.96406	-0.00017	H	-0.52240	3.03467	0.00090
C	0.98986	1.52135	-0.00128	N	2.51975	-0.37874	-0.00567
C	1.24662	0.12329	-0.00290	C	3.66437	0.52587	0.00435
C	0.11104	-0.71945	-0.00186	C	2.73983	-1.82013	0.00164
C	-1.17254	-0.15728	-0.00091	H	3.67208	1.17642	-0.87919
N	-1.41091	1.17350	-0.00014	H	4.58158	-0.06202	-0.00091
C	-2.32676	-1.05008	-0.00037	H	3.67102	1.16027	0.89986
N	-3.53567	-0.56867	0.00107	H	2.29902	-2.29976	-0.88154
N	-4.59349	-0.13311	0.00229	H	2.31372	-2.29021	0.89746
H	0.21171	-1.79728	-0.00171	H	3.81132	-2.01721	-0.00671
H	1.79165	2.24790	-0.00093	H	-2.24757	-2.12946	-0.00106

4d – C3 deprotonation

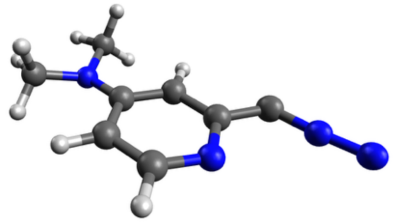

Energy (gas phase) = -529.139689
Energy (solvated) = -529.213145
0 imaginary frequencies

C	-0.32895	1.97142	-0.00735	H	-0.51107	3.04679	0.02068
C	0.98761	1.52252	-0.04644	N	2.50535	-0.40106	-0.16882
C	1.21978	0.12037	-0.08712	C	3.64086	0.45908	0.14375
C	0.08222	-0.70276	-0.05840	C	2.69921	-1.83143	0.03334
C	-1.22637	-0.15694	-0.02106	H	3.65649	1.33874	-0.50560
N	-1.42318	1.20040	0.00373	H	4.56353	-0.09570	-0.03484
C	-2.35400	-1.05667	-0.00340	H	3.63961	0.80300	1.19056
N	-3.53759	-0.60427	0.03104	H	2.10642	-2.40767	-0.68260
N	-4.67464	-0.31918	0.06135	H	2.42398	-2.15912	1.04871
H	0.16604	-1.78149	-0.06643	H	3.74984	-2.07391	-0.13524
H	1.79347	2.24462	-0.04615				

1e (R = OMe)

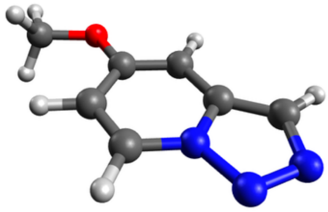

Energy (gas phase) = -510.300996
Energy (solvated) = -510.312148
0 imaginary frequencies

C	-0.16161	-1.56873	0.00000	H	0.06081	2.31854	0.00000
C	-1.30731	-0.82187	0.00000	H	-0.12852	-2.65087	0.00000
C	-1.22966	0.60982	0.00000	H	-2.25887	-1.33581	0.00000
C	0.00000	1.23686	0.00000	H	3.12778	1.54683	0.00000
C	1.17307	0.44922	0.00000	O	-2.32977	1.41292	0.00000
N	1.04480	-0.93309	0.00000	C	-3.62770	0.82355	0.00000
C	2.55870	0.63011	0.00000	H	-4.32761	1.65943	0.00000
N	3.15789	-0.58220	0.00000	H	-3.79147	0.21512	0.89749
N	2.27083	-1.54550	0.00000	H	-3.79147	0.21512	-0.89749

1e – C3 deprotonation

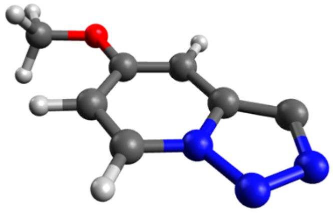

Energy (gas phase) = -509.681879
Energy (solvated) = -509.771368
0 imaginary frequencies

C	-0.06464	-1.57073	0.00000	H	0.03298	2.31342	0.00000
C	-1.24370	-0.86401	0.00000	H	-2.17548	-1.41620	0.00000
C	-1.20855	0.56685	0.00000	O	-2.36273	1.35043	0.00000
C	0.00000	1.22857	0.00000	C	-3.61132	0.69667	0.00000
C	1.22010	0.51242	0.00000	H	-4.36821	1.48579	0.00000
N	1.12066	-0.90061	0.00000	H	-3.75125	0.07028	0.89402
C	2.59436	0.84322	0.00000	H	-3.75125	0.07028	-0.89402
N	3.20866	-0.39672	0.00000	H	-0.01024	-2.65401	0.00000
N	2.35750	-1.43851	0.00000				

1e – C7 deprotonation

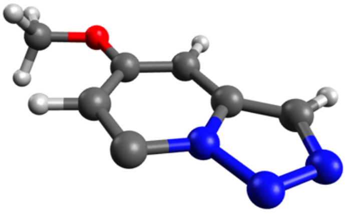

Energy (gas phase) = -509.712831
Energy (solvated) = -509.790541
0 imaginary frequencies

C	-0.16381	-1.69997	0.00000	H	0.05844	2.30573	0.00000
C	-1.28121	-0.85463	0.00000	H	-2.25595	-1.33444	0.00000
C	-1.21601	0.56776	0.00000	H	3.10999	1.53807	0.00000
C	0.00000	1.22271	0.00000	O	-2.33729	1.39019	0.00000
C	1.16189	0.42042	0.00000	C	-3.61337	0.78114	0.00000
N	1.01688	-0.96749	0.00000	H	-4.33748	1.60110	0.00000
C	2.54452	0.61597	0.00000	H	-3.77163	0.16025	0.89235
N	3.15488	-0.59764	0.00000	H	-3.77163	0.16025	-0.89235
N	2.26745	-1.55957	0.00000				

4e (R = OMe)

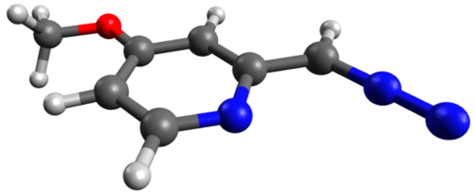

Energy (gas phase) = -510.298653
Energy (solvated) = -510.306047
0 imaginary frequencies

C	-0.16365	1.75760	0.00014	H	-0.35711	-2.04290	0.00015
C	-1.43525	1.17714	0.00031	H	-2.31634	1.80573	0.00043
C	-1.50425	-0.22417	0.00032	H	-0.07357	2.84233	0.00013
C	-0.31586	-0.95899	0.00015	O	-2.65626	-0.94444	0.00043
C	0.90452	-0.26879	-0.00002	C	-3.89913	-0.24507	-0.00020
N	0.98534	1.08155	-0.00002	H	-4.67041	-1.01566	0.00004
C	2.15177	-1.01643	-0.00020	H	-4.00400	0.37685	-0.89690
N	3.29776	-0.39103	-0.00035	H	-4.00436	0.37778	0.89582
N	4.29480	0.16383	-0.00048	H	2.20183	-2.09698	-0.00021

4e – C3 deprotonation

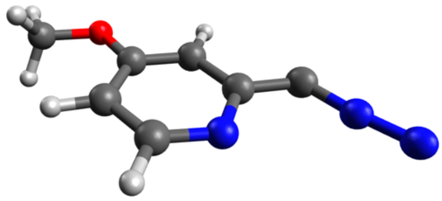

Energy (gas phase) = -509.728043
Energy (solvated) = -509.803362
0 imaginary frequencies

C	-0.18433	1.76623	0.00001	H	-0.28243	-2.02658	0.00000
C	-1.45406	1.16784	0.00004	H	-2.34858	1.77839	0.00006
C	-1.46982	-0.24142	0.00003	H	-0.12717	2.85814	0.00001
C	-0.27765	-0.94223	0.00001	O	-2.63242	-0.99469	0.00006
C	0.97240	-0.25048	-0.00001	C	-3.86702	-0.30726	0.00007
N	0.98144	1.13313	-0.00001	H	-4.64278	-1.07752	0.00007
C	2.18031	-1.00069	-0.00006	H	-3.98510	0.32222	-0.89323
N	3.31657	-0.42311	-0.00010	H	-3.98509	0.32221	0.89337
N	4.42078	-0.04878	-0.00008				

References

(1) López-Rodríguez, R.; Ros, A.; Fernández, R.; Lassaletta, J. M. *J. Org. Chem.* **2012**, *77*, 9915−9920.

(2) Comba, P.; Morgen, M.; Wadepohl, H. *Inorg. Chem.* **2013**, *52*, 6481.

(3) Dou, D. Y.; Qi, D. F.; Zhao, T. Y.; Zheng, P. X.; Bao, X. *Dalton Trans.* **2024**, *53*, 8619–8625.

(4) Witty, D. R.; Macpherson, D. T.; Giblin, G. M. P.; Stanway, S. J.; Vong, A. K. K. WO 2013/093497A1, **2013**.

(5) Charoensutthivarakul, S.; Hong, W. D.; Leung, S. C.; Gibbons, P. D.; Bedingfield, P. T. P.; Nixon, G. L.; Lawrenson, A. S.; Berry, N. G.; Ward, S. A.; Biagini, G. A.; O'Neill, P. M. (2015). *Med. Chem. Commun.* **2015***, 6,* 1252–1259.

(6) Roy, S.; Das, S. K.; Chattopadhyay, B. *Angew. Chem., Int. Ed.* **2018**, *57*, 2238−2243.

(7) Yang, Y.; Jia, J. H.; Pei, X. L.; Zheng, H.; Nan, Z. A.; Wang, Q. M. *Chem. Commun.* **2015**, *51*, 3804–3807.

(8) Scocchera, E.; Reeve, S. M.; Keshipeddy, S.; Lombardo, M. N.; Hajian, B.; Sochia, A. E.; Wright, D. L. *ACS Med. Chem. Lett.* **2016**, *7*, 692−696.

(9) Nara, S. J. *et al*. *J. Med. Chem.* **2022**, *65*, 8948−8960.

(10) WO2013/102145A1, **2013**.

(11) Cong, X.; Zhuo, Q; Hao, N; Mishra, A; Nishiura, M; Hou, Z. *Angew. Chem. Int. Ed.* **2024**, *63*, e202318203.

(12) Kumar, S. *J. Org. Chem.* **2002**, *67*, 8842−8846.

(13) Raghavendra, M. S.; Lam, Y. *Tetrahedron letters*, **2004**, *45*, 6129−6132.

(14) Xin, L.; Wan, W.; Yu, Y.; Wan, Q.; Ma, L.; Huang, X. *ACS Catal.* **2021**, 11, 1570−1577.

(15) Liu, S.; Sawicki, J.; Driver, T. G. *Org. Lett.* **2012***,* 14, 3744−3747.

(16) Reddy, R. J.; Sharadha, N.; Kumari, A. H. *Org. Biomol. Chem.* **2022**, *20*, 4331–4337.

(17) Dwyer, M.P.; Paruch, K.; Alvarez, C.; Doll, R. J.; Keertikar, K.; Duca, J.; Fischmann, T. O.; Hruza, A.; Madison, V.; Lees, E.; Parry, D.; Seghezzi, W.; Sgambellone, N.; Shanahan, F.; Wiswell, D.; Guzi, T. J. *Bioorg. Med. Chem. Lett.* **2007**, 6216−6219.

(18) Magavi, S. S.; Parks, D. J.; Tait, B. D.; Cho, J.; Agrawal, R.; Shaw, P.R. WO2023/081923 A1, **2023**.

(19) Kendall, J. D.; O'Connor, P. D.; Marshall, A. J.; Frédérick, R.; Marshall, E. S.; Lill, C. L.; Lee, W. J.; Kolekar, S.; Chao, M.; Malik, A.; Yu, S. Q.; Chaussade, C.; Buchanan, C.; Rewcastle, G. W.; Baguley, B. C.; Flanagan, J. U.; Jamieson, S. M. F.; Denny, W. A.; Shepherd, P. R. *Bioorg. Med. Chem.* **2012**, *20*, 68–85.

(20) Mennie, K. M.; Reutershan, M. H.; White, C.; Adams, B.;Becker, B.; Deng, J.; Katz, J. D.; LaBlue, E.; Margrey, K.; Saurí, J. *Org. Lett.* **2021**, 23, 4694–4698.

(21) Dong, C.; Wang, X.; Pei, Z.; Shen, R. *Org. Lett.* **2019**, 21, 4148−4152.

(22) Diana, L.; Hsin-Ping L.; Tourin, B.; Pascal, R., Mouad, A.; Abdallah, H. *Eur. J. Org. Chem.* **2019**, 2602–2611

(23) Ruwei, S.; Chao, D.; Jianlin, Y.; Li-Biao, H. *Adv. Synth. Catal.* **2018**, *360*, 4252 – 4258.

(24) Helan, V.; Gulevicha, A. V.; Gevorgyan, V. *Chem. Sci.*, **2015**, *6*, 1928–1931.

(25) a) Neufeld, R.; Stalke, D. *Chem. Sci.* **2015**, *6*, 3354–3364. b) Bole, L. J.; Tortajada, A.; Hevia, E. *Angew. Chem. Int. Ed.* **2022**, *61*, e202204262.

(26) Watanabe, A.; Sato, Y.; Ogura, K.; Tatsumi, Y. WO/2017047602A1, **2017**.

(27) Masse, C. E.; Greenwood, J. R.; Mondal, S.; Xu, J.; Ghanakota, P.; McRobb, F. M.; Boyles, N. WO/2022140527A1, **2022**.

(28) Fu, J. WO/2003078435A1, **2003**.

(29) Prante, O.; Tietze, R.; Hocke, C.; Löber, S.; Hübner, H.; Kuwert, T.; Gmeiner, P. *J. Med. Chem.* **2008**, *51*, 1800–1810.

(30) a) Neufeld, R.; Stalke, D. *Chem. Sci.* **2015**, *6*, 3354–3364. b) Bole, L. J.; Tortajada, A.; Hevia, E. *Angew. Chem. Int. Ed.* **2022**, *61*, e202204262.

(31) Shen, K.; Fu, Y.; Li, J-N.; Liu, L.; Guo, Q-X. *Tetrahedron* **2007**, *63*, 1568–1576.

NMR Spectra for Known Compounds

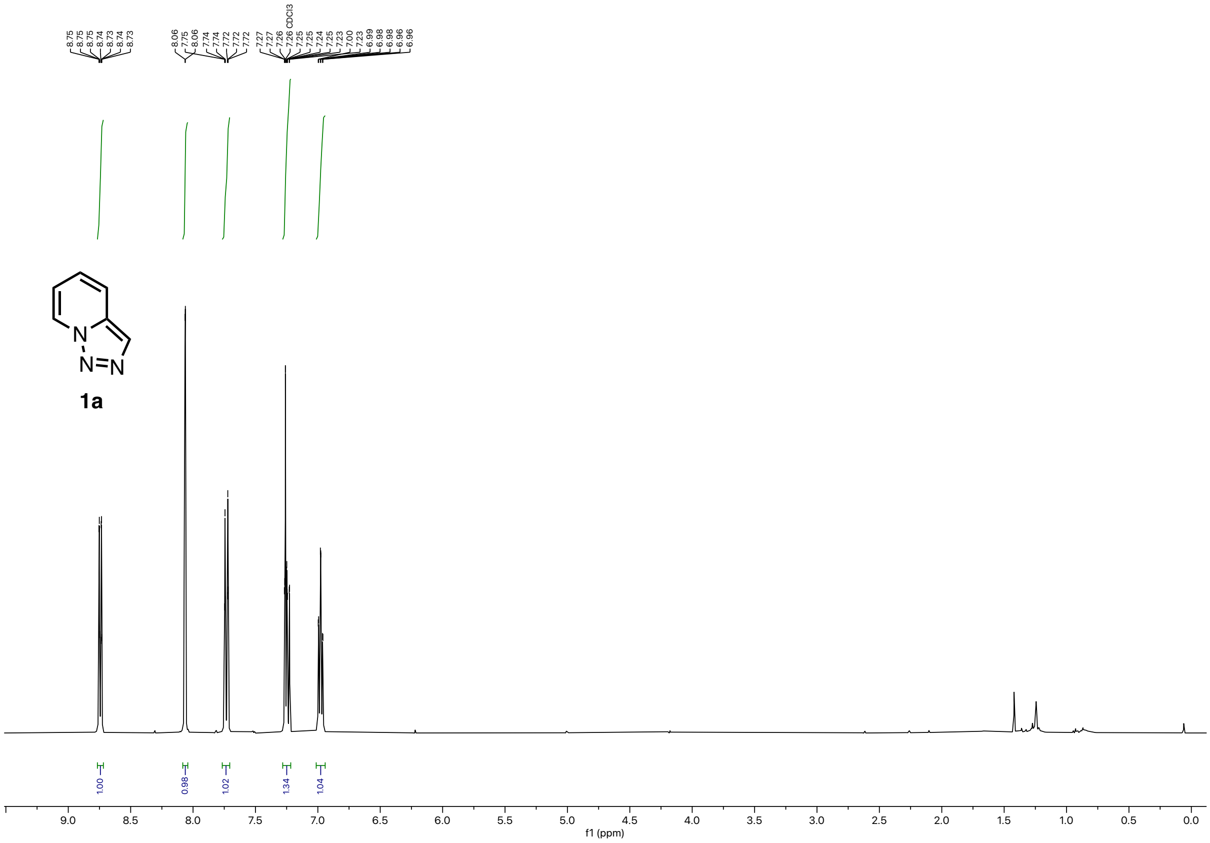

^{1}H spectrum (400 MHz, $CDCl_3$) of **1a**

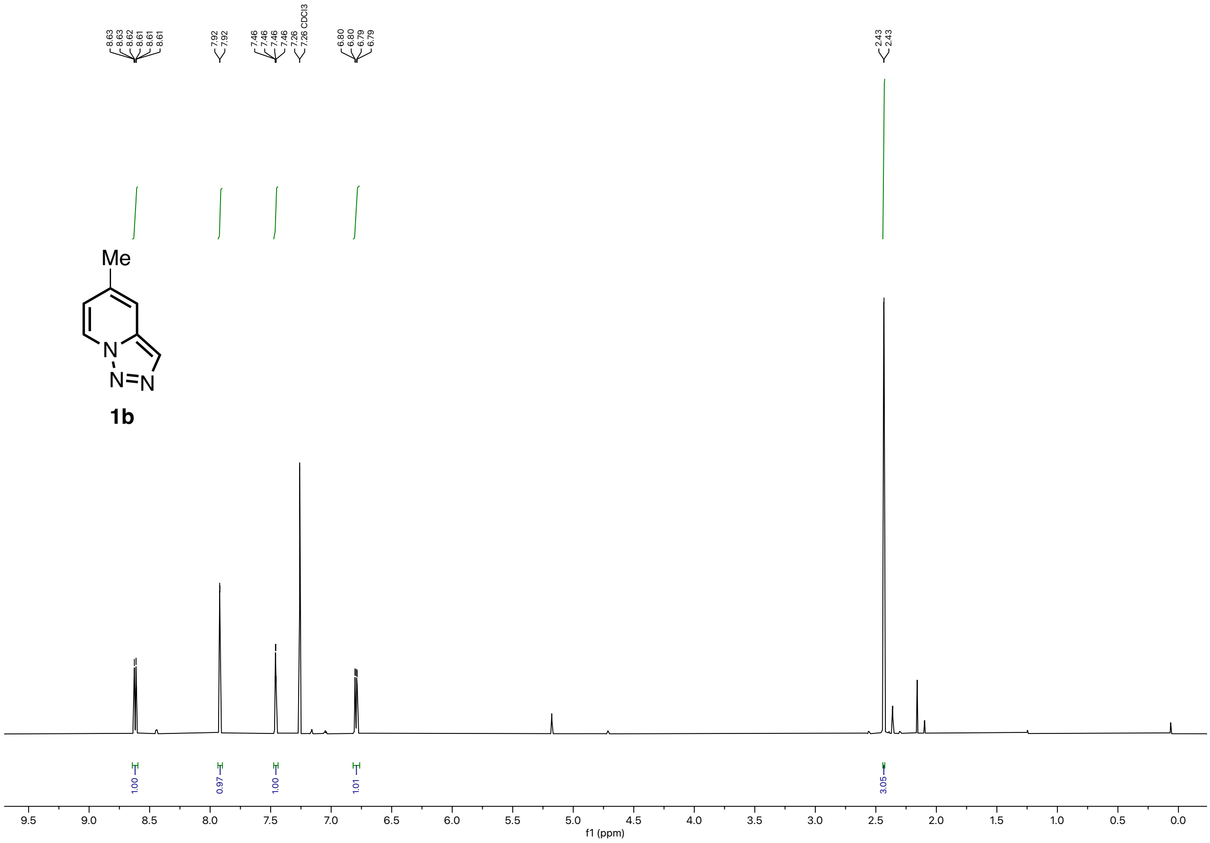

^{1}H spectrum (500 MHz, $CDCl_3$) of **1b**

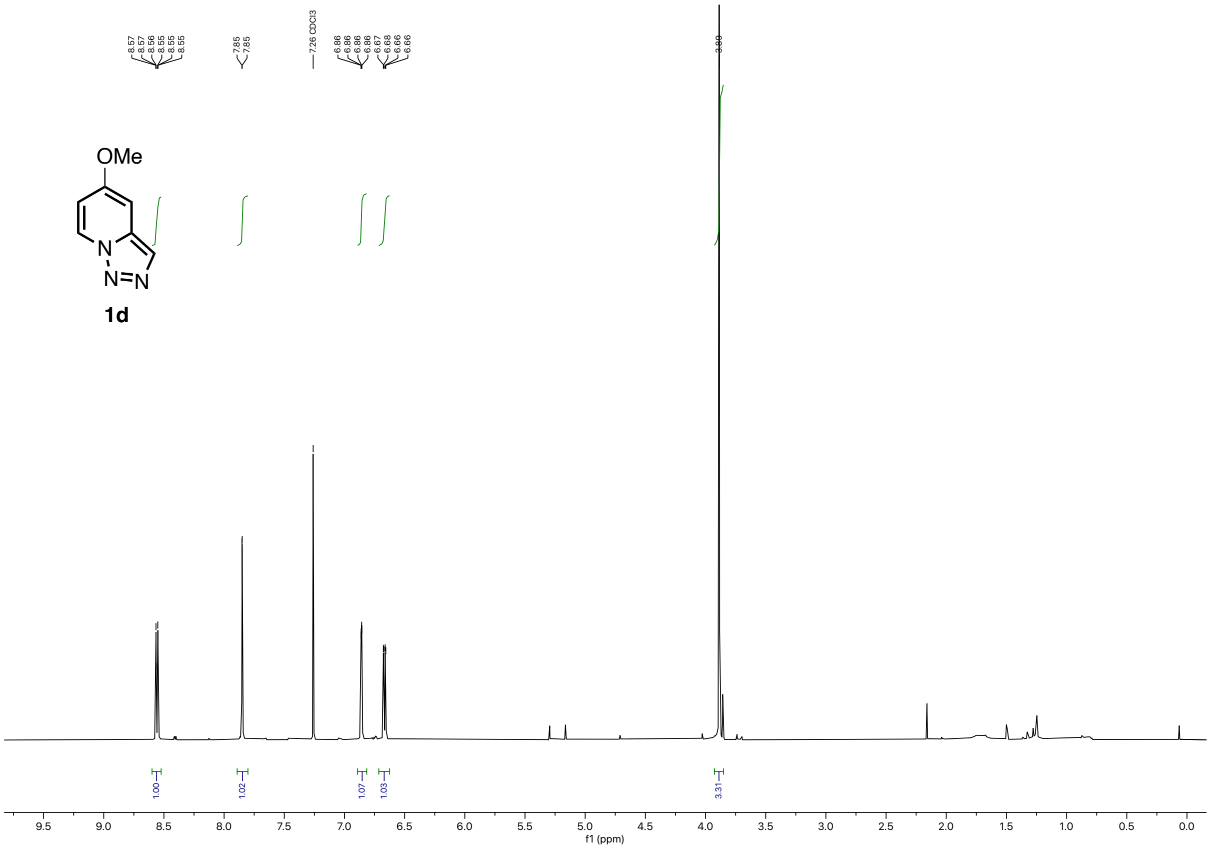

^{1}H spectrum (500 MHz, $CDCl_3$) of **1d**

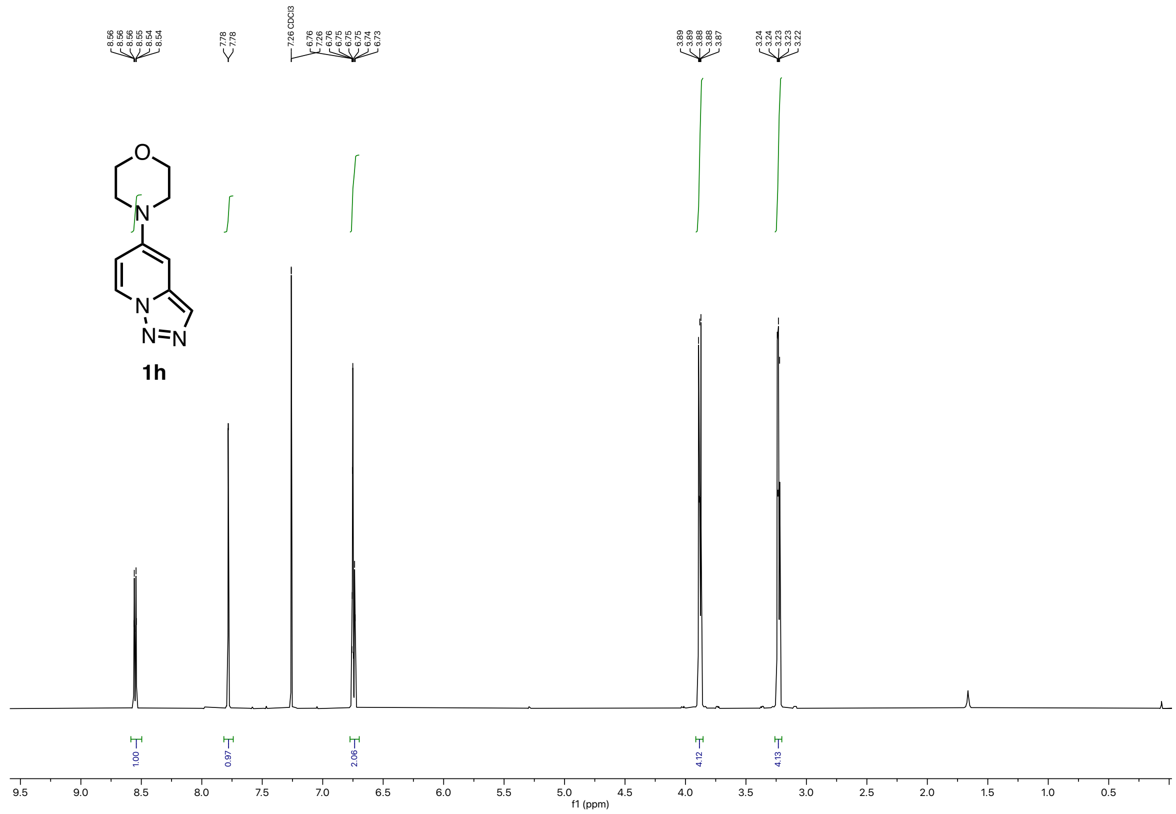

^{1}H spectrum (500 MHz, $CDCl_3$) of **1h**

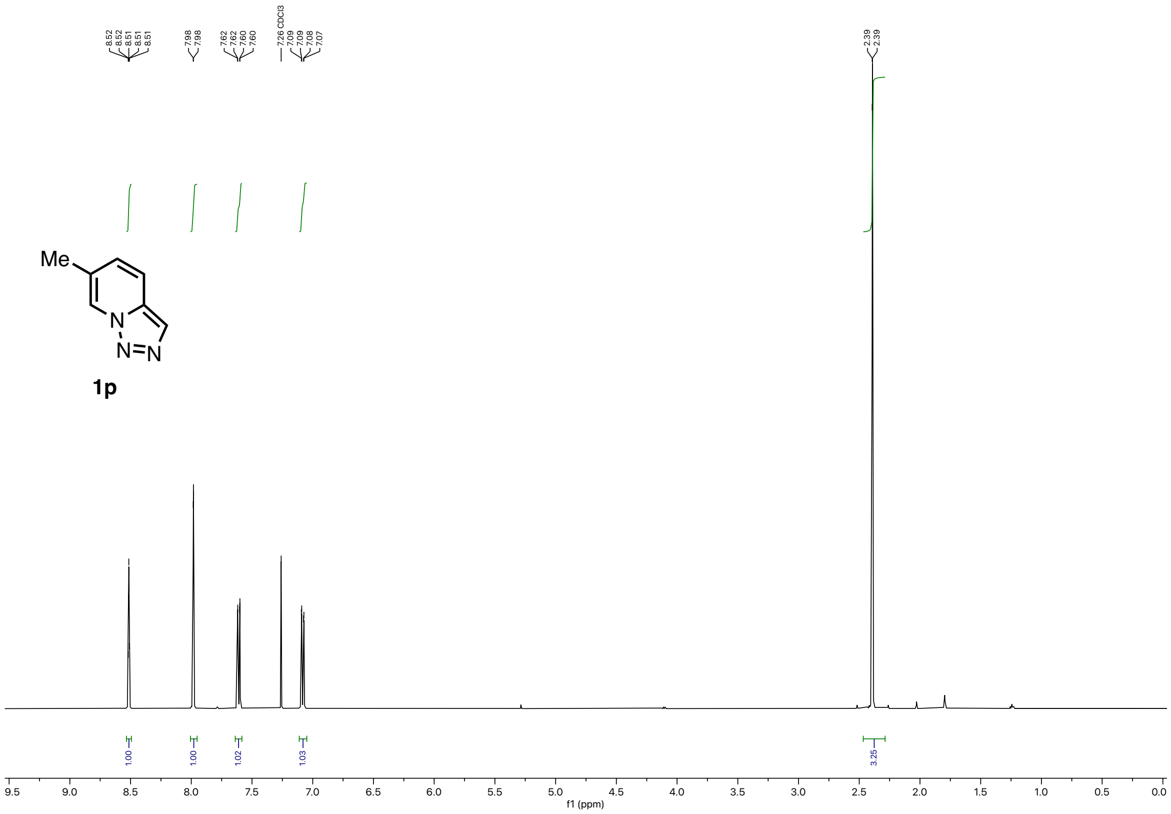

^{1}H spectrum (500 MHz, $CDCl_3$) of **1p**

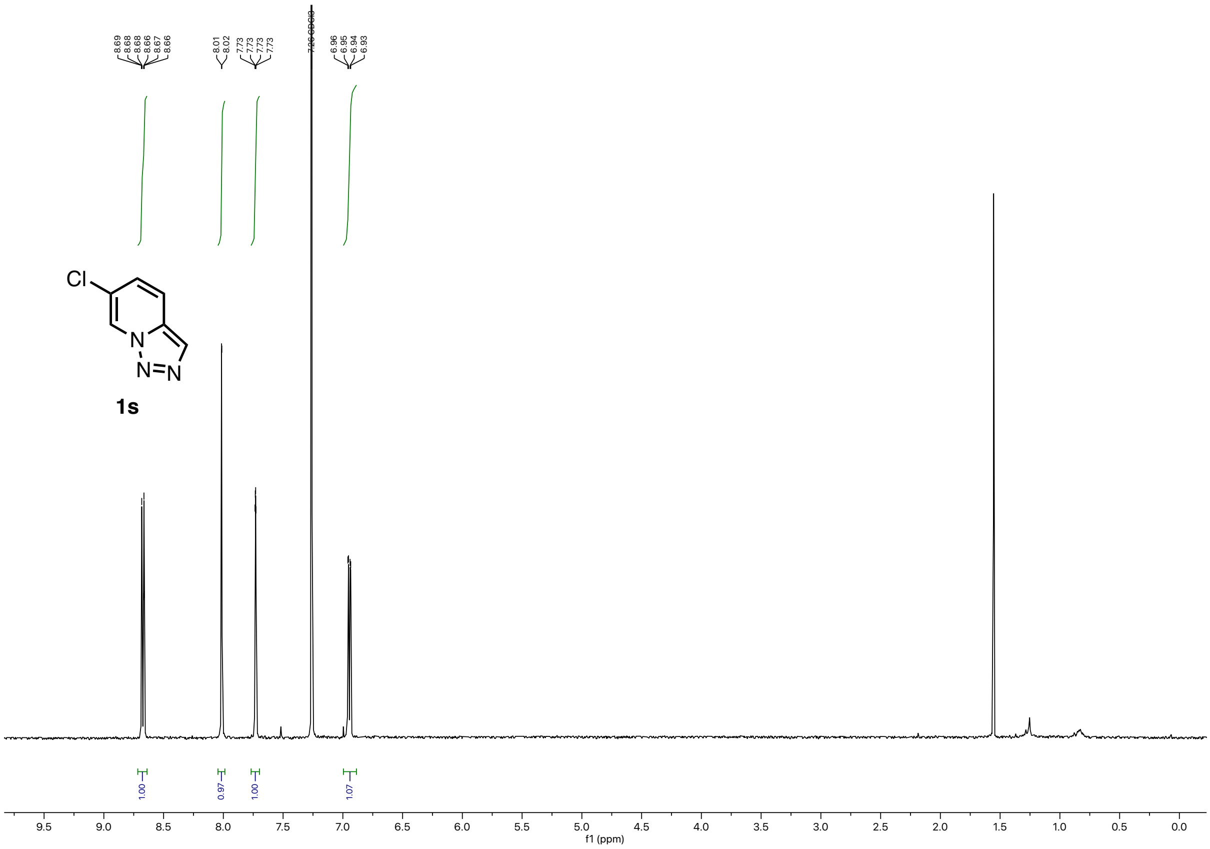

^{1}H spectrum (500 MHz, $CDCl_3$) of **1s**

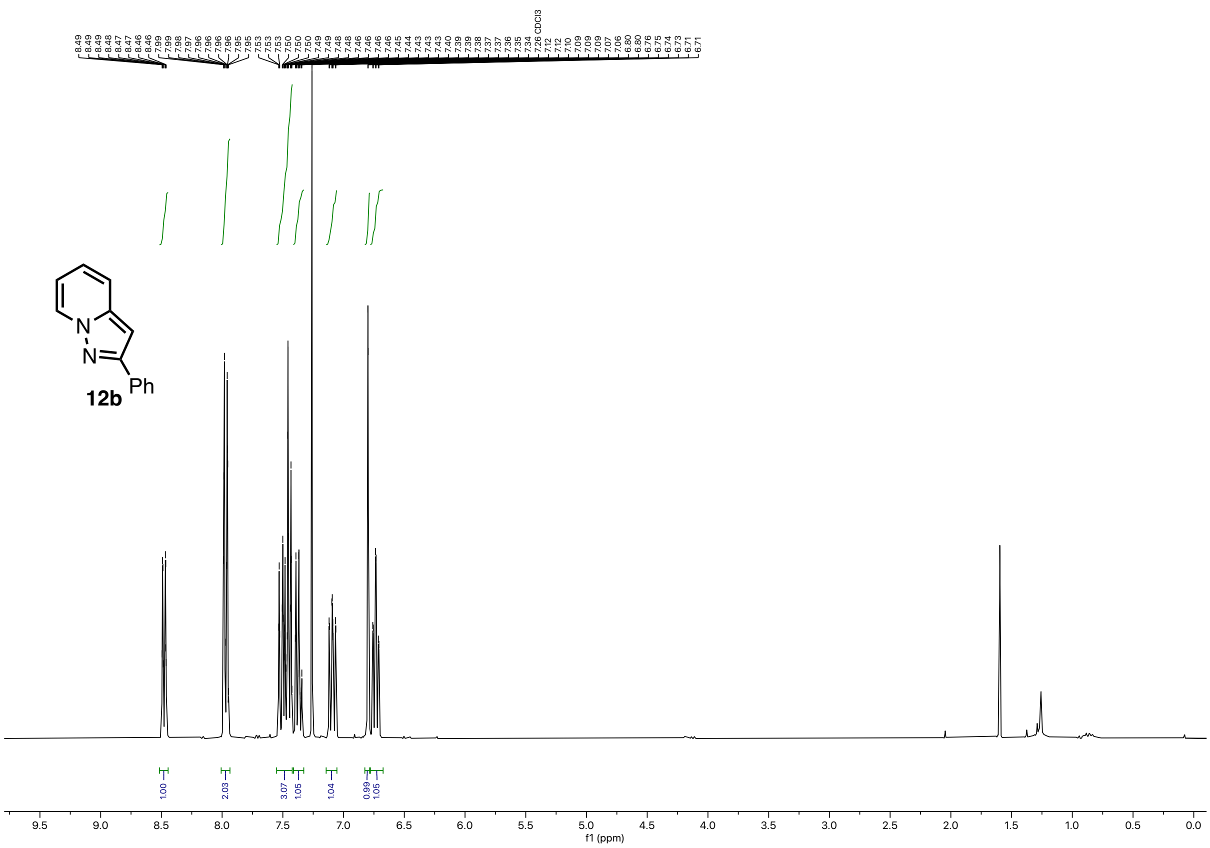

^1H spectrum (500 MHz, $CDCl_3$) of **12b**

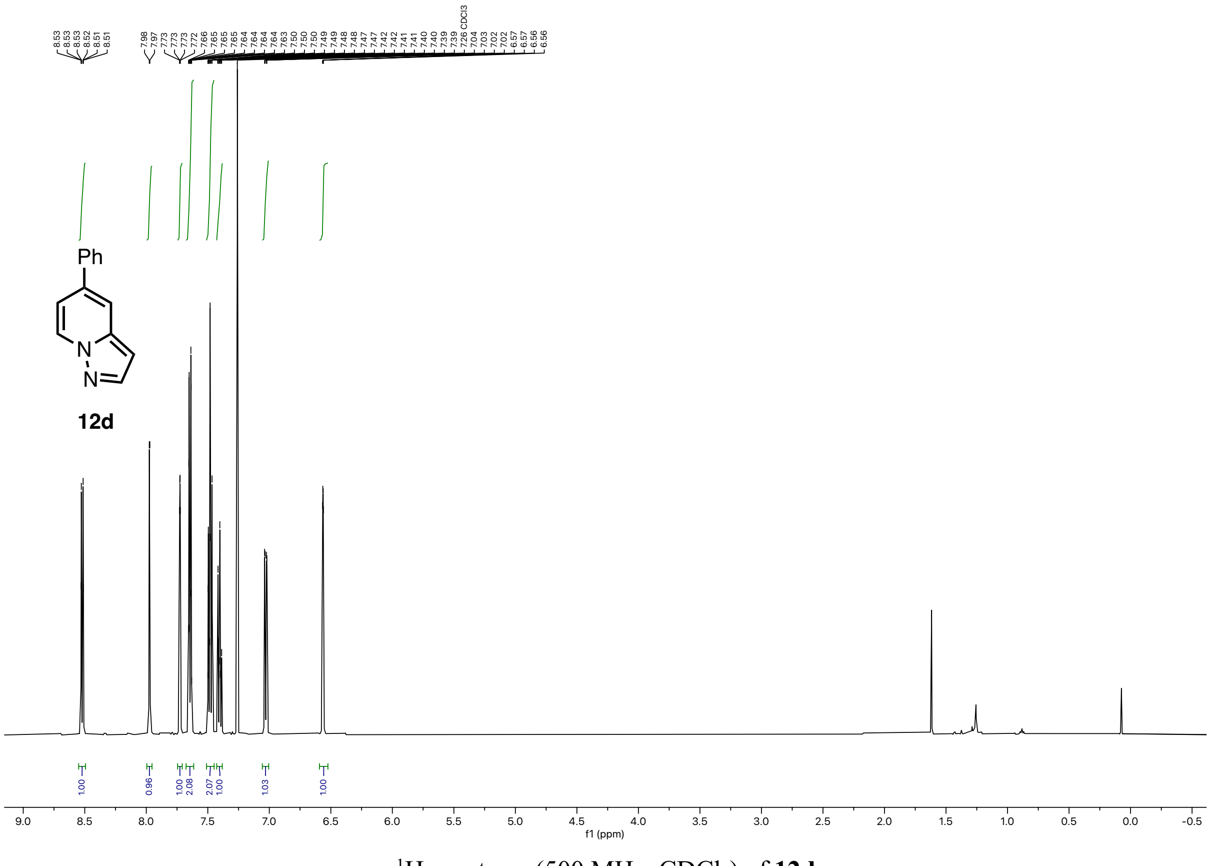

^1H spectrum (500 MHz, $CDCl_3$) of **12d**

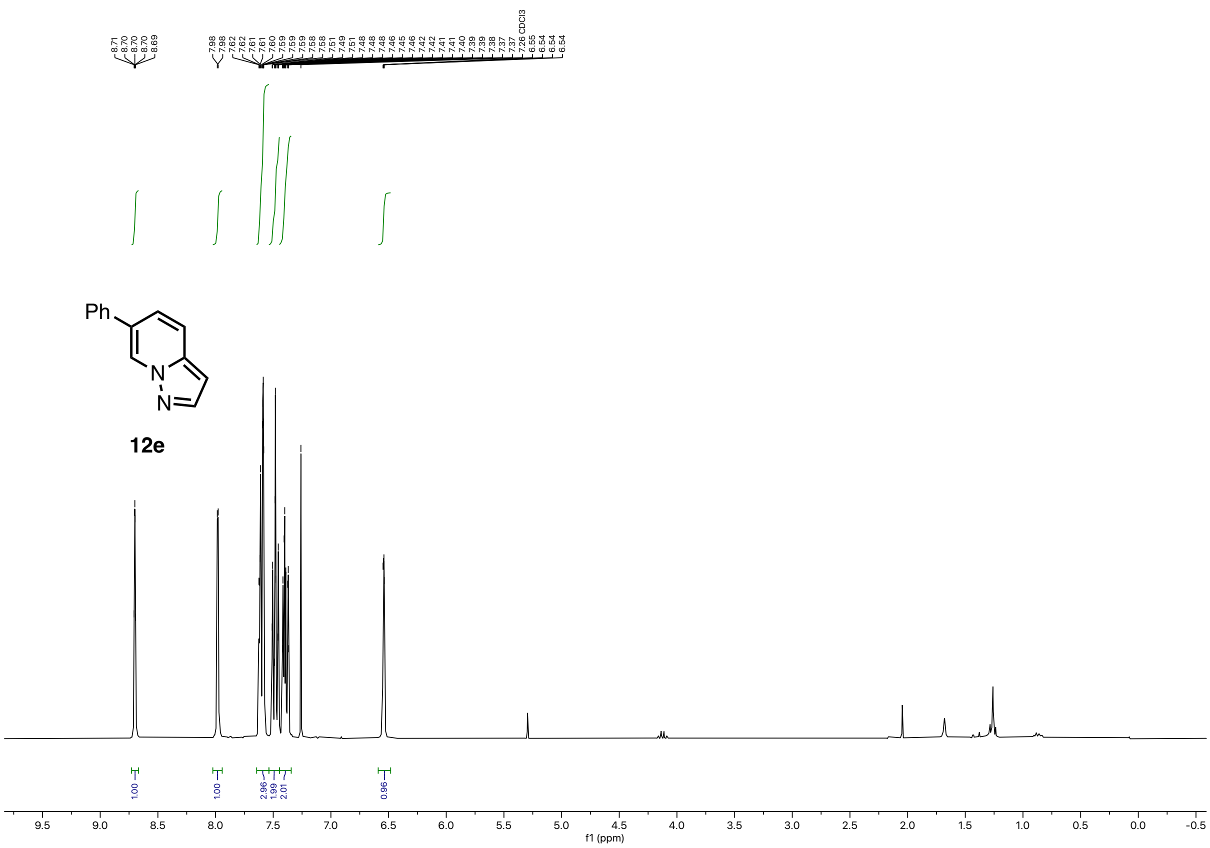

^{1}H spectrum (500 MHz, $CDCl_3$) of **12e**

NMR Spectra for Unknown Compounds

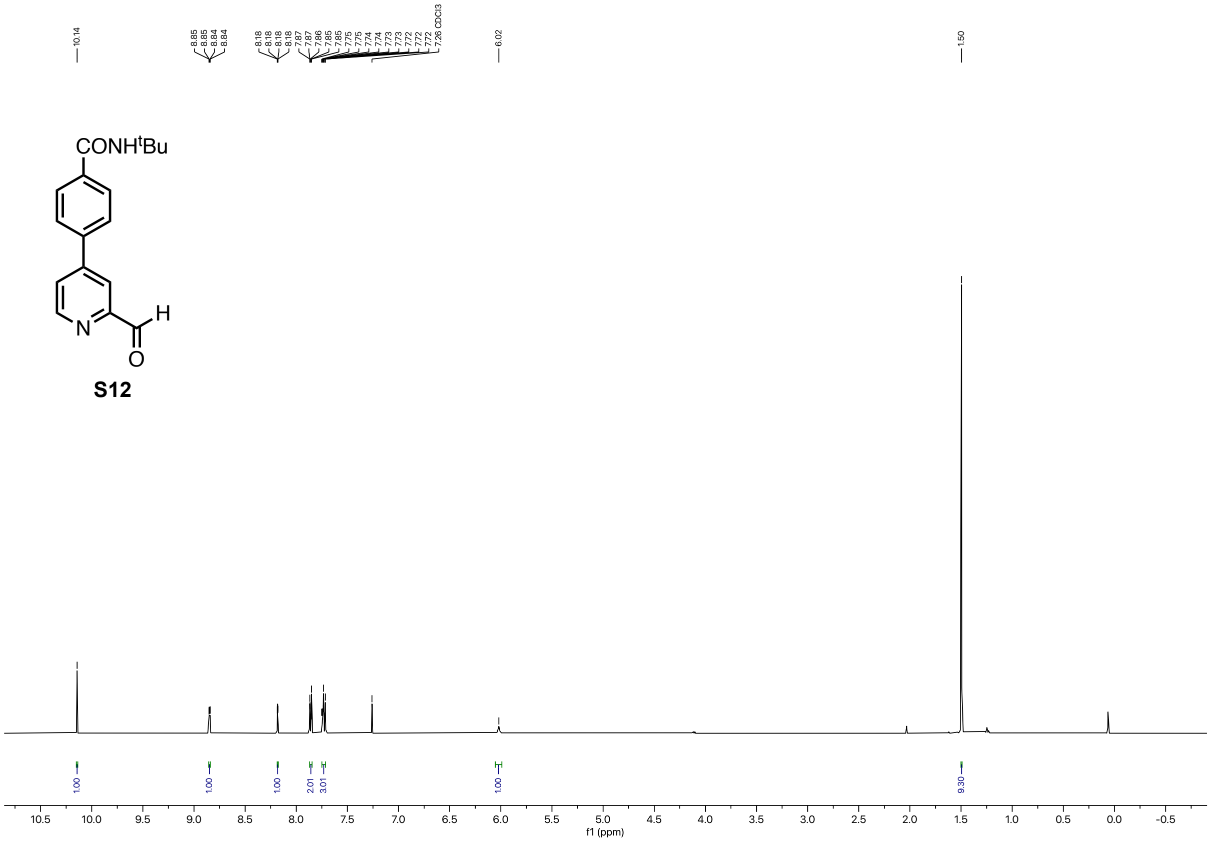

^{1}H spectrum (500 MHz, $CDCl_3$) of **S12**

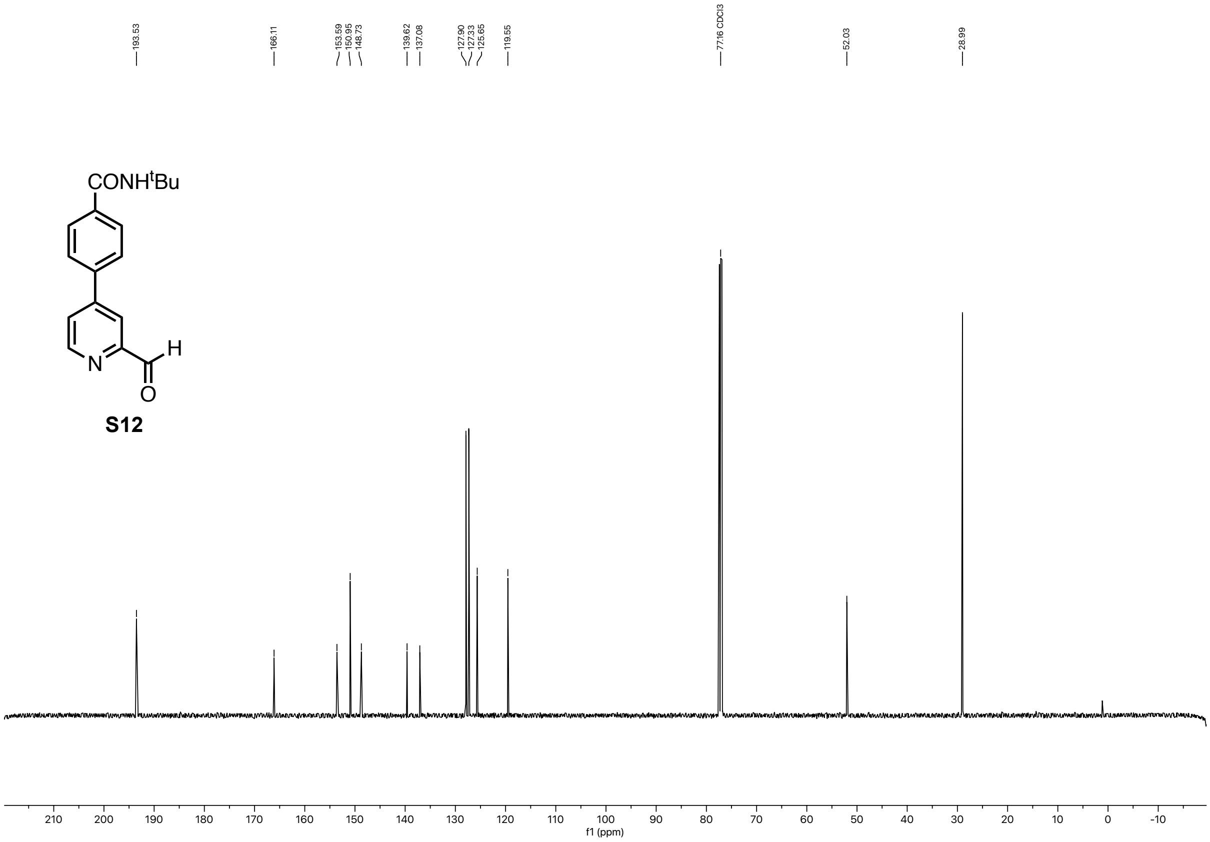

^{13}C spectrum (126 MHz, $CDCl_3$) of **S12**

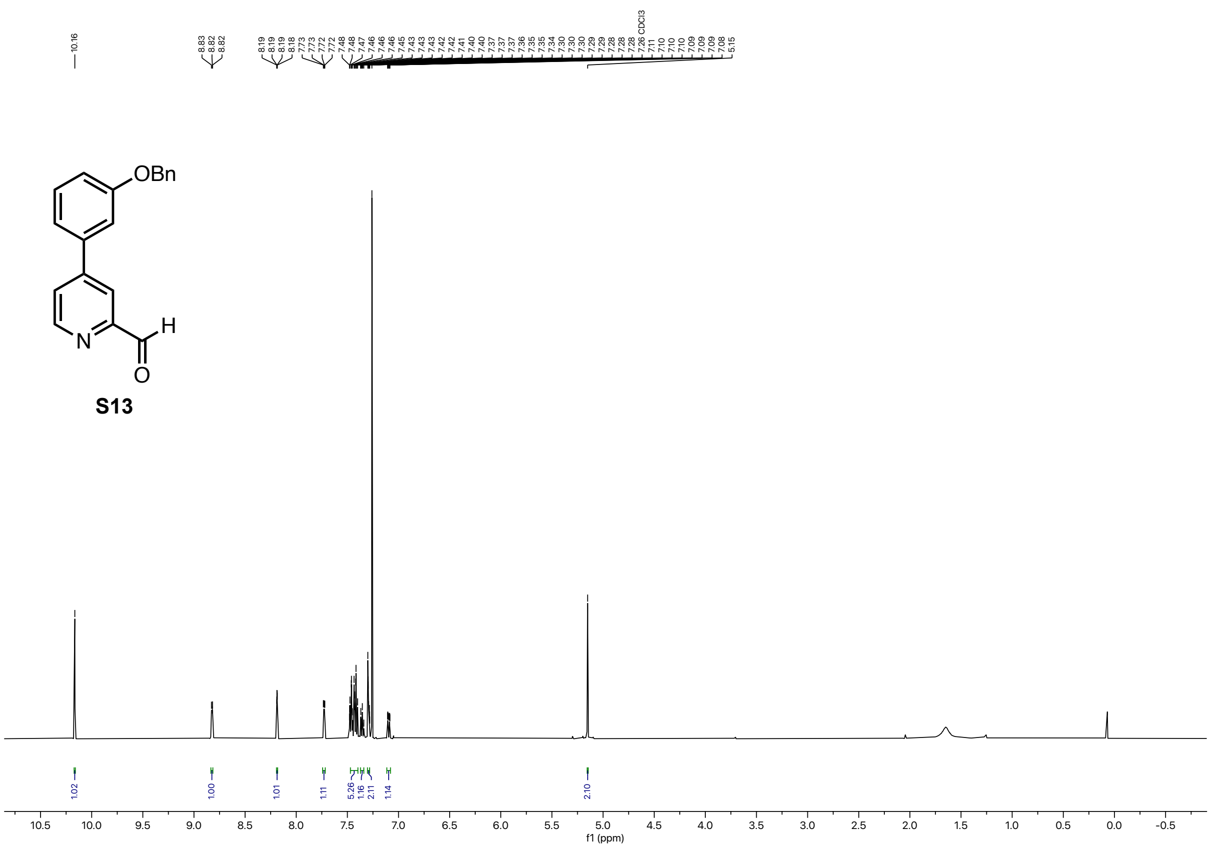

^{1}H spectrum (500 MHz, $CDCl_3$) of **S13**

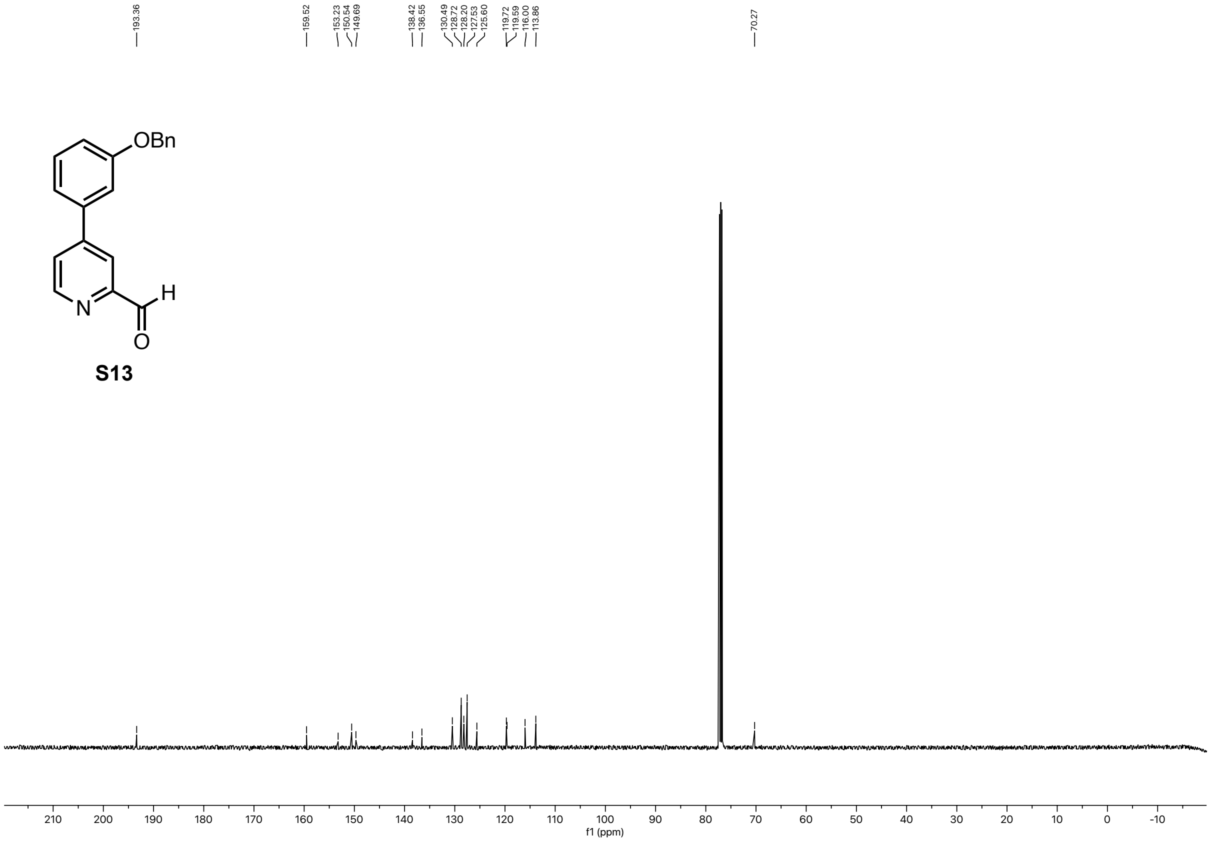

^{13}C spectrum (126 MHz, $CDCl_3$) of **S13**

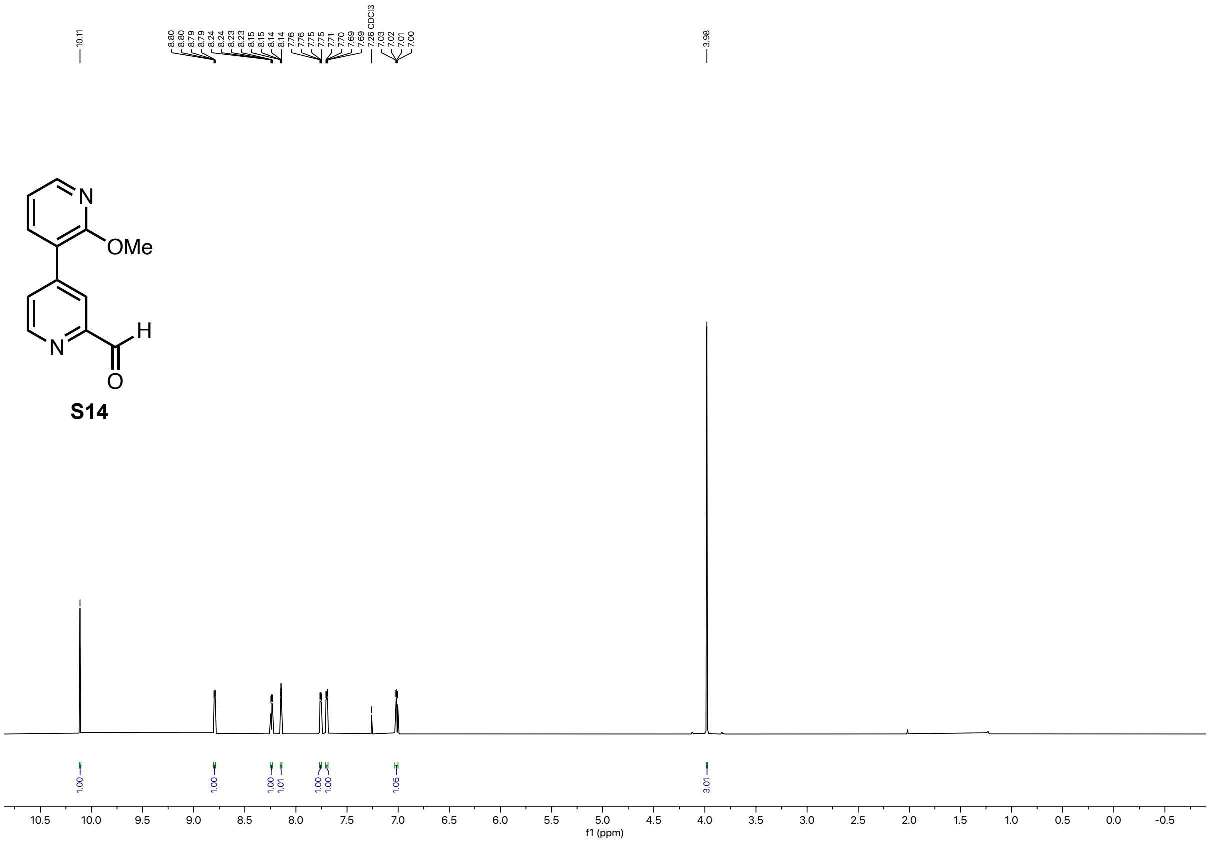

^{1}H spectrum (500 MHz, $CDCl_3$) of **S14**

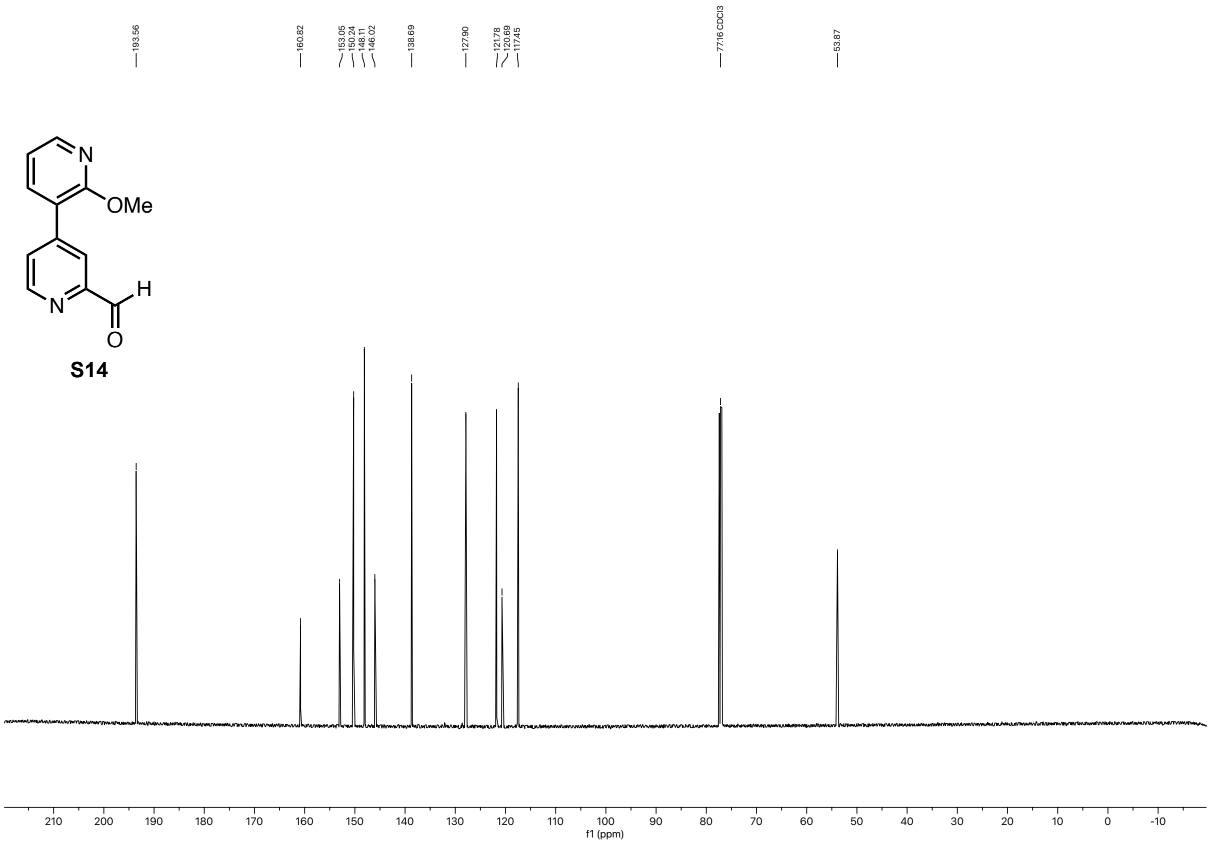

^{13}C spectrum (126 MHz, $CDCl_3$) of **S14**

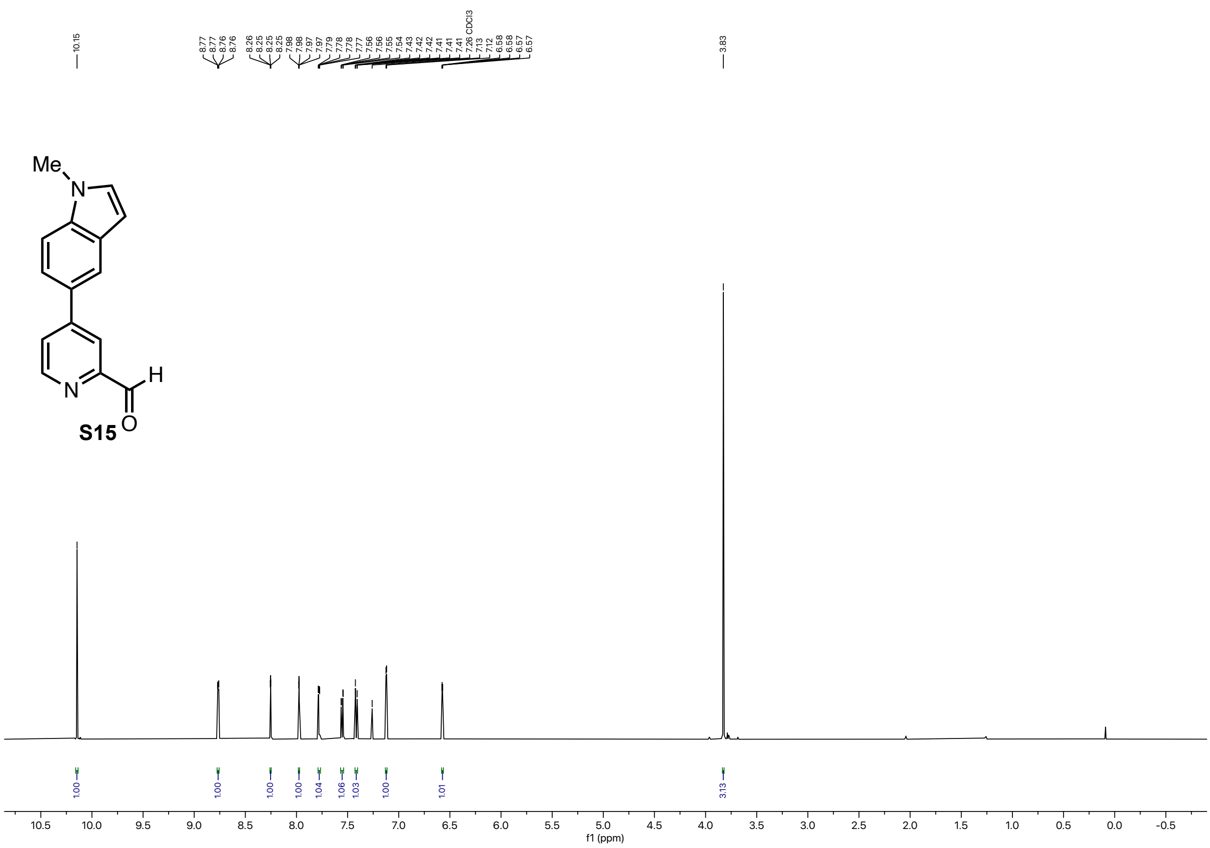

^{1}H spectrum (500 MHz, $CDCl_3$) of **S15**

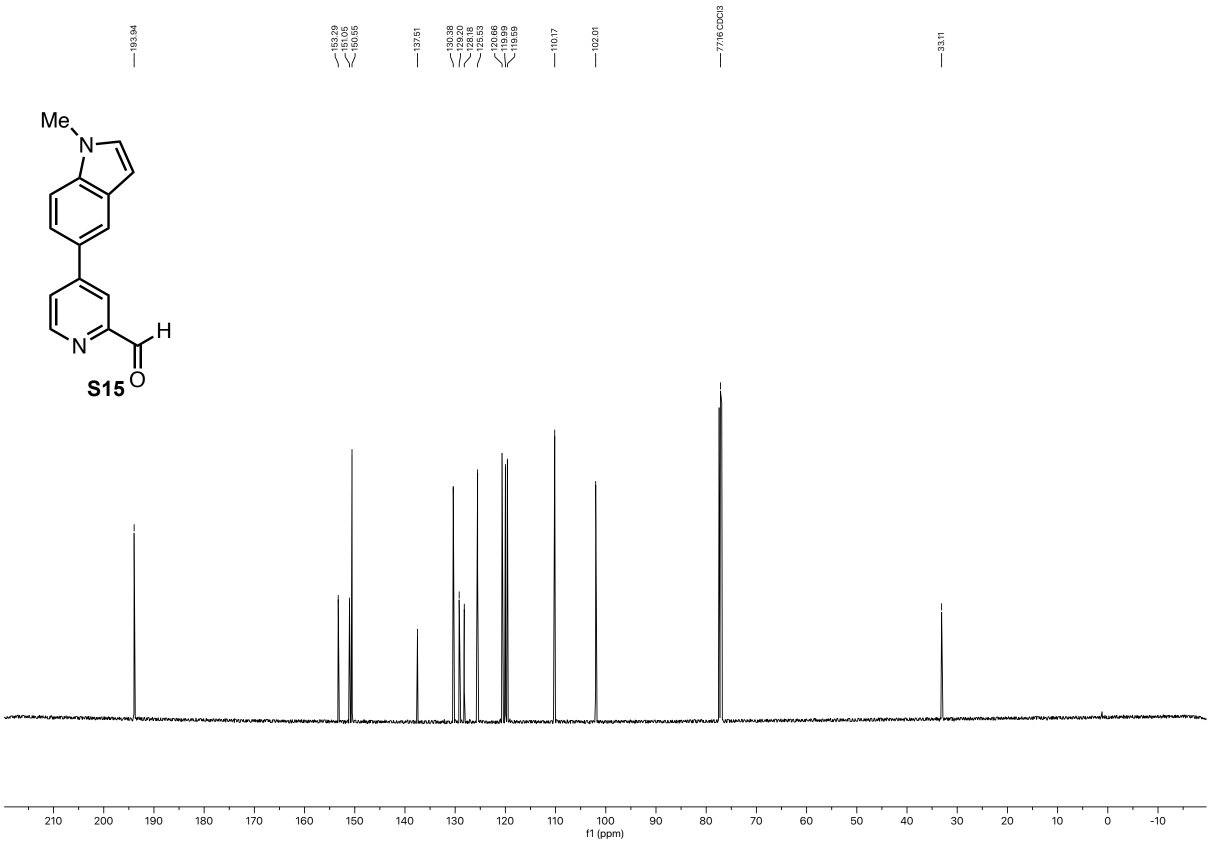

^{13}C spectrum (126 MHz, $CDCl_3$) of **S15**

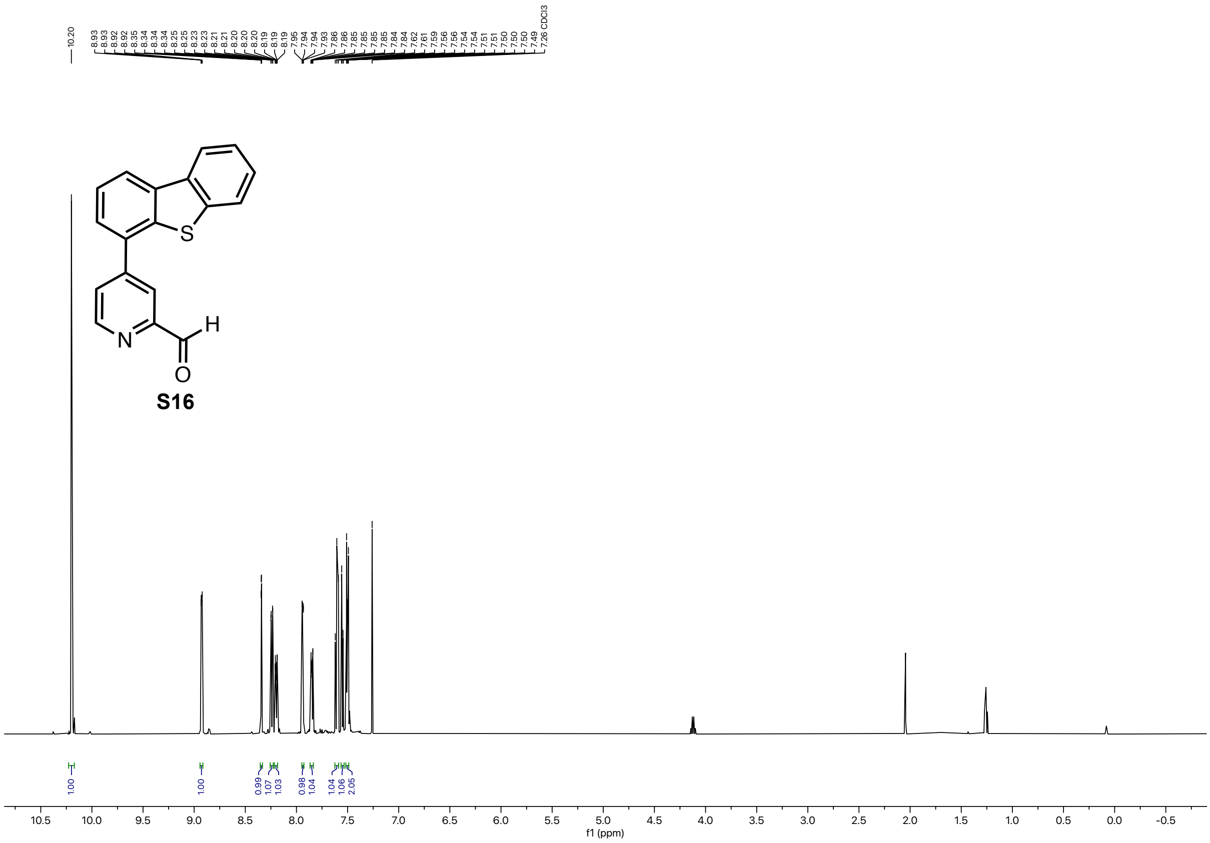

^{1}H spectrum (500 MHz, $CDCl_3$) of **S16**

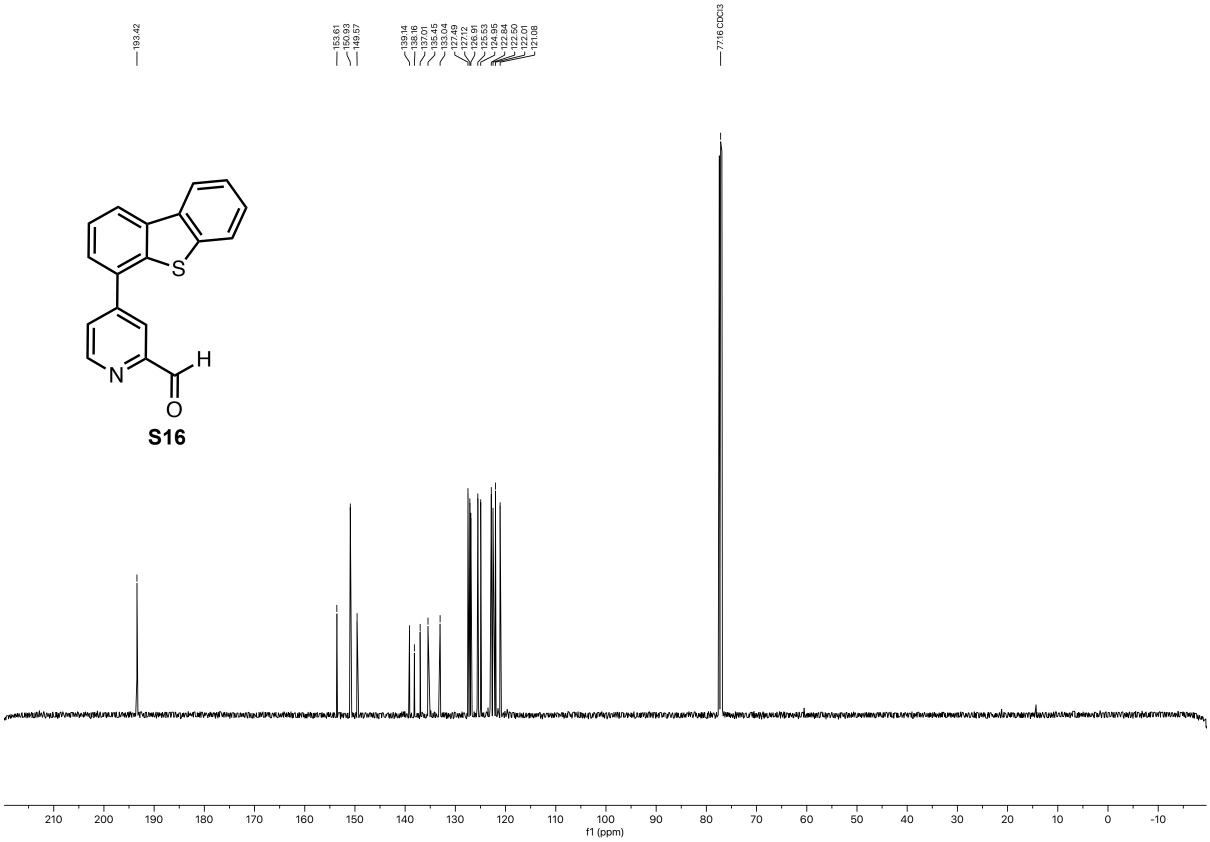

^{13}C spectrum (126 MHz, $CDCl_3$) of **S16**

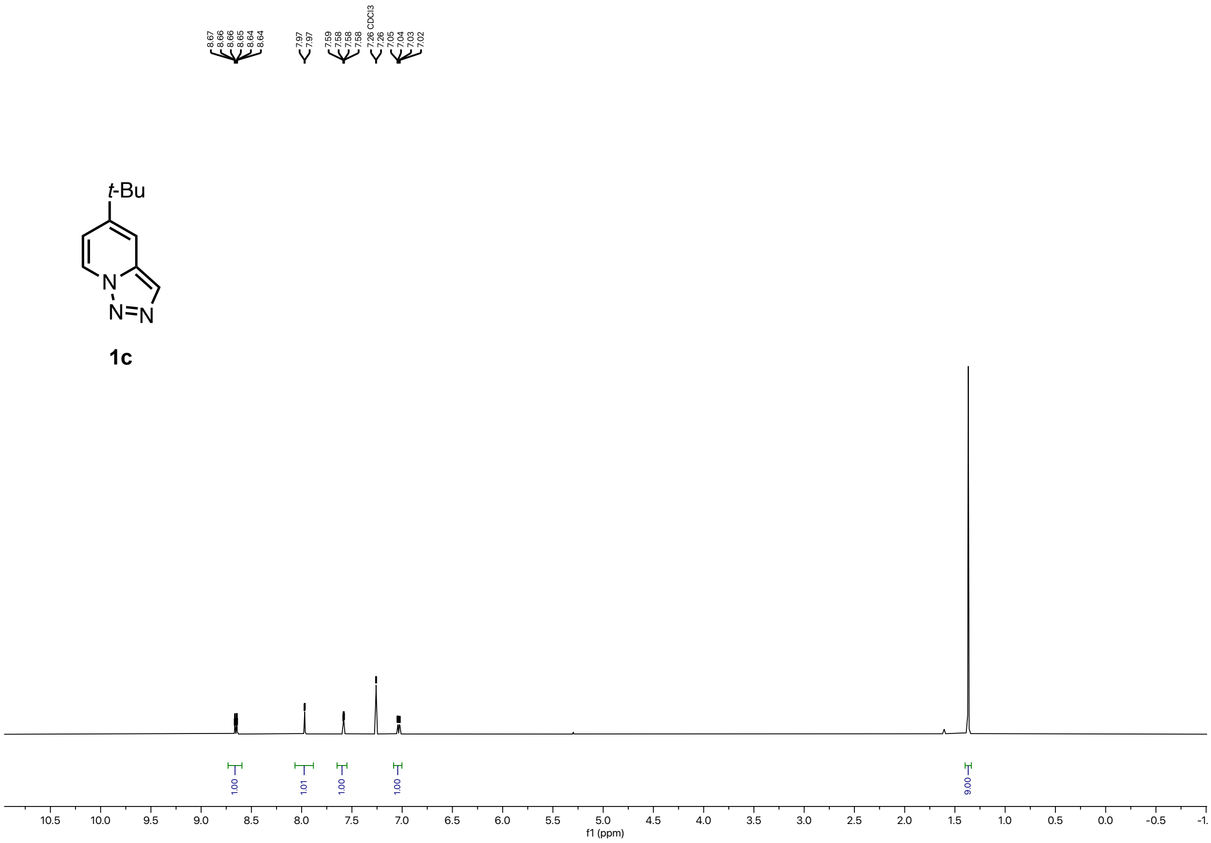

^{1}H spectrum (400 MHz, CD_3Cl) of **1c**

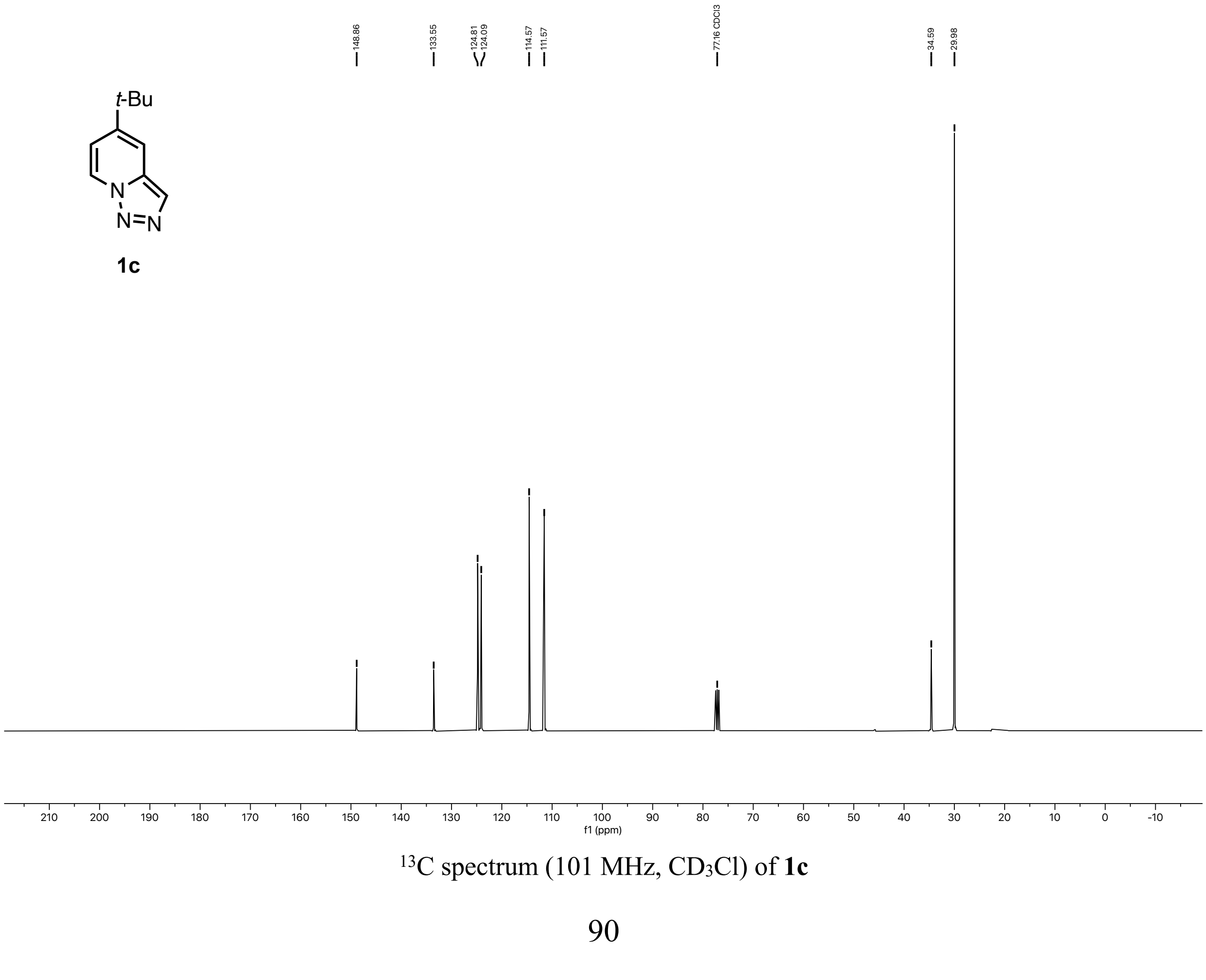

^{13}C spectrum (101 MHz, CD_3Cl) of **1c**

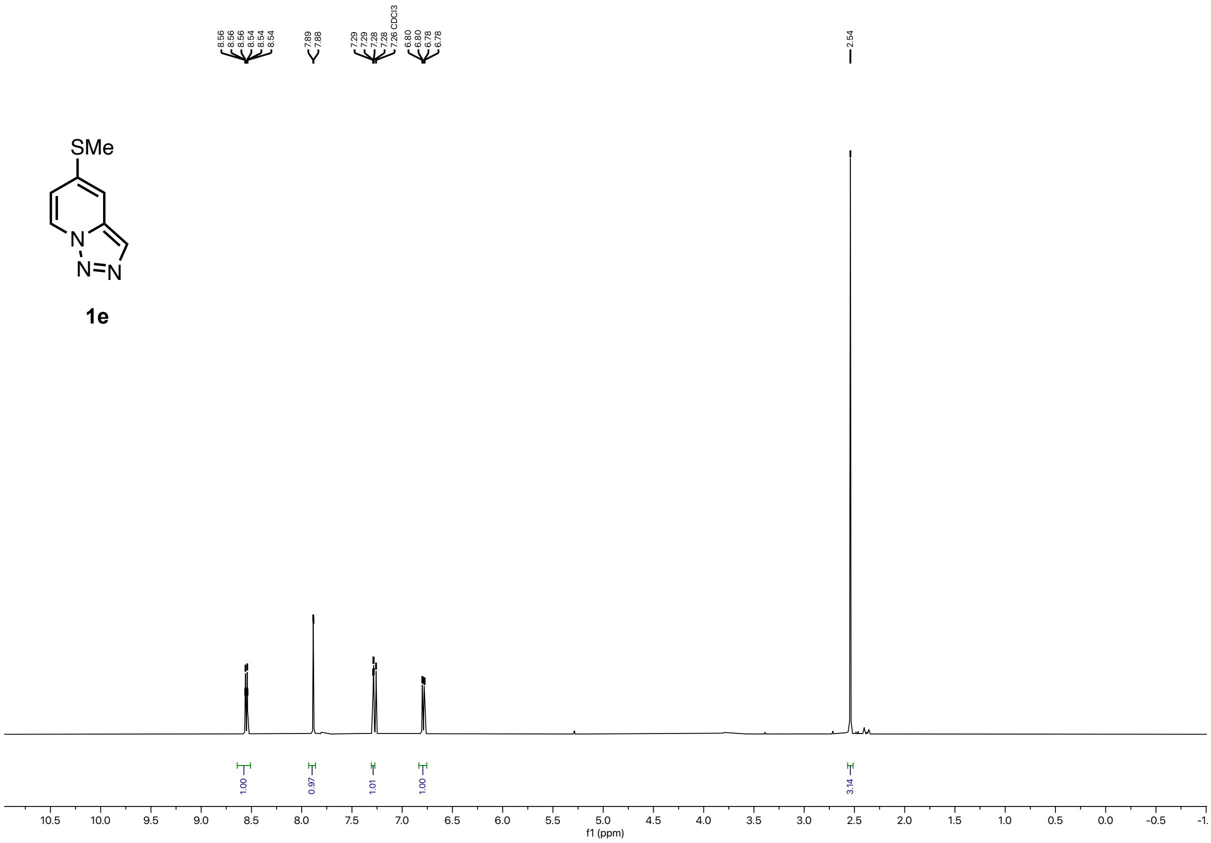

^{1}H spectrum (400 MHz, $CDCl_3$) of **1e**

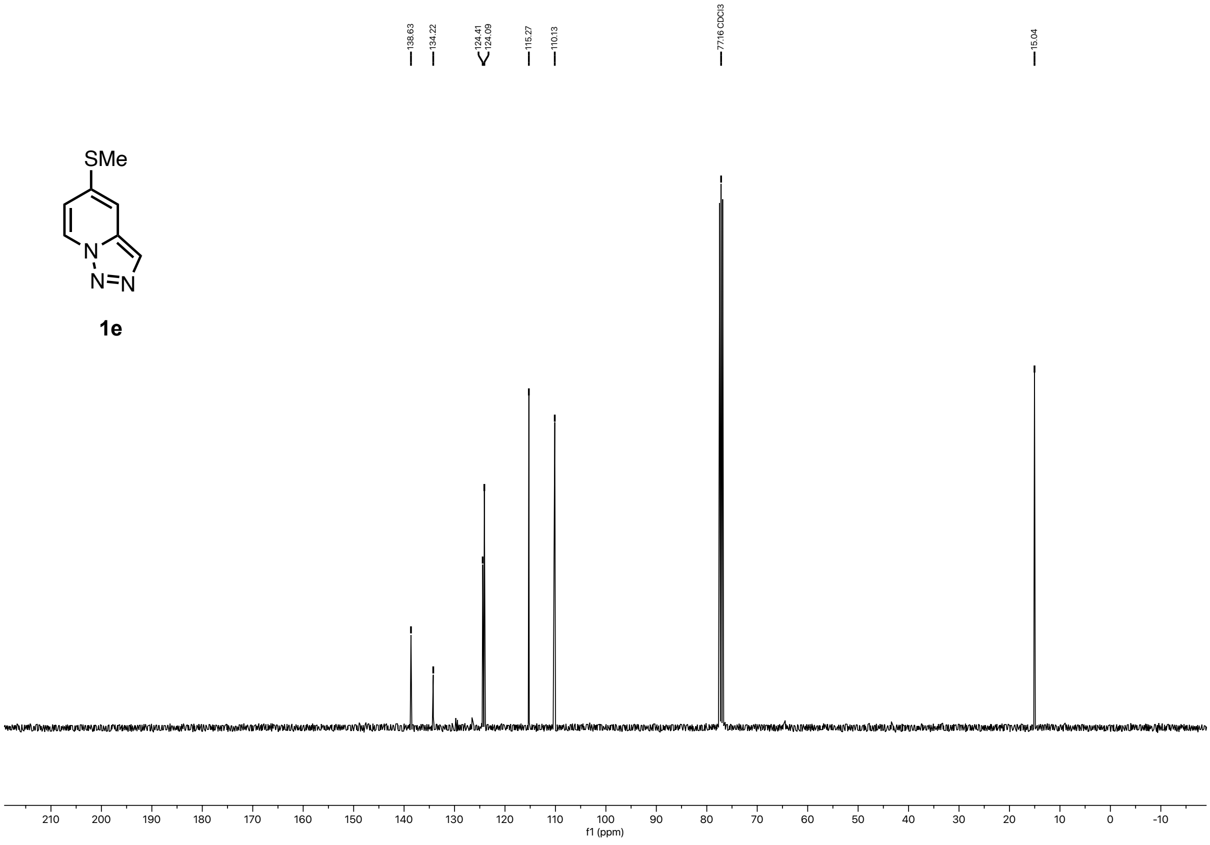

^{13}C spectrum (101 MHz, $CDCl_3$) of **1e**

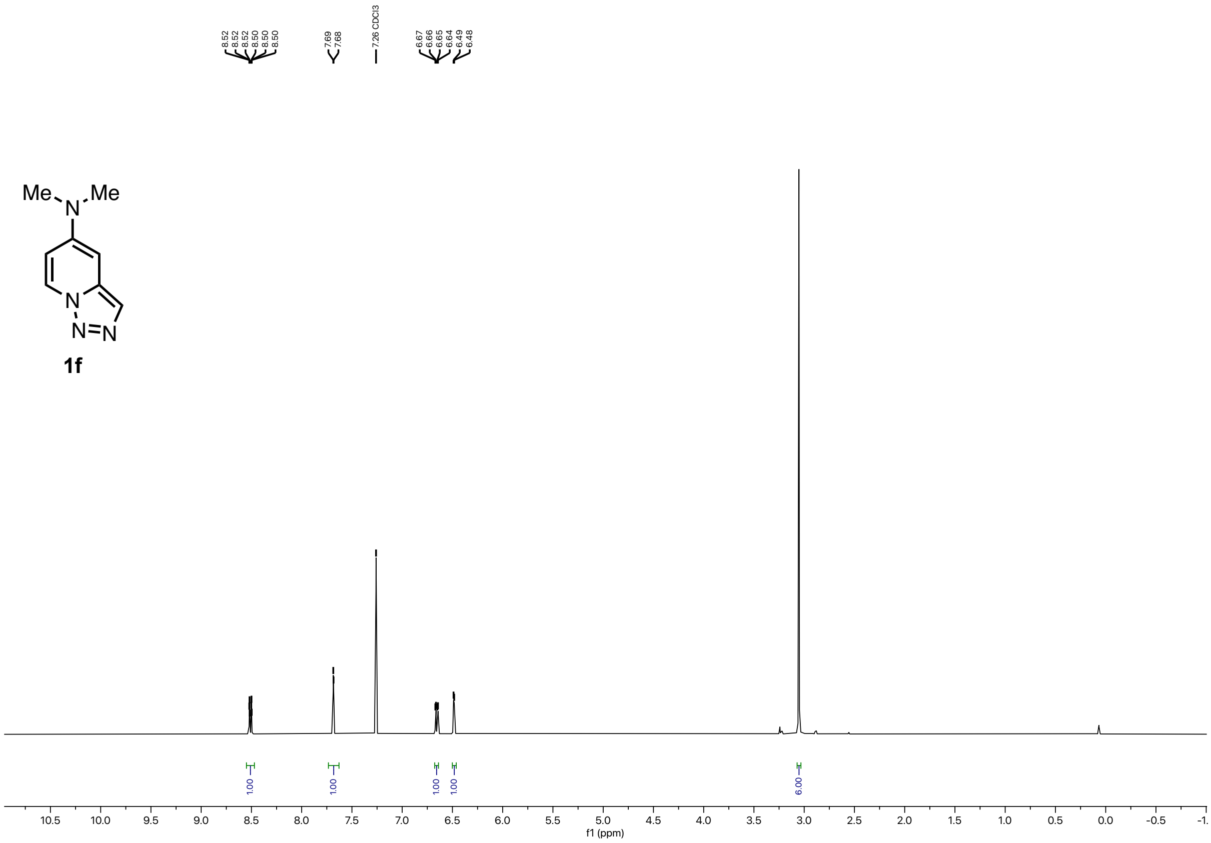

^{1}H spectrum (400 MHz, $CDCl_3$) of **1f**

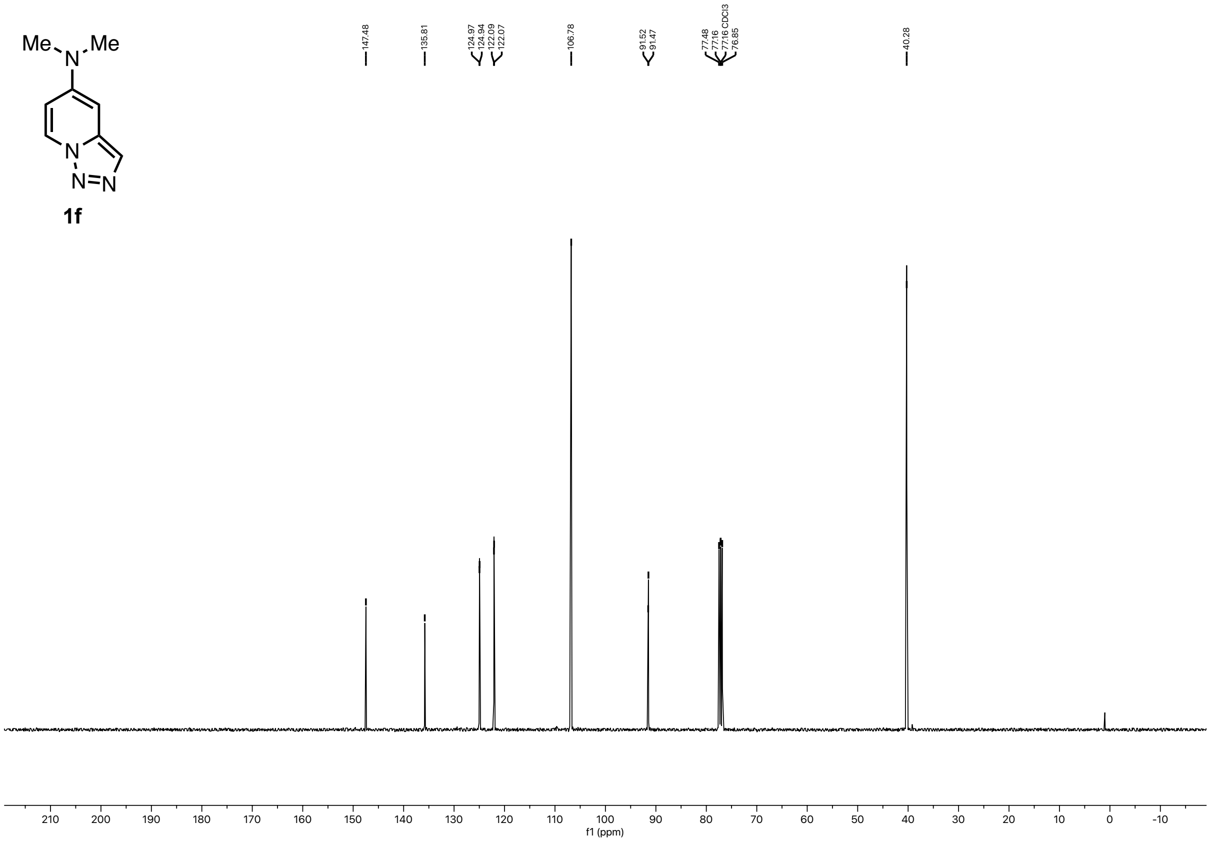

^{13}C spectrum (101 MHz, $CDCl_3$) of **1f**

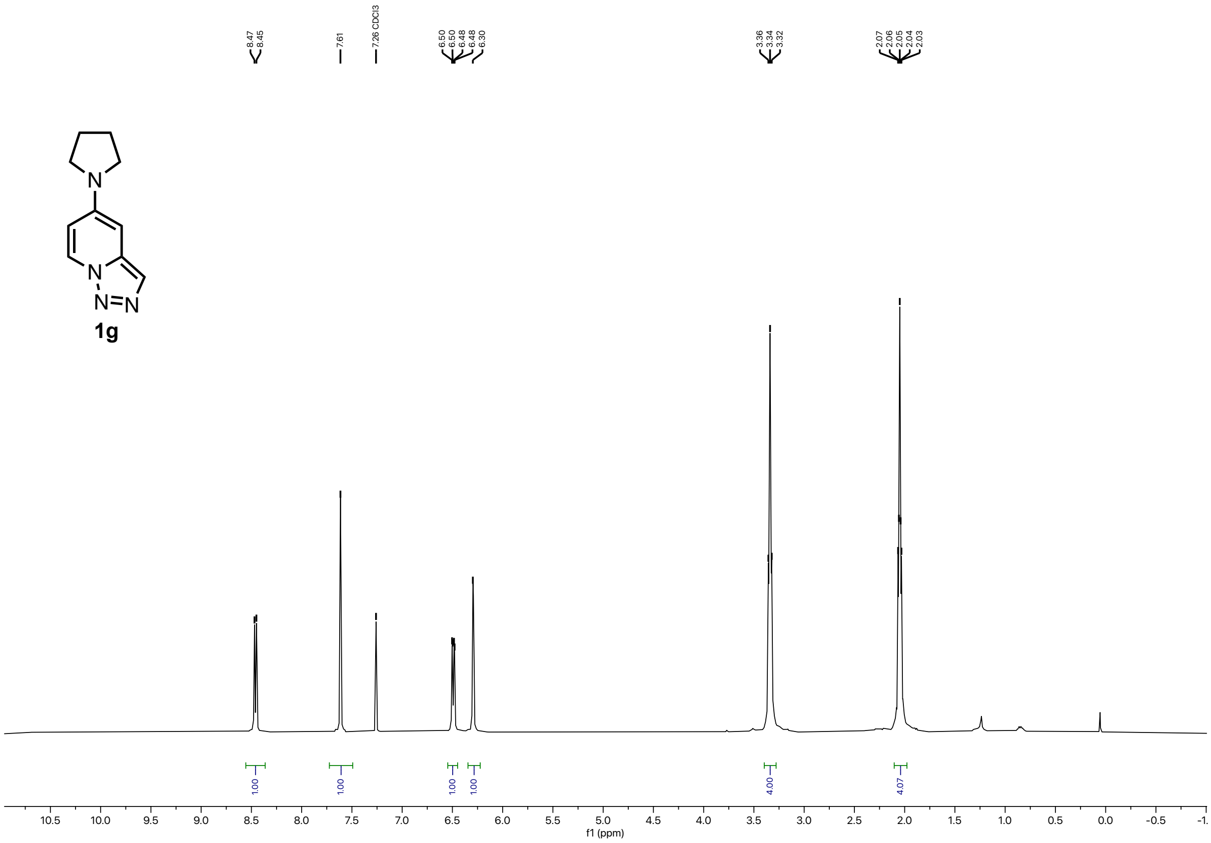

^{1}H spectrum (500 MHz, $CDCl_3$) of **1g**

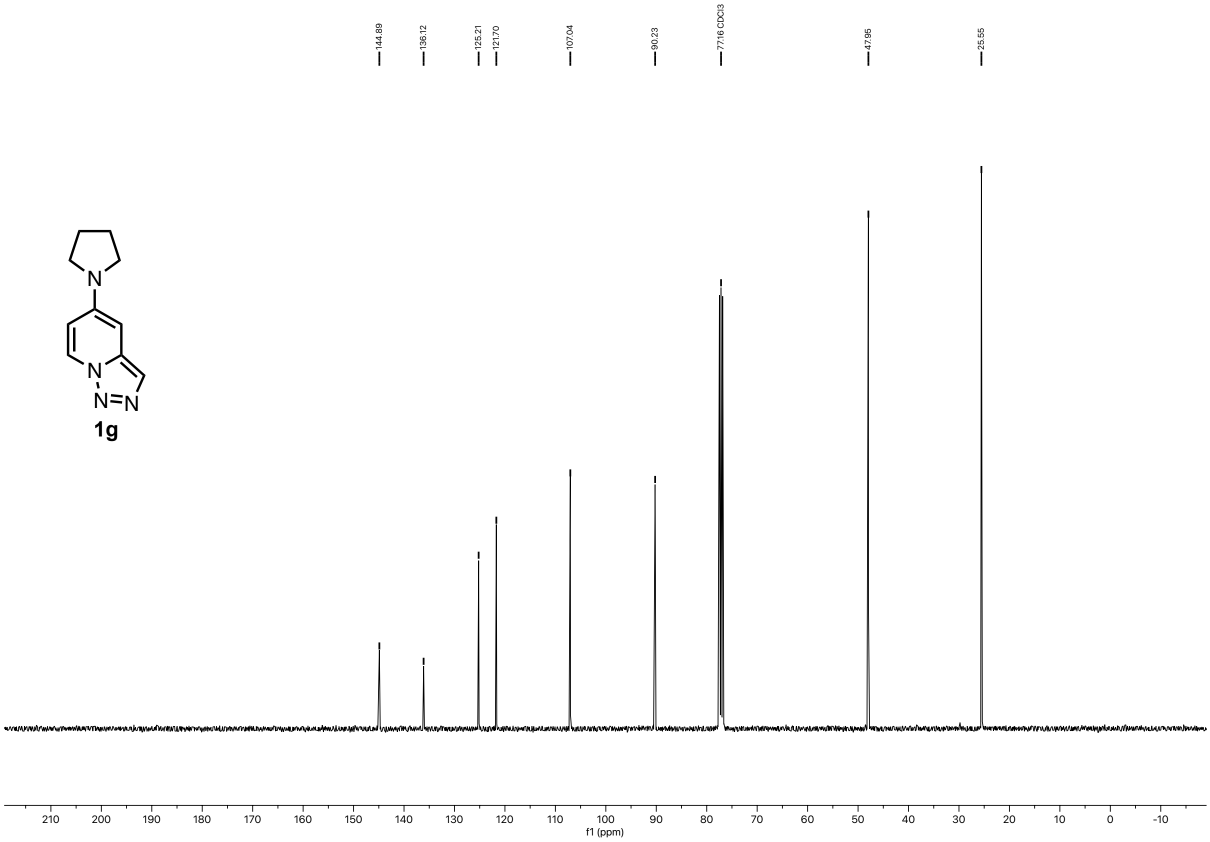

^{13}C spectrum (101 MHz, $CDCl_3$) of **1g**

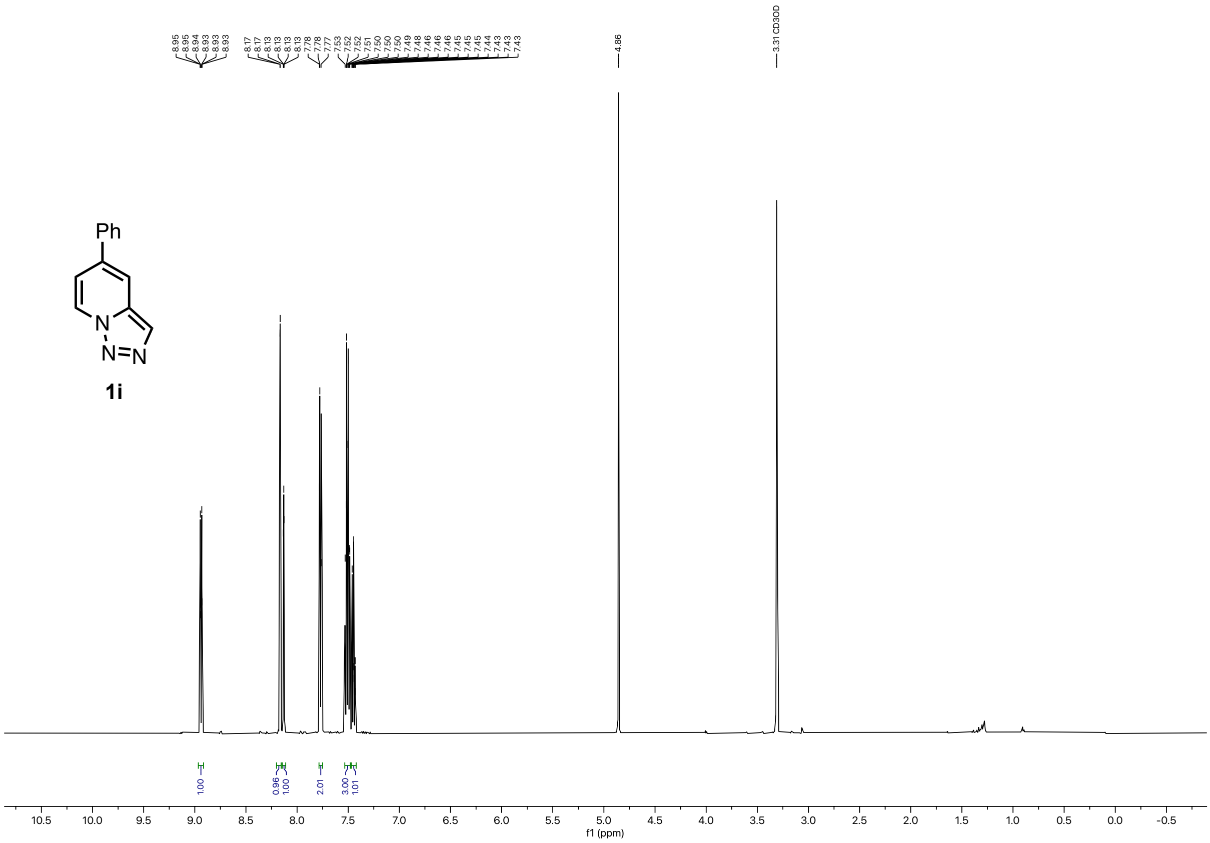

^{1}H spectrum (500 MHz, CD_3OD) of **1i**

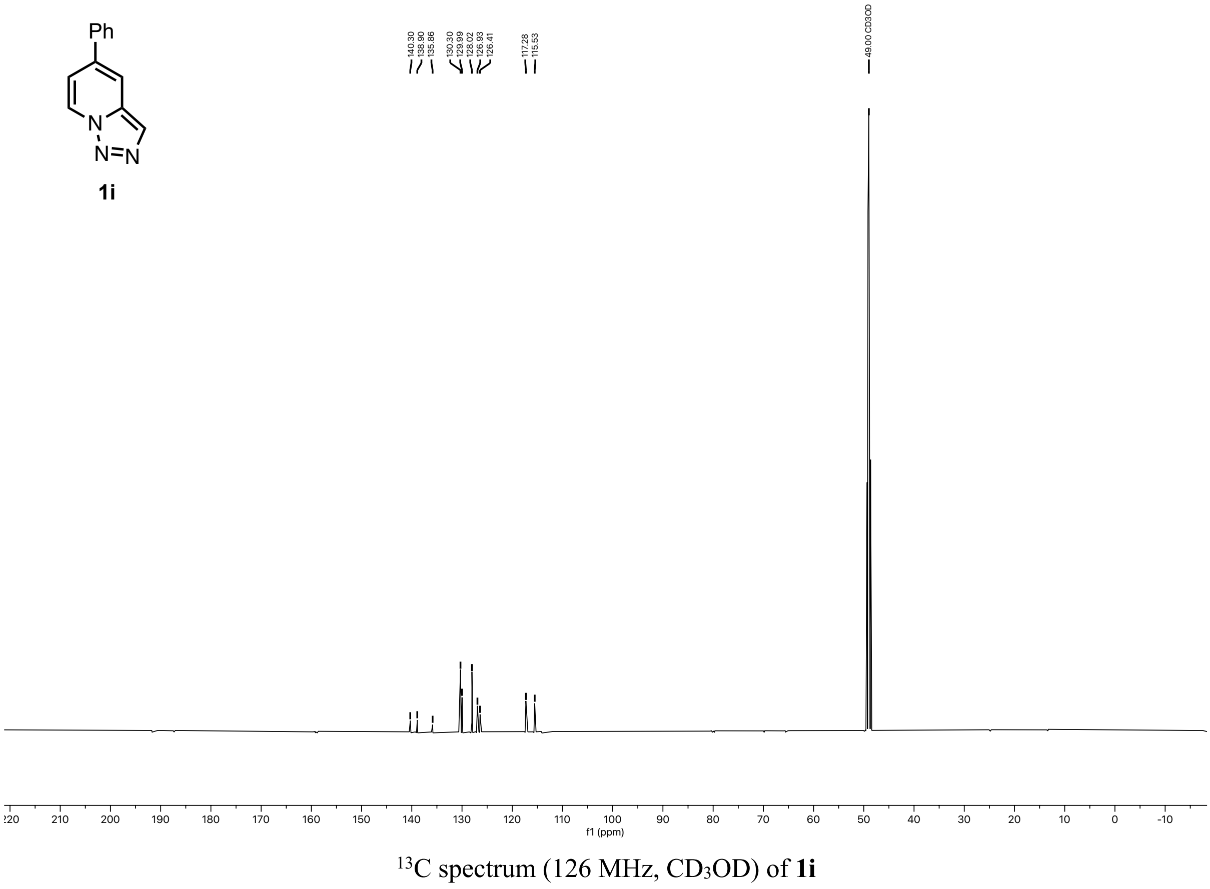

^{13}C spectrum (126 MHz, CD_3OD) of **1i**

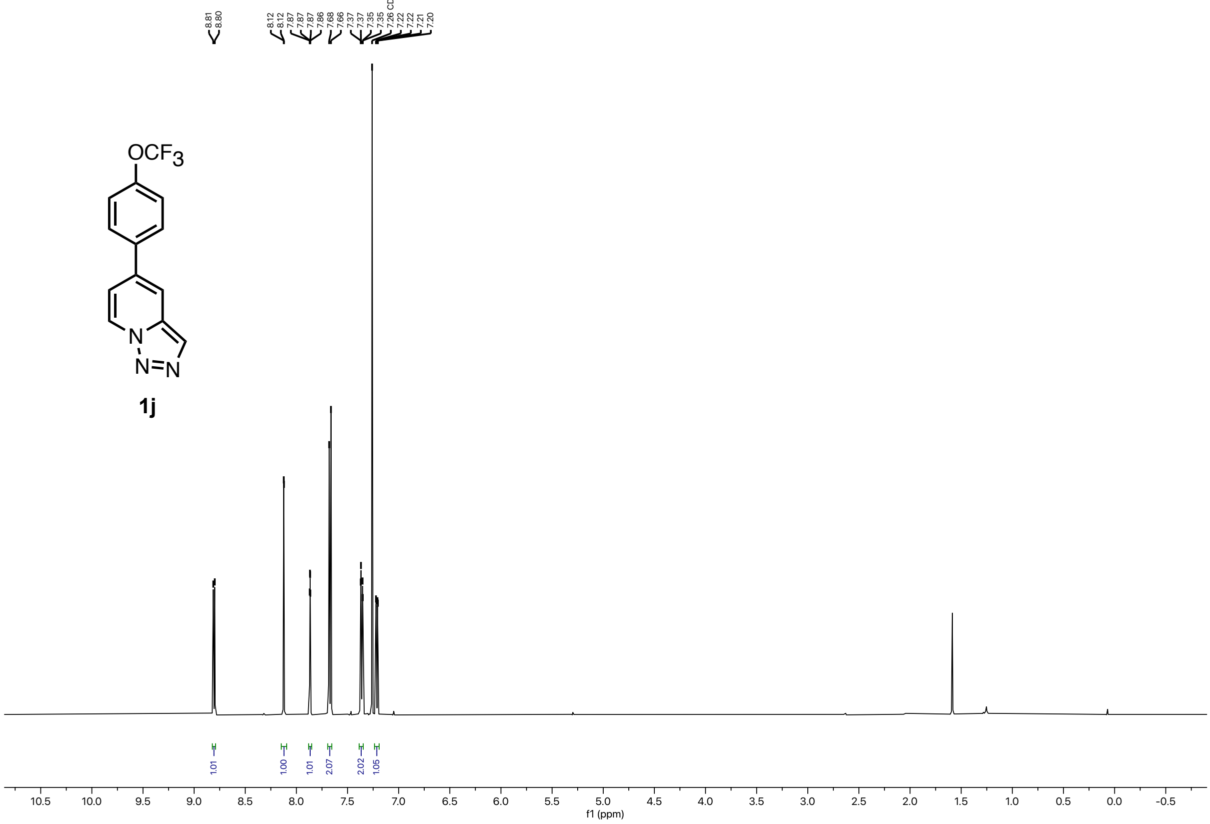

^{1}H spectrum (500 MHz, CD3Cl) of **1j**

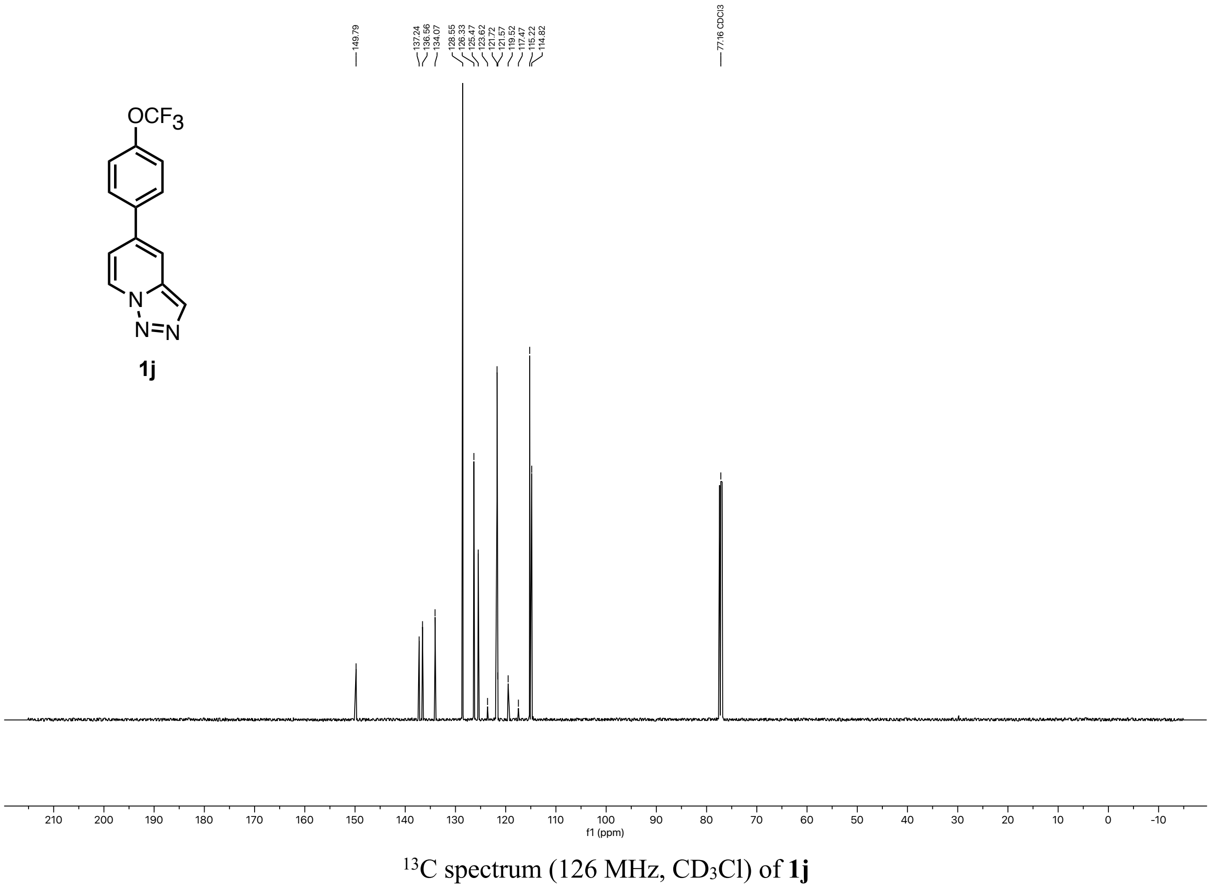

^{13}C spectrum (126 MHz, CD3Cl) of **1j**

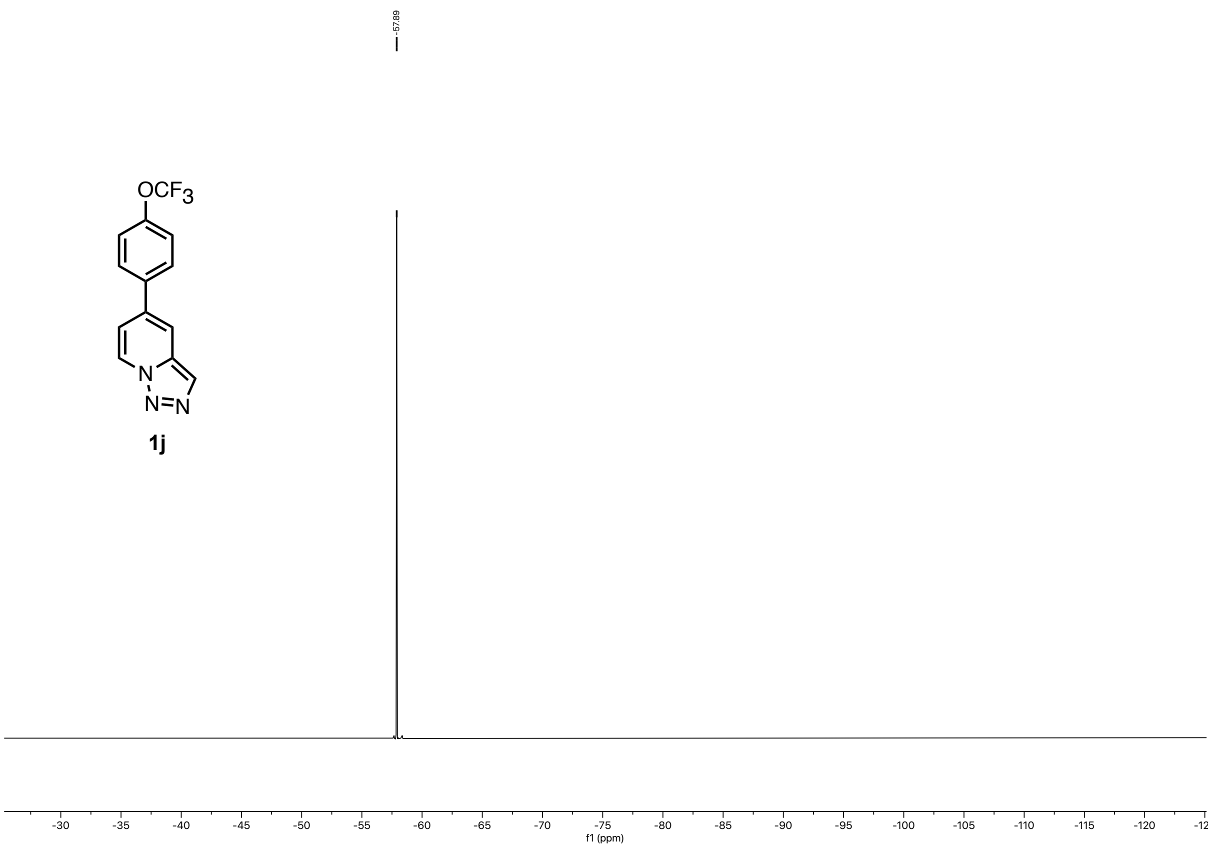

^{19}F spectrum (376 MHz, CDCl3) of **1j**

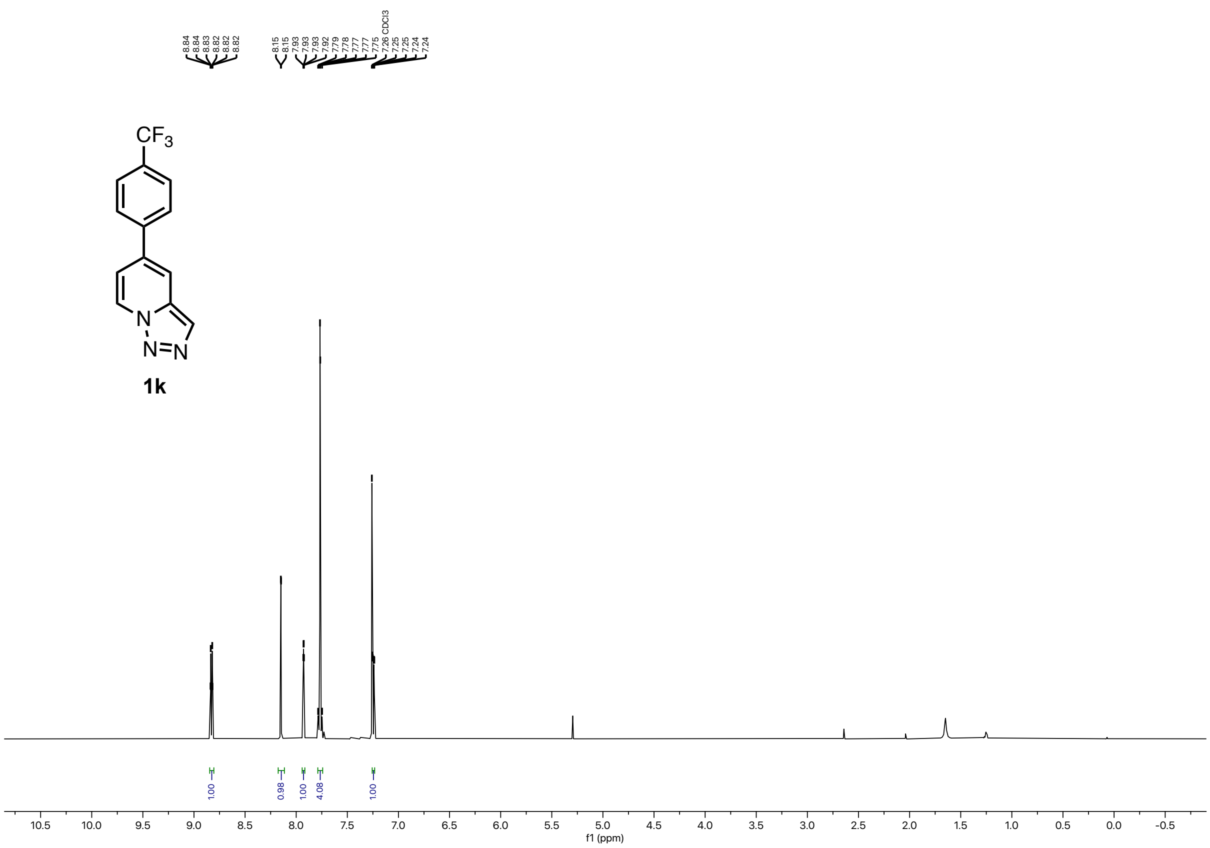

^{1}H spectrum (500 MHz, CD_3Cl) of **1k**

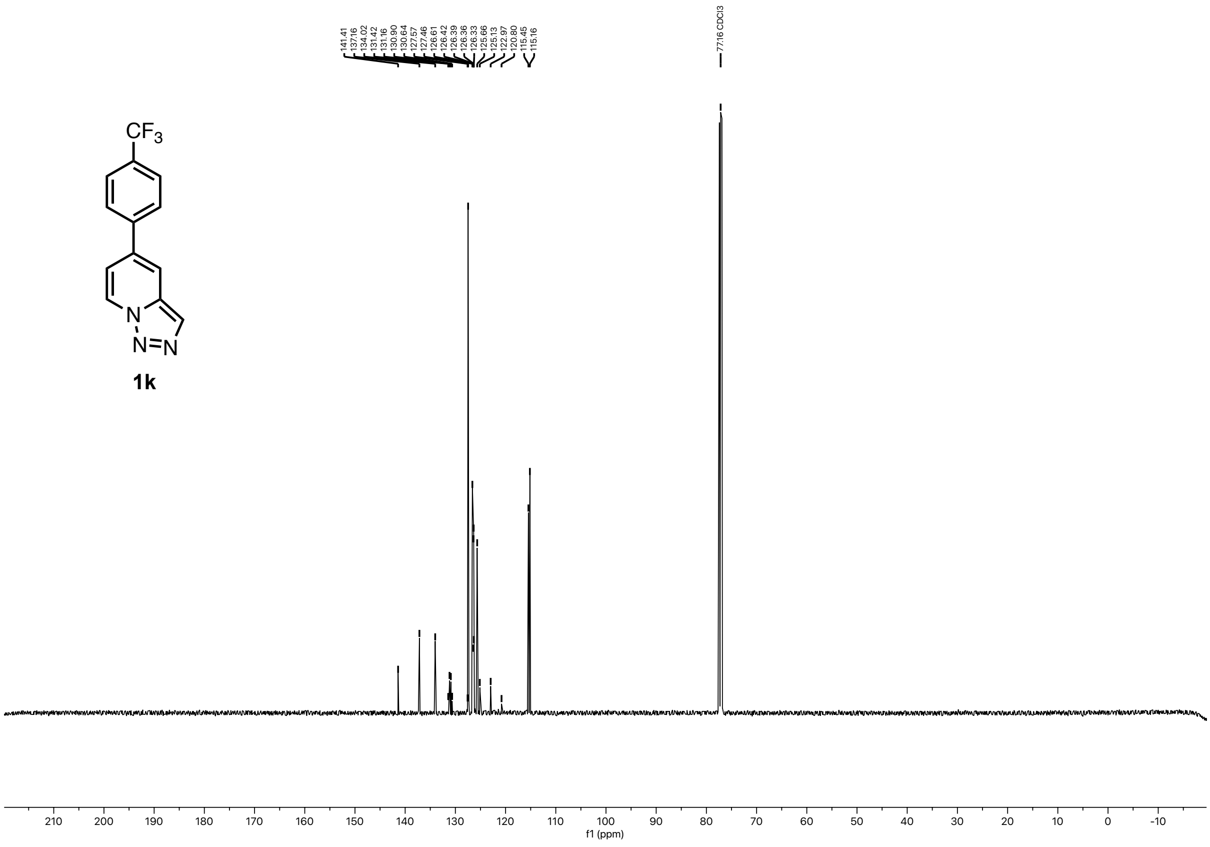

^{13}C spectrum (126 MHz, CD_3Cl) of **1k**

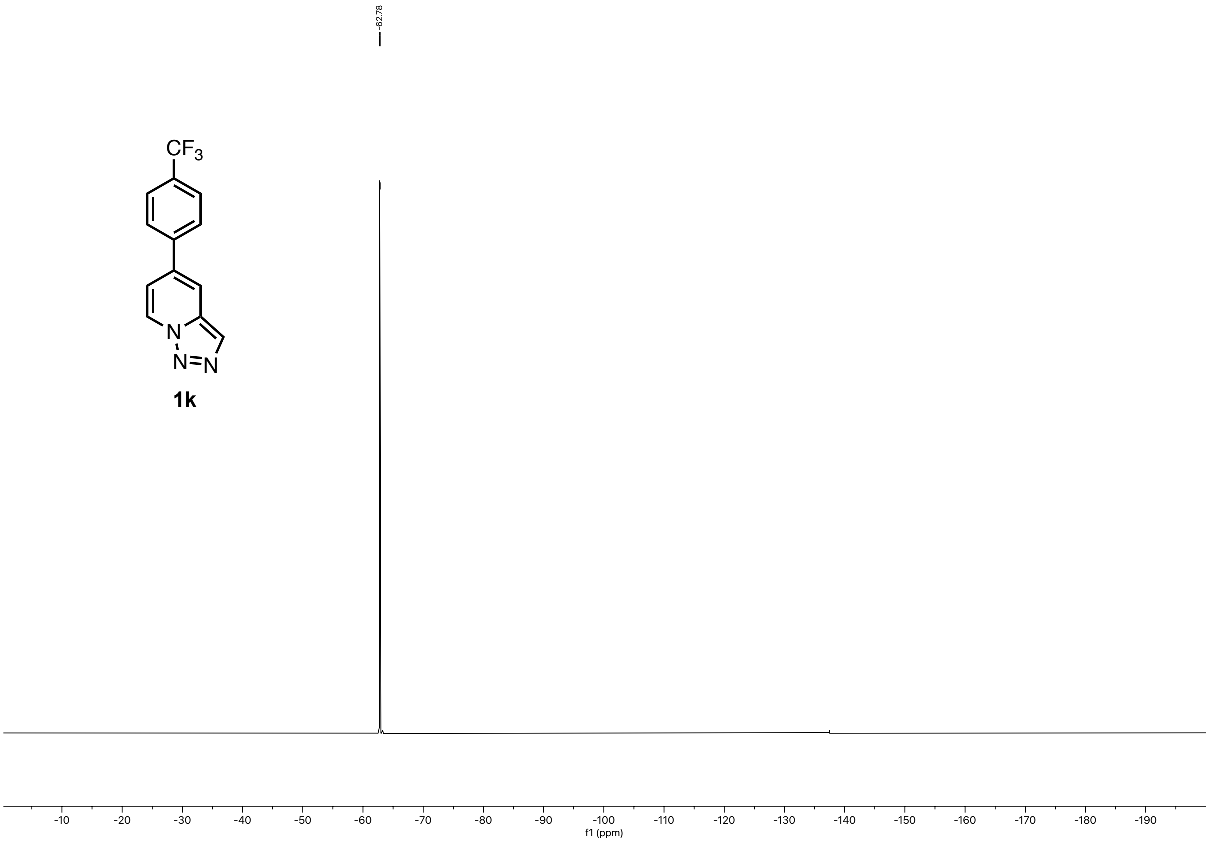

^{19}F spectrum (376 MHz, CDCl3) of **1k**

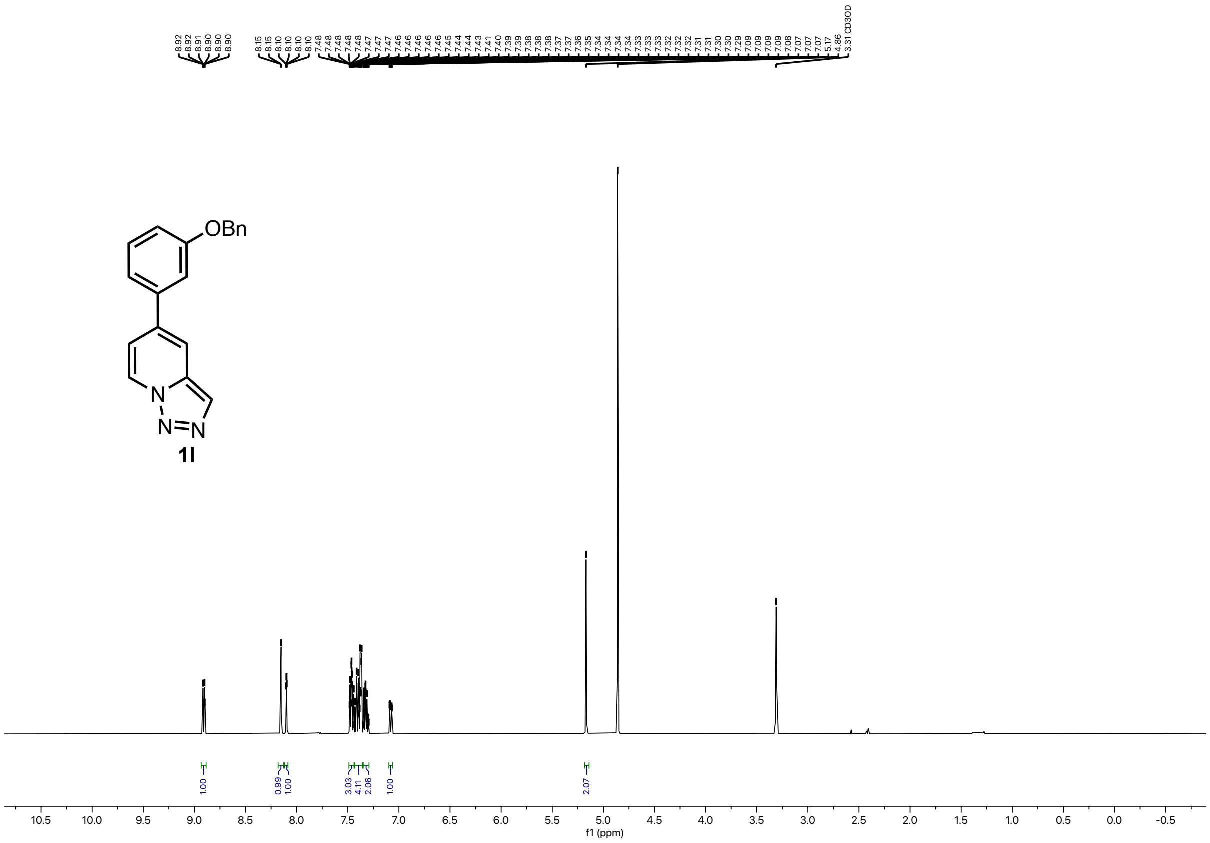

^{1}H spectrum (500 MHz, CD_3OD) of **1l**

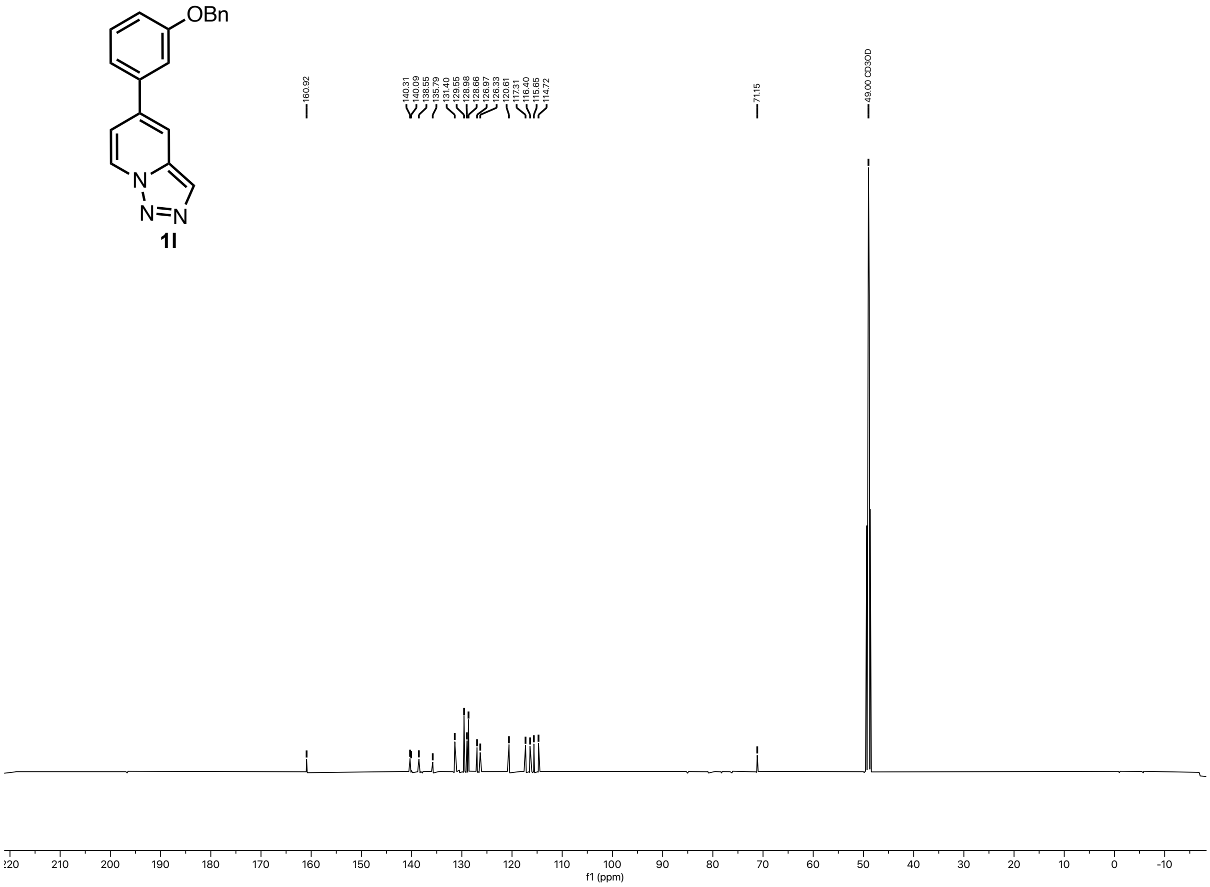

^{13}C spectrum (126 MHz, CD_3OD) of **1l**

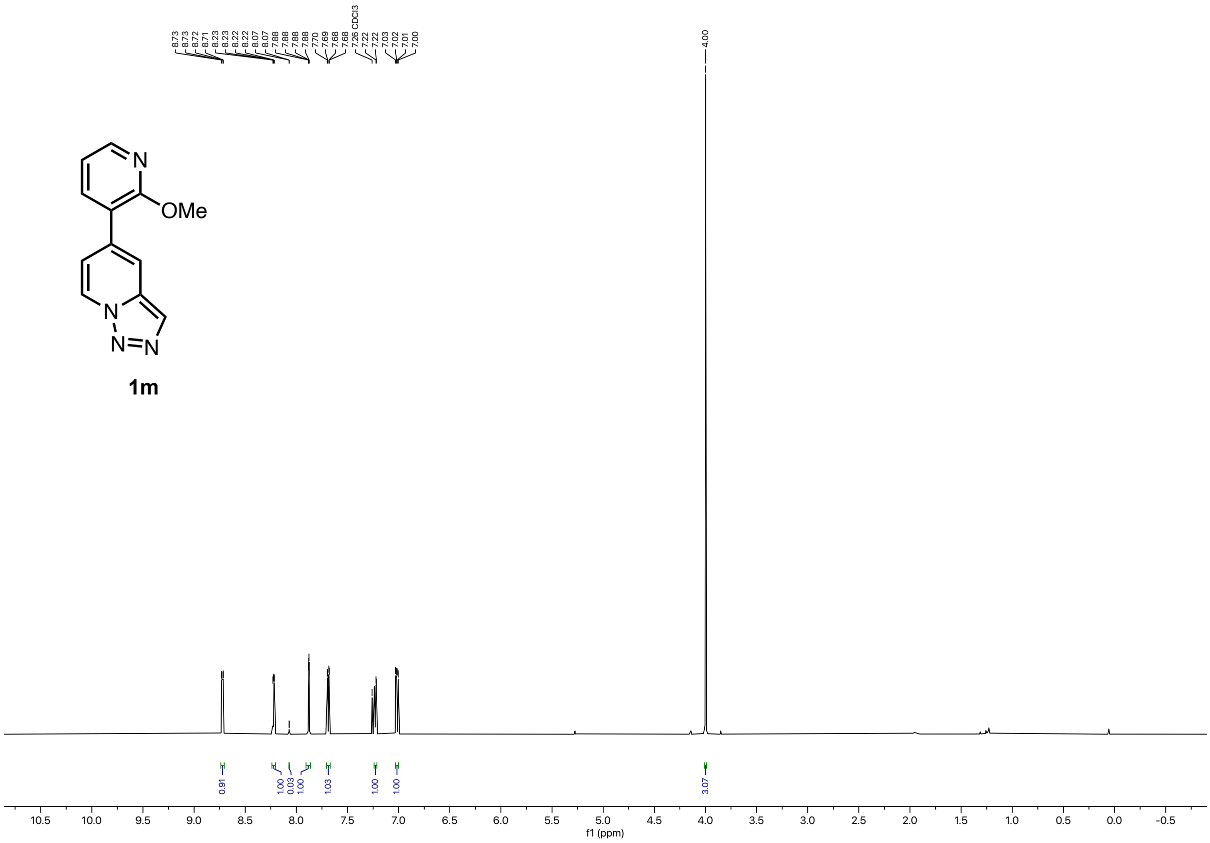

^{1}H spectrum (500 MHz, CD_3Cl) of **1m**

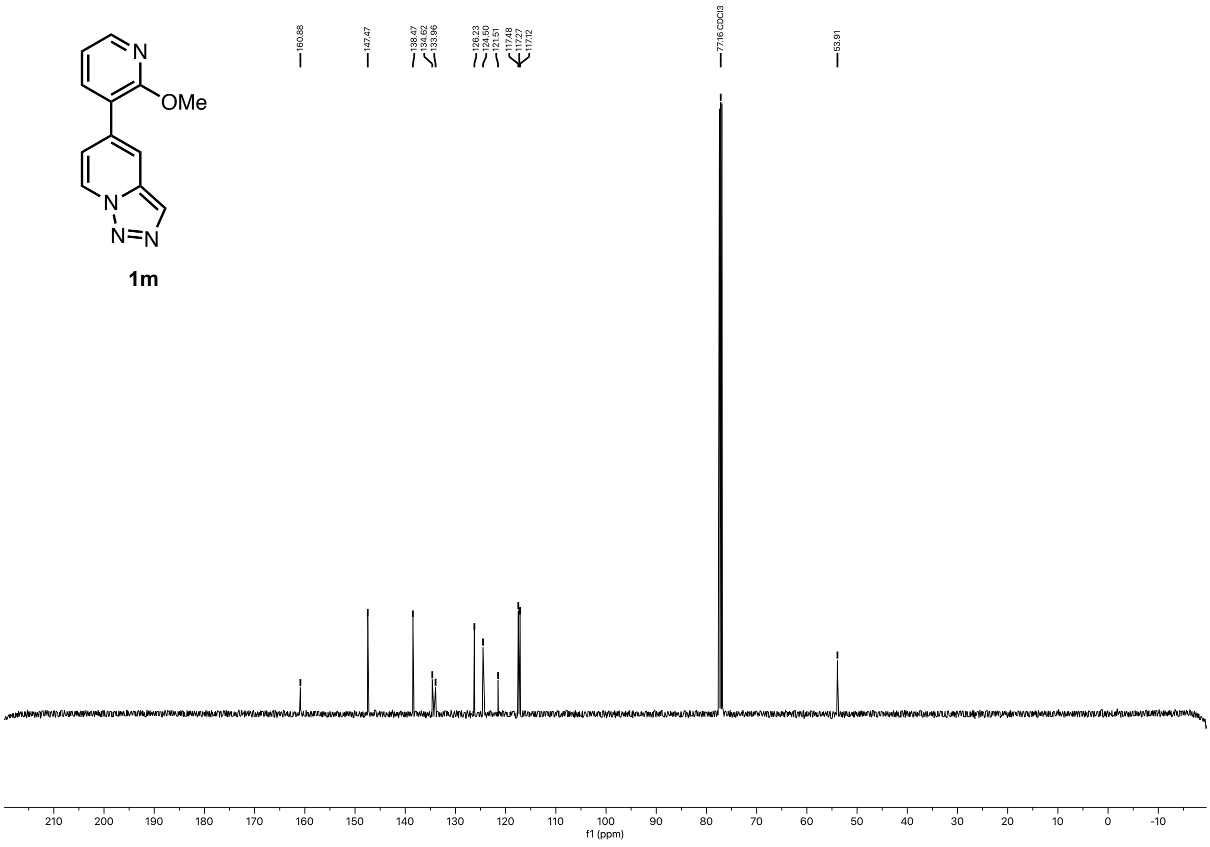

^{13}C spectrum (126 MHz, CD_3Cl) of **1m**

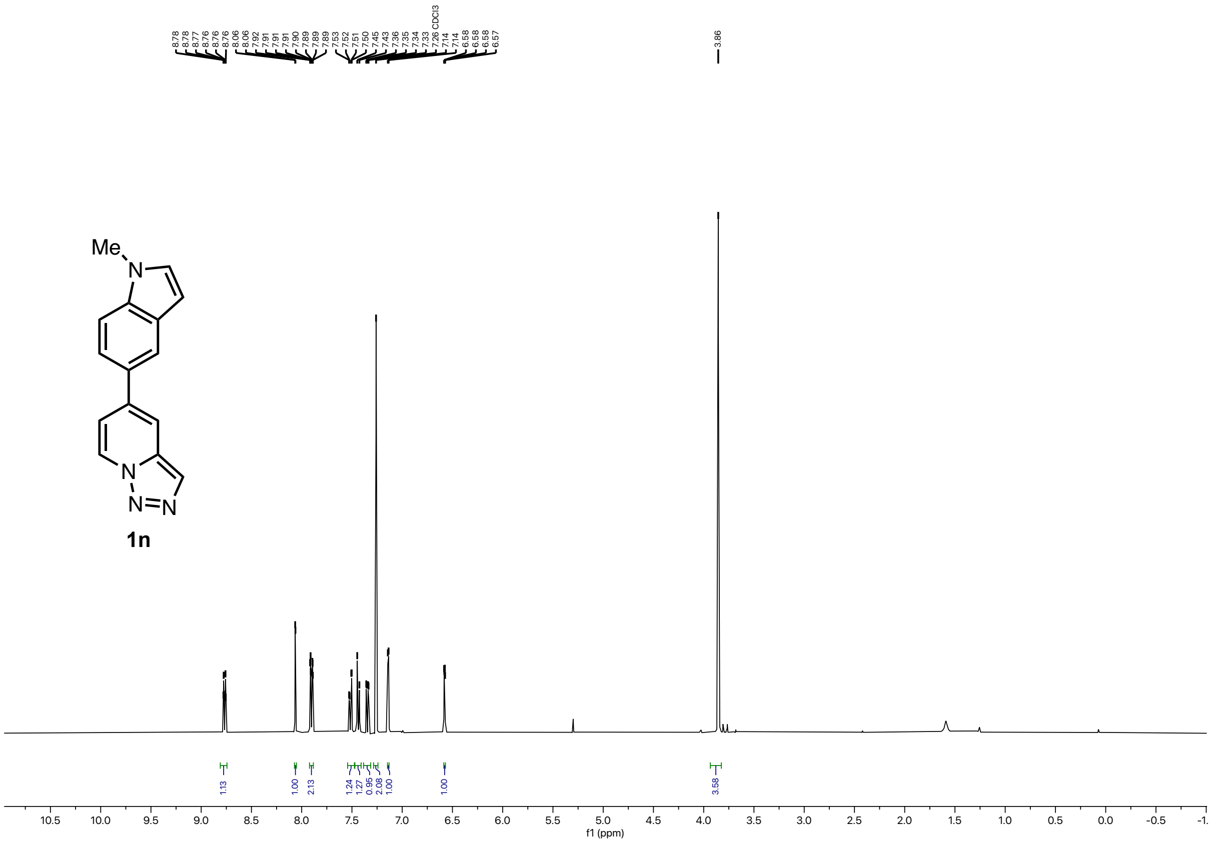

^{1}H spectrum (500 MHz, CD3Cl) of **1n**

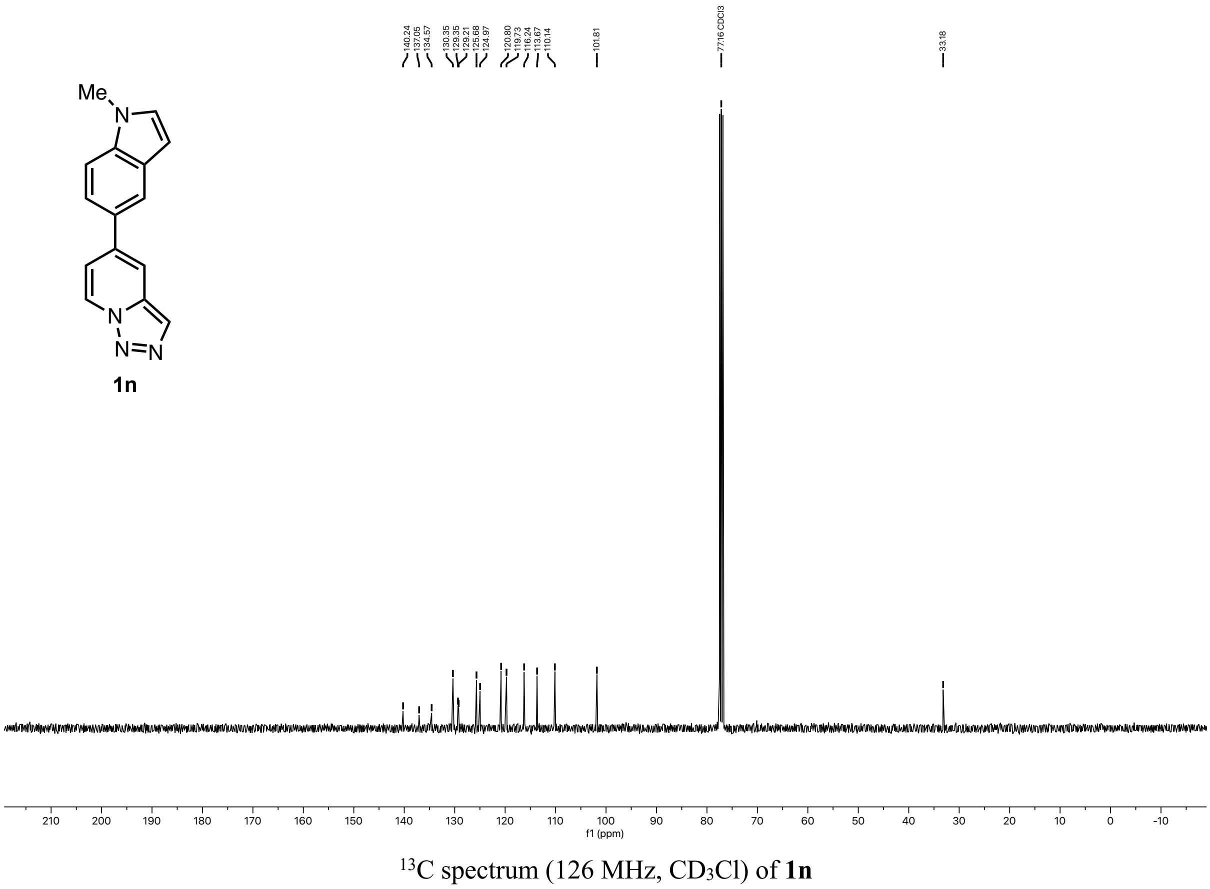

^{13}C spectrum (126 MHz, CD3Cl) of **1n**

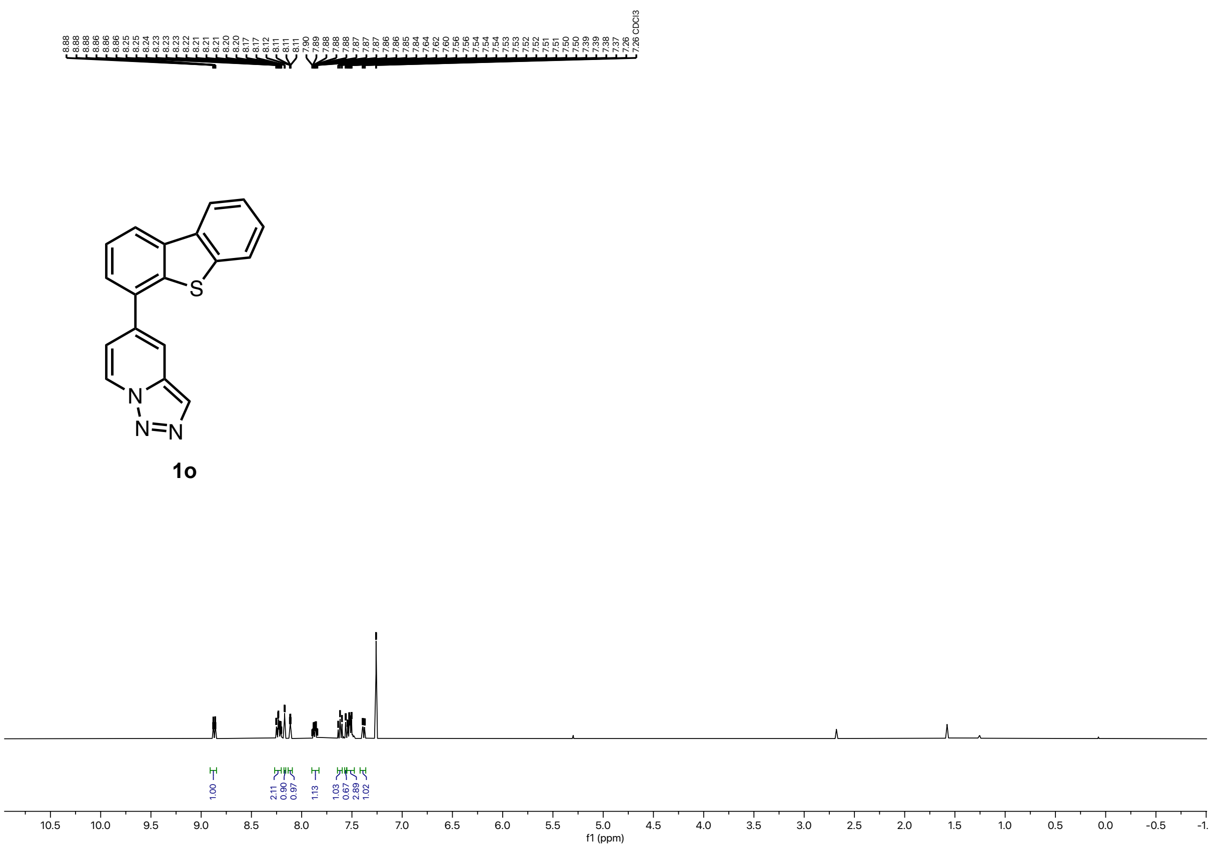

^{1}H spectrum (500 MHz, CD$_3$Cl) of **1o**

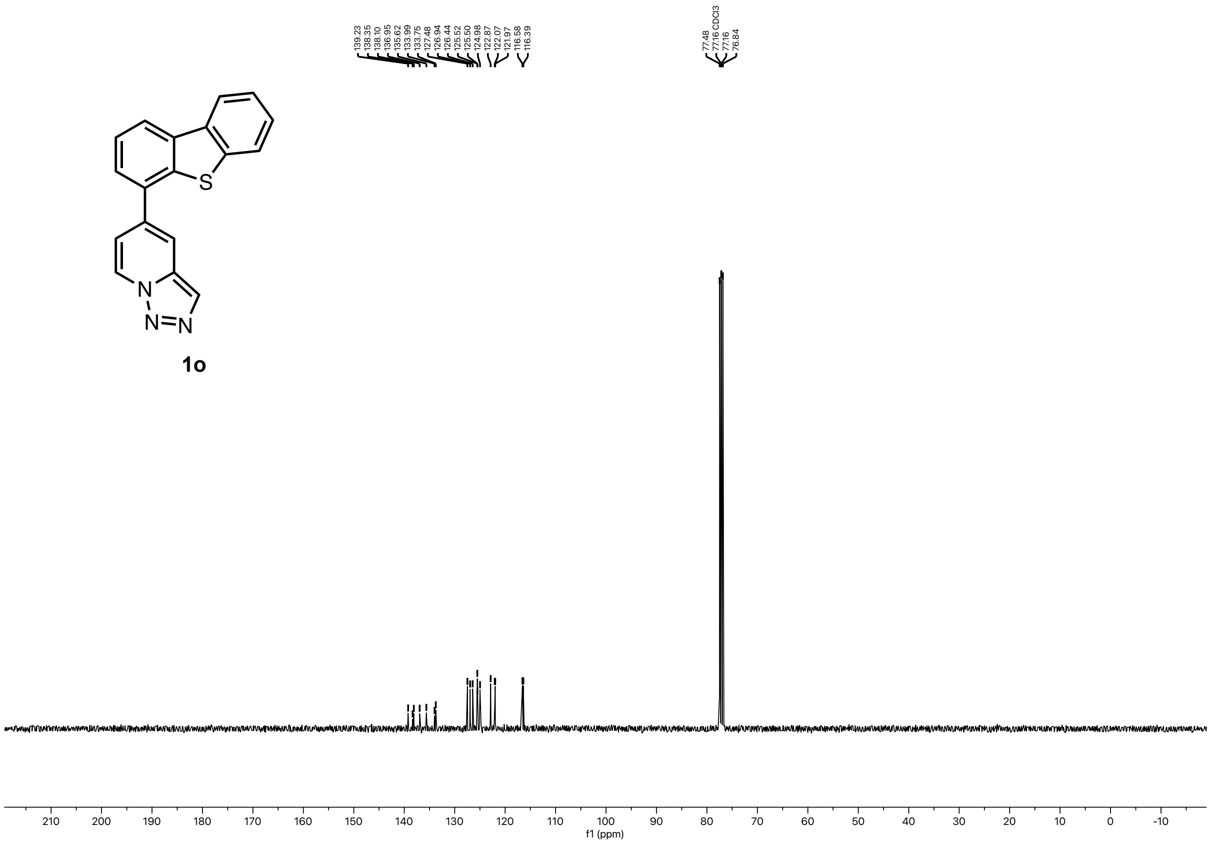

^{13}C spectrum (126 MHz, CD$_3$Cl) of **1o**

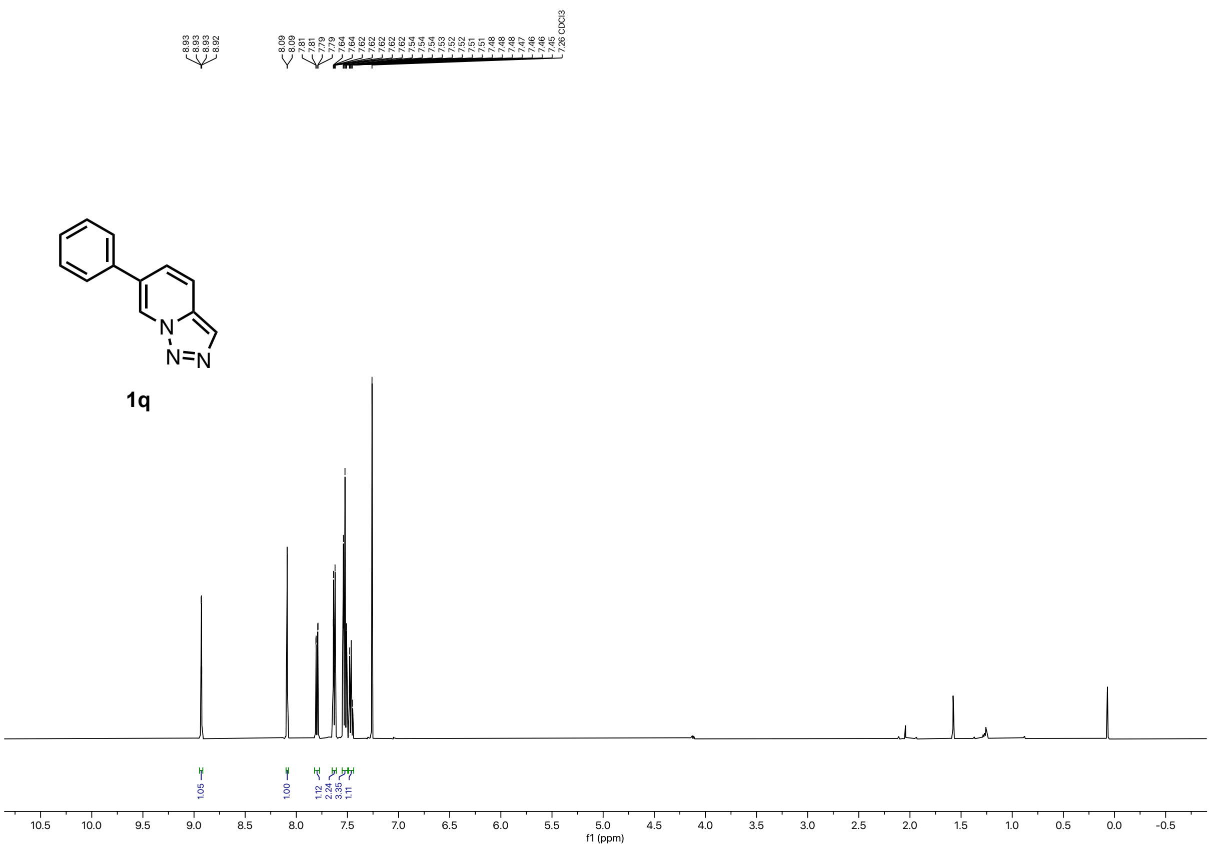

^{1}H spectrum (500 MHz, CD_3Cl) of **1q**

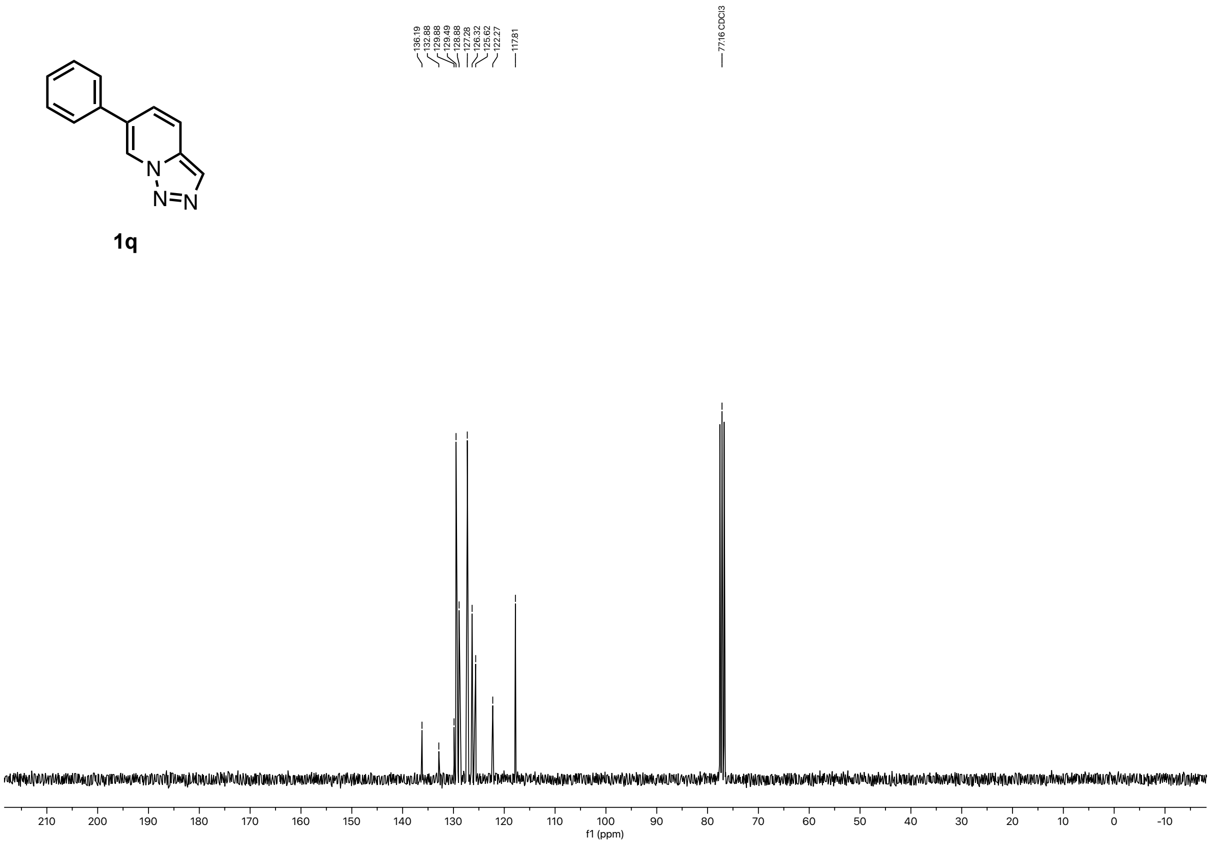

^{13}C spectrum (75 MHz, CD_3Cl) of **1q**

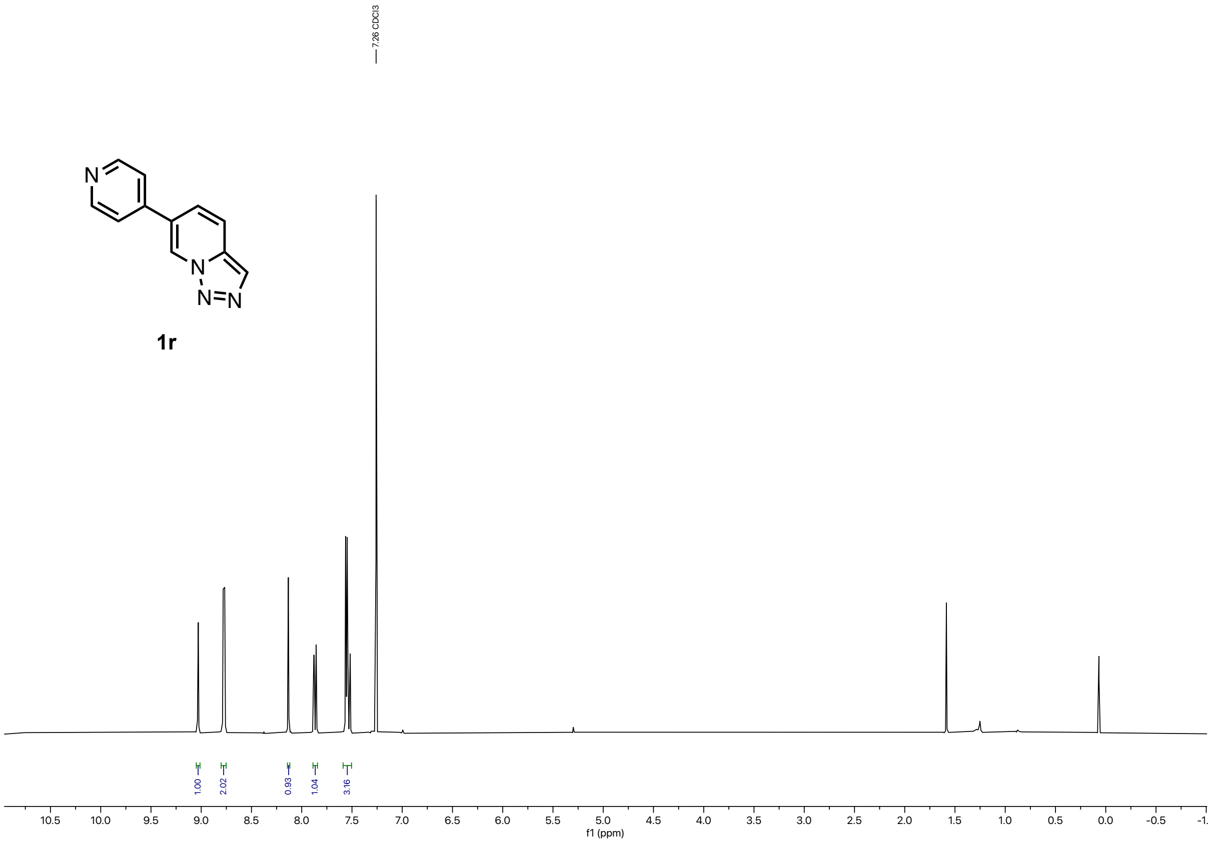

^{1}H spectrum (400 MHz, CD_3Cl) of **1r**

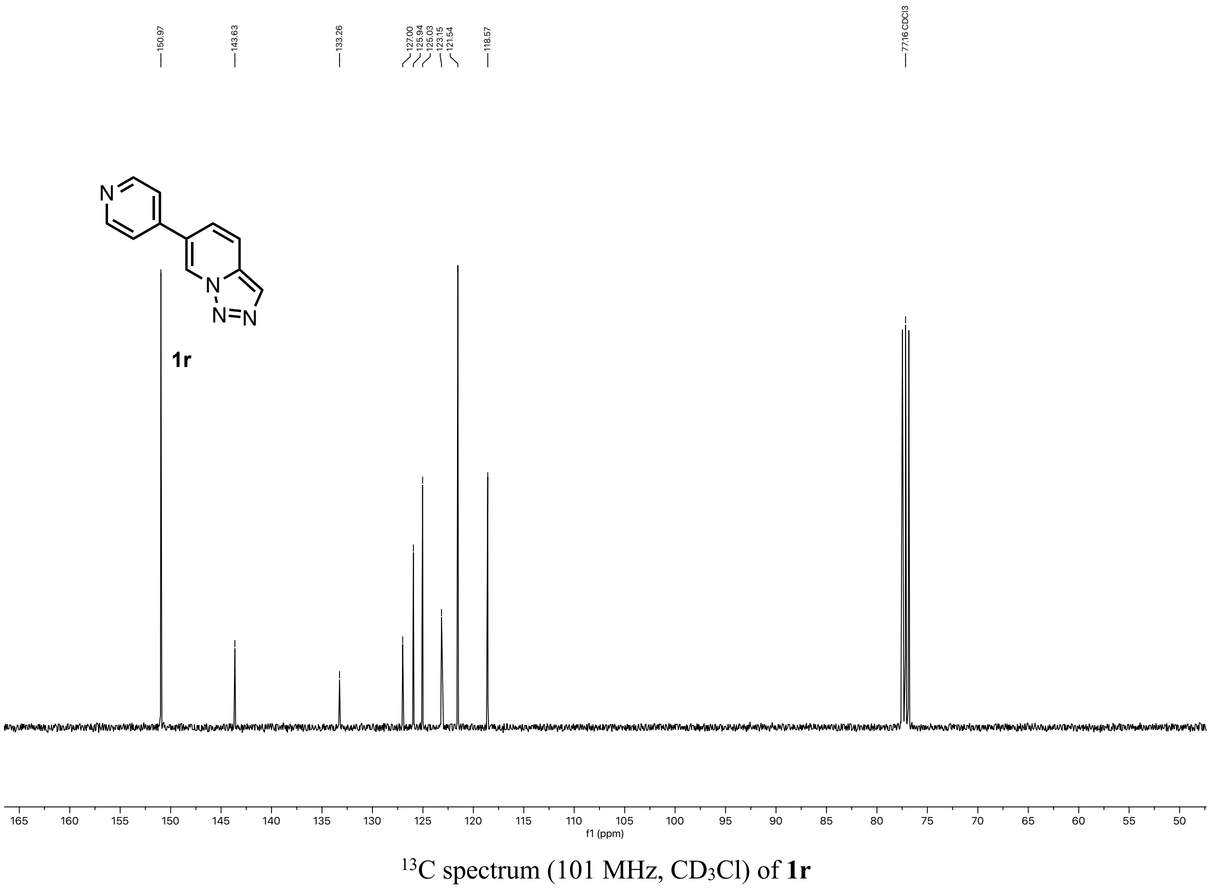

^{13}C spectrum (101 MHz, CD_3Cl) of **1r**

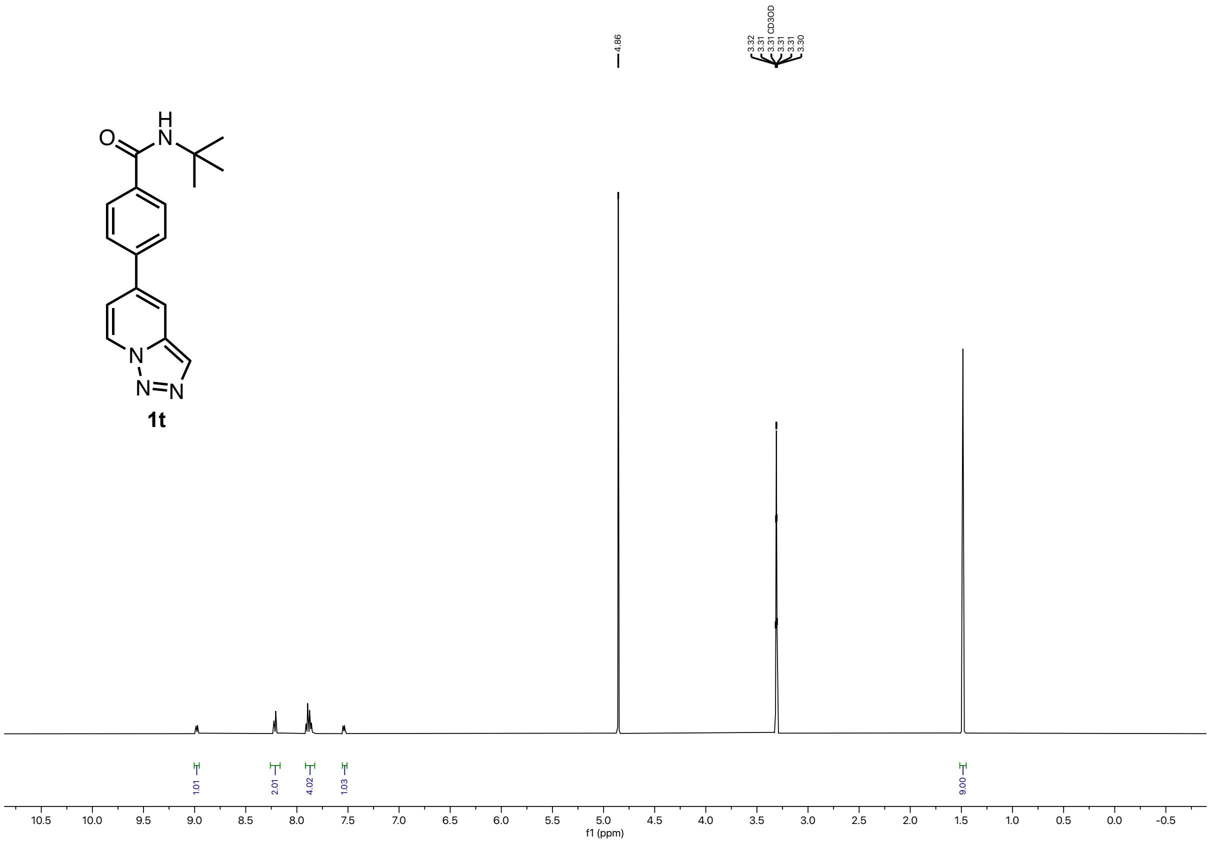

^{1}H spectrum (500 MHz, CD_3OD) of **1t**

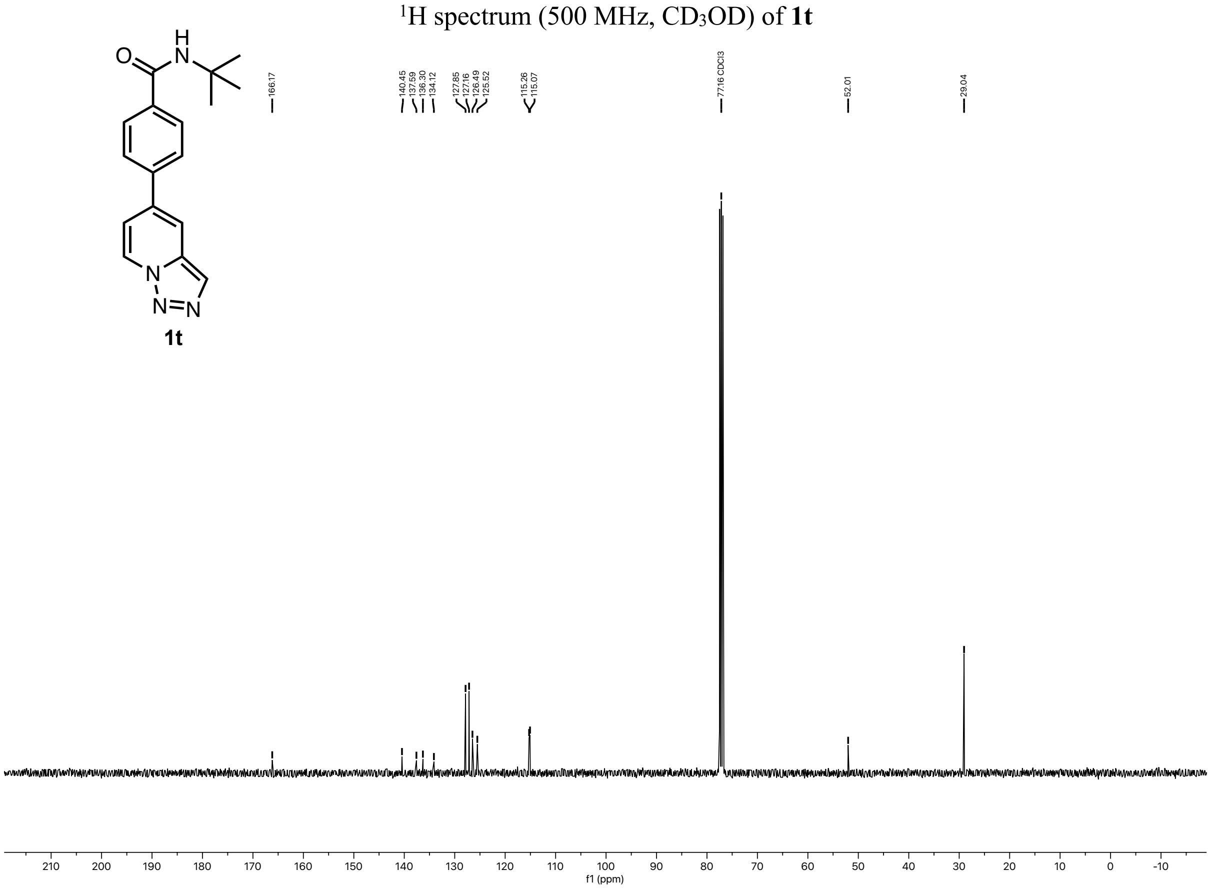

^{13}C spectrum (126 MHz, CD_3Cl) of **1t**

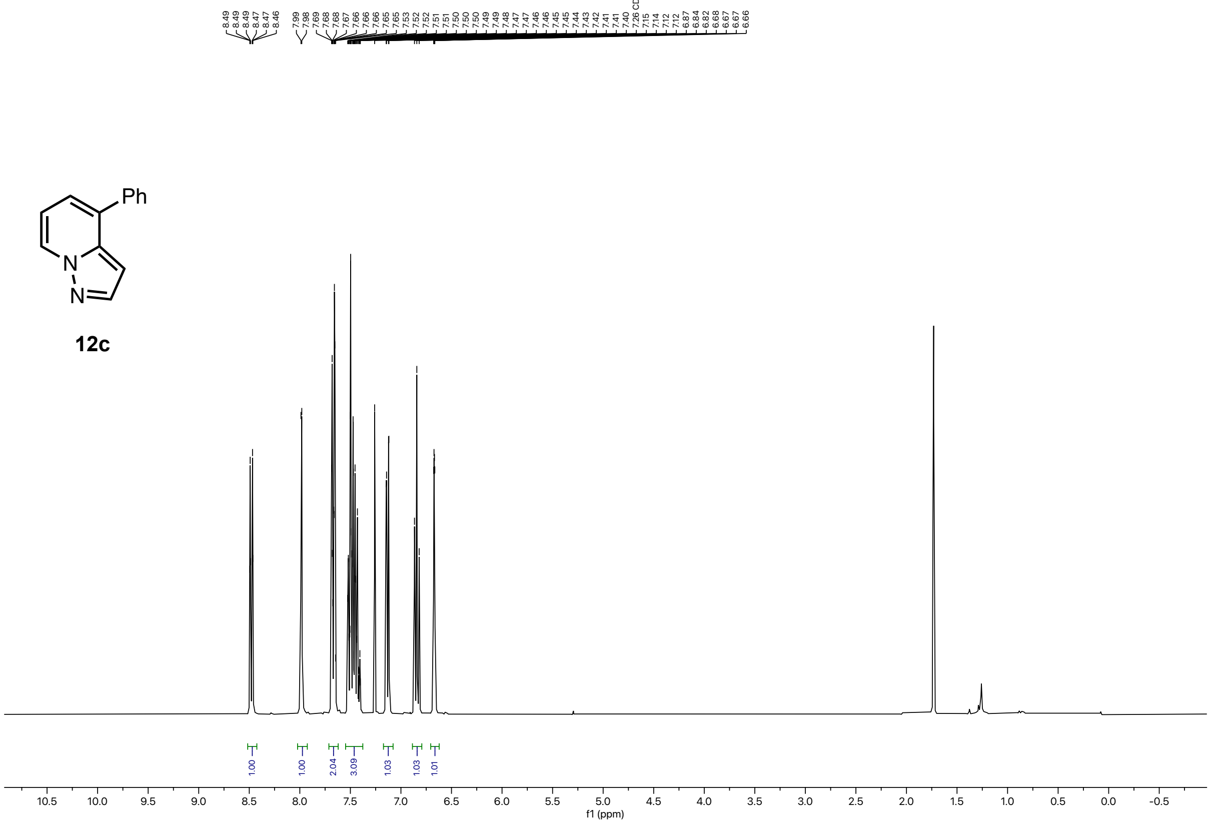

^1H spectrum (300 MHz, CD_3Cl) of **12c**

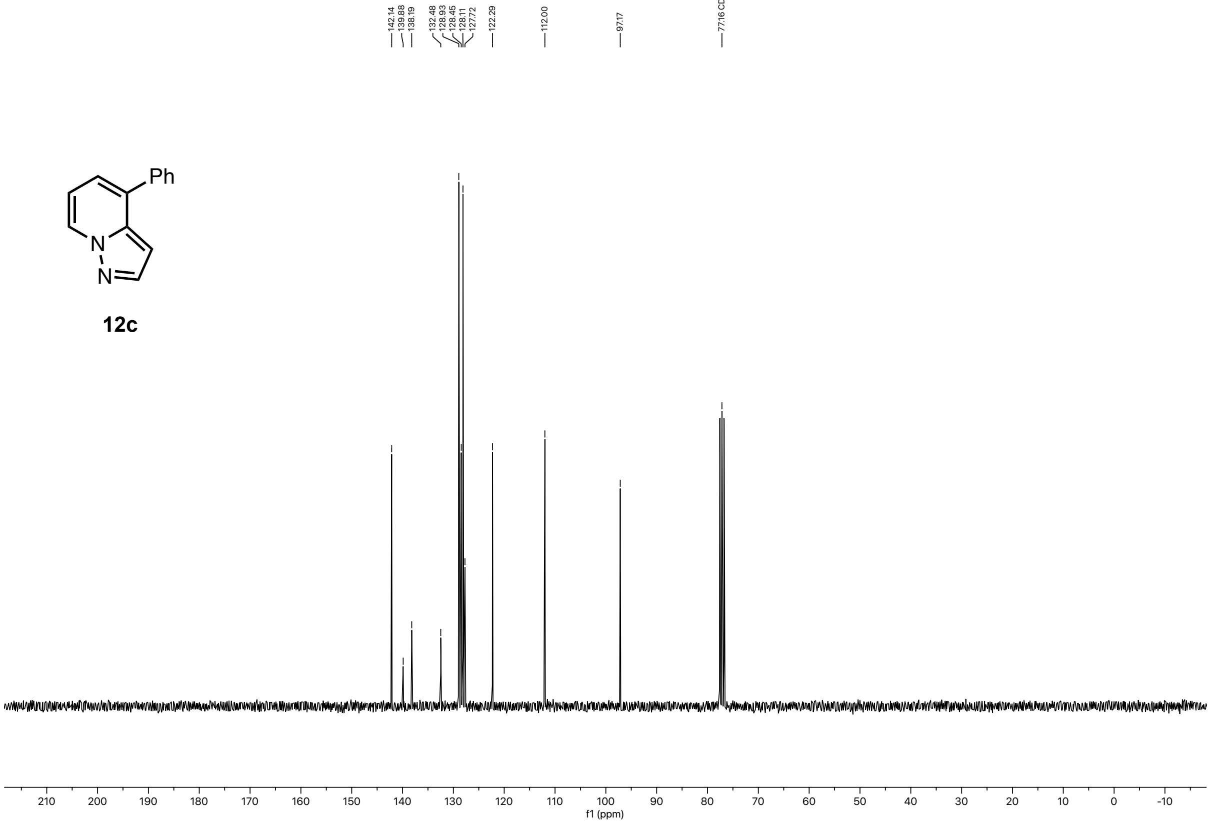

^{13}C spectrum (75 MHz, CD_3Cl) of **12c**

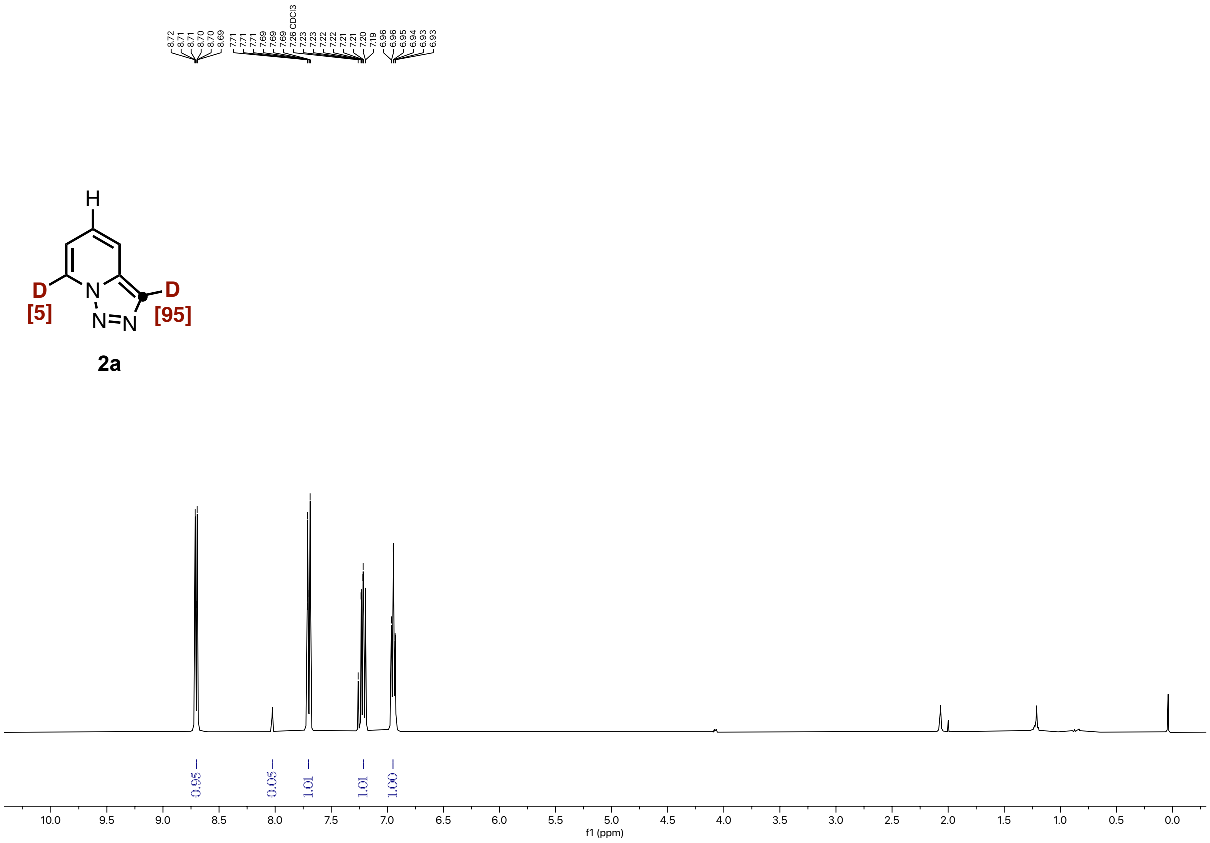

^{1}H spectrum (500 MHz, $CDCl_3$) of **2a**

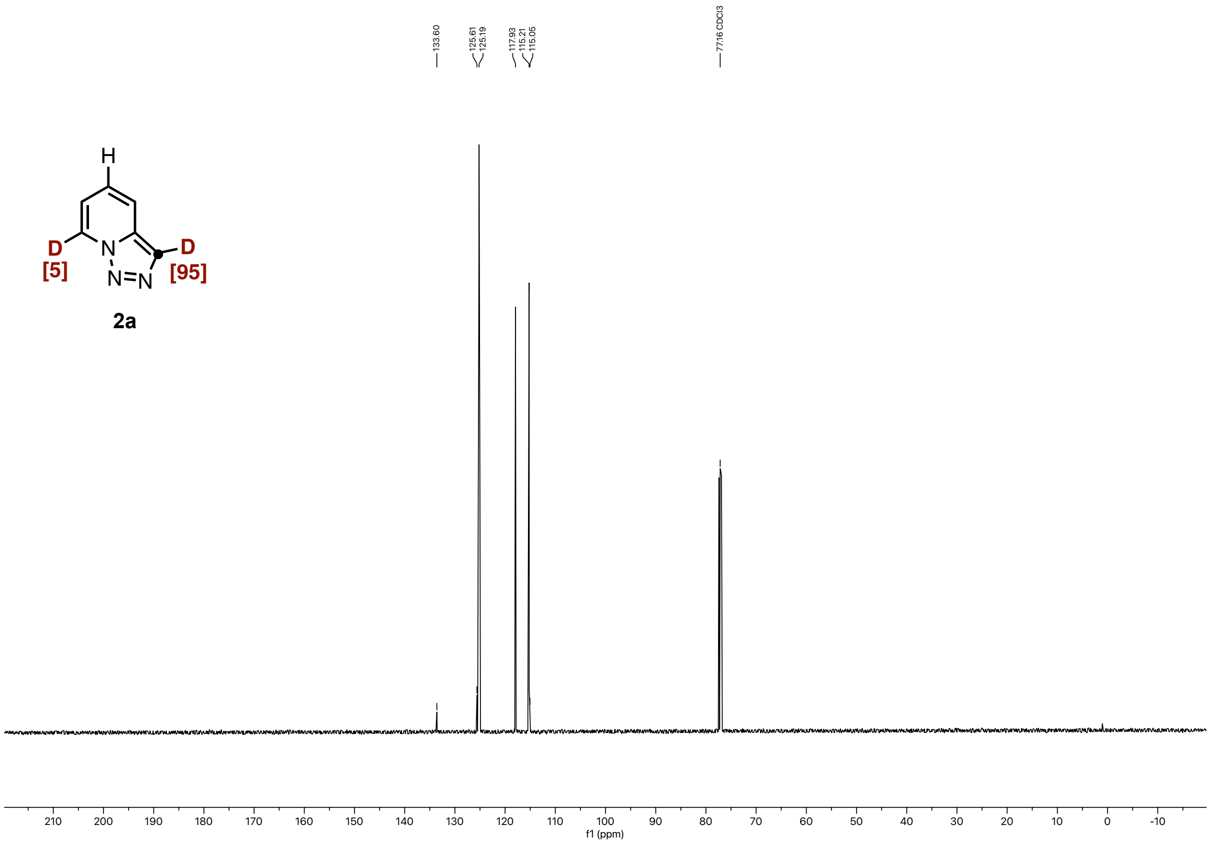

^{13}C spectrum (126 MHz, CD_3Cl) of **2a**

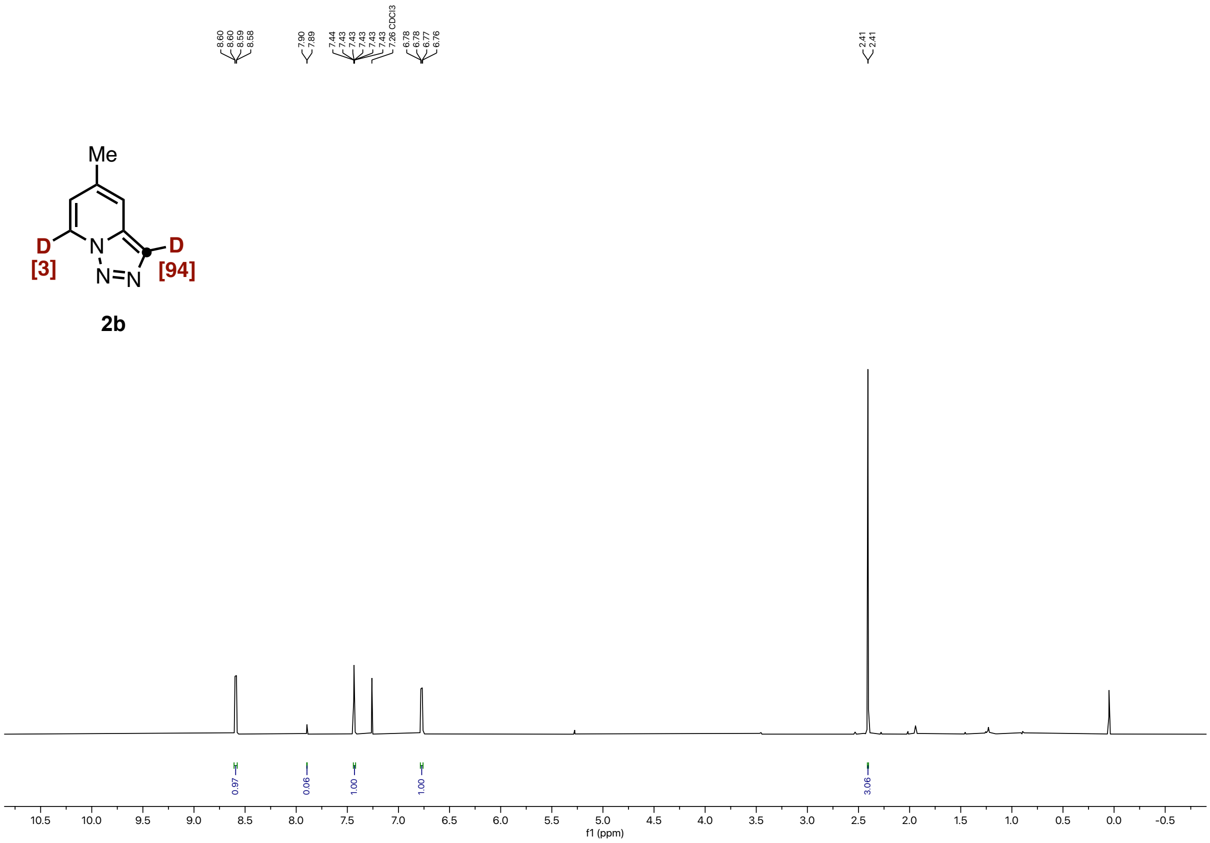

^{1}H spectrum (500 MHz, CD_3Cl) of **2b**

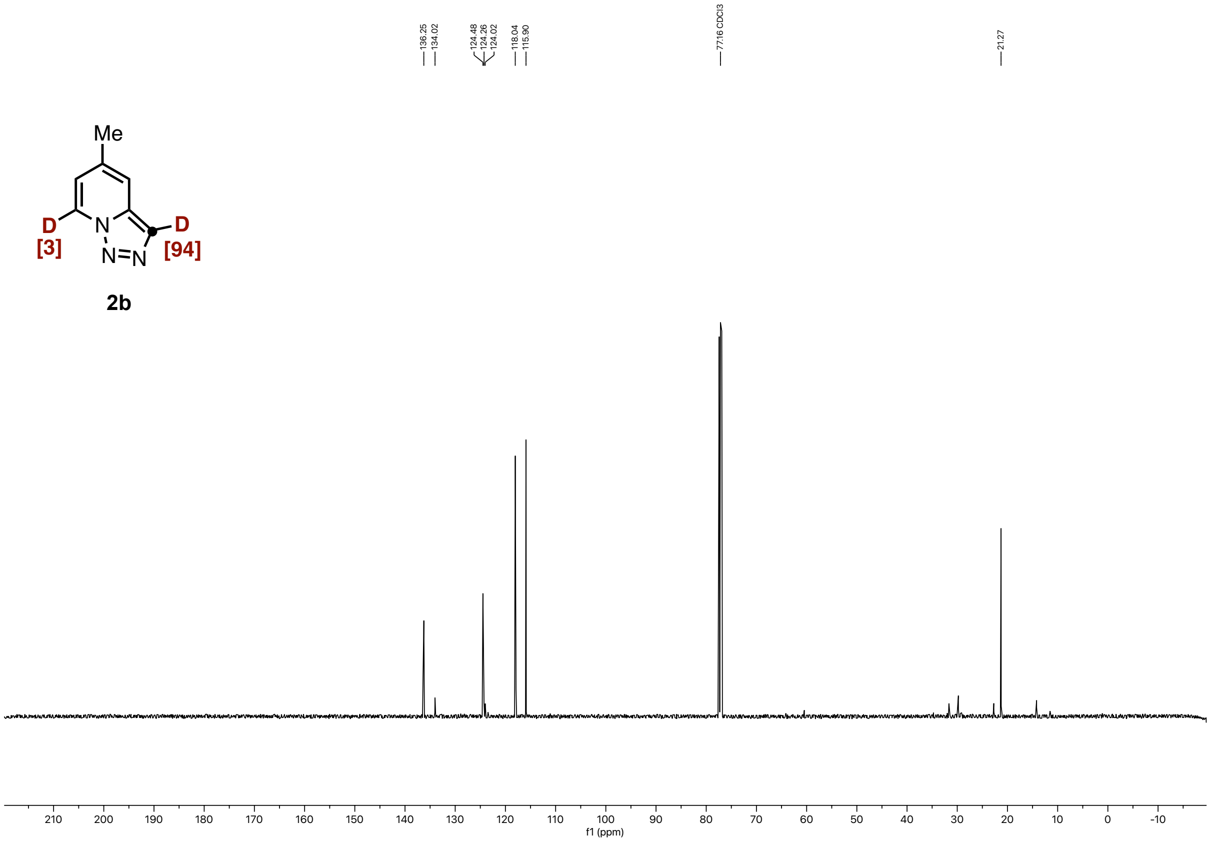

^{13}C spectrum (126 MHz, CD_3Cl) of **2b**

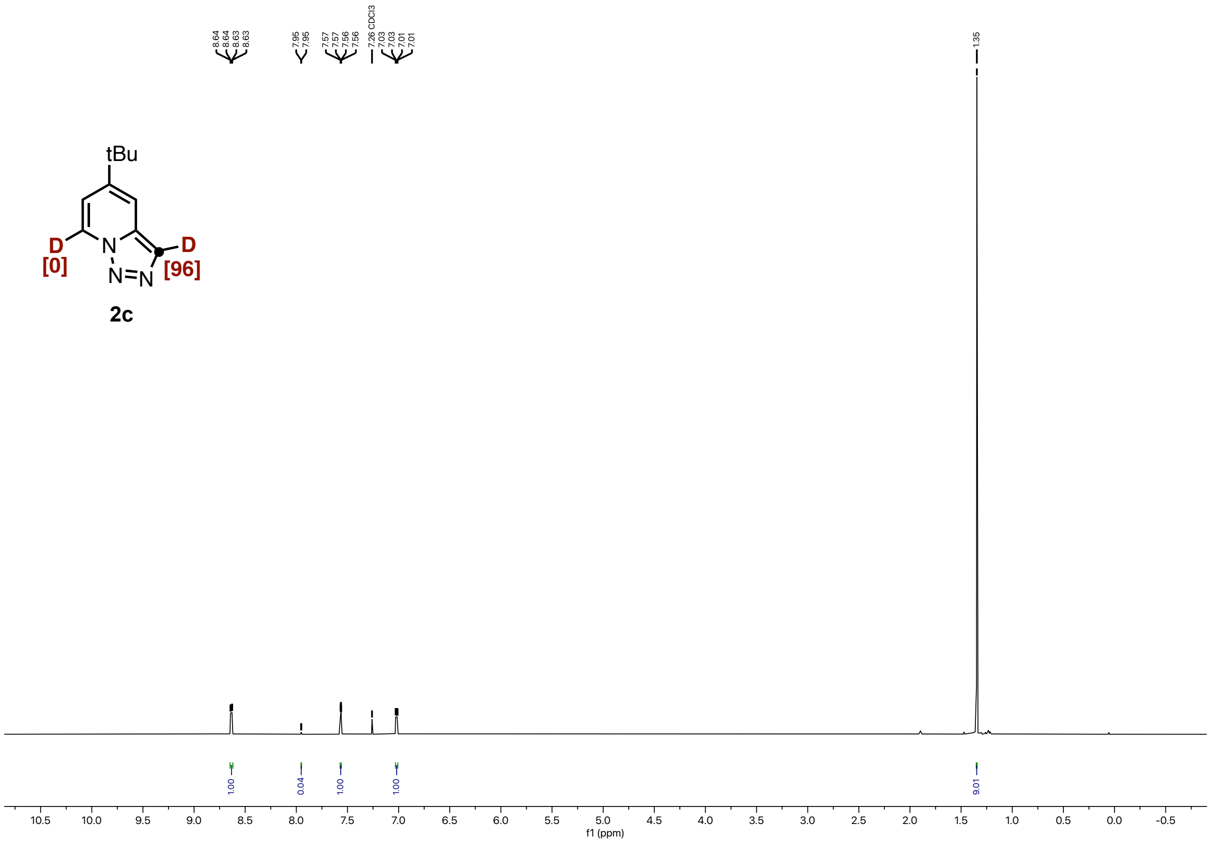

^{1}H spectrum (500 MHz, CD$_3$Cl) of **2c**

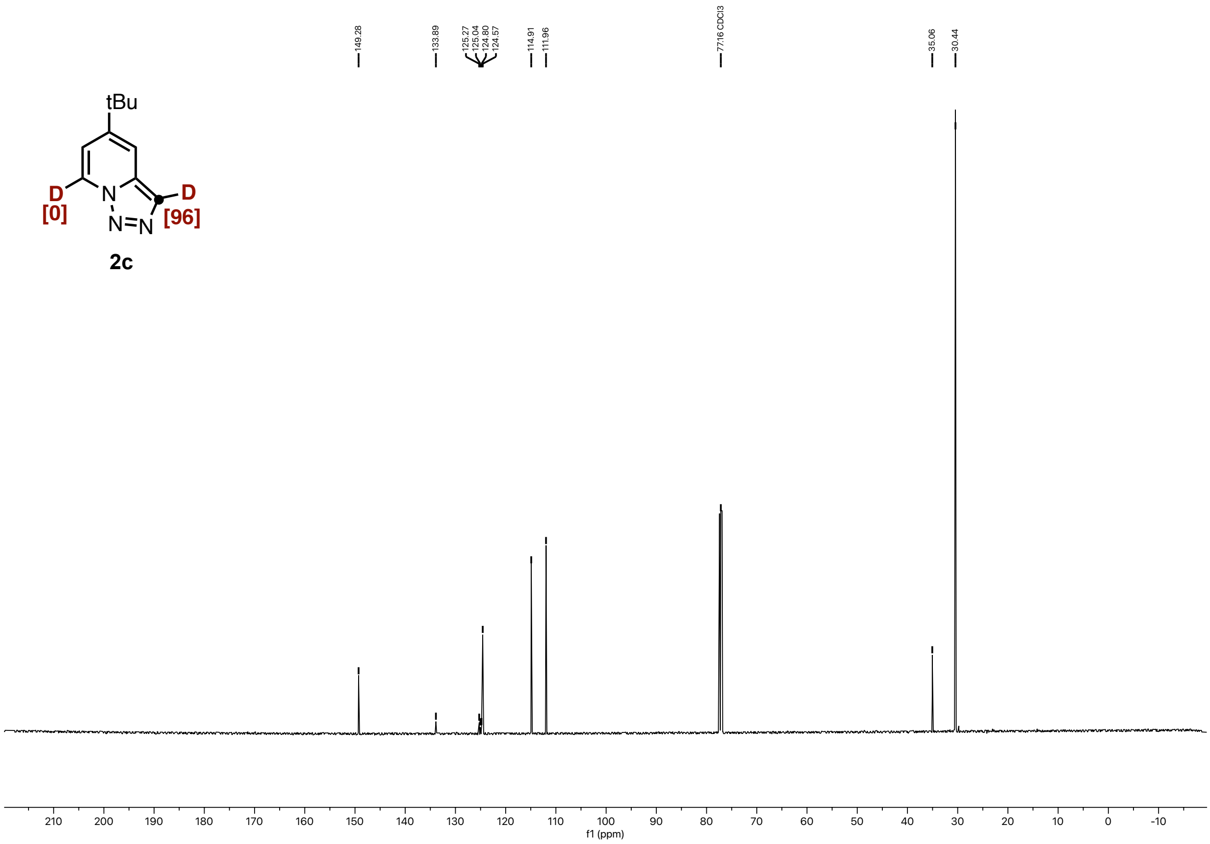

^{13}C spectrum (126 MHz, CD$_3$Cl) of **2c**

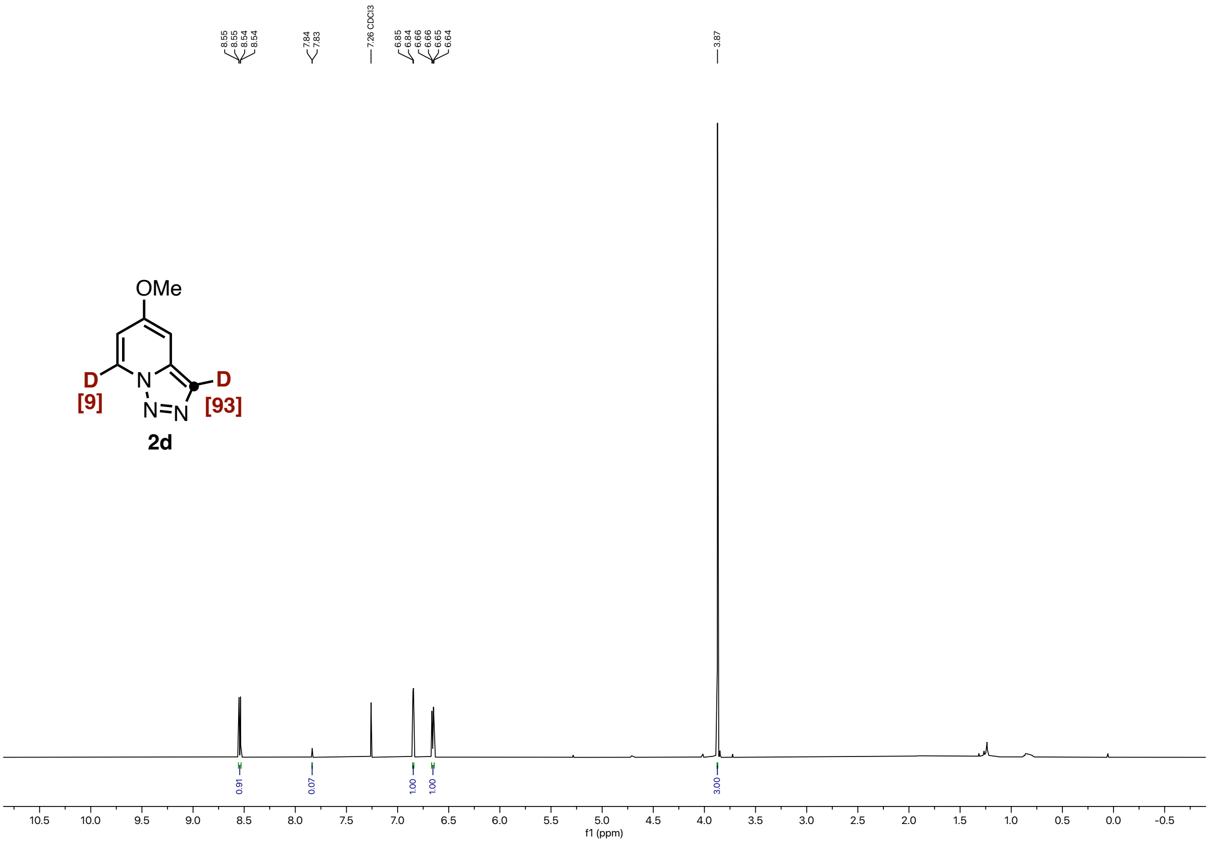

^{1}H spectrum (500 MHz, CD_3Cl) of **2d**

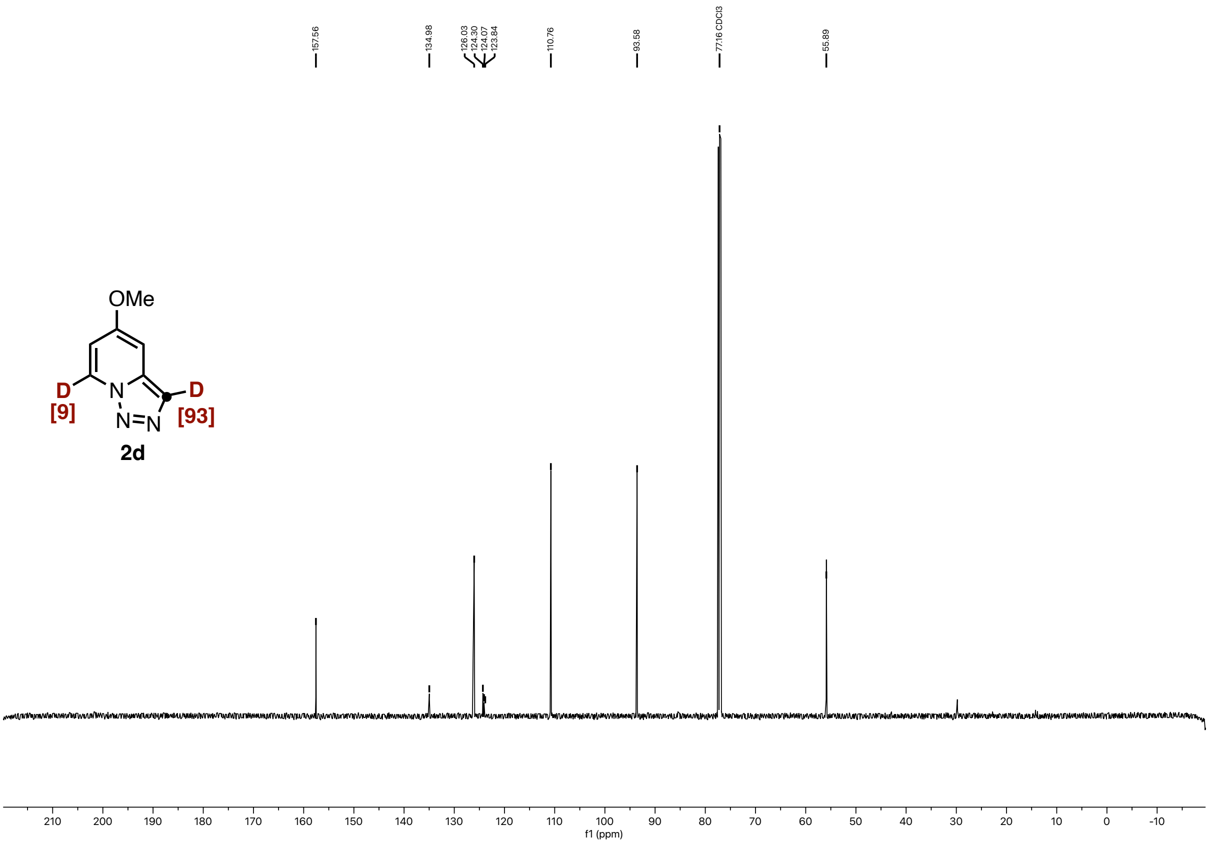

^{13}C spectrum (500 MHz, CD_3Cl) of **2d**

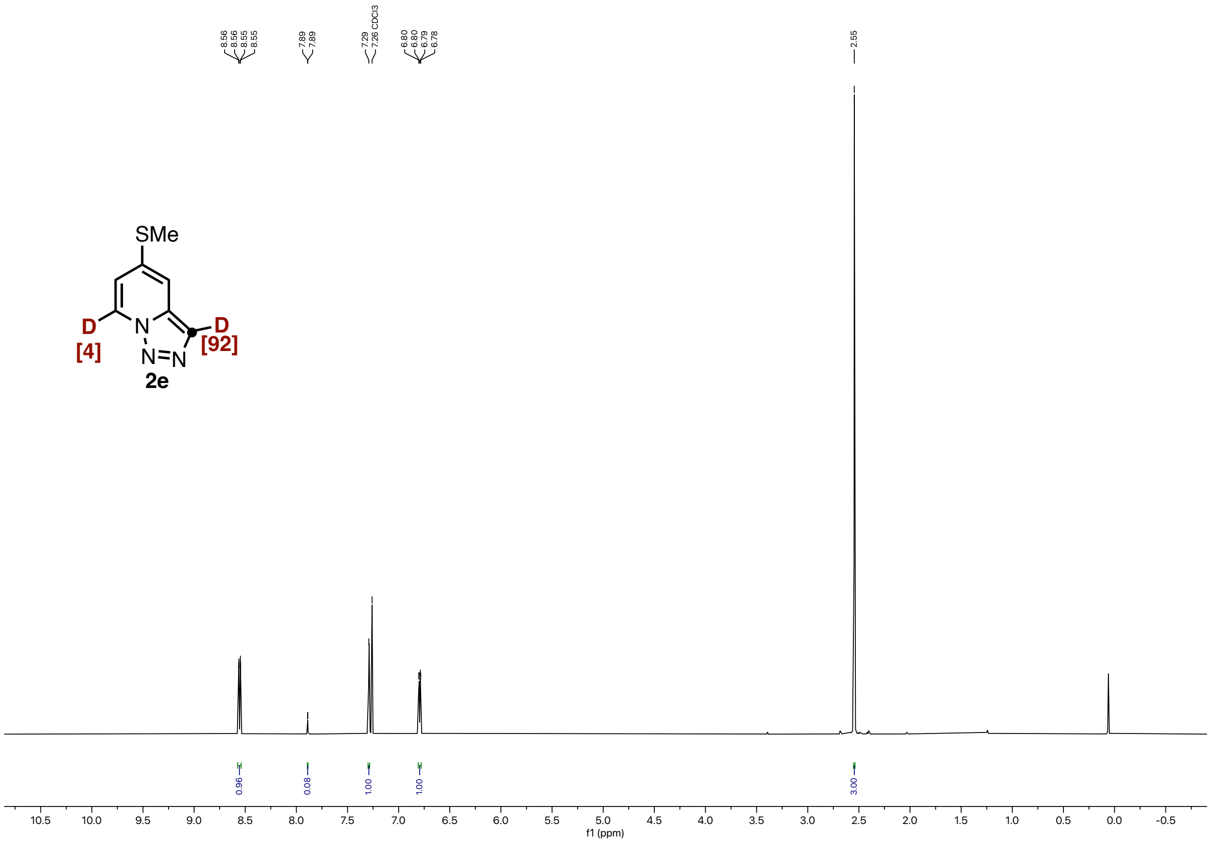

^{1}H spectrum (500 MHz, CD$_3$Cl) of **2e**

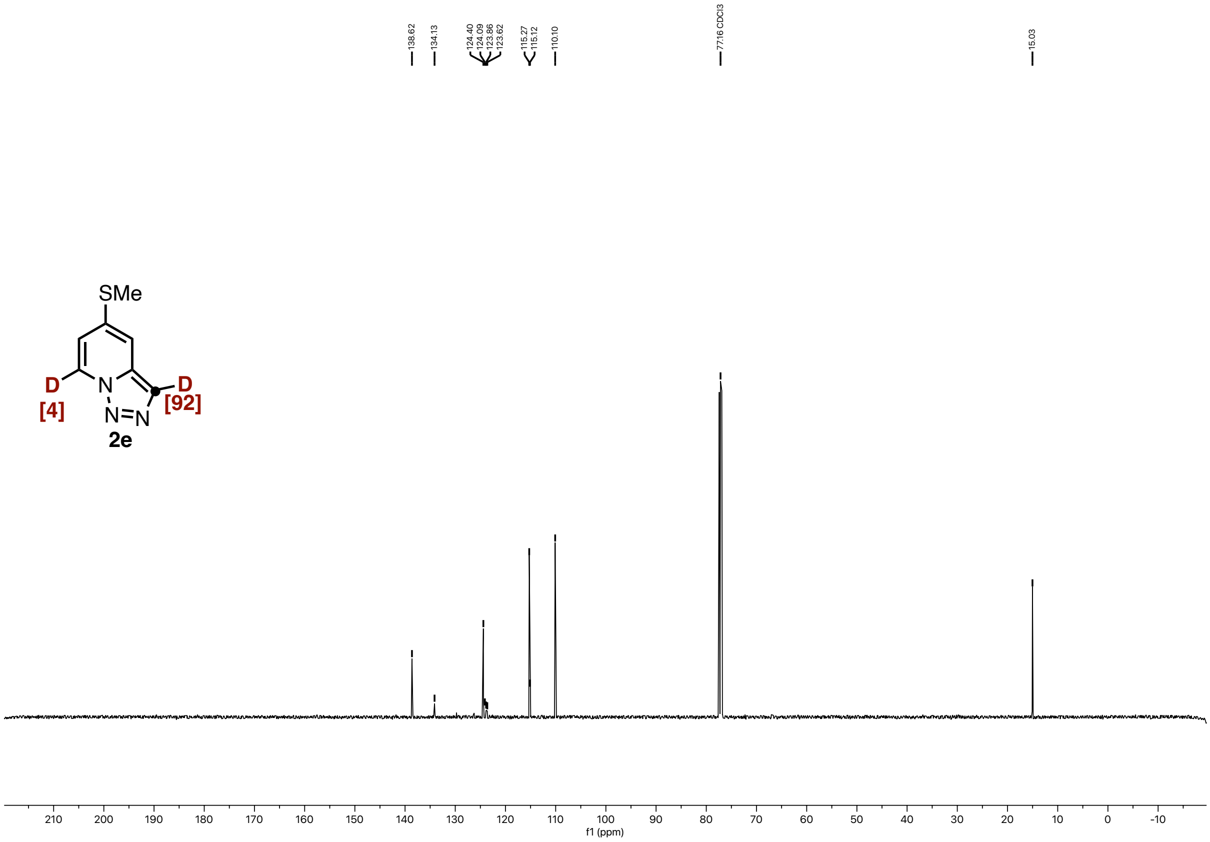

^{13}C spectrum (500 MHz, CD$_3$Cl) of **2e**

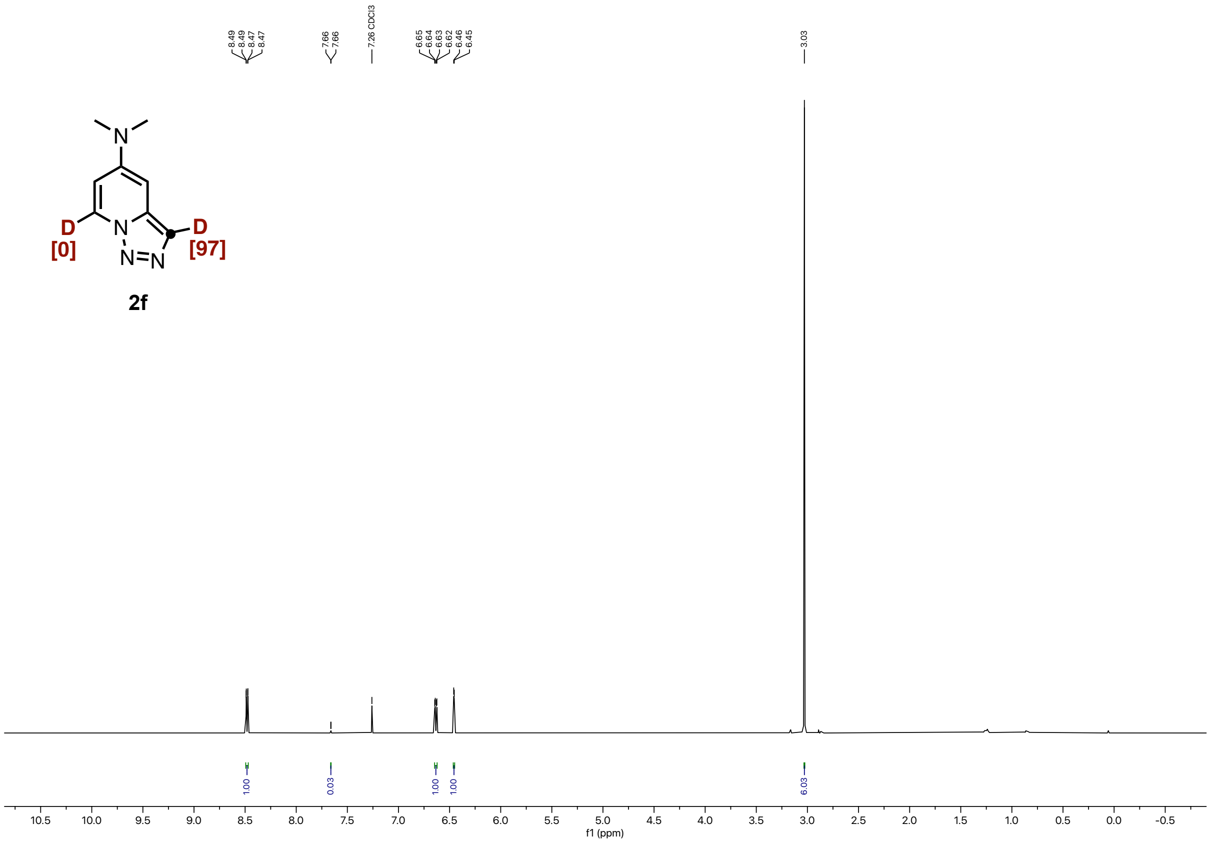

^{1}H spectrum (500 MHz, CD_3Cl) of **2f**

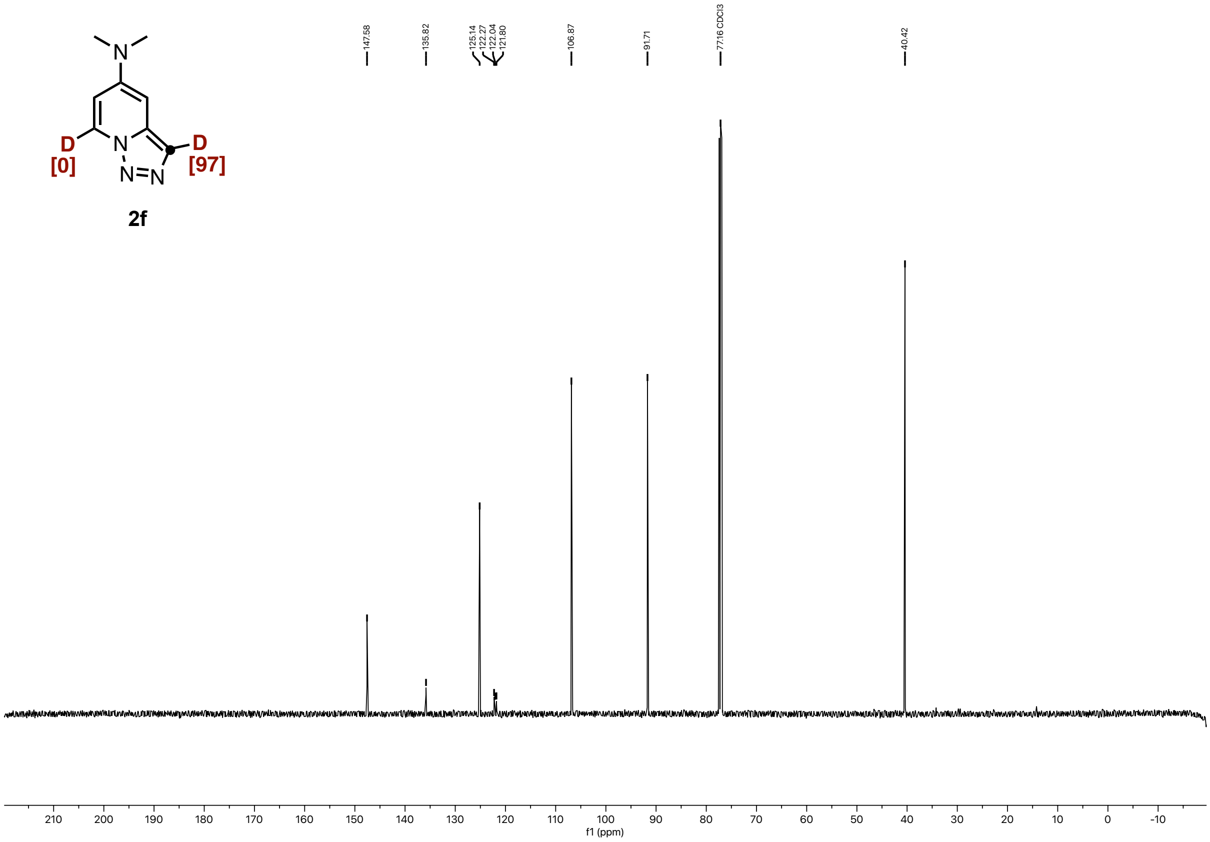

^{13}C spectrum (500 MHz, CD_3Cl) of **2f**

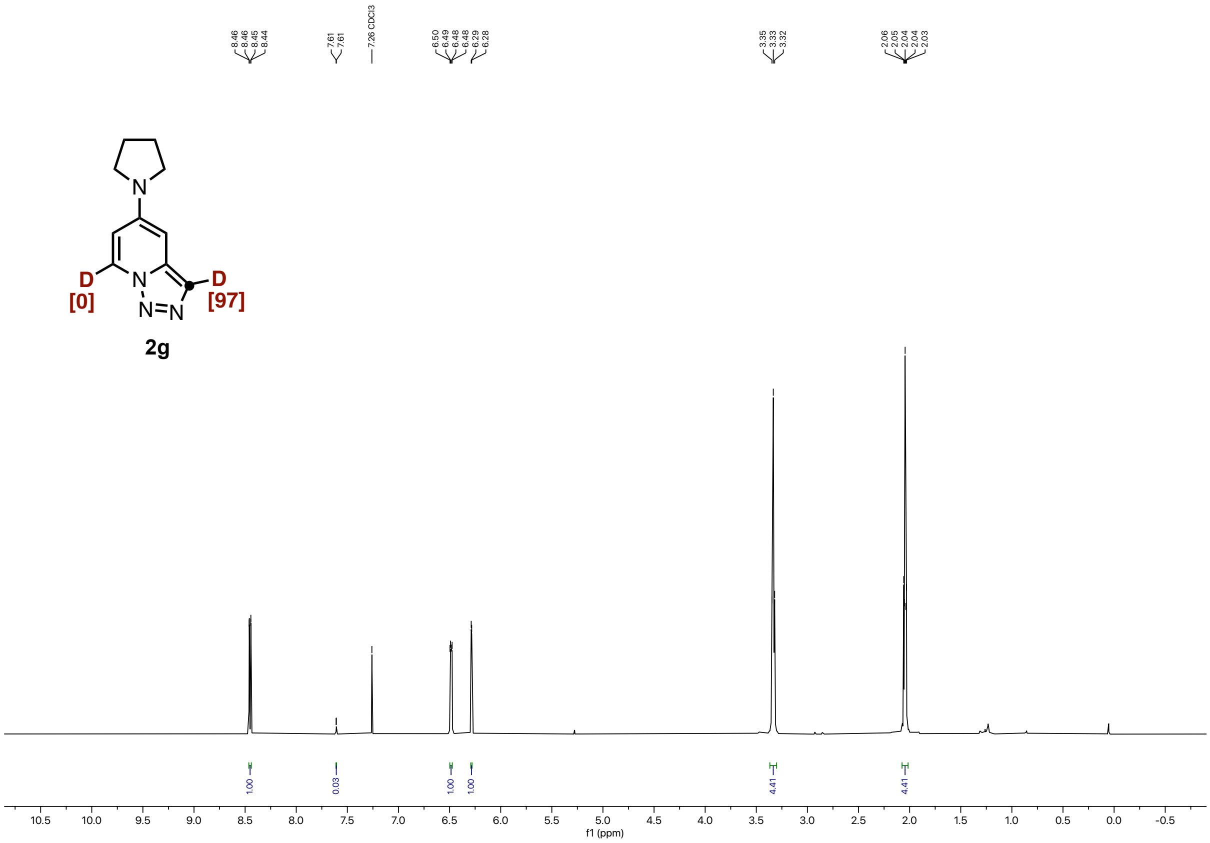

^{1}H spectrum (500 MHz, CD_3Cl) of **2g**

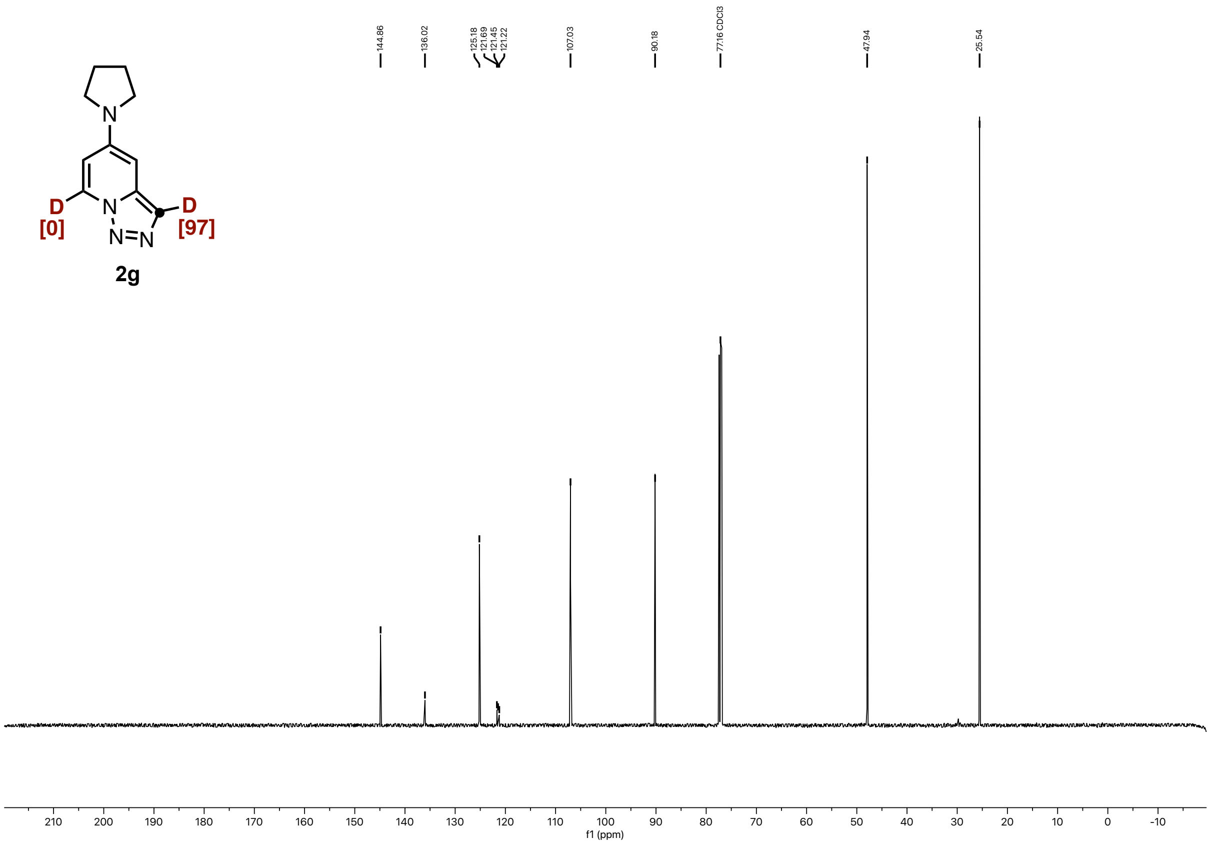

^{13}C spectrum (500 MHz, CD_3Cl) of **2g**

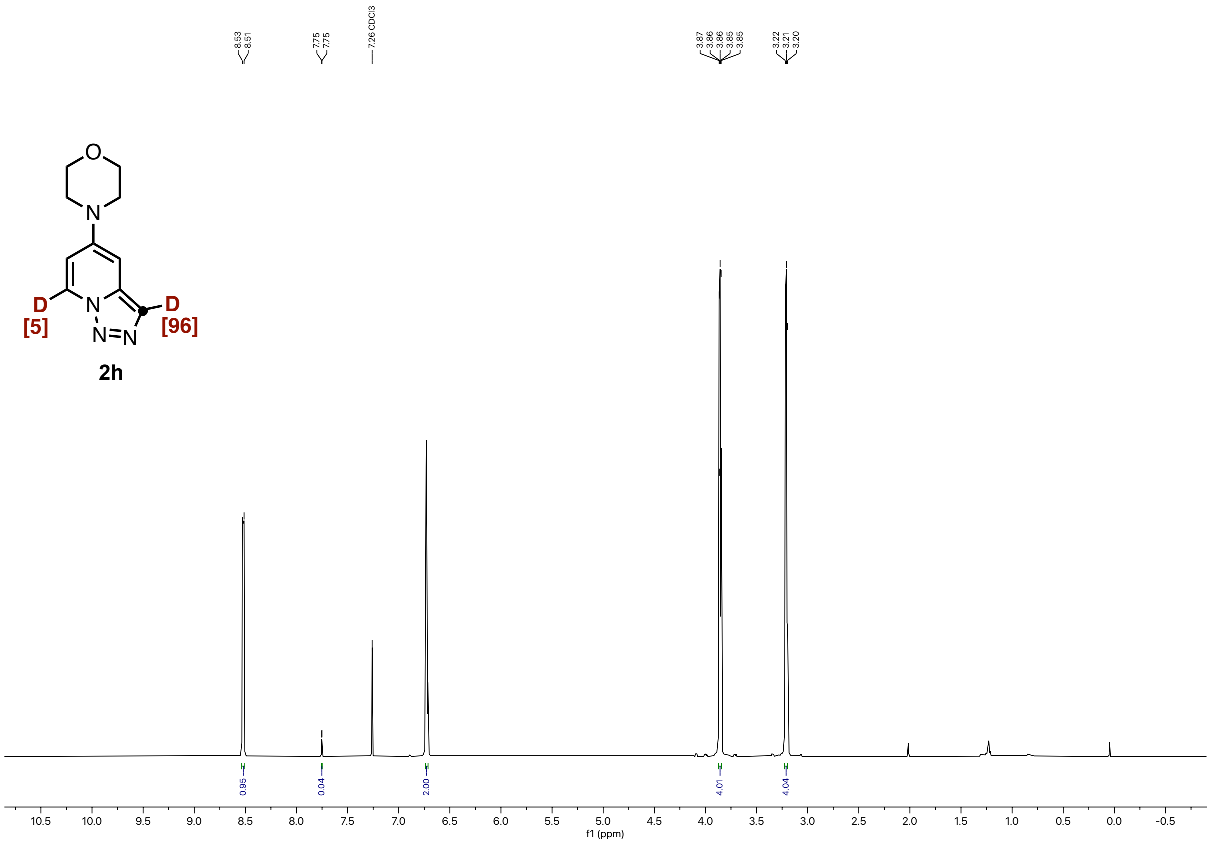

^{1}H spectrum (500 MHz, CD_3Cl) of **2h**

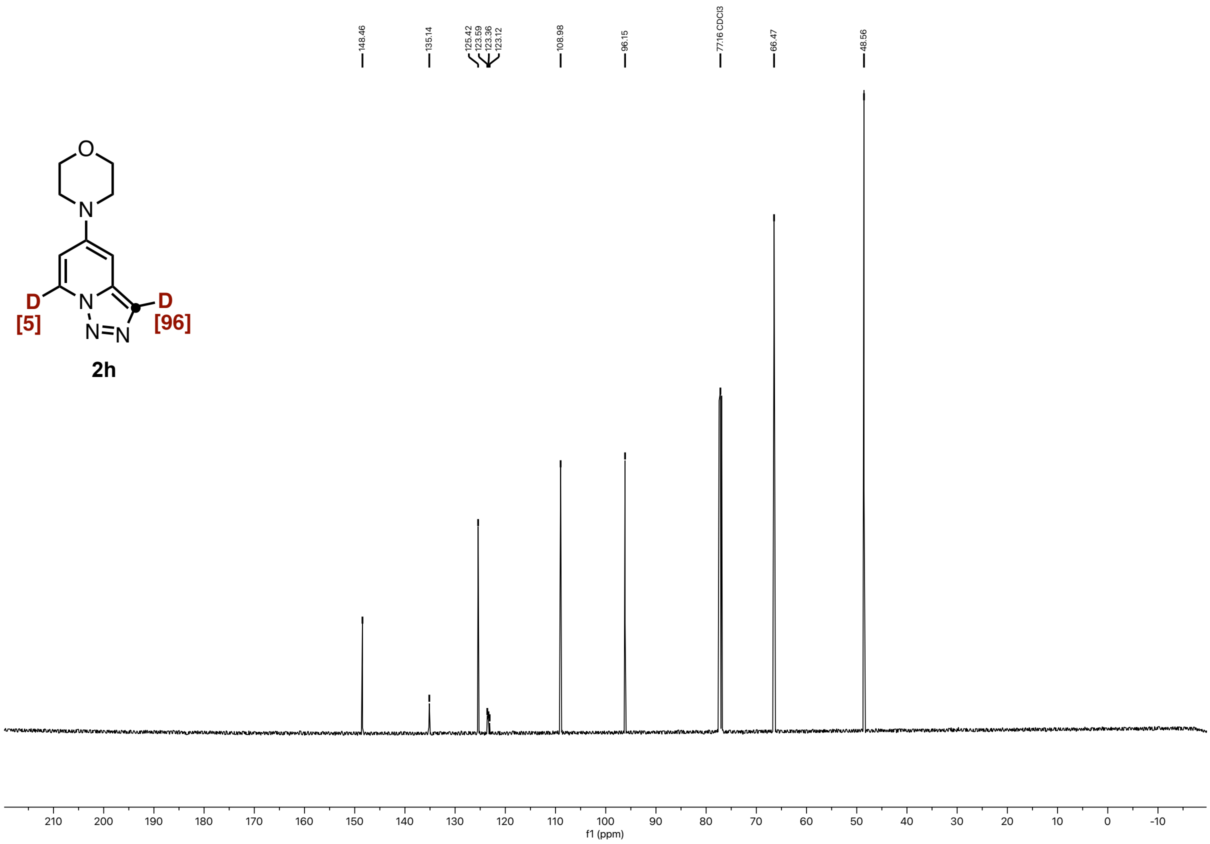

^{13}C spectrum (500 MHz, CD_3Cl) of **2h**

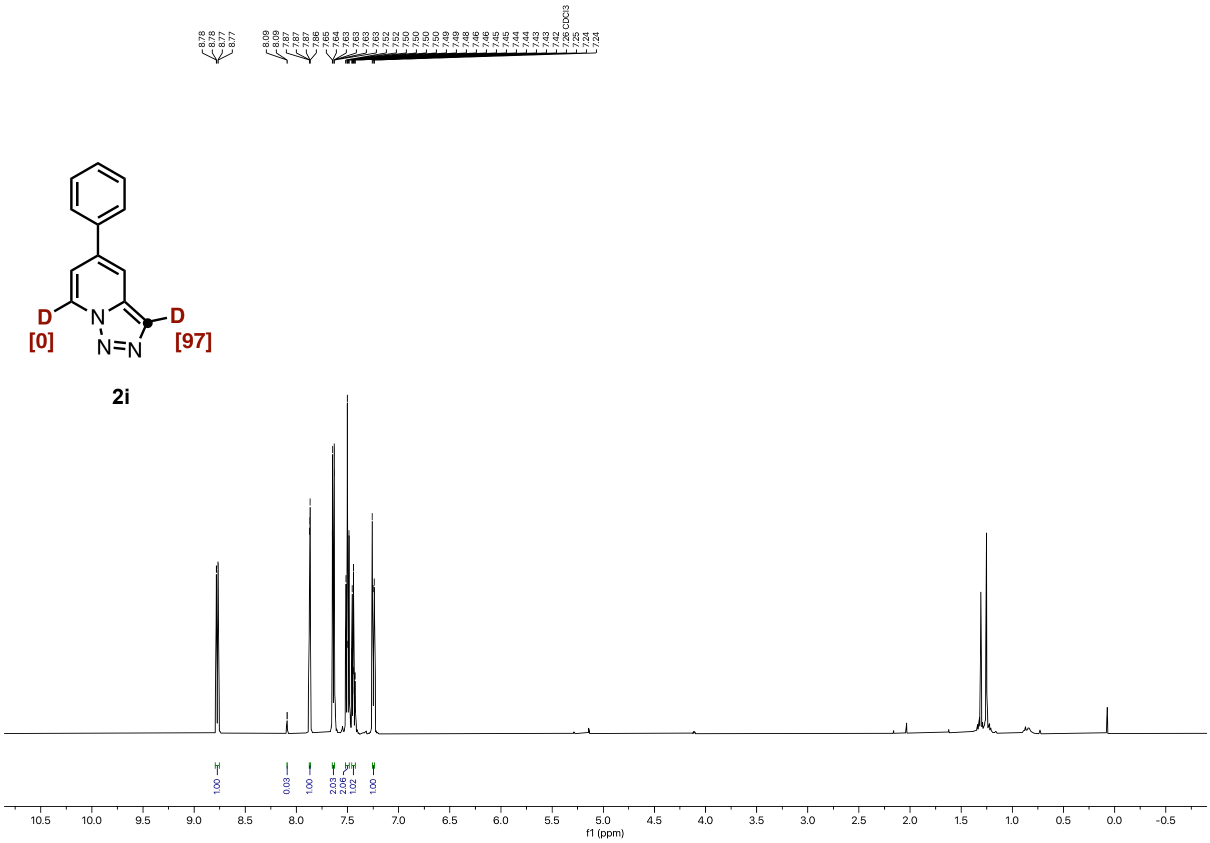

^{1}H spectrum (500 MHz, CD_3Cl) of **2i**

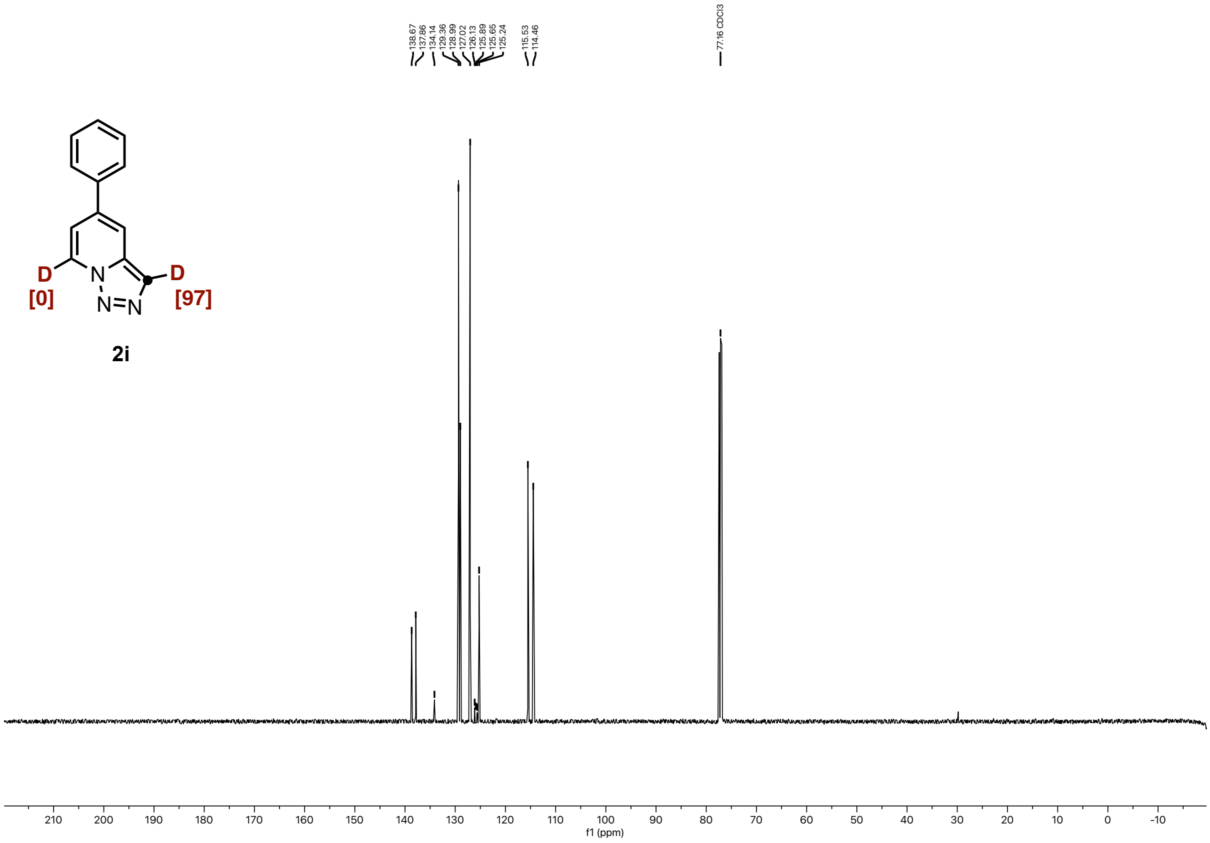

^{13}C spectrum (500 MHz, CD_3Cl) of **2i**

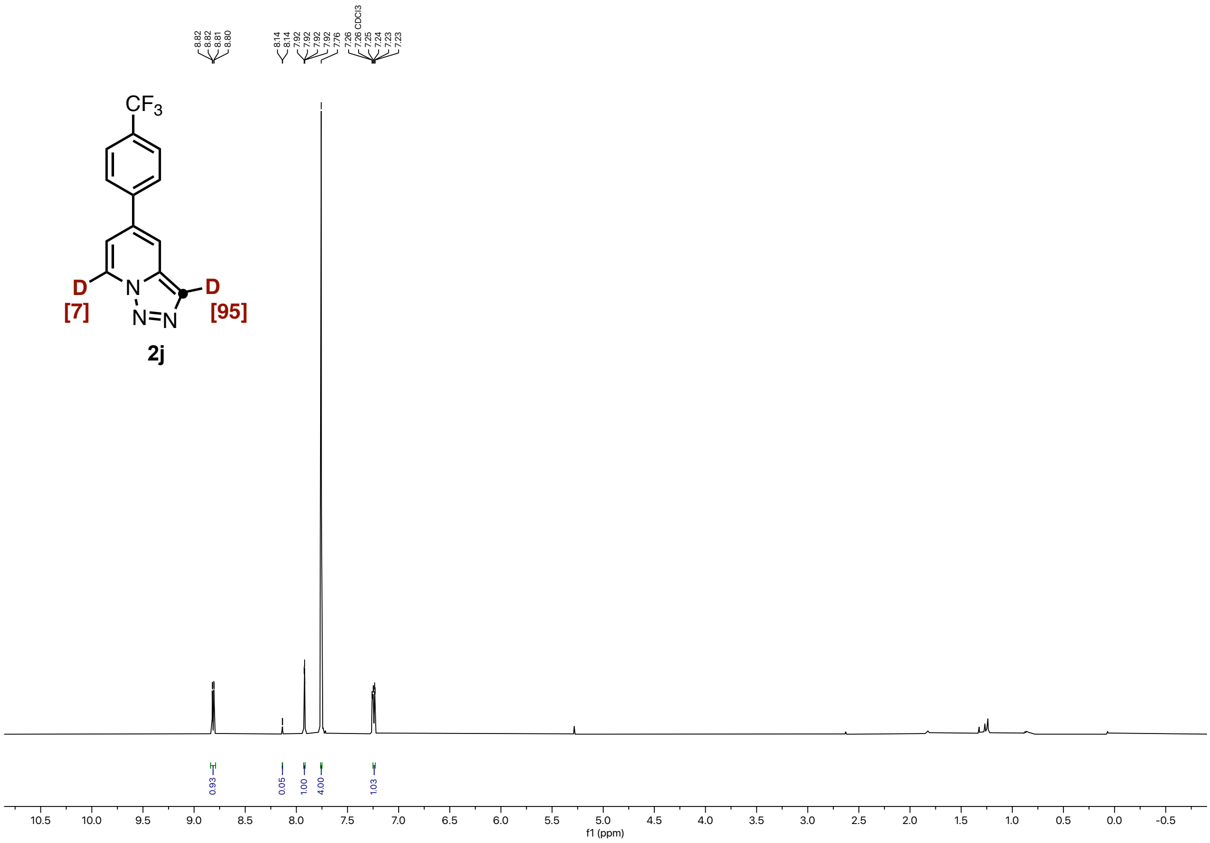

^{1}H spectrum (500 MHz, CD3Cl) of **2j**

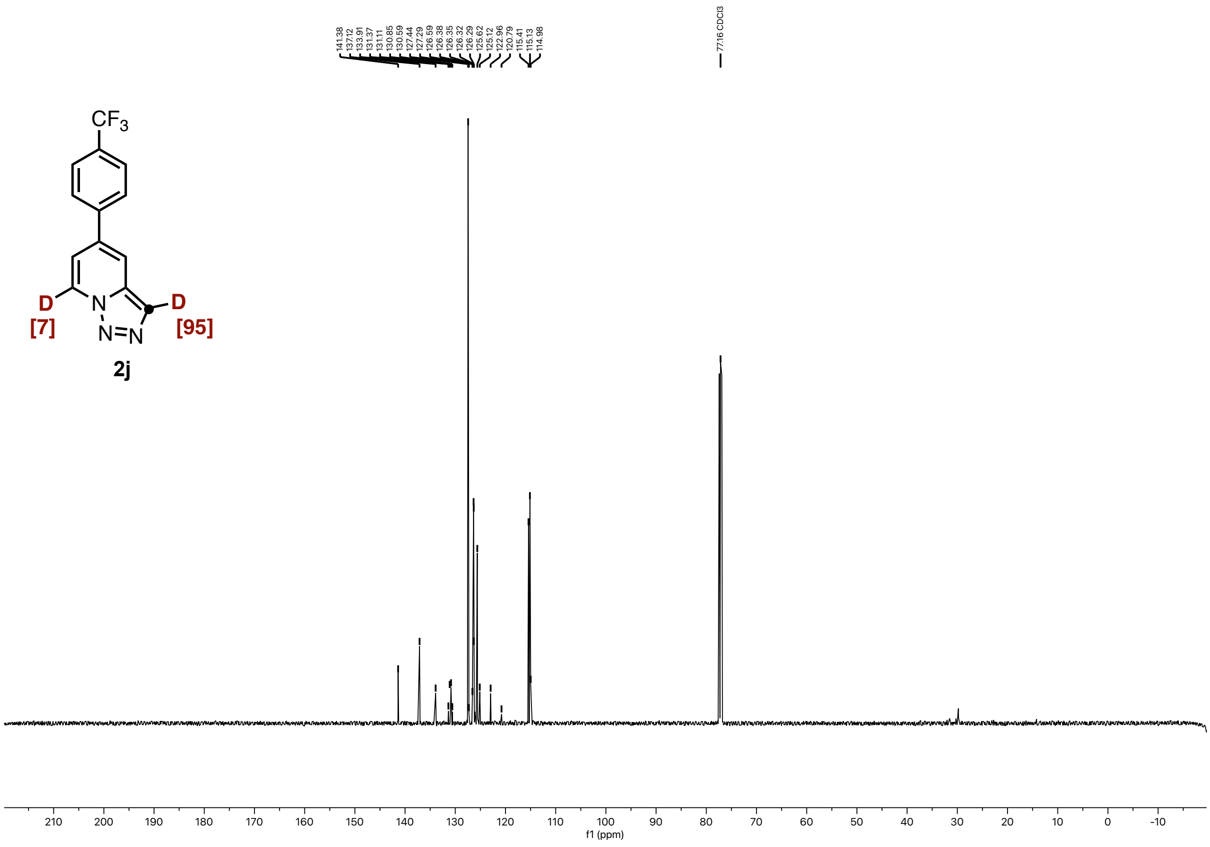

^{13}C spectrum (500 MHz, CD3Cl) of **2j**

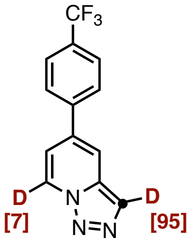

2j

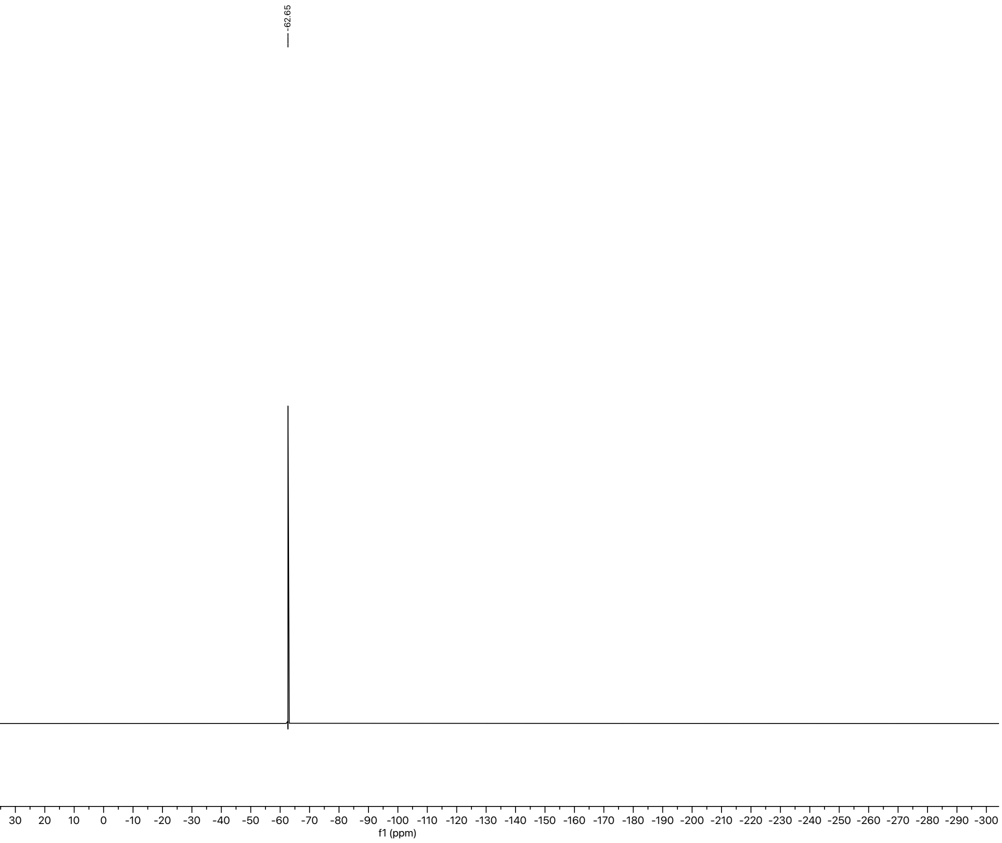

^{19}F spectrum (471 MHz, $CDCl_3$) of **2j**

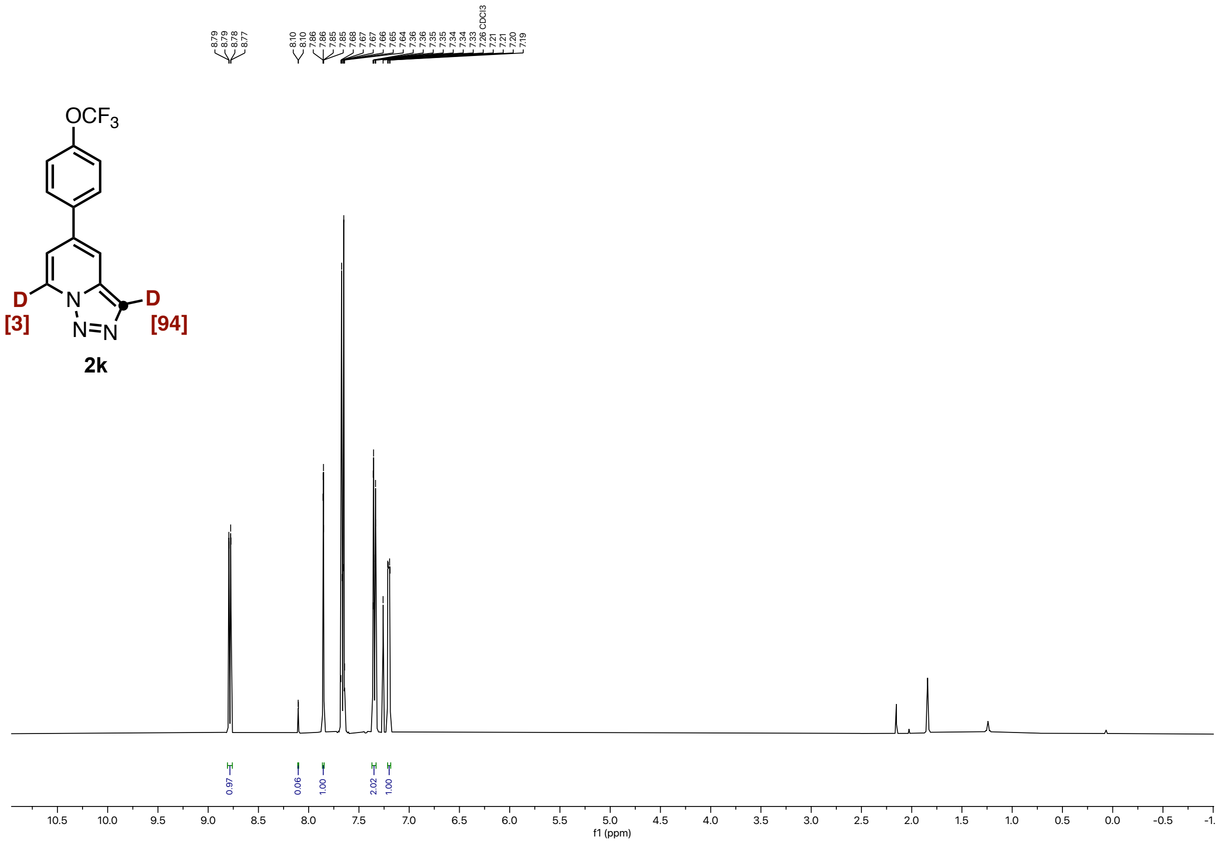

^{1}H spectrum (500 MHz, CD3Cl) of **2k**

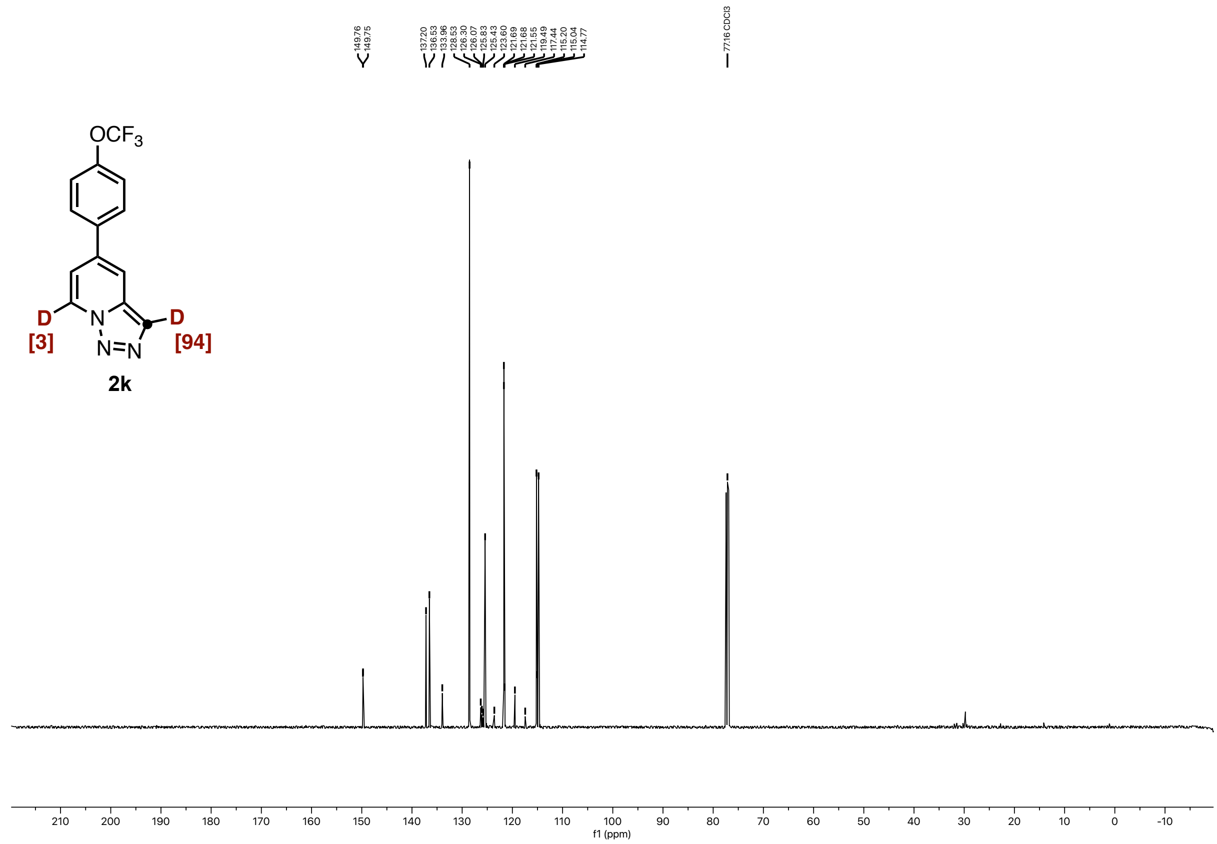

^{13}C spectrum (500 MHz, CD3Cl) of **2k**

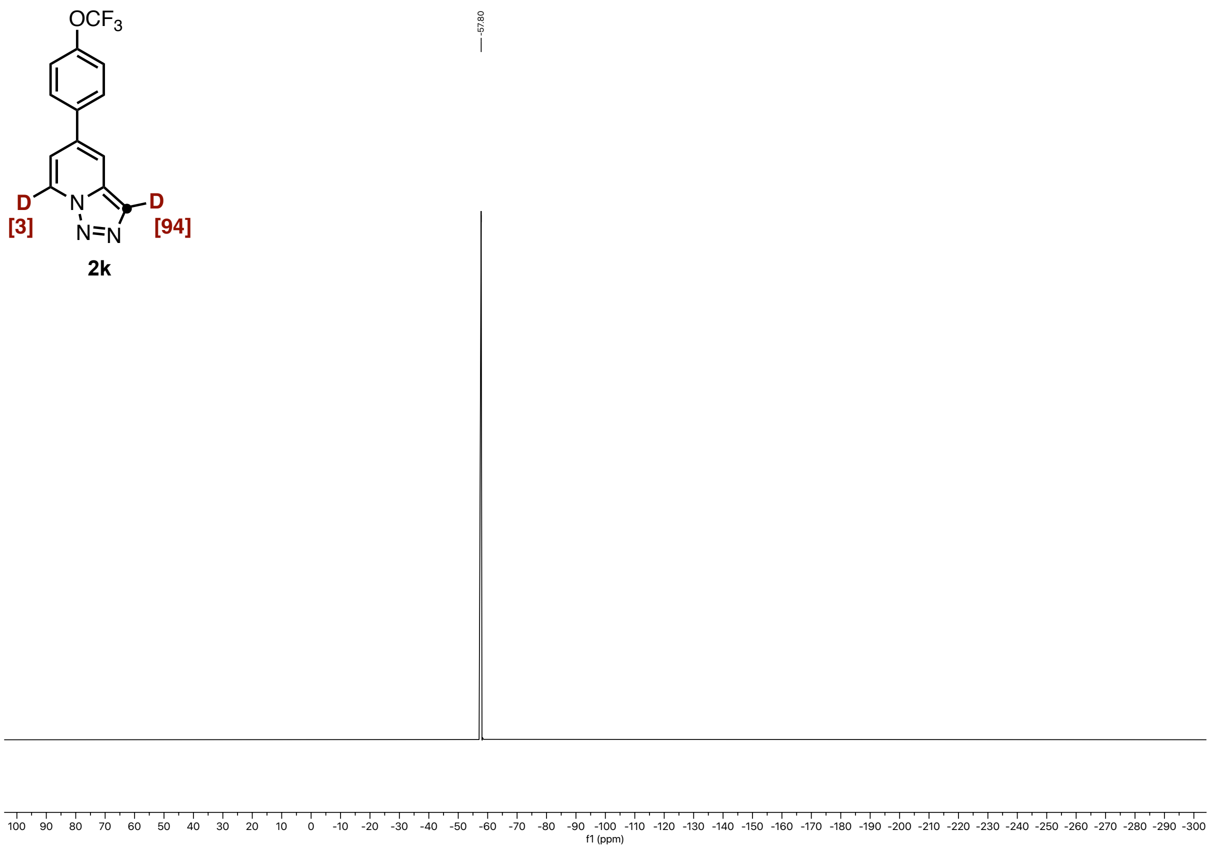

^{19}F spectrum (471 MHz, $CDCl_3$) of **2k**

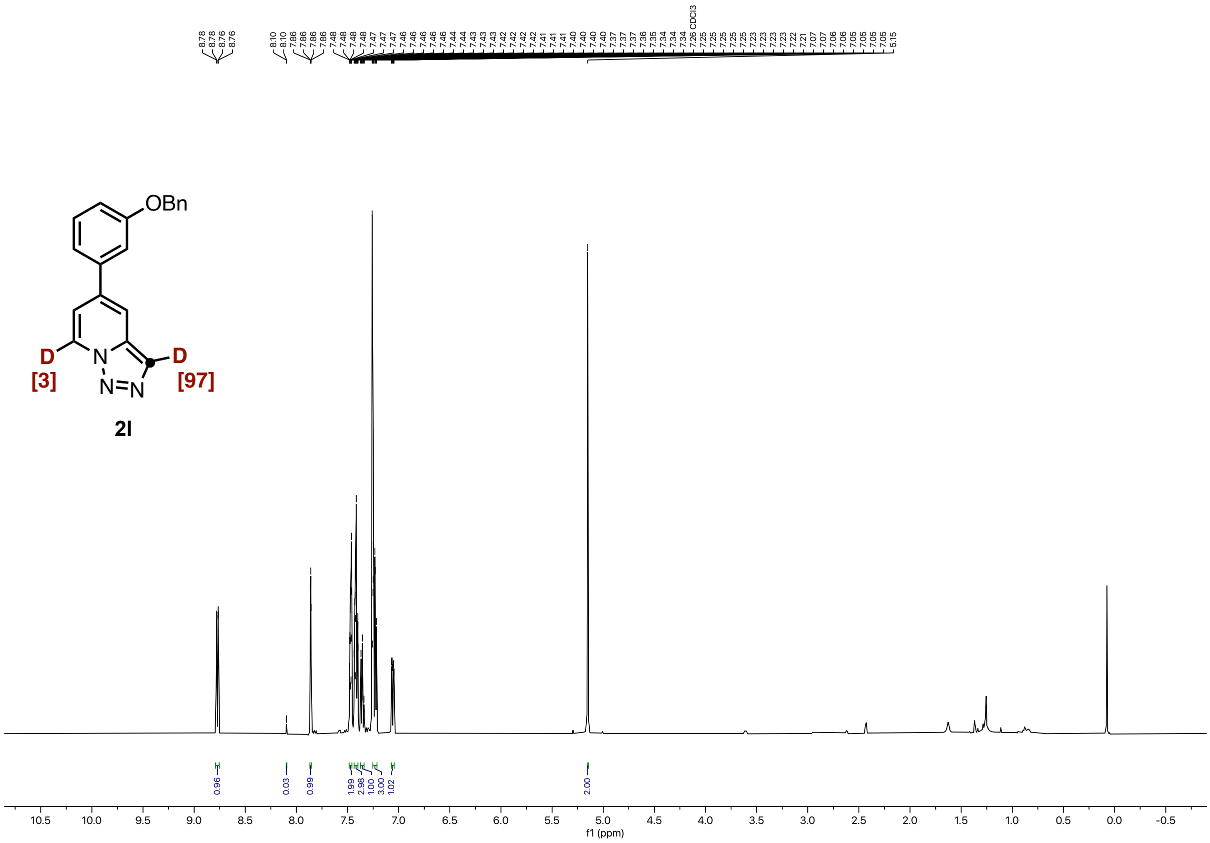

^{1}H spectrum (500 MHz, CD3Cl) of **2l**

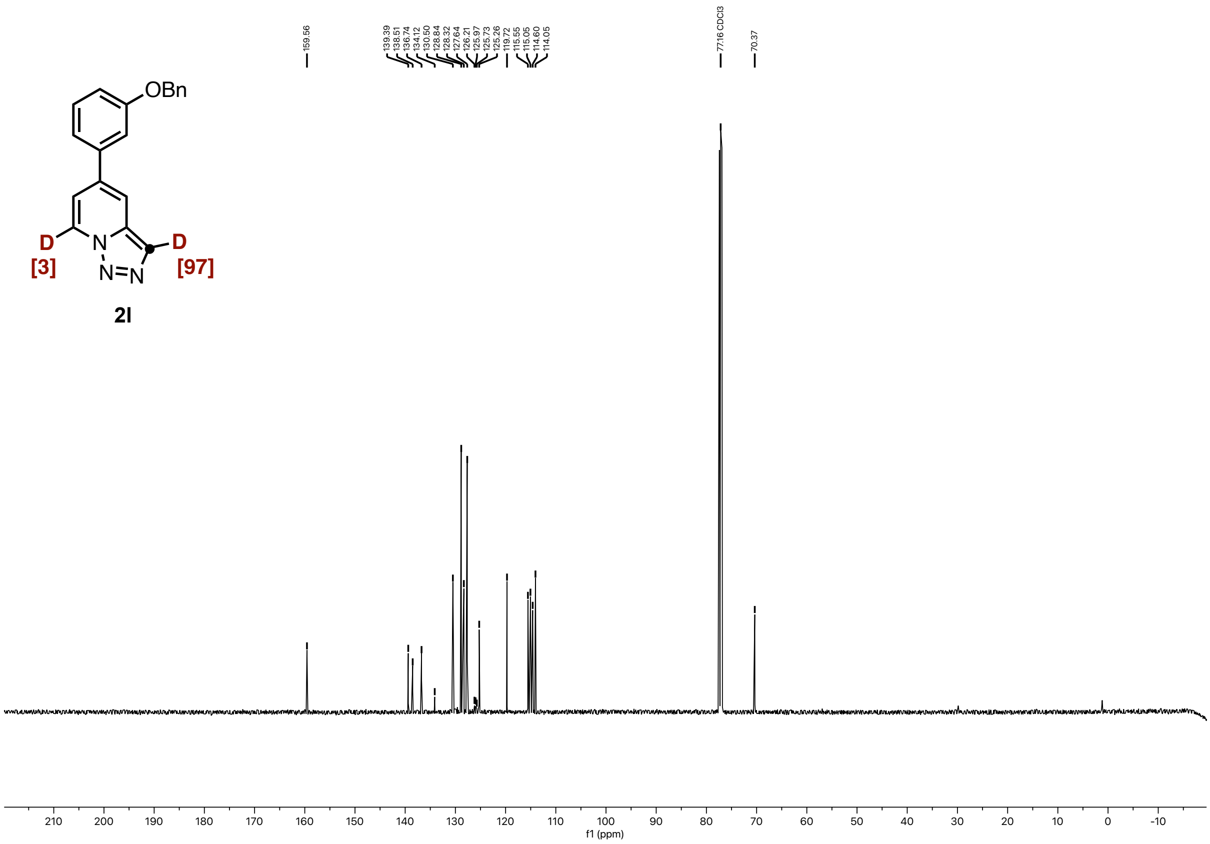

^{13}C spectrum (500 MHz, CD3Cl) of **2l**

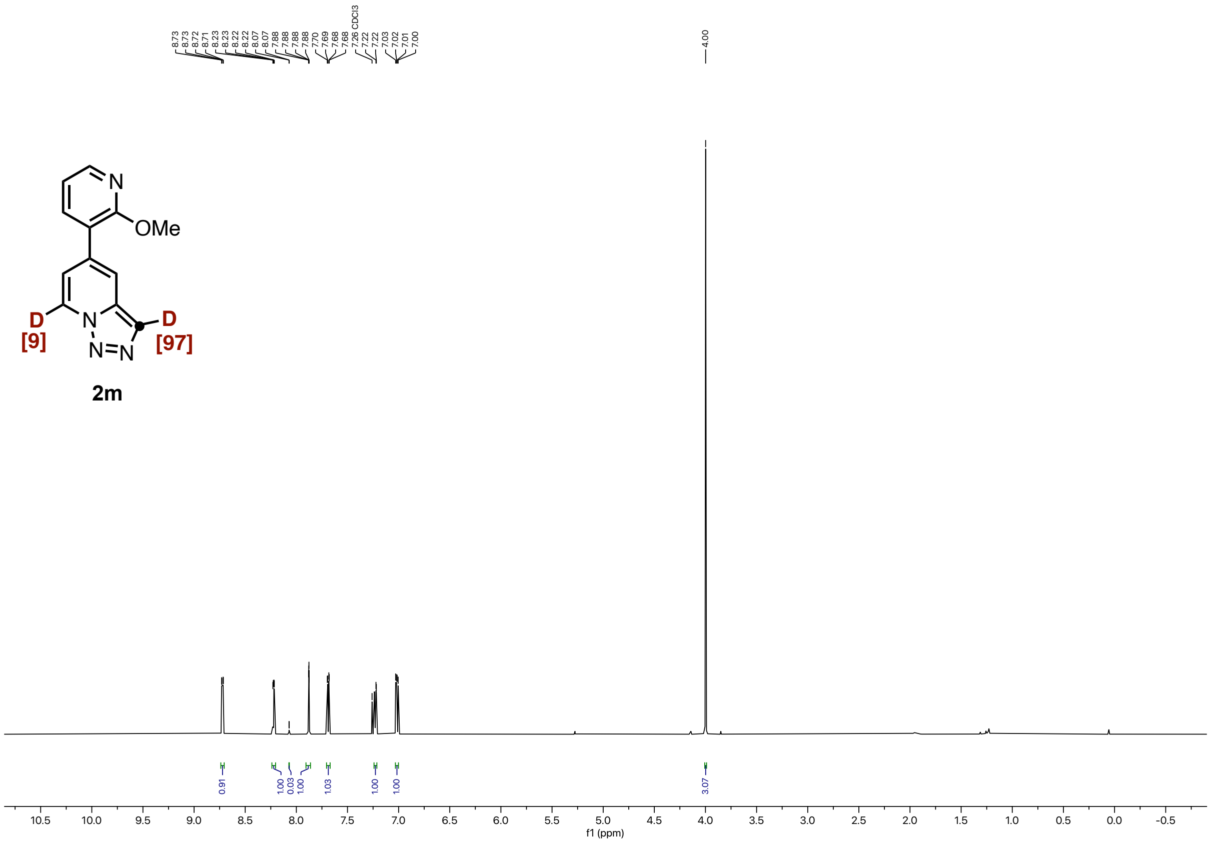

^{1}H spectrum (500 MHz, CD_3Cl) of **2m**

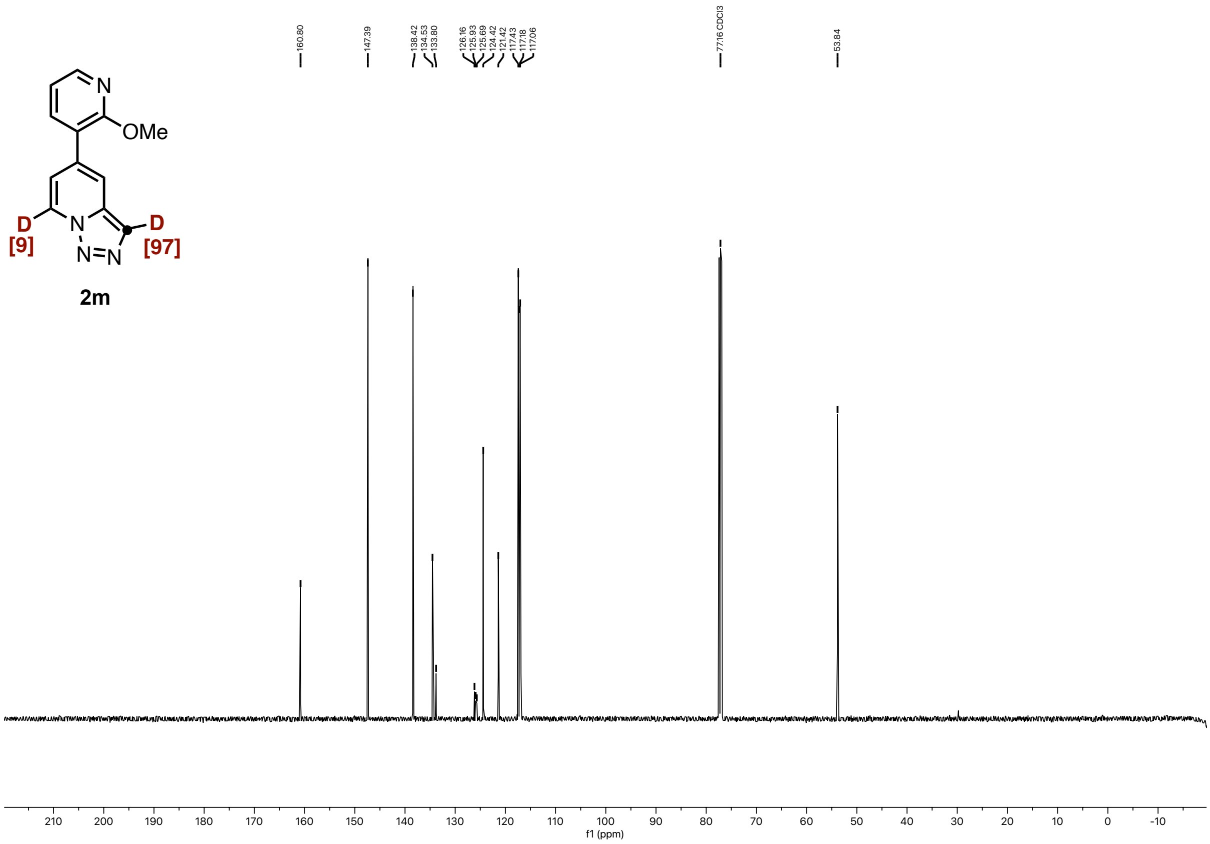

^{13}C spectrum (500 MHz, CD_3Cl) of **2m**

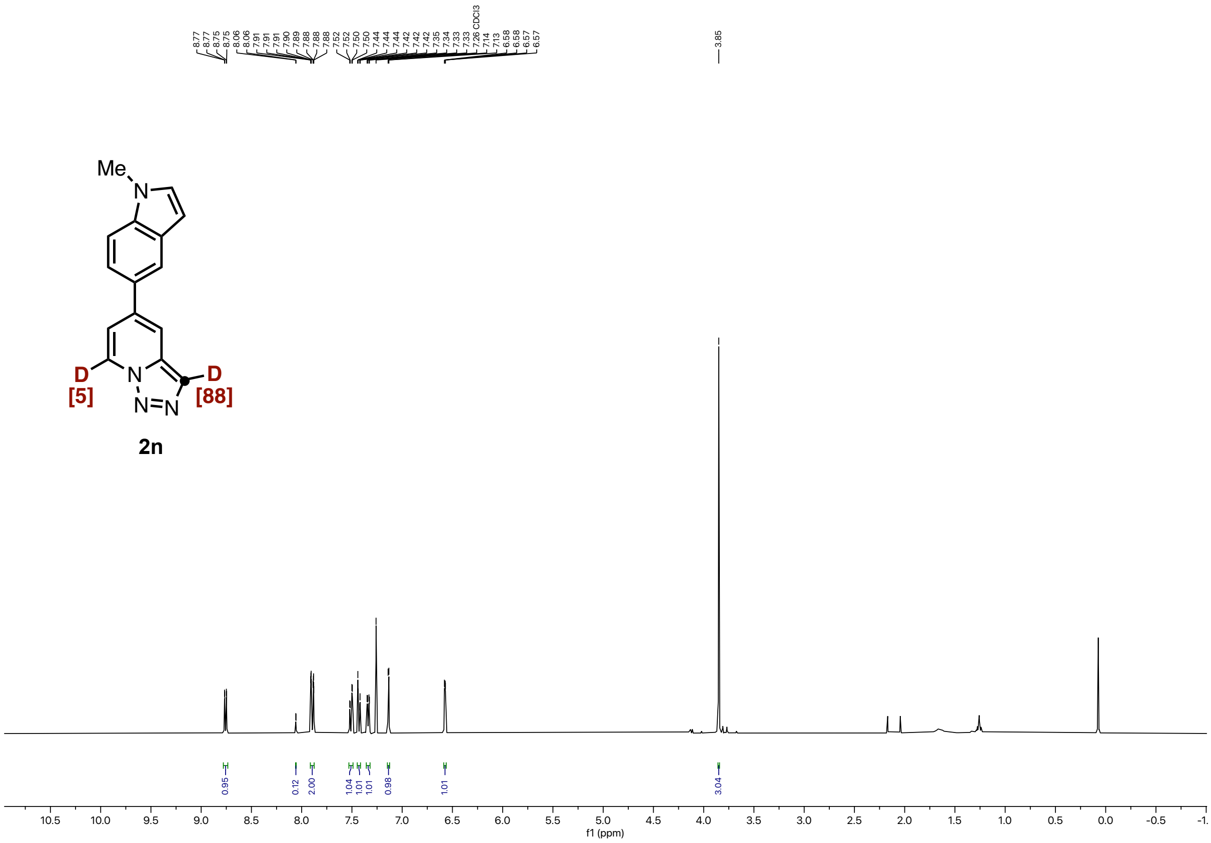

^{1}H spectrum (500 MHz, CD3Cl) of **2n**

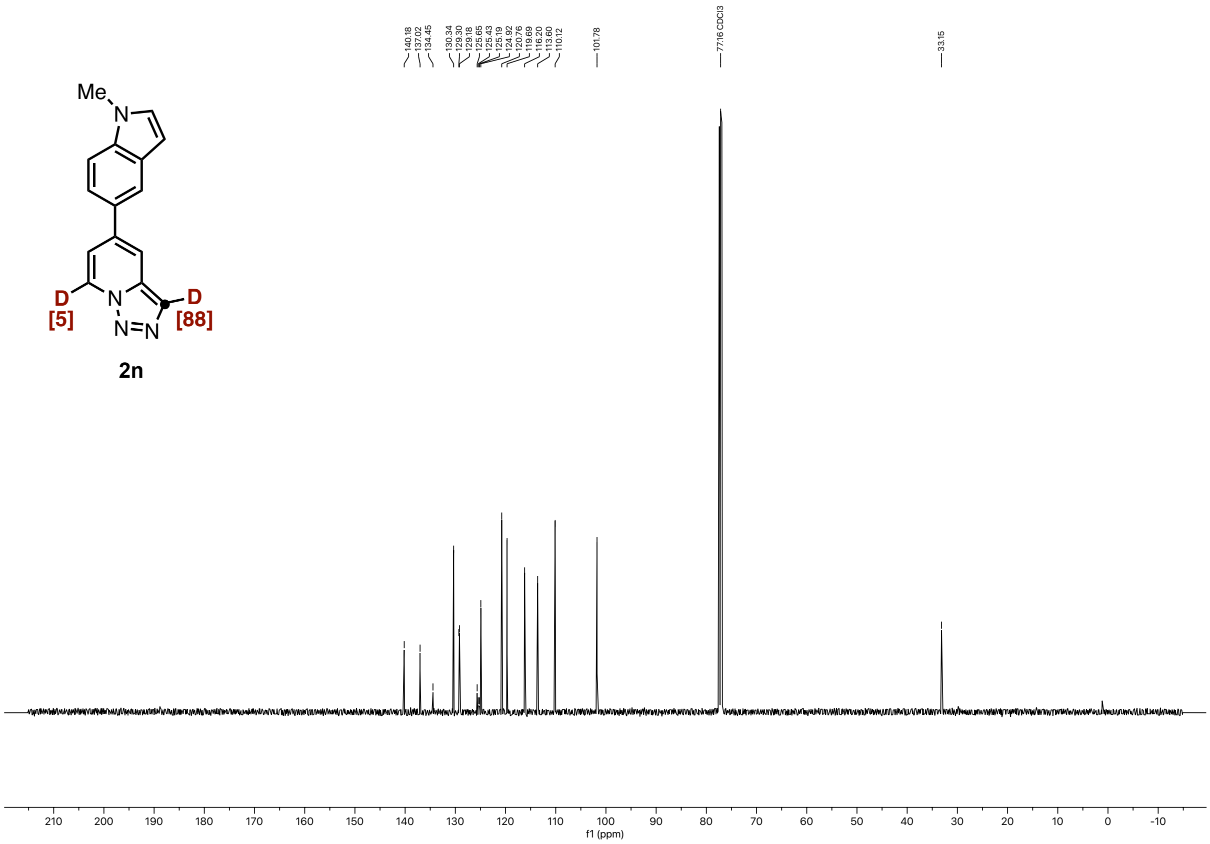

^{13}C spectrum (500 MHz, CD3Cl) of **2n**

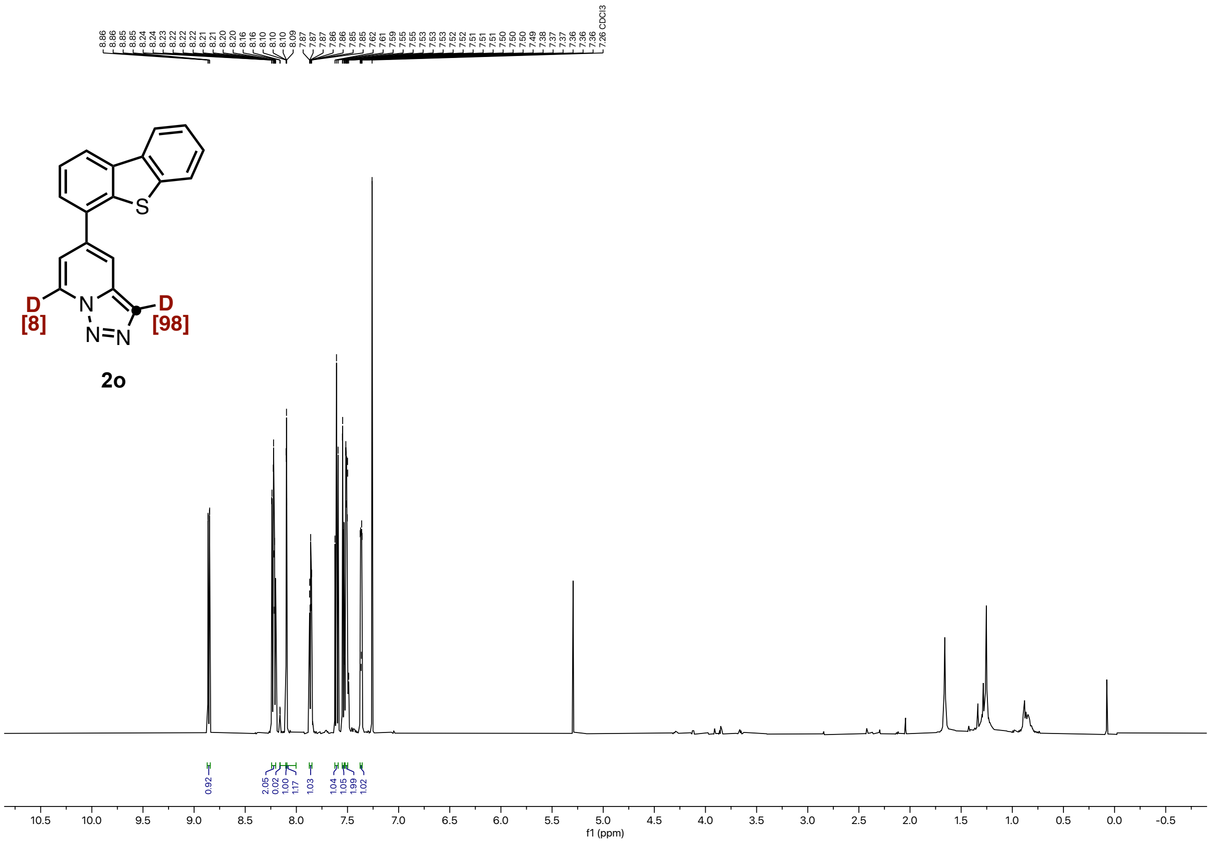

^{1}H spectrum (500 MHz, CD3Cl) of **2o**

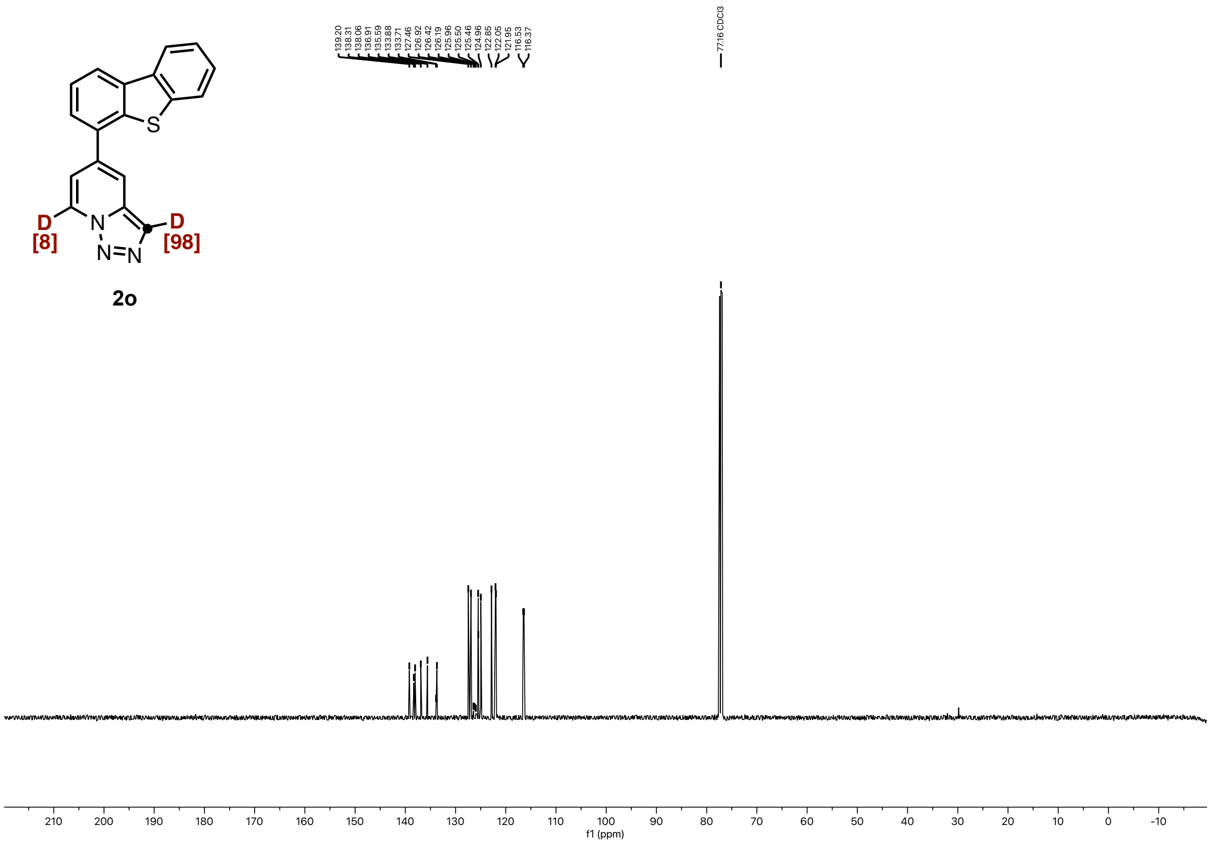

^{13}C spectrum (500 MHz, CD3Cl) of **2o**

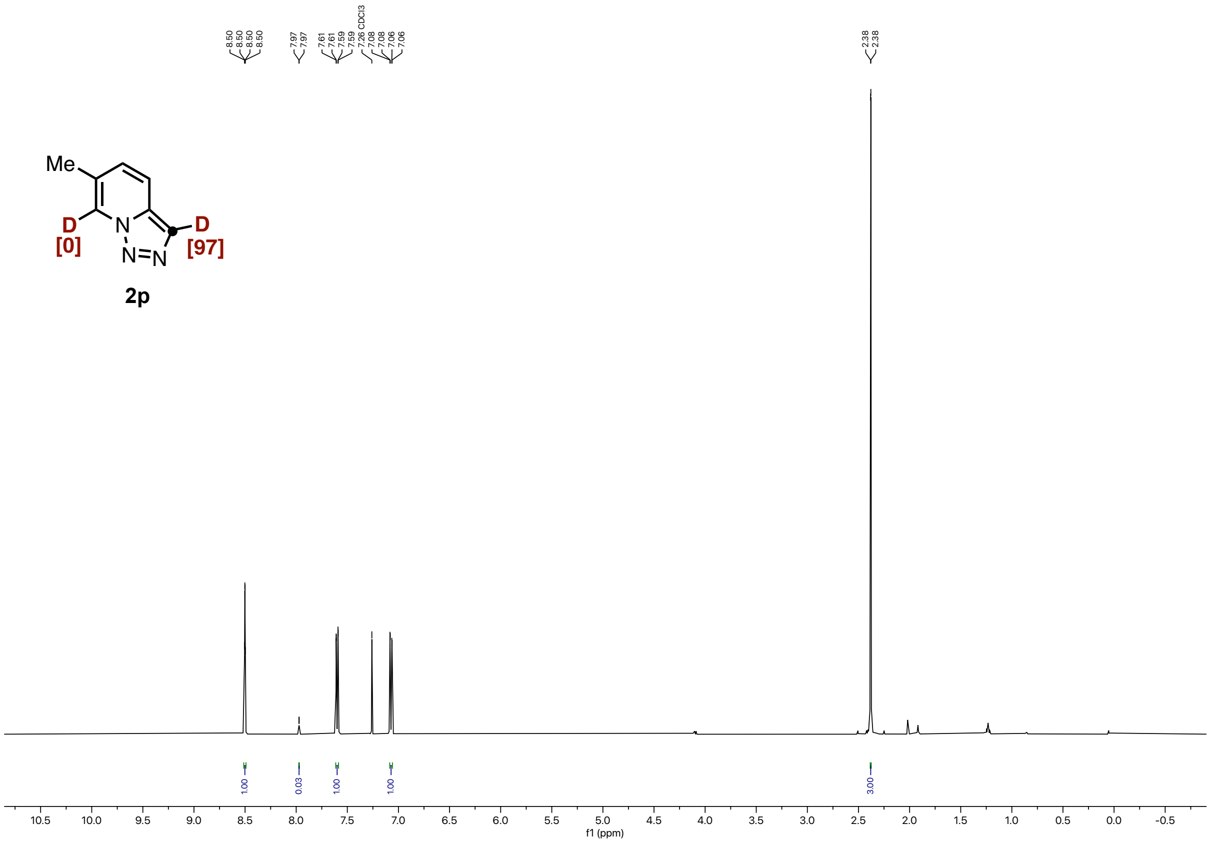

^{1}H spectrum (500 MHz, CD3Cl) of **2p**

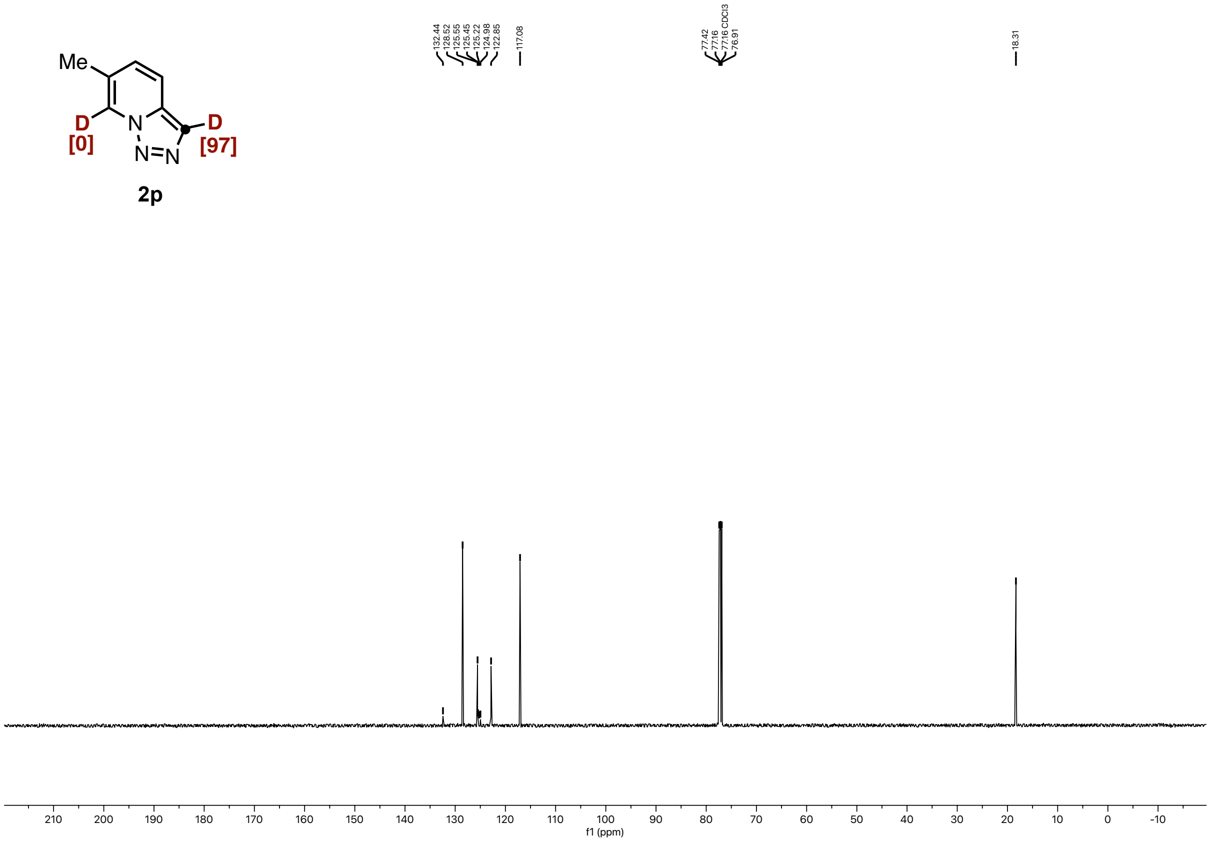

^{13}C spectrum (126 MHz, CD3Cl) of **2p**

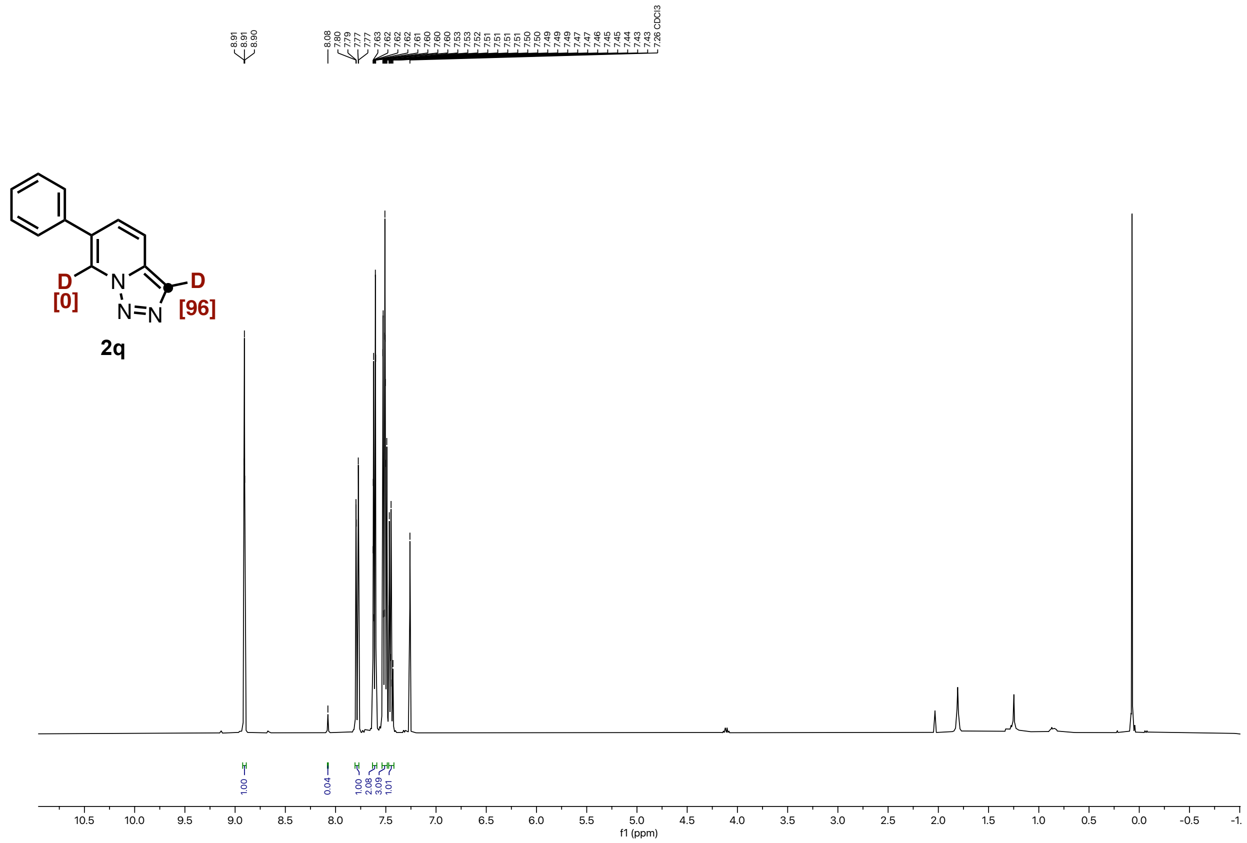

^{1}H spectrum (500 MHz, CD3Cl) of **2q**

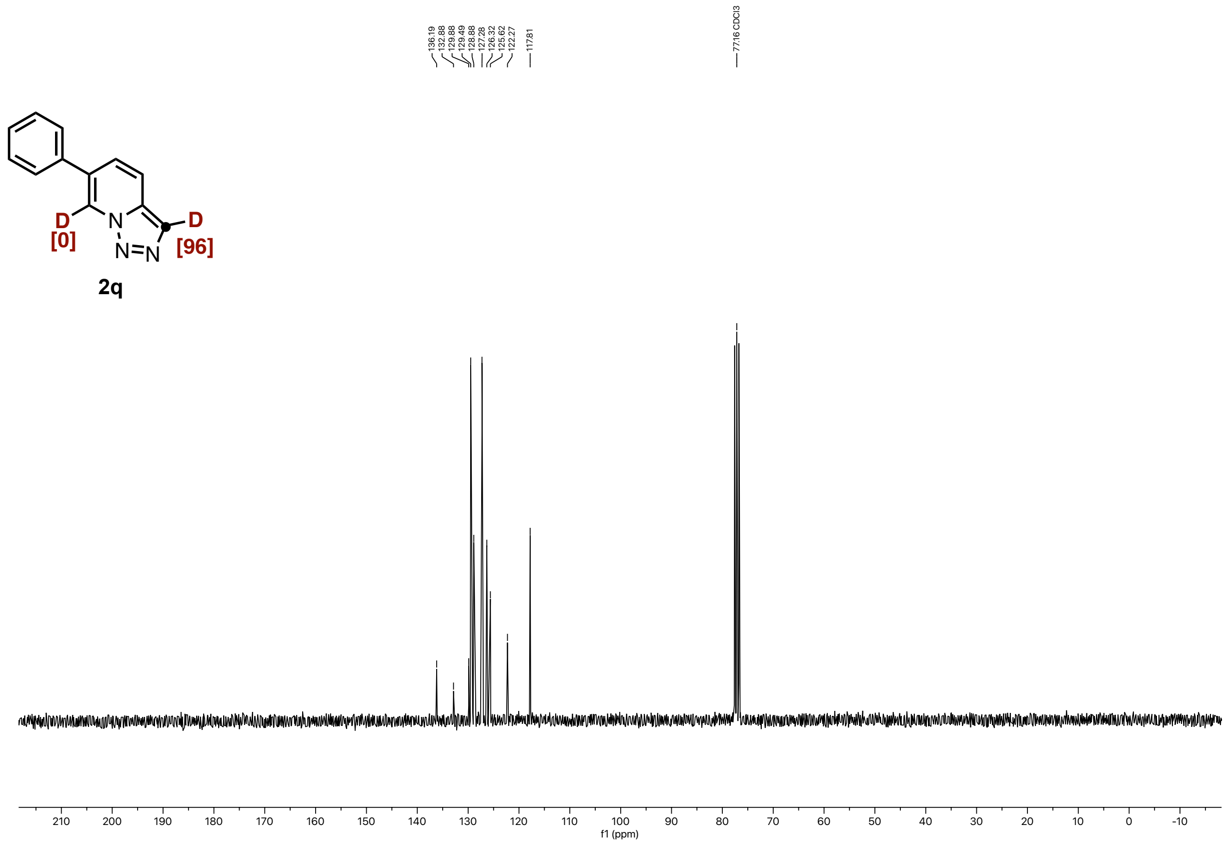

^{13}C spectrum (75 MHz, CD3Cl) of **2q**

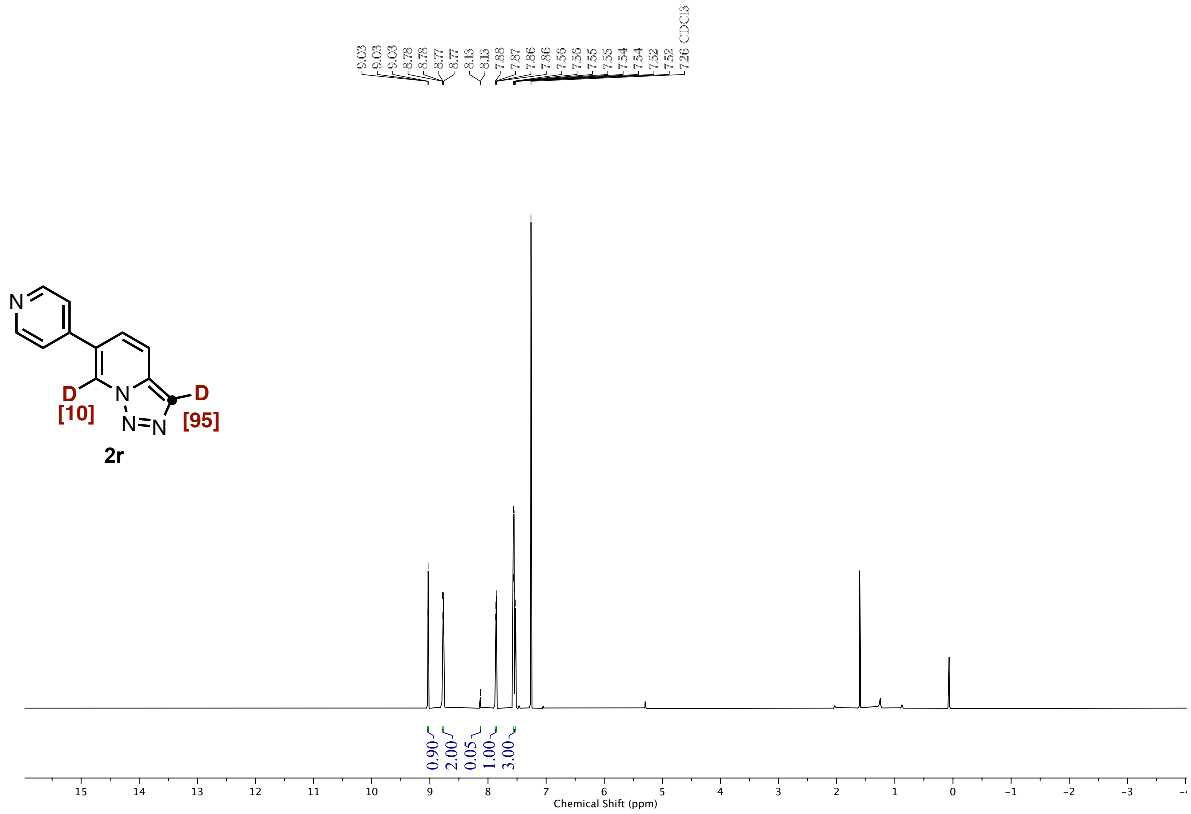

^{1}H spectrum (300 MHz, CD_3Cl) of **2r**

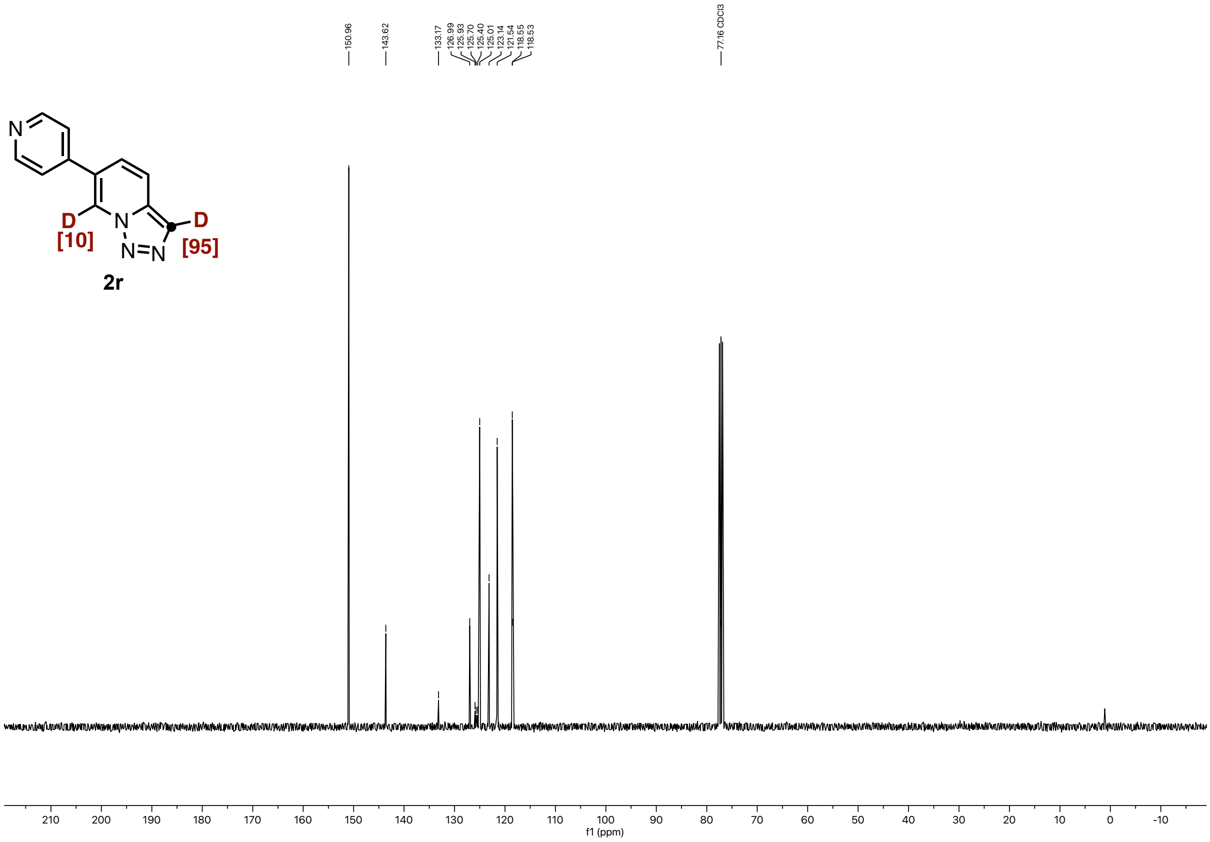

^{13}C spectrum (101 MHz, CD_3Cl) of **2r**

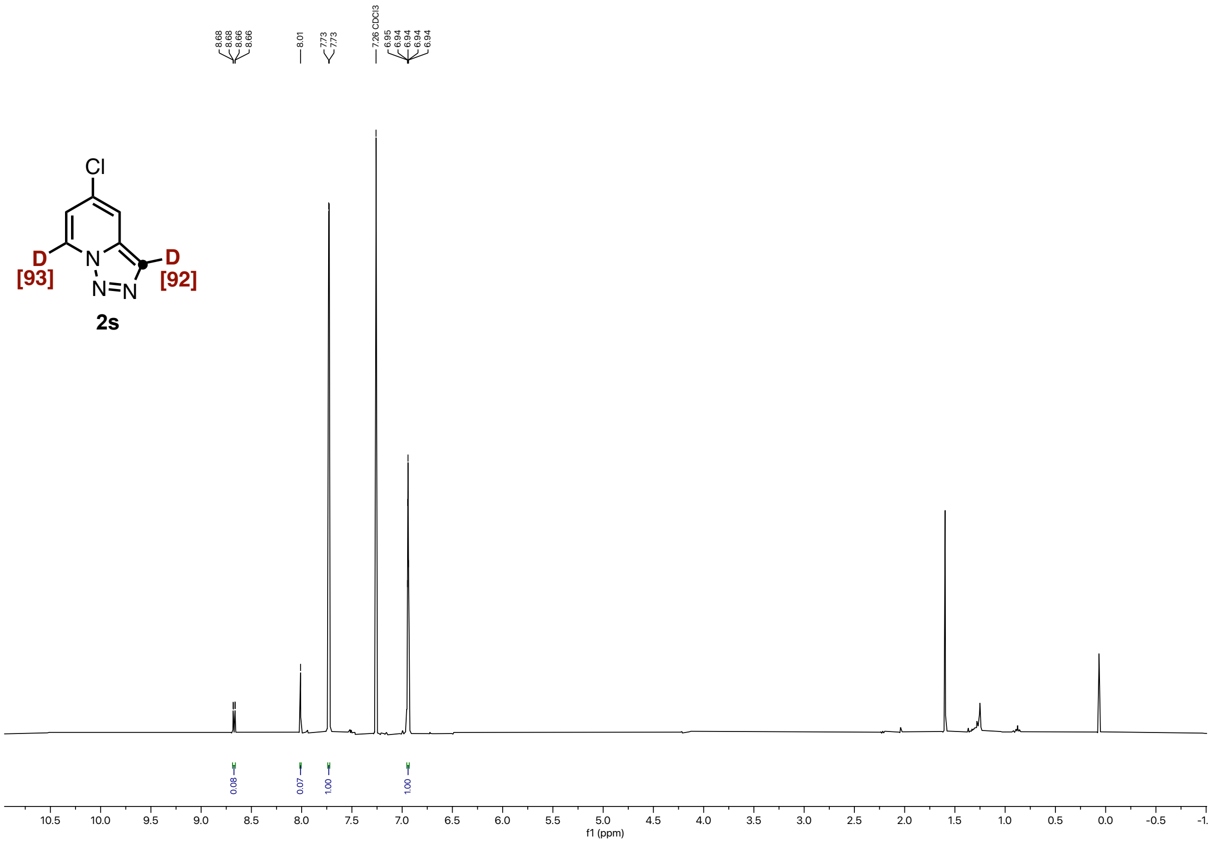

^{1}H spectrum (400 MHz, CD_3Cl) of **2s**

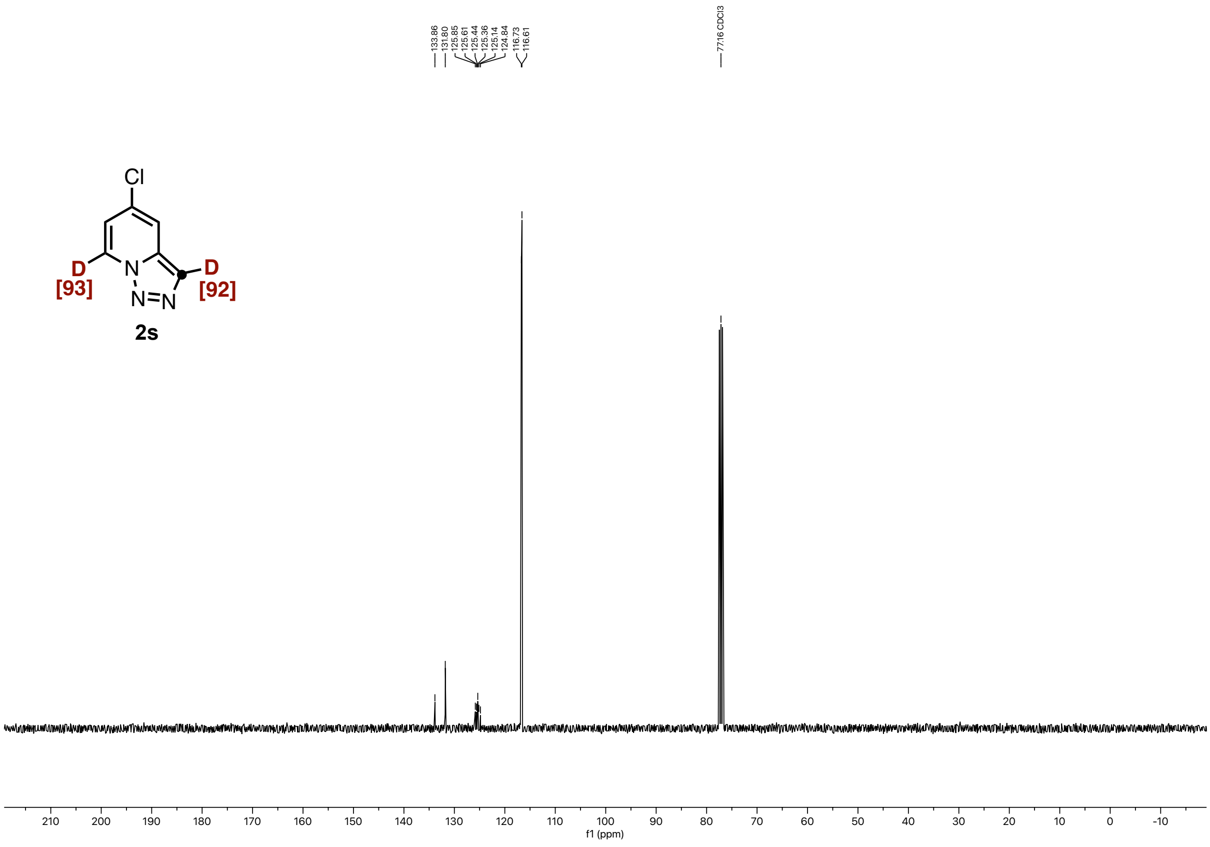

^{13}C spectrum (101 MHz, CD_3Cl) of **2s**

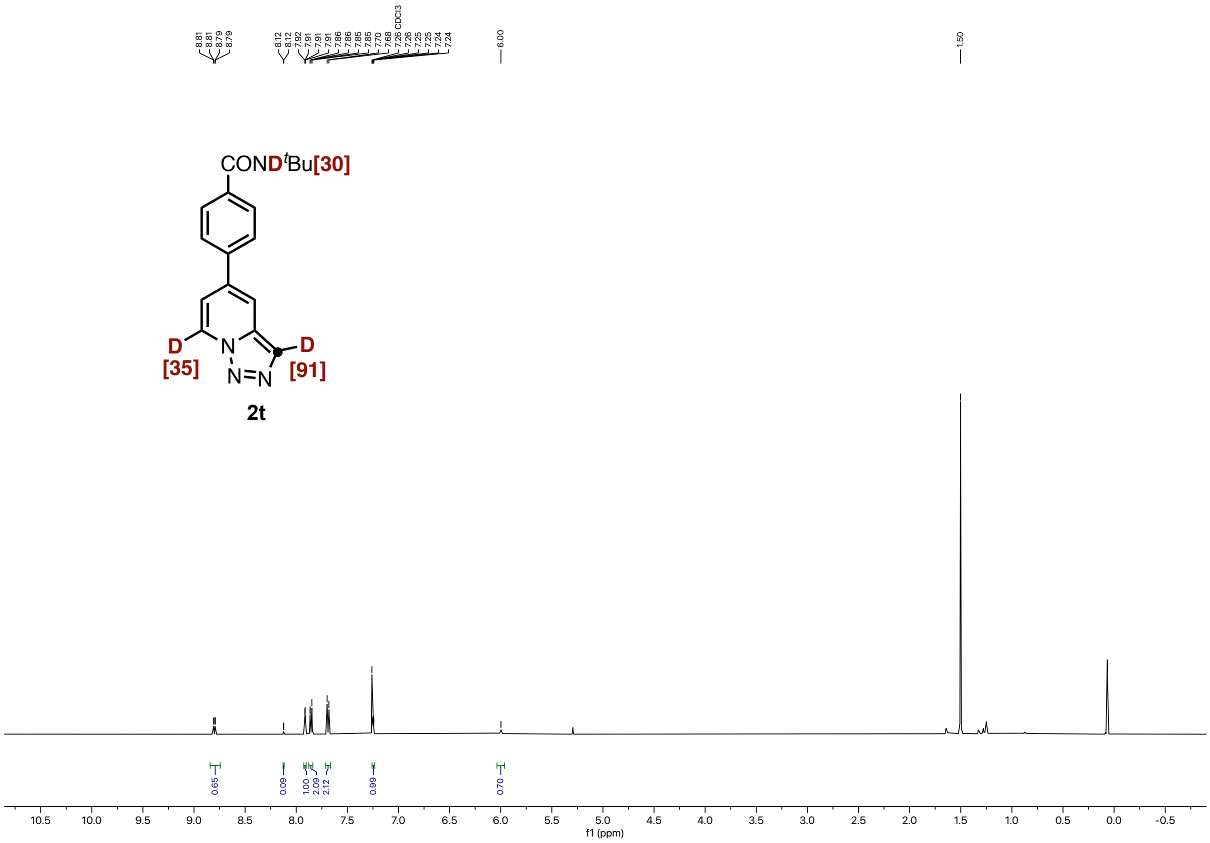

^{1}H spectrum (500 MHz, CD_3Cl) of **2t**

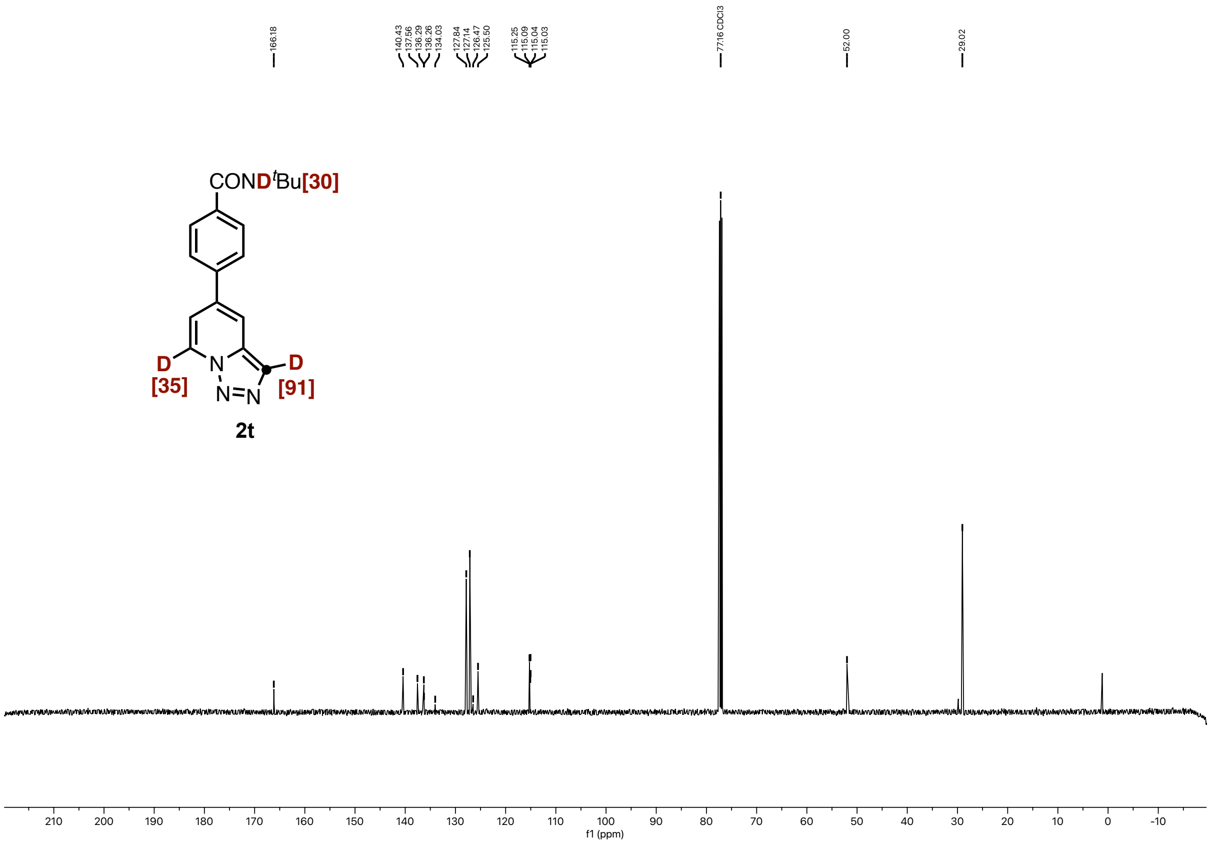

^{13}C spectrum (126 MHz, CD_3Cl) of **2t**

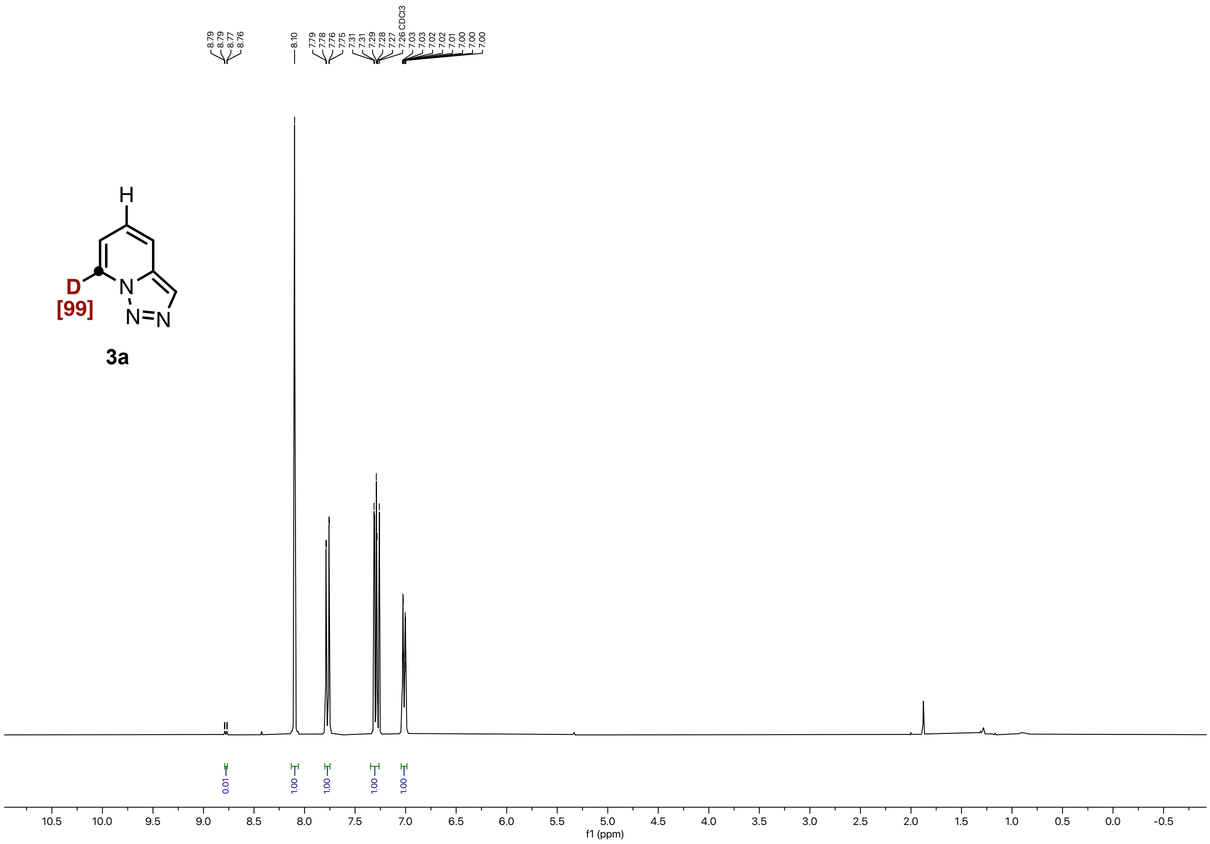

^{1}H spectrum (300 MHz, CD_3Cl) of **3a**

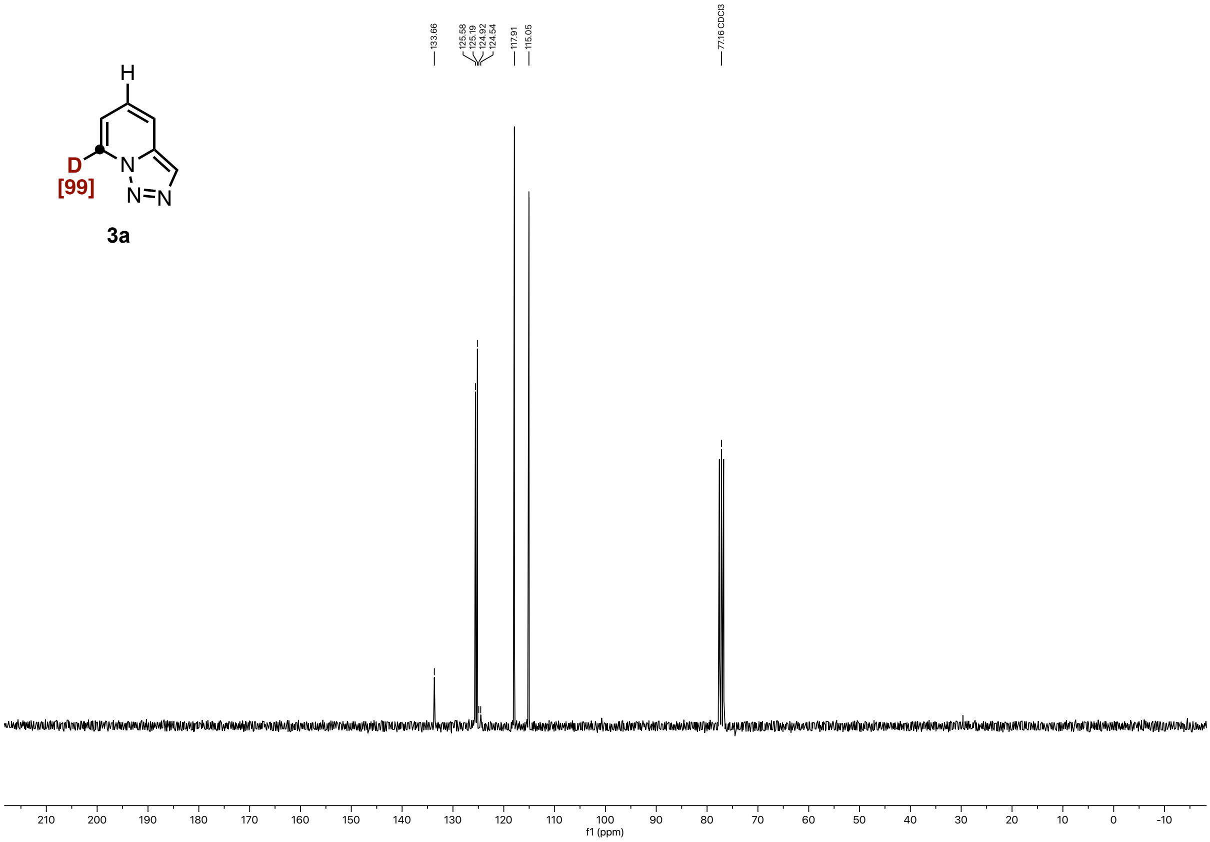

^{13}C spectrum (75 MHz, CD_3Cl) of **3a**

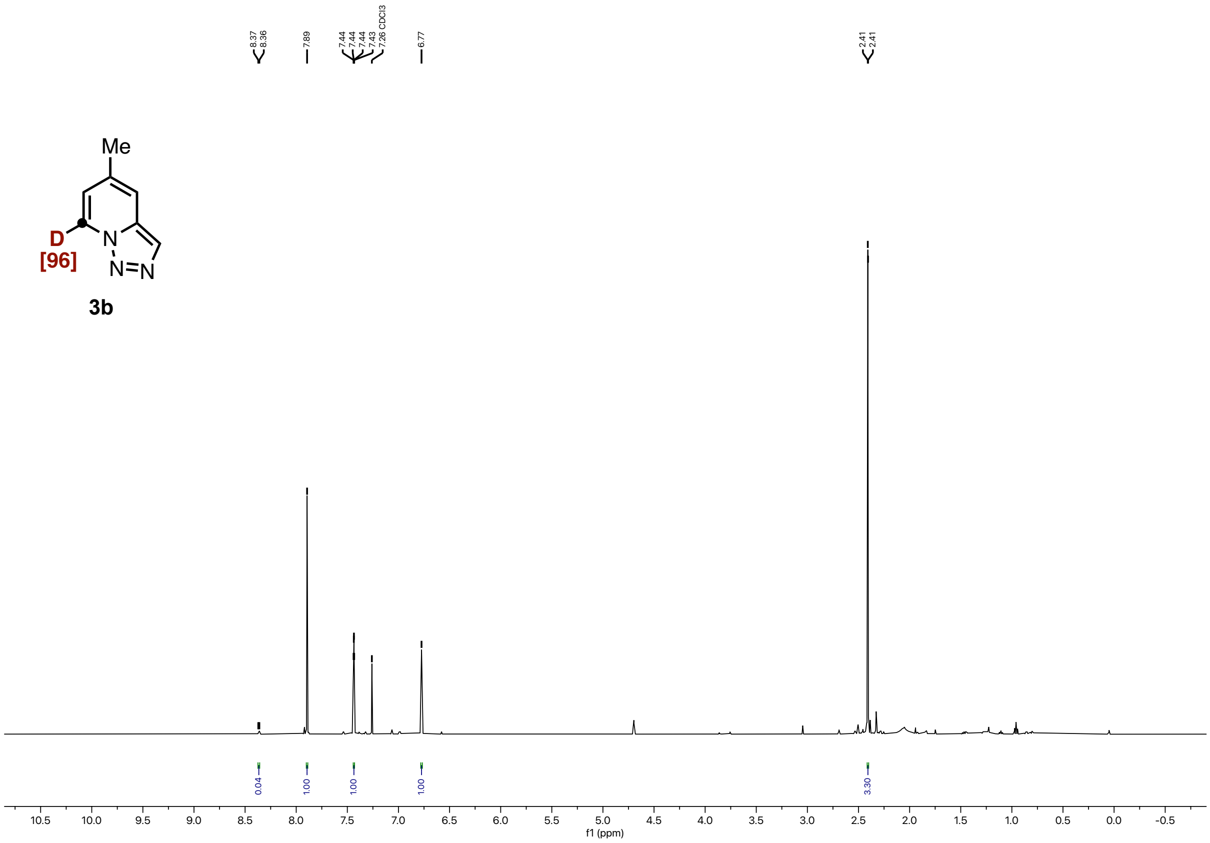

^{1}H spectrum (500 MHz, CD_3Cl) of **3b**

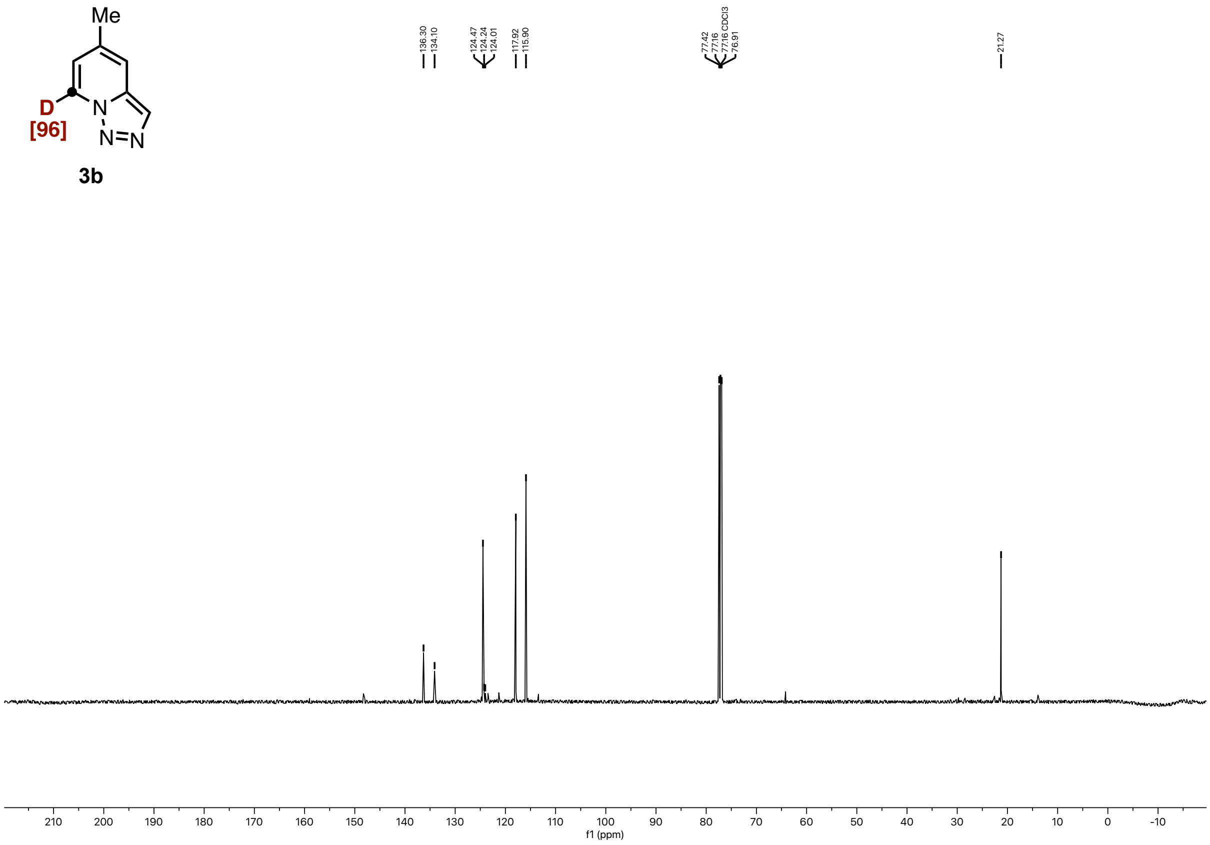

^{13}C spectrum (500 MHz, CD_3Cl) of **3b**

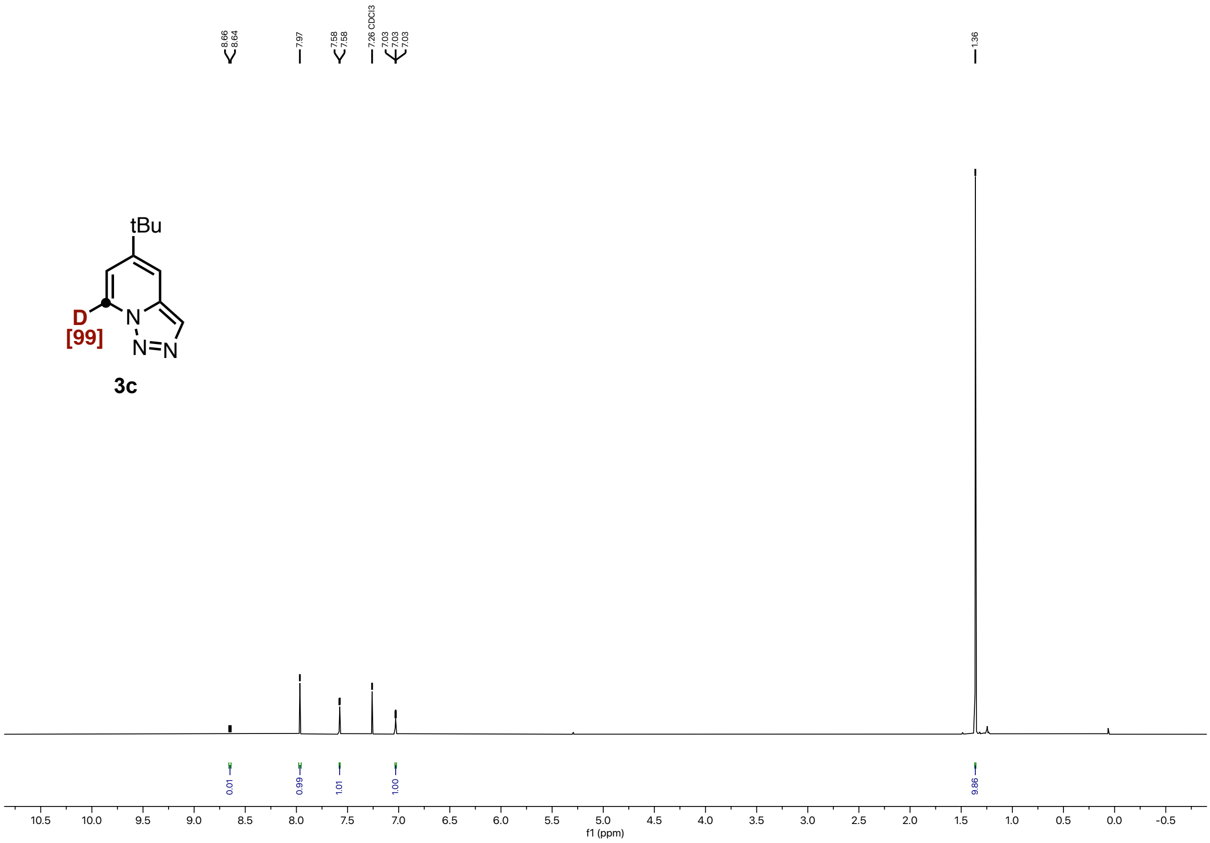

^{1}H spectrum (500 MHz, CD3Cl) of **3c**

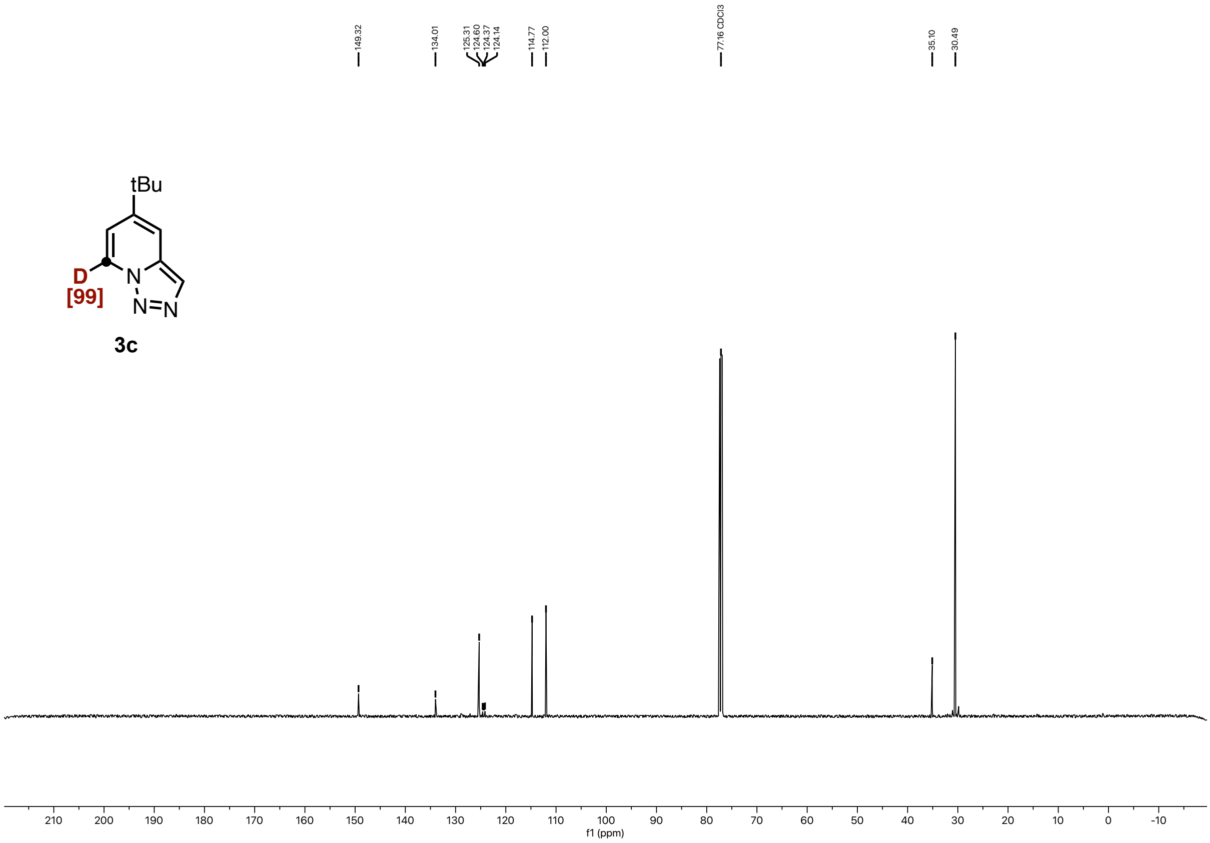

^{13}C spectrum (500 MHz, CD3Cl) of **3c**

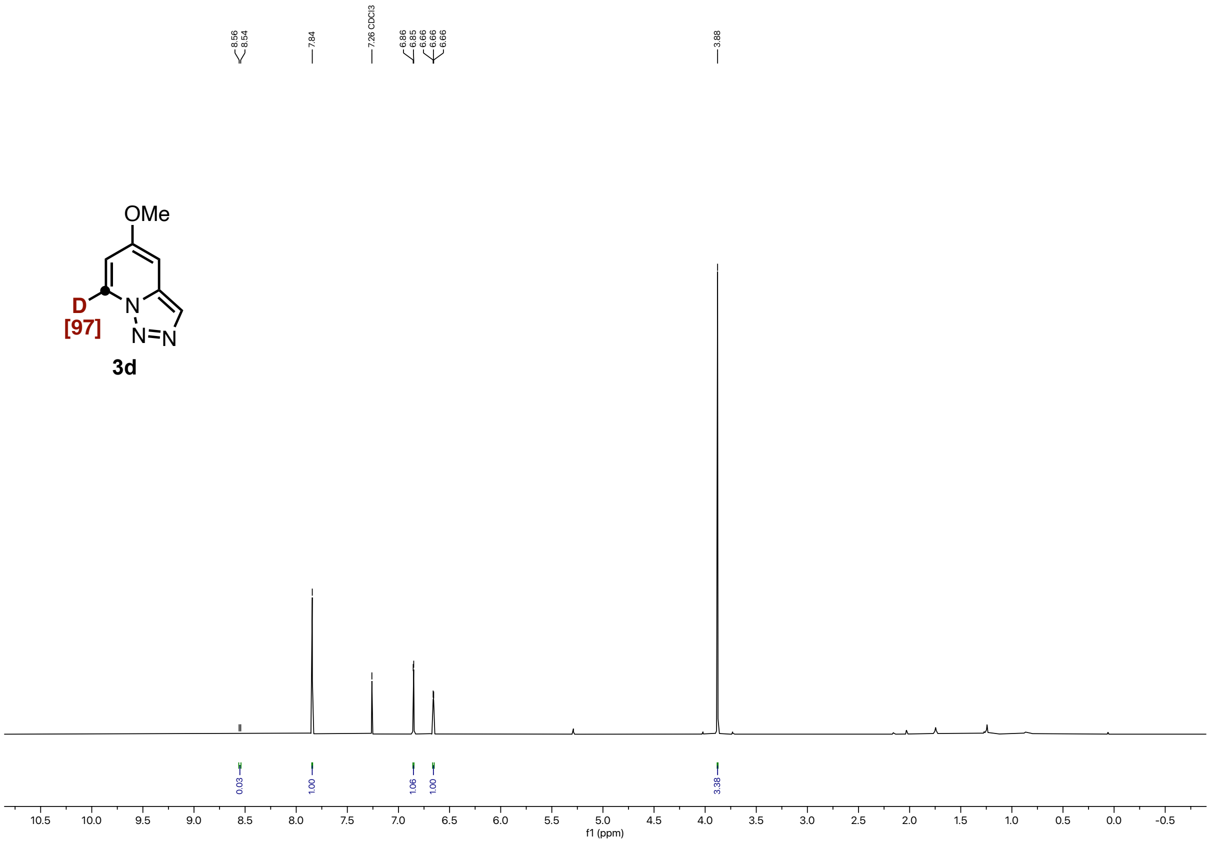

^{1}H spectrum (500 MHz, CD_3Cl) of **3d**

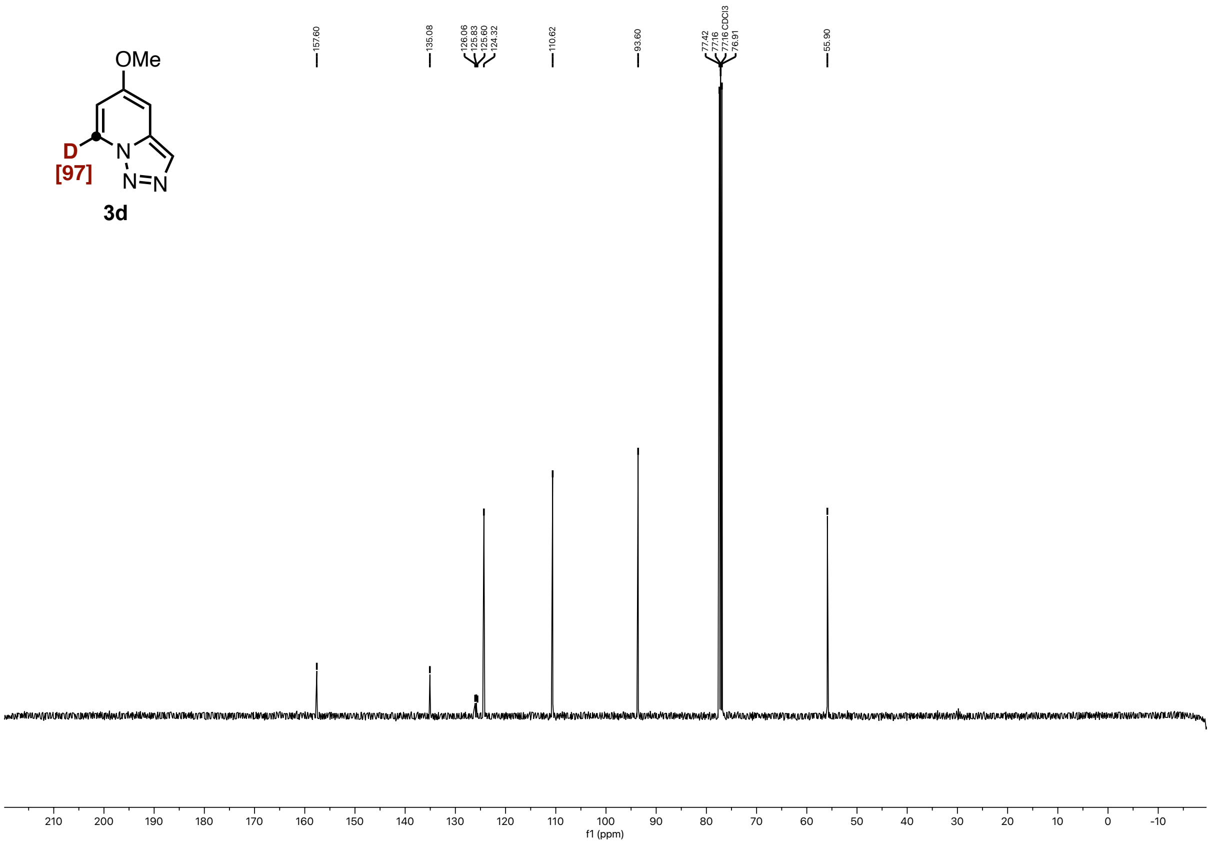

^{13}C spectrum (126 MHz, CD_3Cl) of **3d**

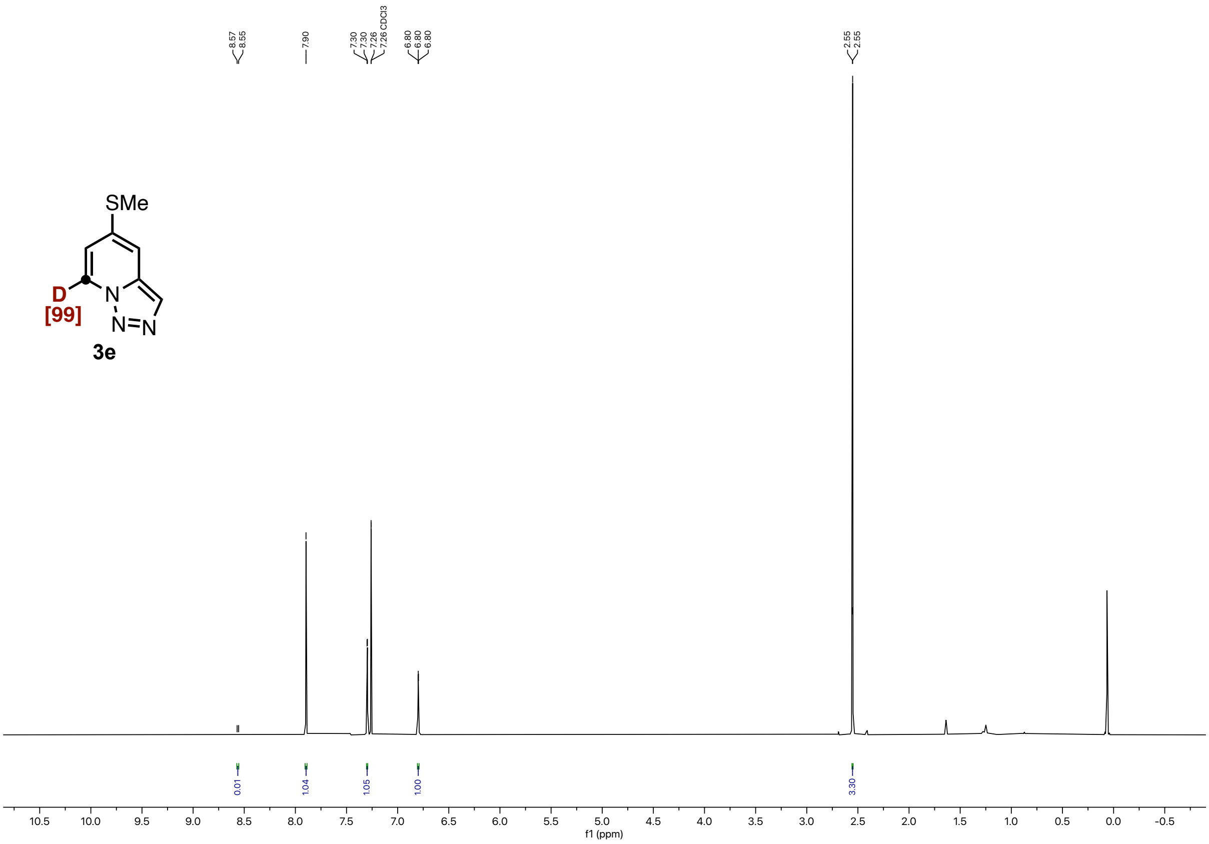

^{1}H spectrum (500 MHz, CD3Cl) of **3e**

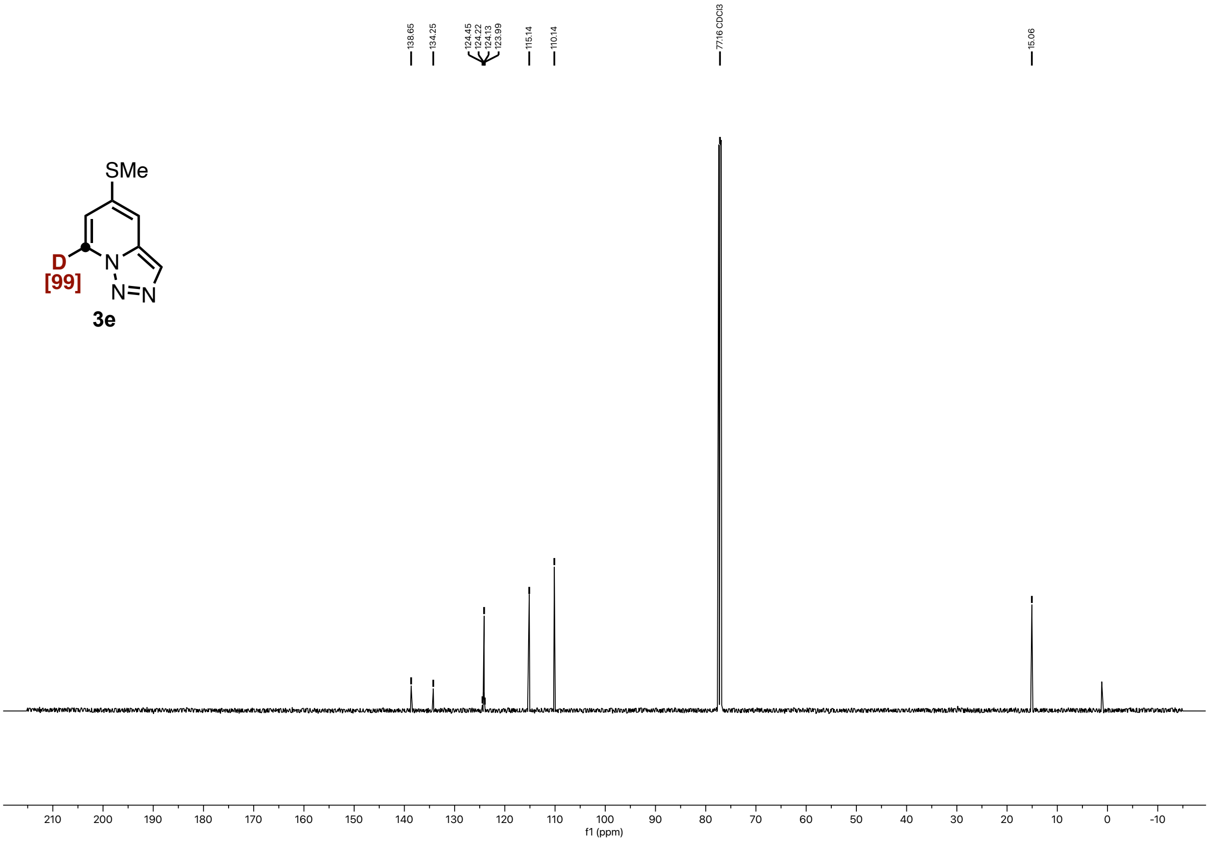

^{13}C spectrum (126 MHz, CD3Cl) of **3e**

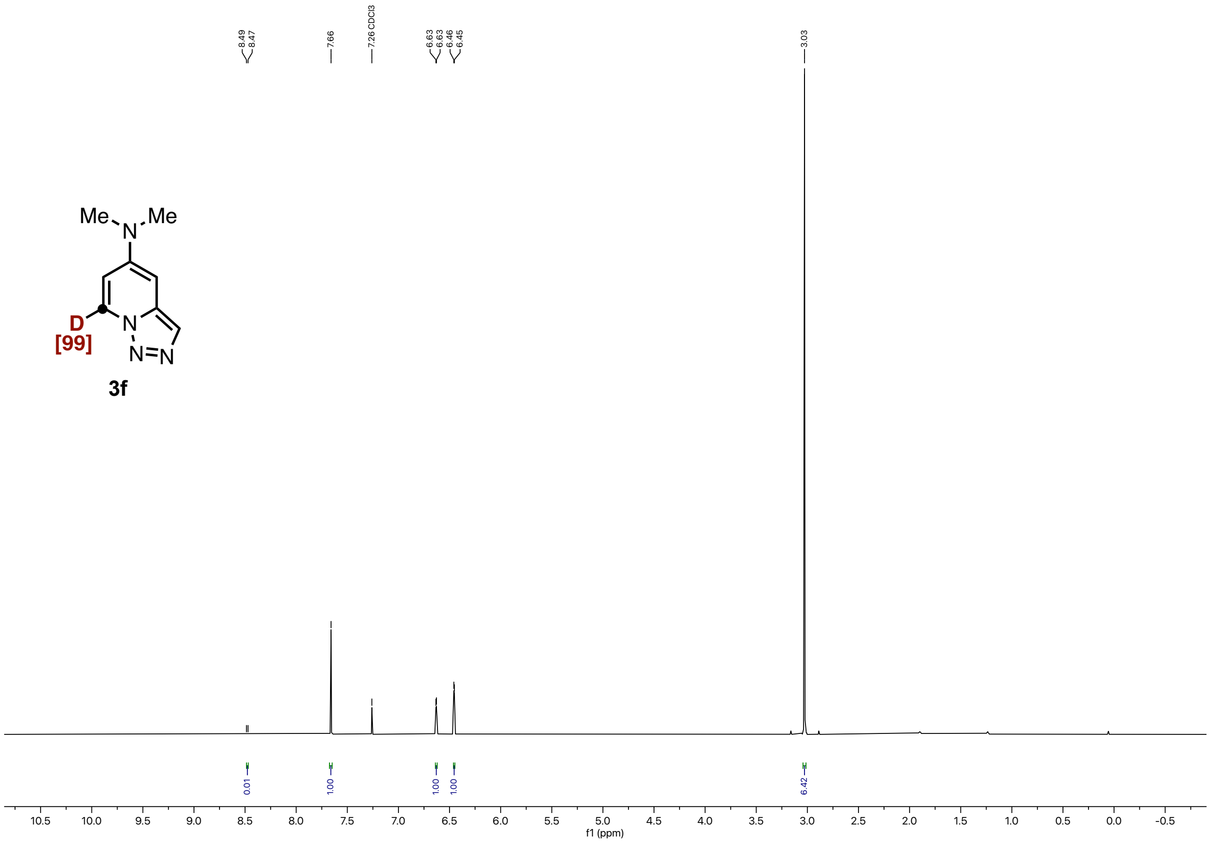

^{1}H spectrum (500 MHz, CD3Cl) of **3f**

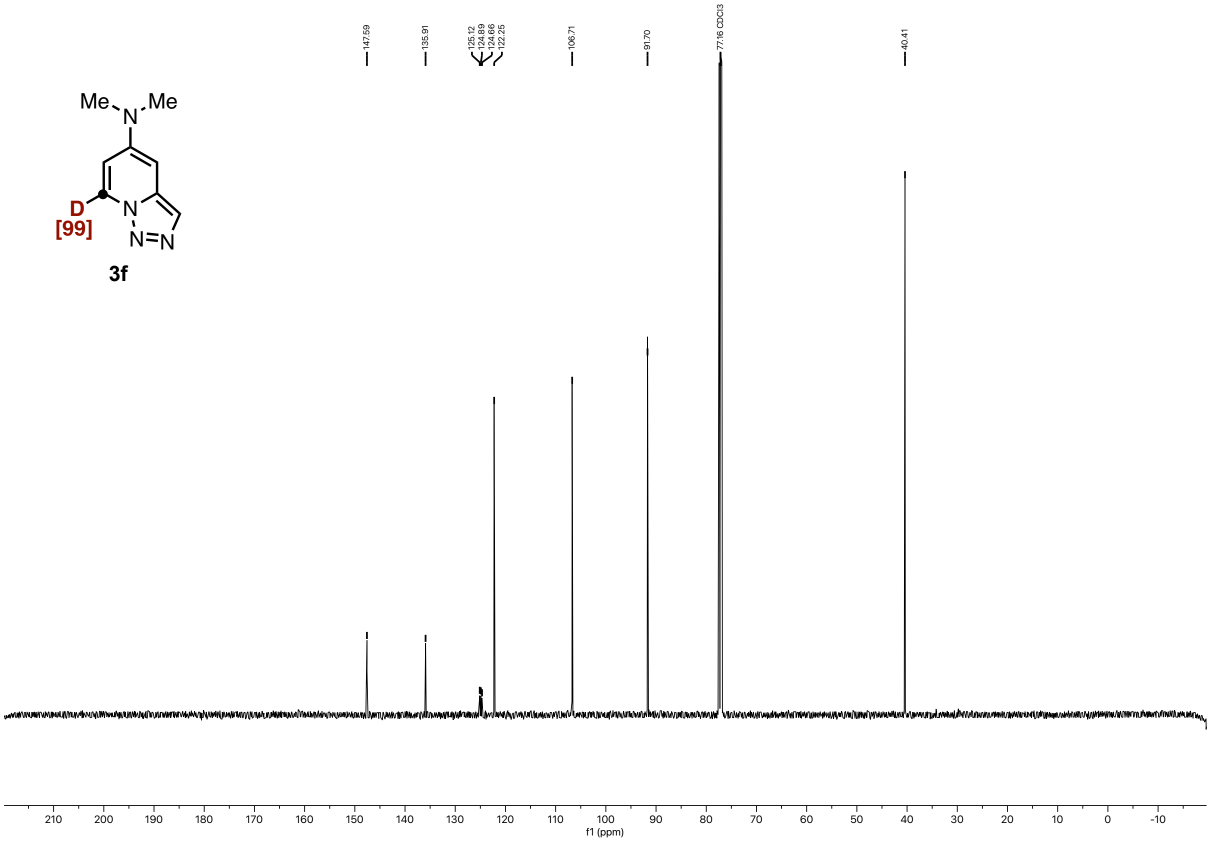

^{13}C spectrum (126 MHz, CD3Cl) of **3f**

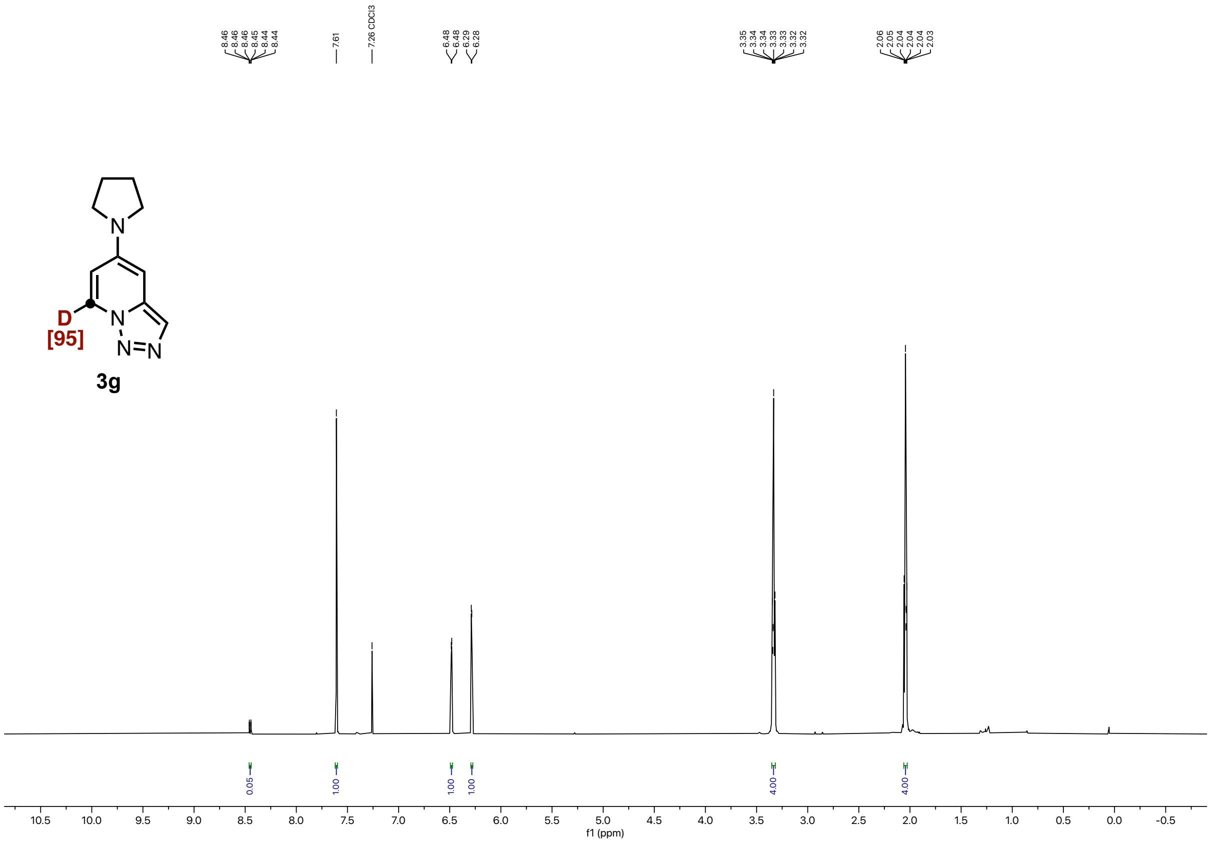

^{1}H spectrum (500 MHz, CD_3Cl) of **3g**

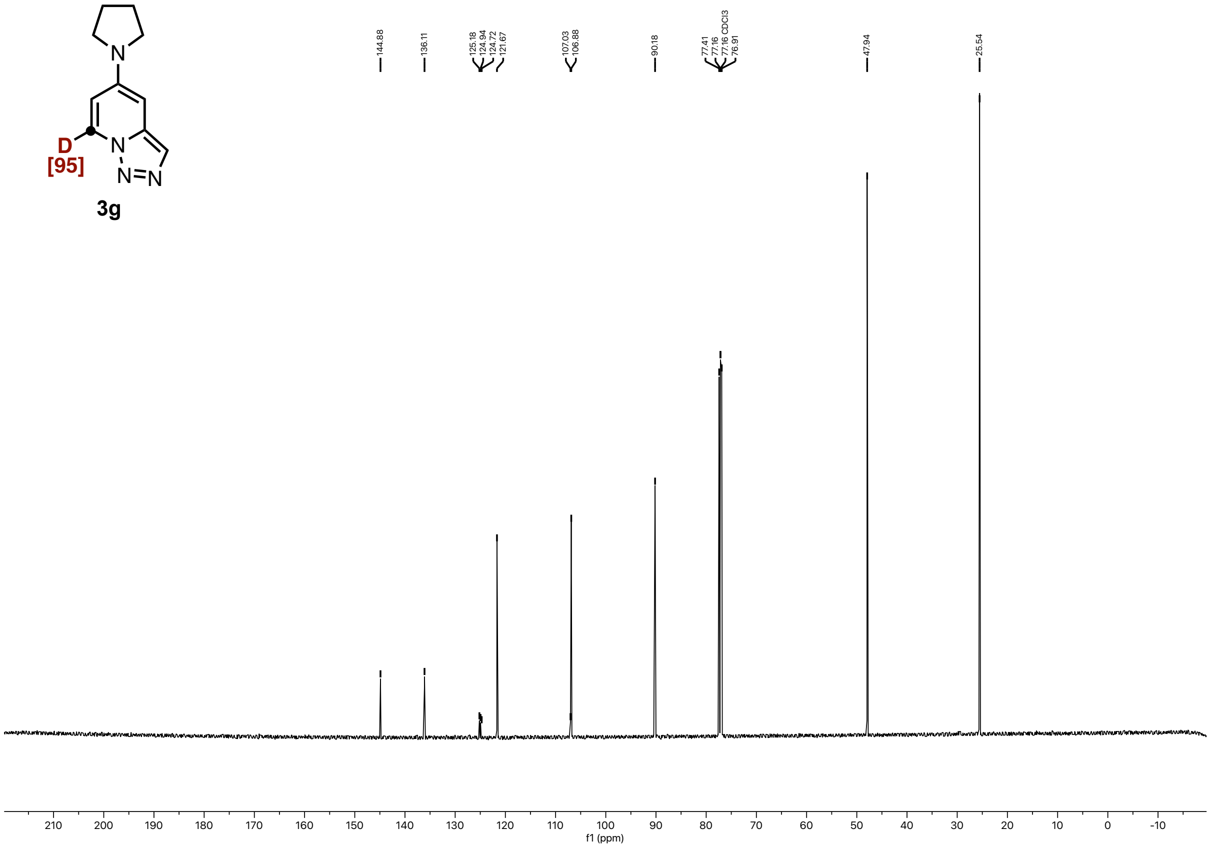

^{13}C spectrum (126 MHz, CD_3Cl) of **3g**

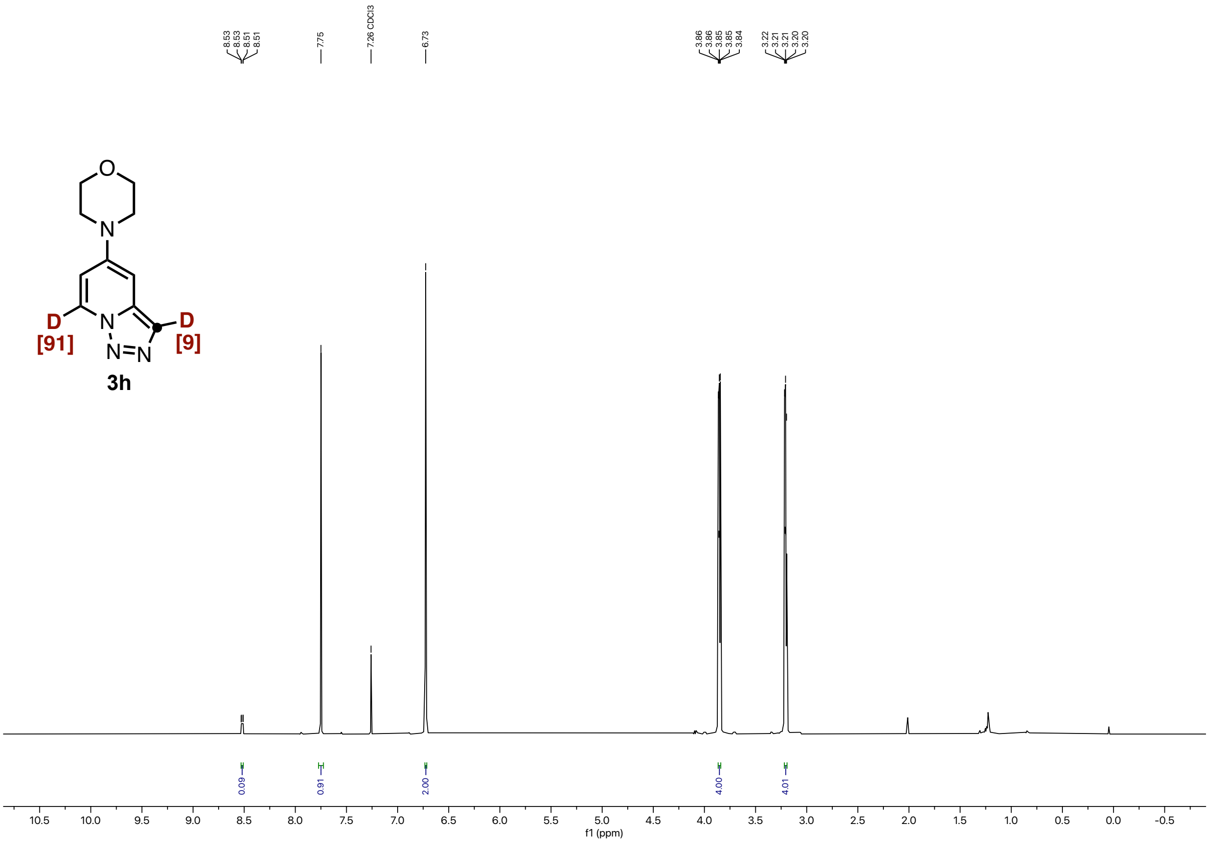

^{1}H spectrum (500 MHz, CD_3Cl) of **3h**

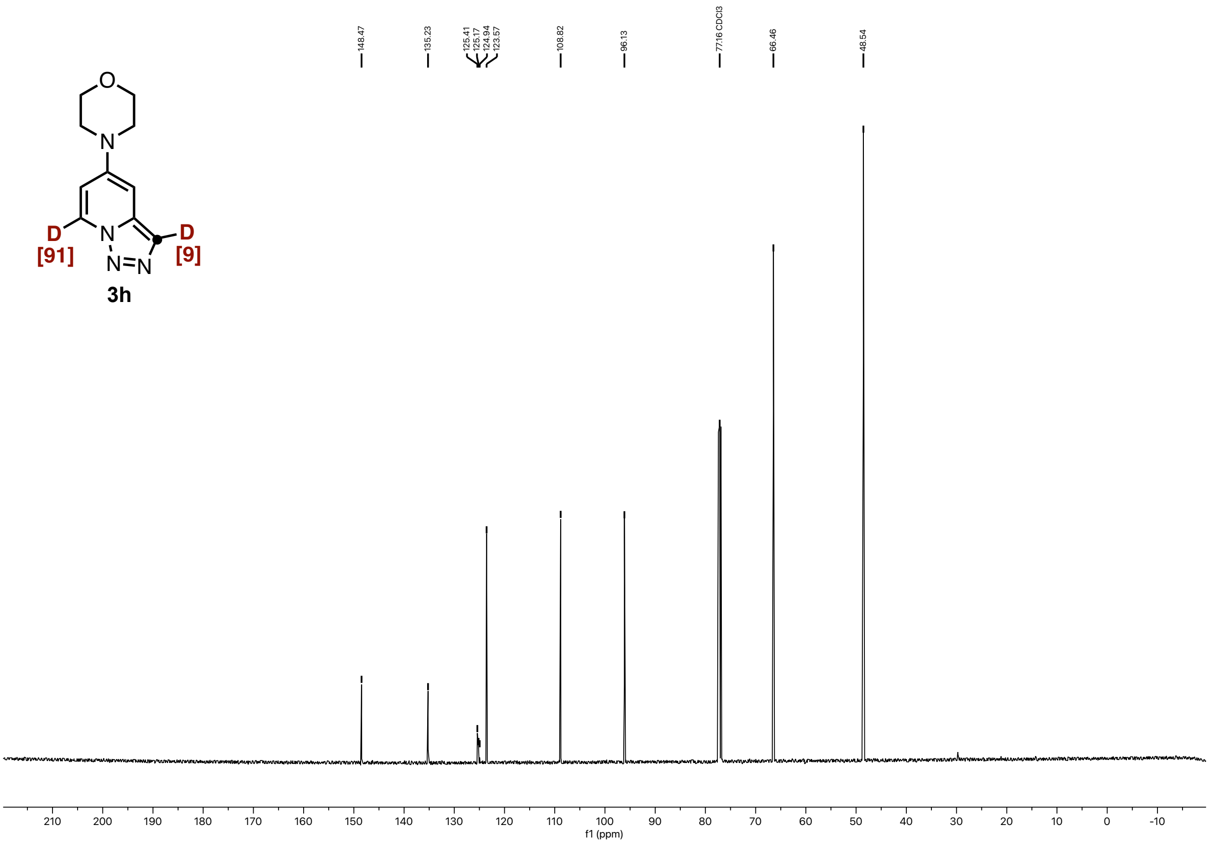

^{13}C spectrum (126 MHz, CD_3Cl) of **3h**

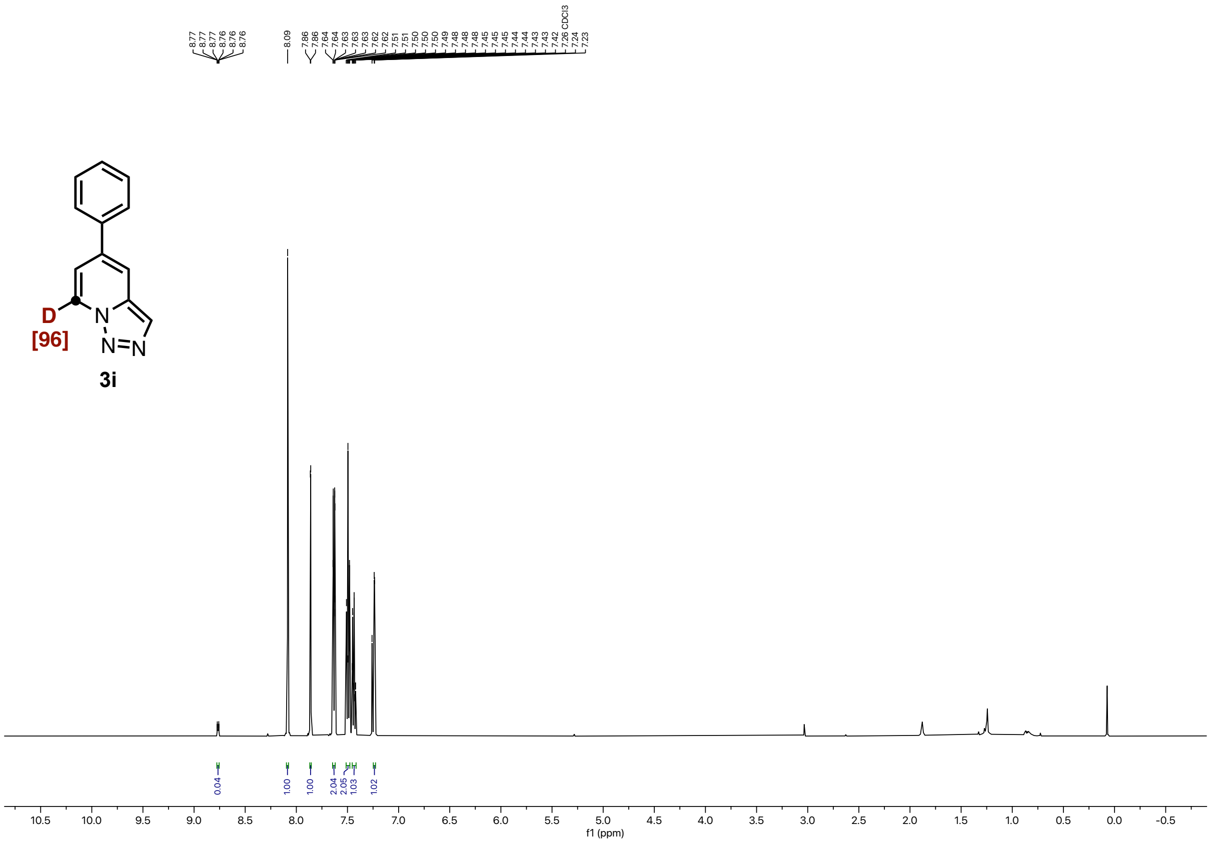

^{1}H spectrum (500 MHz, CD3Cl) of **3i**

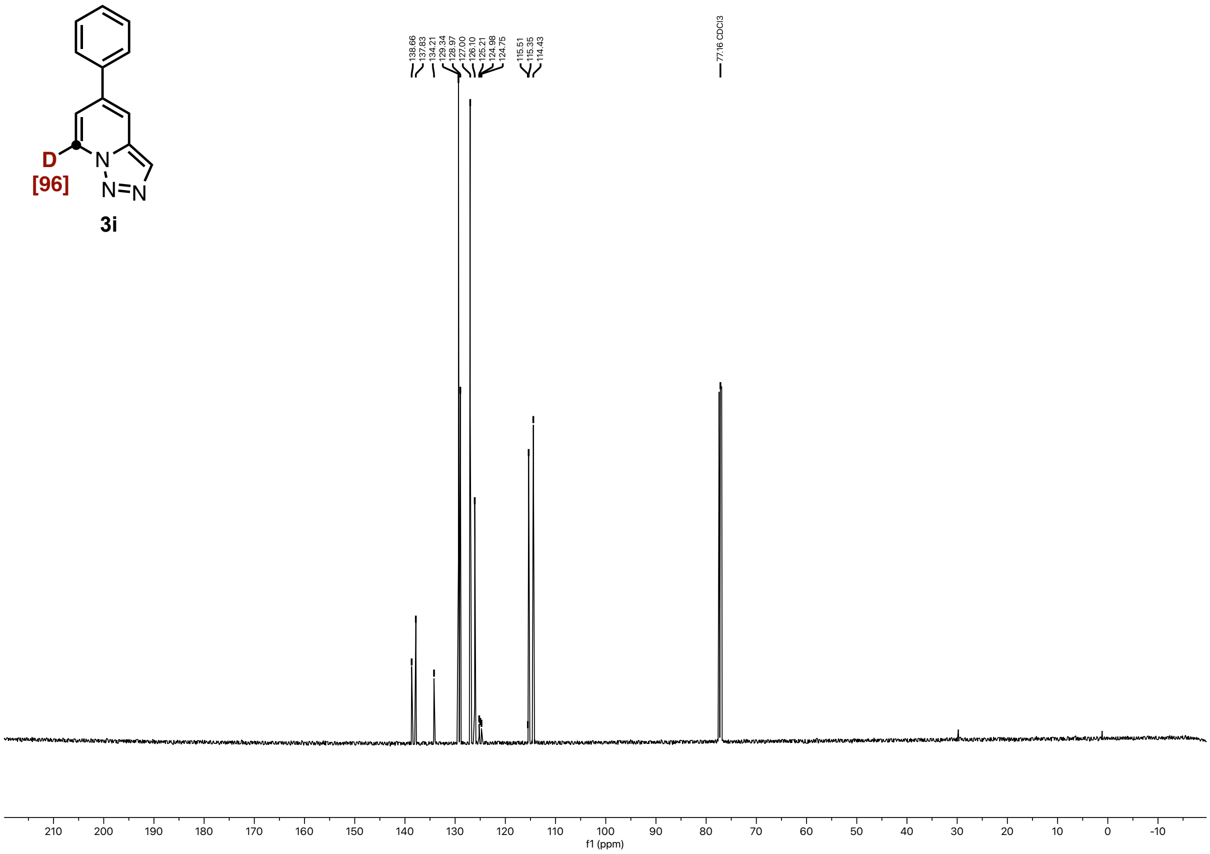

^{13}C spectrum (126 MHz, CD3Cl) of **3i**

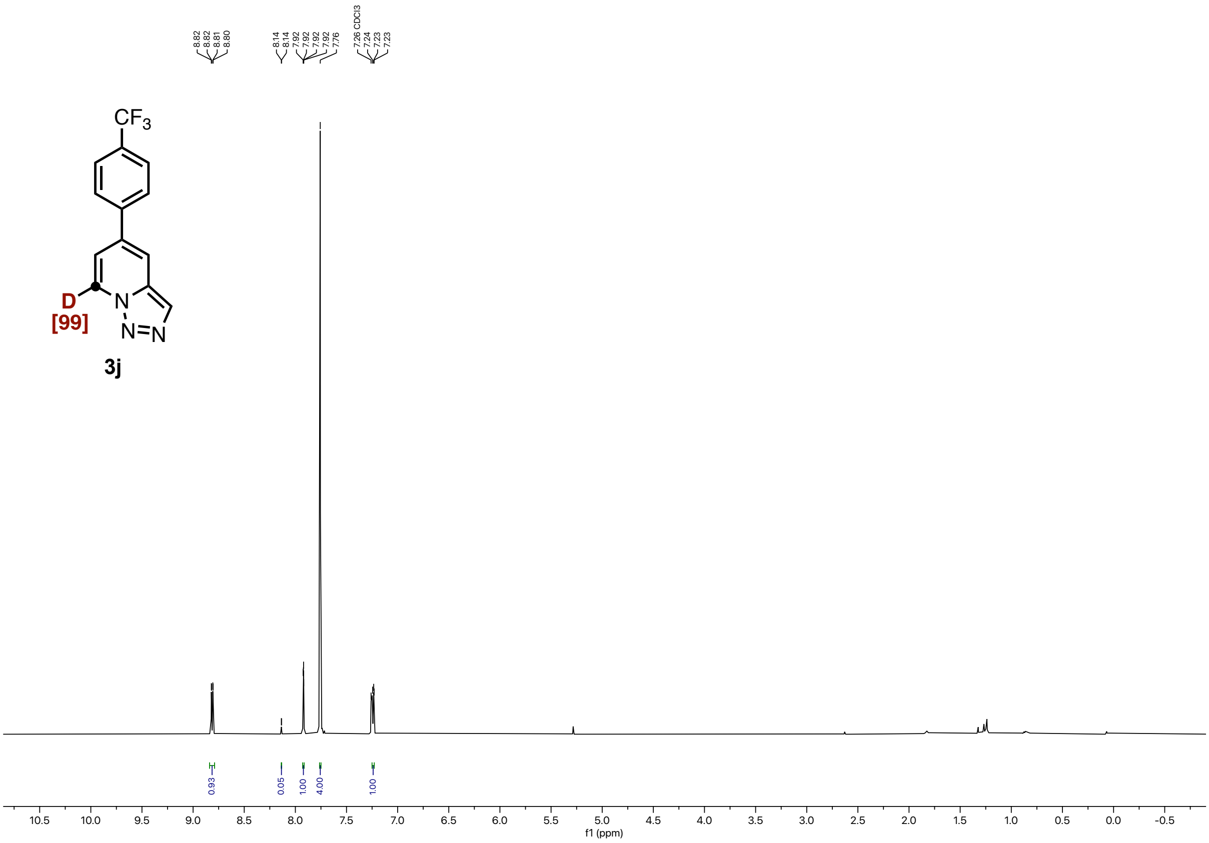

^{1}H spectrum (500 MHz, CD3Cl) of **3j**

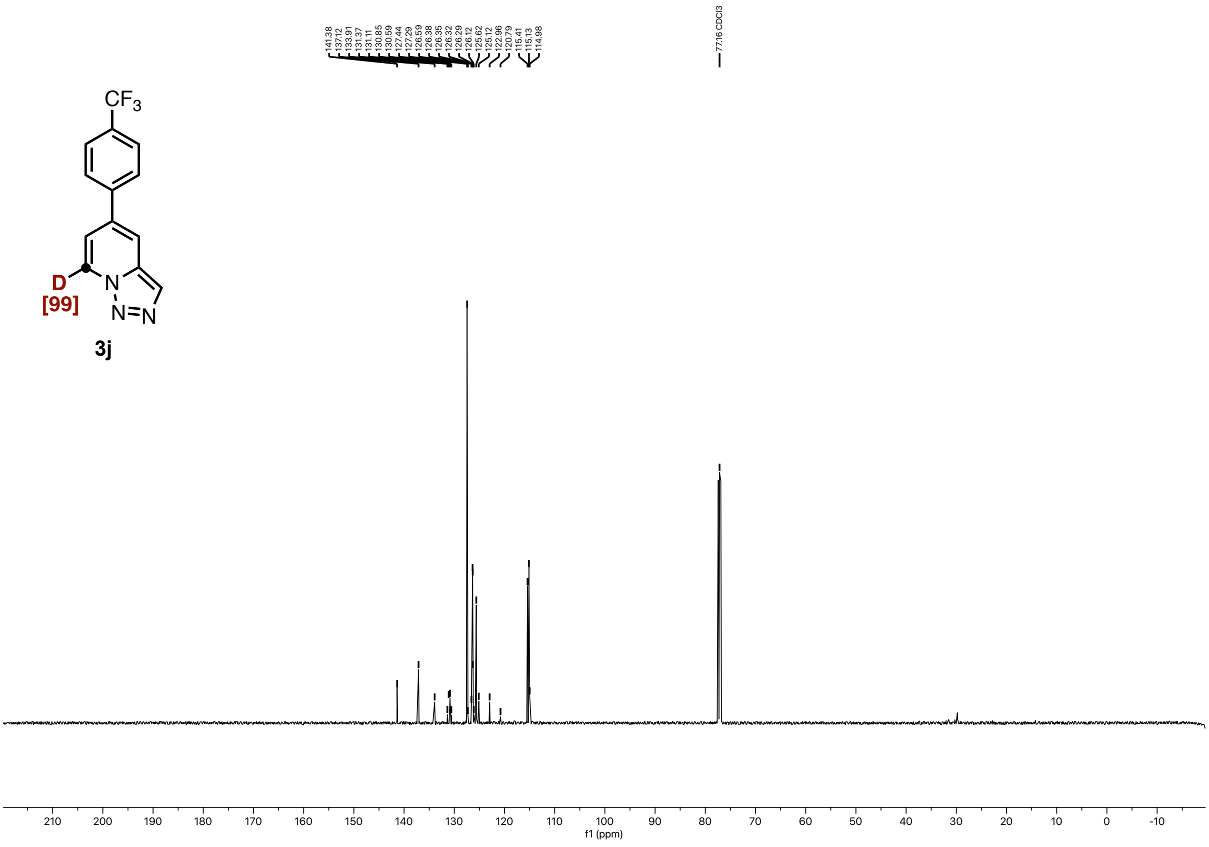

^{13}C spectrum (126 MHz, CD3Cl) of **3j**

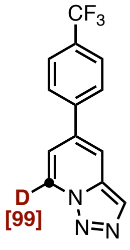

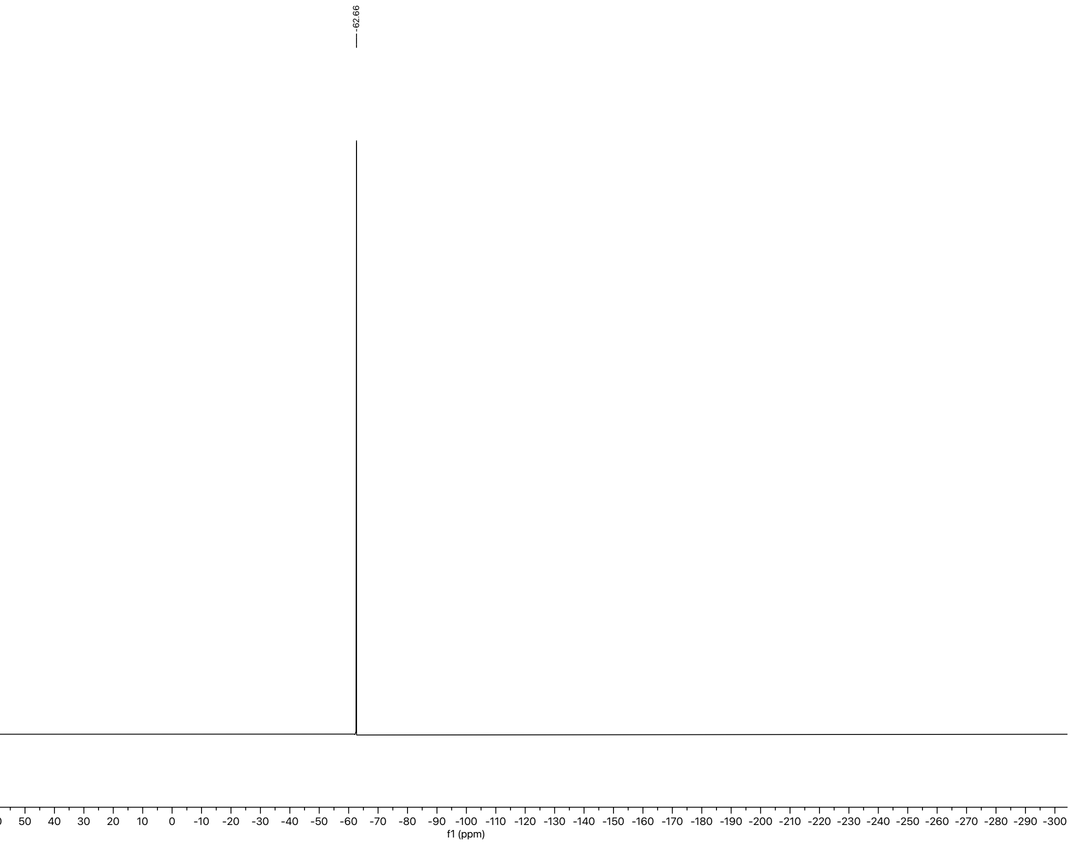

^{19}F spectrum (471 MHz, $CDCl_3$) of **3j**

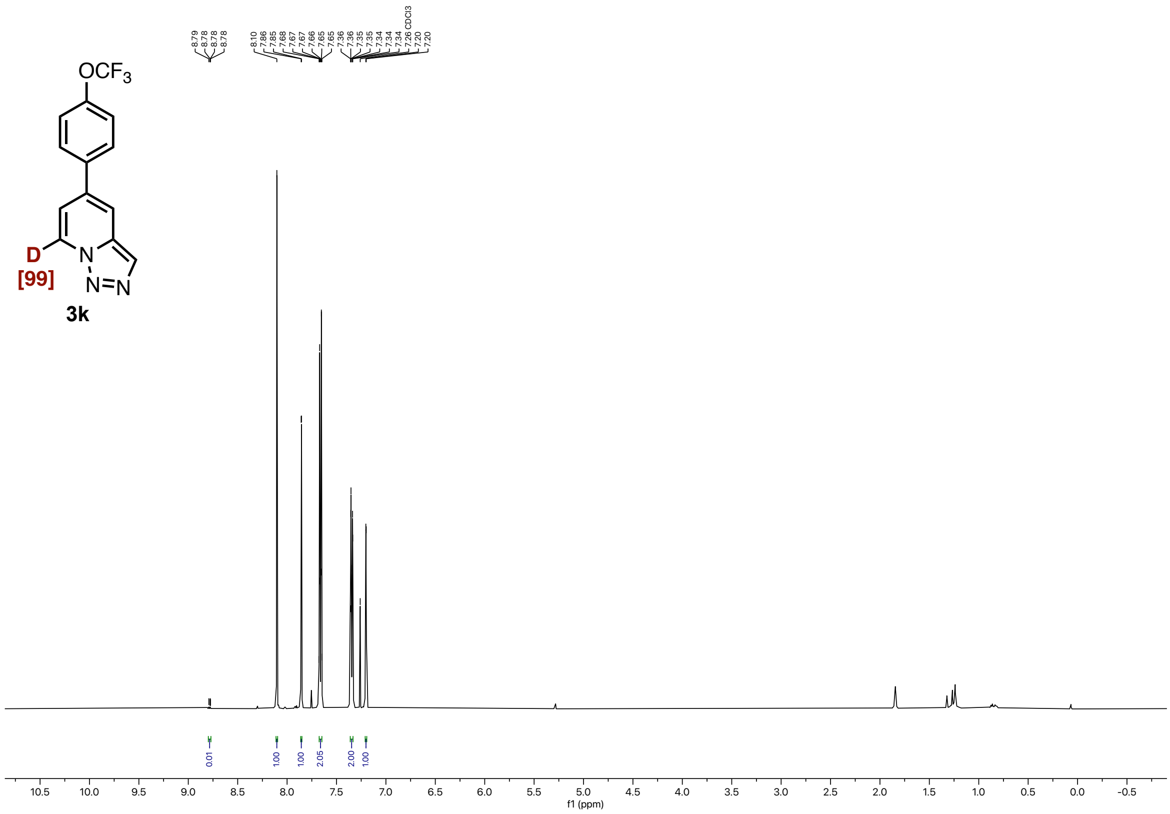

^{1}H spectrum (500 MHz, CD3Cl) of **3k**

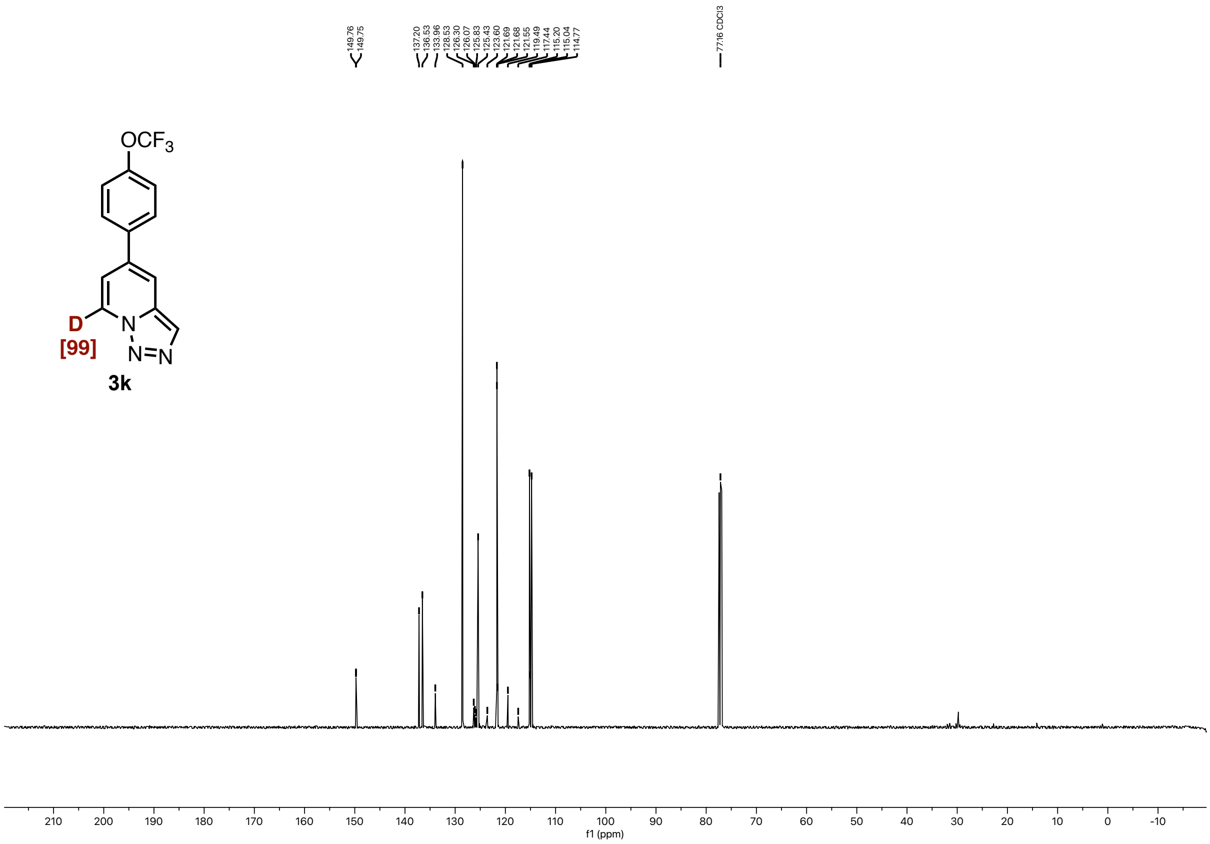

^{13}C spectrum (500 MHz, CD3Cl) of **3k**

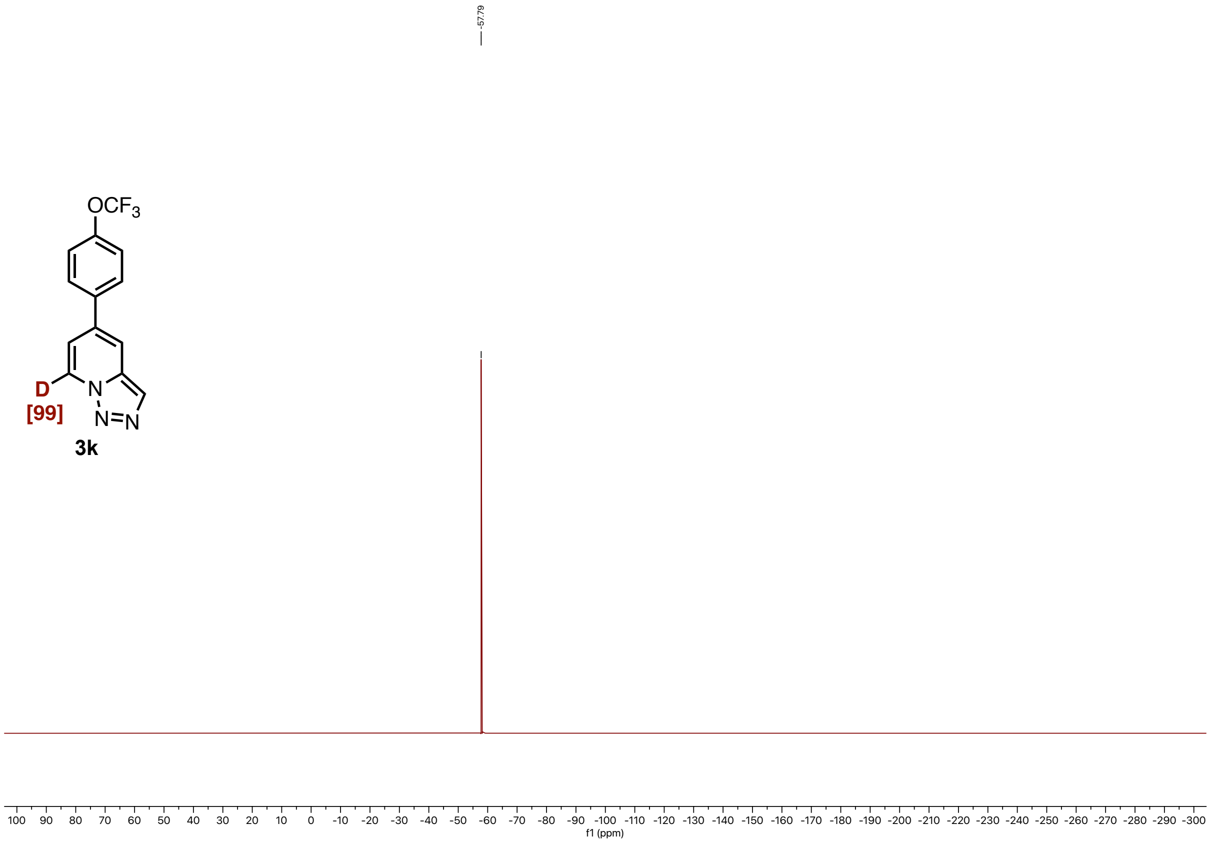

^{19}F spectrum (471 MHz, $CDCl_3$) of **3k**

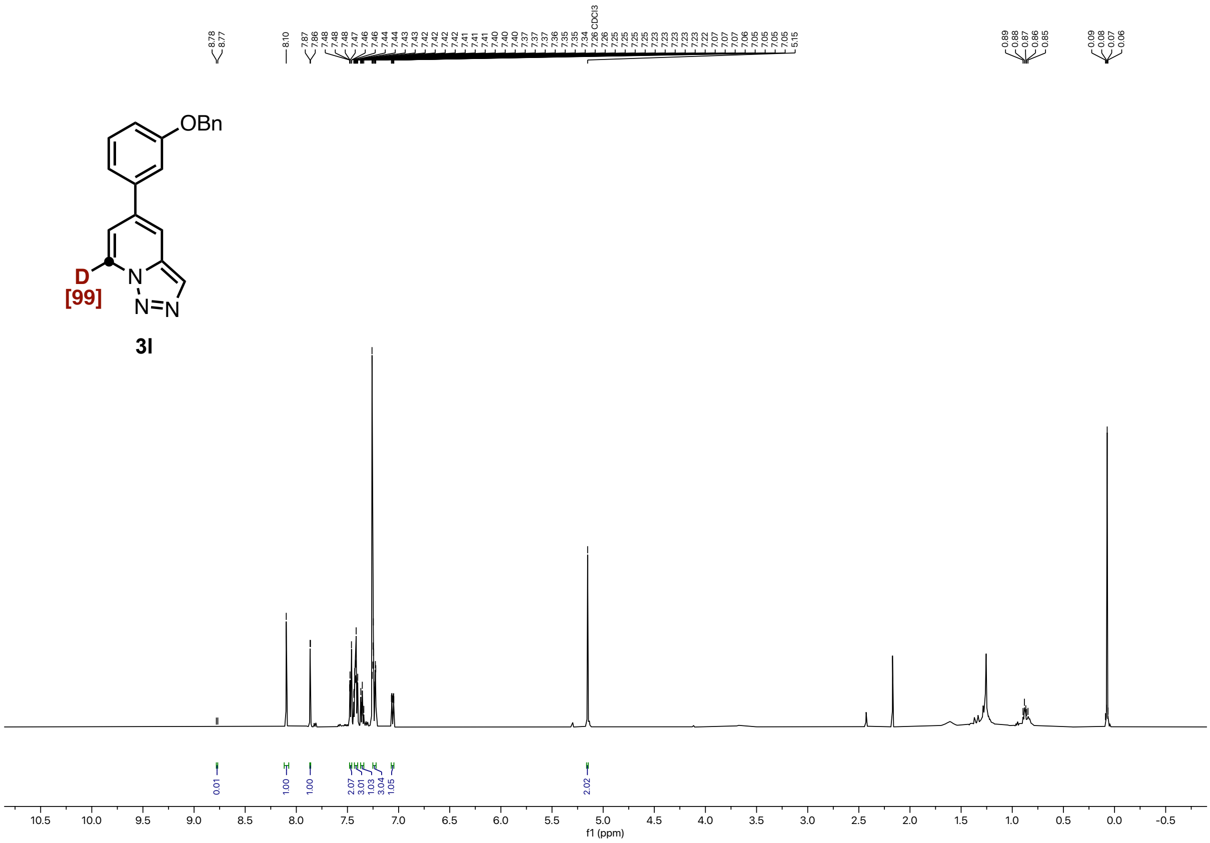

^1H spectrum (500 MHz, CD_3Cl) of **3l**

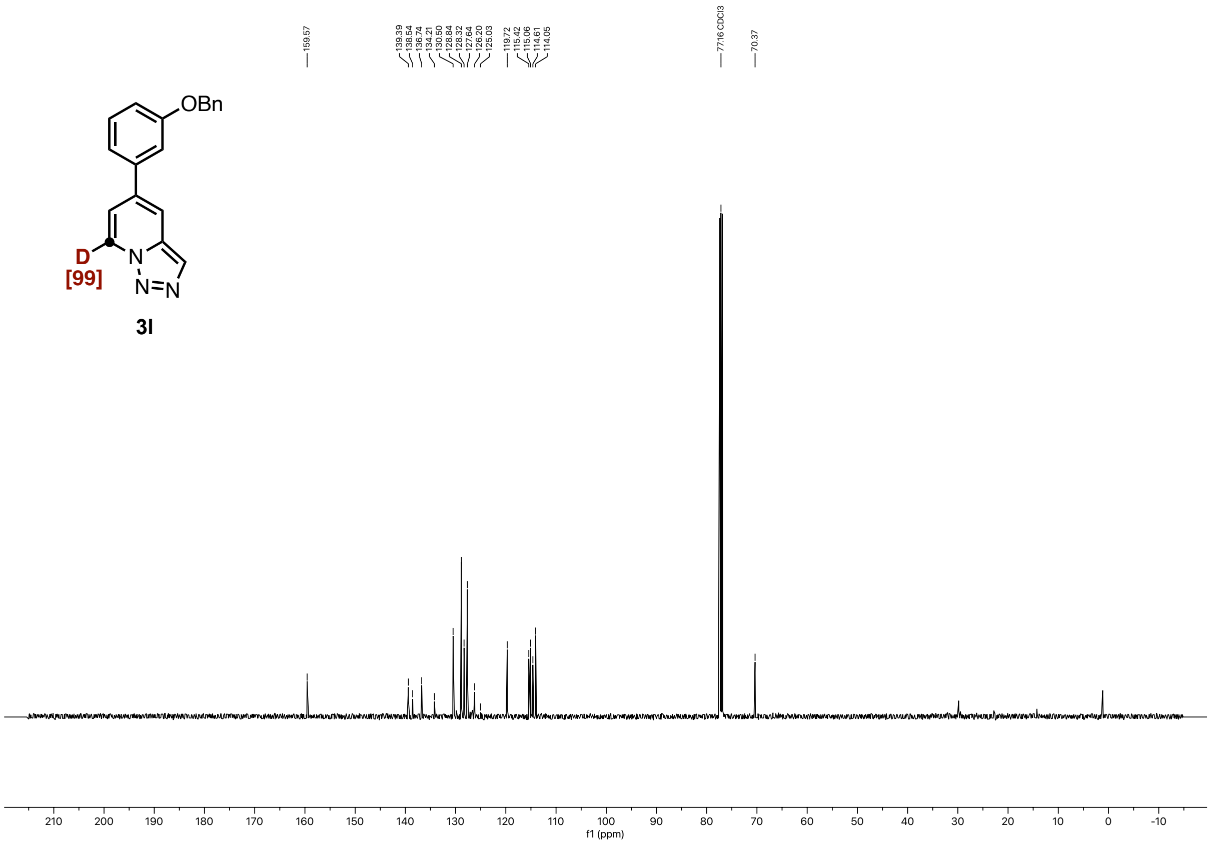

^{13}C spectrum (500 MHz, CD_3Cl) of **3l**

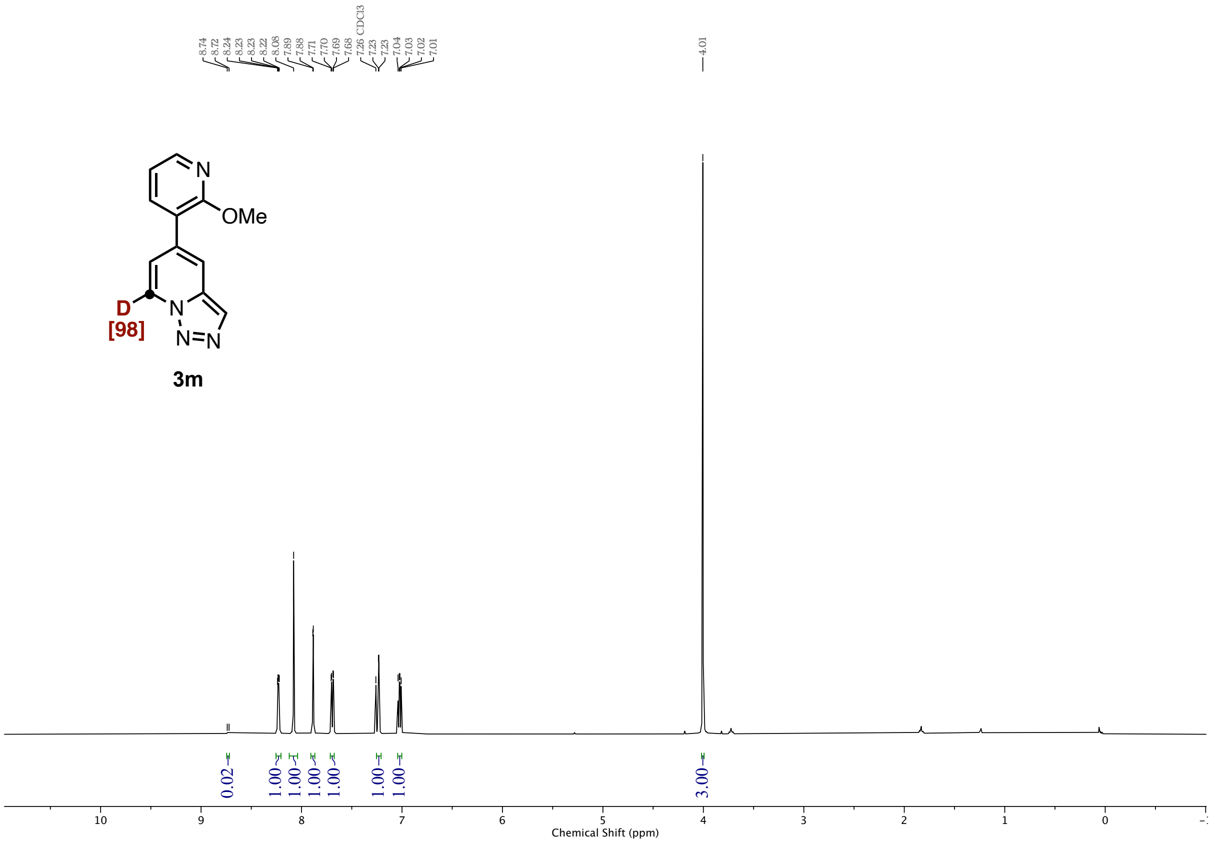

^{1}H spectrum (500 MHz, CD_3Cl) of **3m**

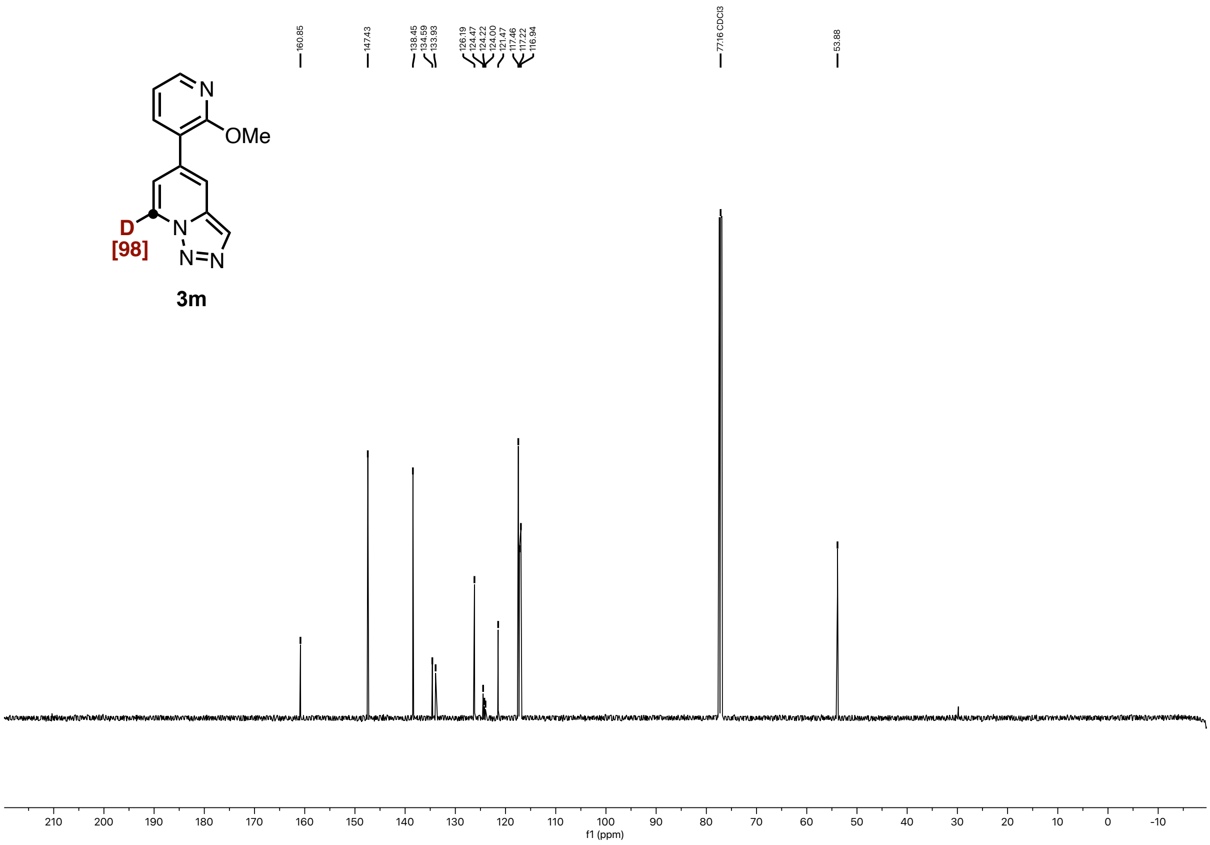

^{13}C spectrum (126 MHz, CD_3Cl) of **3m**

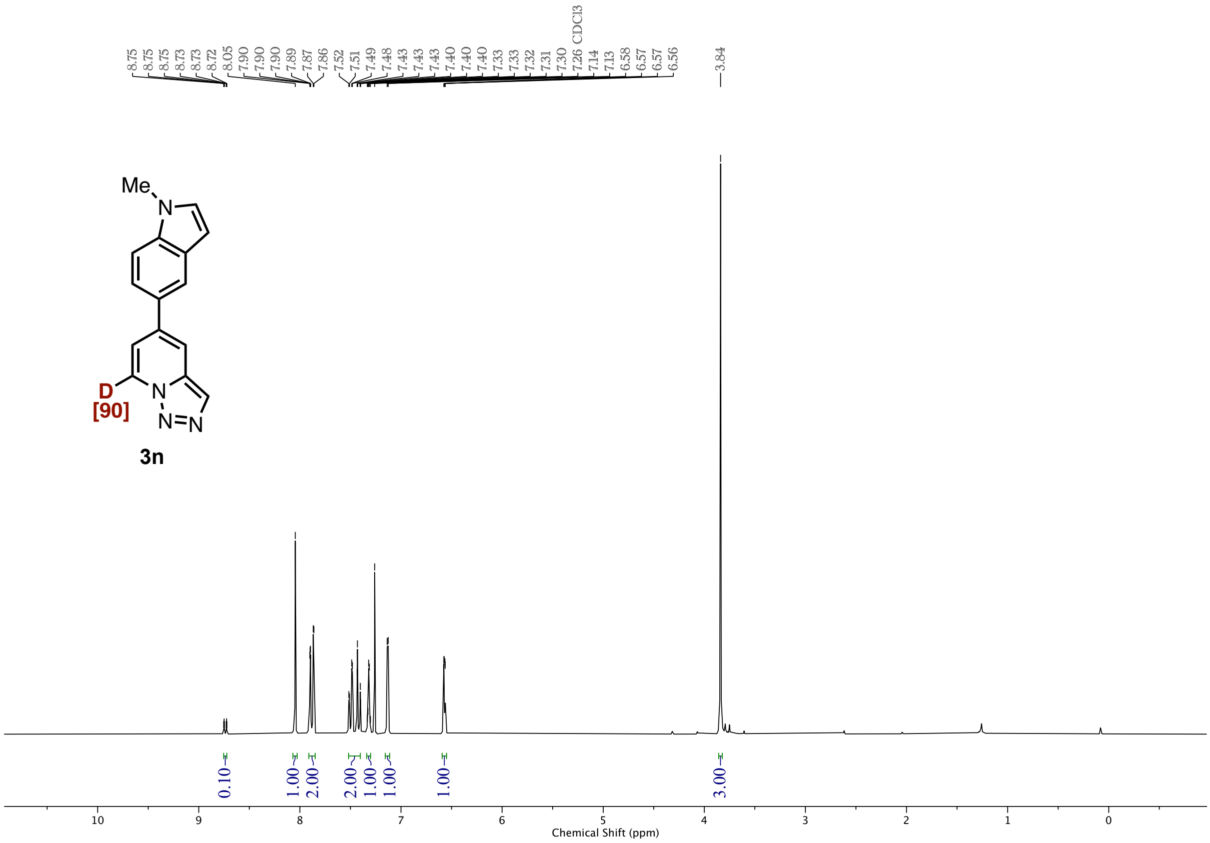

^{1}H spectrum (500 MHz, CD3Cl) of **3n**

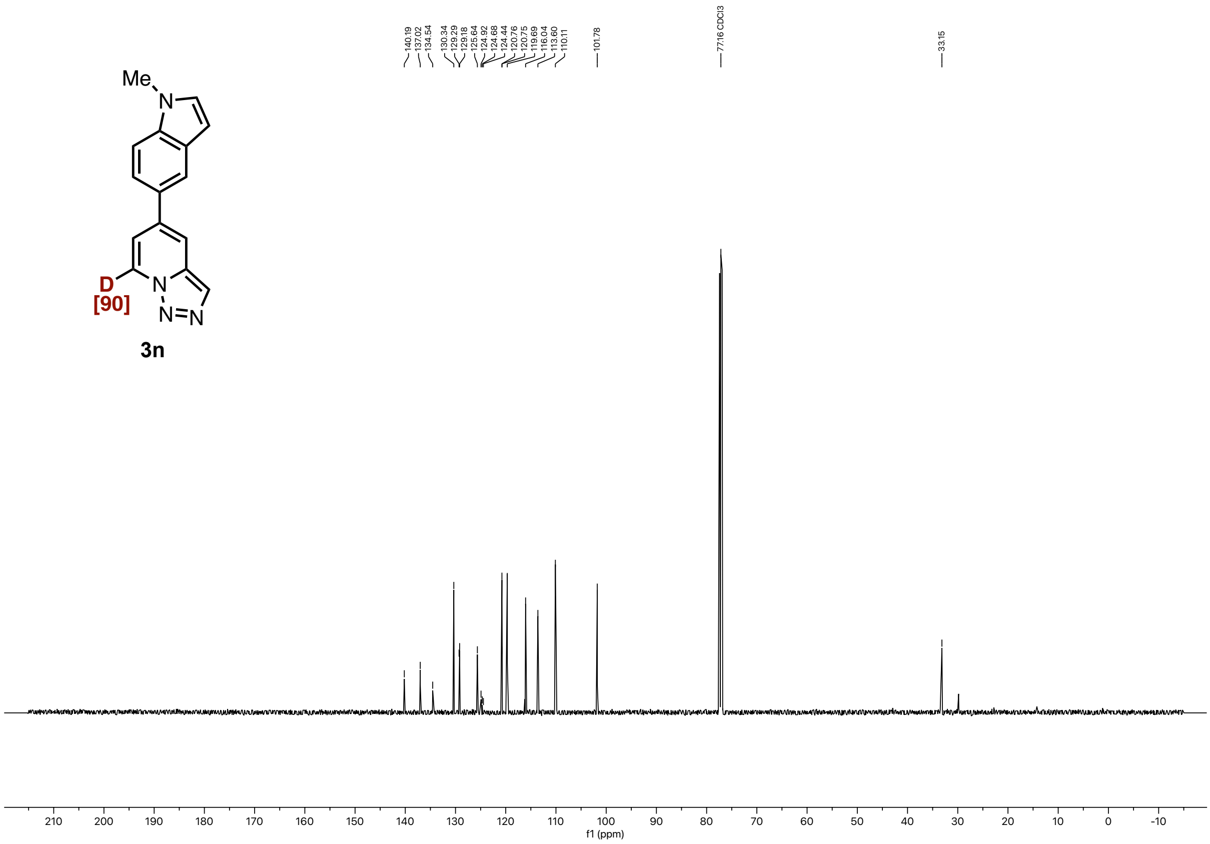

^{13}C spectrum (126 MHz, CD3Cl) of **3n**

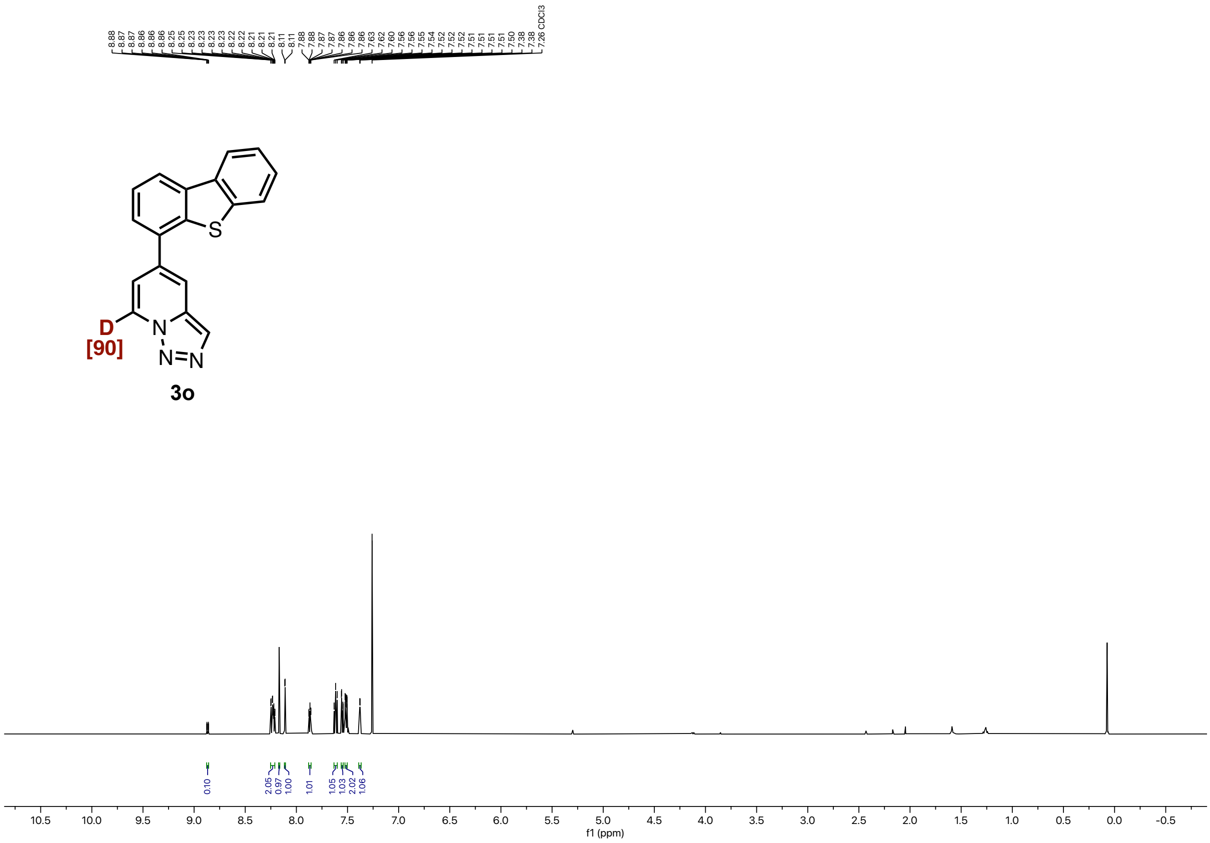

^{1}H spectrum (500 MHz, CD_3Cl) of **3o**

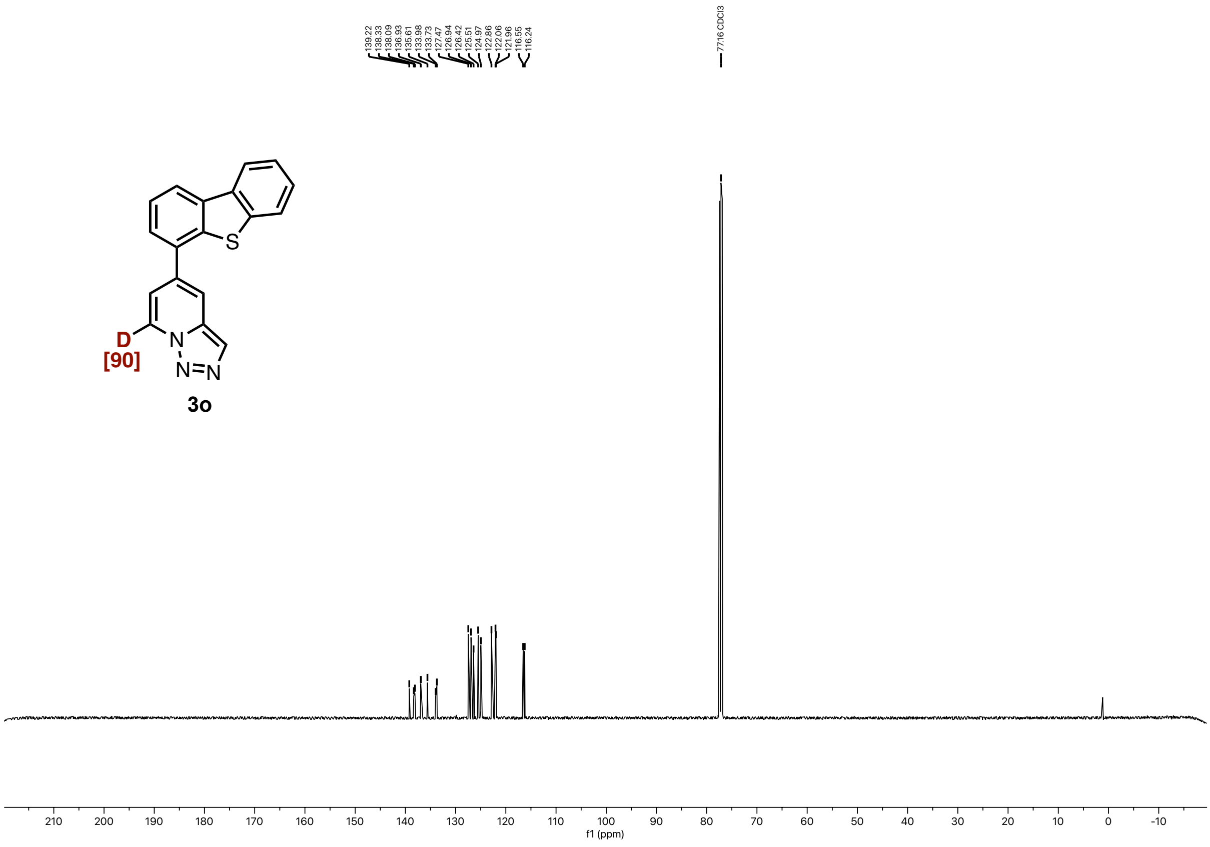

^{13}C spectrum (126 MHz, CD_3Cl) of **3o**

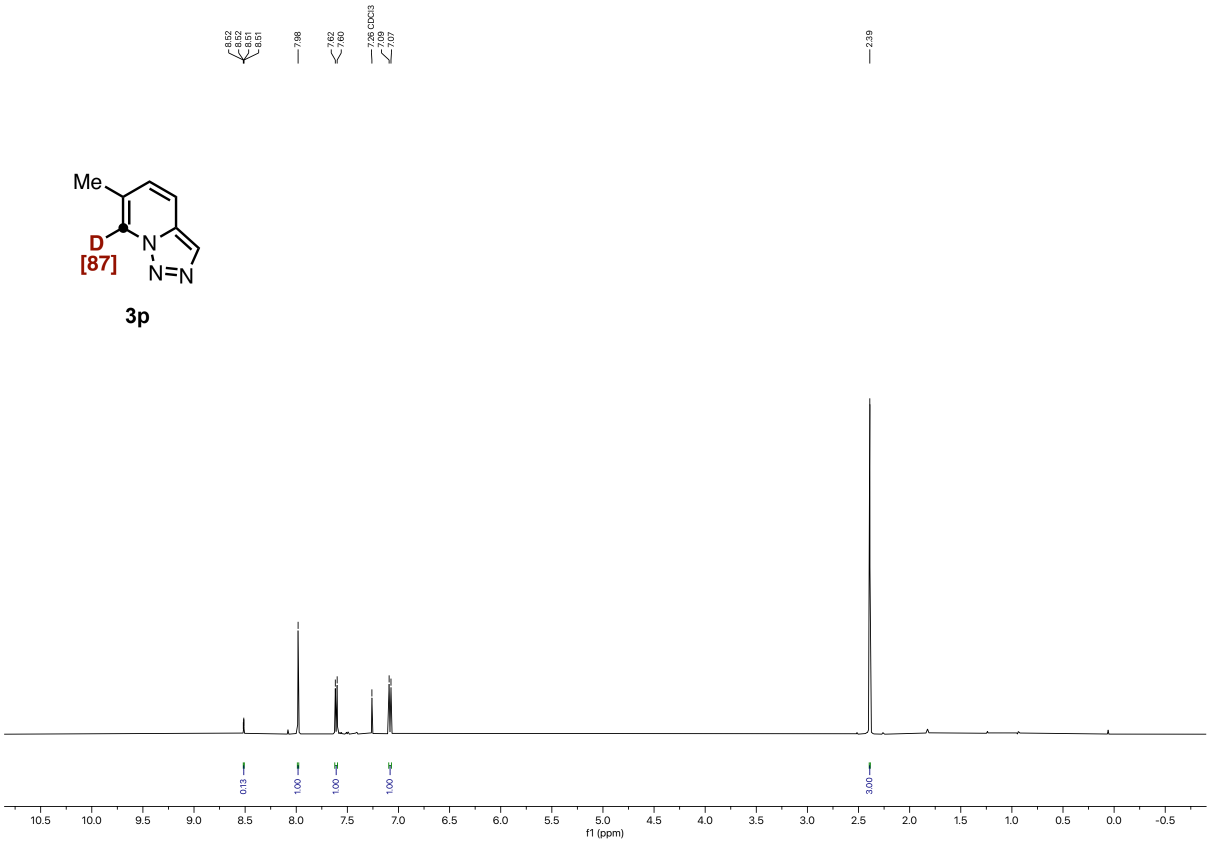

^{1}H spectrum (500 MHz, CD_3Cl) of **3p**

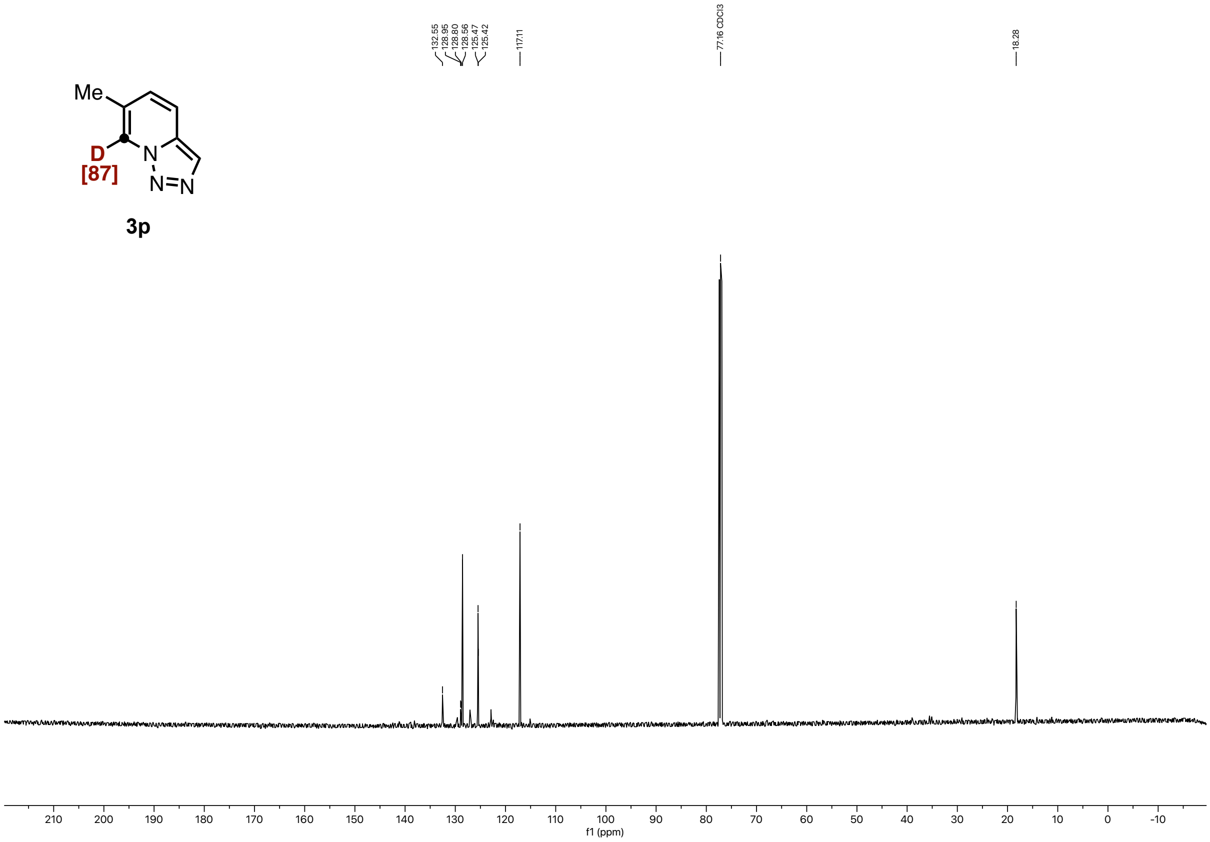

^{13}C spectrum (126 MHz, CD_3Cl) of **3p**

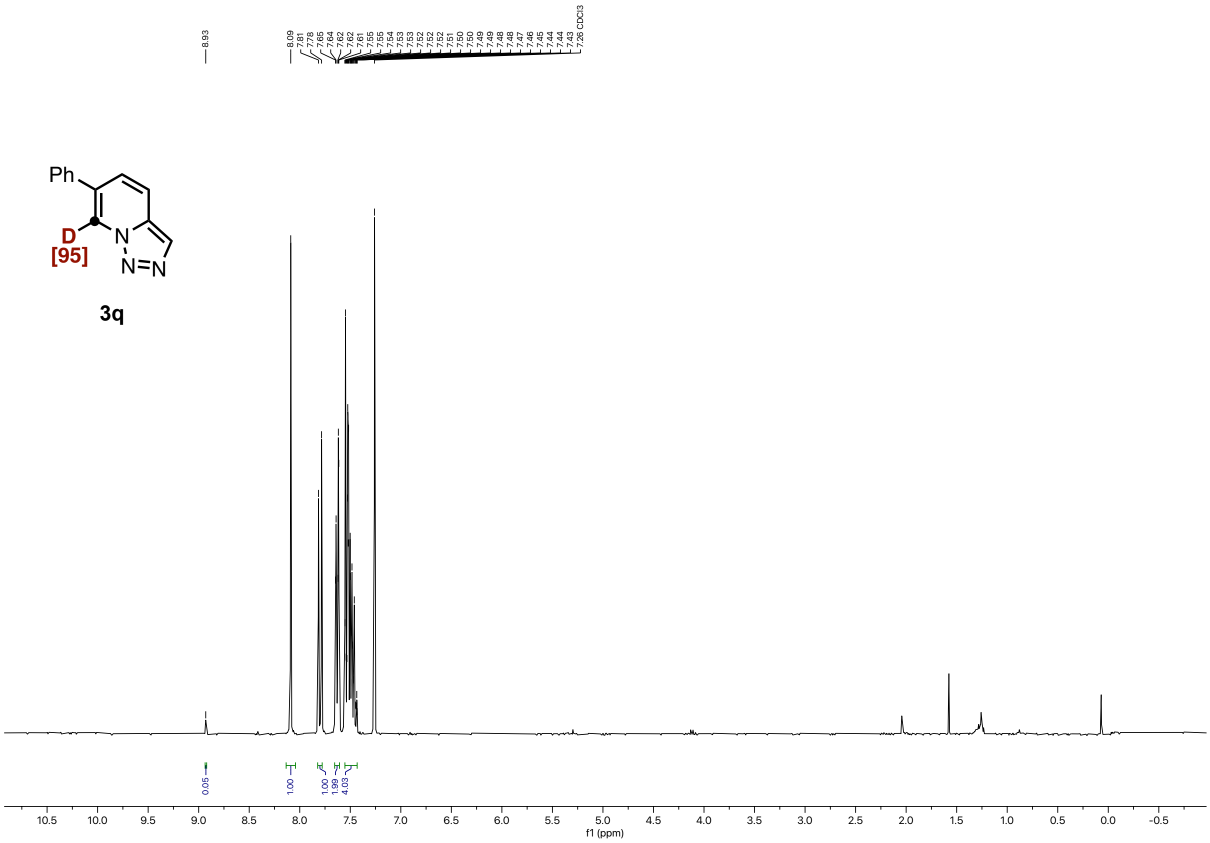

^{1}H spectrum (500 MHz, CD_3Cl) of **3q**

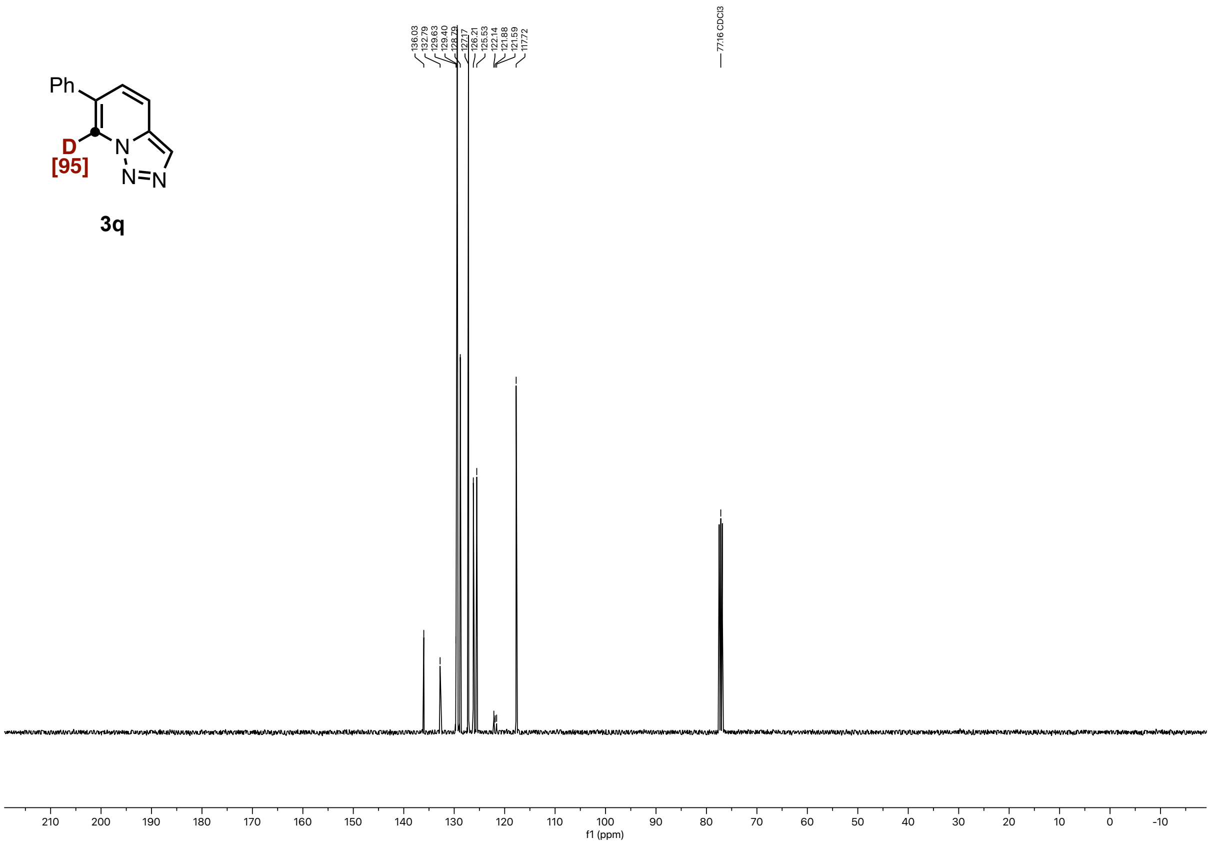

^{13}C spectrum (126 MHz, CD_3Cl) of **3q**

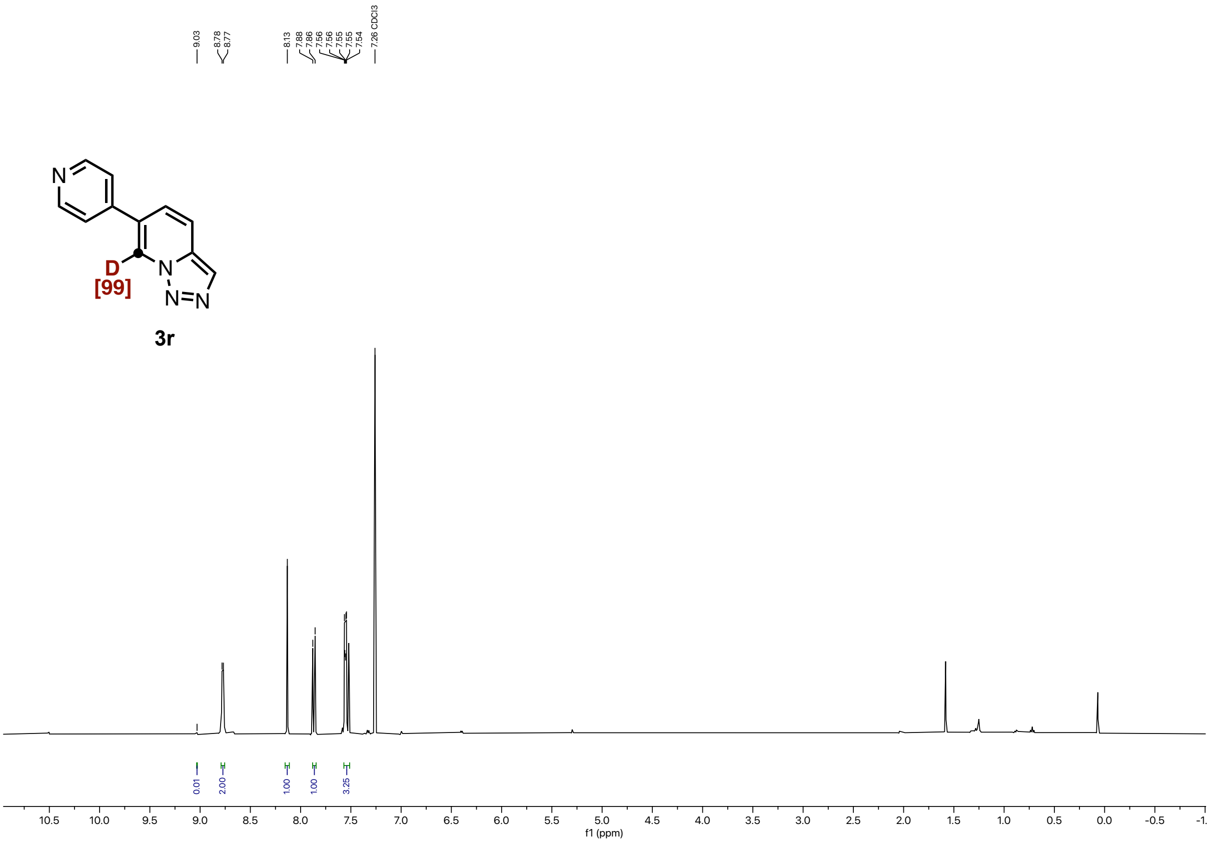

^{1}H spectrum (400 MHz, CD3Cl) of **3r**

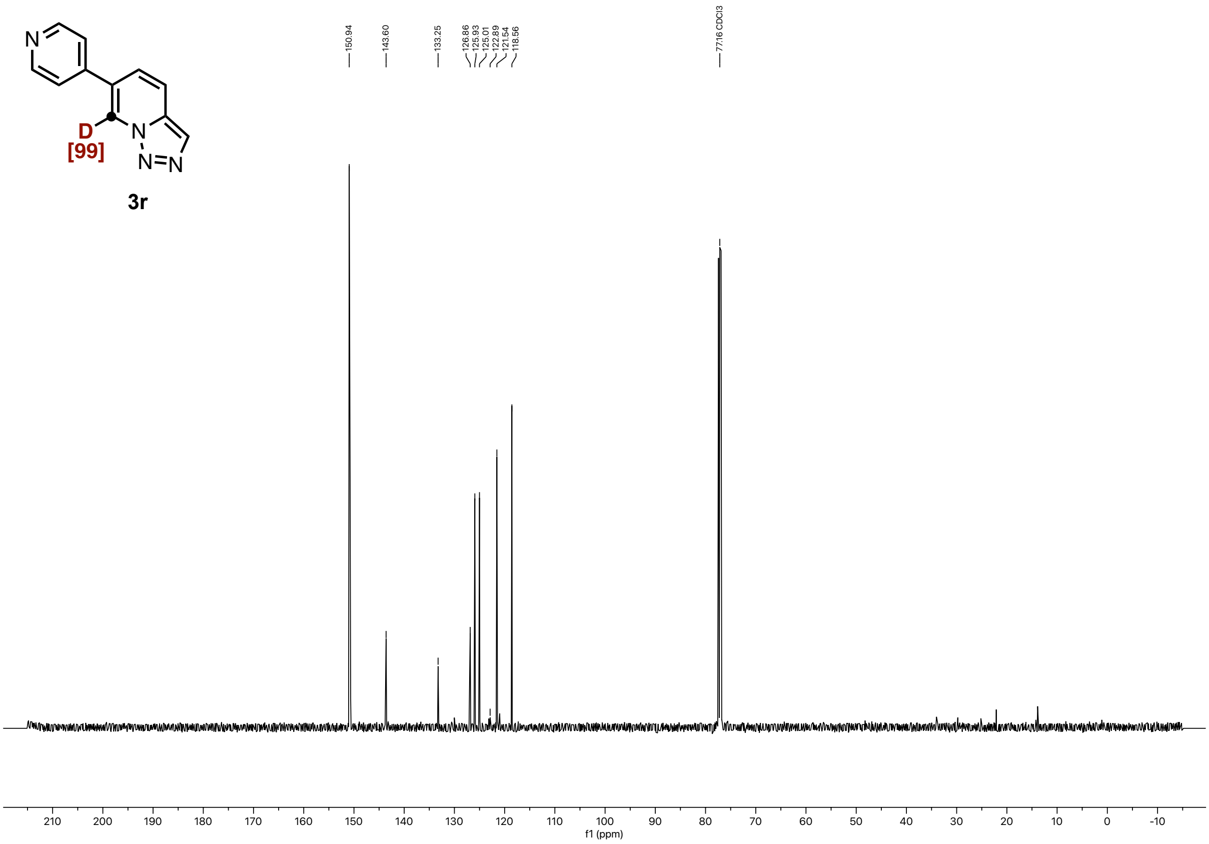

^{13}C spectrum (101 MHz, CD3Cl) of **3r**

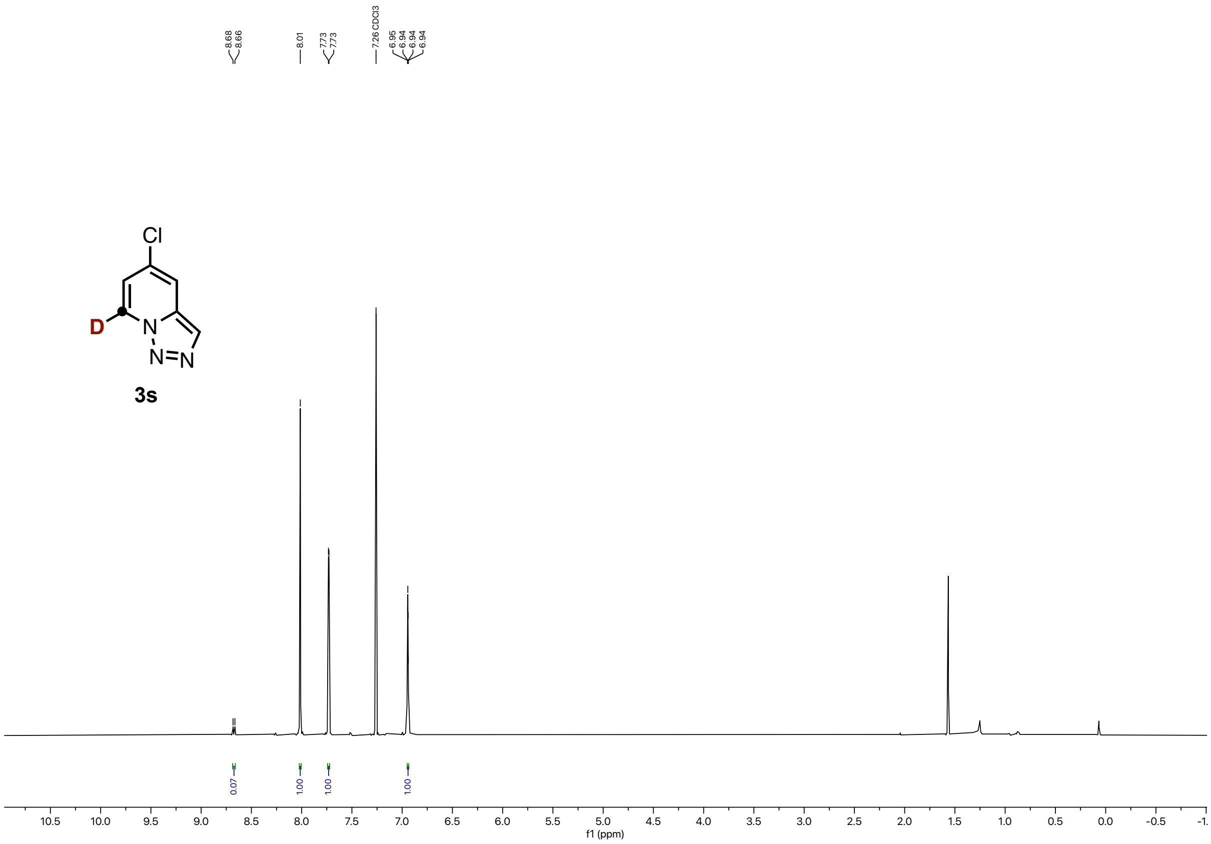

^{1}H spectrum (400 MHz, CD_3Cl) of **3s**

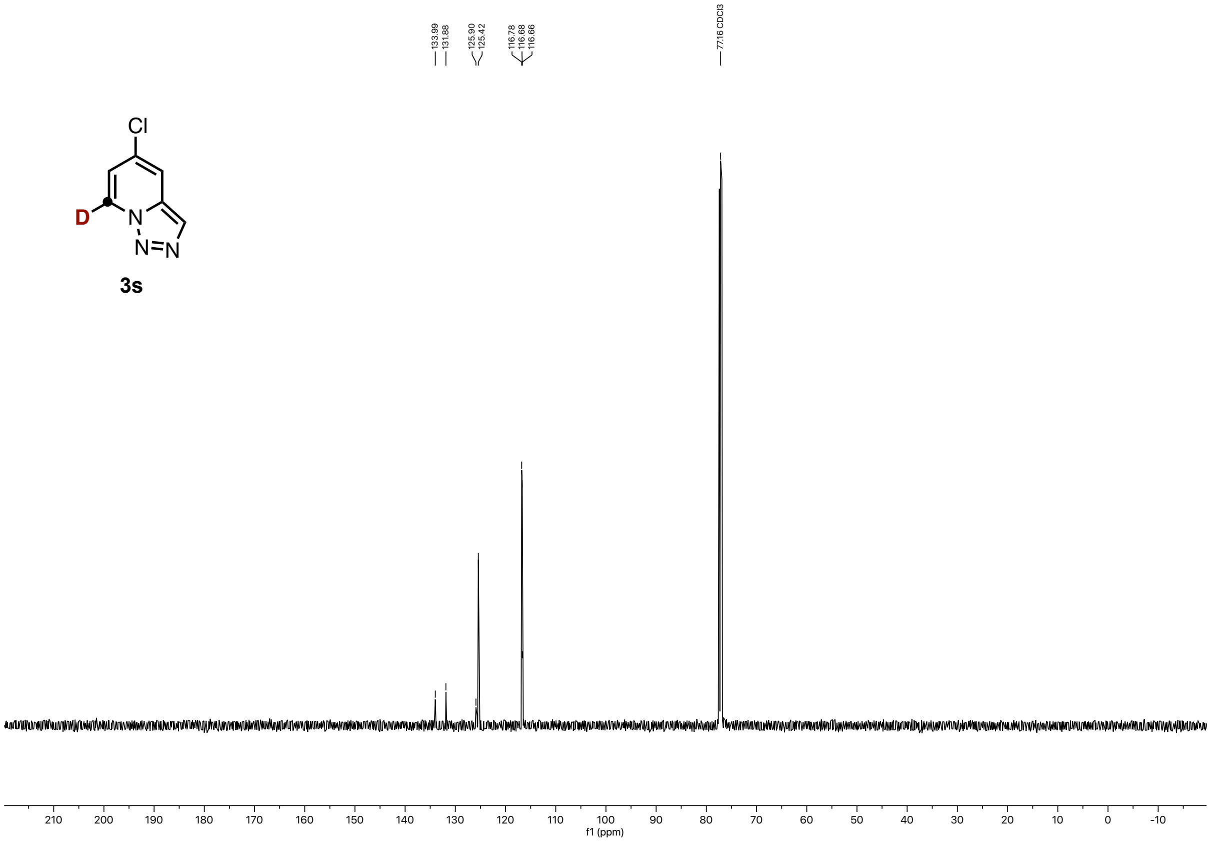

^{13}C spectrum (101 MHz, CD_3Cl) of **3s**

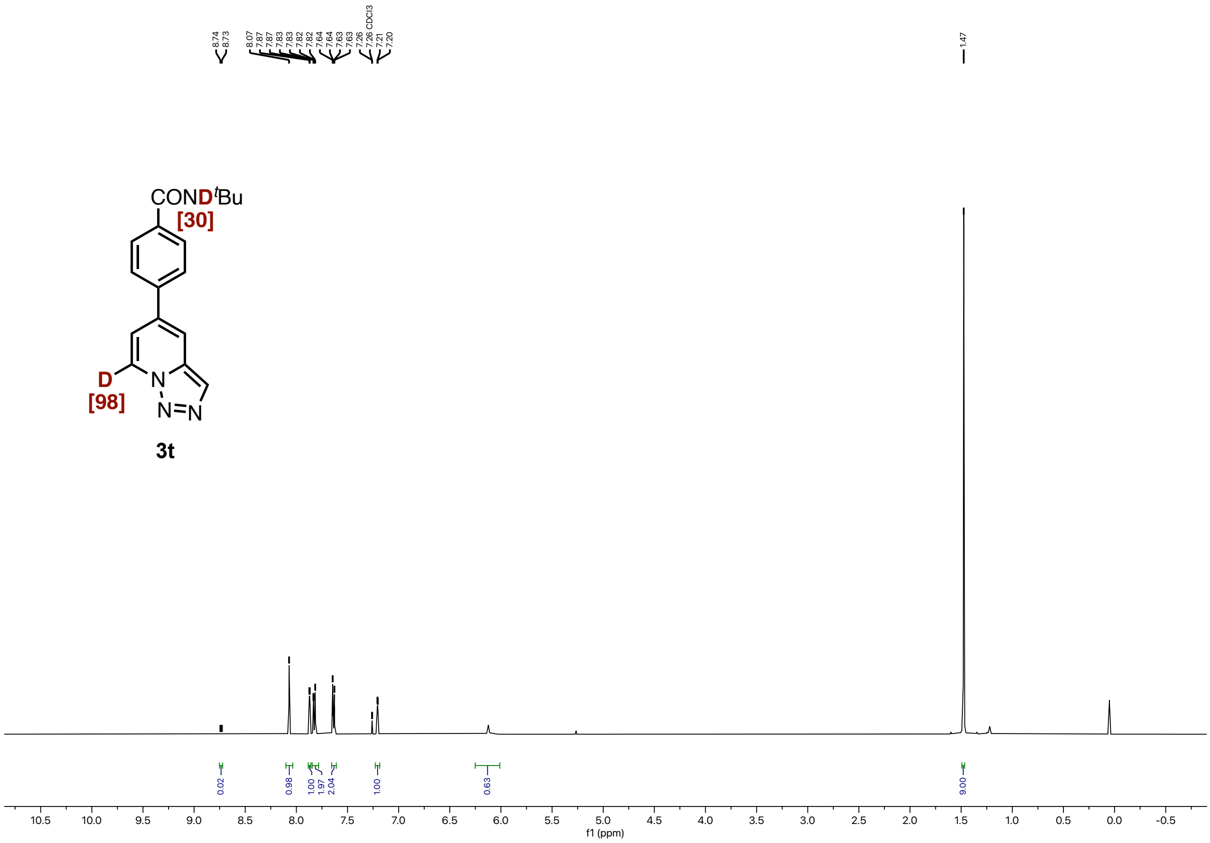

^{1}H spectrum (500 MHz, CD_3Cl) of **3t**

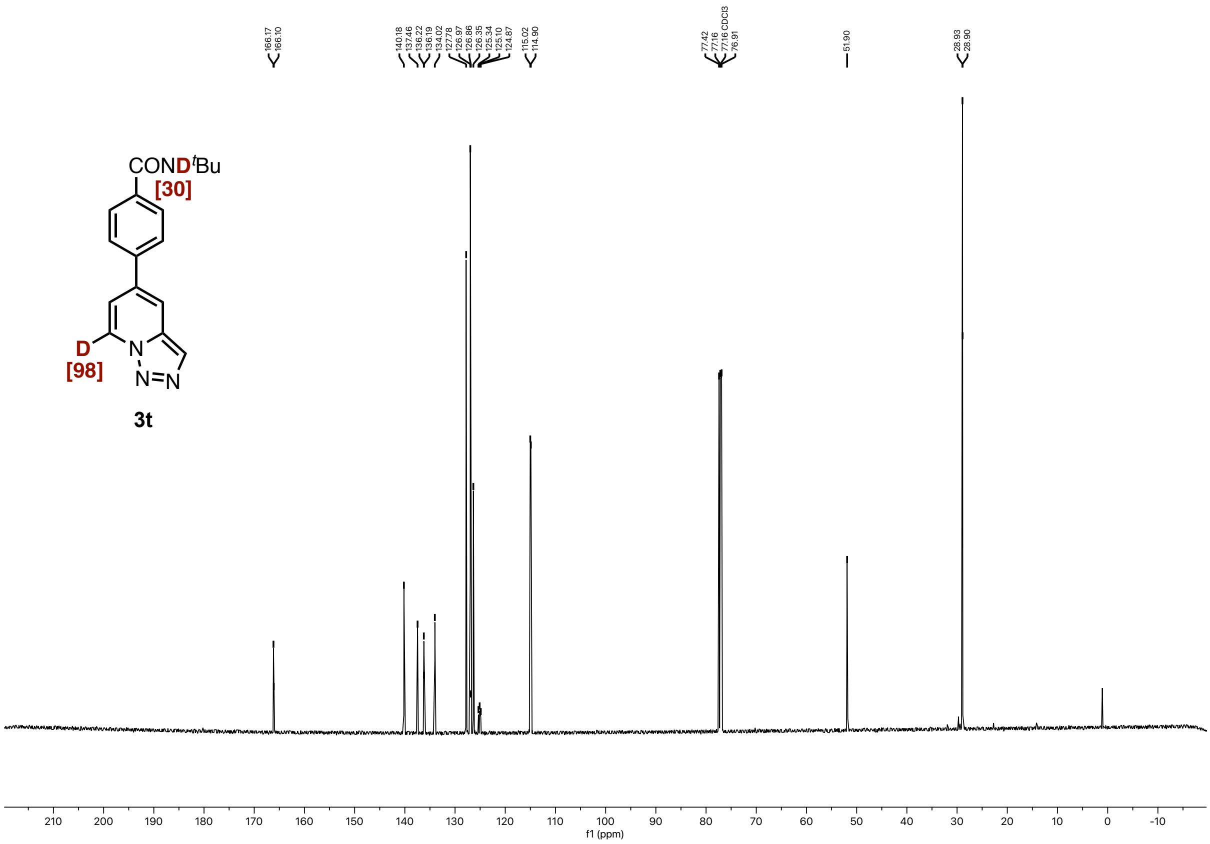

^{13}C spectrum (126 MHz, CD_3Cl) of **3t**

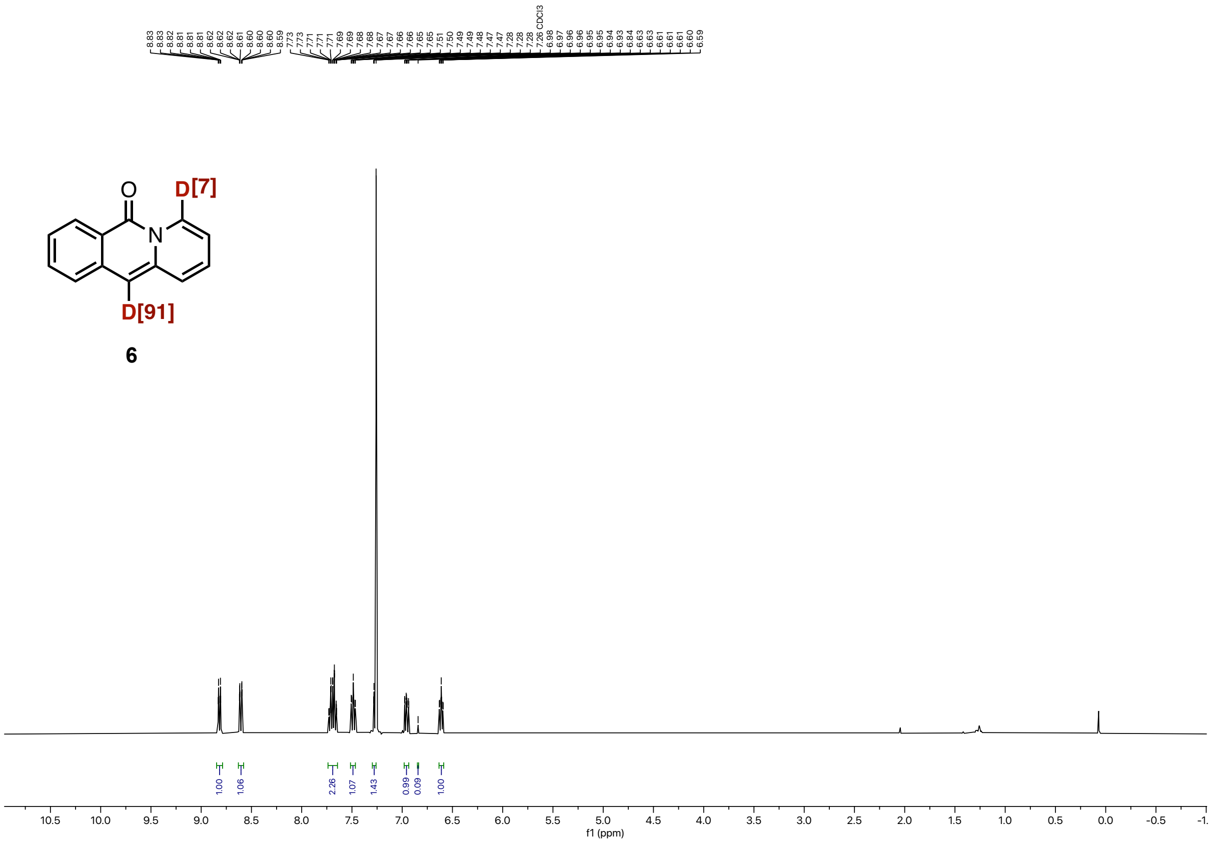

^{1}H spectrum (400 MHz, CD_3Cl) of **6**

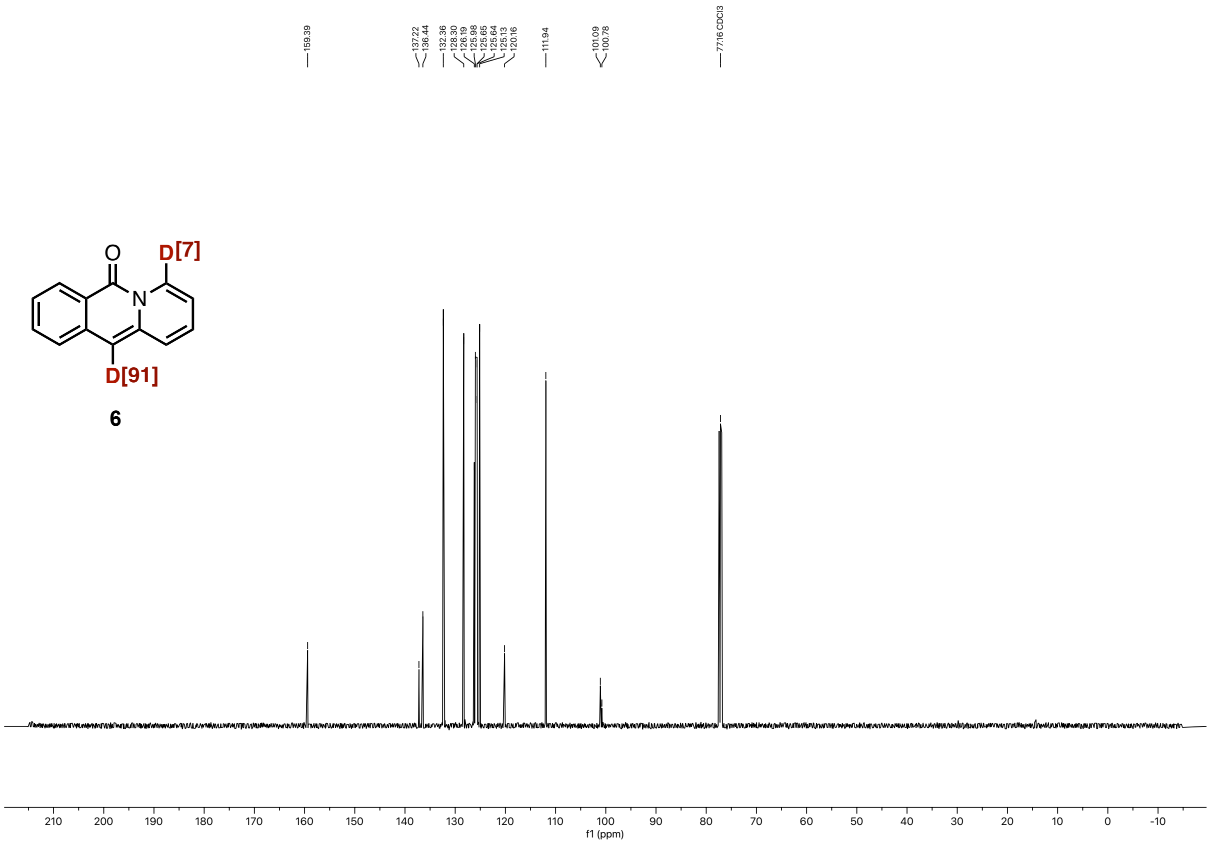

^{13}C spectrum (126 MHz, CD_3Cl) of **6**

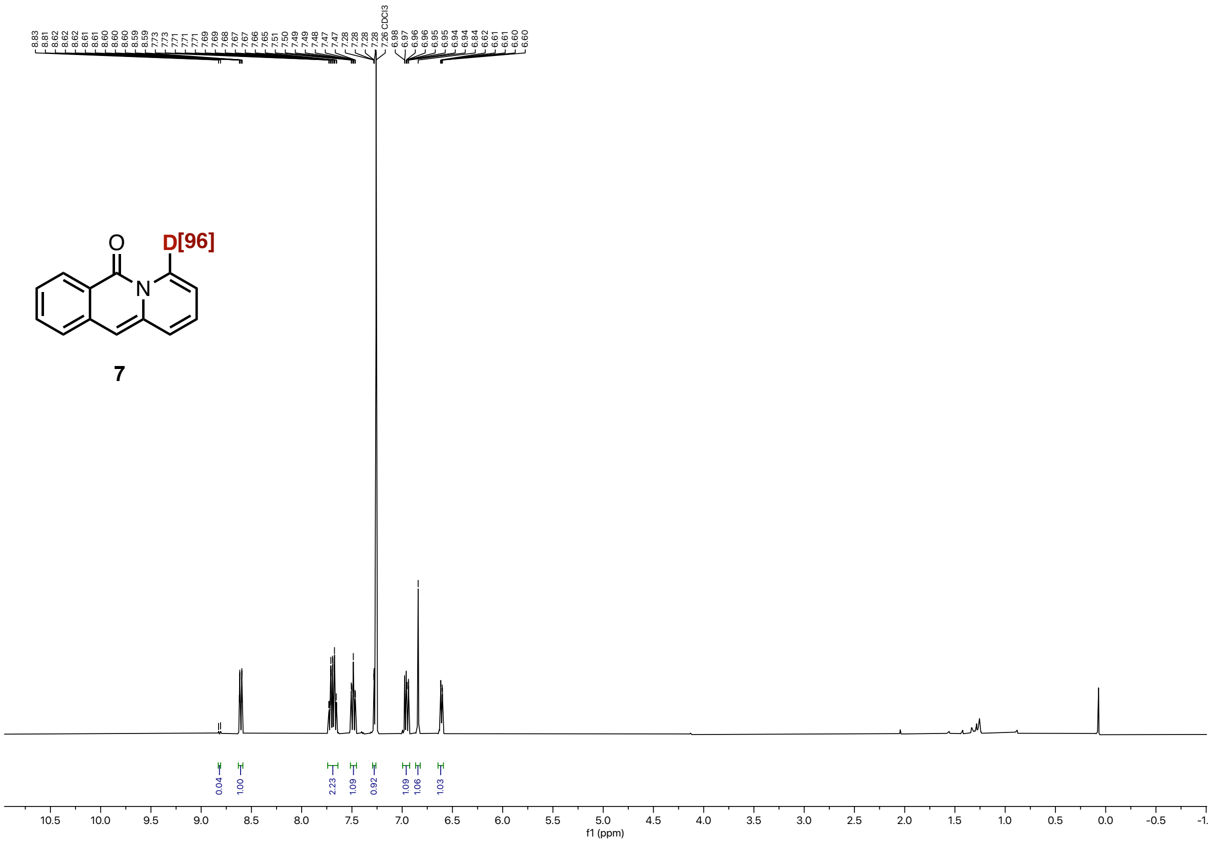

^{1}H spectrum (400 MHz, CD_3Cl) of **7**

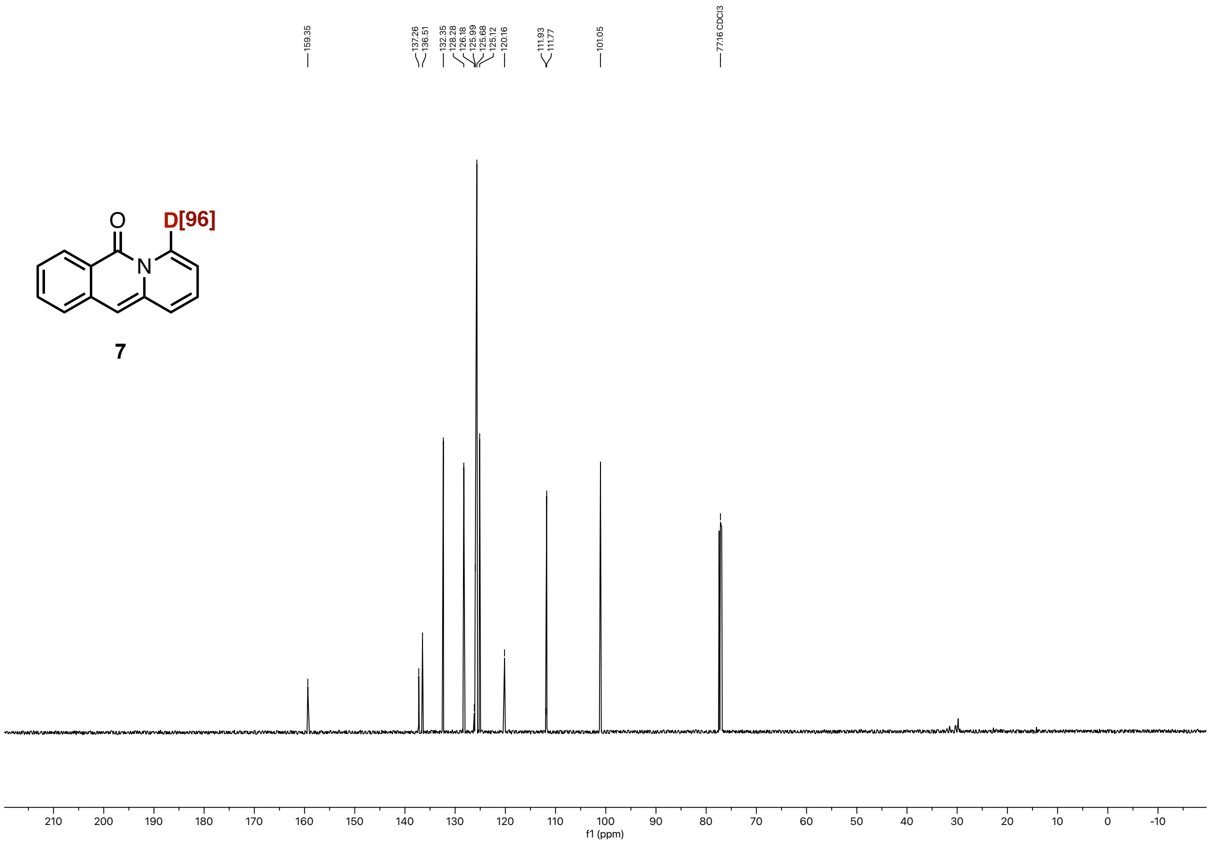

^{13}C spectrum (126 MHz, CD_3Cl) of **7**

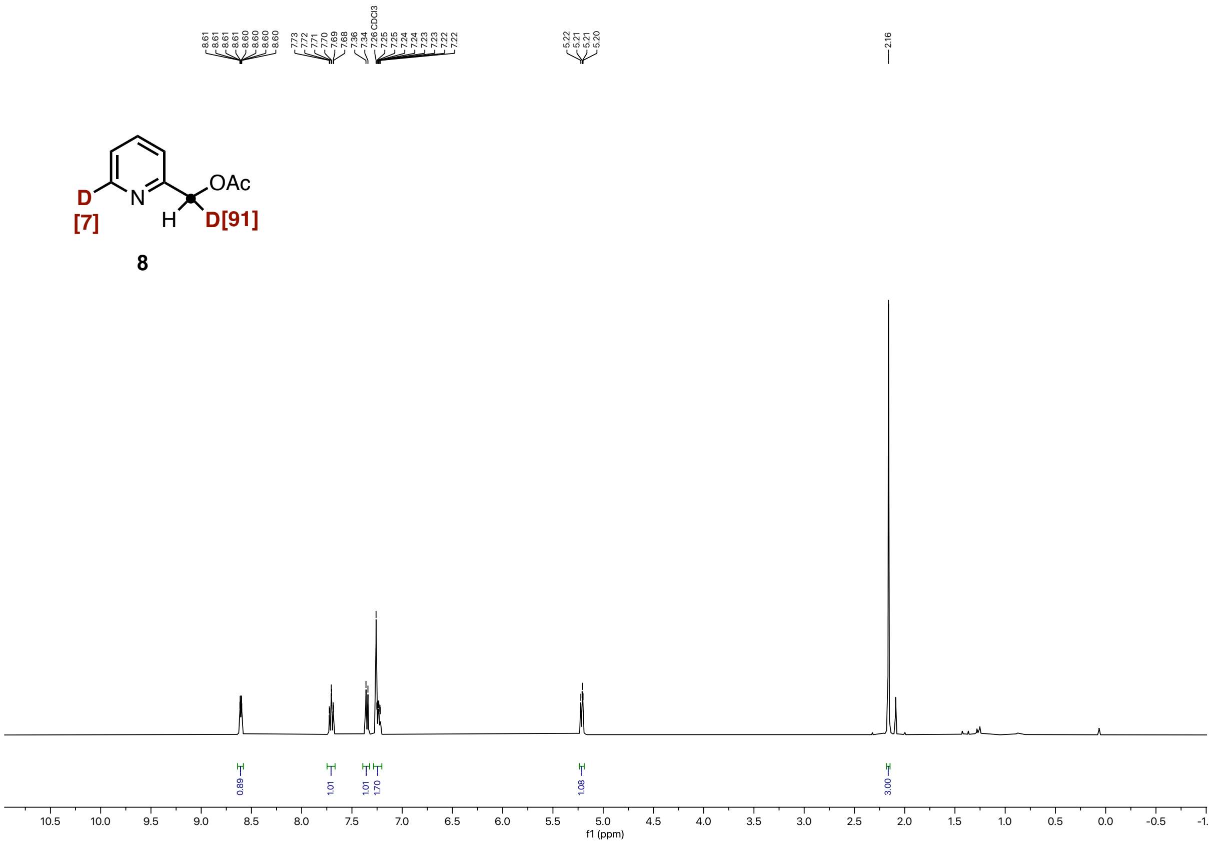

^{1}H spectrum (500 MHz, CD_3Cl) of **8**

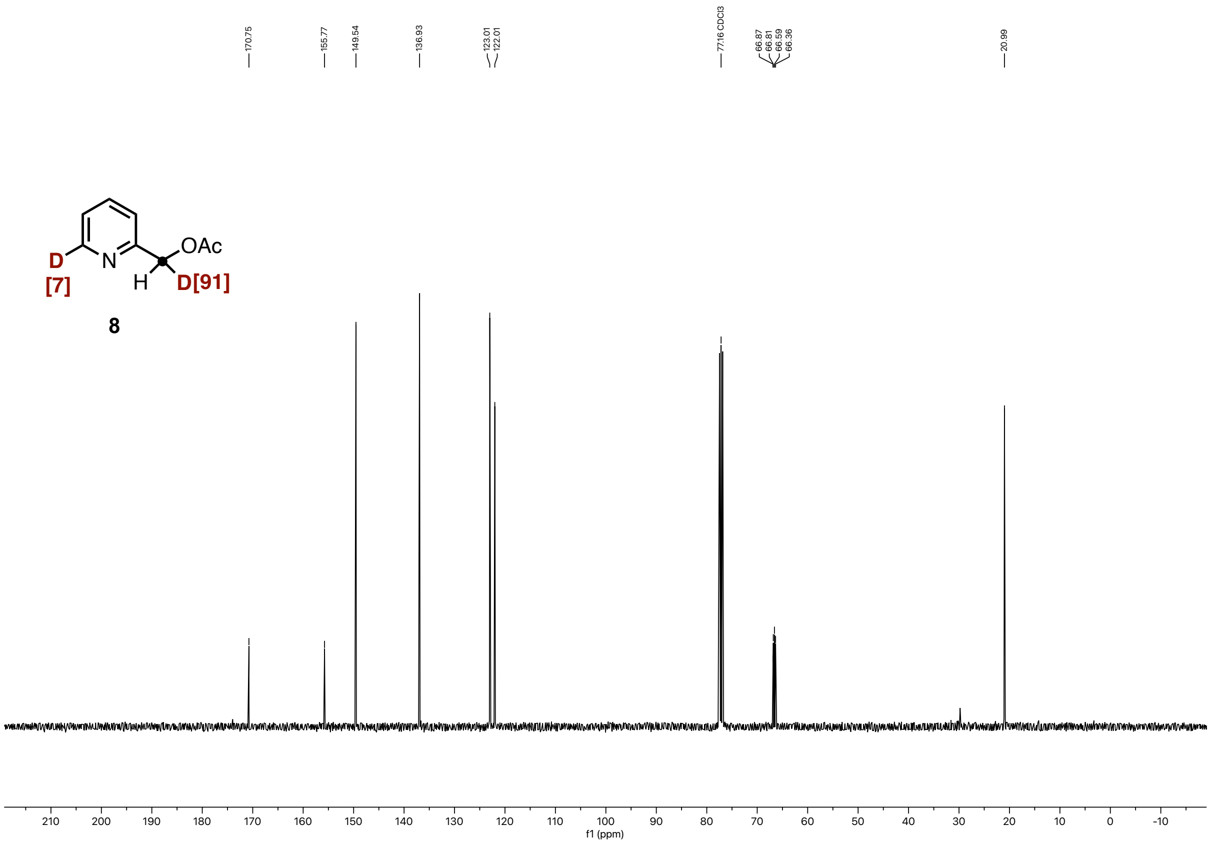

^{13}C spectrum (101 MHz, CD_3Cl) of **8**

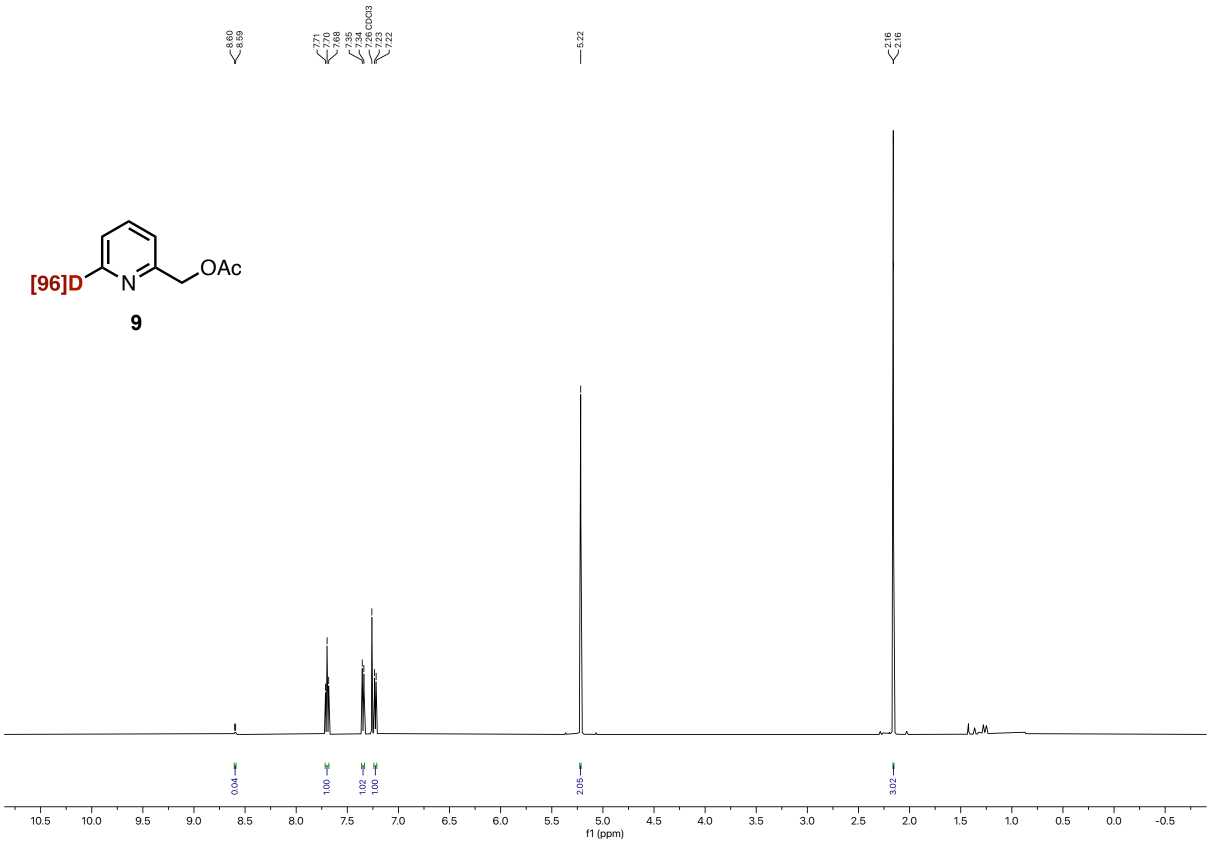

^{1}H spectrum (500 MHz, CD3Cl) of **9**

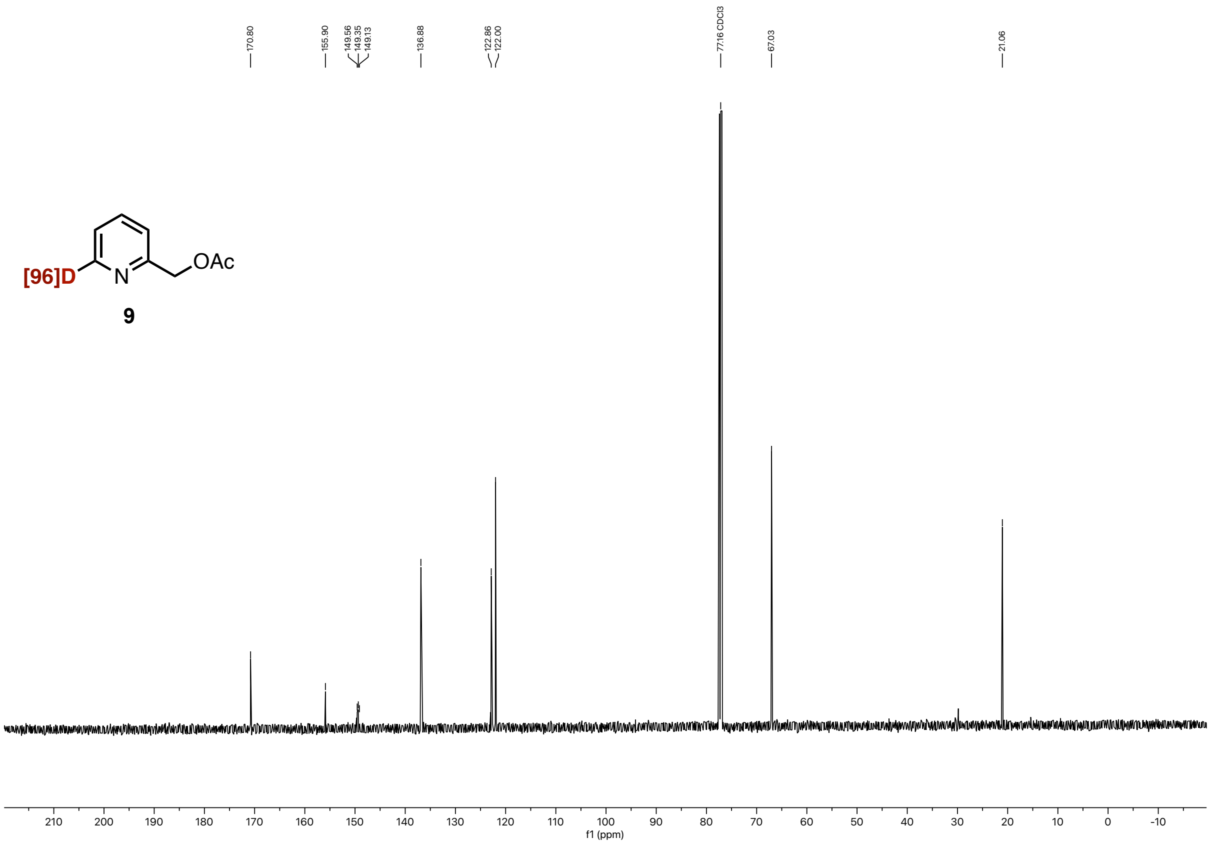

^{13}C spectrum (126 MHz, CD3Cl) of **9**

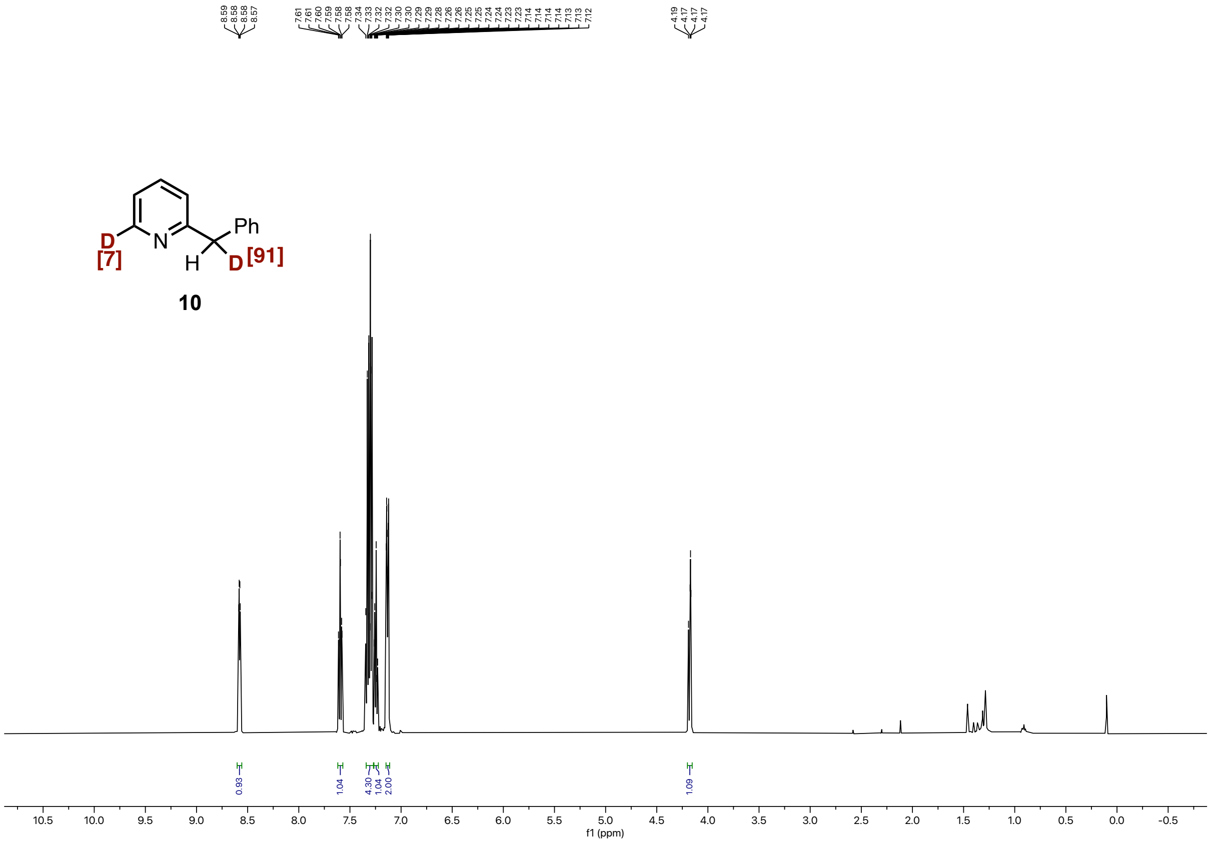

^{1}H spectrum (500 MHz, CD3Cl) of **10**

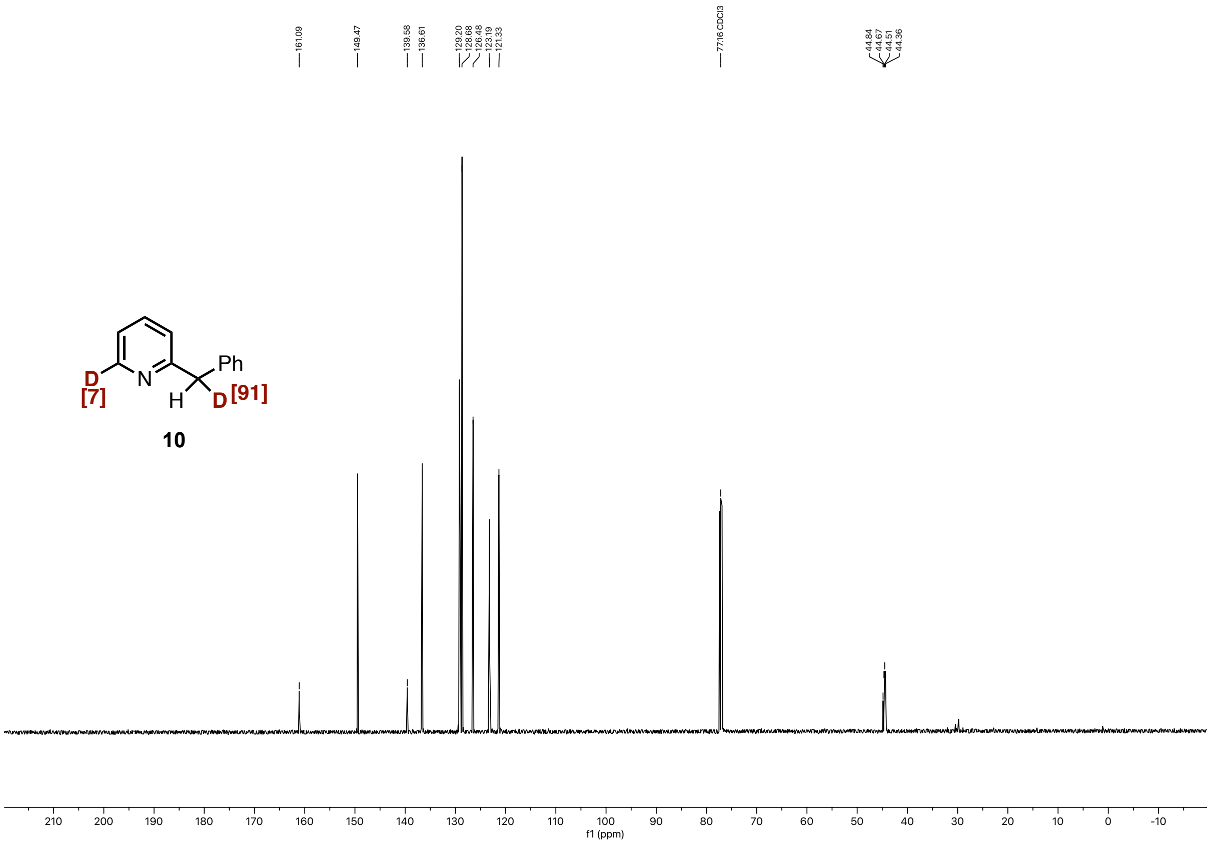

^{13}C spectrum (101 MHz, CD3Cl) of **10**

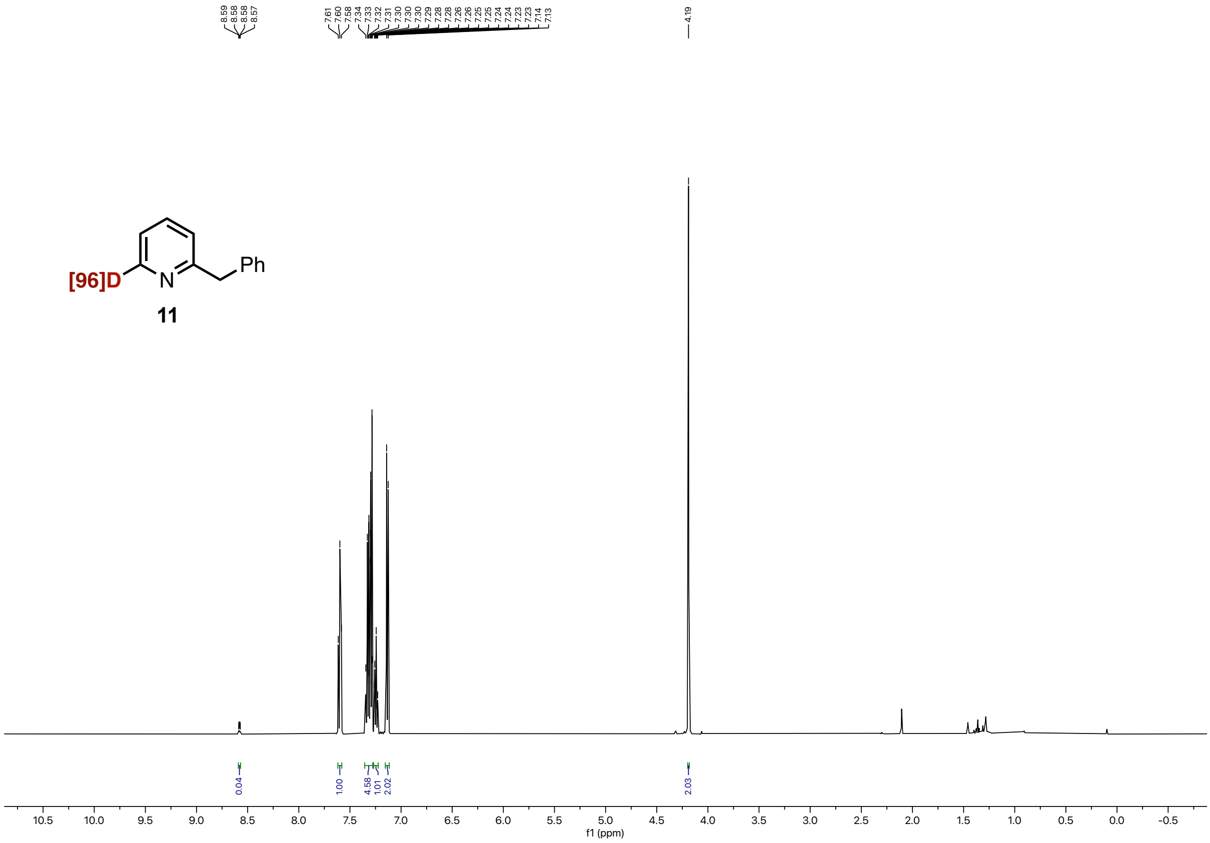

^{1}H spectrum (500 MHz, CD_3Cl) of **11**

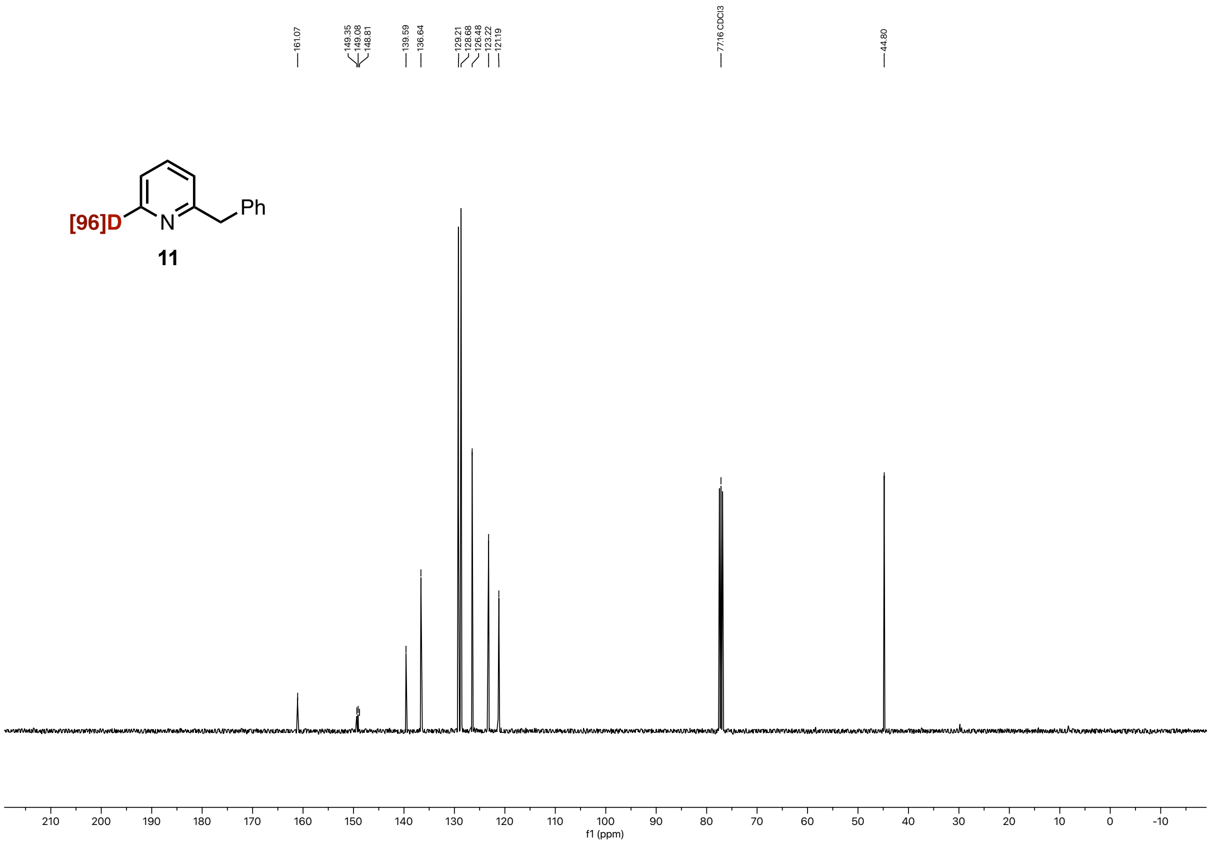

^{13}C spectrum (101 MHz, CD_3Cl) of **11**

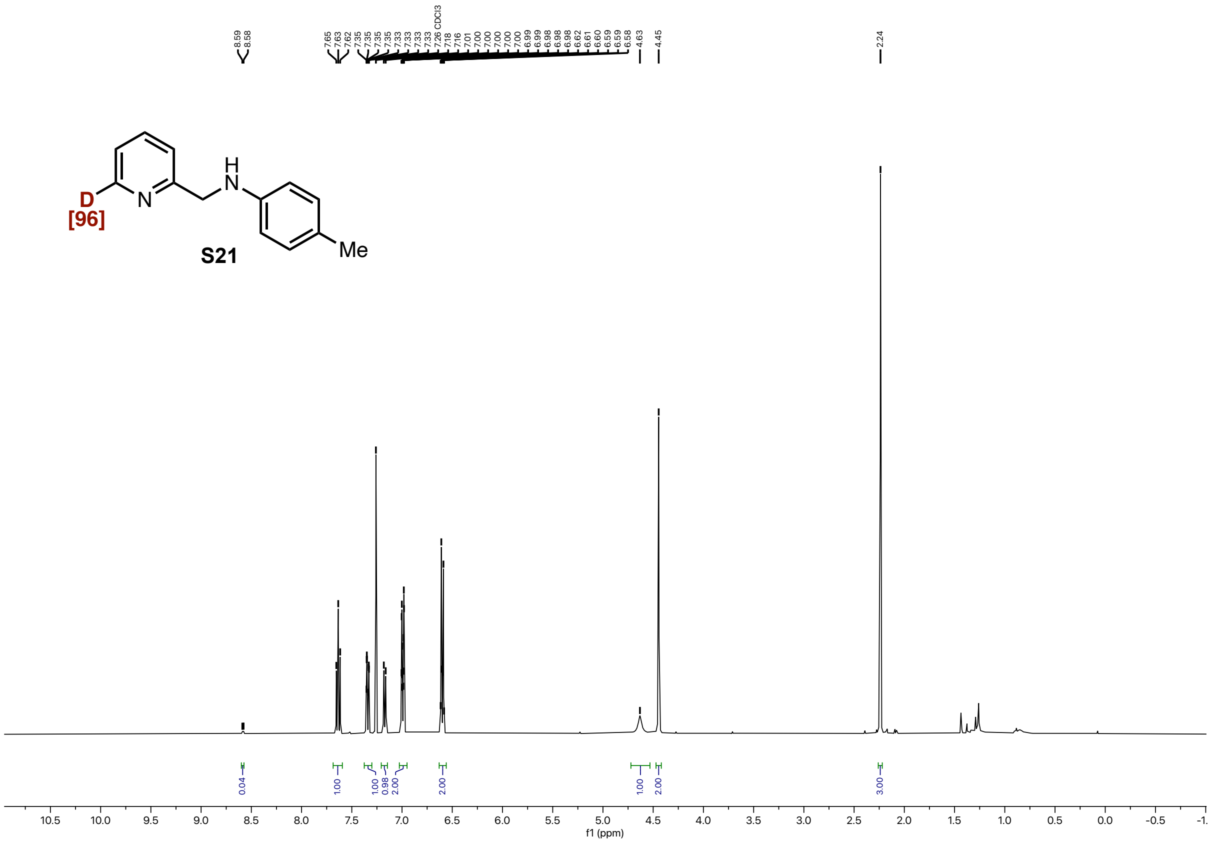

^{1}H spectrum (400 MHz, $CDCl_3$) of **S21**

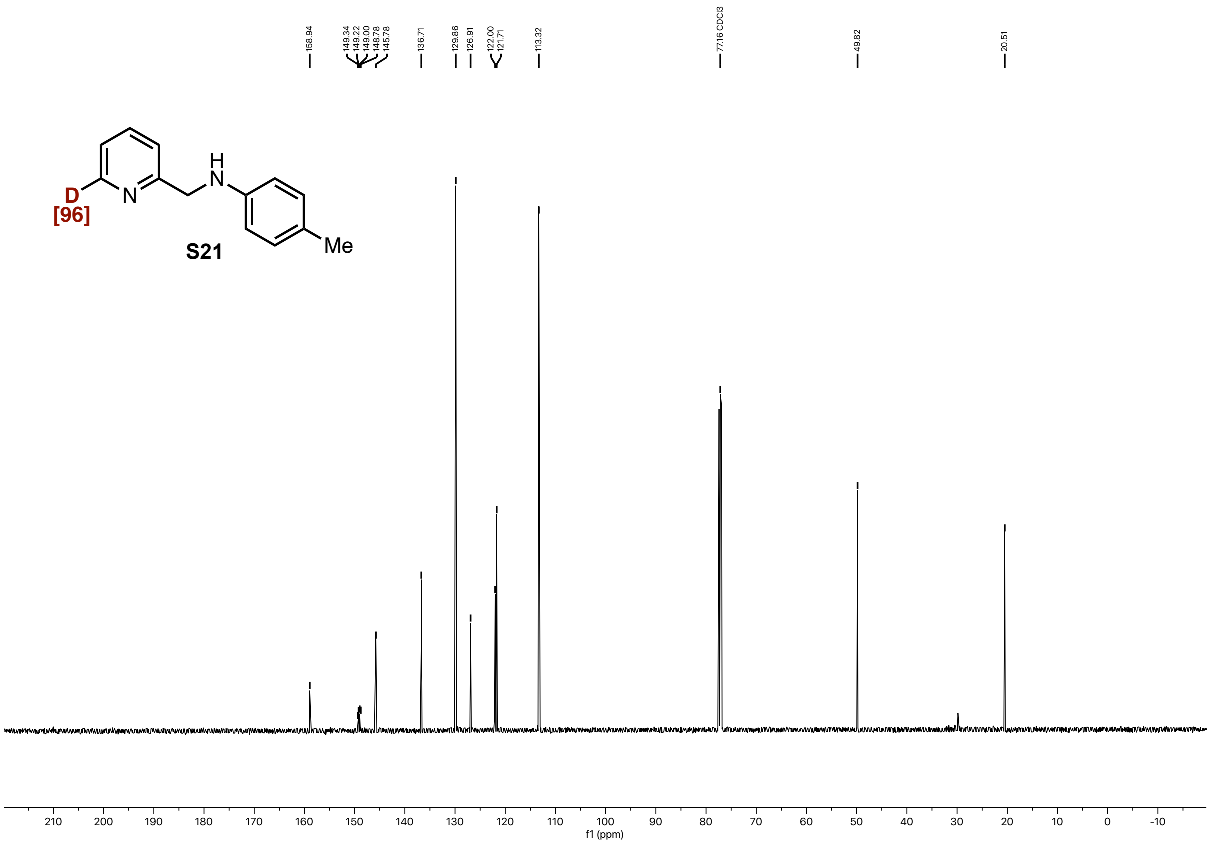

^{13}C NMR (126 MHz, $CDCl_3$) of **S21**

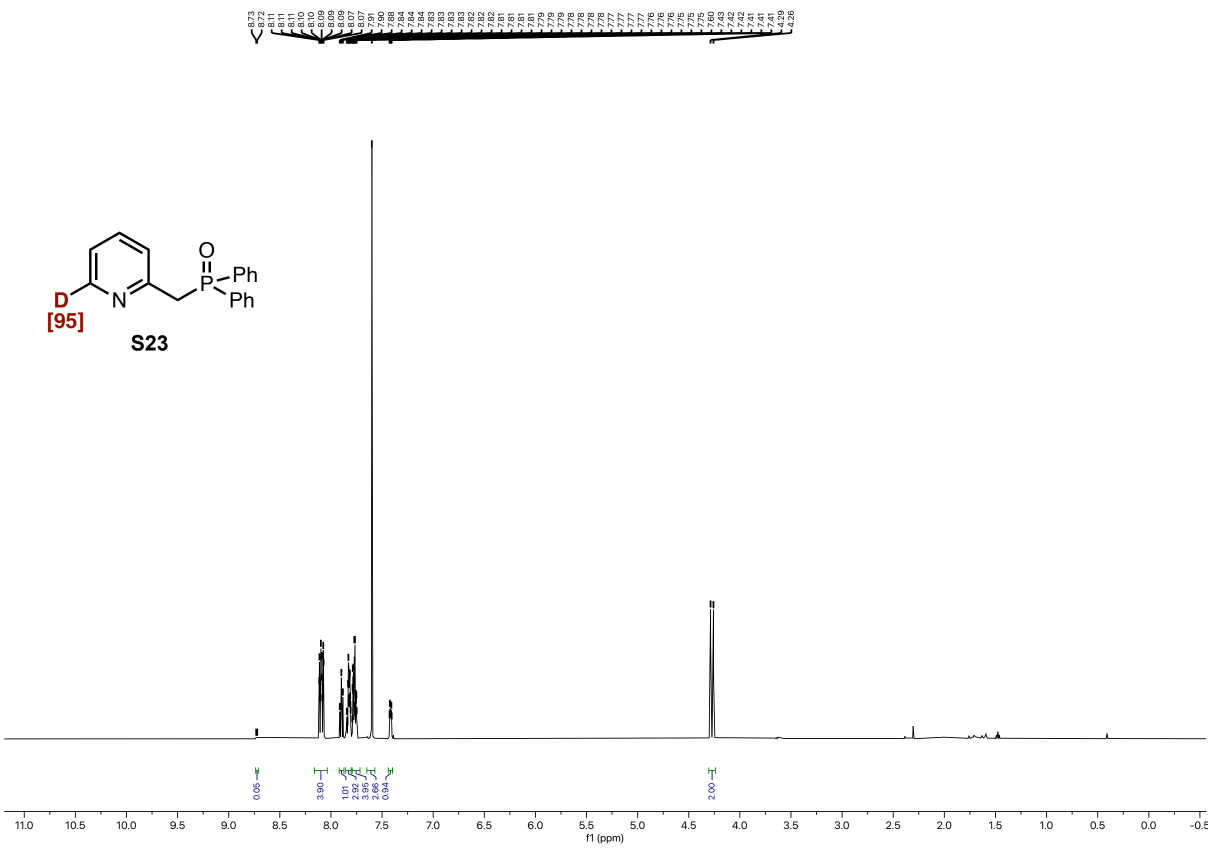

^{1}H NMR (500 MHz, $CDCl_3$) of **S23**

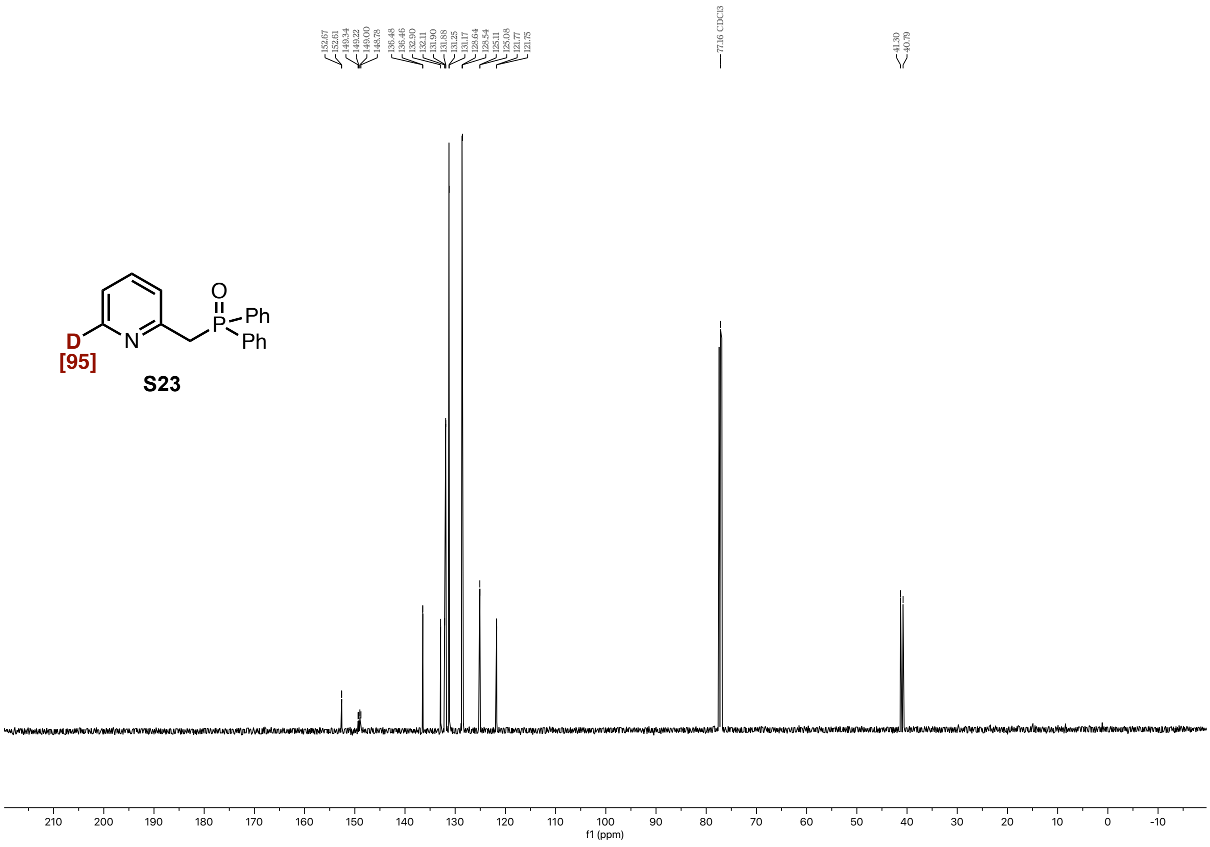

^{13}C NMR (126 MHz, $CDCl_3$) of **S23**

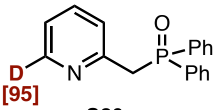

S23

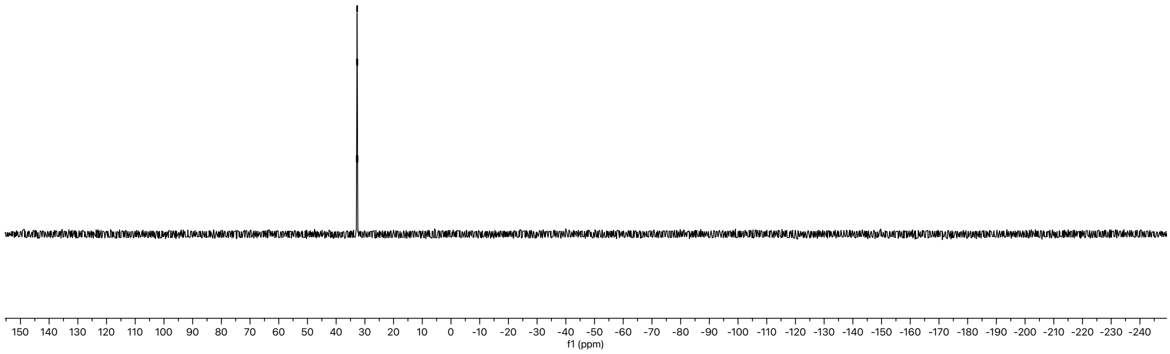

^{31}P NMR (202 MHz, $CDCl_3$) of **S23**

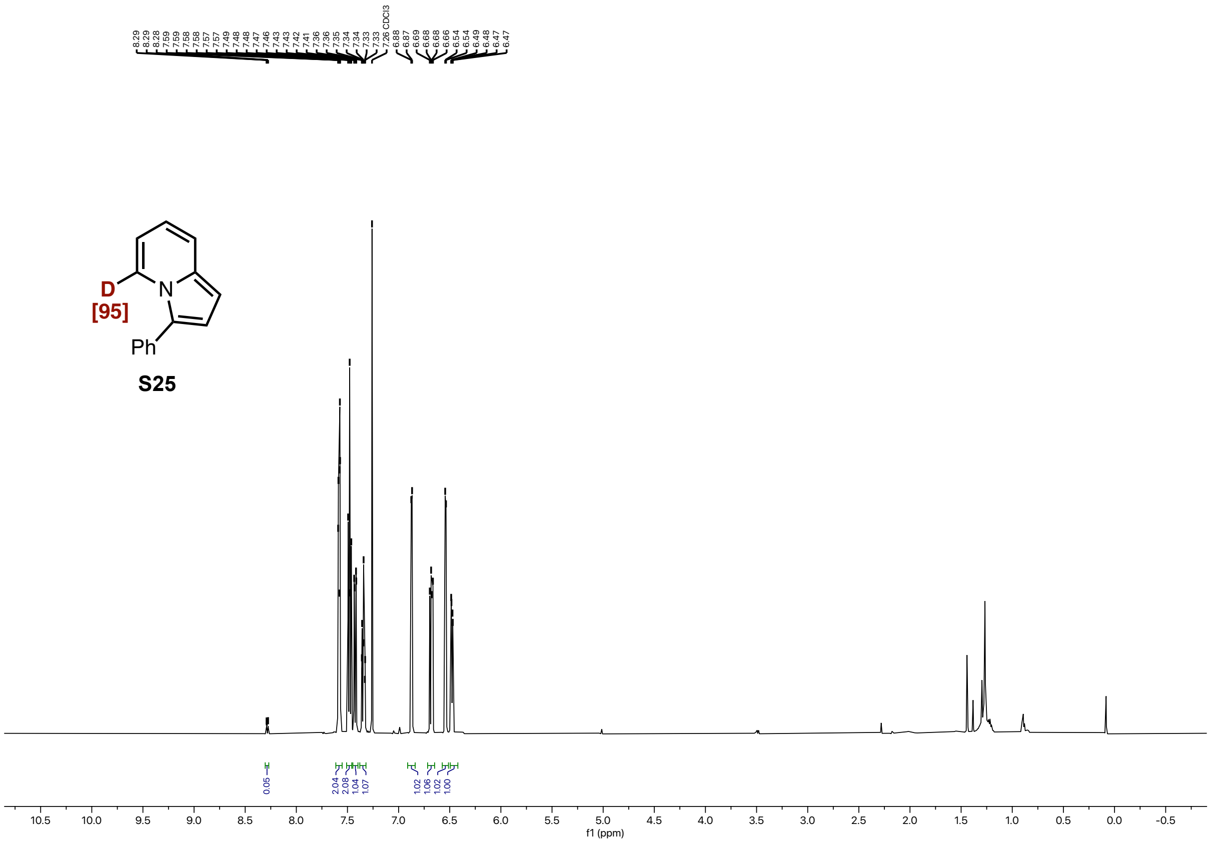

^{1}H NMR (500 MHz, $CDCl_3$) of **S25**

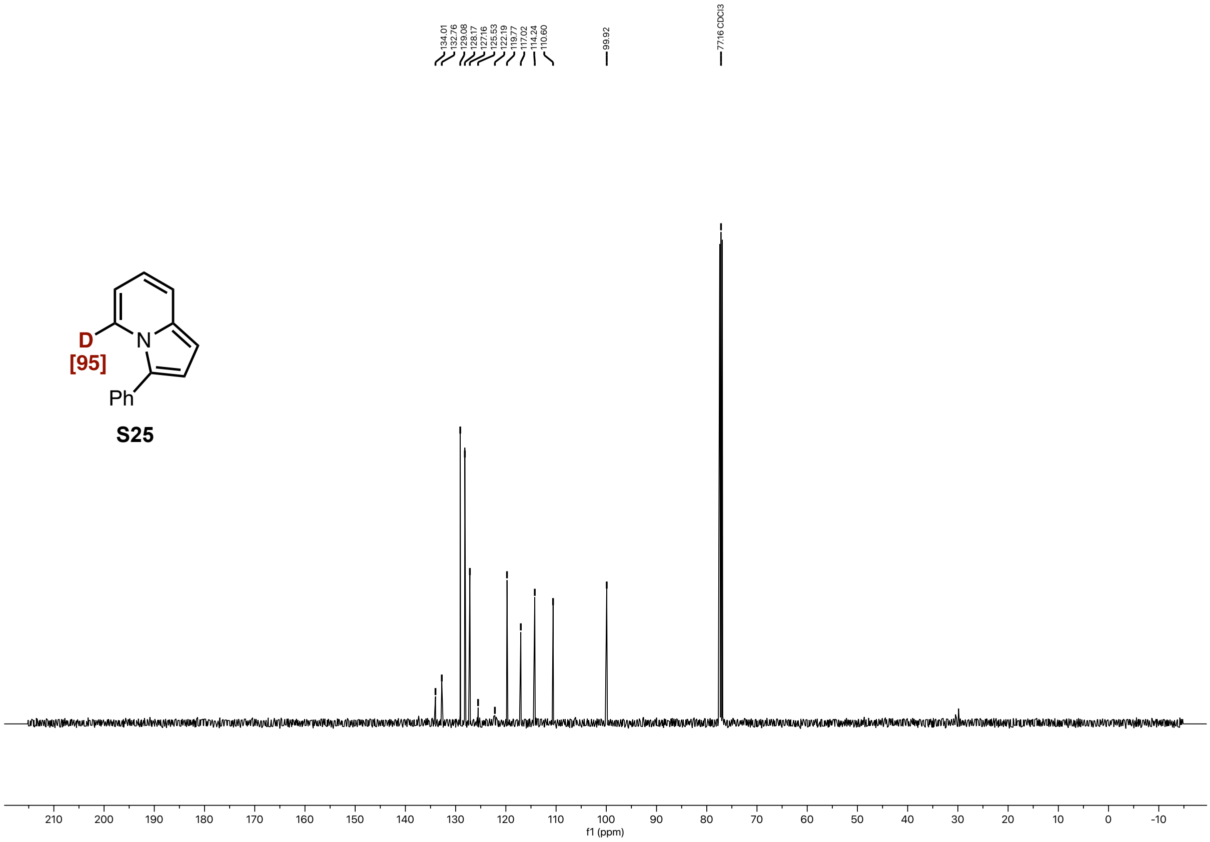

^{13}C NMR (126 MHz, $CDCl_3$) of **S25**

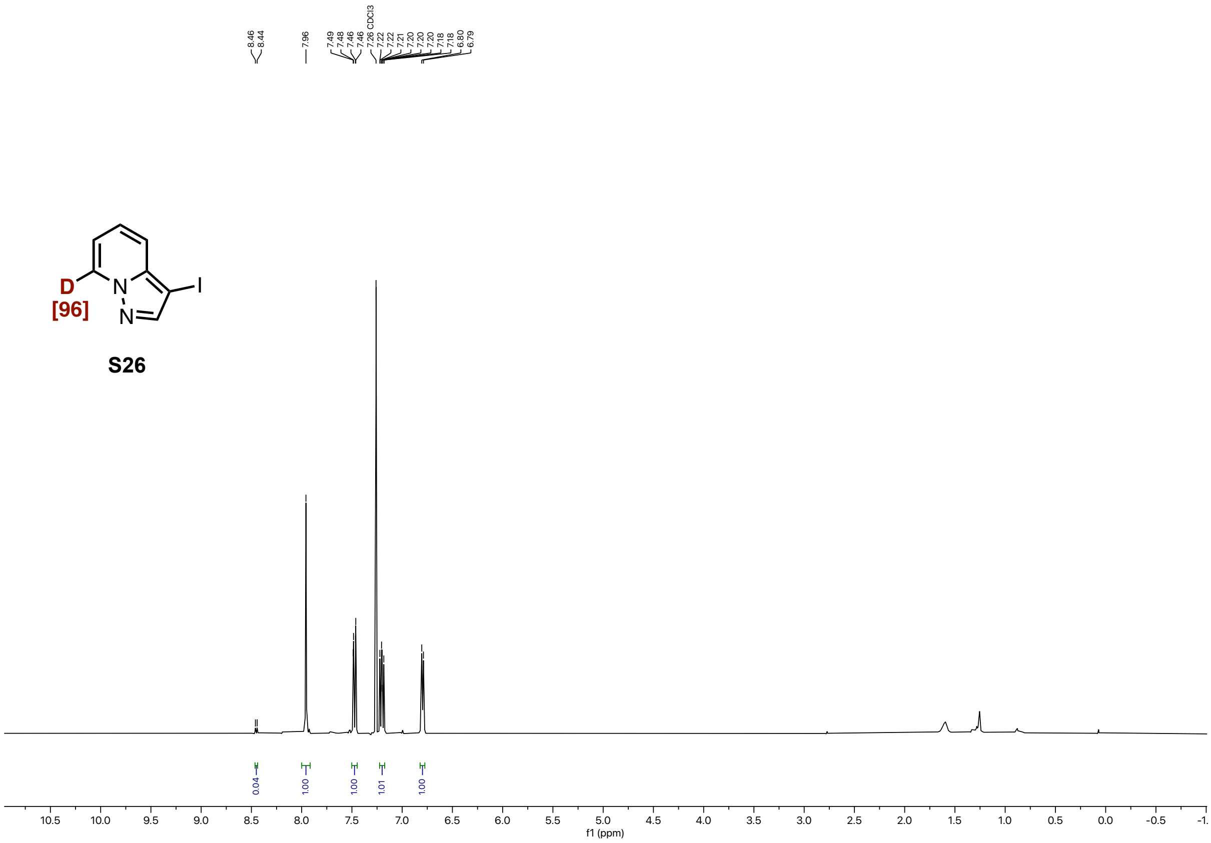

^{1}H spectrum (400 MHz, CD_3Cl) of **S26**

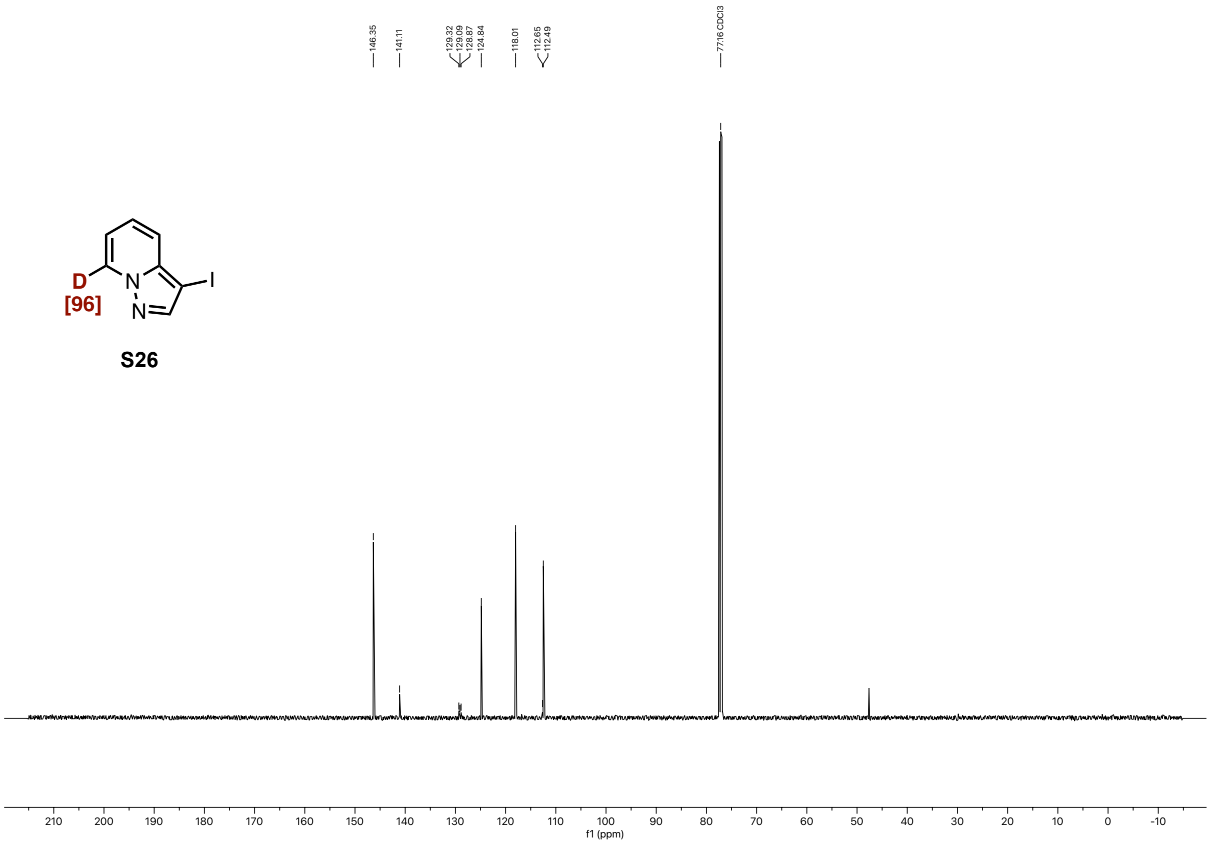

^{13}C spectrum (126 MHz, CD_3Cl) of **S26**

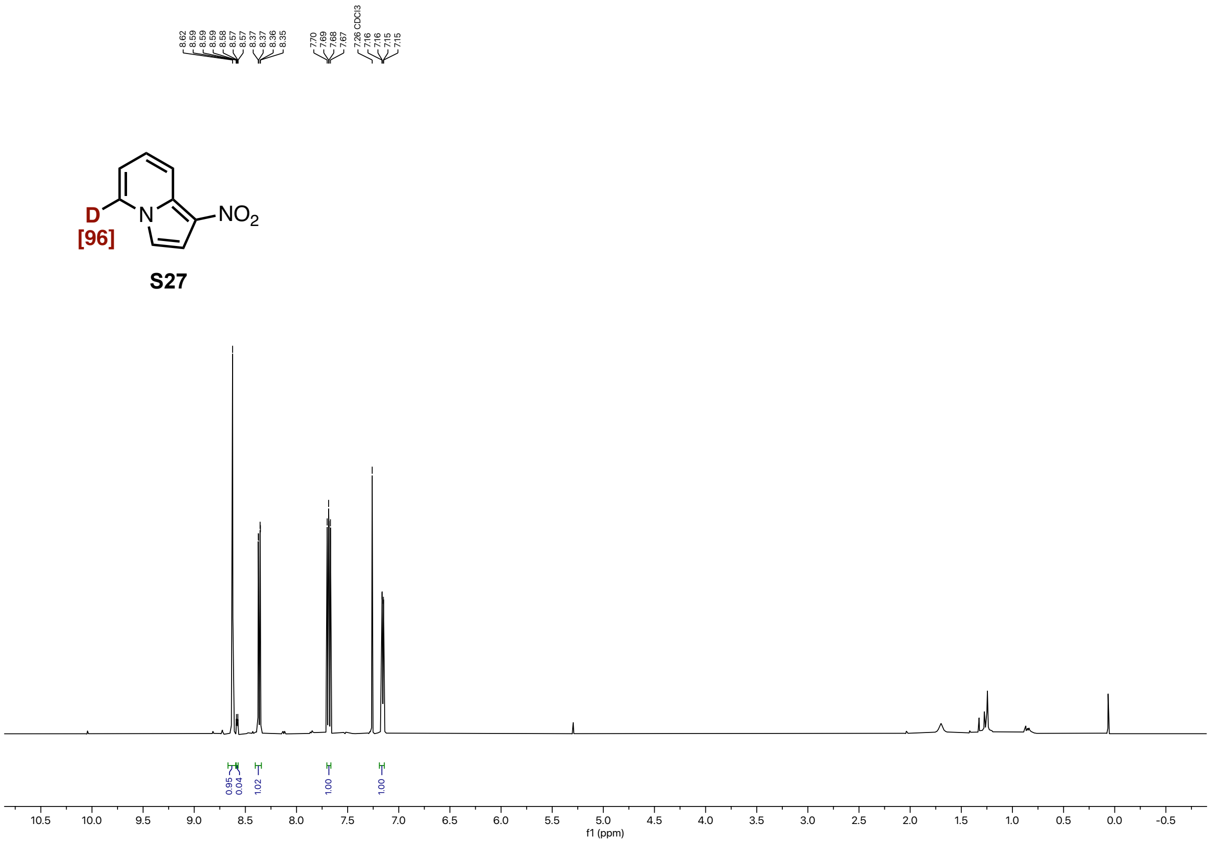

^{1}H spectrum (500 MHz, CD_3Cl) of **S27**

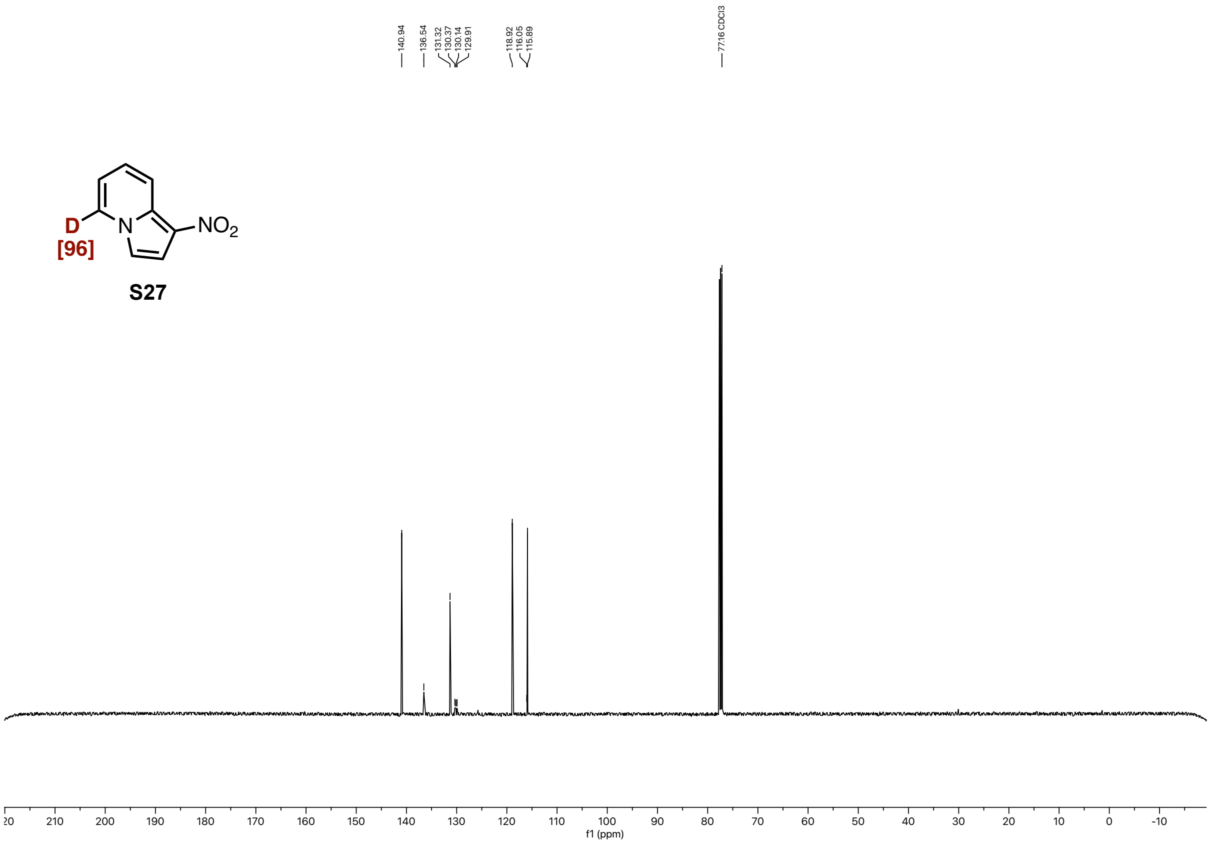

^{13}C spectrum (126 MHz, CD_3Cl) of **S27**

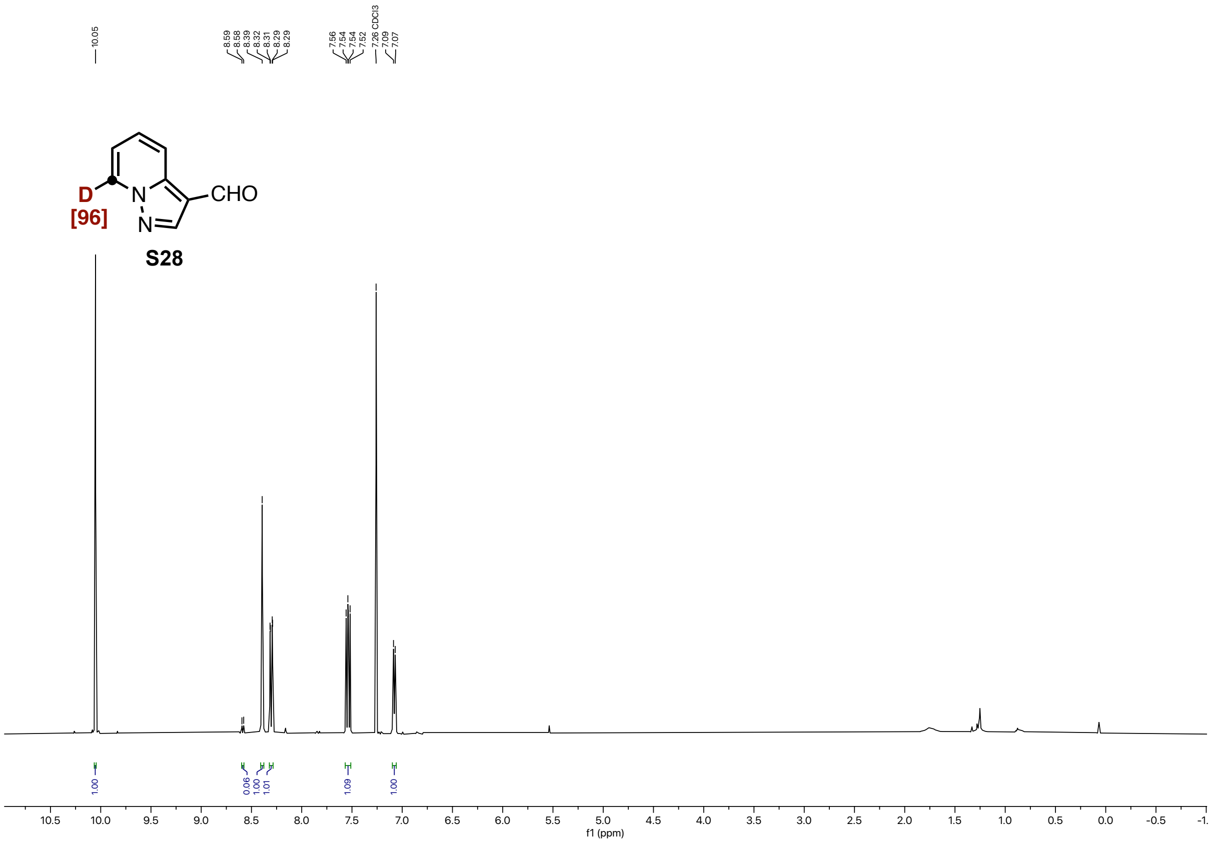

^{1}H spectrum (500 MHz, CD_3Cl) of **S28**

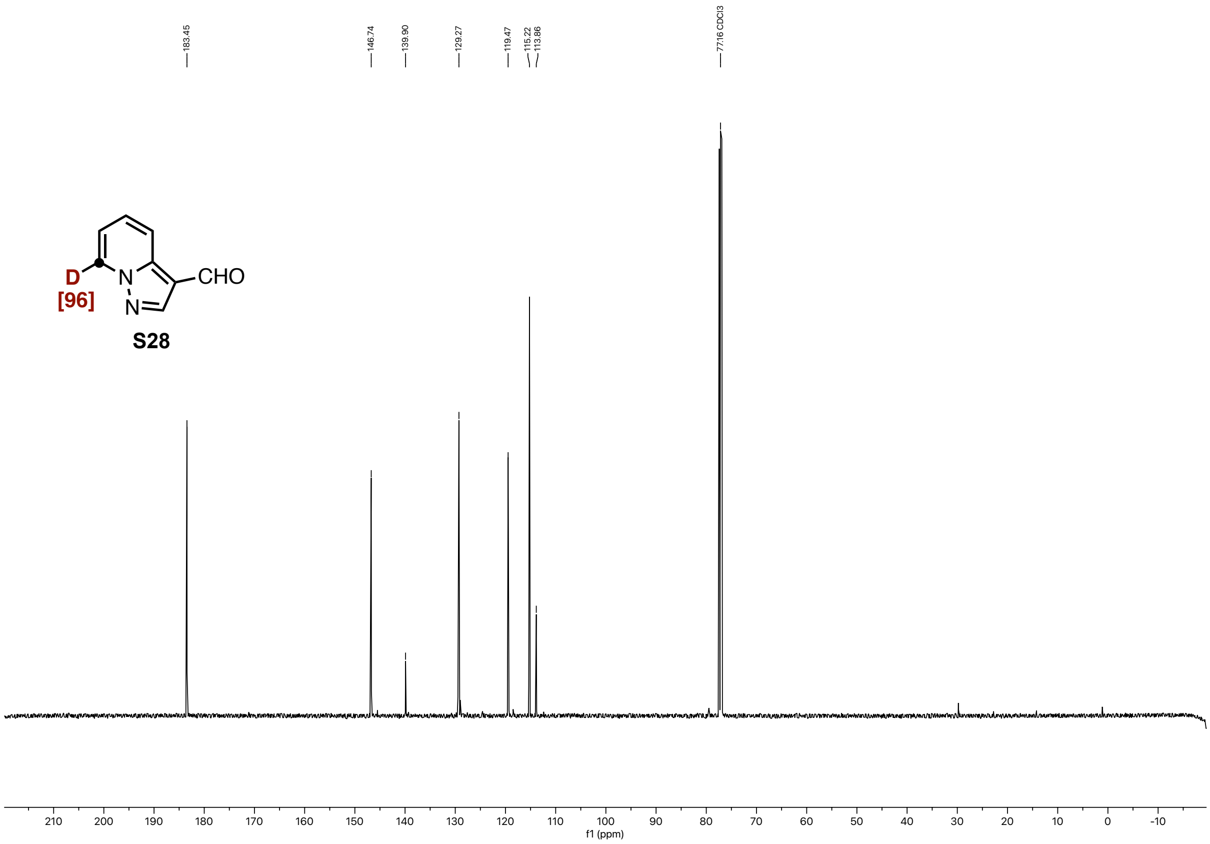

^{13}C spectrum (126 MHz, CD_3Cl) of **S28**

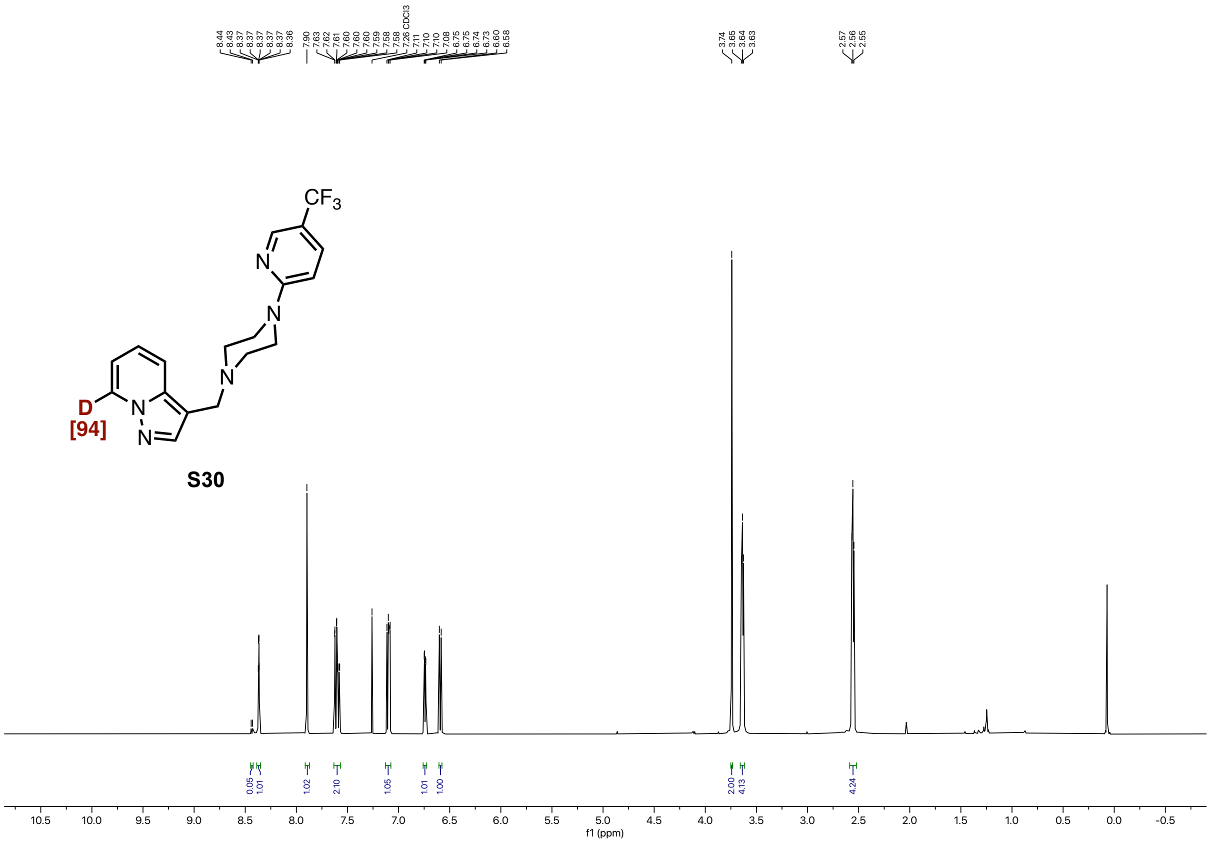

^{1}H spectrum (500 MHz, CD_3Cl) of **S30**

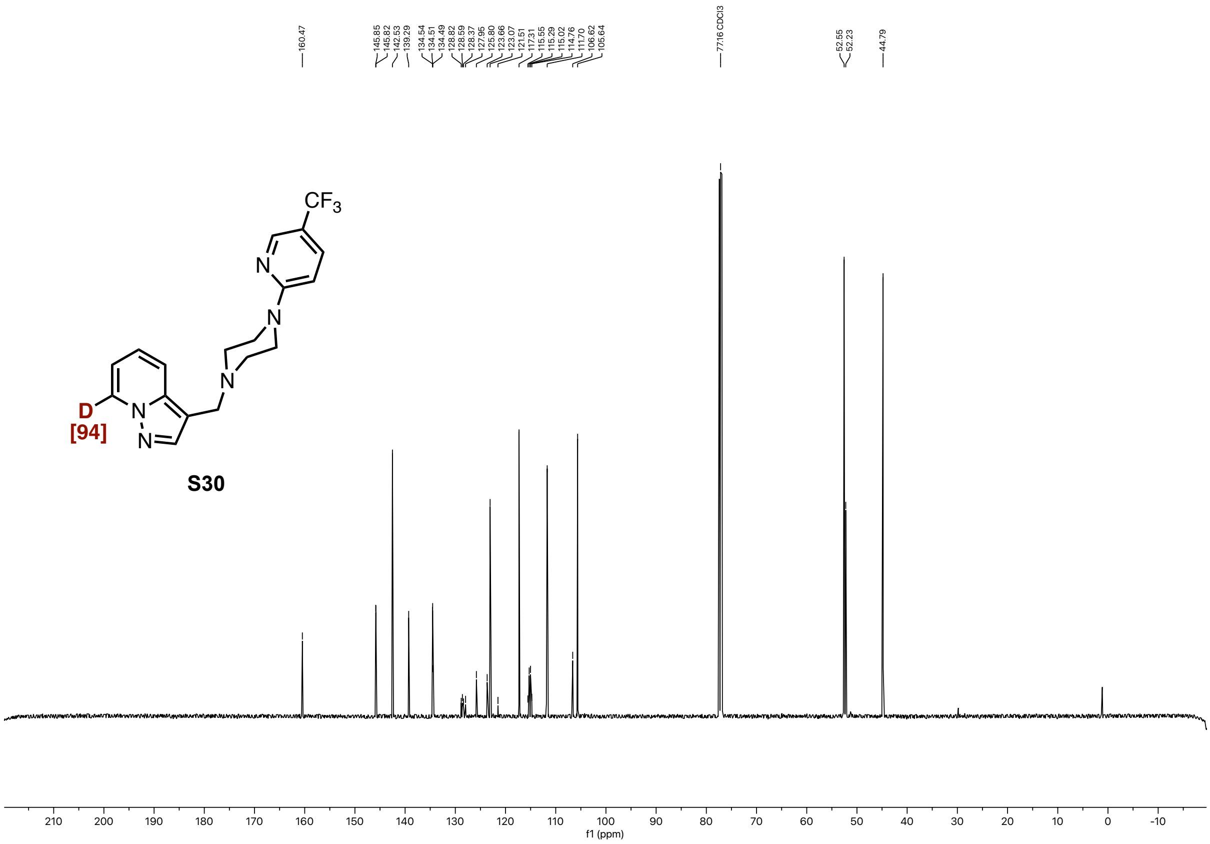

^{13}C spectrum (126 MHz, CD_3Cl) of **S30**

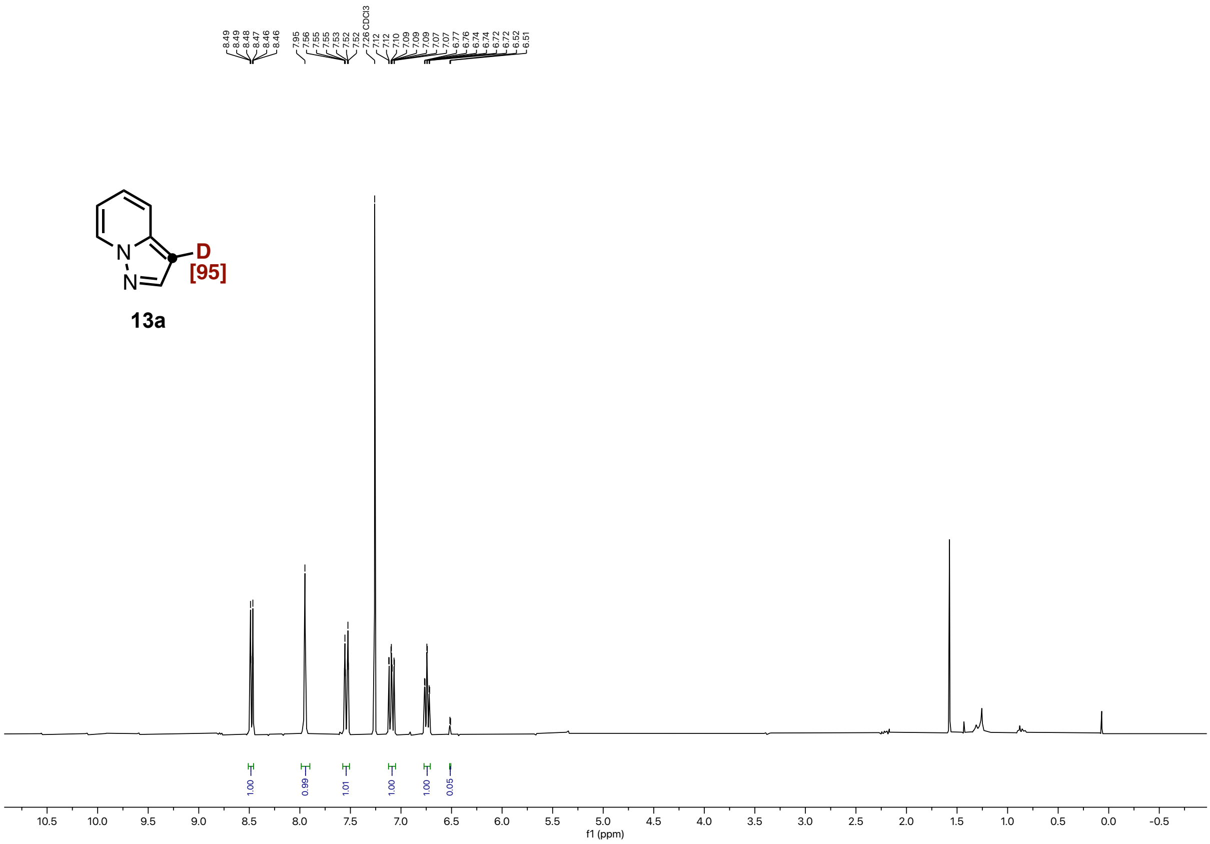

^{1}H spectrum (300 MHz, CD_3Cl) of **13a**

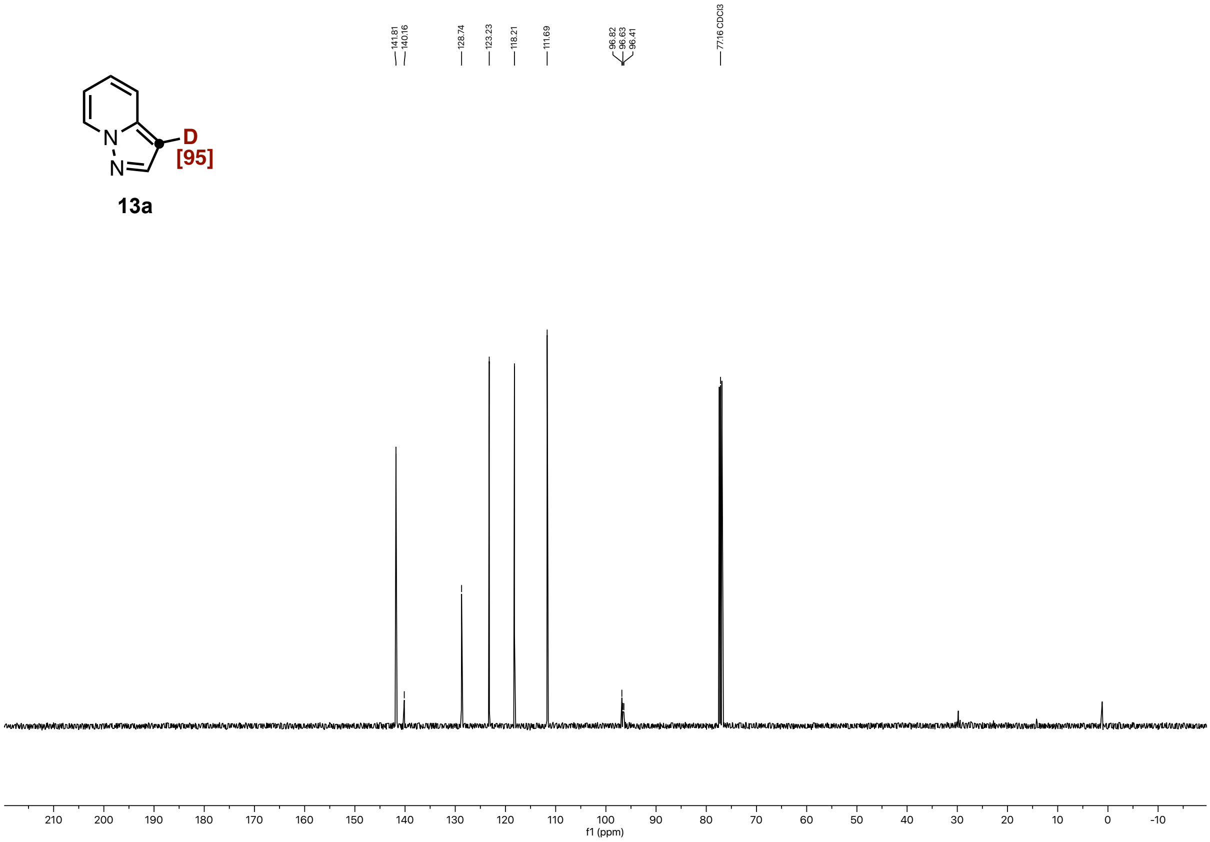

^{13}C spectrum (126 MHz, CD_3Cl) of **13a**

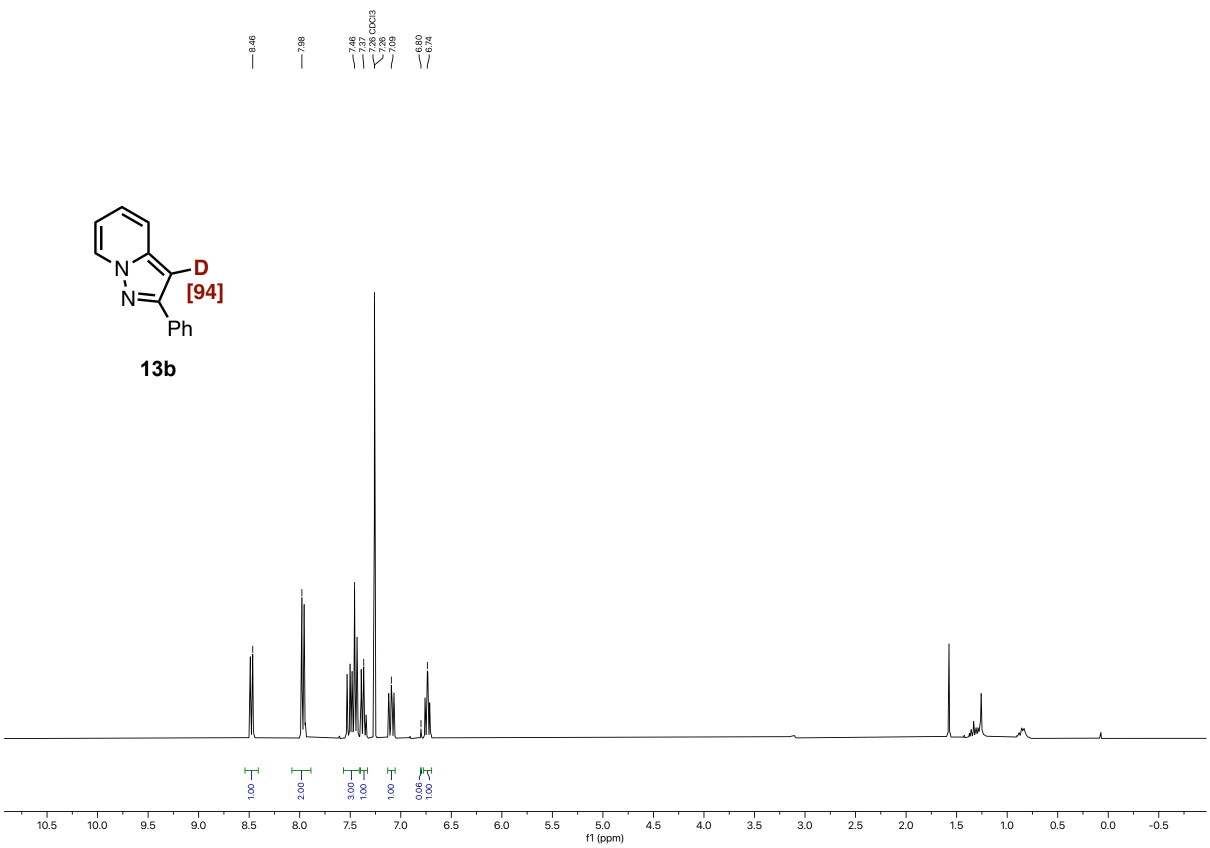

^{1}H spectrum (300 MHz, CD_3Cl) of **13b**

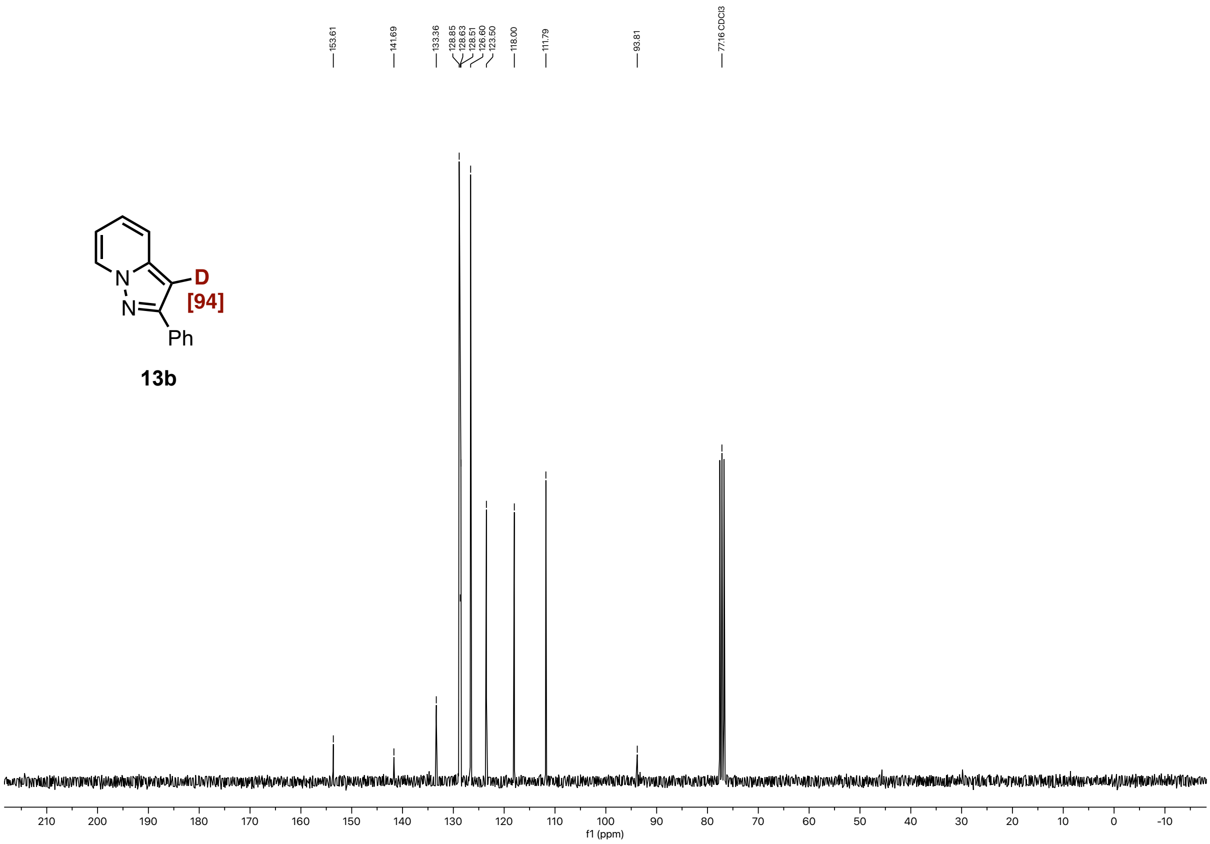

^{13}C spectrum (75 MHz, CD_3Cl) of **13b**

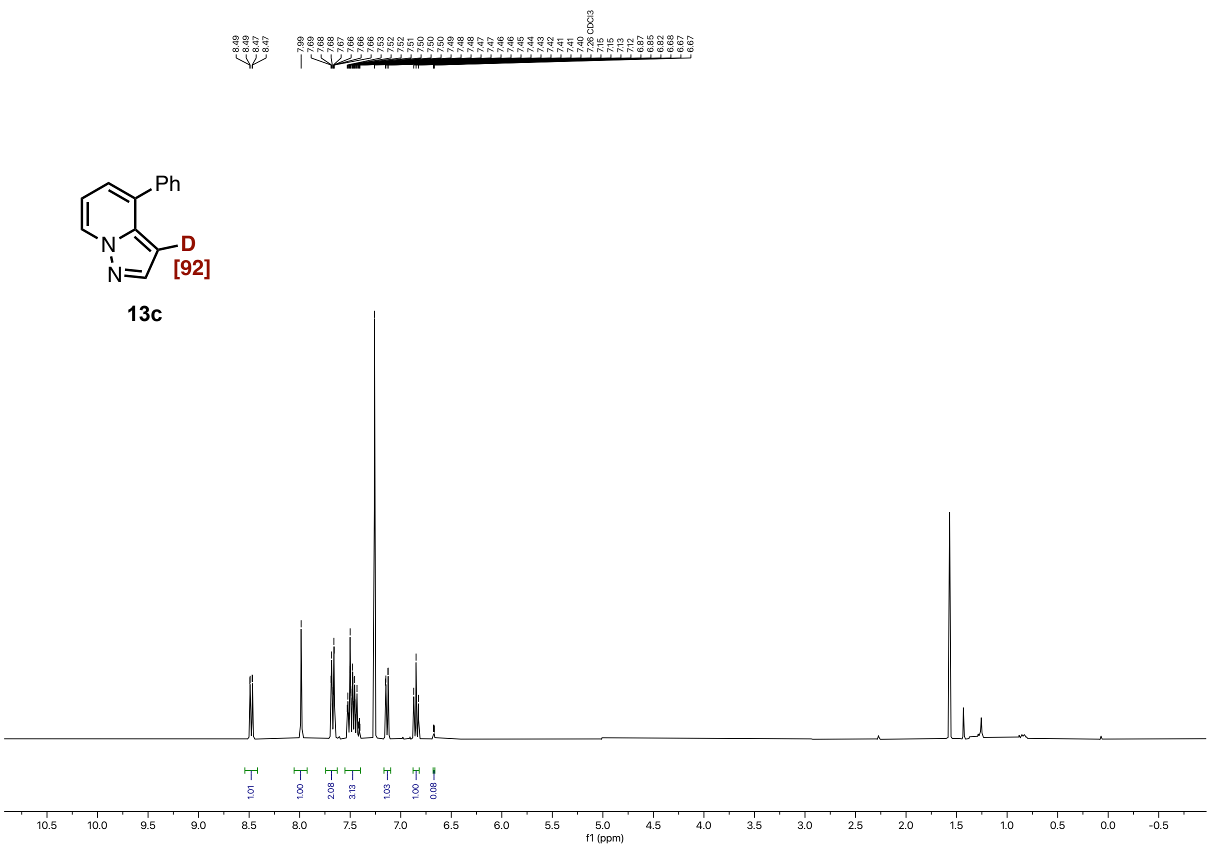

^{1}H spectrum (300 MHz, CD_3Cl) of **13c**

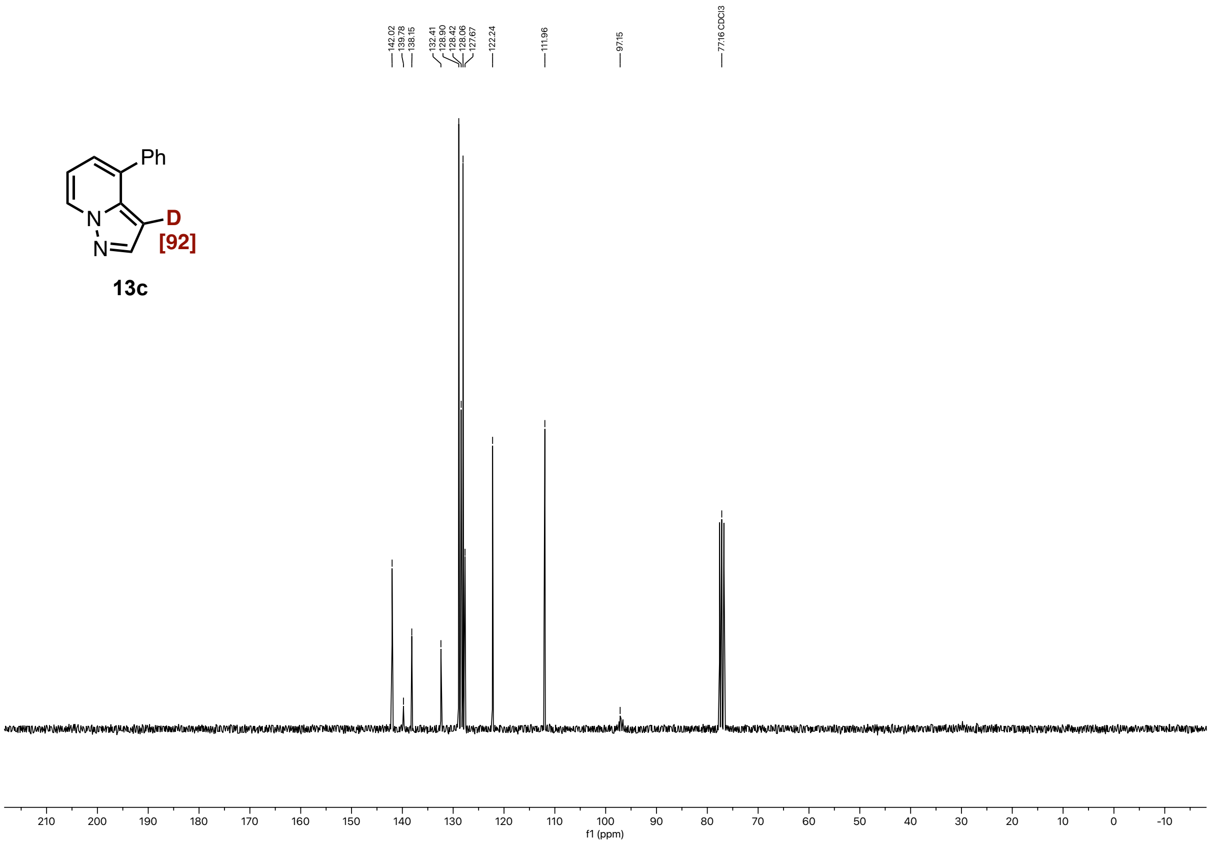

^{13}C spectrum (75 MHz, CD_3Cl) of **13c**

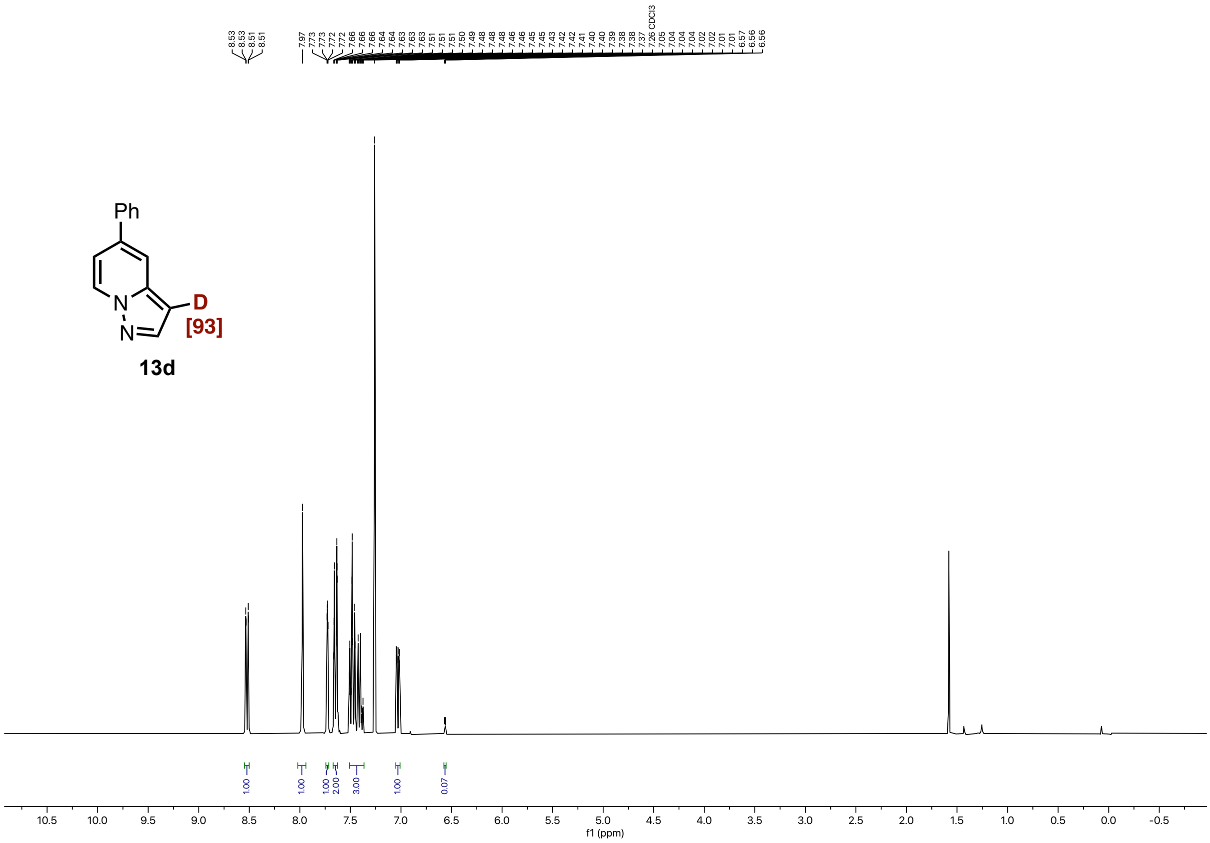

^{1}H spectrum (300 MHz, CD_3Cl) of **13d**

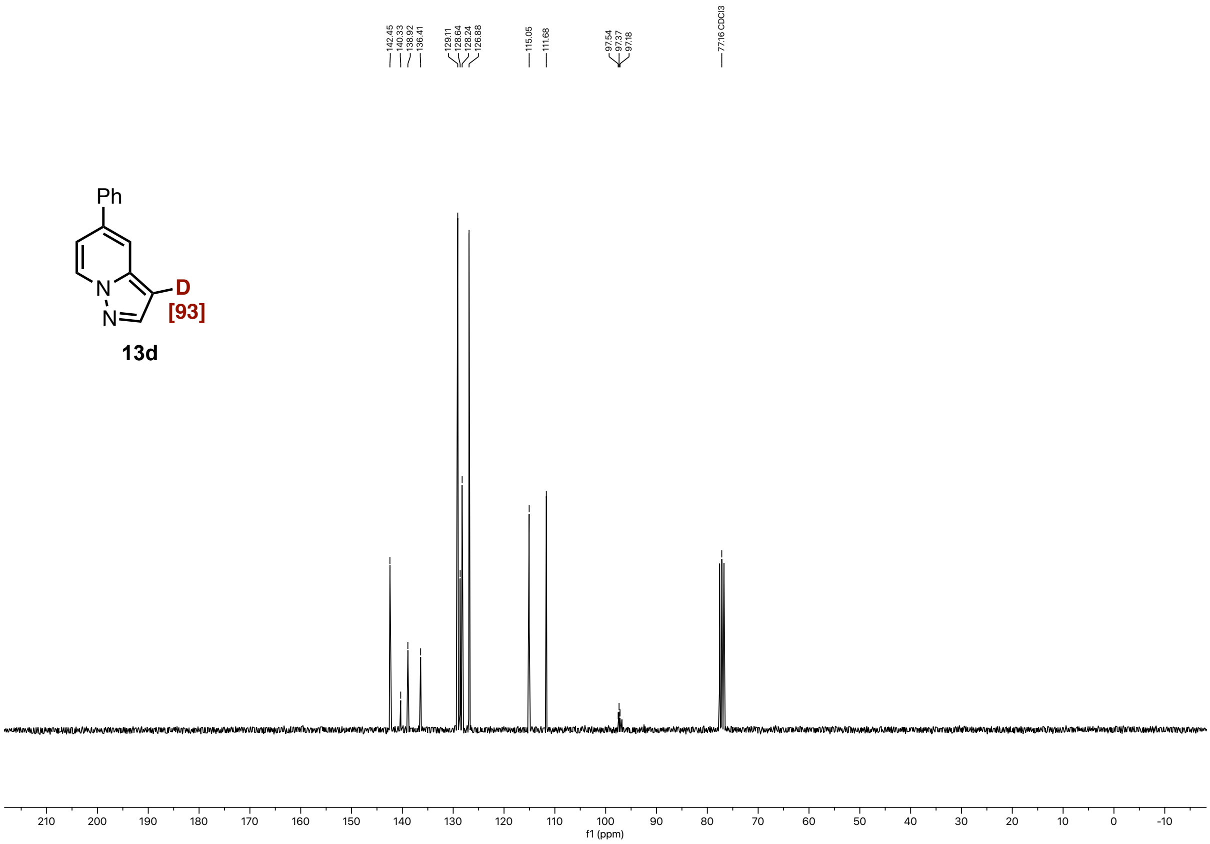

^{13}C spectrum (75 MHz, CD_3Cl) of **13d**

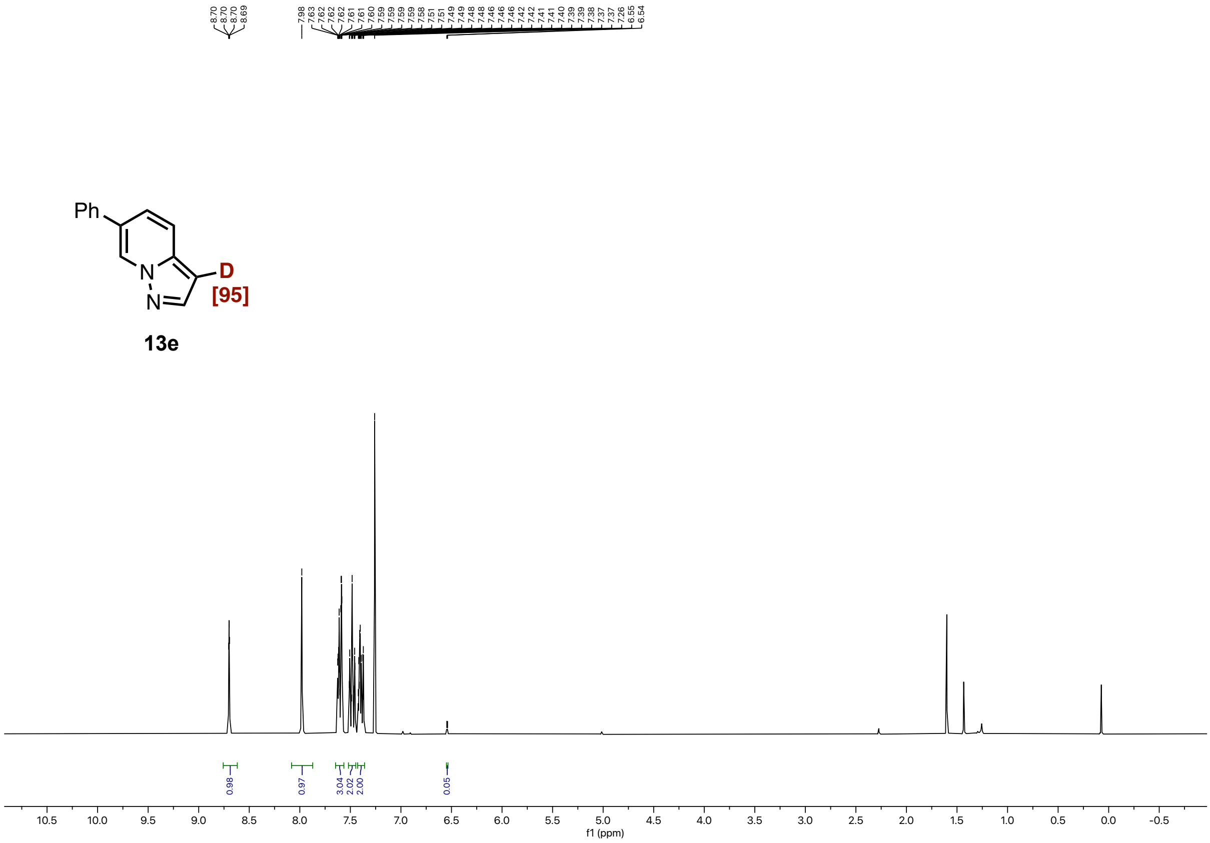

^{1}H spectrum (300 MHz, CD_3Cl) of **13e**

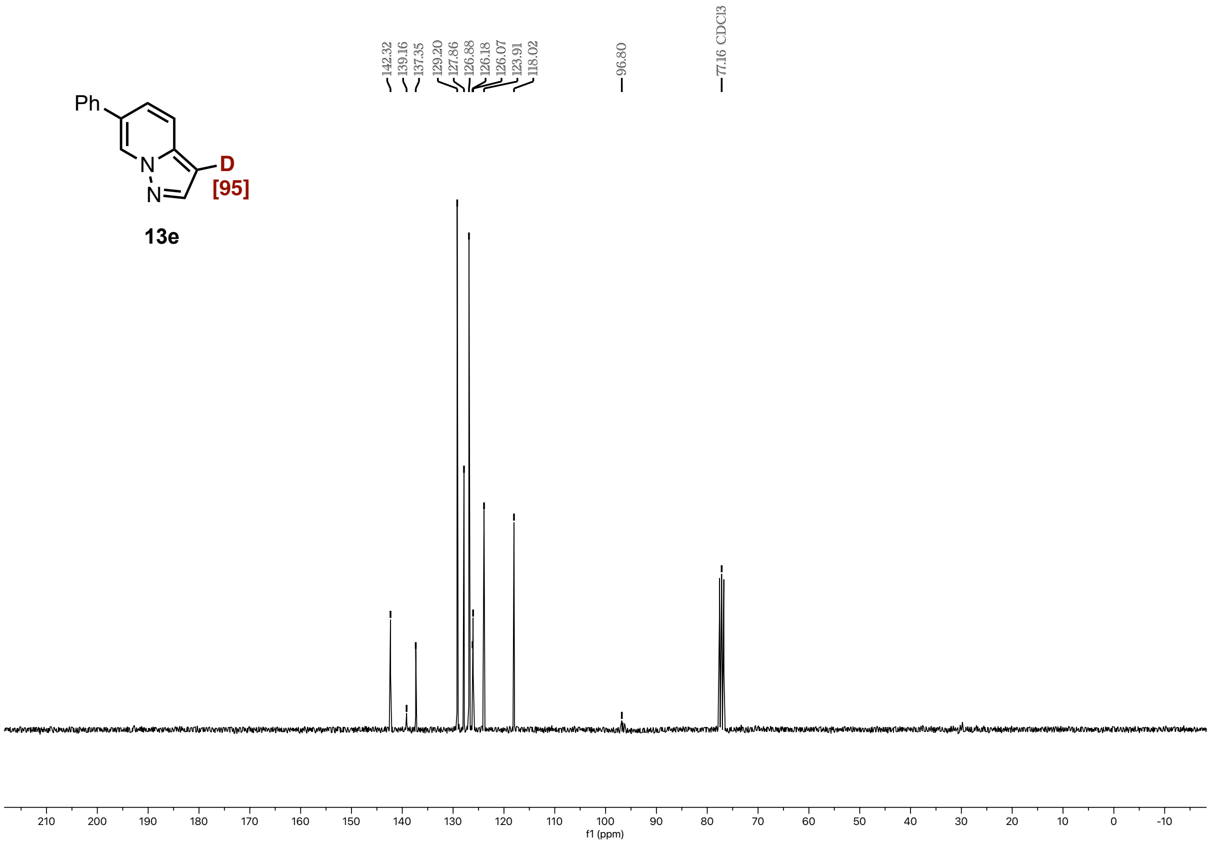

^{13}C spectrum (75 MHz, CD_3Cl) of **13e**

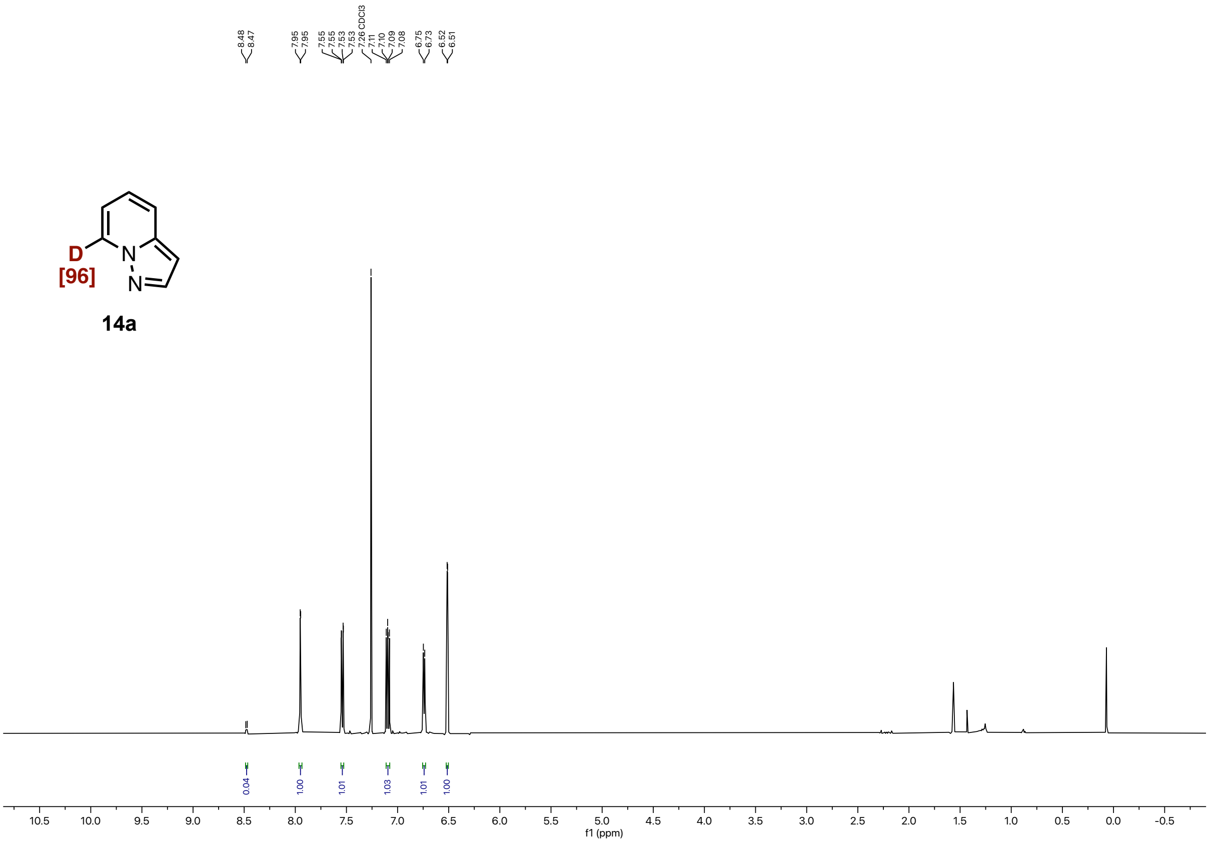

^{1}H spectrum (500 MHz, CD_3Cl) of **14a**

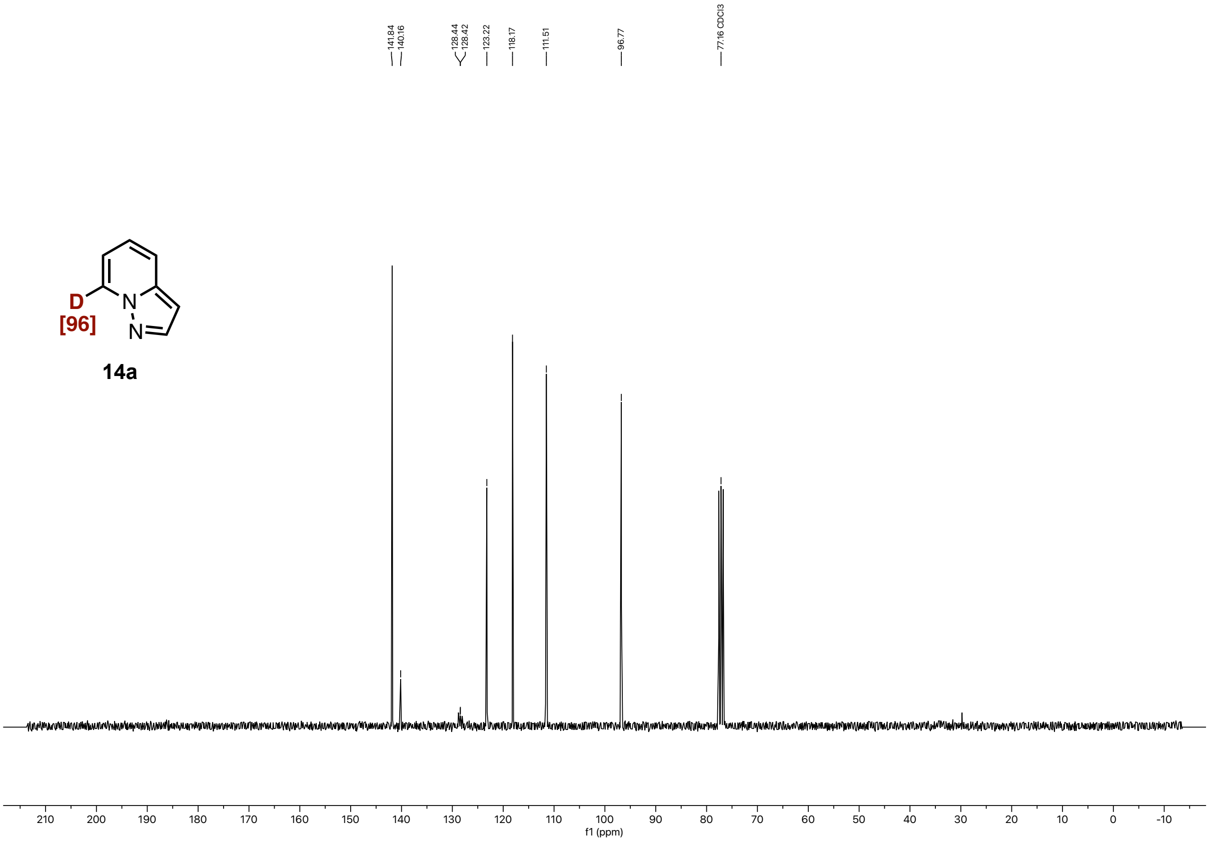

^{13}C spectrum (75 MHz, CD_3Cl) of **14a**

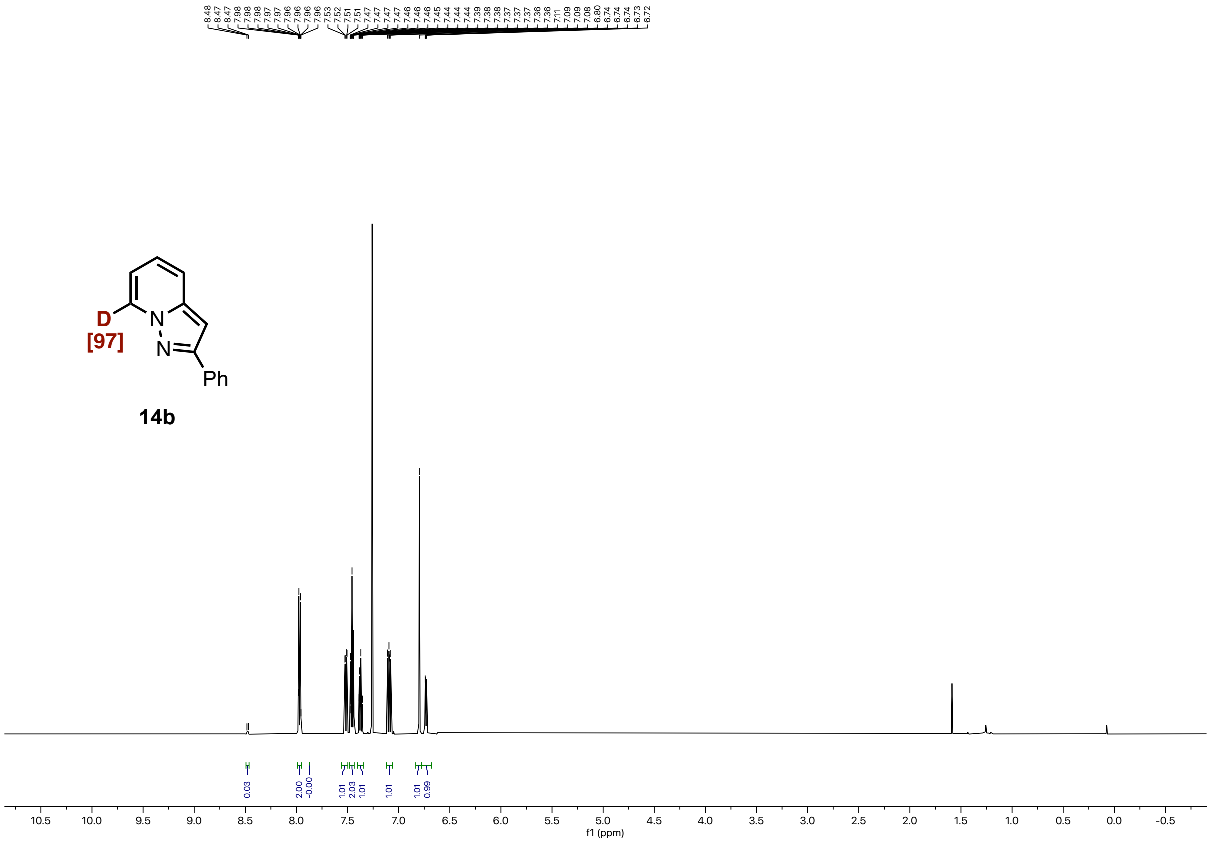

^{1}H spectrum (500 MHz, CD_3Cl) of **14b**

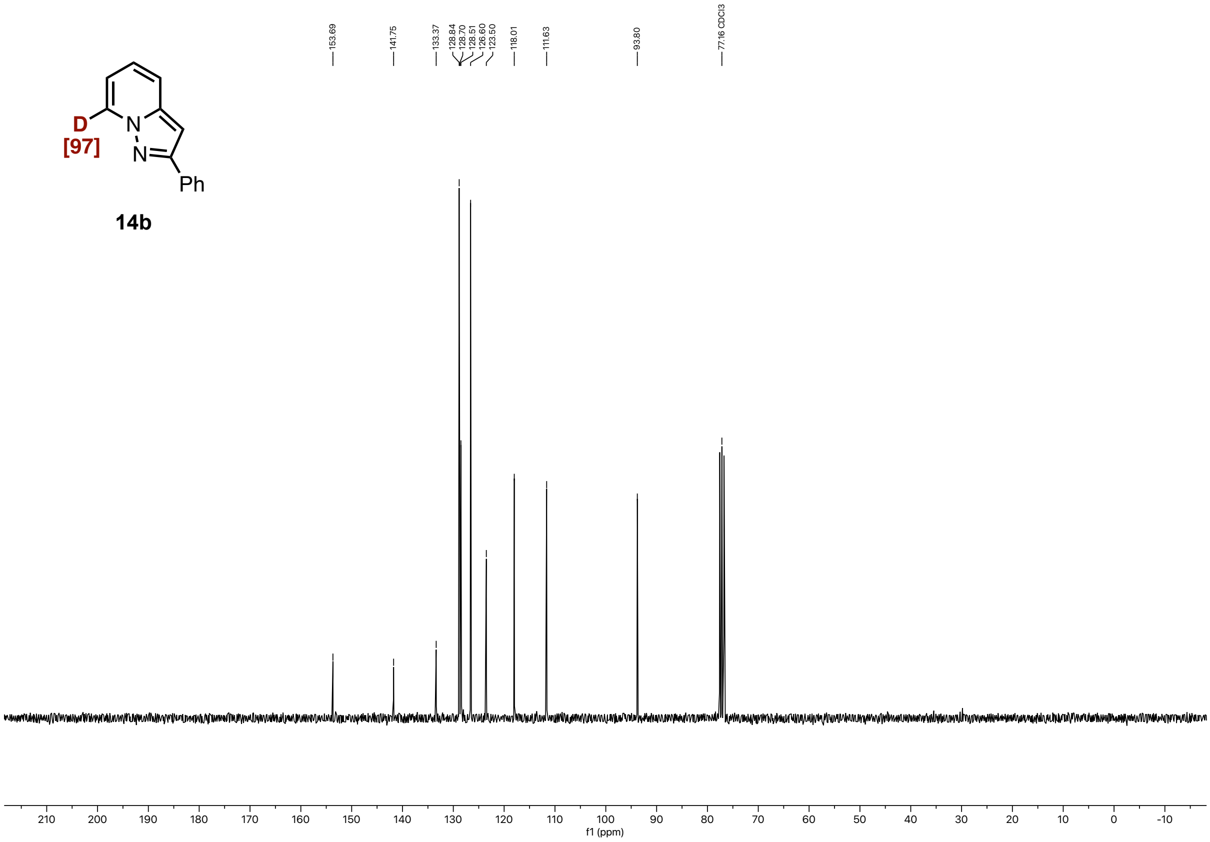

^{13}C spectrum (75 MHz, CD_3Cl) of **14b**

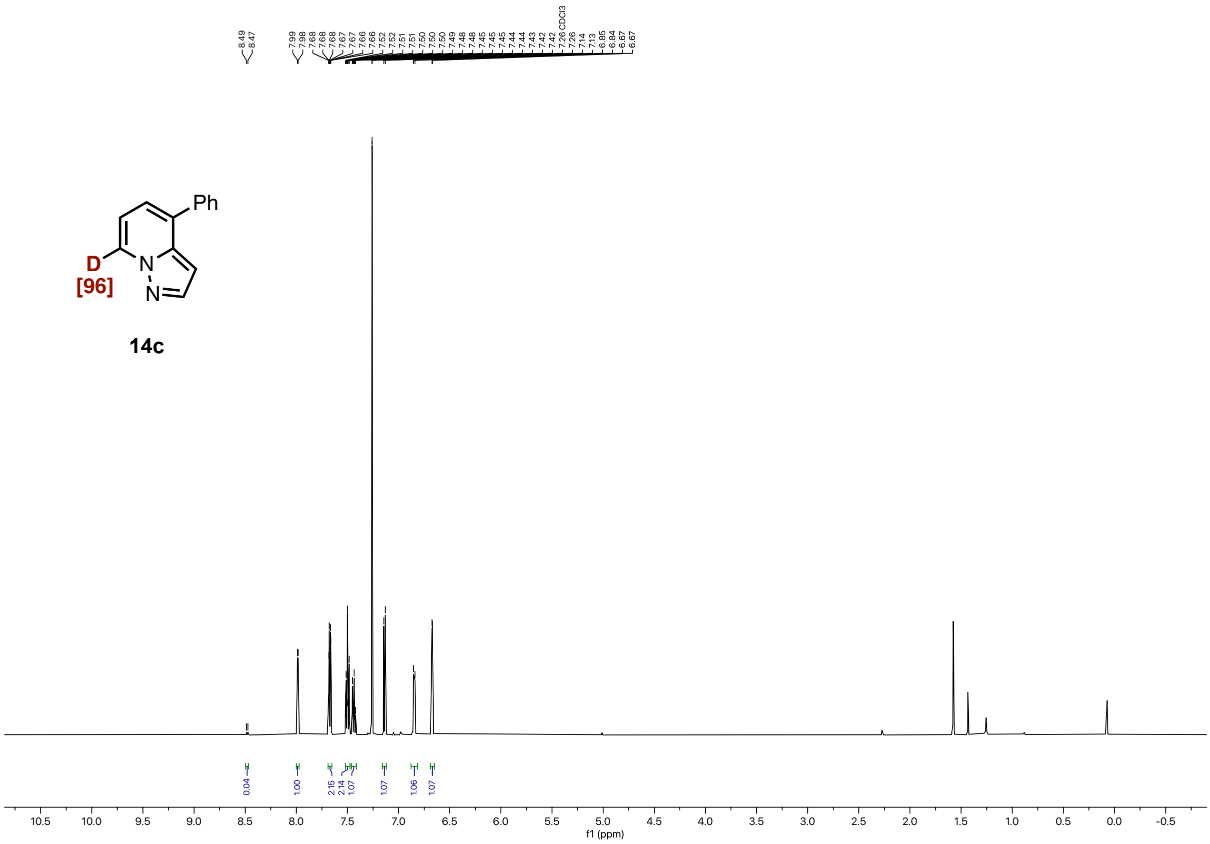

^{1}H spectrum (500 MHz, CD3Cl) of **14c**

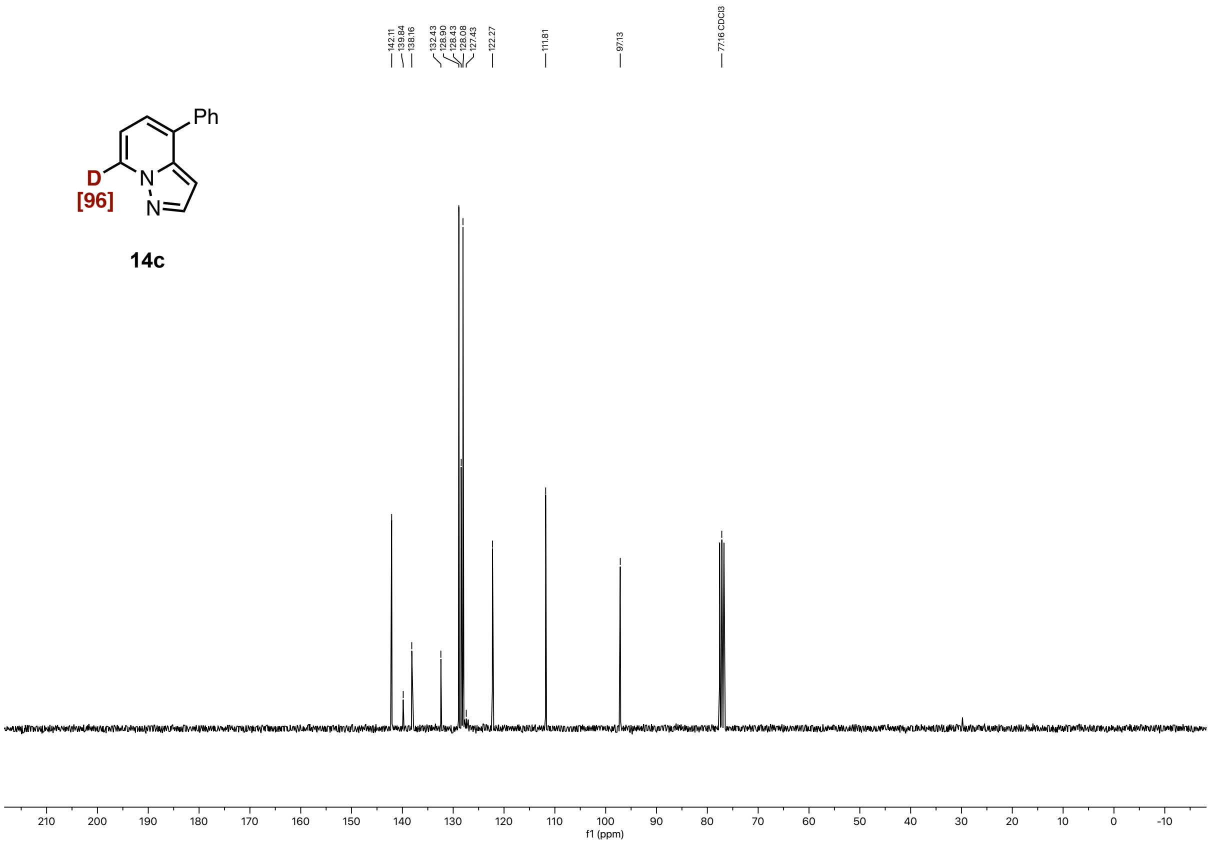

^{13}C spectrum (75 MHz, CD3Cl) of **14c**

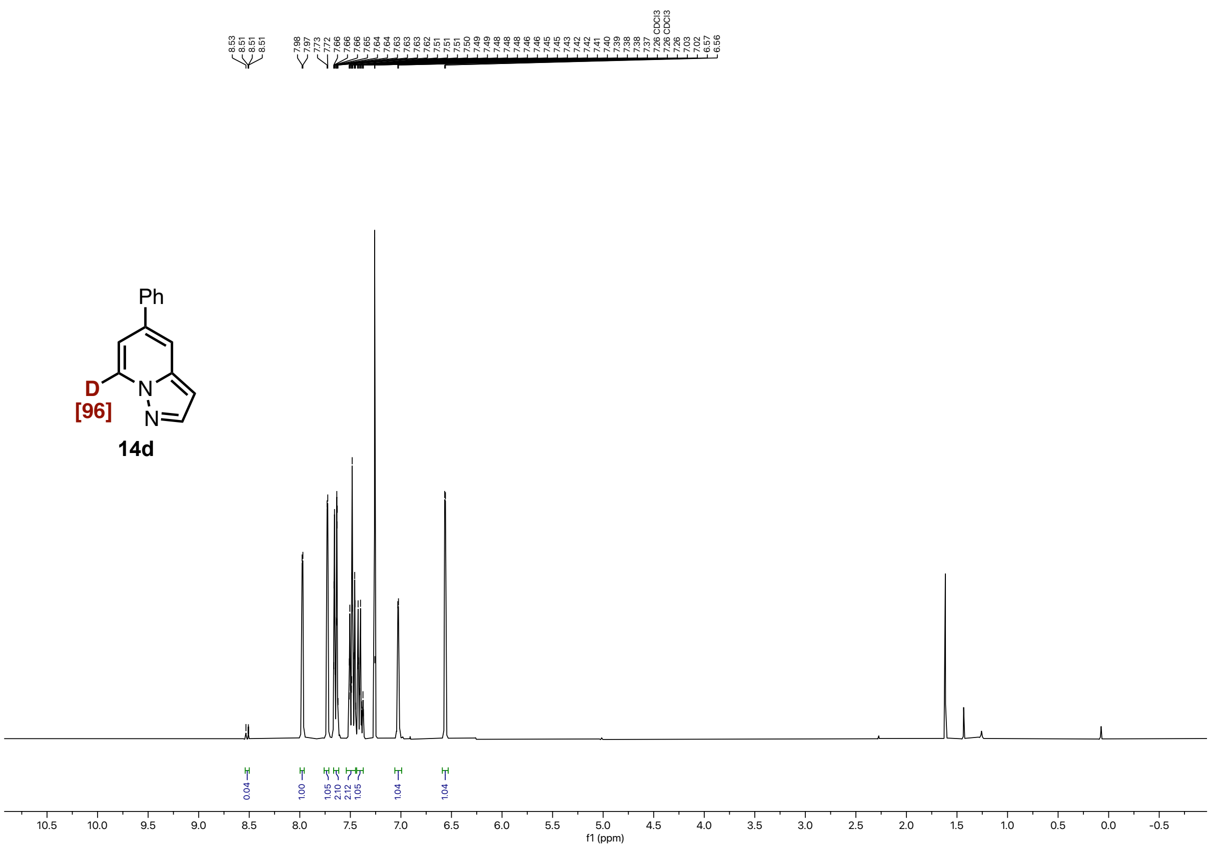

^{1}H spectrum (300 MHz, CD_3Cl) of **14d**

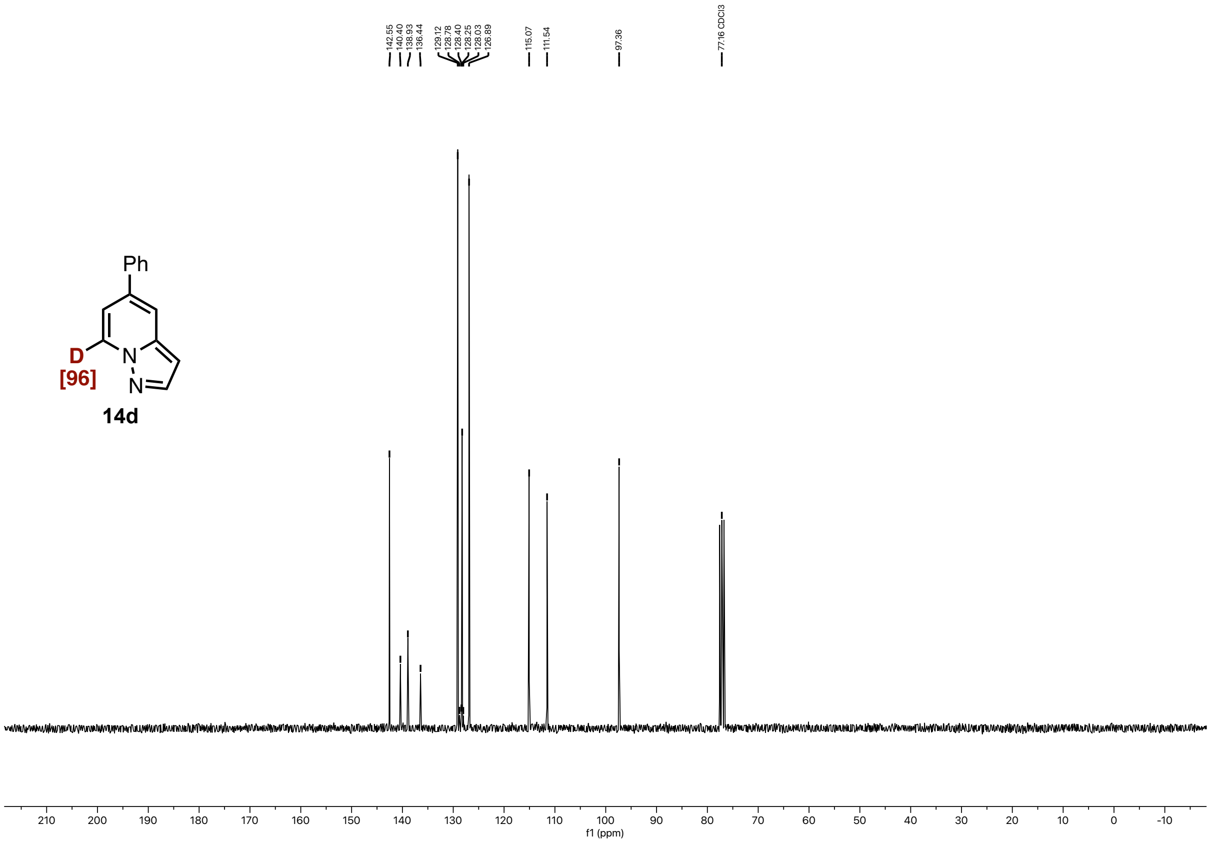

^{13}C spectrum (75 MHz, CD_3Cl) of **14d**

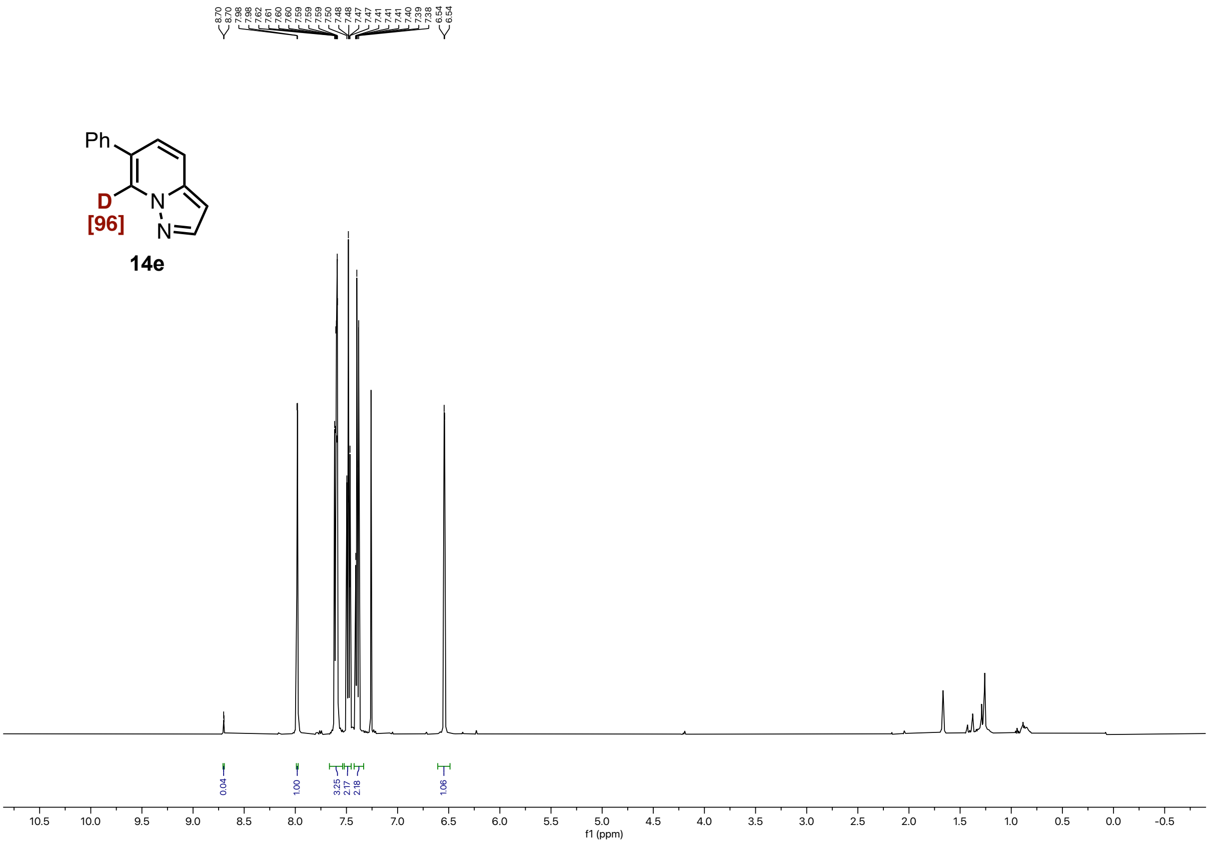

^{1}H spectrum (500 MHz, CD3Cl) of **14e**

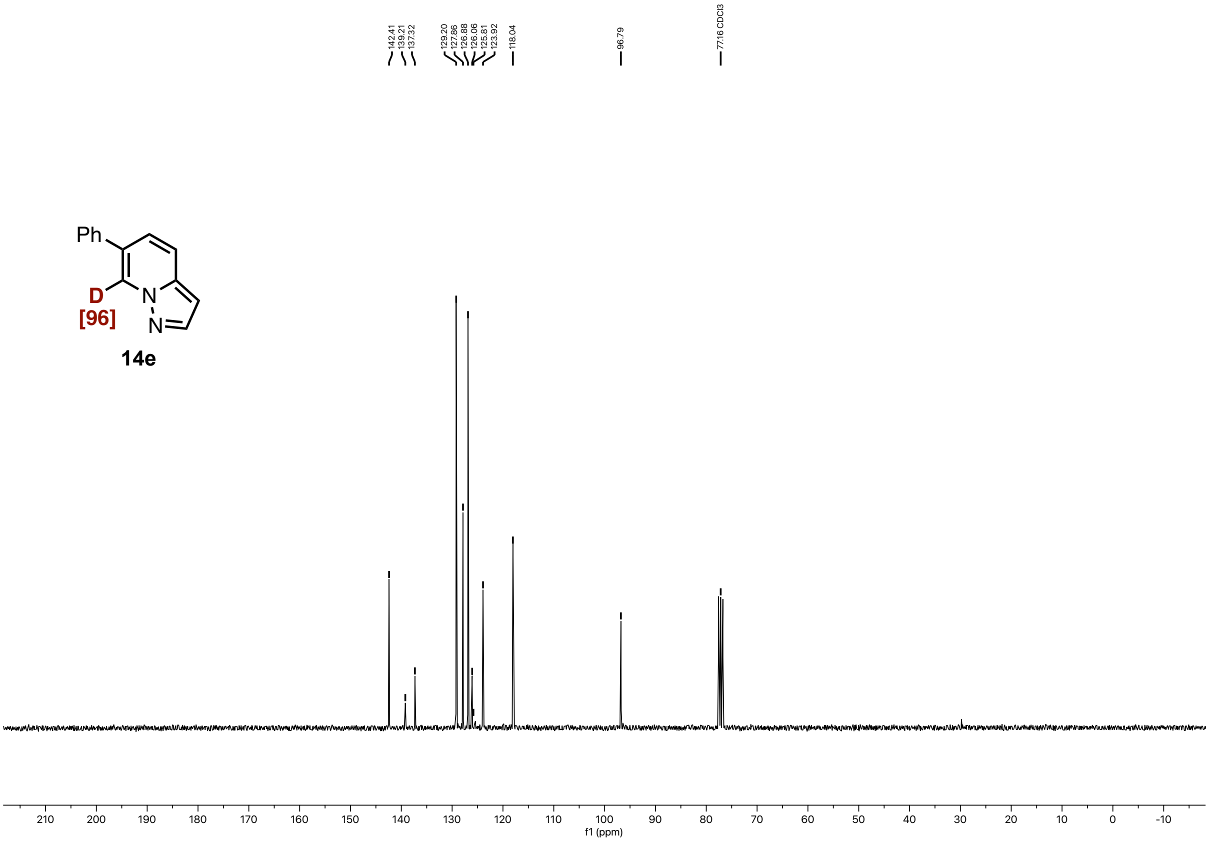

^{13}C spectrum (75 MHz, CD3Cl) of **14e**
